# Single-Cell Transcriptomics Reveals the Complexity of the Tumor Microenvironment of Treatment-Naive Osteosarcoma

**DOI:** 10.3389/fonc.2021.709210

**Published:** 2021-07-21

**Authors:** Yun Liu, Wenyu Feng, Yan Dai, Mengying Bao, Zhenchao Yuan, Mingwei He, Zhaojie Qin, Shijie Liao, Juliang He, Qian Huang, Zhenyuan Yu, Yanyu Zeng, Binqian Guo, Rong Huang, Rirong Yang, Yonghua Jiang, Jinling Liao, Zengming Xiao, Xinli Zhan, Chengsen Lin, Jiake Xu, Yu Ye, Jie Ma, Qingjun Wei, Zengnan Mo

**Affiliations:** ^1^ Department of Spinal Bone Disease, First Affiliated Hospital of Guangxi Medical University, Nanning, China; ^2^ Department of Trauma Orthopedic and Hand Surgery, First Affiliated Hospital of Guangxi Medical University, Nanning, China; ^3^ Center for Genomic and Personalized Medicine, School of Preclinical Medicine, Guangxi Medical University, Nanning, China; ^4^ Guangxi Key Laboratory for Genomic and Personalized Medicine, Guangxi Key Laboratory of Colleges and Universities, Nanning, China; ^5^ Guangxi Collaborative Innovation Center for Genomic and Personalized Medicine, Guangxi Medical University, Nanning, China; ^6^ Department of Bone and Soft Tissue Surgery, The Affiliated Tumor Hospital, Guangxi Medical University, Nanning, China; ^7^ School of Biomedical Sciences, The University of Western Australia, Perth, WA, Australia; ^8^ Department of Medical Oncology, First Affiliated Hospital of Guangxi Medical University, Nanning, China; ^9^ Guangxi Key Laboratory of Regenerative Medicine, Research Centre for Regenerative Medicine, Guangxi Medical University, Nanning, China

**Keywords:** single-cell RNA sequencing, tumor microenvironment, naive osteosarcoma, heterogeneity, osteolysis

## Abstract

Osteosarcoma (OS), which occurs most commonly in adolescents, is associated with a high degree of malignancy and poor prognosis. In order to develop an accurate treatment for OS, a deeper understanding of its complex tumor microenvironment (TME) is required. In the present study, tissues were isolated from six patients with OS, and then subjected to single-cell RNA sequencing (scRNA-seq) using a 10× Genomics platform. Multiplex immunofluorescence staining was subsequently used to validate the subsets identified by scRNA-seq. ScRNA-seq of six patients with OS was performed prior to neoadjuvant chemotherapy, and data were obtained on 29,278 cells. A total of nine major cell types were identified, and the single-cell transcriptional map of OS was subsequently revealed. Identified osteoblastic OS cells were divided into five subsets, and the subsets of those osteoblastic OS cells with significant prognostic correlation were determined using a deconvolution algorithm. Thereby, different transcription patterns in the cellular subtypes of osteoblastic OS cells were reported, and key transcription factors associated with survival prognosis were identified. Furthermore, the regulation of osteolysis by osteoblastic OS cells *via* receptor activator of nuclear factor kappa-B ligand was revealed. Furthermore, the role of osteoblastic OS cells in regulating angiogenesis through vascular endothelial growth factor-A was revealed. C3_TXNIP^+^ macrophages and C5_IFIT1^+^ macrophages were found to regulate regulatory T cells and participate in CD8^+^ T cell exhaustion, illustrating the possibility of immunotherapy that could target CD8^+^ T cells and macrophages. Our findings here show that the role of C1_osteoblastic OS cells in OS is to promote osteolysis and angiogenesis, and this is associated with survival prognosis. In addition, T cell depletion is an important feature of OS. More importantly, the present study provided a valuable resource for the in-depth study of the heterogeneity of the OS TME.

## Introduction

Osteosarcoma (OS) is a highly aggressive malignant bone tumor that is associated with a high relapse rate in spite of the availability of treatment methods, comprising of combined treatment with surgery and multiagent chemotherapy (1). Survival rates have failed to show any marked improvement over the course of the last few decades. In addition, the etiology of OS remains unclear, although its pathology is characterized by the heavy infiltration of complex cells, including malignant mesenchymal tumor cells, immune cells, fibroblasts and vascular networks, suggesting the presence of a highly complicated tumor microenvironment (TME) (2, 3).

Substantial evidence has indicated that the biological behavior of tumor cells is heavily influenced by the TME (4). Bidirectional interactions between different types of tumor cells and the TME have been shown to enhance tumor progression on multiple levels (5, 6). Recent evidence has suggested that osteoclasts (OCs) are involved in OS-mediated osteolysis (7), macrophages are involved in a number of different mechanisms underpinning tumor biology (8), endothelial cells are involved in OS-mediated angiogenesis (9), and so on. Cancer-associated fibroblasts (CAFs) (4, 10), which are the main source of the collagen-producing cells, are involved in the growth and metastasis of solid tumors. However, the underlying mechanism remains unclear in OS. Therefore, an improved understanding of the interactions between the cell clusters and the TME should give rise to novel therapeutic opportunities; however, at the present time, conventional bulk next-generation sequencing techniques are limited in terms of their ability to resolve tumor subpopulations and the TME (11, 12).

Recent advances in single-cell genomics have provided powerful new tools for the exploration of genetic and functional heterogeneity (13), for the reconstruction of evolutionary lineages (14) and for the detection of rare subpopulations (15). Additionally, single-cell RNA sequencing (scRNA-seq) studies on human tumors have provided novel insights into tumor heterogeneity and distinct subpopulations, findings that have proven to be pivotal in elucidating tumor-associated mechanisms (16–18). In comparison with the existing research on solid tumors, however, relatively few studies have been published on the single-cell transcriptome of OS.

In order to help resolve this problem, the present study was designed to investigate intratumoral heterogeneity in OS. An unbiased approach was adopted that used scRNA-seq to characterize transcriptional changes and cellular heterogeneity in OS. An scRNA-seq atlas of OS was constructed, and important biological processes, including osteolysis, angiogenesis and T cell exhaustion, were identified in the TME of OS. The resultant findings of this investigation should help both in terms of elucidating the underlying biological mechanisms of OS, and in improving clinical treatment strategies for patients with OS.

## Materials and Methods

### Sample Collection and Tissue Dissociation

The present study was approved by the Ethics Committee of The First Affiliated Hospital of Guangxi Medical University (approval no. 2019KY-E-097). Samples of six patients diagnosed with OS (**Table S1**) were collected at The First Affiliated Hospital of Guangxi Medical University. The patients provided written informed consent, and agreed to donate specimens for the present study.

Fresh OS specimens were collected during surgery. None of the patients were treated with chemotherapy or radiation therapy prior to tumor resection. Tumor tissues were dissected at ~2 cm from the tumor edge, and placed in a solution containing Hank’s balanced salt solution (cat. no. 311-512-CL; Wisent Bio Products) and 1% antibiotic-antimycotic (cat. no. 15240062; Thermo Fisher Scientific, Inc.) on ice. The fresh samples were transported to the laboratory within 20 min.

### Single Cell Suspension Preparation

Following the removal of the fat, visible vessels and surrounding necrotic area, a small section of the tumor tissues was cut and rinsed with cold Dulbecco’s phosphate-buffered saline (DPBS; cat. no. C14190500BT; Thermo Fisher Scientific, Inc.). Subsequently, the tissues were cut into ~1 mm^3^ pieces, placed on ice, transferred to DPBS and washed twice with DBPS. Collagenase 2 (1mg/mL) was used to digest the tissues into a single-cell suspension for 45 min at 37°C. Following filtering of the cells using a 100-μm cell strainer in DPBS with 1% fetal bovine serum (FBS; cat. no. 10091148; Thermo Fisher Scientific, Inc.), the suspended cells were centrifuged at 300 × g for 5 min. After discarding the supernatant, red blood cells were removed using 1× red blood cell lysis buffer (10× diluted to 1×; cat. no. B250015; BioLegend, Inc.) for 5 min, and the cells were passed through a 40-μm cell strainer. Finally, the single-cell suspension was resuspended in DPBS with 1% FBS following two washes with DPBS. Cell viability was confirmed to be >80% in all samples using the Trypan Blue exclusion assay (0.4%; cat. no. 420301; Thermo Fisher Scientific, Inc.), and the cell suspensions were kept on ice prior to performing the next procedures.

### 10× Genomics Single-Cell 3’-mRNA Sequencing

The single cells ultimately obtained from each sample were loaded onto a 10× Genomics Chromium Single-Cell Chip, along with the single-cell master mix and single-cell 3’-gel beads (10×; Switchgear Genomics) to generate single-cell gel bead-in-emulsions (or GEMs). The OS samples were processed using 10× Genomics V3 barcoding chemistry kits, according to the manufacturer’s instructions. mRNA in droplets underwent reverse transcription reactions, and cDNA amplification was subsequently performed, according to the manufacturer’s instructions. The single-cell libraries were then sequenced on an Illumina HiSeq X Ten instrument (Illumina, Inc.).

### Quality Control and Cell-Type Recognition

The data quality control process was analyzed using the Seurat package (version 3.1.1; https://satijalab.org/seurat/install.html) (19, 20). Three cases of OS were merged using the Merge function. The single-cell data had a gene number <300 and >4,500; those with a mitochondrial gene number of >10% were considered to be low-quality cells, and these were directly filtered out. The Harmony package (version 1.0; https://github.com/immunogenomics/Harmony) was then used to eliminate the batch effect of the cellular data (21). Subsequently, primary cell cluster analysis was performed using the FindClusters function of the Seurat package (resolution = 0.15), and the visual clustering results were presented through performing uniform manifold approximation and projection (UMAP) dimension reduction analysis. The different cell types were subsequently analyzed as follows: i) myeloid cell data were extracted using the SubsetData function in the Seurat package, followed by the FindClusters function (resolution = 0.30); ii) osteoblastic OS cell data were extracted using the SubsetData function of the Seurat package, and the FindClusters function (resolution = 0.06) was subsequently used to perform cluster analysis again; iii) OC data were extracted using the SubsetData function in the Seurat package, and the FindClusters function (resolution = 0.10) was then used to perform cluster analysis again; iv) natural killer T (NK/T) cell data were extracted using the SubsetData function of the Seurat package, and the FindClusters function (resolution = 0.2) was subsequently used to achieve cluster analysis again; v) B-cell and plasma cell data were extracted using the SubsetData function in the Seurat package, and then the FindClusters function (resolution = 0.10) was subsequently used to achieve cluster analysis again; and vi) CAF data were extracted using the SubsetData function in the Seurat package, and then the FindClusters function (resolution = 0.04) was subsequently used to achieve cluster analysis again. Markers from all clusters were identified using the FindAllMarkers function of the Seurat package. Major cell types were annotated based on their respective gene expression levels in a known set of genes, as follows: osteoblastic OS cells (ALPL, RUNX2, IBSP) (22–24); myeloid cells (LYZ, CD68) (25); OCs (ACP5, CTSK) (26); CAFs (COL1A1, FAP, VIM) (27); NK/T cells (CD2, CD3D, CD3E, CD3G, GNLY, NKG7, KLRD1, KLRB1) (28, 29); endothelial cells (EGFL7, PLVAP) (30, 31); B cells (MS4A1, CD79A) (32, 33); and plasma cells (IGHG1, MZB1) (33, 34).

### RNA Velocity Analysis

RNA velocity is an indicator of dynamic changes in transcripts, and is able to predict changes in future cell states (35). To obtain the.loom files, velocyto.py (version 0.17.17) was provided with the barcode.tsv and.bam files, as well as the human genome annotation file, GRCH38-3.0.0. The.loom files were then uploaded to the R package (version 3.6.3) using the ReadVelocity function in the velocyto.R package (version 0.6.0). The following parameters were set: DeltaT = 1; kCells = 25; and fit.quantile = 0.02. Finally, the velocity vector arrows were projected onto the UMAP plot, which was obtained in the Seurat package.

### Pseudo-Time Trajectory Analysis

The evolutionary processes of myeloid cells, osteoblastic OS cells, OCs, NK/T cells and CAFs were analyzed using the Monocle3 package (version 2.14.0; https://coletrapnelllab.github.io/monocle3/). According to previous reports (36–38), Monocle3 was mainly used for the operation of the myeloid cells, osteoblasts (OBs), OCs, NK/T cells and CAFs in two steps. In the first step, cells were organized into potentially discontinuous trajectories. In the second step, genes were identified that varied in their expression over those trajectories. As for the order_cells parameter, the cell starting point that coincided with the result of RNA velocity analysis was selected. It should also be noted that, during the process of OC analysis, quasi-temporal analysis on six genes (ACP5, CTSK, ATP6V0D2, CD14, CD74 and HLA-DRA) was also performed.

### Functional Enrichment Analysis

Osteoblastic OS cells, CAFs, NK/T cells and myeloid cells underwent gene set variation analysis (GSVA; package version, 1.34.0) in order to identify which gene set was significantly enriched in each subset. All gene sets were downloaded from the Molecular Signatures Database, MSigDB (https://www.gseamsigdb.org/gsea/downloads.jsp), and GSVA was performed as previously described (39). Gene Ontology (GO) analysis (clusterProfiler package, version 3.14.3) was performed to detect which of the biological processes were significantly enriched in each subtype of B cells and plasma cells.

### Cell-Cell Communication Analysis

To detect possible interactions across diverse cell types, CellPhoneDB (a publicly available repository of curated receptors, ligands and their interactions) was used to analyze cell-cell communication based on ligands and receptors. CellPhoneDB is widely used in ligand and receptor studies for single-cell sequencing (40, 41). In order to study the molecular-interaction networks among cell types, CellphoneDB.py (version 0.22; https://github.com/Teichlab/cellphonedb) was used to analyze osteoblastic OS cells, OCs, CAFs, endothelial cells, macrophages, regulatory T cells (Tregs) and CD8^+^ T cells. The ligand-receptor pair data were filtered using P < 0.05 as a cutoff. Data of biological significance were then selected for presentation.

### Copy Number Variation Analysis and Identification of Malignant OBs

To identify the CNV values of osteoblastic OS cells as compared with other cells (myeloid cells 1/2, NK/T cells, plasmocytes and B cells), CNVs were calculated using the infercnv package (version 1.2.1), as described previously (42, 43). The comparative reference cells were myeloid cells, NK/T cells, plasma cells and B cells. Using single-cell sequencing data, CNVs of each cell type were calculated according to their expression levels. The following parameters were set: Cutoff, 0.1; cluster_by_groups = TRUE; denoise = TRUE; and HMM = TRUE. Parameters that are not otherwise mentioned were set as default parameters.

### Single-Cell Regulatory Network Inference and Clustering Analysis

SCENIC is a method for reconstructing gene regulatory networks and identifying stable cell states from scRNA-seq data (44). Transcription factor (TF) activity was calculated using SCENIC (version 1.1.3), as described previously (45). A read count matrix was inputted, with the cell types represented as columns and gene symbols shown in rows. The filtered matrix was used to establish gene regulatory networks and to determine cell states and regulatory factors. Subsequently, the Wilcoxon rank sum test was used to analyze the differentially activated TFs among the different cell types. TFs with an adjusted P-value <0.05 and logFC >0.1 were considered to be significantly upregulated.

### Data Acquisition and Correlation to Public Datasets

Transcriptome RNA-seq data and the clinical information pertaining to the corresponding OS cases were obtained from the TARGET database (https://ocg.cancer.gov/programs/target). A total of 88 samples were included in this dataset; of those samples, following the exclusion of incomplete survival data, 85 samples were included in the present study. To validate the significance of the osteoblastic OS cells’ subtypes, the TARGET OS cohort was divided into three subgroups using the class discovery tool, ConsensusClusterPlus (version 1.50.0). DESeq2 (version 1.26.0) was used to perform normalization and differential gene expression analysis (Cluster2/3 vs Cluster1), and the obtained differential gene expression (| log2FC | >1 and adjust P-value <0.05) was used for visualization and subsequent analysis (46). CIBERSORT (version 1.03) was used for estimating the abundance profiles of the osteoblastic OS cells in the 85 samples. After the calculations had been made, samples with P < 0.05 were used for subsequent analysis and figure display (**Figure S1D**). Mann-whitney test was used to evaluate whether the relative abundances and malignant gene scores of the osteoblastic OS cells’ subtypes in the different TARGET OS subgroups were revealed to be significantly different.

### Multiplex Immunofluorescence Staining

Multiplex immunofluorescence staining was processed using the TSA fluorescence kits, according to the manufacturer’s instructions (Panovue Co., Ltd.). Briefly, after having been subjected to high-temperature antigen retrieval, 3-μm thick sections were incubated with blocking solution for 10 min at 25°C, and subsequently, the primary antibodies were applied overnight at 4°C. The antibodies used in these experiments were as follows: anti-alkaline phosphatase (ALPL; rabbit; cat. no. MA5-24845; dilution, 1:200; Thermo Fisher Scientific, Inc.), anti-tumor necrosis factor superfamily member 11 (TNFSF11; also known as RANKL; rabbit; cat. no. PA5-110268; dilution, 1:200; Thermo Fisher Scientific, Inc.), anti-interferon (IFN)-induced protein with tetratricopeptide repeats 1 (IFIT1; rabbit; cat. no. 14769S; dilution, 1:500; Cell Signaling Technology, Inc.) and anti-CD68 (rabbit; cat. no. 76437T; dilution, 1:500; Cell Signaling Technology, Inc.). The tissues were then incubated with Polymer horseradish peroxidase (HRP)-anti-mouse/Rabbit IgG secondary antibody for 10 min at 25˚C. Subsequently, tissues were soaked with fluorophore working solution for 10 min. The sections were heat-treated after the application of each fluorophore and primary antibody, and then incubated with the secondary antibody and another fluorophore working solution. Tissue sections were counterstained with 4’-6’-diamidino-2-phenylindole (DAPI; Beijing Solarbio Science & Technology Co., Ltd.) for 5 min after all the antigens had been labeled. Finally, multi-layer TIFF images were obtained using the Axio Imager M2P Imaging System (Carl Zeiss AG) for further analysis.

### *In Vitro* Experiments

FBS, Eagle’s minimal essential medium with alpha modification (α-MEM), and Gibco™ Dulbecco’s modified Eagle’s medium (DMEM) were purchased from Thermo Fisher Scientific, Inc. Recombinant human macrophage colony-stimulating factor (M-CSF) and RANKL were purchased from R&D Systems, Inc, whereas the tartrate-resistant acid phosphatase (TRAP) staining kit was purchased from Sigma-Aldrich Corp. (St. Louis, MO, USA). The enzyme-linked immunosorbent assay (ELISA) kits were purchased from Elabscience Biotechnology Co., Ltd.

Conditioned medium of cancer cell lines was prepared as described previously (47). OS cell lines (MG63, U-2 OS, Saos-2, 143B, K2M2 and DLM8) were incubated in DMEM containing 10% FBS until the cells reached 80% confluence at 37°C. Subsequently, the DMEM was replaced with serum-free DMEM, and the cells were re-incubated at 37°C for 16 h. The supernatant of the culture medium was collected for subsequent OC culture. Bone marrow macrophages (BMMs) were cultured as described previously (48). In addition, BMMs were divided into conditioned medium of an OS cell line group (OSCM; 20%, v/v) and non-conditioned medium of an OS cell line group (NOSCM; 0%, v/v; note that the cell line group here comprised K2M2 and DLM8 cells). The cells of both the groups were fixed with 2.5% glutaraldehyde for 15 min, and the cells were subsequently subjected to TRAP straining. TRAP^+^-multinucleated cells (i.e., those with >3 nuclei) were counted as OCs.

Human umbilical vascular endothelial cells (HUVECs) were cultured as described previously (49). HUVECs were seeded into Matrigel™-coated 96-well plates (3×10^4^ cells/well) and the cells (MG63, U-2 OS, Saos-2 or 143B cells) were treated either with OSCM or NOSCM for 8 h. Subsequently, the total length and number of junctions were quantified using ImageJ software.

ELISA was performed to detect the expression of RANKL and VEGFA. The OSCM and NOSCM were collected for ELISA detection. The experiments were performed precisely following the instructions of the ELISA kit. Diluent (40 μl) and 10 μL of OSCM or NOSCM were added to the 96-well ELISA plate. After incubating at 37°C for 60 min, the ELISA plate was washed five times. Subsequently, biotinylated antibody (100 μl) was added to each pore, and the ELISA plate was incubated at 37°C for 60 min. Then, antibiotic-protein HRP solution (100 μl) was added to each pore. After a further incubation at 37°C for 30 min, the ELISA plate was washed five times. Subsequently, substrate solution (100 μl) was added into each pore, and the ELISA plate was incubated at 37°C for 15 min. Finally, the terminal solution (100 μl) was added to each pore, and the absorbance of the ELISA plates was measured at 450 nm.

### Statistical Analysis

All statistical analysis was performed using R package, version 3.6.3 (http://www.rproject.org). Not all violin plots are shown displaying each data point due to the overall distribution being obscured by a multitude of data points. P<0.05 was considered to indicate a statistically significant difference.

## Results

### Single Cell Expression Atlas of OS

**Figure 1:**
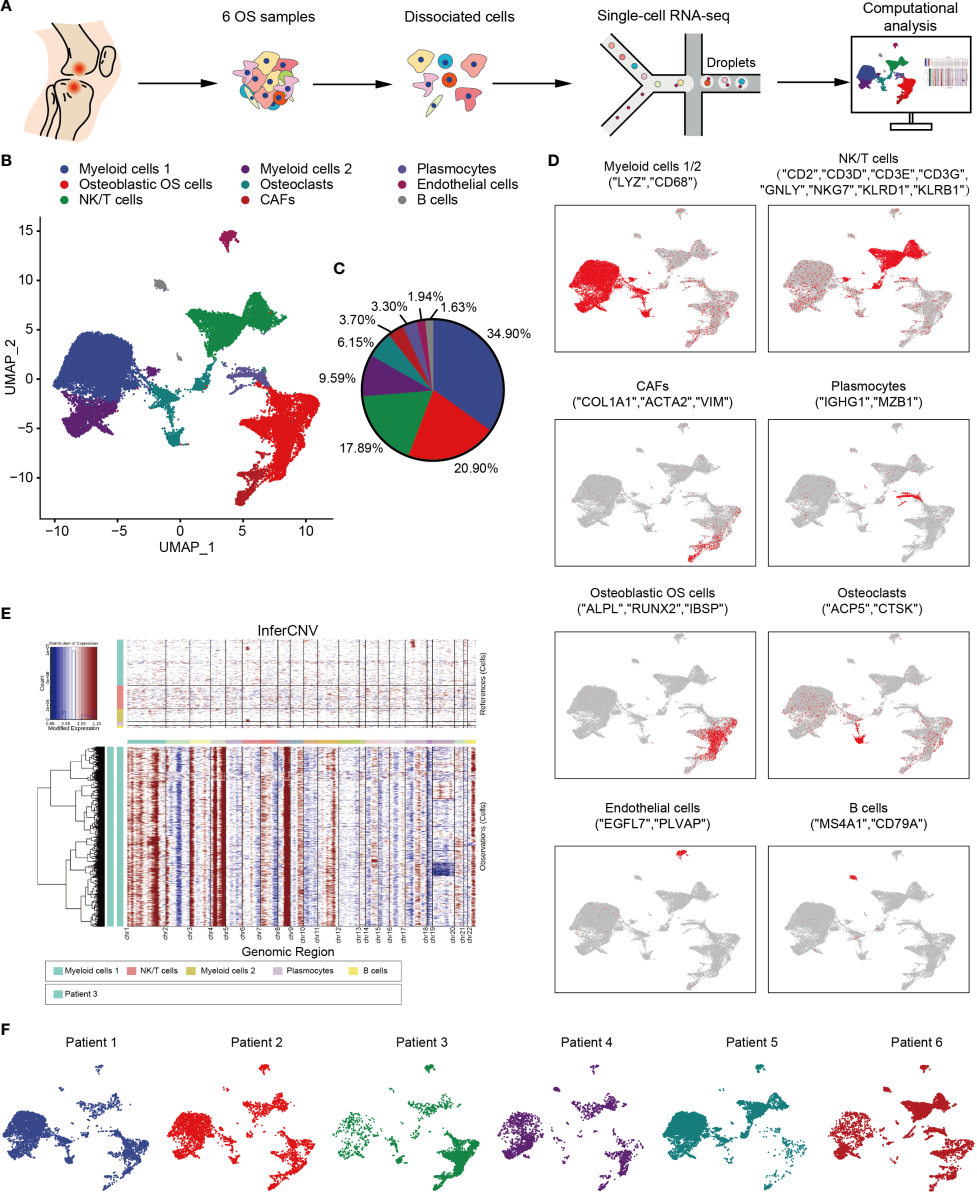
Overview of single cells derived from OS tissues. **(A)** Workflow depicting collection and processing of specimens of OS tumors for scRNA-seq. **(B)** UMAP plot of all the single cells, with each color-coded for the 9 major cell types. **(C)** Pie chart, indicating the cell composition of OS. **(D)** UMAP plots of the normalized marker expression of the 9 major cell types. **(E)** The large-scale chromosomal landscape in patient 3 was calculated using reference cells (myeloid cells 1/2, NK/T cells, plasmocytes and B cells); the red color represents an increased copy number, whereas the blue color represents a decreased copy number. **(F)** UMAP plot of all the single cells, with each cell color-coded for different patients. OS, osteosarcoma; UMAP, uniform manifold approximation and projection; scRNA-seq, single-cell RNA sequencing; NK, natural killer.

In order to explore the TME and cellular heterogeneity of OS, scRNA-seq analysis was conducted on primary tumors from six patients who had not received neoadjuvant chemotherapy (**Figure 1A**). After initial quality control checks had been accomplished, 29,278 cells were available. To investigate the cellular composition of OS, UMAP analysis was performed on differentially expressed genes across all cells, and this analysis led to the identification of nine main clusters (**Figures 1B, C**). The cell clusters were annotated based on the expression levels of specific marker genes, and immune (i.e., myeloid cells, NK/T cells, B cells and plasmocytes) and non-immune (i.e., osteoblastic OS cells, endothelial cells, OCs and CAFs) cells were identified (**Figure 1D**). In addition, with the identification of osteoblastic OS cells, large-scale chromosomal CNV analysis was performed for the six patients using reference cell myeloid cells, NK/T cells, B cells and plasmocytes (**Figures 1E** and **S1A**). This analysis revealed that, whereas the genomic regions of chromosomes 1, 4p, 4q, 8q and 11q were frequently increased in the OS cells, the 10 and 18 regions were frequently decreased, which was consistent with previous study (50). Among these cell types, the top three highest cell populations were found to be myeloid cells, NK/T cells and osteoblastic OS cells (**Figure 1C**). Almost all of the different cell types were identified in all six patients (**Figure 1F**).

### The Important Role of Inflammation and Ossification Associated Osteoblastic OS Cells in Humans

**Figure 2:**
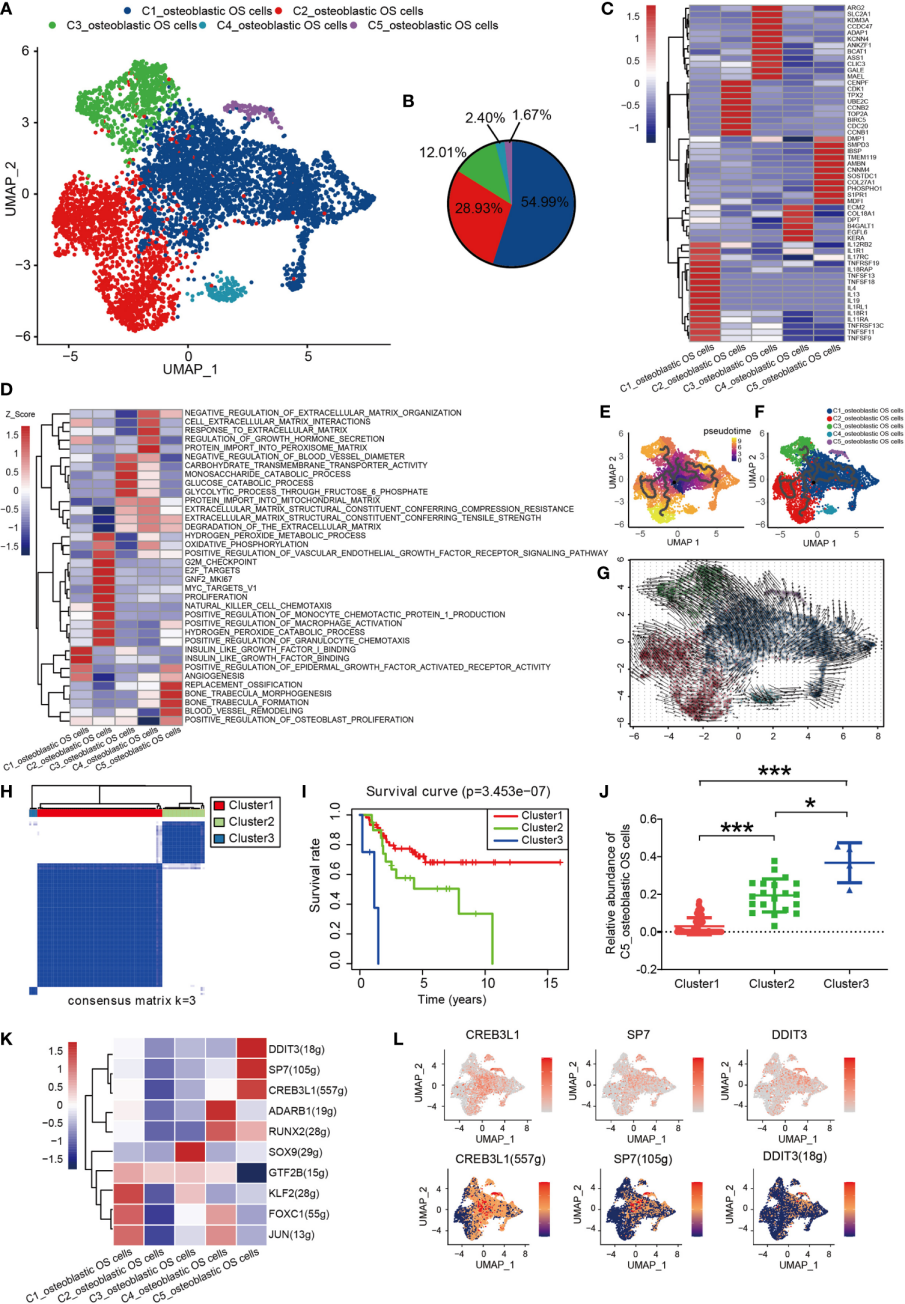
Heterogeneity of osteoblastic OS cell populations in OS. **(A)** UMAP plot showing osteoblastic OS cells. Different cell types are represented by the different colors. **(B)** Pie chart, indicating the cell composition of osteoblastic OS cells. **(C)** Heat map showing the marker genes of each cluster, with the selected osteoblastic OS cell marker genes in each cluster highlighted. **(D)** GSVA, showing the function of different types of osteoblastic OS cells. **(E, F)** Differentiation and developmental trajectories of osteoblastic OS cells in OS. **(G)** RNA velocity field projected onto the UMAP plot of the osteoblastic OS cells; the arrows indicate the direction of differentiation and the average velocity. **(H)** OS patients in the TARGET OS cohort were clustered into 3 clusters by ConsensusClusterPlus, based on cell clusters identified in this profile. **(I)** Kaplan-Meier survival curve of 3 patient clusters. **(J)** Relative abundance of C5_osteoblastic OS cell Clusters 1 (left), 2 (middle) and 3 (right). **(K)** Heatmap of the AUC scores of expression regulation by transcription factors estimated by SCENIC. **(L)** UMAP plot of osteoblastic OS cells, color-coded for the expression level (up) and for the AUC of the estimated regulon activity of these transcription factors (down). *P < 0.05,***P < 0.001; OS, osteosarcoma; UMAP, uniform manifold approximation and projection; GSVA, gene set variation analysis; SCENIC, single-cell regulatory network inference and clustering; AUC, area under the curve.

Osteoblastic OS cells were then subjected to UMAP dimension reduction analysis, and five distinct subgroups were identified: C1_osteoblastic OS cells, C2_osteoblastic OS cells, C3_osteoblastic OS cells, C4_osteoblastic OS cells and C5_osteoblastic OS cells (**Figures 2A, B**). In order to distinguish between the different cell types, an investigation of marker genes and GSVA were conducted. Based on the gene markers and GSVA, C1_osteoblastic OS cells were found to express inflammatory cell markers [including interleukin 17 receptor C (51), interleukin 4 (52), TNFSF11 (53), etc.] and were associated with inflammation. C2_osteoblastic OS cells, corresponding to a primordial proliferative osteoblastic population, were relatively more highly expressed compared with other specific gene markers [topoisomerase 2-alpha (TOP2A) (47), CENPF (54) and cyclin-dependent kinase 1 (CDK1) (55) that are associated with the cell cycle and cell proliferation. C3_osteoblastic OS cells were shown to express markers associated with cell metabolism [including arginase 2 (56), the glucose transporter solute carrier family 2 member 1 (57), argininosuccinate synthetase-1 (58), etc.], and they were also enriched in genes connected with carbohydrate transmembrane transporter activity and glucose catabolic processes. C4_osteoblastic OS cells, expressing cellular matrix markers [including extracellular matrix protein 2 (59), collagen type XVIII, alpha-1 (60), EGF-like domain multiple 6 (61), etc.], were enriched with genes responsive to the processes of extracellular matrix and protein import into the peroxisome matrix. C5_osteoblastic OS cells, corresponding to the original cluster of ossification osteoblastic cells, exhibited high expression levels of ossification markers [including sphingomyelin phosphodiesterase 3 (62), sclerostin domain-containing protein 1 (63), phosphoethanolamine/phosphocholine phosphatase 1 (64), etc.], and had a high level of genes involved in replacement ossification, bone trabecula morphogenesis and bone trabecula formation. In addition, C1_osteoblastic OS cells and C5_osteoblastic OS cells were associated with angiogenesis (**Figures 2C, D**).

To investigate the origin, differentiation and development of osteoblastic OS cells in the data of the present study, trajectory and RNA velocity analyses of osteoblastic OS cells were performed (**Figures 2E–G**). The results revealed the presence of partial C1_osteoblastic OS cells in the starting position of the developmental trajectory. This finding indicated that partial C1_osteoblastic OS cells have the ability to differentiate into other cell subtypes. The results from the multiplex immunofluorescence experiments also revealed the presence of C1_osteoblastic OS cells (**Figure S2A**).

In order to further explore the clinical significance of all osteoblastic OS cells, the OS dataset in the TARGET database was analyzed. The patients with OS were divided into three different clusters using ConsensusClusterPlus (**Figures 2H** and **S1E**). Compared with the OS patients from Cluster 1, the OS patients in Clusters 2 and 3 showed a worse prognosis (**Figure 2I**). Moreover, difference analysis was conducted on the samples of Cluster3/Cluster2 with worse survival and those of Cluster1 with an improved survival performance (**Figures S3A, B**), and 518 differential genes were obtained, which were mostly highly expressed in C5_osteoblastic OS cells (**Figure S3C**). Moreover, survival analysis of all 518 differential genes was conducted (**Supplementary Document 1**), and 85 genes with high expression were associated with poorer survival prognosis (**Supplementary Document 2**). Almost half of these 85 genes were highly expressed in C1_osteoblastic OS cells and C5_osteoblastic OS cells (**Figure S3D**). Next, it was found that C5_osteoblastic OS cells had the highest score after scoring the 85 malignant genes, and that of C1_osteoblastic OS cells was the second highest (**Figure S3E**). Interestingly, it was observed that C5_osteoblastic OS cells exhibited the most significant differences when comparing patients in Cluster 1 with the patients in Cluster 2/3. The number of C5_osteoblastic OS cells was increased in patients in Cluster 2/3 (**Figure 2J**). Furthermore, C1_osteoblastic OS cells exhibited the most significant differences when comparing patients in Cluster 1 with the patients in Cluster 2. It was found that the number of C1_osteoblastic OS cells was increased in patients in Cluster 2 (**Figure S3F**). These findings show that in our data, C5_osteoblastic OS cells and C1_osteoblastic OS cells have a malignant state, among which C5_osteoblastic OS cells have a more malignant state.

SCENIC analysis was performed to determine the TFs of osteoblastic OS cells. The genes of four TFs (DDIT3, SP7 and CREB3L1) were significantly activated in C5_osteoblastic OS cells, and these TFs have been shown to be expressed in osteosarcoma in previous studies (65–67) (**Figures 2K, L** and **S1B**). Further survival analysis revealed that a high expression level of SP7 was associated with a worse prognosis (**Figure S1C**). In previous studies, SP7 was shown to induce the differentiation of mesenchymal stem cells into osteoblasts and determine the fate of osteoblasts (68). In addition, SP7 was associated with lymphatic metastasis and poorer survival in breast cancer (69). However, to the best of our knowledge, no relevant prognostic information has been reported in the study of osteosarcoma, and further studies are required.

### Different States of OCs in Human OS

**Figure 3:**
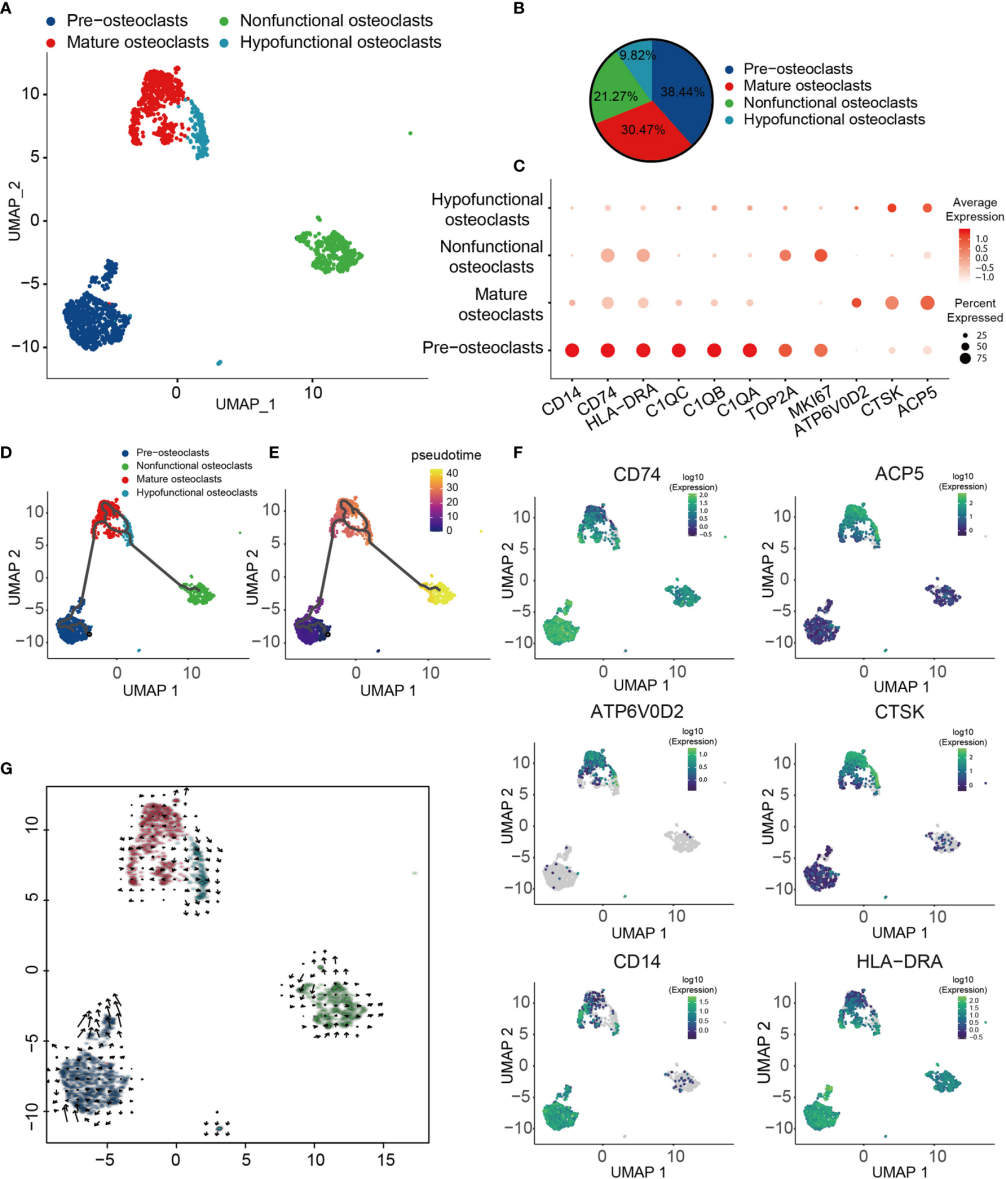
Heterogeneity of OC populations in human OS. **(A)** UMAP plot showing OCs, with different cell types represented by different colors. **(B)** Pie chart, indicating the cell composition of OC. **(C)** Dot-plot of marker genes in each cell subtype; shades of red represent the expression level, and dot sizes represent relative abundance. **(D, E)** Differentiation and developmental trajectories of OCs in OS, with different colors representing different cell subtypes. **(F)** Differentiation and developmental trajectories using marker gene expression (CD74, ACP5, ATP6V0D2, CTSK, CD14 and HLA-DRA). **(G)** RNA velocity field projected onto the UMAP plot of the OC; arrows indicate the direction of differentiation and the average velocity. OC, osteoclast; OS, osteosarcoma; UMAP, uniform manifold approximation and projection.

OCs have been shown to serve a crucial role in the pathogenesis of OS (70). To obtain a more comprehensive understanding of the types of OC in OS, four different types of OCs were obtained and renamed according to their specific gene expression profiles (**Figures 3A, B**). These four major cell types included progenitor, mature, hypofunctional and non-functional OCs. OCs were noted to be multinucleated giant cells derived from precursor cells of hematopoietic macrophage/monocyte lines (71, 72). Therefore, progenitor OCs were found to express high levels of major histocompatibility complex II (MHC-II; CD74), HLA-DRA, CD14, TOP2A and marker of proliferation Ki-67 (MKI67) (**Figure 3C**). Mature OCs expressed a higher D2 isoform of vacuolar (H^+^) ATPase, V0 domain (ATP6V0D2), cathepsin K (CTSK) and tartrate acid phosphatase (ACP5), which serve as OC differentiation marker genes (**Figure 3C**). Among them, ATP6V0D2 has previously been shown to be necessary for OC maturation, and it is highly expressed in OCs (73). The transcription profile of these cells was visualized, and through Monocle trajectory and RNA velocity analyses the cells were mapped along a pseudo-time trajectory to examine the profile’s directionality. OCs followed a differentiation trajectory that mainly started from the initial cluster of partial progenitor OCs, from which point they differentiated into the state of mature OCs, subsequently progressing from mature to hypofunctional or non-functional OCs (**Figures 3D, E, G**). Cell-trajectory analysis of marker genes also confirmed this result: CD74, CD14 and HLA-DRA were found to be located at the initial position of pseudo-time, and the expression levels of CTSK, ACP5 and ATP6V0D2 were shown to be gradually increased (**Figure 3F**).

### Functional Diversity of CAFs in Human OS

**Figure 4:**
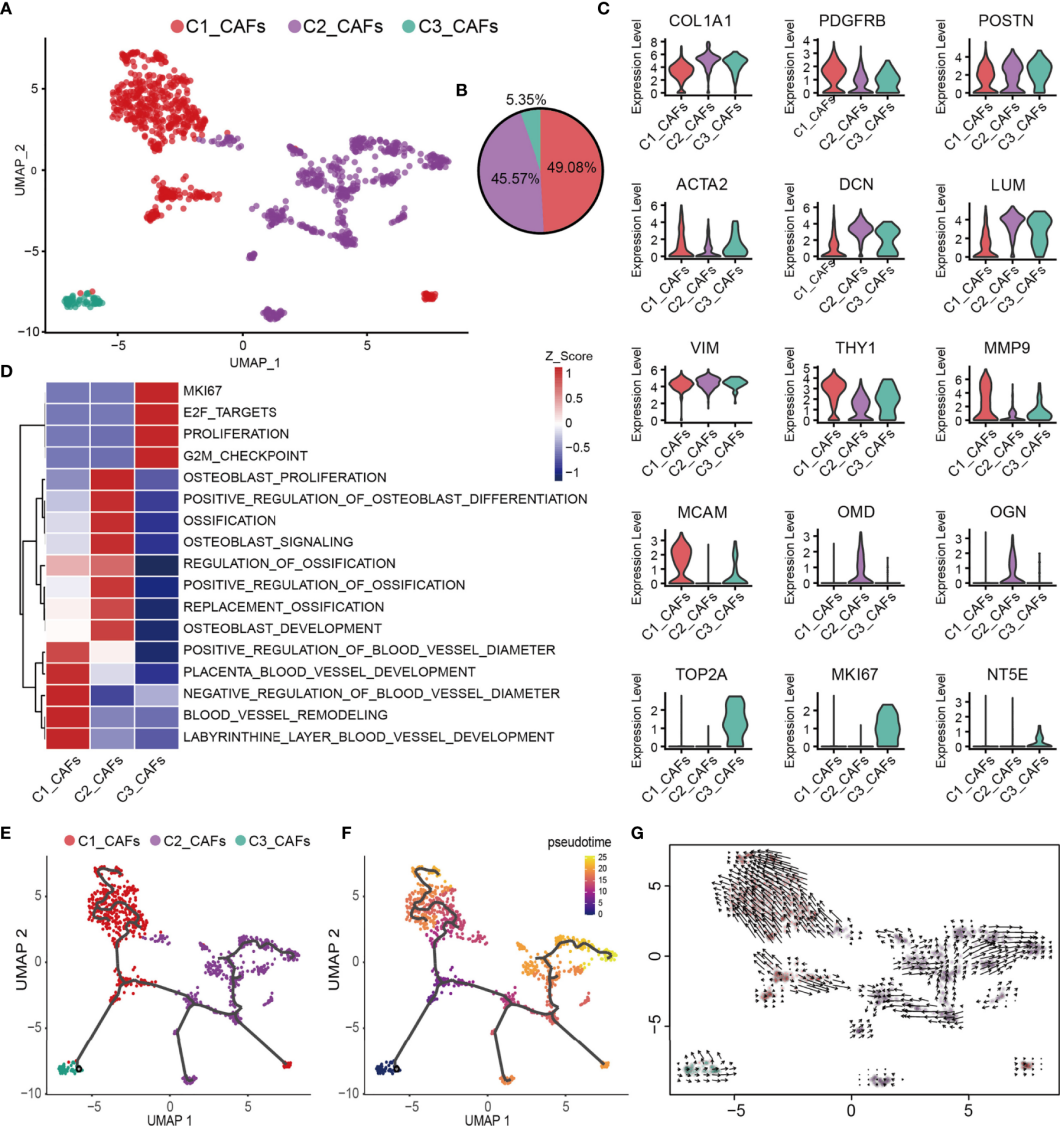
Heterogeneity of CAF populations in human OS. **(A)** UMAP plot showing CAFs, with different cell types represented by the different colors. **(B)** Pie chart, showing the cell composition of CAFs; different cell types are represented by different colors. **(C)** Violin plots showing relevant marker genes of CAF subtypes. **(D)** GSVA, showing the function of C1_CAFs, C2_CAFs and C3_CAFs. **(E, F)** Differentiation and developmental trajectories of CAFs in OS; different colors represent the different cell subtypes. **(G)** RNA velocity field projected onto the UMAP plot of the CAFs; arrows indicate the direction of differentiation and the average velocity. CAFs, cancer-associated fibroblasts; OS, osteosarcoma; UMAP, uniform manifold approximation and projection; GSVA, gene set variation analysis.

CAFs are one of the most abundant cell types in several different types of tumor, and accumulating evidence has suggested that they fulfill a fundamental role in influencing the malignant phenotype (74). To understand the heterogeneity of OS CAFs in more detail, transcriptomic data specific for CAFs were extracted from the total cell population, and a recombination analysis of the data was performed. UMAP analysis revealed that these cells formed three distinct subclusters with unique gene signatures (**Figures 4A, B**). Common CAF markers, including COL1A1, FAP, PDGFRB, POSTN, ACTA2, DCN, LUM, THY1 and the mesenchymal cell marker VIM, were expressed in all subgroups, which confirmed the identity of the cells as CAFs (**Figure 4C**). Based on the gene markers and GSVA, C1_CAFs were found to express tumor angiogenetic and invasion markers [including matrix metalloproteinase 9 and melanoma cell adhesion molecule] (75, 76) that are associated with vascular remodeling, vascular development and vascular diameter size. C2_CAFs exhibited high expression levels of ossification markers [including osteomodulin and osteoglycin], and were found to have high levels of genes involved in osteoblast proliferation, development and ossification. C3_CAFs expressed relatively higher levels of specific gene markers (TOP2A and MKI67), which are associated with the cell cycle and cell proliferation (**Figures 4C, D**). To investigate the origin, differentiation and development of CAFs in the present study, trajectory and RNA velocity analyses of CAFs were performed (**Figures 4E–G**). The results revealed the presence of partial C3_CAFs in the starting position of the developmental trajectory. This indicated that C3_CAFs have the ability to differentiate into other cell subtypes.

### Distinct Functional Composition of Myeloid Cells in the TME in OS

**Figure 5:**
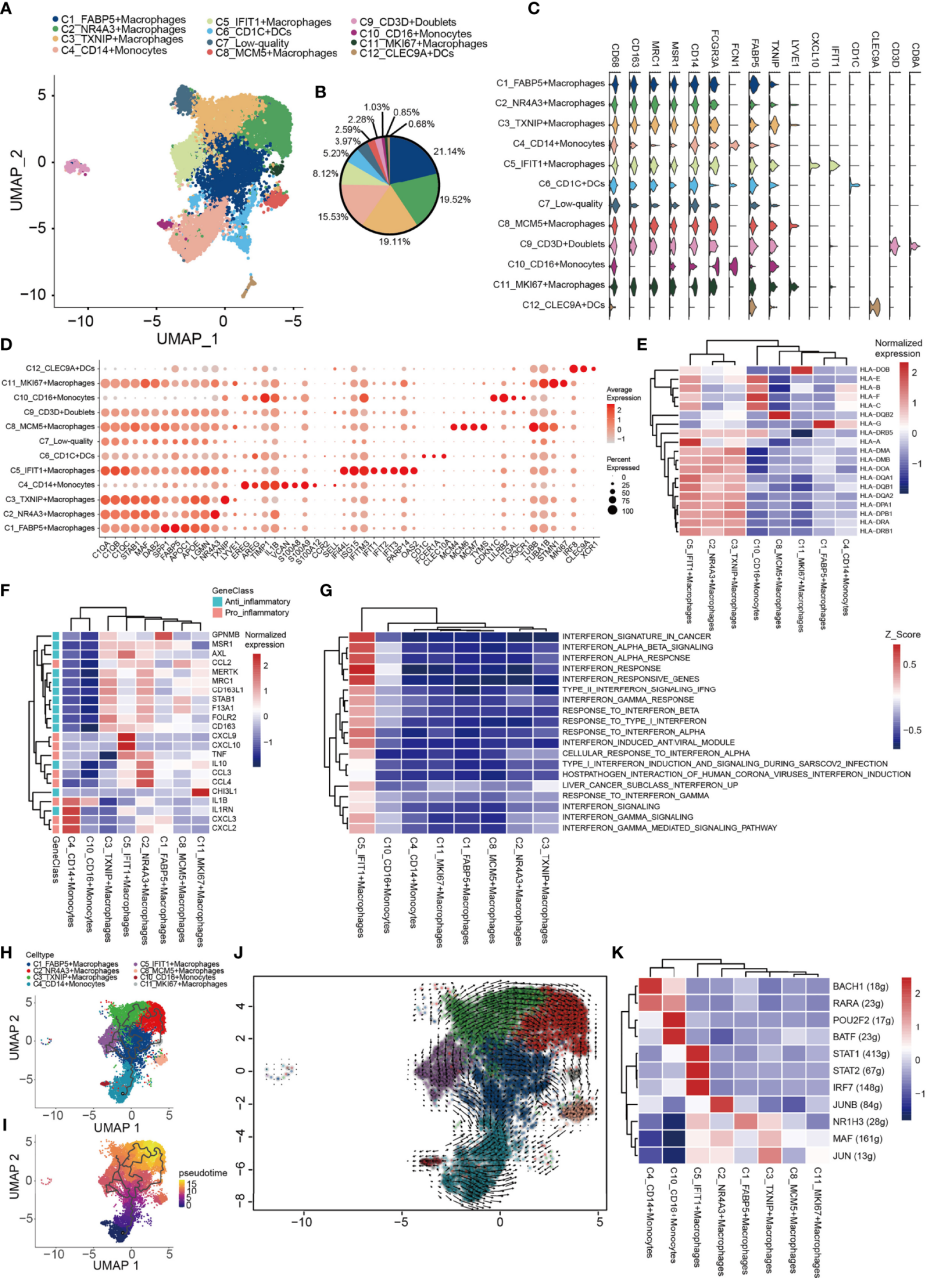
Heterogeneity of myeloid cells populations in human OS. **(A)** UMAP plot showing the myeloid cells, with different cell types represented by the different colors. **(B)** Pie chart showing the cell composition of myeloid cells, with different cell types represented by the different colors. **(C)** Violin plots showing relevant marker genes of the myeloid cell subtypes. **(D)** Dot-plot of marker genes in each cell subtype; shades of red represent the expression level, and dot sizes represent the relative abundance. **(E)** Heat map showing the encoding major histocompatibility complex class I and II molecules of the genes of each macrophage subtype. **(F)** Heat map showing the anti-inflammatory and pro-inflammatory genes of each macrophage subtypes. **(G)** GSVA showing the interferon-related functions of each macrophage subtype. **(H, I)** Differentiation and developmental trajectories of different cell subtypes in macrophages. **(J)** RNA velocity field projected onto the UMAP plot of the macrophages; arrows indicate the direction of differentiation and the average velocity. **(K)** Heat map of the AUC scores of expression regulation by transcription factors estimated by SCENIC. OS, osteosarcoma; UMAP, uniform manifold approximation and projection; GSVA, gene set variation analysis; SCENIC, single-cell regulatory network inference and clustering; AUC, area under the curve.

Subsequently, integrative unsupervised re-clustering of the myeloid cell populations from all six OS tissue samples was performed. From the total of 13,025 myeloid cells, 12 unaligned clusters were identified, including 2 monocyte (C4, C10), 6 macrophage (C1-C3, C5, C8, C11) and 2 dendritic cell (DC) (C6, C12) clusters (**Figures 5A, B**). A low-quality cluster (C7) and a myeloid/T-cell doublet cluster (C9_CD3D) are not discussed further in this article. Monocytes are differentiated from macrophages on the basis of low expression of specific macrophage markers [for example, CD68, macrophage scavenger receptor 1 and macrophage mannose receptor 1 (MRC1)] (**Figure 5C**). C4_CD14^+^ monocytes correspond to classical monocytes based on the high expression levels of the proteins CD14, VCAN and S100A8/9/12, and cells of this subtype are usually recruited during inflammation. The number of C10_CD16^+^ monocytes was most similar to that characteristically found for CD16^+^ patrolling monocytes, with a low expression of CD14 and high expression levels of Fc fragment of IgG receptor IIIa (also known as CD16) and other marker genes (CDKN1C, LILRB2, TGAL and CX3CR1) (77) (**Figures 5C, D**). Macrophages (C1–C3, C5, C8, C11) representing tumor-associated macrophages (TAMs) were identified in OS (**Figure 5A**). The C1_FABP5^+^ macrophages were characterized *via* their expression patterns of genes associated with lipid metabolism, including APOC1, APOE, LGMN and FABP5 (**Figures 5C, F**). The C2_NR4A3^+^ macrophage populations expressed both M1- and M2-type markers (CCL3, CCL4, TNF, AXL, CD163 and MRC1) (**Figures 5C, F**). C3_TXNIP^+^ macrophages were found to be similar to anti-inflammatory M2-polarized macrophages. These macrophages expressed highly both thioredoxin-interacting protein (TXNIP) and the M2 marker genes, MERTK, MRC1, STAB1 and CD163 (**Figures 5C, F**). C5_IFIT1^+^ macrophages expressed numerous type I interferon (IFN)-signaling/IFN-stimulated genes (ISGs) and pro-inflammatory genes, including CCL2, CCL3, CCL4, CXCL9, CXCL10 and TNF, whose proteins attract NK cells, T cells and immature DCs in the TME (**Figure 5F**). Importantly, these macrophages also highly expressed genes encoding MHC class I and II molecules, which specifically present antigens to cytotoxic CD8 T lymphocytes (**Figure 5E**). GSVA revealed that C5_IFIT1^+^ macrophages experienced an upregulation of the requisite signaling pathways in response to IFN-α and -β, and type II IFN (IFNG) (**Figure 5G**). These results indicated that C5_IFIT1^+^ macrophages may be derived from a proinflammatory microenvironment in the OS lesions under IFN stimulation. The multiplex immunofluorescence results also revealed the presence of C5_IFIT1^+^ macrophages (**Figure S2B**). C8_MCM5^+^ and C11_MKI67^+^ macrophages are two subtypes of macrophages that specifically express lymphatic vessel endothelial hyaluronan receptor 1 (LYVE1), but which show low expression levels of HLA-related genes, similar to the recently reported LYVE1^high^MHCII^low^ tissue-resident macrophages (78). Interestingly, C8_MCM5^+^ macrophages specifically express genes of the minichromosome maintenance (MCM) protein family, including MCM4, MCM5 and MCM7 (**Figure 5D**). It has been reported in the literature that MCMs are expressed in all cycling cells throughout the cell cycle, although they are lost in quiescent and differentiating cells (79). C11_MKI67^+^ macrophages were also observed to highly express cell-cycle-associated genes, including STMN1 and MKI67, suggesting that these macrophages may be tissue-resident cells that have the ability to proliferate. Subsequently, a trajectory analysis of the monocyte/macrophages based on the Monocle3 algorithm was performed. For monocyte/macrophages, the differentiation trajectory also exhibited a branched structure, starting with partial C4_CD14^+^ monocytes; next on the time scale were C1_FABP5^+^ macrophages, which further separated into C5 and C11, or C2 and C3 macrophages, suggesting that C1 macrophages are endowed with high plasticity prior to M2 differentiation (**Figures 5H–J**). Subsequently, the regulatory network that underlies the macrophages’ subset was examined using SCENIC, and specific TF regulons for the macrophages’ subset were identified (**Figure 5K**). The genes regulated by IRF7, STAT1 and STAT2 were upregulated in C5_IFIT1^+^ macrophages. STAT1 is the major transcriptional factor that controls the polarization of M1 macrophages (80). Collectively, in OS, all the macrophages were found to work together to create both an inflammatory and anti-inflammatory environment.

Finally, 765 DCs were also identified. C12_CLEC9As corresponded to conventional type 1 DCs (cDC1; CLEC9A and XCR1), and C6_CD1Cs corresponded to type 2 DCs [cDC2; CD1C, CLEC10A and Fc fragment of IgE receptor Ia (FCER1A)] (**Figure 5C**) (81, 82).

### Terminally Differentiated CD8^+^ T Cells Are Found in Human OS

**Figure 6:**
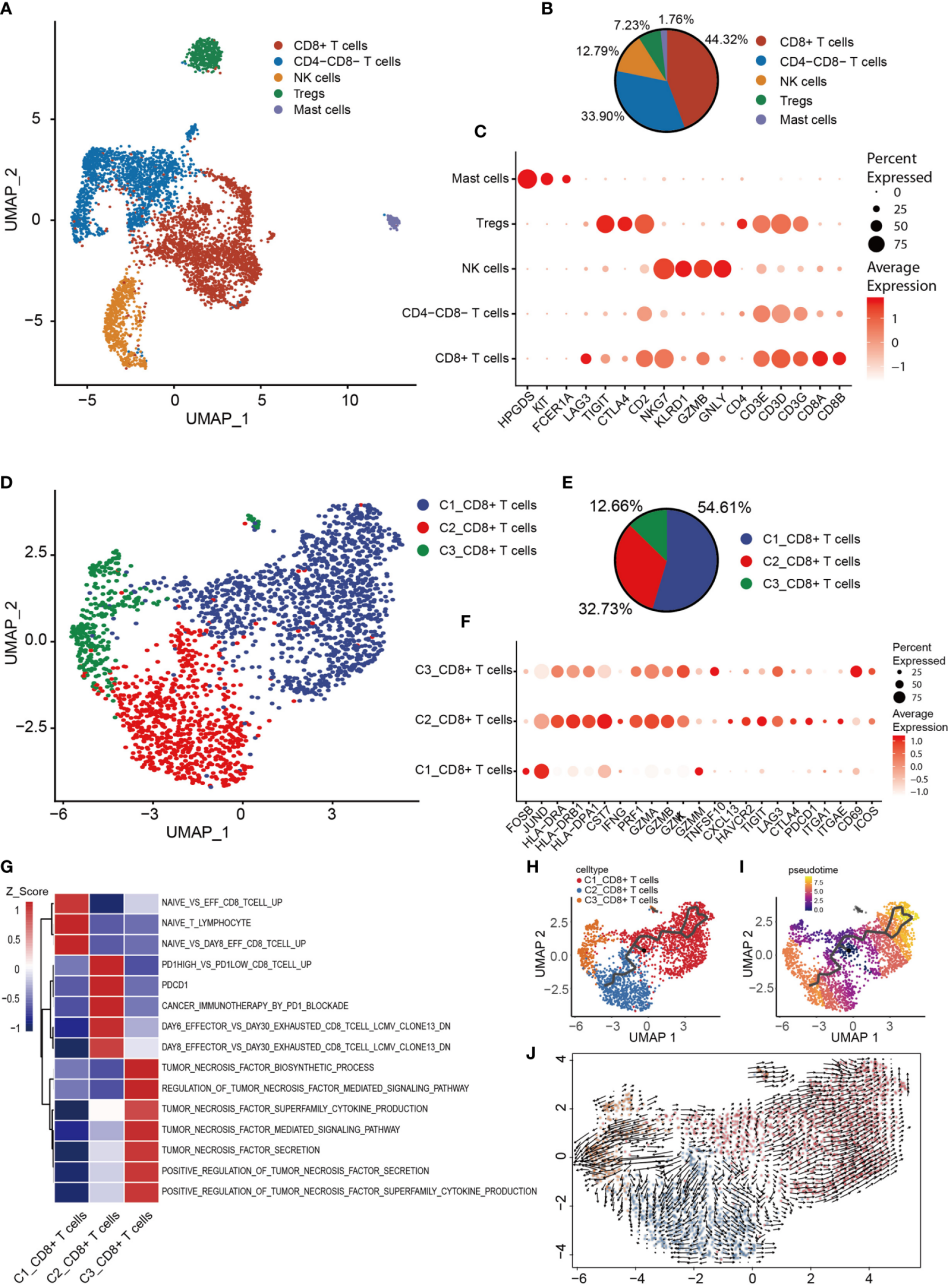
Heterogeneity of NK/T cells populations in OS. **(A)** UMAP plot showing NK/T cells, with different cell types represented by the different colors. **(B)** Pie chart, showing the cell composition of NK/T cells, with different cell types represented by the different colors. **(C)** Dot-plot showing the marker genes in each cell subtype of NK/T cells; shades of red represent the expression level, and dot sizes represent the relative abundance. **(D)** UMAP plot representing CD8^+^ T cells, with different cell types represented by the different colors. **(E)** Pie chart showing the cell composition of CD8^+^ T cells, with different cell types represented by the different colors. **(F)** Dot-plot showing the marker genes in each cell subtype of CD8^+^ T cells; shades of red represent the expression level, and dot sizes represent the relative abundance. **(G)** GSVA, showing the function of the different subtypes of CD8^+^ T cells. **(H, I)** Differentiation and developmental trajectories of CD8^+^ T cells, with different colors representing different cell subtypes. **(J)** RNA velocity field projected onto the UMAP plot of the CD8^+^ T cells; arrows indicate the direction of differentiation and average velocity. OS, osteosarcoma; UMAP, uniform manifold approximation and projection; GSVA, gene set variation analysis; NK, natural killer.

Based on T and NK cell signature gene markers (CD2, CD3E, CD3D, CD3G, NKG7, GNLY, KLRD1 and KLRB1), the T/NK cluster was thereby identified (**Figure 1E**). Subsequently, dimensionality reduction and clustering of the NK/T cells was performed. Overall, 5,239 NK/T cells were re-clustered into four subtypes (**Figures 6A, B**). CD8^+^ T, CD8^-^CD4^-^ T, NK, Treg and mast cells were identified based on the expression of the signature genes in each cluster (**Figure 6C**). Mast cells were identified based on the expression of characteristic genes [FCER1A, KIT (encoding a receptor tyrosine kinase) and hematopoietic prostaglandin D synthase]; the identification of the mast cells might have been obscured by the low-resolution setting previously, as mast cells were mixed in with NK/T cells.

To further clarify the characteristics of CD8^+^ T cells, CD8^+^ T cells were extracted for re-clustering, and 2,322 cells were subdivided into three groups (**Figures 6D, E**). Subsequently, the expression of selected T-cell function-associated genes was compared across the diverse T cell subtypes (**Figure 6F**). C1_CD8^+^ T cells were shown to be early-stage CD8^+^ T cells due to the increased expression of the early genes (JUND and FOSB) and low expression of cytotoxicity genes (GZMK, GZMA, GZMB and PRF1) (**Figure 6F**), whereas C2_CD8^+^ and C3_CD8^+^ T cells were identified as having high expression levels of cytotoxicity-associated genes, thereby revealing them to be cytotoxic T lymphocytes. C3_CD8^+^ T cells expressed the activated gene CD69 and the co-stimulatory gene, inducible T cell co-stimulator (**Figure 6F**), which indicated that C3_CD8^+^ T cells were cytotoxic T lymphocytes.

C2_CD8^+^ T cells express relatively high levels of genes associated with the immune checkpoint (PDCD1, CTLA4, LAG3, TIGIT and HAVCR2), and also CXCL13 and the tissue-resident genes, integrin subunit alpha-E (ITGAE) and integrin subunit alpha-1 (ITGA1) (**Figure 6F**), suggesting that C2_CD8^+^ T cells were in a state of exhaustion, consistent with the characteristics of exhausted T cells. GSVA revealed that C1_CD8^+^ T cells were enriched in a gene set associated with naive T cells, C2_CD8^+^ T cells were enriched in a gene set associated with exhausted T cells, and C3_CD8^+^ T cells were enriched in a gene set associated with TNF secretion (**Figure 6G**). The trajectory analysis and RNA velocity analysis of CD8^+^ T cells suggested that the differentiation trajectory began with the partial C1_CD8^+^ T cells, which bifurcated into C2_CD8^+^ T cells and C3_CD8^+^ T cells (**Figures 6H–J**). Essentially, these findings revealed that exhausted T cells were present in OS.

### Exploring the Unique Heterogeneity of B Cells in OS

**Figure 7:**
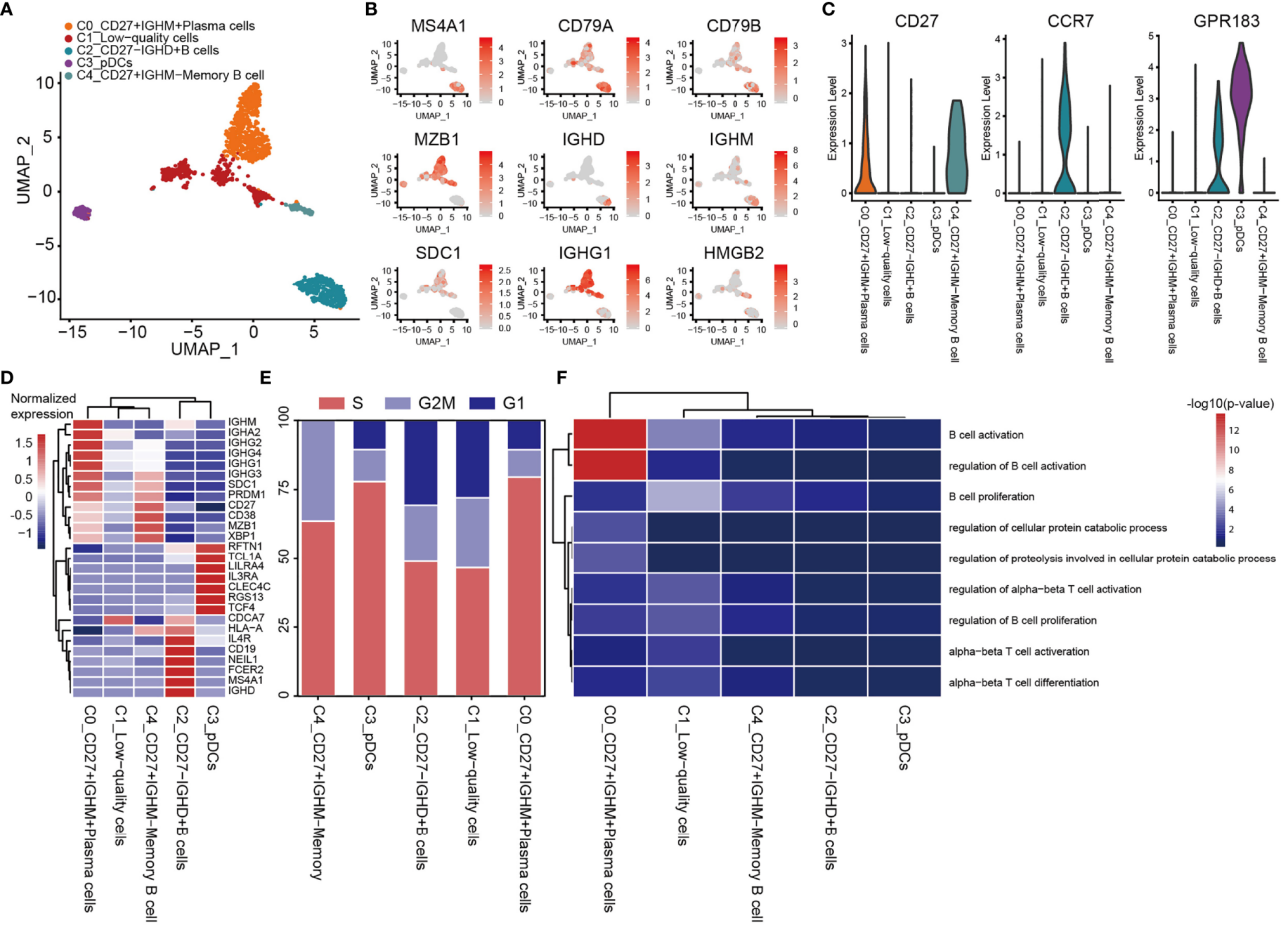
Heterogeneity of B cell populations in OS. **(A)** UMAP plot showing B cells, with different cell types represented by the different colors. **(B)** Cell types are defined by known genes the red color represents genes expressed, with gray representing no genes expressed. **(C)** Violin plots, showing relevant marker genes of B cells. **(D)** Heat map showing the marker genes of each cluster, with the selected B cell marker genes in each cluster highlighted. **(E)** Fractions of cells for the B cell subclusters are shown, with predicted cell cycle phases. **(F)** GO analysis, showing the function of subpopulations of B cells. OS, osteosarcoma; UMAP, uniform manifold approximation and projection; GO, gene onotology.

A total of 1,443 B cells were obtained, and these were grouped into 5 clusters by re-clustering. The C2 cluster of cells were follicular B cells (MS4A1/CD20 and CD79A/B), which were found in lymphoid follicles of tertiary lymphatic structure within the tumor, and C0 and C4 clusters were antibody-secretory cells (MZB1 and SDC1/CD138) (**Figures 7A, B**). CD27 is often used to identify the memory B cell population (83), and its role in signal transduction may facilitate the differentiation of memory B cells into plasma cells. CD27^+^ (C0 and C4) memory B cells and CD27^-^ naive B cells (C2_CD27^-^IGHD^+^ B cells; **Figures 7B, C**) were also observed. The CD27^-^ naive B cells have unique CD27^-^ IGHD (IgD)/IGHM (IgM) characteristics (**Figures 7B, D**), and are able to migrate through germinal centers (GCs) to produce CD27^+^ memory B cells. This migration of B cells in lymphatic follicles is controlled by the chemokine receptor CCR7 and the G-protein-coupled receptor EBI2 (GPR183) (**Figure 7C**) (84). C4_CD27^+^IGHM^-^ memory B cells express very low levels of IGHD and IGHM, although the expression of IGHG3 was found to be elevated (**Figures 7B, D**). IGHM undergoes a ‘switch-like’ recombination to form other immunoglobulin homotypes in GCs, suggesting that cells of the C4 cluster had completed the switch-like recombination. Several uniquely expressed genes were observed in these cells, which were enriched in proliferating cells (**Figure 7E**). C3_pDC preferentially expressed classic marker genes of plasmacytoid DCs (pDCs), including leukocyte immunoglobulin-like receptor subfamily A member 4 (LILRA4), interleukin-3 receptor subunit alpha (IL3RA), transcription factor 4 (TCF4) and C-type lectin domain family 4, member C (CLEC4C) (**Figure 7D**). C3_pDCs also expressed high levels of both GC migration factor GPR183 and anti-GC migration factor RGS13, which inhibited GC B lymphocytes’ response to the CXC-chemokine ligands CXCL12 and CXCL13 (85). A cluster of plasma B cells (C0_CD27^+^IGHM^+^ plasma cells) expressing high levels of the immunoglobulin heavy chains, IGHG1, IGHG2 (IgG), IGHA1 and IGHA2 (IgA) were also identified (**Figure 7D**). These cells were found to be mature plasma cells, according to the antibody-secretion capacity of PRDM1 (Blimp-1) (86). GO analysis revealed that both C2_CD27^-^IGHD^+^ B cells and C0_CD27^+^IGHM^+^ plasma cells were enriched in the processes of B cell activation and proliferation (**Figure 7F**).

### Complex Intercellular and Molecular Interaction Networks in the OS TME

**Figure 8:**
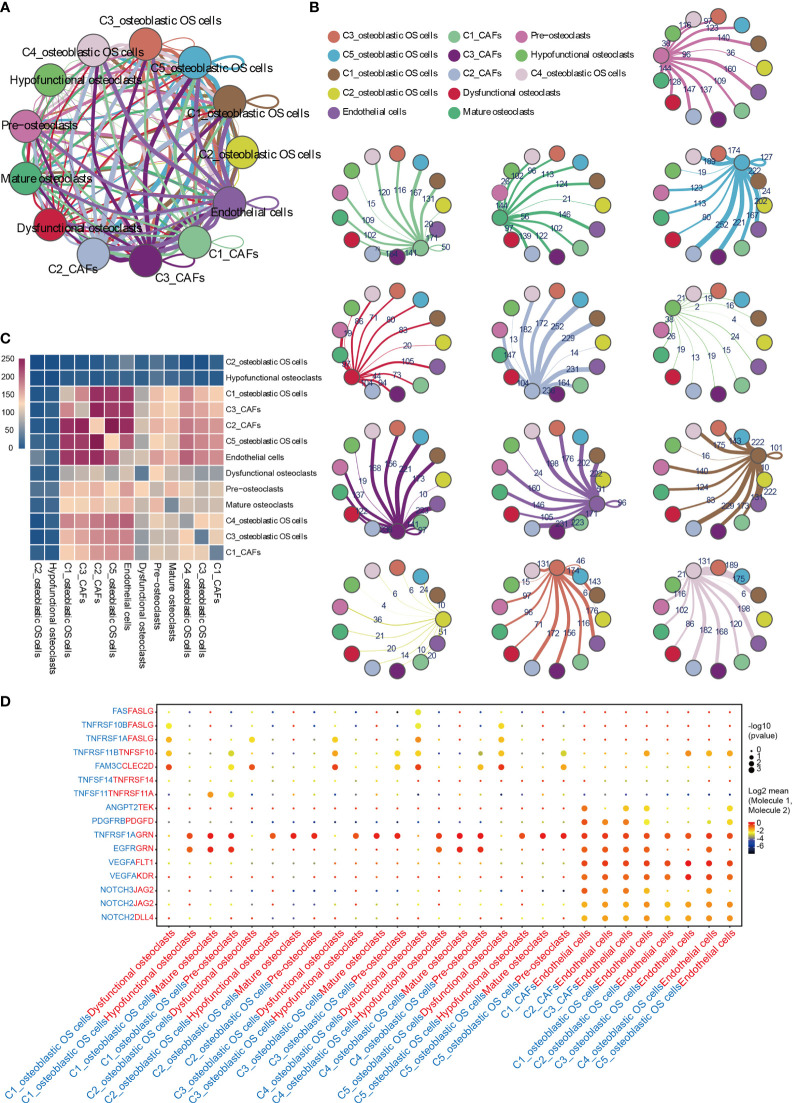
CellPhoneDB analysis of nonimmune cells in the OS. **(A)** Interaction network constructed by CellPhoneDB. Each line color indicates the ligands expressed by the cell population represented by the same color (labeled). The lines connect to the cell types that express the cognate receptors. The line thickness is proportional to the number of ligands when cognate receptors are present in the recipient cell type. **(B)** Detailed view of the ligands expressed by each major cell type, and the cells expressing the cognate receptors primed to receive the signal, are shown. Numbers indicate the quantity of ligand-receptor pairs for each intercellular link. **(C)** Heat map showing the number of potential ligand-receptor pairs among the predicted cell types. **(D)** Overview of selected ligand-receptor interactions of cells in OS. OS, osteosarcoma; TME, tumor microenvironment.

**Figure 9:**
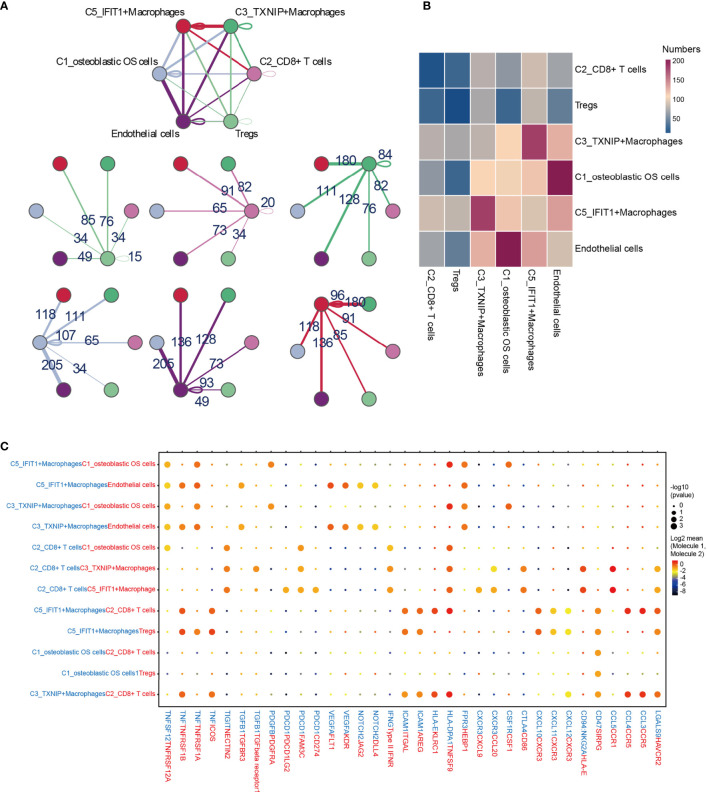
CellPhoneDB analysis of immune cells in the OS. **(A)** Interaction network constructed by TXNIP^+^ macrophages, IFIT1^+^ macrophages, osteoblastic OS cells, endothelial cells, Tregs and CD8^+^ T cells 2. **(B)** Heat map showing the potential ligand-receptor pairs among the predicted cell types. **(C)** Ligand-receptor pairs with a biological significance are shown in bubble diagrams. OS, osteosarcoma.

To describe the molecular links underlying the intercellular relationships, CellPhoneDB analysis was used to identify the molecular interactions between ligand-receptor pairs and major cell types in order to construct cellular communication networks. First, CellPhoneDB was used to analyze cellular communication among the osteoblastic OS cells, OCs and CAFs. The results of the CellPhoneDB analysis revealed the numbers of ligand receptors among all cell types (**Figures 8A–C**). Subsequently, the cells of interest were selected, and the ligand-receptor relationships among them were identified (**Figure 8D**). Osteoblastic OS cells and CAFs are able to produce VEGFA, which binds to VEGF receptors (FLT1 and KDR) on endothelial cells, thereby promoting angiogenesis. In addition, TNFSF1A, EGFR, NOTCH1, NOTCH2 and NOTCH3 are expressed in CAFs. These receptors are able to bind to granulin (GRN), Jagged-2 (JAG2) and Delta-like ligand 4 (DLL4), thereby promoting angiogenesis. Malignant tumors usually cause osteolysis, a process featuring classic ligand-receptor interactions between OC and osteoblastic OS cells involved in OC formation, such as TNFSF11-TNFRSF11A. According to the ELISA experimental data, tumor cells were able to secrete VEGFA and TNFSF11 (RANKL) (**Figures S2C, D**). In addition, tumor cells were found to be able to stimulate the tubular formation of HUVECs, and to stimulate the formation of OCs (**Figures S2E, F**). These data are consistent with the CellPhoneDB analysis. With respect to the communication between immune cells and tumor cells, CellPhoneDB analysis was used to explore the cellular communication among osteoblastic OS cells, macrophages and T cells. The results of the CellPhoneDB analysis revealed the numbers of ligand receptors among the three cell types (**Figures 9A, B**). Subsequently, the cells of interest were selected, and the ligand-receptor relationships among them were identified (**Figure 9C**). The majority of the ligand-receptor interactions occurring between IFIT1^+^ and TXNIP^+^ macrophages and endothelial cells involved angiogenic factors, such as VEGFA_KDR, VEGFA_FLT1, NOTCH2_DDL4 and NOTCH2_JAG2. Potential ligand-receptor interactions were also identified between C5_IFIT1^+^ macrophages and CD8^+^ T cells or Treg cells, including those involving chemokines (CXCL10_CCR3 and CXCL12_CCR3), adhesion junctions (ICAM1_ITGAL and ICAM1_AREG) and immune regulation (LGALS9_HAVCR2, PDCD1_PDCD1LG2 and PDCD1_CD274/PD-L1) (**Figure 9C**), which have been shown to promote the immune-suppressive activity of Tregs and CD8^+^ T cell exhaustion in TME (87, 88). Therefore, these results demonstrated the complexity of macrophage function in OS, greatly influencing T cell function with respect to balancing immune activation and inhibition. However, the interactions between tumor cells and T cells that are commonly observed were found to be mutually inhibited. Apart from TIGIT-NECTIN2/NECTIN3 and PDCD1-FAM3C, receptor-ligand pairings of IFNG-type II IFN production regulator (IFNR) were also identified between C2_CD8 T cells and osteoblastic OS cells (**Figure 9C**).

TIGIT/PVR exerts a key role in inhibiting the anti-tumor effects of CD8 T cells and NK cells (89, 90). Antibody double-blocking of TIGIT/PVR and programmed cell death protein 1 (PD-1)/programmed death-ligand 1 (PD-L1) has been demonstrated to produce synergistic effects in both tumor models and clinical trials (91). Tumor cells were also found to interact with Tregs through PVR-TIGIT ligand-receptor interactions, a process that enhances the immunosuppressive function and stability of Tregs (92).

## Discussion

The collected existing literature on OS mostly comprises studies on normal osteobiology, osteoimmunology, OS-associated models, genetic and genomic studies of OS, and finally, clinical studies (2). These studies have provided a useful basis for understanding the current treatment strategies for OS; however, they have not solved the problem of OS heterogeneity, and accurately analyzing the cellular components of OS has proven to be challenging. To address the heterogeneity of OS, six patients who had not received neoadjuvant chemotherapy for OS (which enables the natural state of OS to be revealed) were included in the present study. To the best of our knowledge, this is the first single-cell transcriptome study to have been performed on patients with OS prior to neoadjuvant chemotherapy. An unbiased approach was adopted using scRNA-seq to characterize the transcriptional changes and cellular heterogeneity in OS. The transcriptomic profiles of a total of 29,278 cells from six primary OS tissues were analyzed. First, the OS was divided into 9 subclusters. Malignant osteoblasts that served important roles were identified, and furthermore, OS cells were identified that had an important role in angiogenesis and osteolysis. In terms of immune cells, C3_TXNIP^+^ macrophages with similar M2 polarization, and C5_IFIT1^+^ macrophages with M1 polarization, were found. The presence of exhausted T cells in OS was also discovered, with the identification of key immune checkpoints. Taken together, the findings of the present study have provided an in-depth insight into the multicellular ecosystem of OS.

The differentiation origin of OS is considered to be either mesenchymal stem cells or OBs (93). In the present study, OS cells were identified in OBs and validated by CNV analysis. The classification of osteoblastic OS cells is closely correlated with the prognosis, indicating its important clinical significance. Currently, due to the high complexity of the TME of OS, it is not possible to fully characterize the heterogeneity of malignant cells. However, regarding the single-cell transcriptome data obtained from the six patients, five osteoblastic OS cell subtypes were identified, having different gene expression patterns. Each of these subtypes of malignant cells served a different role, and several important results were also highlighted. The ability of C1_osteoblastic OS cells to differentiate into other malignant subsets. Notably, the relative abundance of C1/5_osteoblastic OS cells was associated with poor prognosis in the TARGET OS cohort. Therefore, C1/5_osteoblastic OS cells may have greater clinical significance in terms of potential therapies and therapeutic applications. The TF expression pattern of C1/5_osteoblastic OS cells was also found to be different from that of other malignant osteoblast subtypes (DDIT3, SP7 and CREB3L1). On further analysis of the TFs, SP7 was found to be strongly associated with prognosis. It has been revealed that SP7 can promote lymph node metastasis of breast cancer, promote angiogenesis, reduce the sensitivity of chemotherapy and have a worse prognosis (69, 94, 95). However, the related phenotypes in human osteosarcoma need to be further investigated.

**Figure 10:**
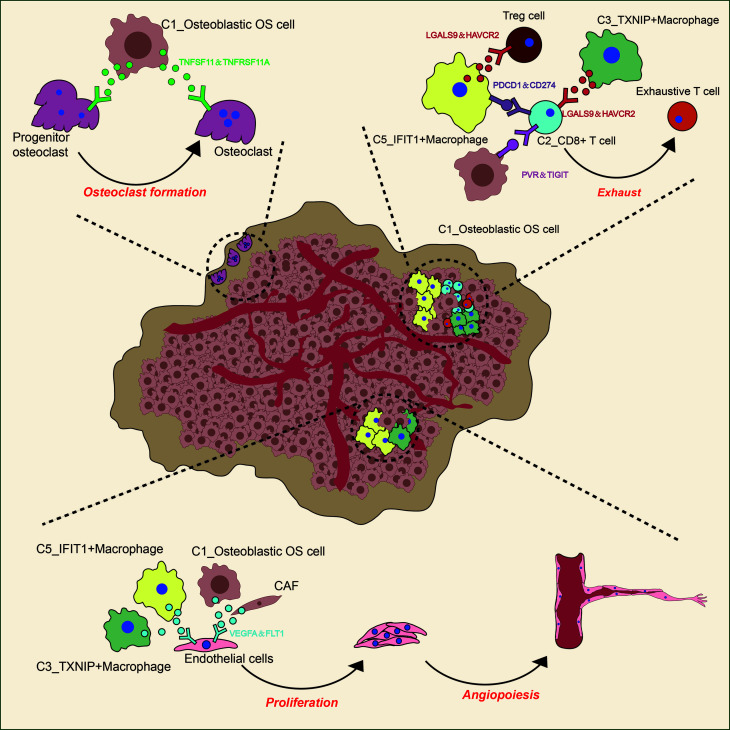
Predicted regulatory network, centered on OS.

Angiogenesis has an important role in the development of OS (**Figure 10**) (96, 97). It is now widely considered that mutations in both oncogenes and tumor suppressor genes cause angiogenesis in tumors (98). The VEGF family is a key gene family that fulfills functions involved in stimulating angiogenesis, the inflammatory response and vascular permeability, and it also has an important role in the regulation of tumor angiogenesis (99, 100). In the present study, through CellPhoneDB analysis, osteoblastic OS cells were found to regulate endothelial cells *via* VEGFA-VEGFRA interactions. Osteolysis is an important biological feature that enables OS cells to break their boundaries, mobilizing them. This unique biological feature is beneficial for the invasive ability of tumor cells (**Figure 10**) (7). OCs are the only cells that are known to undergo bone resorption in the human body. The cellular interactions occurring between OS cells and OCs are worthy of further investigation. The data from the present study have suggested that osteoblastic OS cells regulate OC differentiation and function through the TNFSF11-TNFRSF11A interaction. In conclusion the communication status of three different cell types involved in the occurrence, development and invasion of OS has been presented in this study. In the future, newly developed detection methods will be able to further clarify the underlying regulatory mechanisms.

Immune cells make up an important part of the complexity of the TME in OS (**Figure 10**) (100). It was observed in the present study that C5_IFIT1^+^ macrophages express high levels of IFN-induced proteins (ISG15, IFIT1 and IFITM3, among others) and the CXC chemokine ligand CXCL10, showing a phenotypic bias of M1 macrophages co-induced by type I and type II IFN, similar to that observed for ISG15^+^ TAMs in the majority of tumors (101). This finding suggested that these macrophages potentially have an anti-tumor capability in OS (102, 103). However, C5_IFIT1^+^ macrophages interact with depleted T cells and Tregs through ligand receptors, such as chemokines (CXCL9/CXCL10 _CXCR3 and CXCL12_CXCR4) and T-cell immune checkpoints, including LGALS9 (binds to HAVCR2), PDCD1LG2 (binds to PD-L2), CD274 (binds to PD-L1), and SPP1 (binds to CD44), indicating that they are also associated with T cell inhibitory signals (104, 105), which may explain why they also exhibit inflammatory suppression. These results have provided useful information, although further studies are required to attain a more detailed understanding of the function of these types of macrophages. Immunotherapy has slowly grown to prominence as a promising approach for cancer treatment. Several studies have suggested that OS can be treated by blocking PD-1 (106–108). PD-1 is the main inhibitory receptor expressed on T cells, which interacts with PD-L1 to cause the exhaustion of T cells and suppression of the immune response (109). Furthermore, the investigation of the cell-cell interactions suggested that C2_CD8^+^ T cells are able to communicate with osteoblastic OS cells; in particular, associations were identified between IFNG_ type II IFNR and PDCD1-FAM3C, which are representative of C2_CD8^+^ T cells mediating the immune response to tumor cells *via* IFN-γ. IFN-γ plus PD-1 blockers have been reported to enhance immune function in pancreatic cancer, and this has been strategically used in the treatment of secondary metastases (110). This may also be applicable in the case of tumor immunotherapy for OS, although further research in this regard is required.

In conclusion, the present study has identified and analyzed the heterogeneity of the TME in OS, providing a valuable single-cell transcriptome atlas, with the identification of novel cell types with important functions in the TME of OS tumors. The study has also provided targets associated with OS survival, which will lay the foundation for future research studies on precision therapy of OS.

## Data Availability Statement

The datasets presented in this study can be found in online repositories. The names of the repository/repositories and accession number(s) can be found below: https://www.ncbi.nlm.nih.gov/, GSE162454.

## Ethics Statement

The studies involving human participants were reviewed and approved by the Ethics Committee of The First Affiliated Hospital of Guangxi Medical University (approval no. 2019KYK-E-097). Written informed consent to participate in this study was provided by the participants’ legal guardian/next of kin.

## Author Contributions

QW, ZM, and JM were responsible for the conception of the study. WF, QH, ZQ, JH, SL, and MH were responsible for the collection of osteosarcoma tissue. YD, MB, JL, BG, RH, YY, WF, QH, ZQ, CL, and JH dissected the osteosarcoma tissues and performed the RNA-seq experiments. WF and ZYY analyzed the data. MH performed the immunofluorescence staining. YL, WF YD, MB, and MH wrote the paper. QW, ZM, JM, JX, ZCY, YJ, ZX, and RY modified the paper. All authors contributed to the article and approved the submitted version.

## Conflict of Interest

The authors declare that the research was conducted in the absence of any commercial or financial relationships that could be construed as a potential conflict of interest.

## Supplementary Material

Supplementary Figure 1
**(A)** Large-scale chromosomal landscape in 6 patients were calculated using reference cell myeloid cells 1/2, NK/T cells, plasmocytes and B cells; red represents an increased copy number, whereas blue indicates a decreased copy number. **(B)** The networks consist of SP7 and their target genes; red nodes represent TFs, whereas the blue nodes represent target genes. **(C)** Kaplan-Meier curve of the independent genes associated with survival (SP7, SOX9, ADARB1, CREB3L1, DDIT3, RUNX2, FOXC1, GTF2B, JUN and KLF2). **(D)** Bar plot showing the proportions of the 5 types of osteoblastic OS cells in OS samples. The column names of the plots indicate the sample IDs. **(E)** CDF plots, revealing the consensus distributions for each k. CDF, cumulative distribution function; TF, transcription factor.

Supplementary Figure 2
**(A)** Multiplex IHC staining of OS tissue. TNFSF11A was co-stained with ALPL (scale bar=100 μm). **(B)** Multiplex IHC staining of OS tissue. IFIT1 was co-stained with CD68 (scale bar=200 μm). **(C, D)** The results of ELISA analysis of VEGFA and TNFSF11A in DMEM of OS cell lines. **(E)** Tube-formation assay of HUVECs treated by conditioned medium of OS cell lines or non-conditioned medium of OS cell lines; representative images are shown, highlighting the promotion effect (scale bar=1,000 μm). **(F)** TRAP staining of BMMs treated by conditioned medium of OS cell lines or non-conditioned medium of OS cell lines; representative images have been selected to show the promotion effect (scale bar=200 μm). ***P < 0.001, **P < 0.01, *P < 0.05. IHC, immunohistochemistry; ALPL, alkaline phosphatase, tissue-nonspecific isozyme; OS, osteosarcoma; TRAP, tartrate-resistant acid phosphatase; DMEM, Dulbecco’s modified Eagle’s medium; HUVECs, human umbilical vascular endothelial cells; BMMs, bone marrow macrophages; VEGFA, vascular endothelial growth factor A; TNFSF11A, TNF superfamily member 11A.

Supplementary Figure 3
**(A)** Volcano plot showing all genes that were differentially expressed between Cluster2 and Cluster1. **(B)** Volcano plot showing all genes that were differentially expressed between Cluster3 and Cluster1. **(C)** Heatmap of the 518 genes that were differentially expressed. **(D)** Heatmap of the 85 malignant genes. **(E)** Box plot of the malignant gene score in osteoblastic OS cells. **(F)** Relative abundance of osteoblastic OS cells (C1, C2, C3 and C4) in Clusters 1 (left), 2 (middle) and 3 (right). OS, osteosarcoma.

Supplementary Document 1 Kaplan-Meier curve of the 518 genes associated with survival.

Supplementary Document 2 Kaplan-Meier curve of the 85 malignant genes associated with survival.
