# Corrigendum: Single-cell transcriptomics reveals the complexity of the tumor microenvironment of treatment-naive osteosarcoma

**DOI:** 10.3389/fonc.2022.1077067

**Published:** 2022-11-15

**Authors:** Yun Liu, Wenyu Feng, Yan Dai, Mengying Bao, Zhenchao Yuan, Mingwei He, Zhaojie Qin, Shijie Liao, Juliang He, Qian Huang, Zhenyuan Yu, Yanyu Zeng, Binqian Guo, Rong Huang, Rirong Yang, Yonghua Jiang, Jinling Liao, Zengming Xiao, Xinli Zhan, Chengsen Lin, Jiake Xu, Yu Ye, Jie Ma, Qingjun Wei, Zengnan Mo

**Affiliations:** ^1^ Department of Spinal Bone Disease, First Affiliated Hospital of Guangxi Medical University, Nanning, China; ^2^ Department of Trauma Orthopedic and Hand Surgery, First Affiliated Hospital of Guangxi Medical University, Nanning, China; ^3^ Center for Genomic and Personalized Medicine, School of Preclinical Medicine, Guangxi Medical University, Nanning, China; ^4^ Guangxi Key Laboratory for Genomic and Personalized Medicine, Guangxi Key Laboratory of Colleges and Universities, Nanning, China; ^5^ Guangxi Collaborative Innovation Center for Genomic and Personalized Medicine, Guangxi Medical University, Nanning, China; ^6^ Department of Bone and Soft Tissue Surgery, The Affiliated Tumor Hospital, Guangxi Medical University, Nanning, China; ^7^ School of Biomedical Sciences, The University of Western Australia, Perth, WA, Australia; ^8^ Department of Medical Oncology, First Affiliated Hospital of Guangxi Medical University, Nanning, China; ^9^ Guangxi Key Laboratory of Regenerative Medicine, Research Centre for Regenerative Medicine, Guangxi Medical University, Nanning, China

**Keywords:** single-cell RNA sequencing, tumor microenvironment, naive osteosarcoma, heterogeneity, osteolysis

In the published article, there was an error in **Figure 1D** as published. The labels of feature plots in **Figure 1D** were misleadingly described. The original labels for “osteoblastic OS cells” should switch with “CAFs”, as well as “Osteoclasts” should switch with “Plasmocytes”. The corrected **Figure 1D** and its caption appear below.

**Figure 1:**
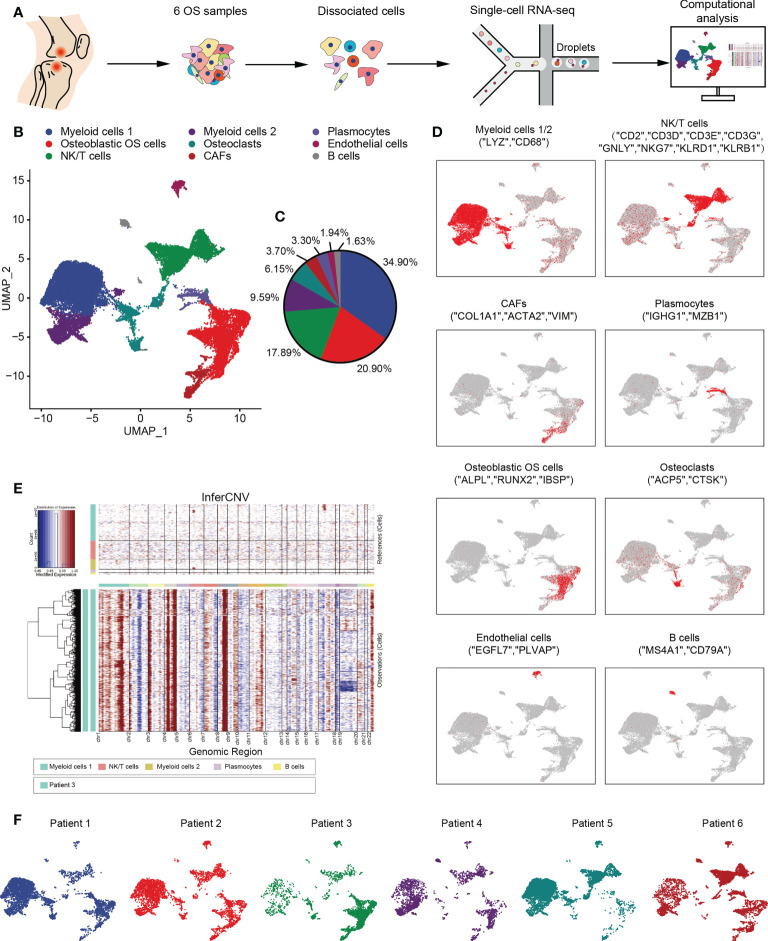
Overview of single cells derived from OS tissues. **(A)** Workflow depicting collection and processing of specimens of OS tumors for scRNA-seq. **(B)** UMAP plot of all the single cells, with each color-coded for the 9 major cell types. **(C)** Pie chart, indicating the cell composition of OS. **(D)** UMAP plots of the normalized marker expression of the 9 major cell types. **(E)** The large-scale chromosomal landscape in patient 3 was calculated using reference cells (myeloid cells 1/2, NK/T cells, plasmocytes and B cells); the red color represents an increased copy number, whereas the blue color represents a decreased copy number. **(F)** UMAP plot of all the single cells, with each cell color-coded for different patients. OS, osteosarcoma; UMAP, uniform manifold approximation and projection; scRNA-seq, single-cell RNA sequencing; NK, natural killer.

In the published article, there was an error in **Figure 1E** as published. The label of “Patient 1” in **Figure 1E** was incorrectly described, which should be changed to “Patient 3”. The corrected **Figure 1E** and its caption appear below.

In the published article, there was an error in **Supplementary Figure 1A** as published. The labels of “Patient 1” in **Supplementary Figure 1A** were incorrectly described. The six “Patient 1” in inferCNV of **Supplementary Figure 1A** should need to be corrected in order.

The authors apologize for this error and state that this does not change the scientific conclusions of the article in any way. The original article has been updated.

## Publisher’s note

All claims expressed in this article are solely those of the authors and do not necessarily represent those of their affiliated organizations, or those of the publisher, the editors and the reviewers. Any product that may be evaluated in this article, or claim that may be made by its manufacturer, is not guaranteed or endorsed by the publisher.

