# Supplementary figures and images for: Single-Cell Transcriptomics Reveals the Complexity of the Tumor Microenvironment of Treatment-Naive Osteosarcoma (part 1 of 3)
Source: Front Oncol. 2021 Jul 21;11:709210. doi: 10.3389/fonc.2021.709210 (PMC8335545; doi:10.3389/fonc.2021.709210)

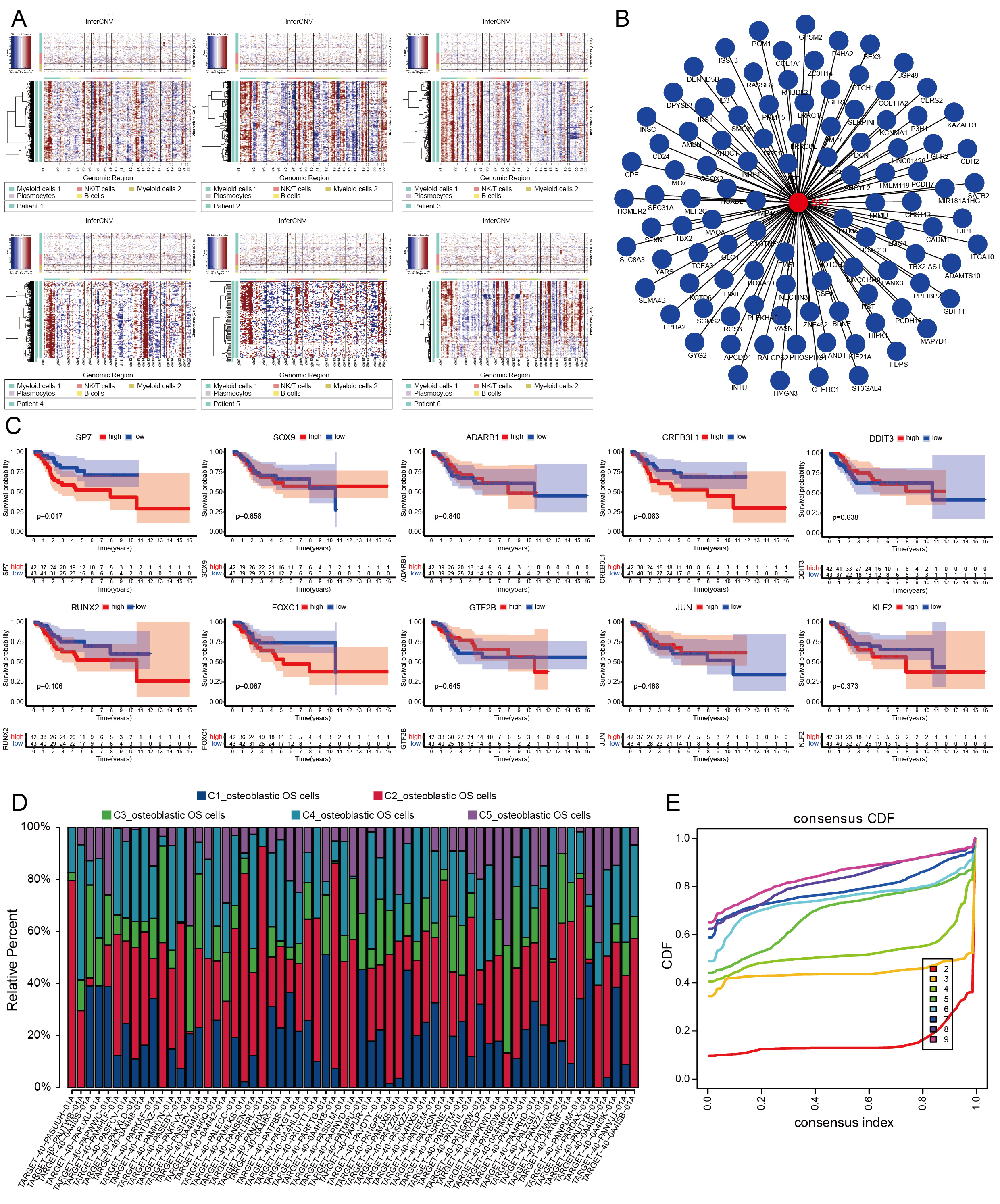

Supplement: Supplementary Figure 1 — (A) Large-scale chromosomal landscape in 6 patients were calculated using reference cell myeloid cells 1/2, NK/T cells, plasmocytes and B cells; red represents an increased copy number, whereas blue indicates a decreased copy number. (B) The networks consist of SP7 and their target genes; red nodes represent TFs, whereas the blue nodes represent target genes. (C) Kaplan-Meier curve of the independent genes associated with survival (SP7, SOX9, ADARB1, CREB3L1, DDIT3, RUNX2, FOXC1, GTF2B, JUN and KLF2). (D) Bar plot showing the proportions of the 5 types of osteoblastic OS cells in OS samples. The column names of the plots indicate the sample IDs. (E) CDF plots, revealing the consensus distributions for each k. CDF, cumulative distribution function; TF, transcription factor. [file Image_1.jpg]

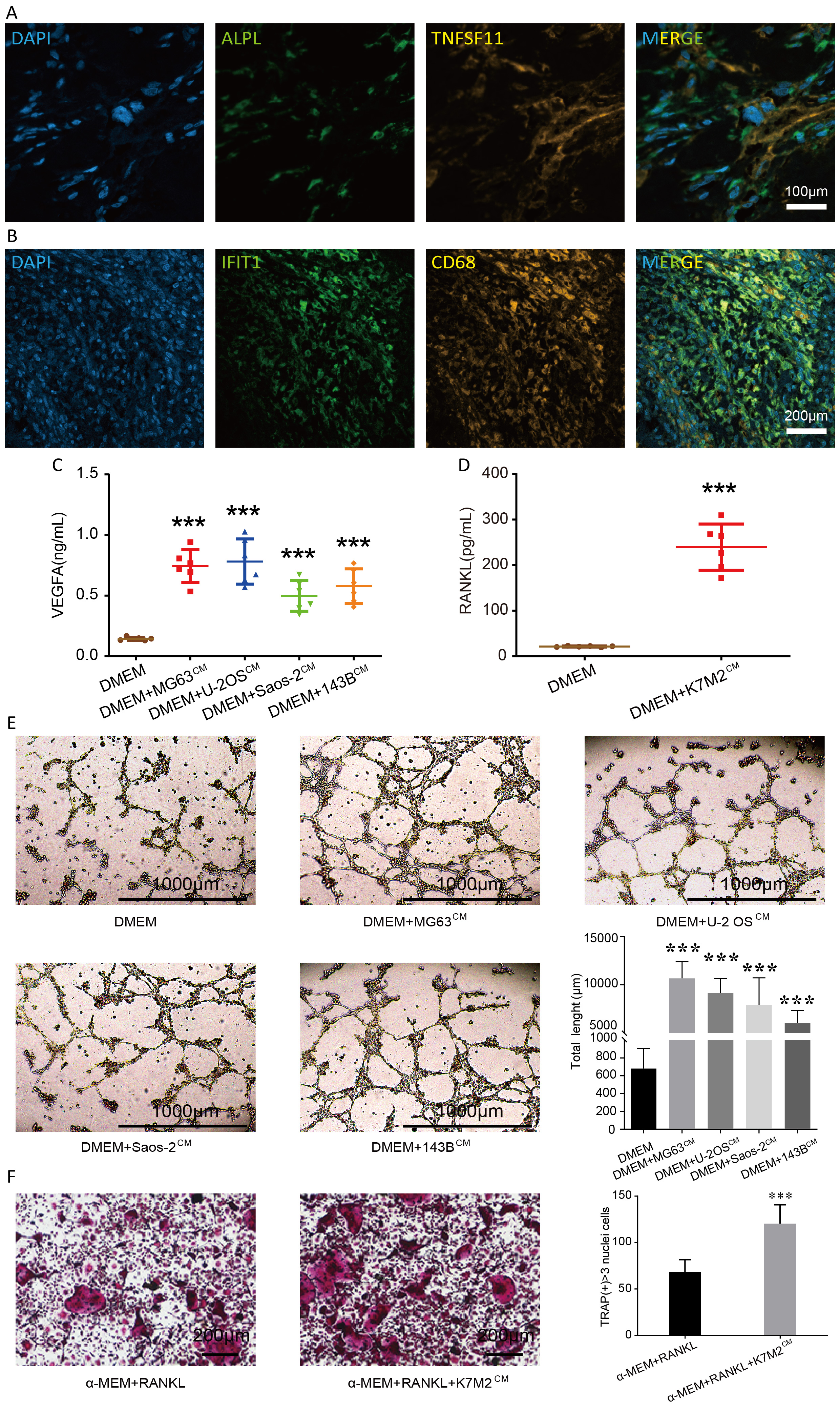

Supplement: Supplementary Figure 2 — (A) Multiplex IHC staining of OS tissue. TNFSF11A was co-stained with ALPL (scale bar=100 μm). (B) Multiplex IHC staining of OS tissue. IFIT1 was co-stained with CD68 (scale bar=200 μm). (C, D) The results of ELISA analysis of VEGFA and TNFSF11A in DMEM of OS cell lines. (E) Tube-formation assay of HUVECs treated by conditioned medium of OS cell lines or non-conditioned medium of OS cell lines; representative images are shown, highlighting the promotion effect (scale bar=1,000 μm). (F) TRAP staining of BMMs treated by conditioned medium of OS cell lines or non-conditioned medium of OS cell lines; representative images have been selected to show the promotion effect (scale bar=200 μm). ***P < 0.001, **P < 0.01, *P < 0.05. IHC, immunohistochemistry; ALPL, alkaline phosphatase, tissue-nonspecific isozyme; OS, osteosarcoma; TRAP, tartrate-resistant acid phosphatase; DMEM, Dulbecco’s modified Eagle’s medium; HUVECs, human umbilical vascular endothelial cells; BMMs, bone marrow macrophages; VEGFA, vascular endothelial growth factor A; TNFSF11A, TNF superfamily member 11A. [file Image_2.jpg]

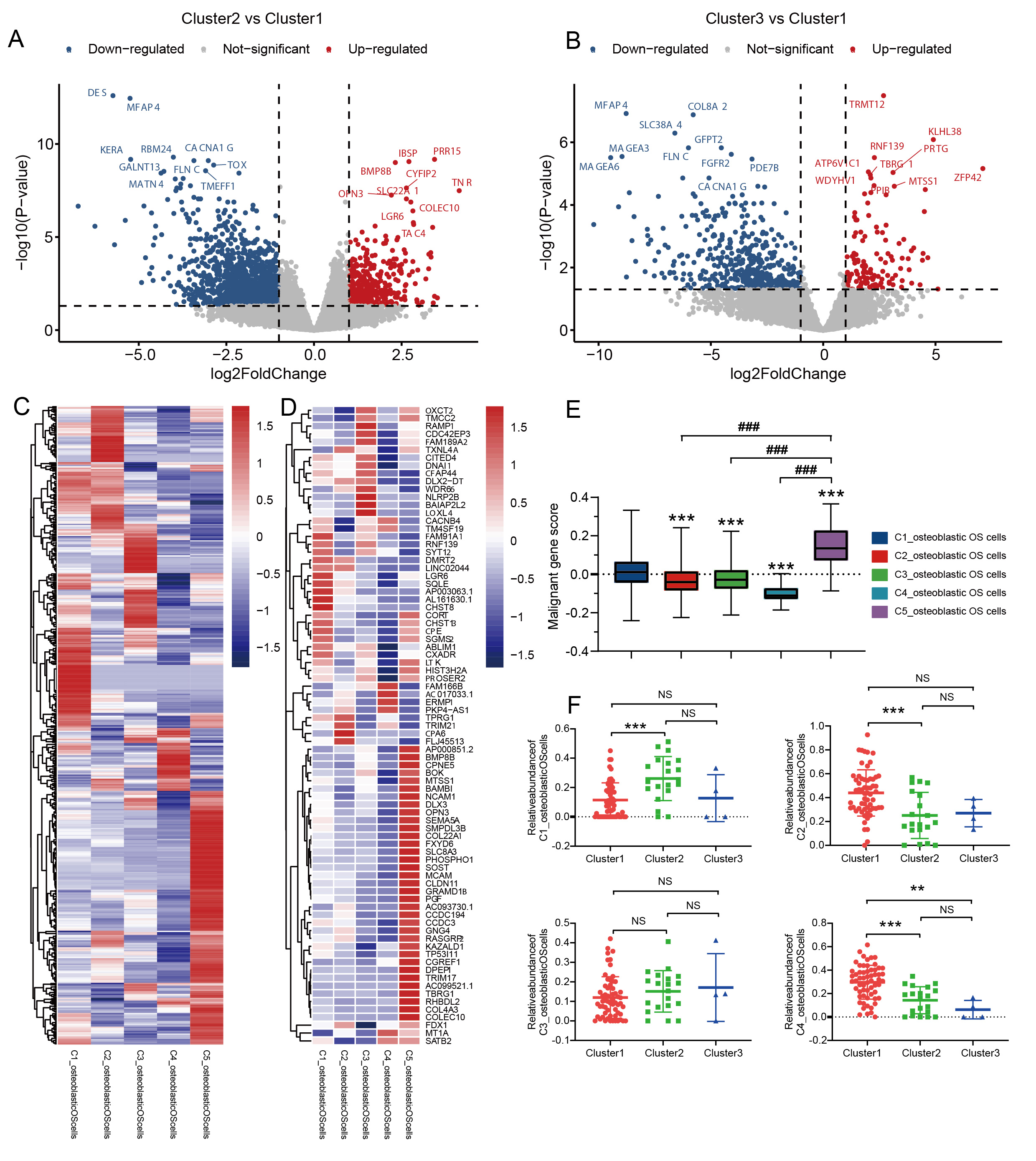

Supplement: Supplementary Figure 3 — (A) Volcano plot showing all genes that were differentially expressed between Cluster2 and Cluster1. (B) Volcano plot showing all genes that were differentially expressed between Cluster3 and Cluster1. (C) Heatmap of the 518 genes that were differentially expressed. (D) Heatmap of the 85 malignant genes. (E) Box plot of the malignant gene score in osteoblastic OS cells. (F) Relative abundance of osteoblastic OS cells (C1, C2, C3 and C4) in Clusters 1 (left), 2 (middle) and 3 (right). OS, osteosarcoma. [file Image_3.jpeg]

ABCA3 high low

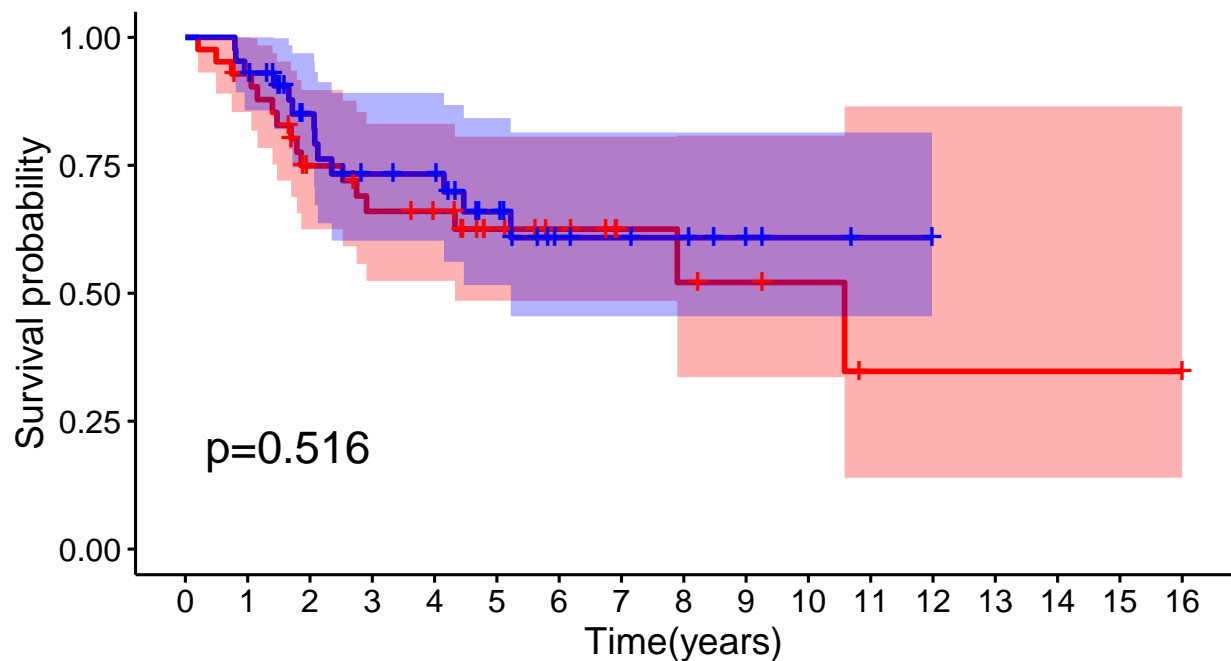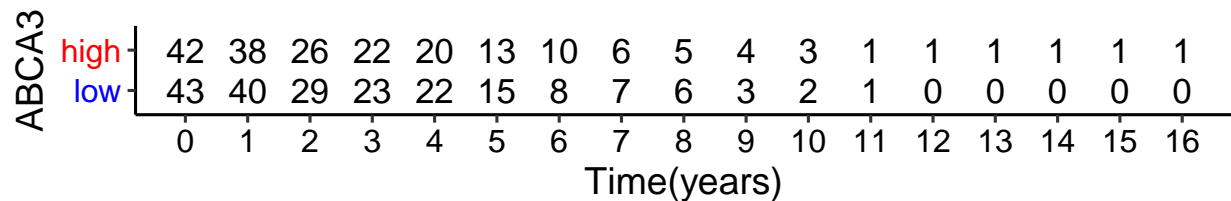

Supplement: Supplementary Document 1 — Kaplan-Meier curve of the 518 genes associated with survival. [file DataSheet_1.zip › Supplementary Document 1/sur.ABCA3.pdf]

ABCA4 + high + low

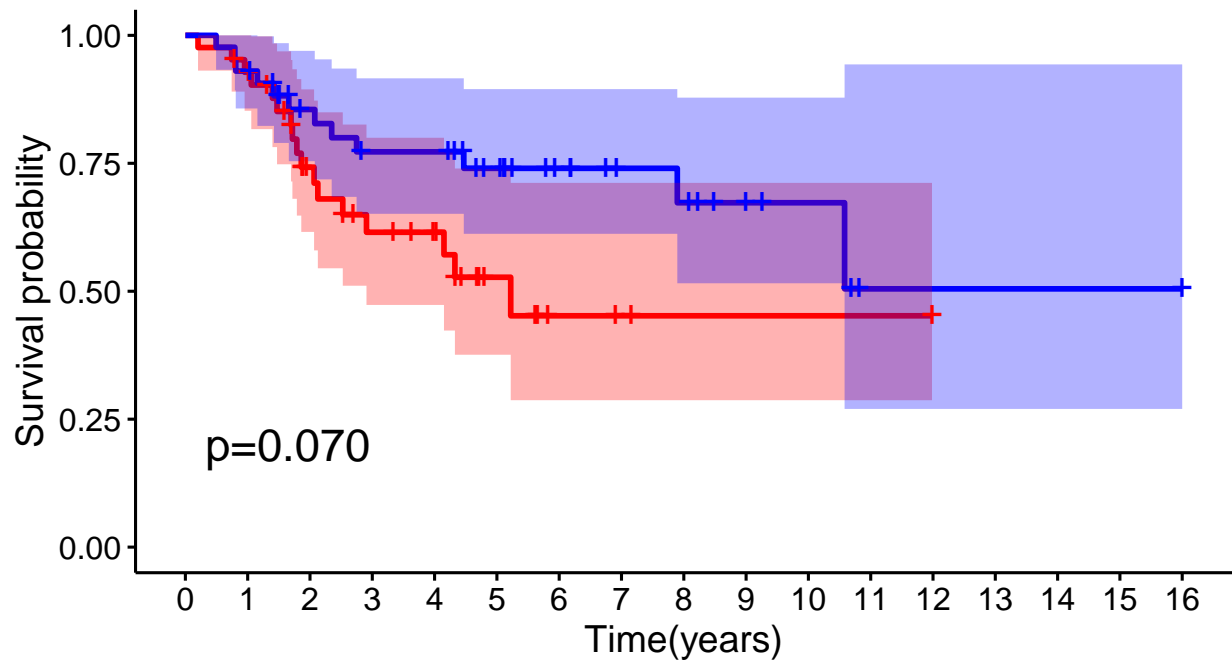

ABCA4

|      |             |    |    |    |    |    |    |    |    |   |    |    |    |    |    |    |    |
|------|-------------|----|----|----|----|----|----|----|----|---|----|----|----|----|----|----|----|
| high | 42          | 38 | 24 | 18 | 15 | 7  | 3  | 2  | 1  | 1 | 1  | 1  | 0  | 0  | 0  | 0  | 0  |
| low  | 43          | 40 | 31 | 27 | 27 | 21 | 15 | 11 | 10 | 6 | 4  | 1  | 1  | 1  | 1  | 1  | 1  |
|      | 0           | 1  | 2  | 3  | 4  | 5  | 6  | 7  | 8  | 9 | 10 | 11 | 12 | 13 | 14 | 15 | 16 |
|      | Time(years) |    |    |    |    |    |    |    |    |   |    |    |    |    |    |    |    |

Supplement: Supplementary Document 1 — Kaplan-Meier curve of the 518 genes associated with survival. [file DataSheet_1.zip › Supplementary Document 1/sur.ABCA4.pdf]

ABCB4 high low

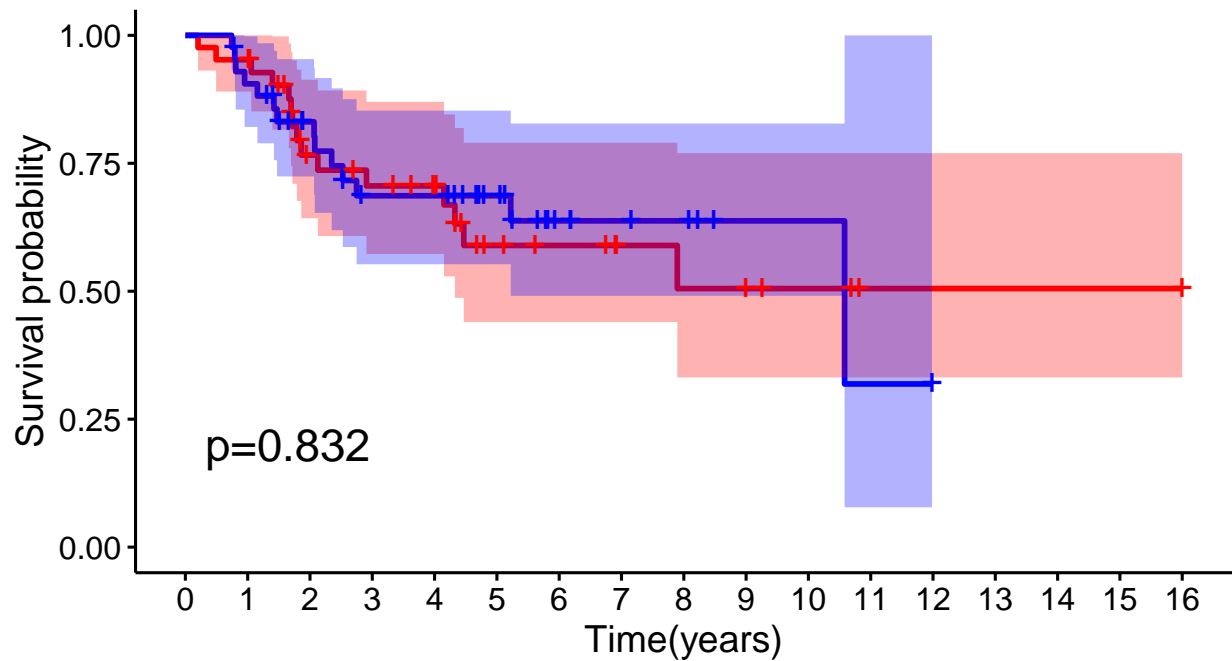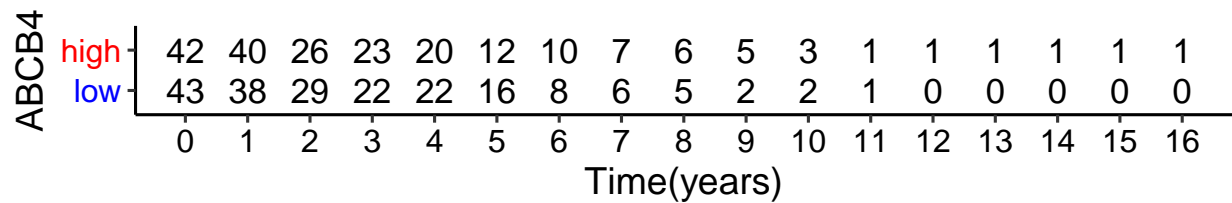

Supplement: Supplementary Document 1 — Kaplan-Meier curve of the 518 genes associated with survival. [file DataSheet_1.zip › Supplementary Document 1/sur.ABCB4.pdf]

ABCC8 + high + low

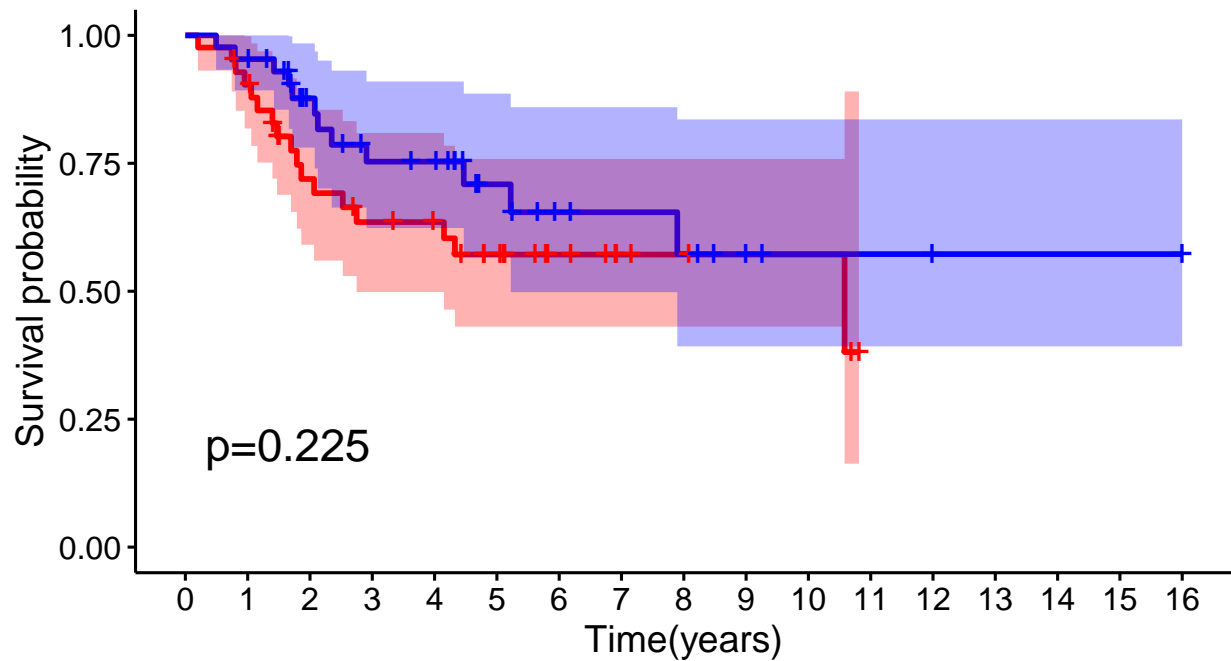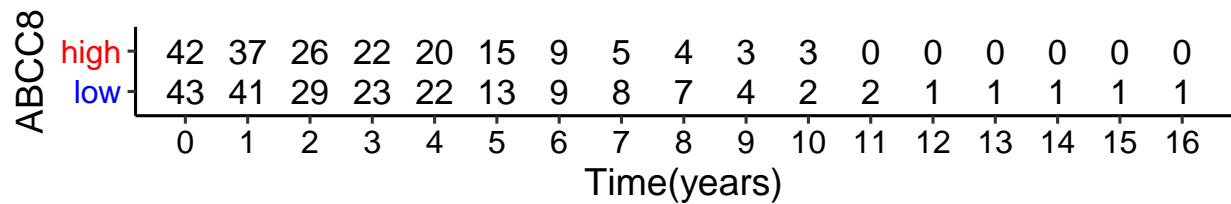

Supplement: Supplementary Document 1 — Kaplan-Meier curve of the 518 genes associated with survival. [file DataSheet_1.zip › Supplementary Document 1/sur.ABCC8.pdf]

ABLIM1 high low

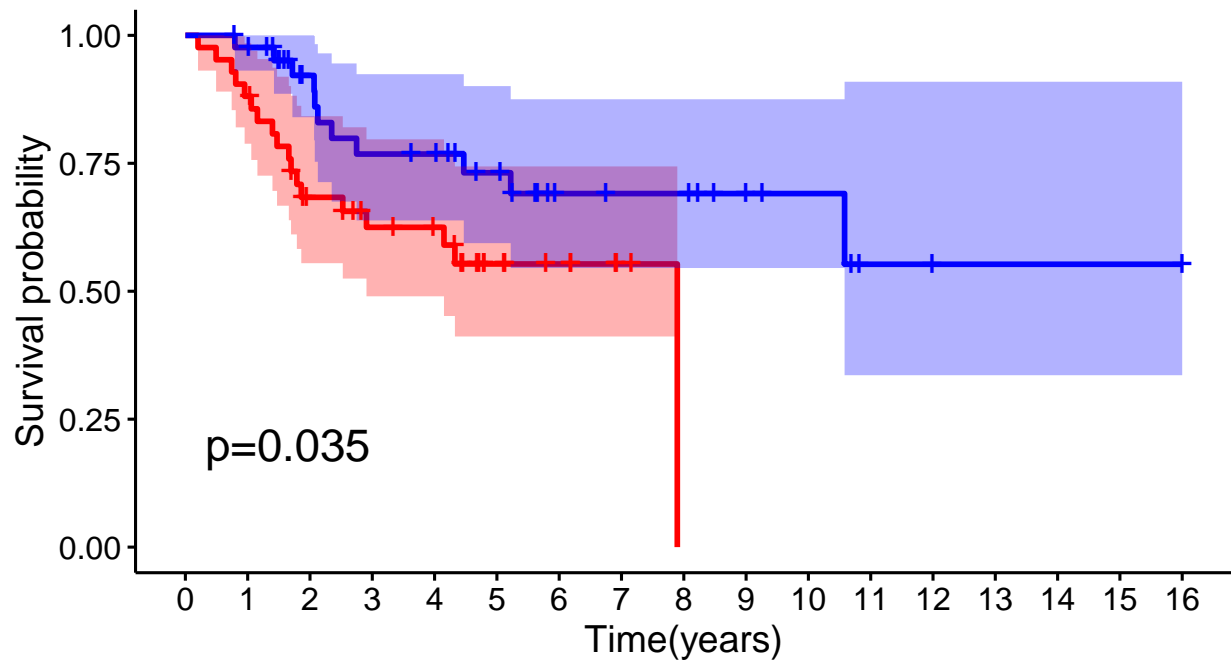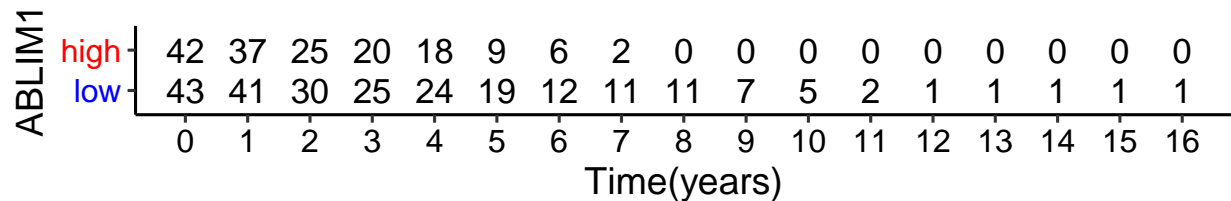

Supplement: Supplementary Document 1 — Kaplan-Meier curve of the 518 genes associated with survival. [file DataSheet_1.zip › Supplementary Document 1/sur.ABLIM1.pdf]

AC003985.2

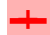

high

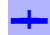

low

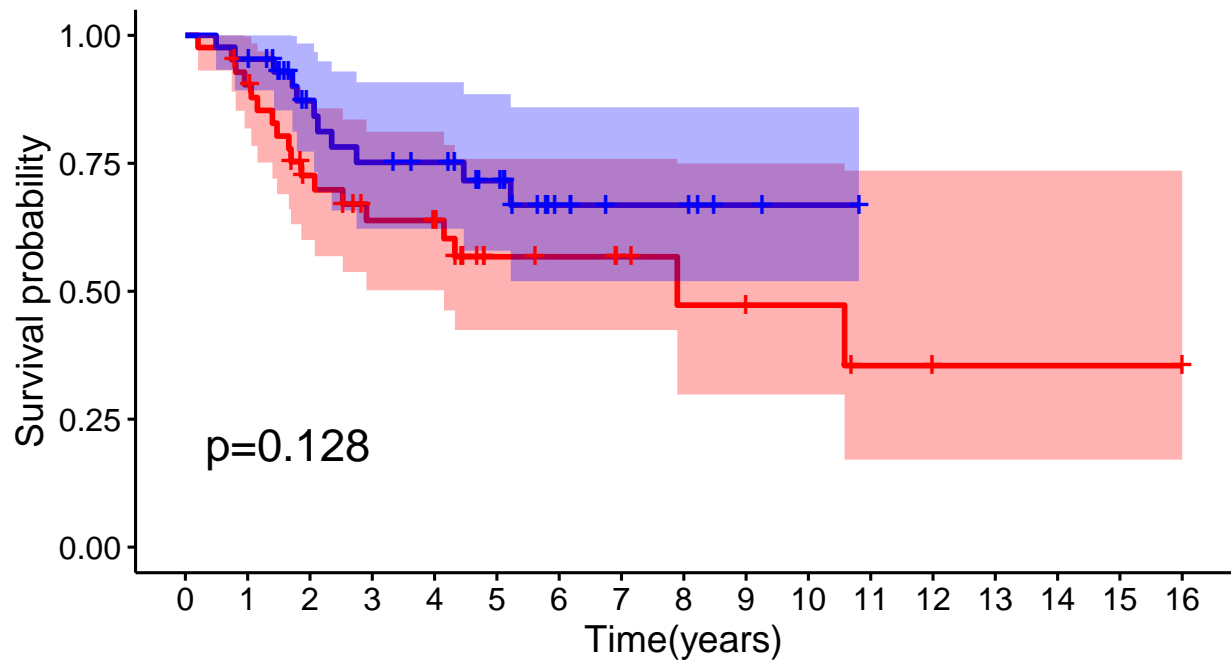

AC003985.2

high

low

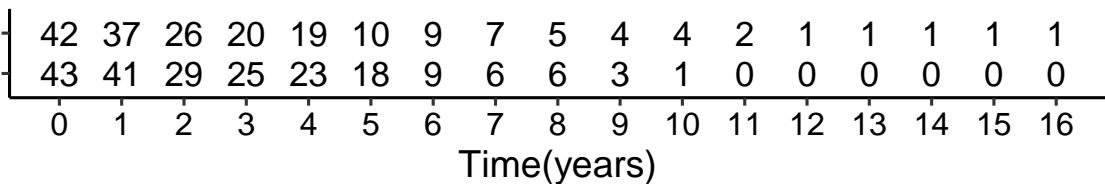

Supplement: Supplementary Document 1 — Kaplan-Meier curve of the 518 genes associated with survival. [file DataSheet_1.zip › Supplementary Document 1/sur.AC003985.2.pdf]

AC003991.1

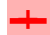

high

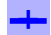

low

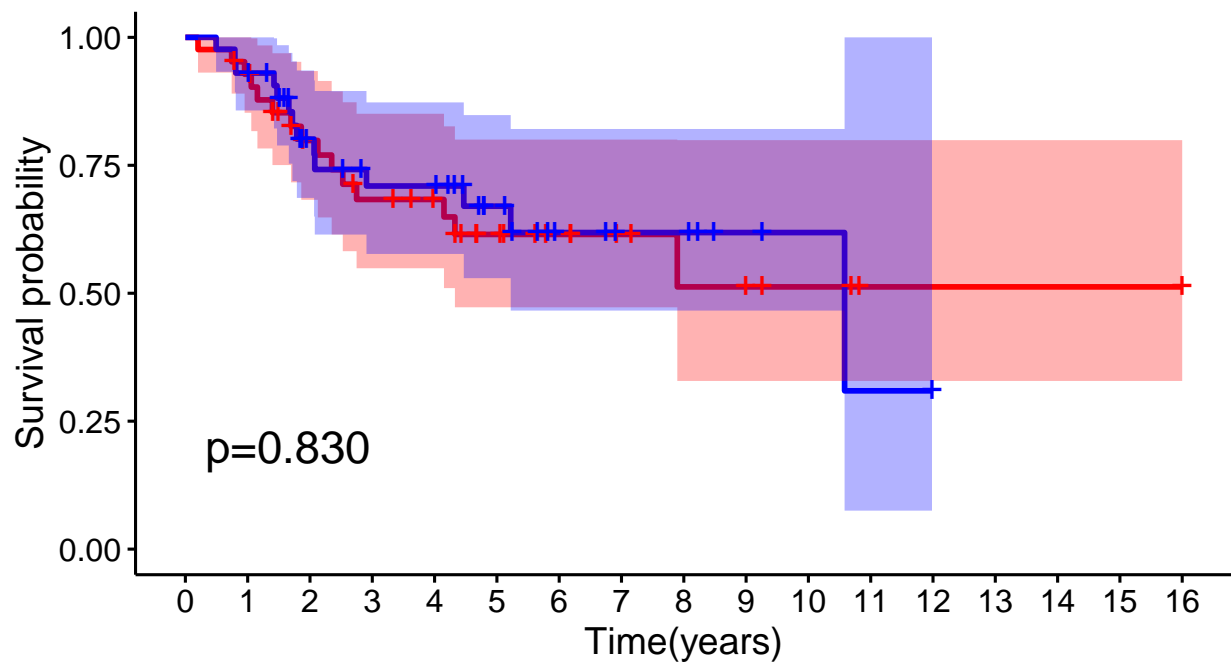

AC003991.1

high

low

|    |    |    |    |    |    |    |   |   |   |    |    |    |    |    |    |    |
|----|----|----|----|----|----|----|---|---|---|----|----|----|----|----|----|----|
| 0  | 1  | 2  | 3  | 4  | 5  | 6  | 7 | 8 | 9 | 10 | 11 | 12 | 13 | 14 | 15 | 16 |
| 42 | 38 | 28 | 23 | 20 | 14 | 10 | 7 | 5 | 4 | 3  | 1  | 1  | 1  | 1  | 1  | 1  |
| 43 | 40 | 27 | 22 | 22 | 14 | 8  | 6 | 6 | 3 | 2  | 1  | 0  | 0  | 0  | 0  | 0  |

Time(years)

Supplement: Supplementary Document 1 — Kaplan-Meier curve of the 518 genes associated with survival. [file DataSheet_1.zip › Supplementary Document 1/sur.AC003991.1.pdf]

AC004148.2

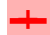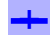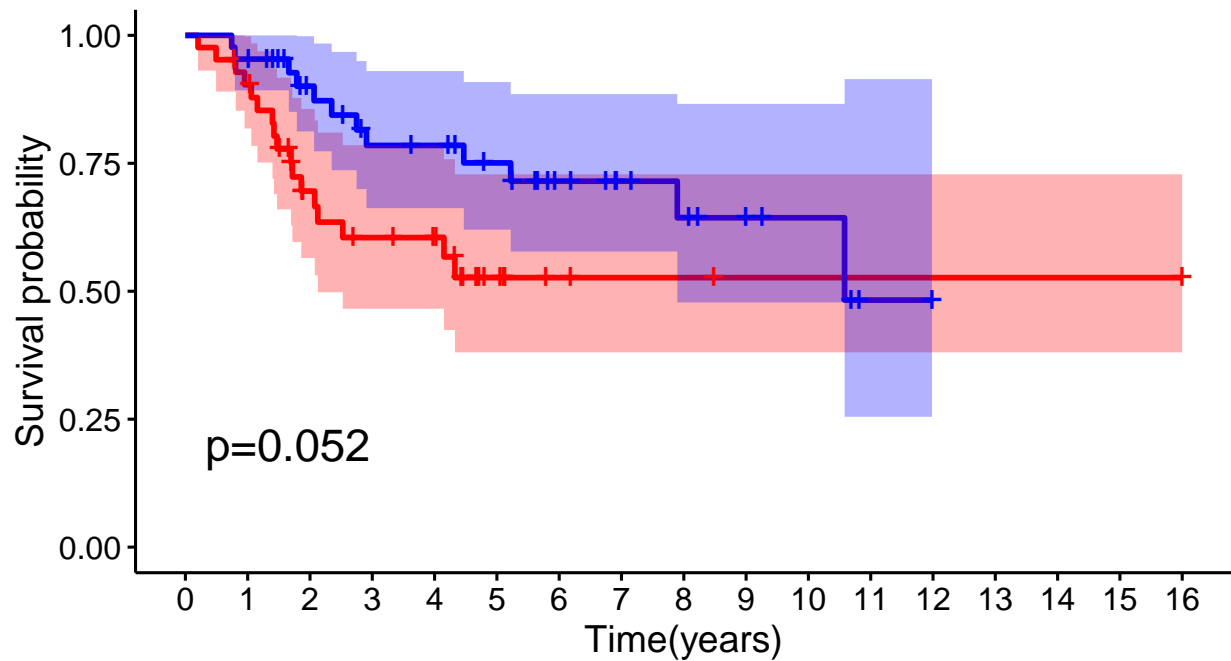

AC004148.2

high

low

|    |    |    |    |    |    |    |    |   |   |    |    |    |    |    |    |
|----|----|----|----|----|----|----|----|---|---|----|----|----|----|----|----|
| 42 | 37 | 23 | 19 | 17 | 7  | 3  | 2  | 2 | 1 | 1  | 1  | 1  | 1  | 1  | 1  |
| 43 | 41 | 32 | 26 | 25 | 21 | 15 | 11 | 9 | 6 | 4  | 1  | 0  | 0  | 0  | 0  |
| 0  | 1  | 2  | 3  | 4  | 5  | 6  | 7  | 8 | 9 | 10 | 11 | 12 | 13 | 14 | 15 |

Time(years)

Supplement: Supplementary Document 1 — Kaplan-Meier curve of the 518 genes associated with survival. [file DataSheet_1.zip › Supplementary Document 1/sur.AC004148.2.pdf]

AC005037.1

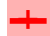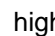

high low

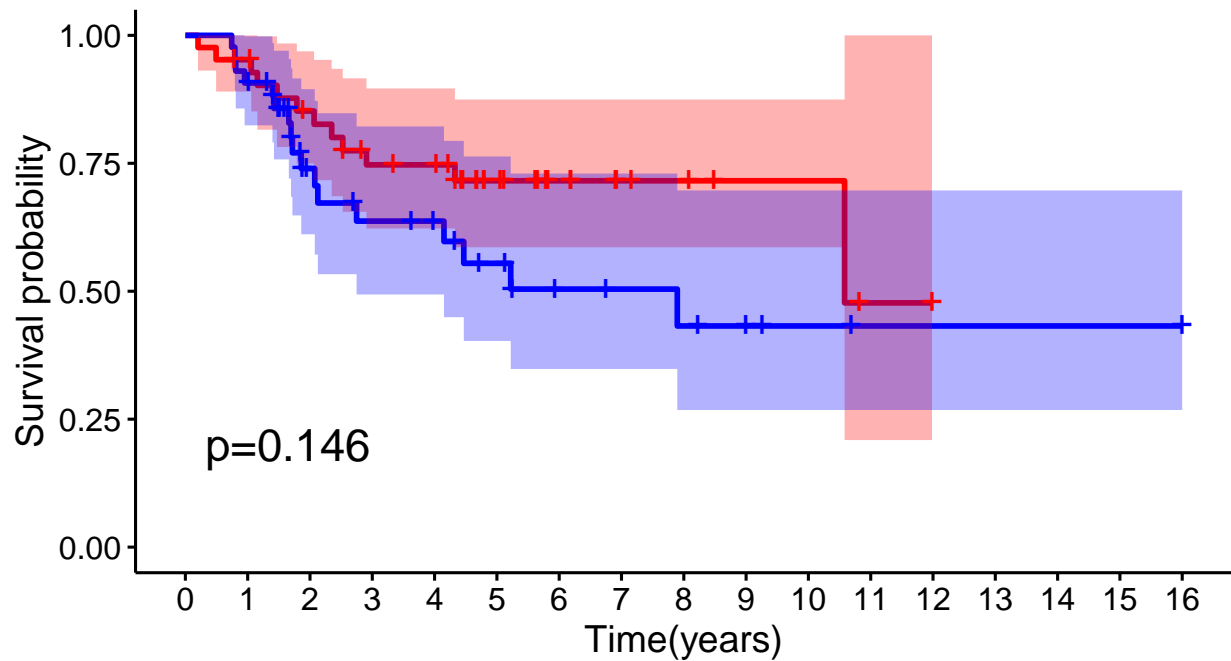

AC005037.1

high

low

|    |    |    |    |    |    |    |   |   |   |    |    |    |    |    |    |
|----|----|----|----|----|----|----|---|---|---|----|----|----|----|----|----|
| 42 | 39 | 33 | 27 | 26 | 16 | 10 | 6 | 5 | 3 | 3  | 1  | 0  | 0  | 0  | 0  |
| 43 | 39 | 22 | 18 | 16 | 12 | 8  | 7 | 6 | 4 | 2  | 1  | 1  | 1  | 1  | 1  |
| 0  | 1  | 2  | 3  | 4  | 5  | 6  | 7 | 8 | 9 | 10 | 11 | 12 | 13 | 14 | 15 |

Time(years)

Supplement: Supplementary Document 1 — Kaplan-Meier curve of the 518 genes associated with survival. [file DataSheet_1.zip › Supplementary Document 1/sur.AC005037.1.pdf]

AC005832.4

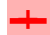

high

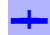

low

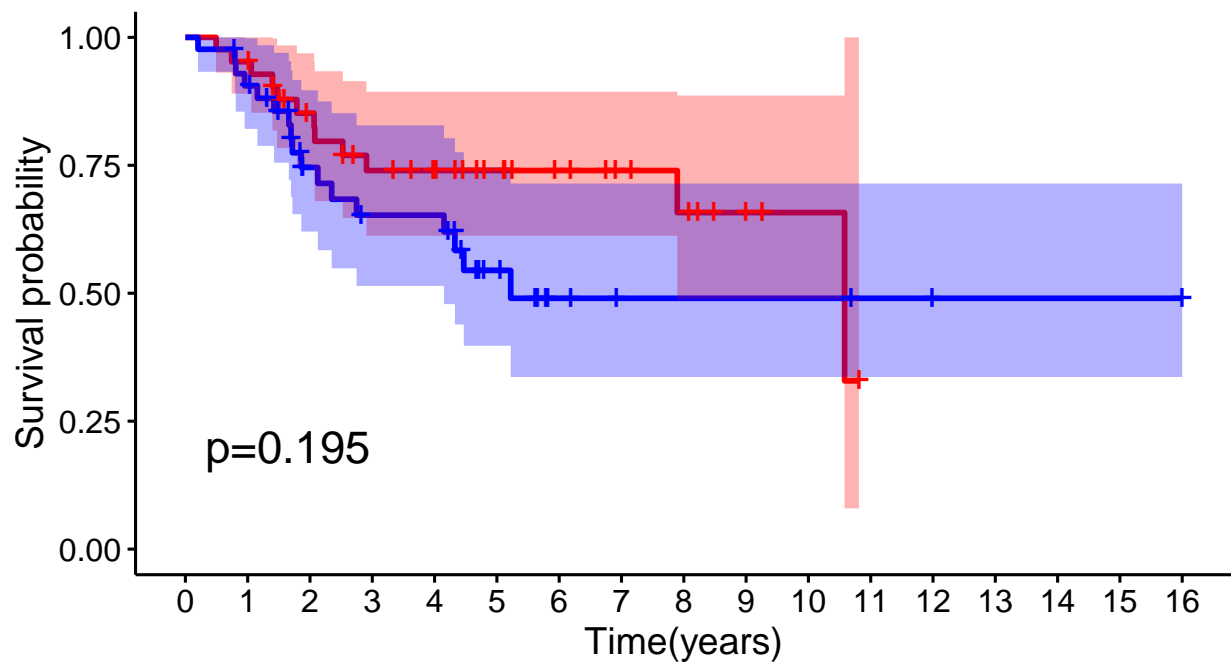

AC005832.4

high

low

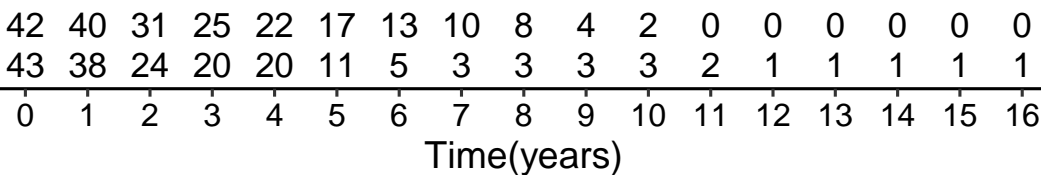

Supplement: Supplementary Document 1 — Kaplan-Meier curve of the 518 genes associated with survival. [file DataSheet_1.zip › Supplementary Document 1/sur.AC005832.4.pdf]

AC007182.1 + high + low

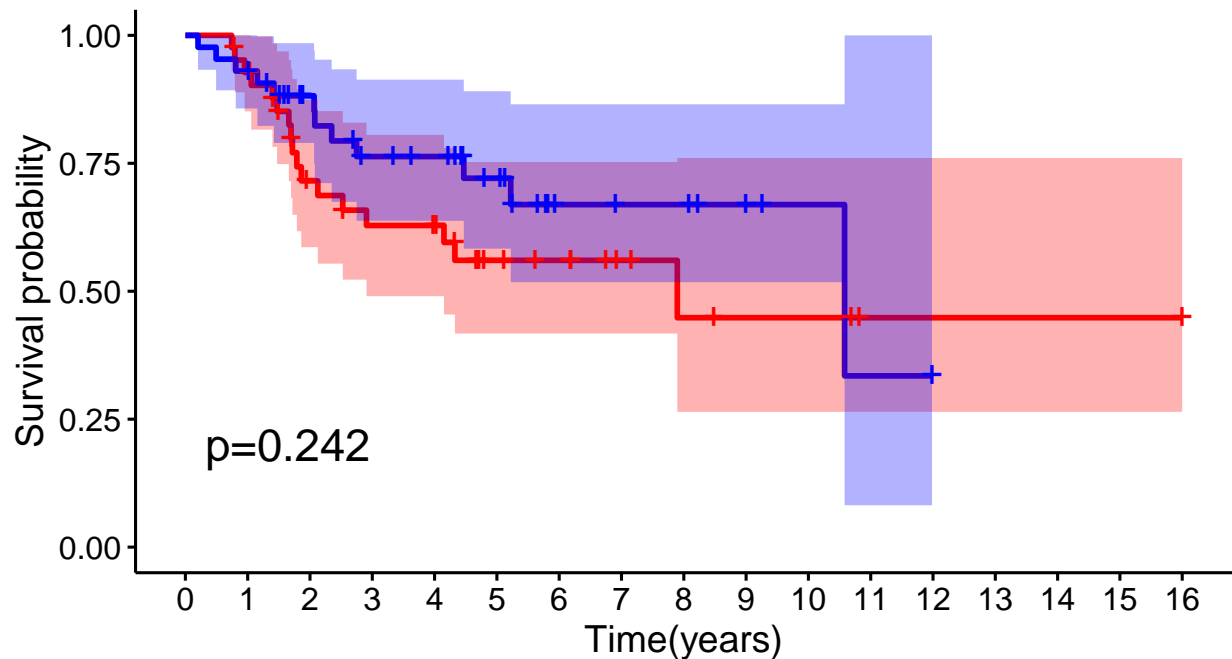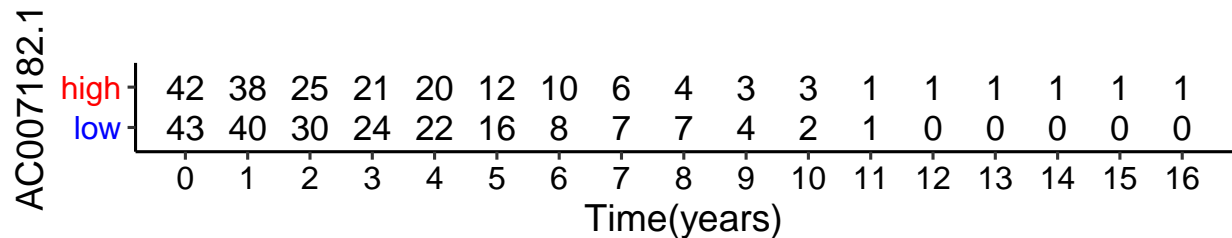

Supplement: Supplementary Document 1 — Kaplan-Meier curve of the 518 genes associated with survival. [file DataSheet_1.zip › Supplementary Document 1/sur.AC007182.1.pdf]

AC007255.1 + high + low

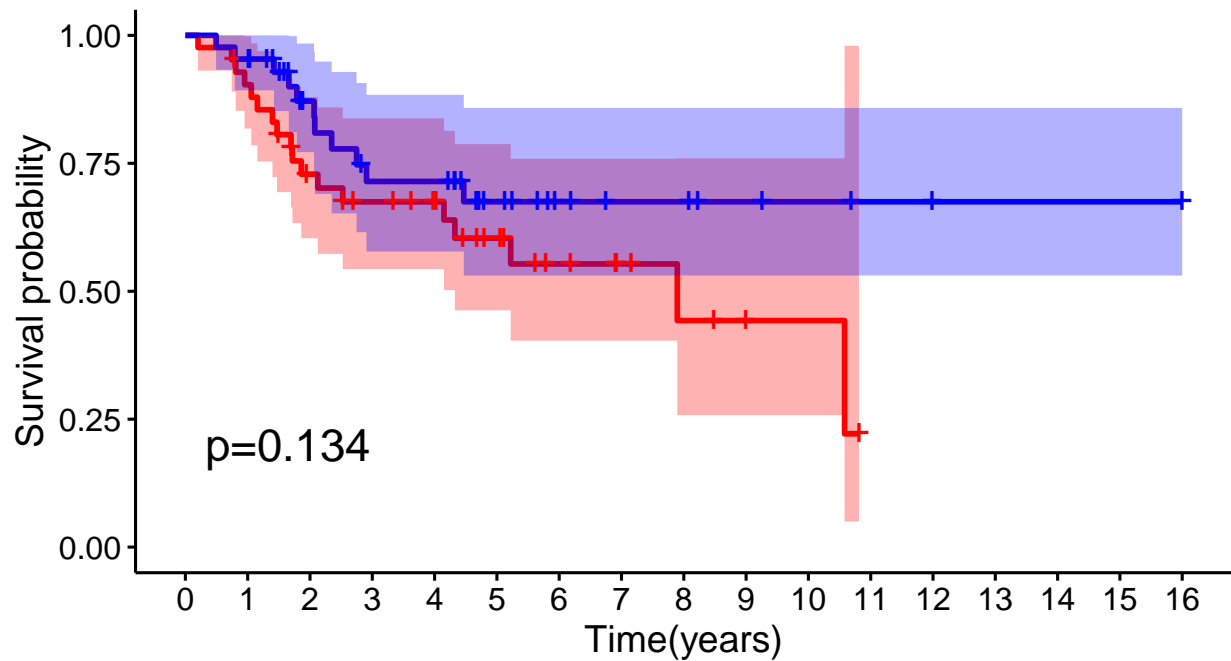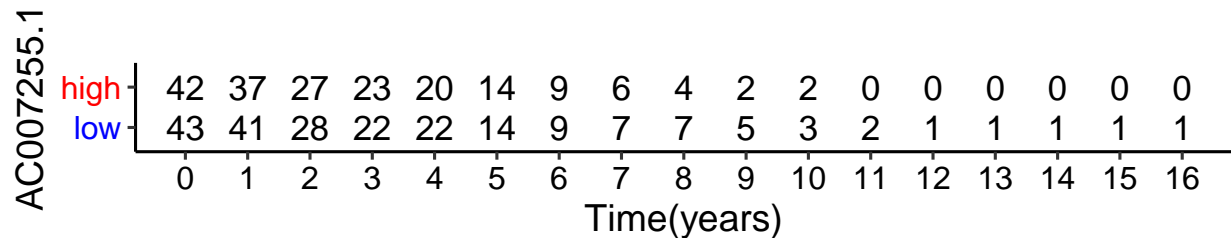

Supplement: Supplementary Document 1 — Kaplan-Meier curve of the 518 genes associated with survival. [file DataSheet_1.zip › Supplementary Document 1/sur.AC007255.1.pdf]

AC009154.2 high low

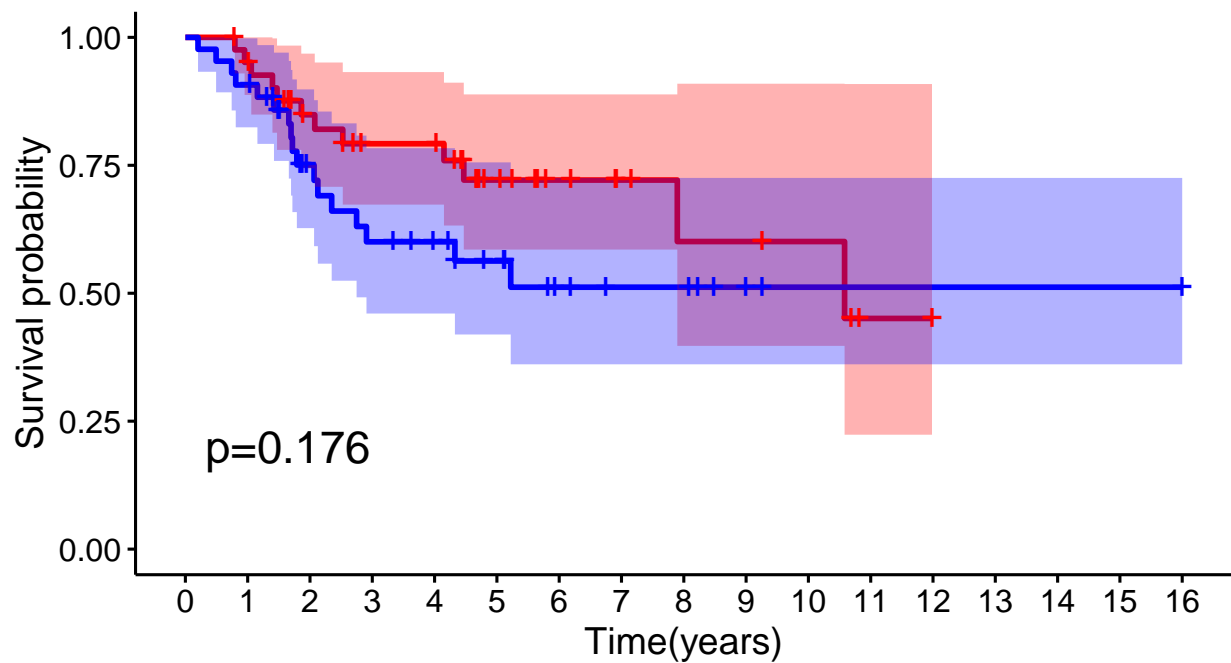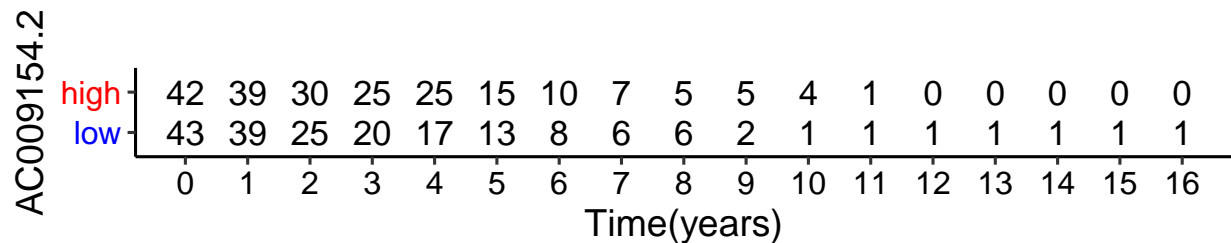

Supplement: Supplementary Document 1 — Kaplan-Meier curve of the 518 genes associated with survival. [file DataSheet_1.zip › Supplementary Document 1/sur.AC009154.2.pdf]

AC009185.1 + high + low

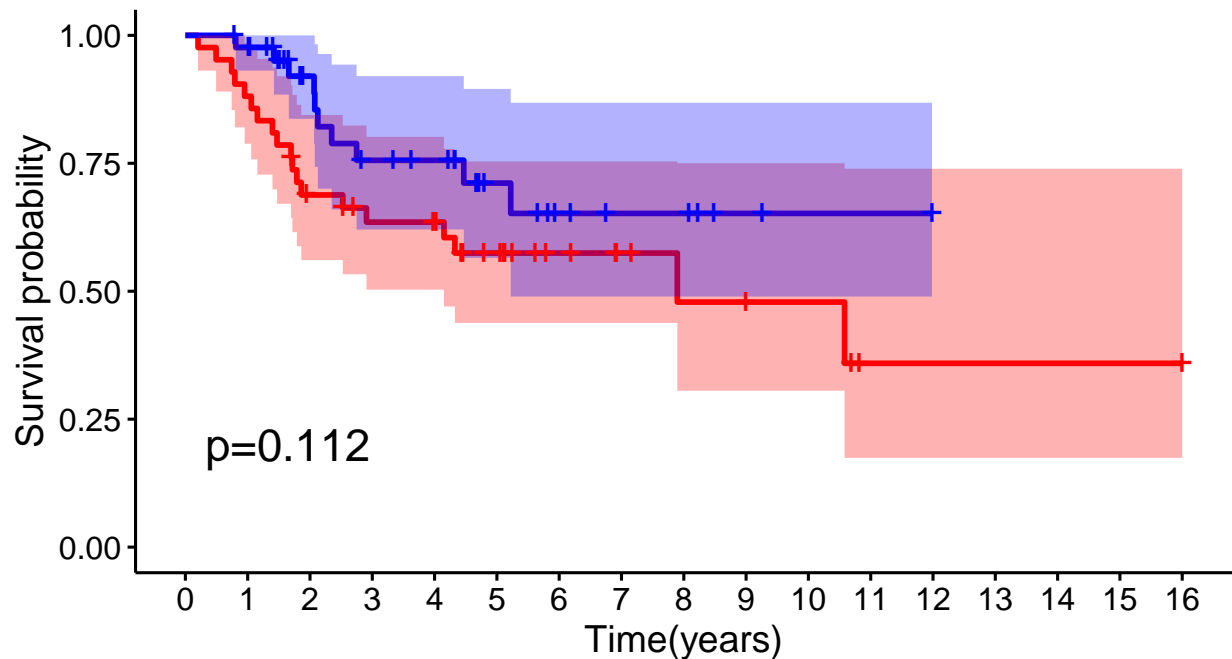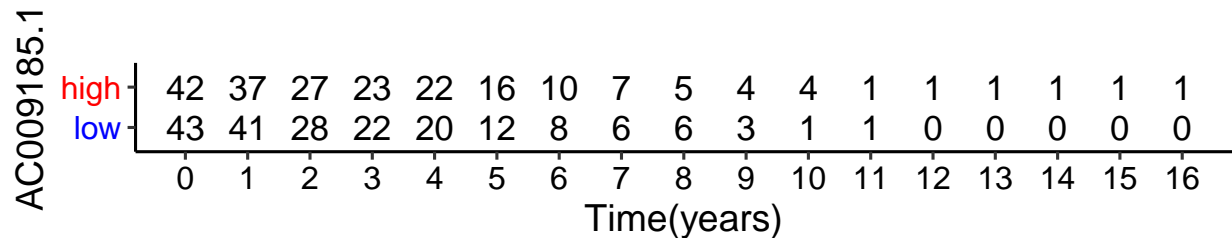

Supplement: Supplementary Document 1 — Kaplan-Meier curve of the 518 genes associated with survival. [file DataSheet_1.zip › Supplementary Document 1/sur.AC009185.1.pdf]

AC009414.2

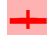

high

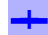

low

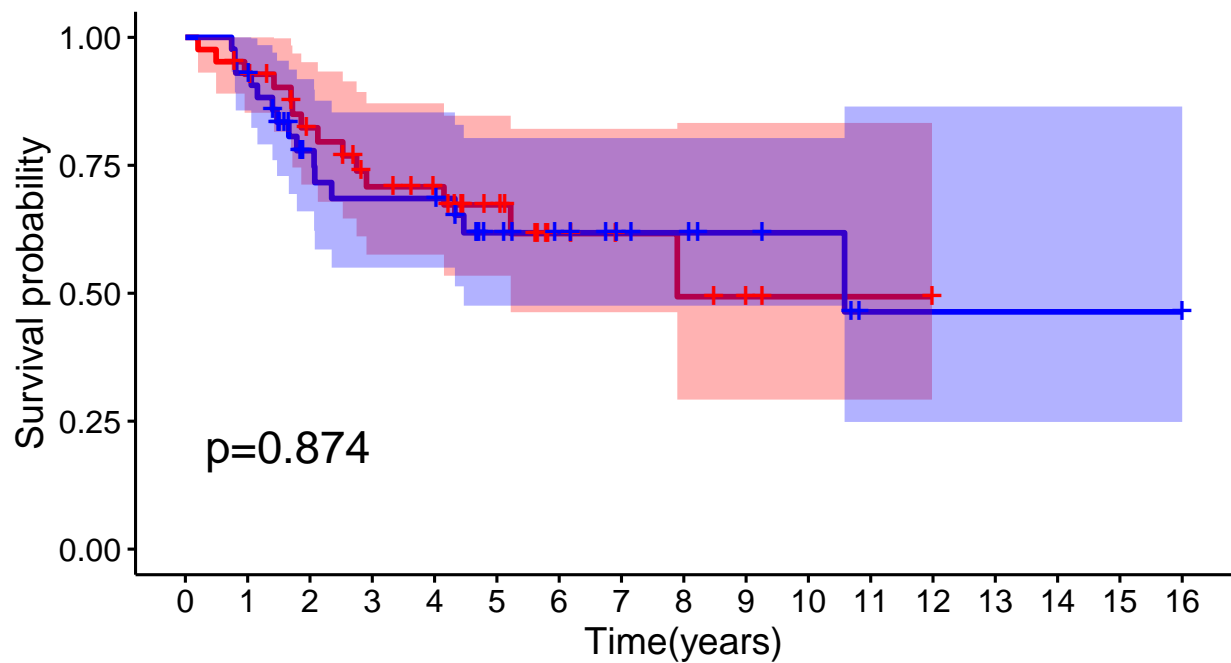

AC009414.2

high

low

|    |    |    |    |    |    |    |   |   |   |    |    |    |    |    |    |
|----|----|----|----|----|----|----|---|---|---|----|----|----|----|----|----|
| 42 | 38 | 30 | 23 | 20 | 14 | 7  | 5 | 4 | 2 | 1  | 1  | 0  | 0  | 0  | 0  |
| 43 | 40 | 25 | 22 | 22 | 14 | 11 | 8 | 7 | 5 | 4  | 1  | 1  | 1  | 1  | 1  |
| 0  | 1  | 2  | 3  | 4  | 5  | 6  | 7 | 8 | 9 | 10 | 11 | 12 | 13 | 14 | 15 |

Time(years)

Supplement: Supplementary Document 1 — Kaplan-Meier curve of the 518 genes associated with survival. [file DataSheet_1.zip › Supplementary Document 1/sur.AC009414.2.pdf]

AC009831.1

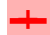

high

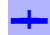

low

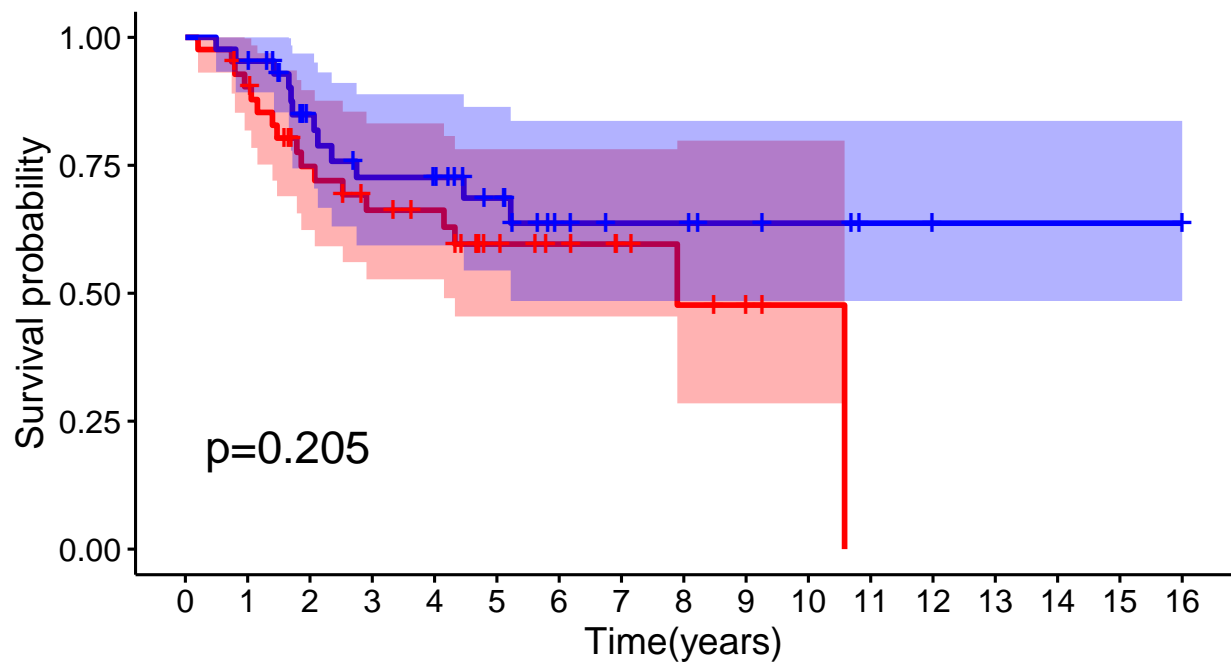

AC009831.1

high

low

|    |    |    |    |    |    |   |   |   |   |    |    |    |    |    |    |
|----|----|----|----|----|----|---|---|---|---|----|----|----|----|----|----|
| 42 | 37 | 27 | 22 | 20 | 12 | 9 | 6 | 4 | 2 | 1  | 0  | 0  | 0  | 0  | 0  |
| 43 | 41 | 28 | 23 | 22 | 16 | 9 | 7 | 7 | 5 | 4  | 2  | 1  | 1  | 1  | 1  |
| 0  | 1  | 2  | 3  | 4  | 5  | 6 | 7 | 8 | 9 | 10 | 11 | 12 | 13 | 14 | 15 |

Time(years)

Supplement: Supplementary Document 1 — Kaplan-Meier curve of the 518 genes associated with survival. [file DataSheet_1.zip › Supplementary Document 1/sur.AC009831.1.pdf]

AC010175.1 + high + low

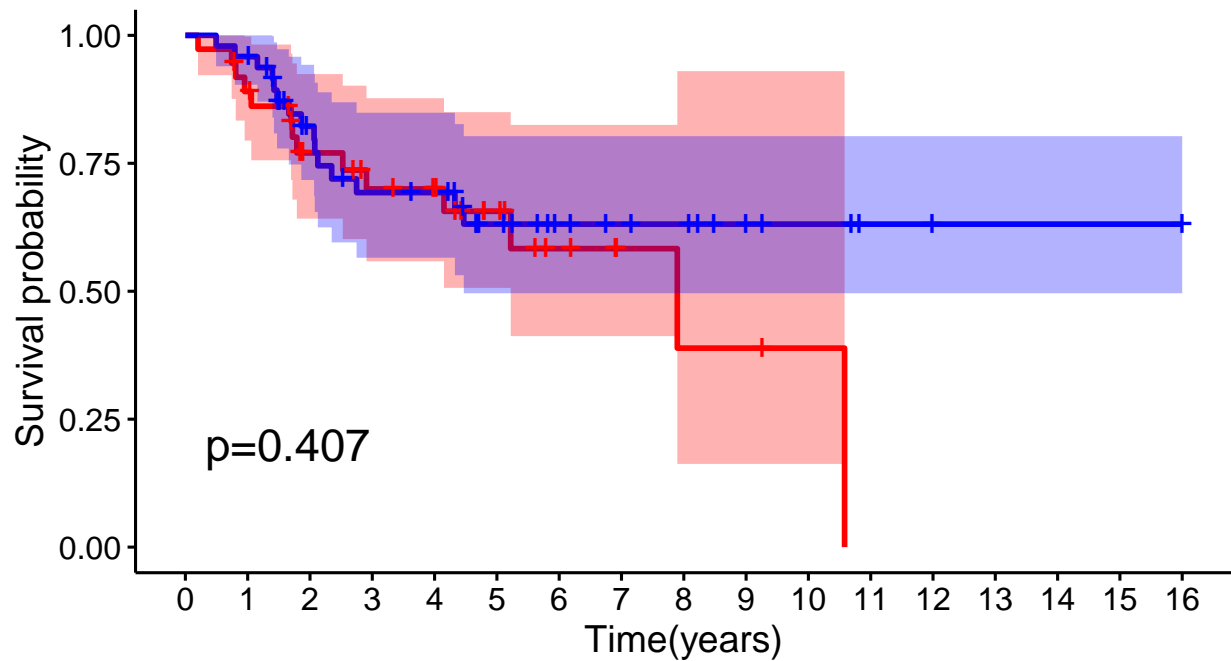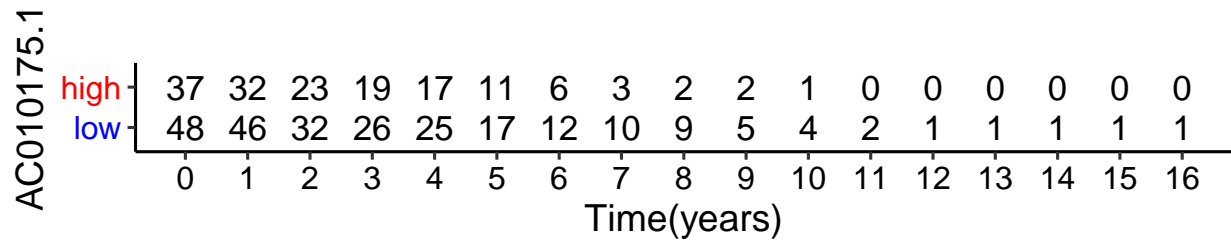

Supplement: Supplementary Document 1 — Kaplan-Meier curve of the 518 genes associated with survival. [file DataSheet_1.zip › Supplementary Document 1/sur.AC010175.1.pdf]

AC010609.1

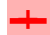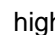

high low

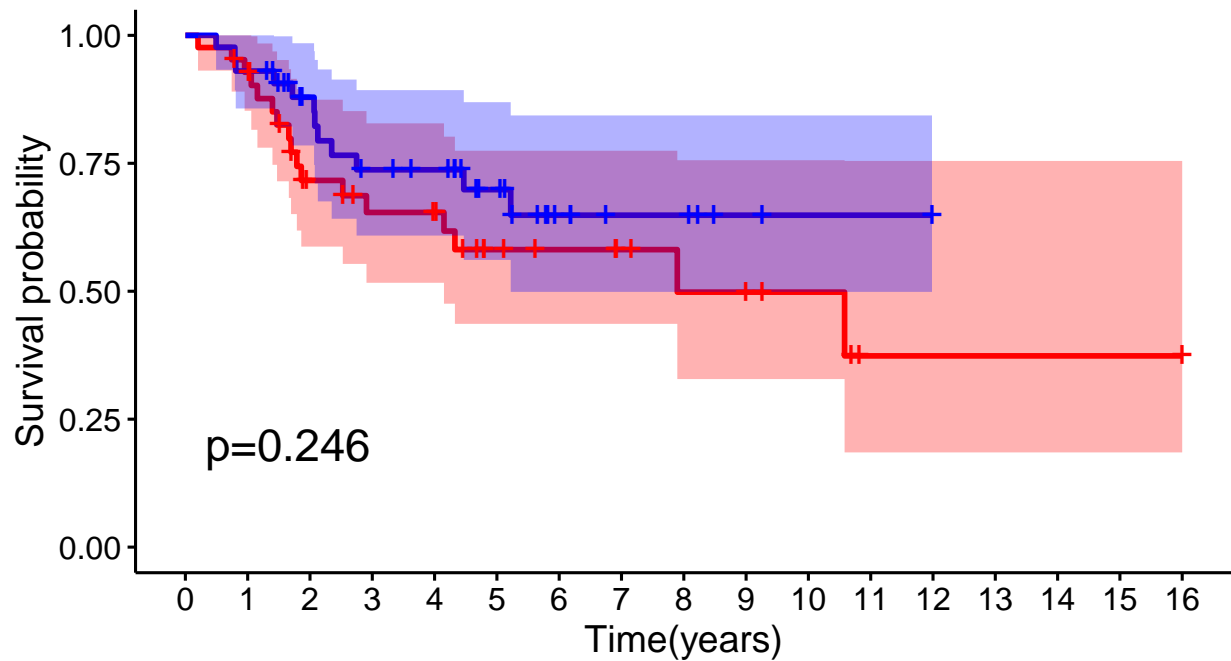

AC010609.1

high

low

|    |    |    |    |    |    |    |   |   |   |    |    |    |    |    |    |
|----|----|----|----|----|----|----|---|---|---|----|----|----|----|----|----|
| 42 | 38 | 24 | 20 | 19 | 12 | 10 | 8 | 6 | 5 | 4  | 1  | 1  | 1  | 1  | 1  |
| 43 | 40 | 31 | 25 | 23 | 16 | 8  | 5 | 5 | 2 | 1  | 1  | 0  | 0  | 0  | 0  |
| 0  | 1  | 2  | 3  | 4  | 5  | 6  | 7 | 8 | 9 | 10 | 11 | 12 | 13 | 14 | 15 |

Time(years)

Supplement: Supplementary Document 1 — Kaplan-Meier curve of the 518 genes associated with survival. [file DataSheet_1.zip › Supplementary Document 1/sur.AC010609.1.pdf]

AC012363.2

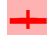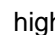

high low

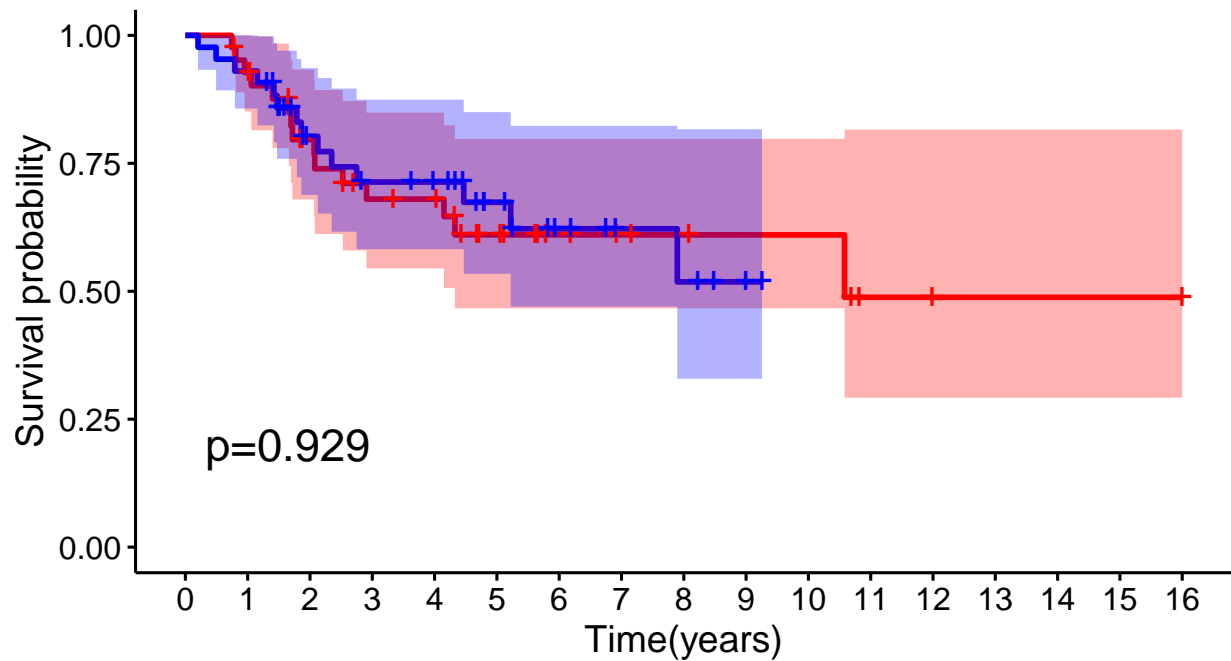

AC012363.2

high

low

|    |    |    |    |    |    |   |   |   |   |    |    |    |    |    |    |
|----|----|----|----|----|----|---|---|---|---|----|----|----|----|----|----|
| 42 | 38 | 28 | 22 | 21 | 14 | 9 | 7 | 6 | 5 | 5  | 2  | 1  | 1  | 1  | 1  |
| 43 | 40 | 27 | 23 | 21 | 14 | 9 | 6 | 5 | 2 | 0  | 0  | 0  | 0  | 0  | 0  |
| 0  | 1  | 2  | 3  | 4  | 5  | 6 | 7 | 8 | 9 | 10 | 11 | 12 | 13 | 14 | 15 |

Time(years)

Supplement: Supplementary Document 1 — Kaplan-Meier curve of the 518 genes associated with survival. [file DataSheet_1.zip › Supplementary Document 1/sur.AC012363.2.pdf]

AC016717.2

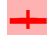

high

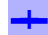

low

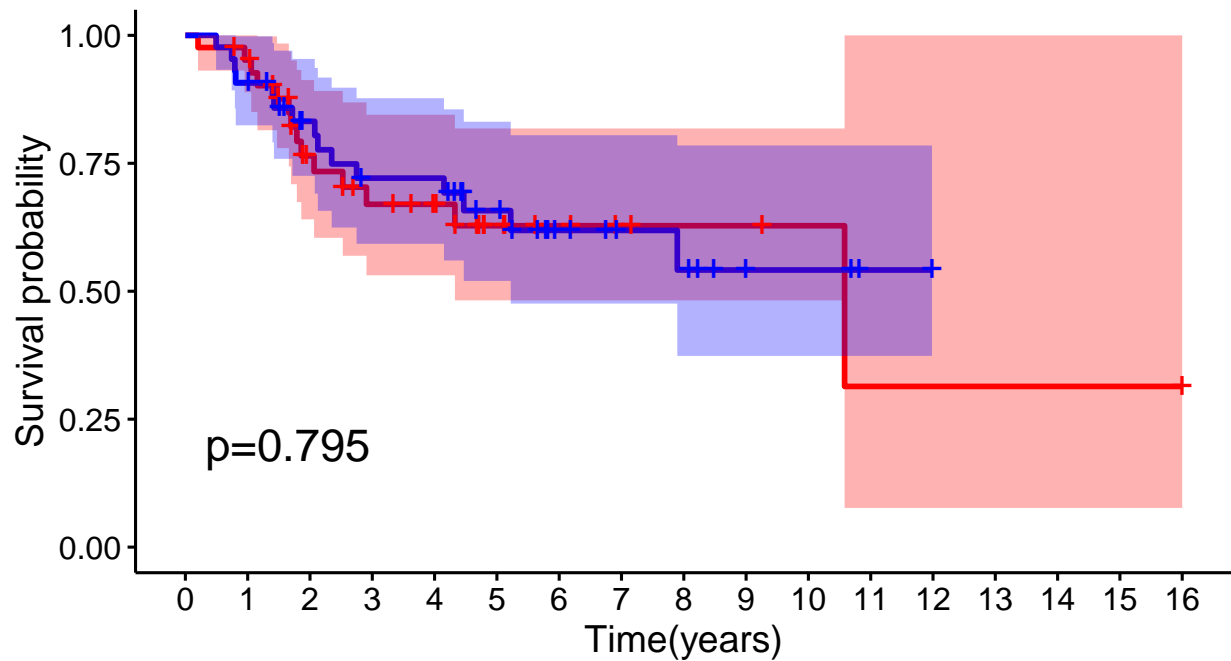

AC016717.2

high

low

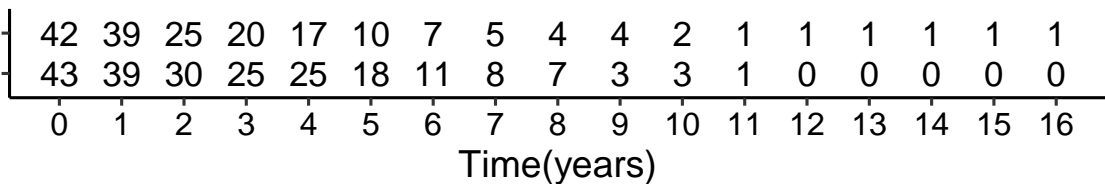

Supplement: Supplementary Document 1 — Kaplan-Meier curve of the 518 genes associated with survival. [file DataSheet_1.zip › Supplementary Document 1/sur.AC016717.2.pdf]

AC016813.1

+ high + low

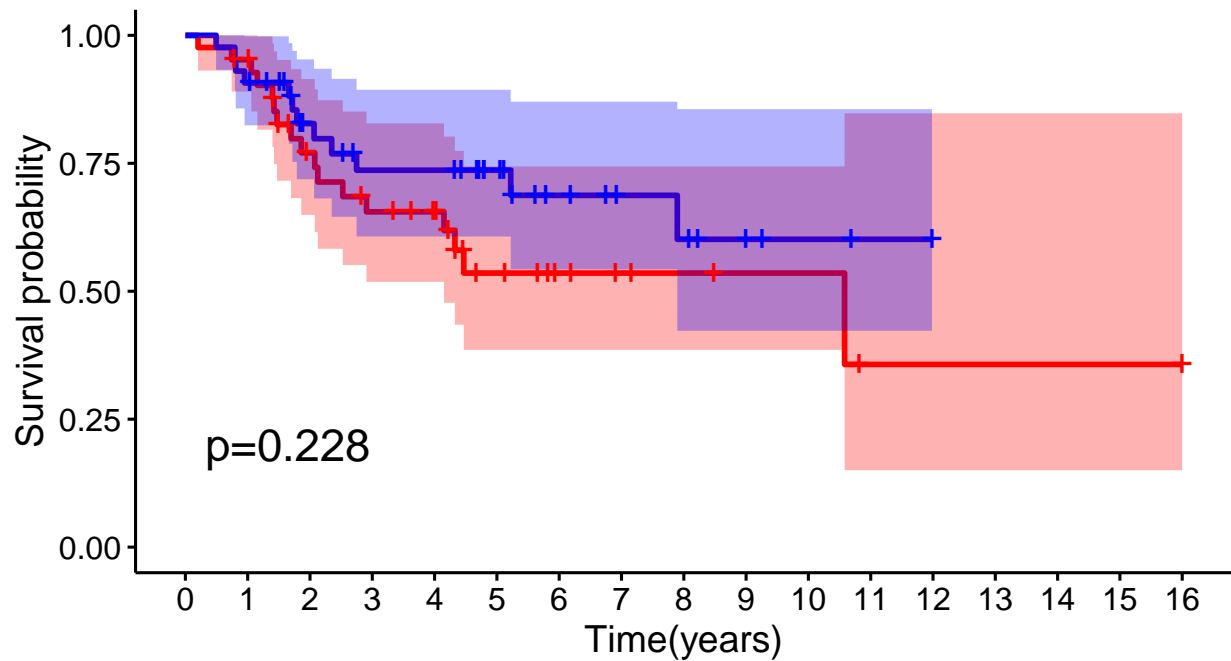

AC016813.1

high

low

|    |    |    |    |    |    |    |   |   |   |    |    |    |    |    |    |
|----|----|----|----|----|----|----|---|---|---|----|----|----|----|----|----|
| 42 | 39 | 27 | 22 | 19 | 11 | 7  | 5 | 4 | 3 | 3  | 1  | 1  | 1  | 1  | 1  |
| 43 | 39 | 28 | 23 | 23 | 17 | 11 | 8 | 7 | 4 | 2  | 1  | 0  | 0  | 0  | 0  |
| 0  | 1  | 2  | 3  | 4  | 5  | 6  | 7 | 8 | 9 | 10 | 11 | 12 | 13 | 14 | 15 |

Time(years)

Supplement: Supplementary Document 1 — Kaplan-Meier curve of the 518 genes associated with survival. [file DataSheet_1.zip › Supplementary Document 1/sur.AC016813.1.pdf]

AC017033.1 + high + low

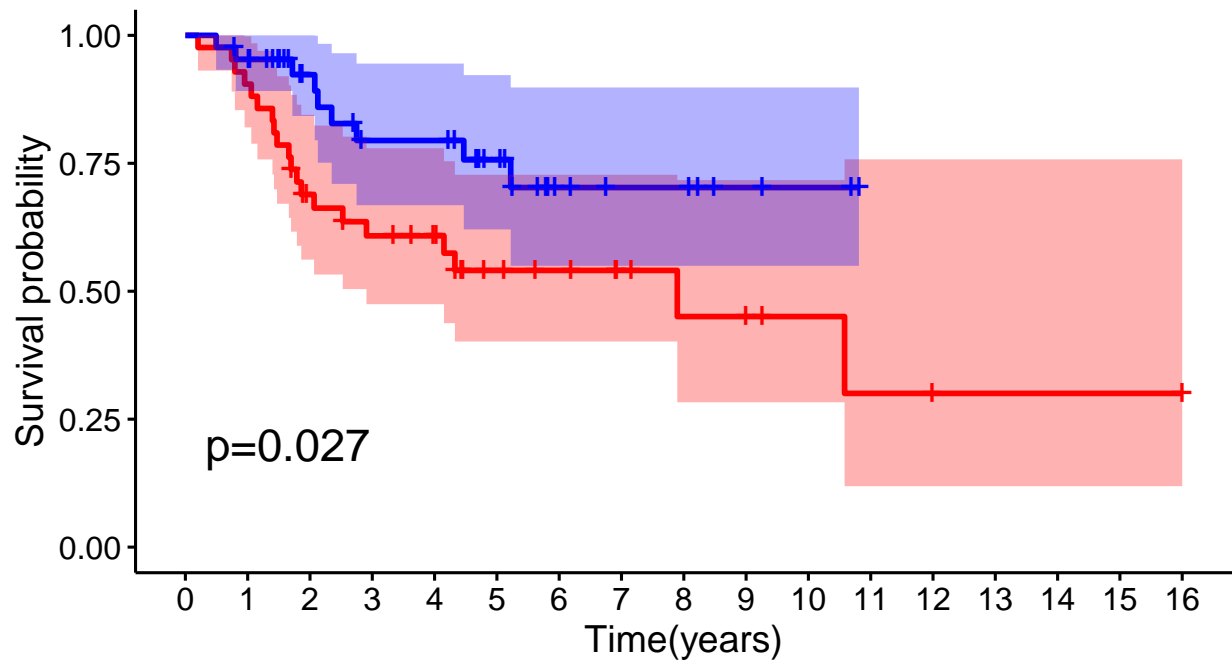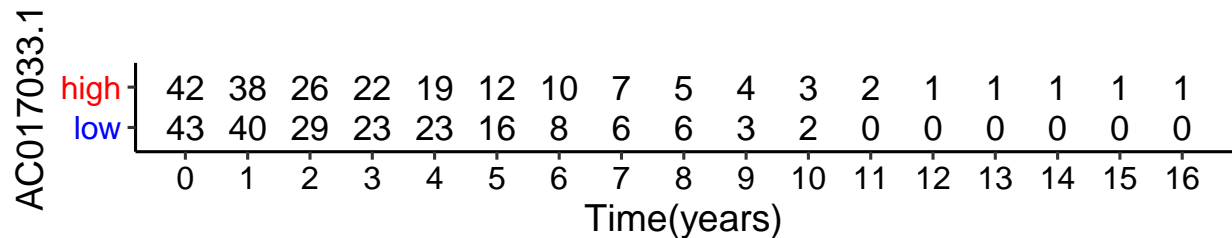

Supplement: Supplementary Document 1 — Kaplan-Meier curve of the 518 genes associated with survival. [file DataSheet_1.zip › Supplementary Document 1/sur.AC017033.1.pdf]

AC019197.1 + high + low

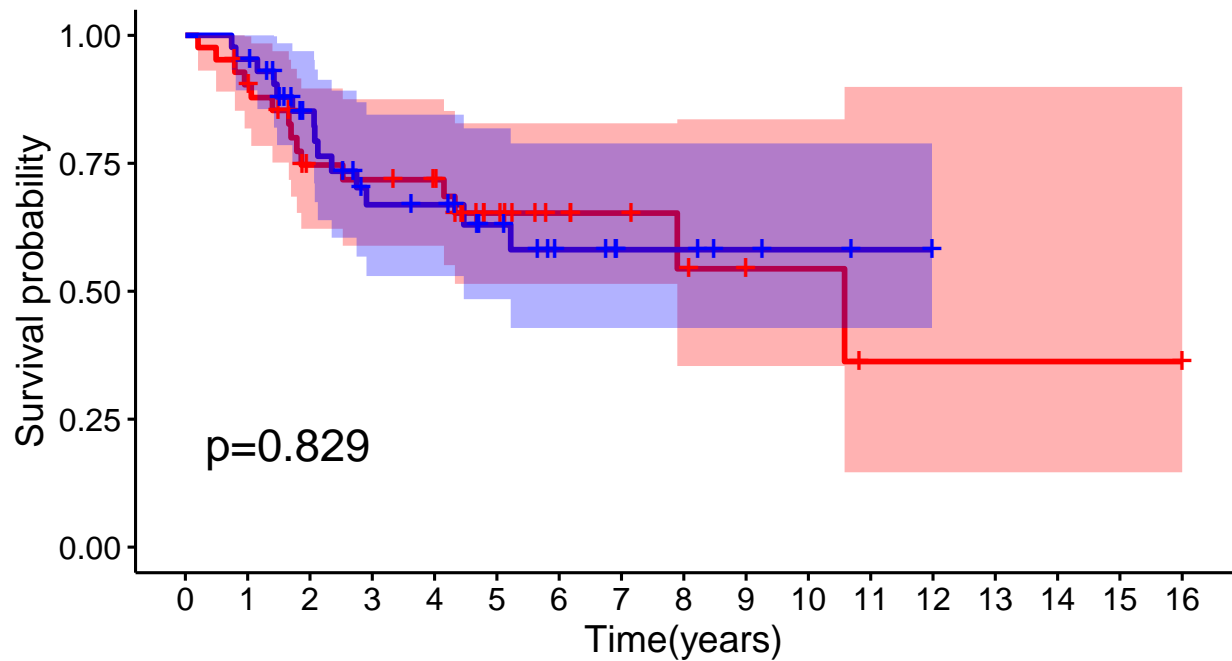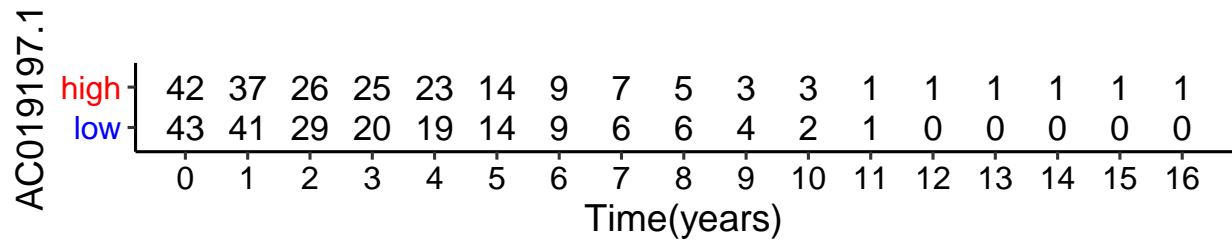

Supplement: Supplementary Document 1 — Kaplan-Meier curve of the 518 genes associated with survival. [file DataSheet_1.zip › Supplementary Document 1/sur.AC019197.1.pdf]

AC020571.1

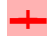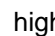

high low

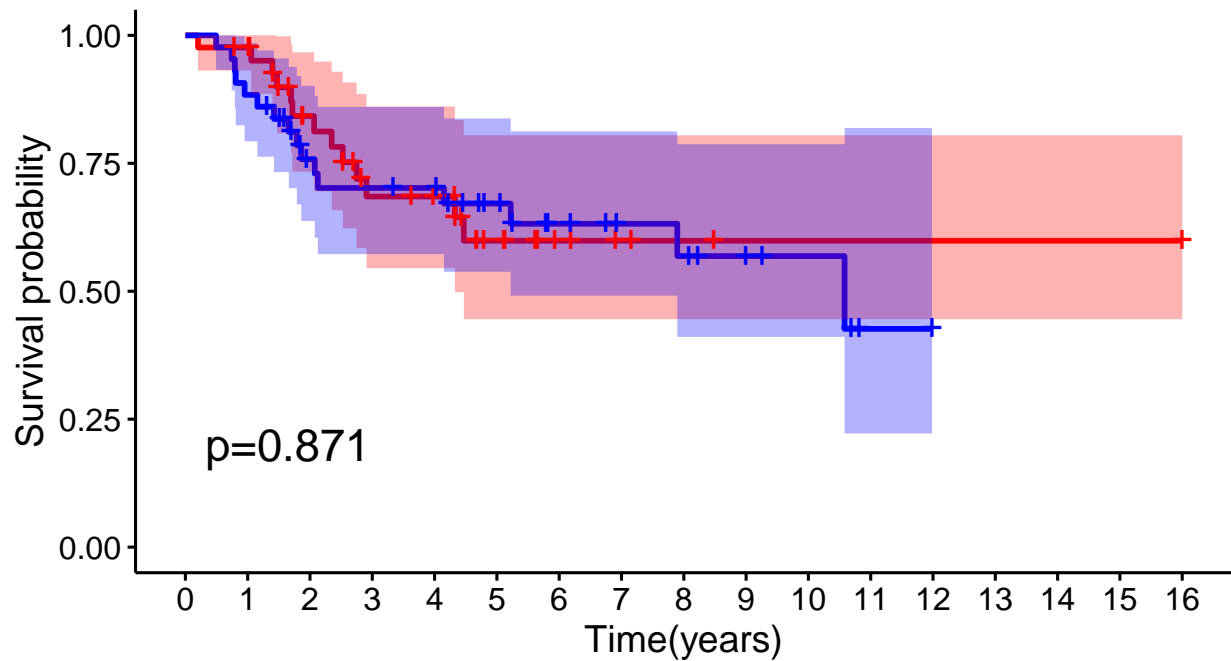

AC020571.1

high

low

|    |    |    |    |    |    |    |    |   |   |    |    |    |    |    |    |
|----|----|----|----|----|----|----|----|---|---|----|----|----|----|----|----|
| 42 | 40 | 28 | 20 | 18 | 10 | 5  | 3  | 2 | 1 | 1  | 1  | 1  | 1  | 1  | 1  |
| 43 | 38 | 27 | 25 | 24 | 18 | 13 | 10 | 9 | 6 | 4  | 1  | 0  | 0  | 0  | 0  |
| 0  | 1  | 2  | 3  | 4  | 5  | 6  | 7  | 8 | 9 | 10 | 11 | 12 | 13 | 14 | 15 |

Time(years)

Supplement: Supplementary Document 1 — Kaplan-Meier curve of the 518 genes associated with survival. [file DataSheet_1.zip › Supplementary Document 1/sur.AC020571.1.pdf]

AC020718.1 high low

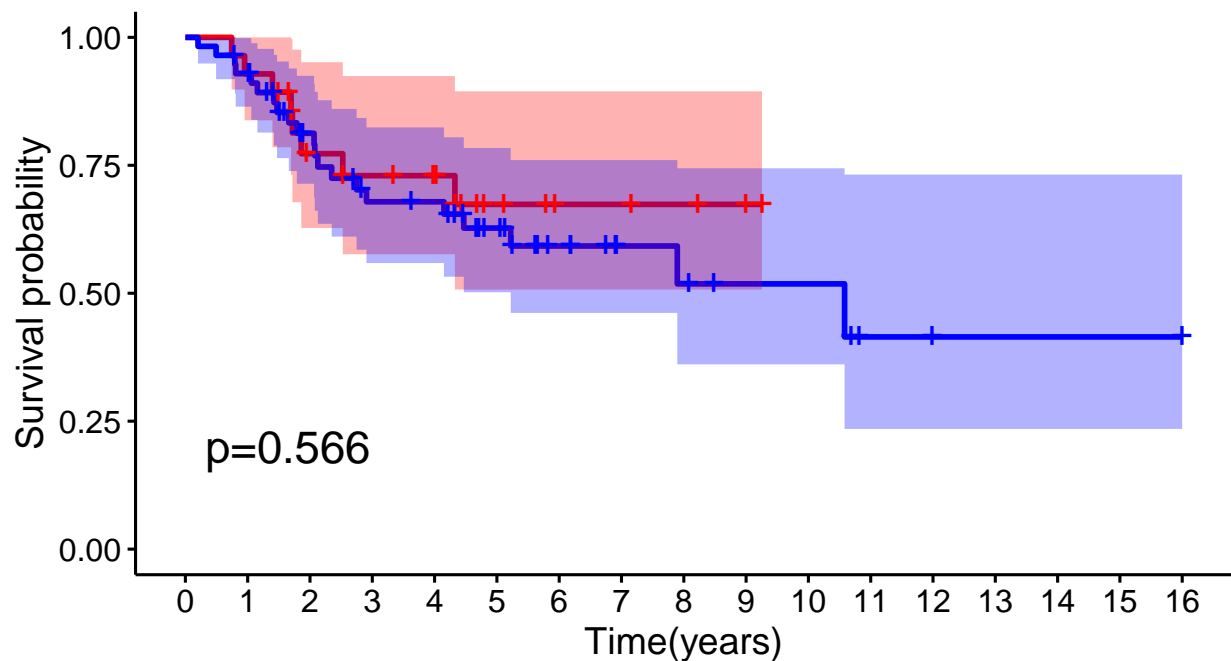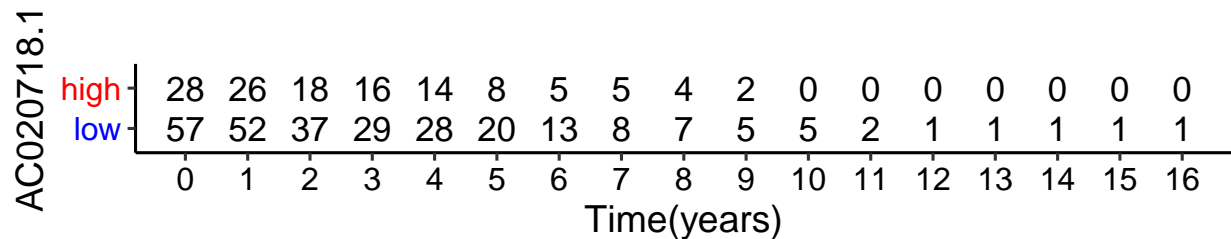

Supplement: Supplementary Document 1 — Kaplan-Meier curve of the 518 genes associated with survival. [file DataSheet_1.zip › Supplementary Document 1/sur.AC020718.1.pdf]

- low

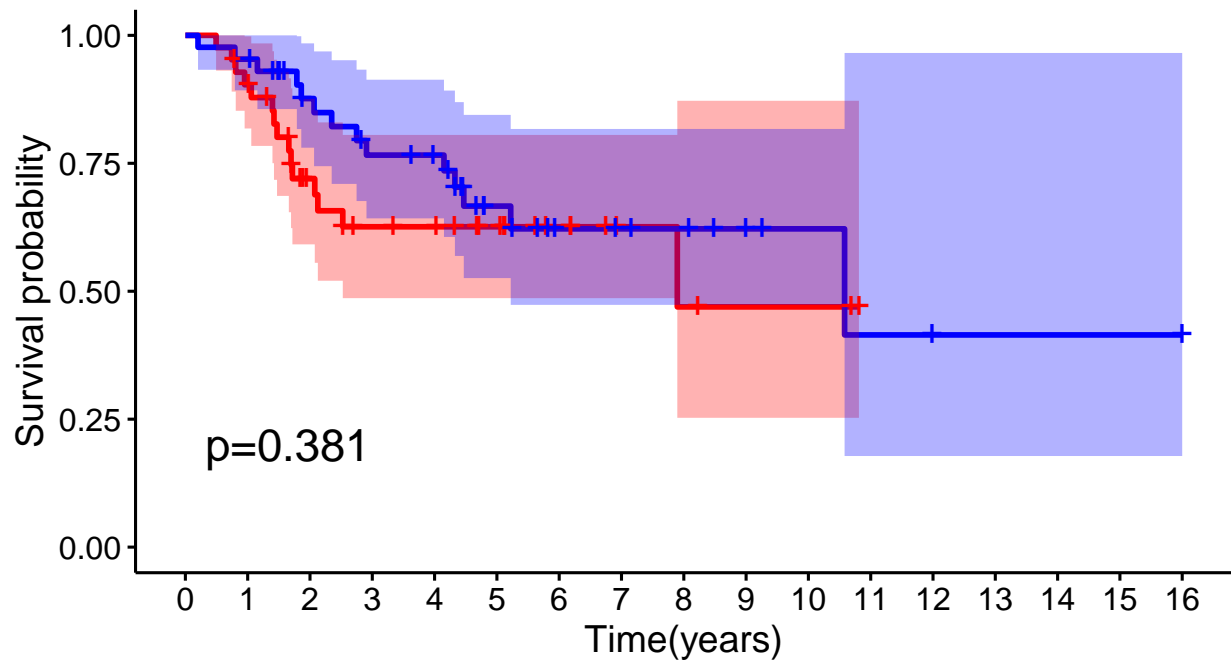

AC020913.2

high

low

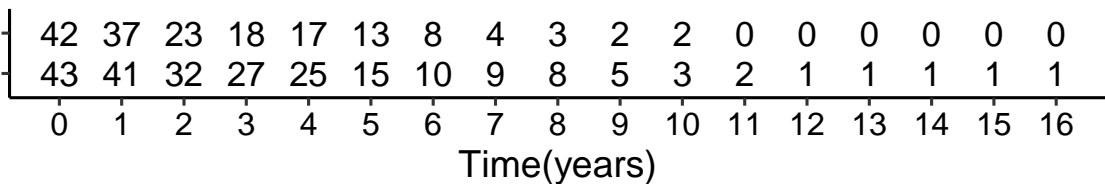

Supplement: Supplementary Document 1 — Kaplan-Meier curve of the 518 genes associated with survival. [file DataSheet_1.zip › Supplementary Document 1/sur.AC020913.2.pdf]

AC021242.3

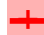

high

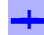

low

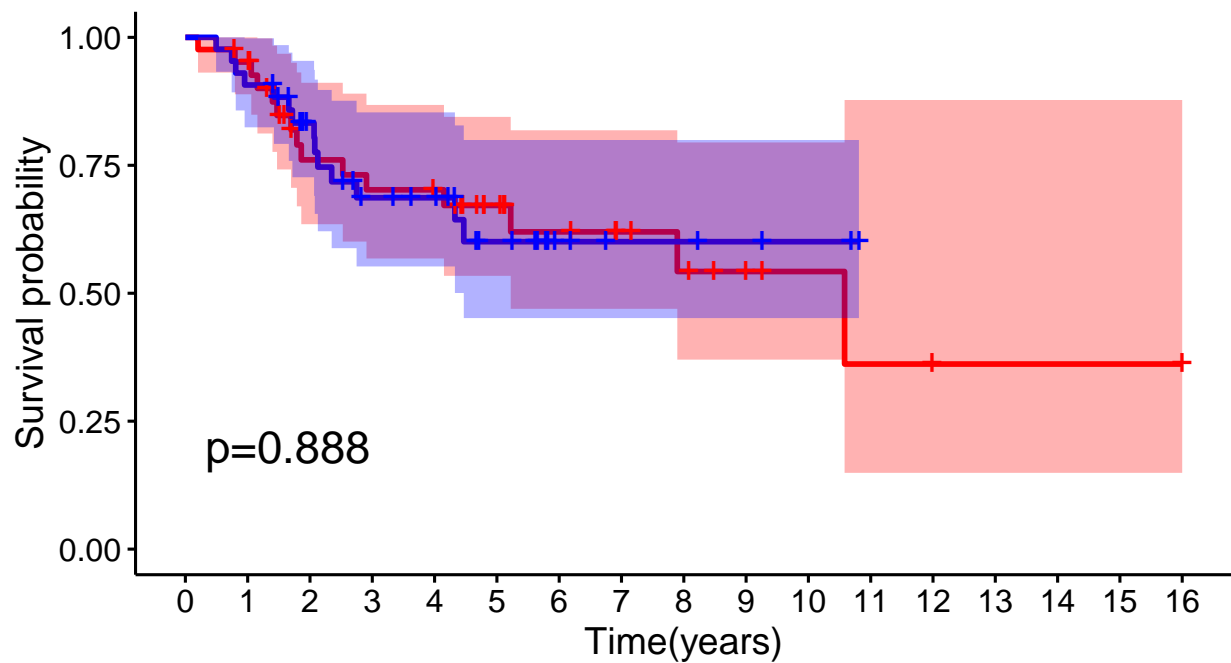

AC021242.3

high

low

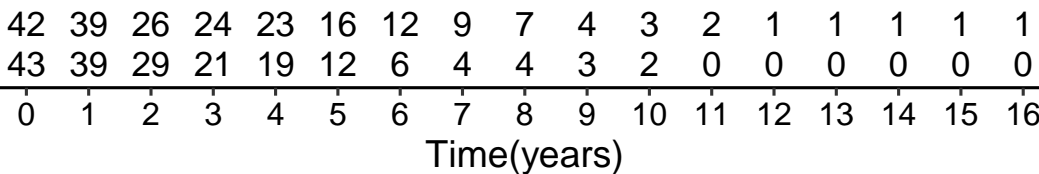

Supplement: Supplementary Document 1 — Kaplan-Meier curve of the 518 genes associated with survival. [file DataSheet_1.zip › Supplementary Document 1/sur.AC021242.3.pdf]

AC025754.2

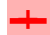

high

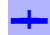

low

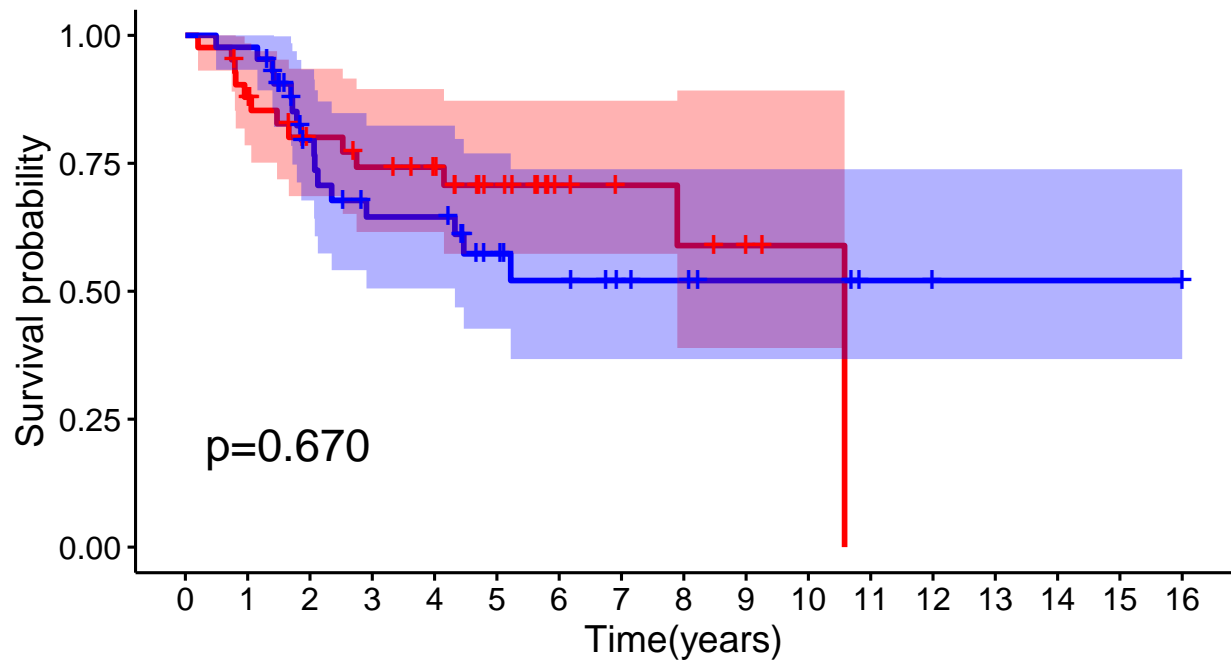

AC025754.2

high

low

|    |    |    |    |    |    |    |   |   |   |    |    |    |    |    |    |
|----|----|----|----|----|----|----|---|---|---|----|----|----|----|----|----|
| 42 | 36 | 28 | 25 | 22 | 15 | 8  | 6 | 5 | 3 | 1  | 0  | 0  | 0  | 0  | 0  |
| 43 | 42 | 27 | 20 | 20 | 13 | 10 | 7 | 6 | 4 | 4  | 2  | 1  | 1  | 1  | 1  |
| 0  | 1  | 2  | 3  | 4  | 5  | 6  | 7 | 8 | 9 | 10 | 11 | 12 | 13 | 14 | 15 |

Time(years)

Supplement: Supplementary Document 1 — Kaplan-Meier curve of the 518 genes associated with survival. [file DataSheet_1.zip › Supplementary Document 1/sur.AC025754.2.pdf]

AC037198.2

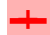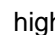

high low

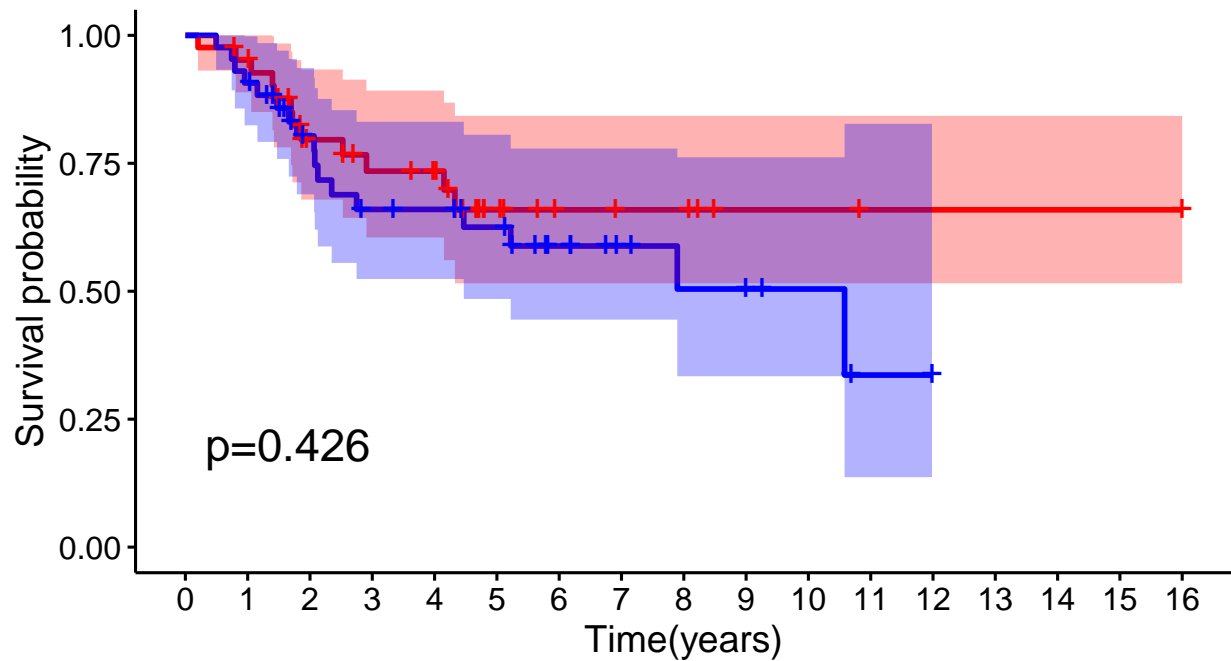

AC037198.2

high

low

|    |    |    |    |    |    |    |   |   |   |    |    |    |    |    |    |
|----|----|----|----|----|----|----|---|---|---|----|----|----|----|----|----|
| 42 | 39 | 27 | 23 | 21 | 10 | 6  | 5 | 5 | 2 | 2  | 1  | 1  | 1  | 1  | 1  |
| 43 | 39 | 28 | 22 | 21 | 18 | 12 | 8 | 6 | 5 | 3  | 1  | 0  | 0  | 0  | 0  |
| 0  | 1  | 2  | 3  | 4  | 5  | 6  | 7 | 8 | 9 | 10 | 11 | 12 | 13 | 14 | 15 |

Time(years)

Supplement: Supplementary Document 1 — Kaplan-Meier curve of the 518 genes associated with survival. [file DataSheet_1.zip › Supplementary Document 1/sur.AC037198.2.pdf]

AC037450.1 + high + low

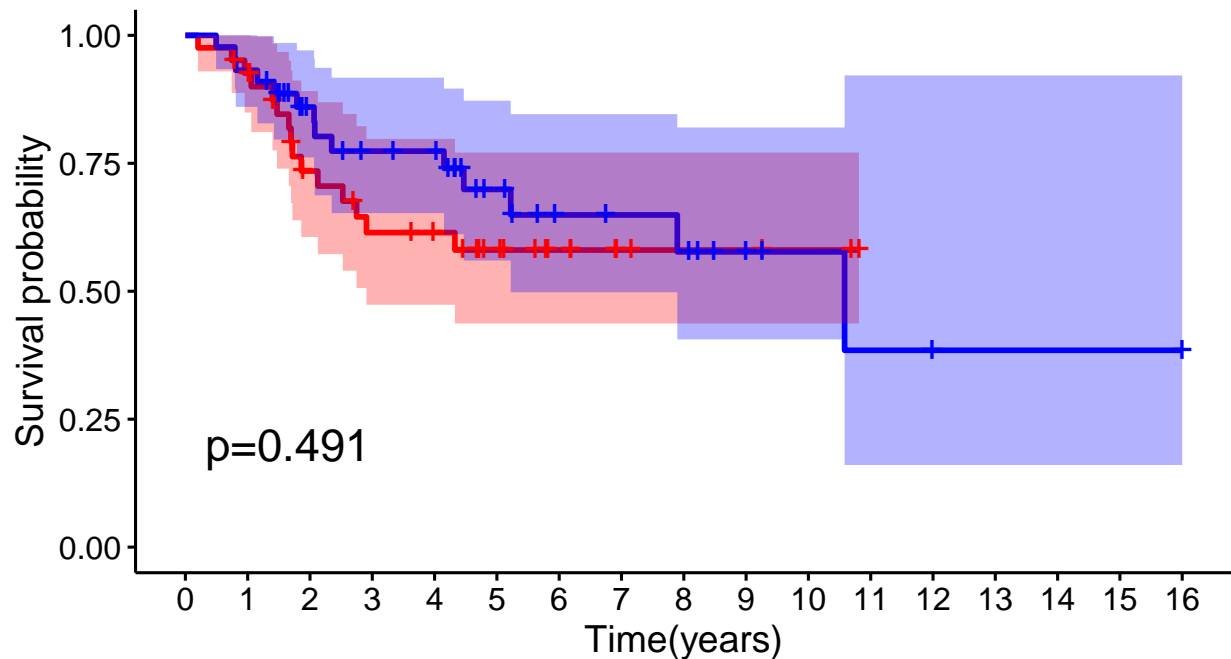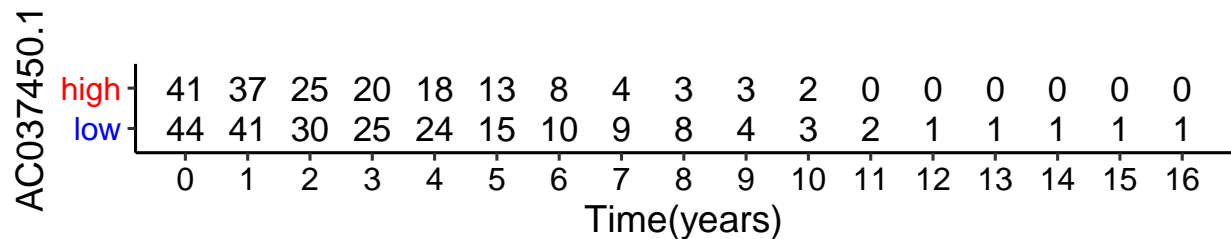

Supplement: Supplementary Document 1 — Kaplan-Meier curve of the 518 genes associated with survival. [file DataSheet_1.zip › Supplementary Document 1/sur.AC037450.1.pdf]

AC046195.1 + high + low

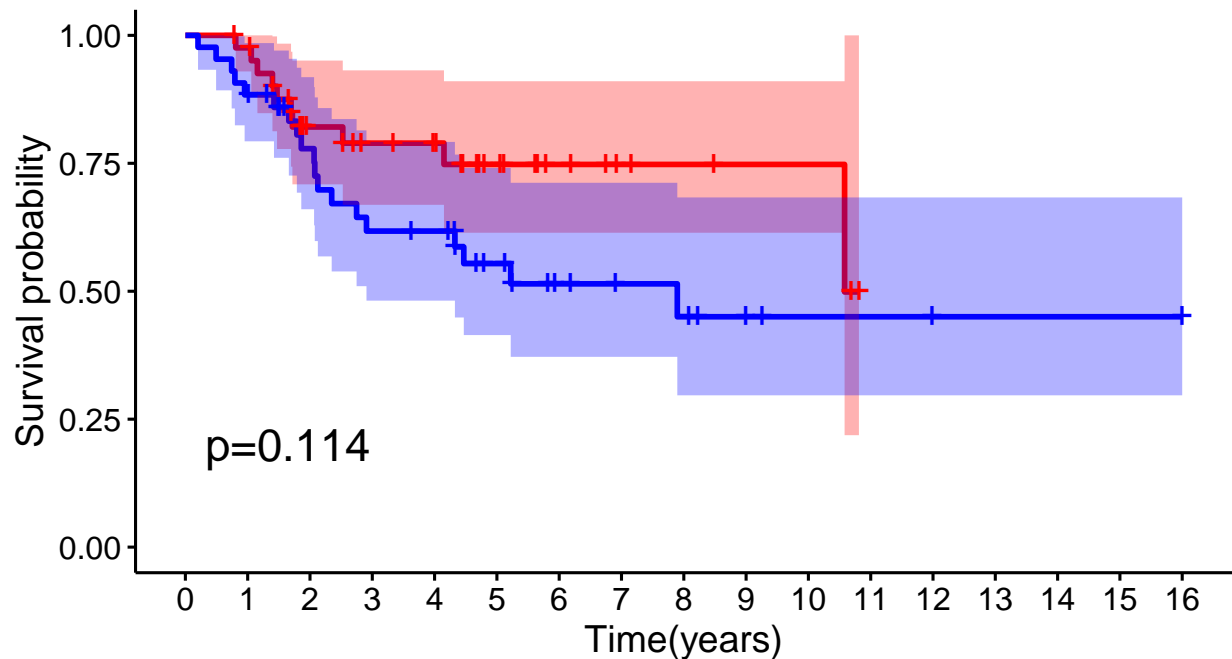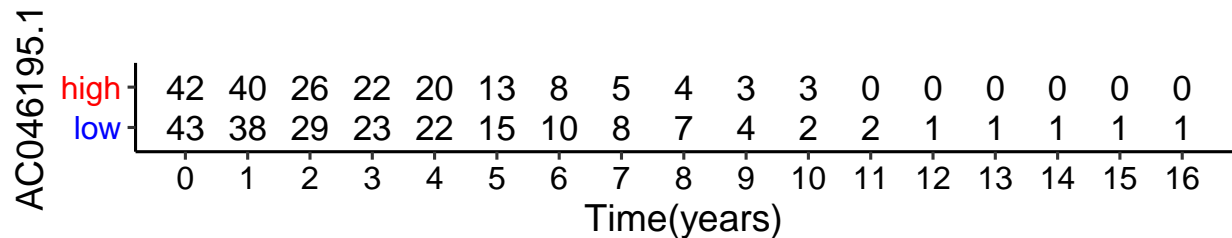

Supplement: Supplementary Document 1 — Kaplan-Meier curve of the 518 genes associated with survival. [file DataSheet_1.zip › Supplementary Document 1/sur.AC046195.1.pdf]

AC068234.2

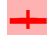

high

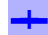

low

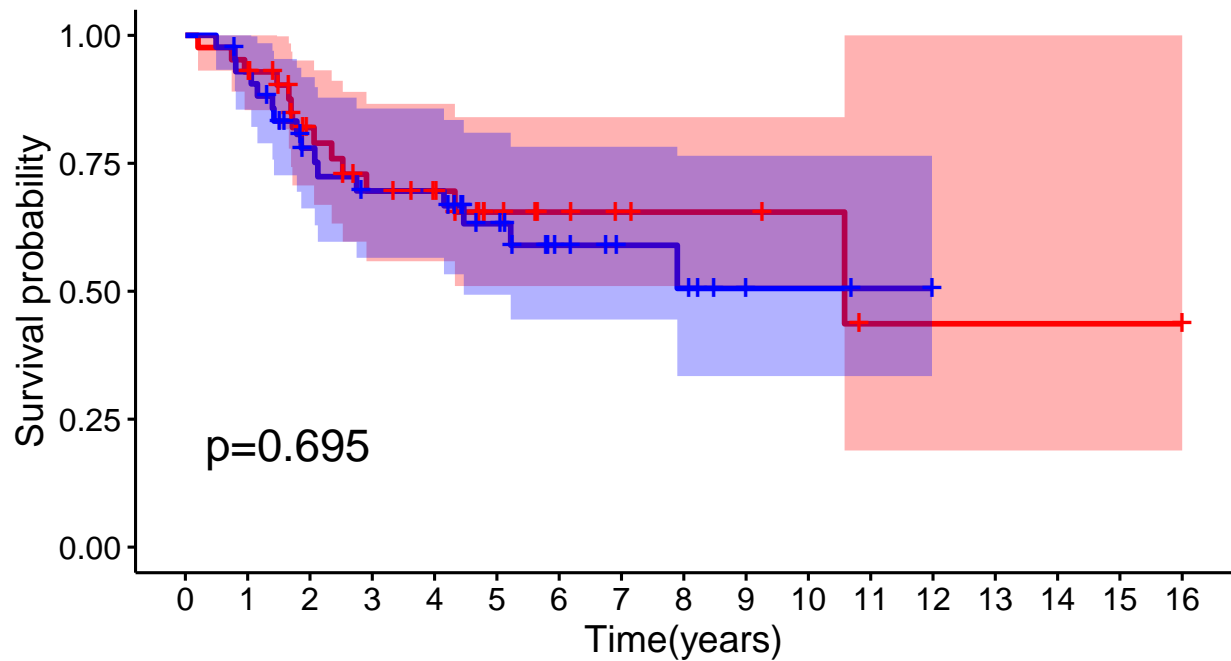

AC068234.2

high

low

|    |    |    |    |    |    |    |   |   |   |    |    |    |    |    |    |    |
|----|----|----|----|----|----|----|---|---|---|----|----|----|----|----|----|----|
| 0  | 1  | 2  | 3  | 4  | 5  | 6  | 7 | 8 | 9 | 10 | 11 | 12 | 13 | 14 | 15 | 16 |
| 42 | 39 | 27 | 21 | 18 | 11 | 8  | 6 | 5 | 5 | 3  | 1  | 1  | 1  | 1  | 1  | 1  |
| 43 | 39 | 28 | 24 | 24 | 17 | 10 | 7 | 6 | 2 | 2  | 1  | 0  | 0  | 0  | 0  | 0  |

Time(years)

Supplement: Supplementary Document 1 — Kaplan-Meier curve of the 518 genes associated with survival. [file DataSheet_1.zip › Supplementary Document 1/sur.AC068234.2.pdf]

AC078845.1 + high + low

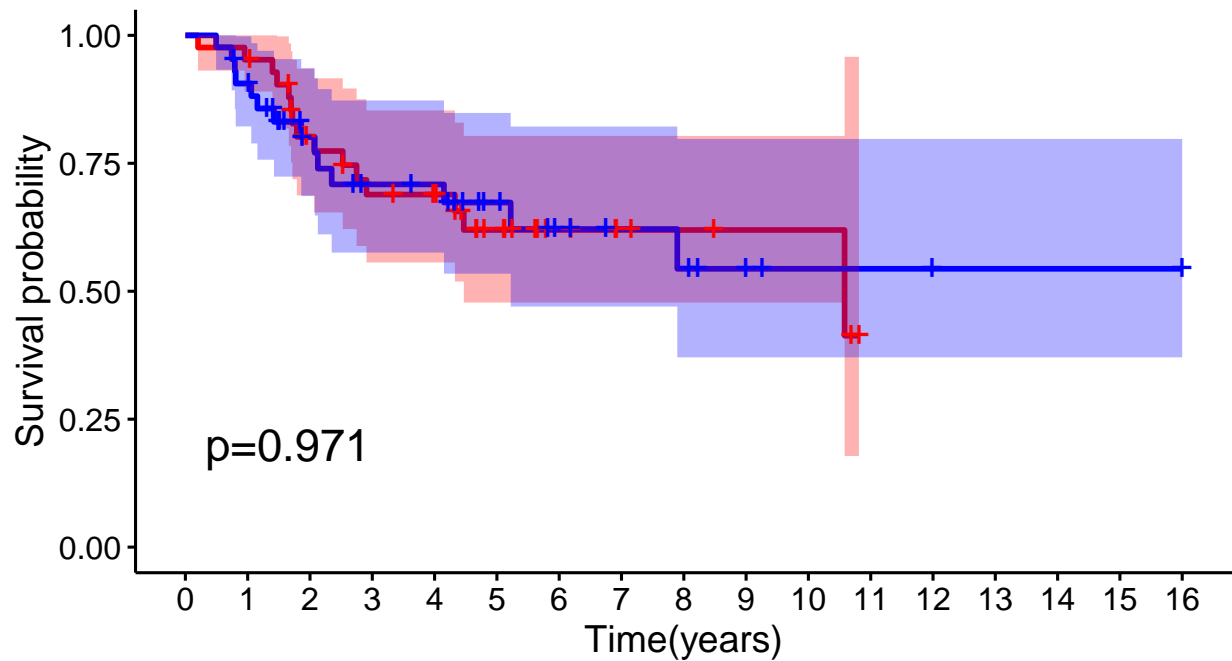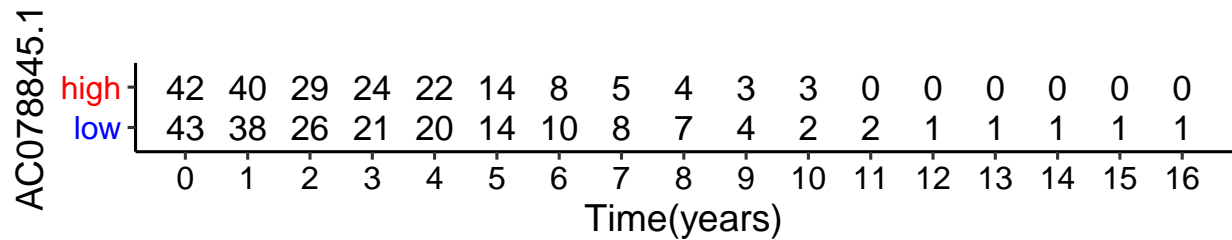

Supplement: Supplementary Document 1 — Kaplan-Meier curve of the 518 genes associated with survival. [file DataSheet_1.zip › Supplementary Document 1/sur.AC078845.1.pdf]

AC087473.1

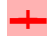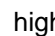

high low

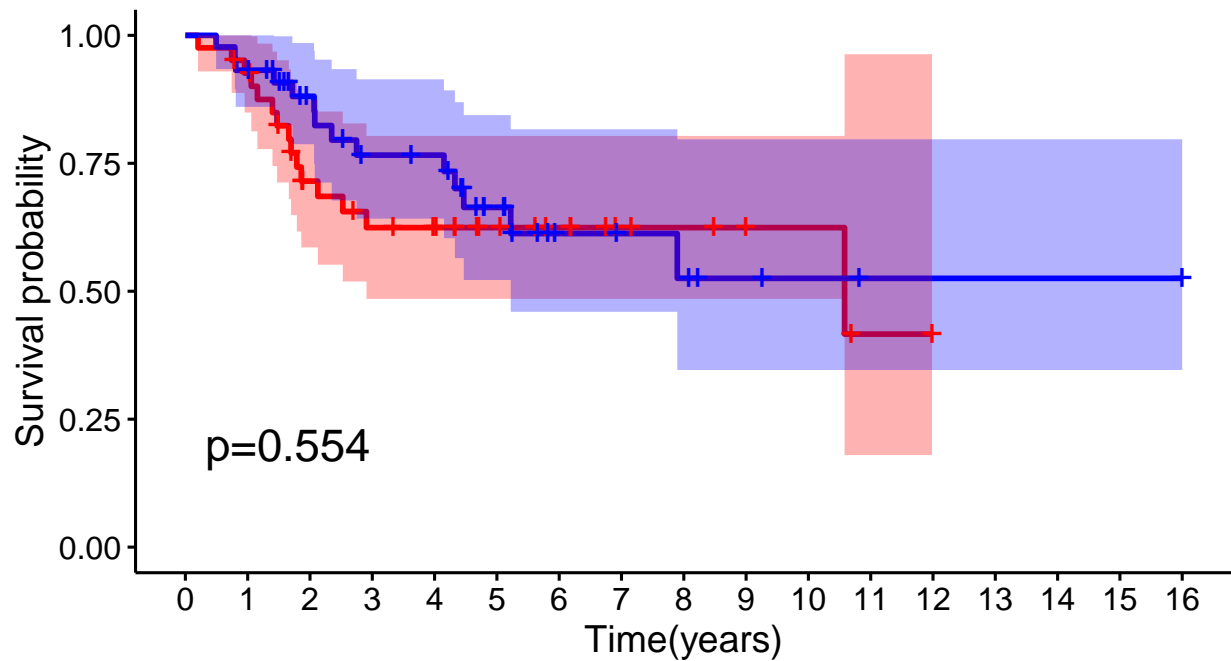

AC087473.1

high

low

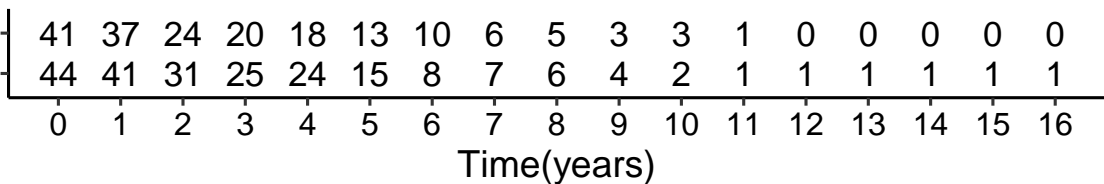

Supplement: Supplementary Document 1 — Kaplan-Meier curve of the 518 genes associated with survival. [file DataSheet_1.zip › Supplementary Document 1/sur.AC087473.1.pdf]

AC090198.1

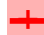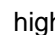

high low

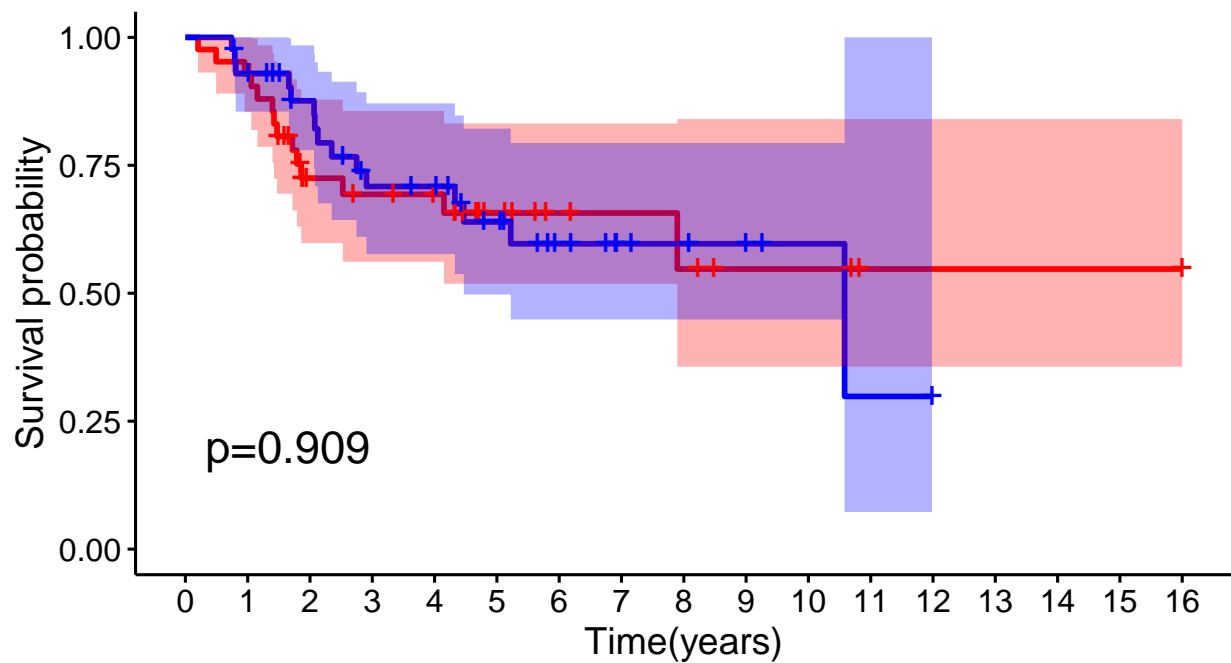

AC090198.1

high

low

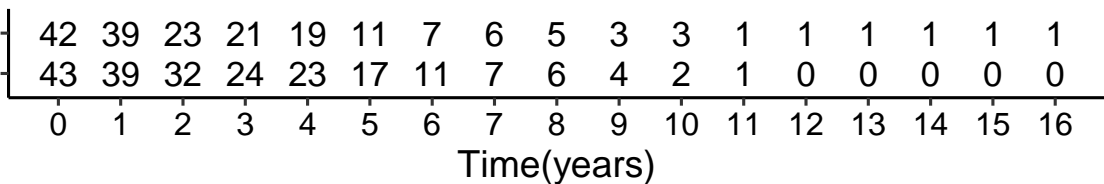

Supplement: Supplementary Document 1 — Kaplan-Meier curve of the 518 genes associated with survival. [file DataSheet_1.zip › Supplementary Document 1/sur.AC090198.1.pdf]

AC090371.1

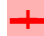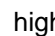

high low

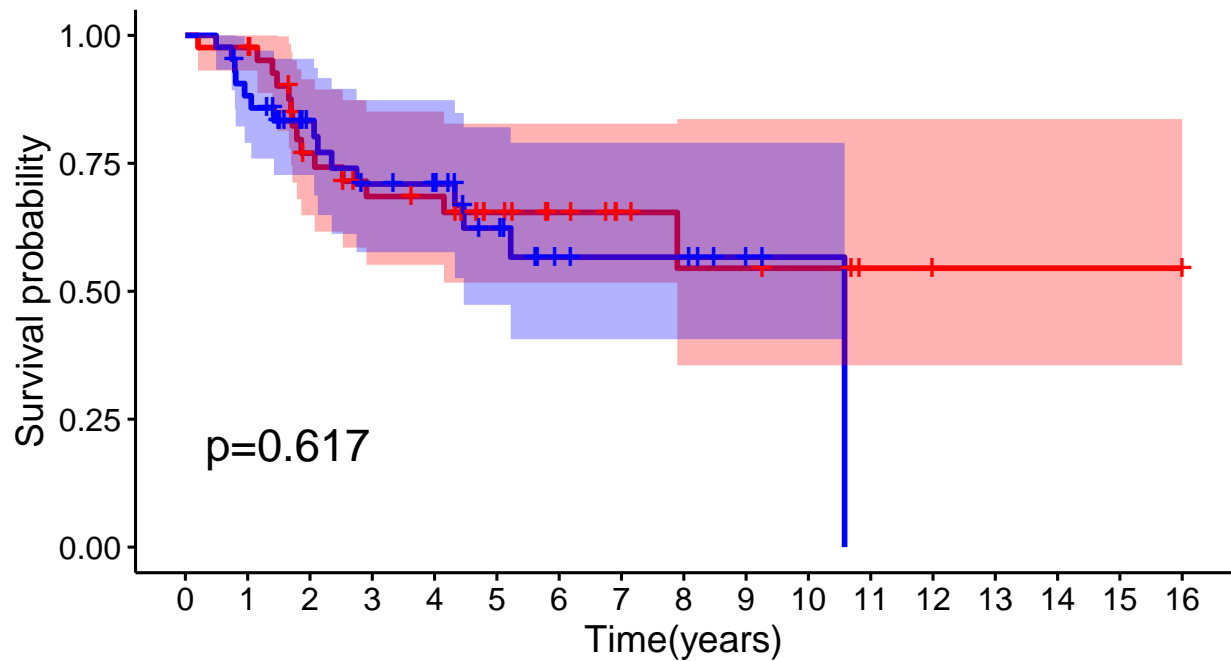

AC090371.1

high

low

|    |    |    |    |    |    |    |   |   |   |   |   |   |   |   |   |
|----|----|----|----|----|----|----|---|---|---|---|---|---|---|---|---|
| 42 | 41 | 28 | 23 | 22 | 15 | 11 | 7 | 5 | 5 | 4 | 2 | 1 | 1 | 1 | 1 |
| 43 | 37 | 27 | 22 | 20 | 13 | 7  | 6 | 6 | 2 | 1 | 0 | 0 | 0 | 0 | 0 |

Time(years)

Supplement: Supplementary Document 1 — Kaplan-Meier curve of the 518 genes associated with survival. [file DataSheet_1.zip › Supplementary Document 1/sur.AC090371.1.pdf]

AC090922.1 high low

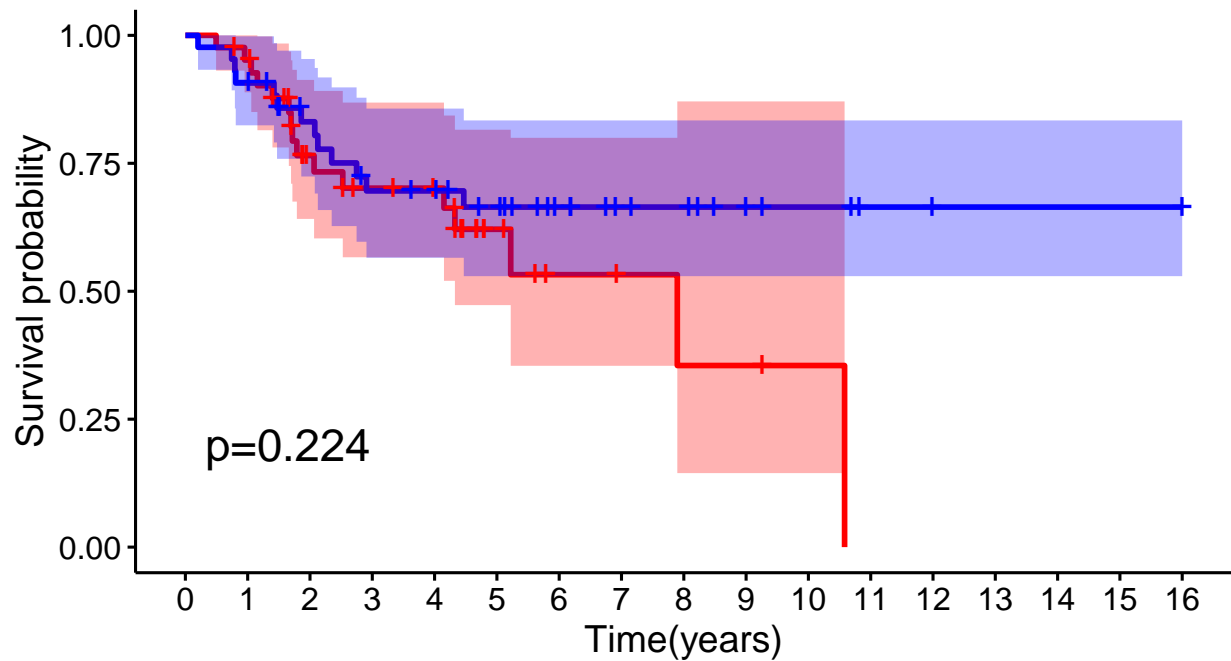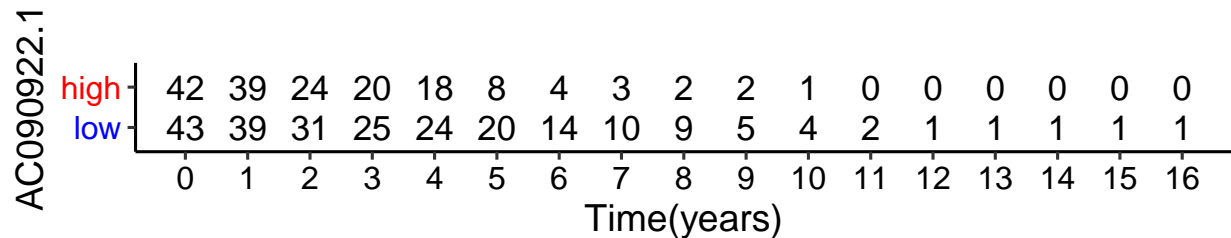

Supplement: Supplementary Document 1 — Kaplan-Meier curve of the 518 genes associated with survival. [file DataSheet_1.zip › Supplementary Document 1/sur.AC090922.1.pdf]

AC091435.2

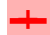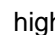

high low

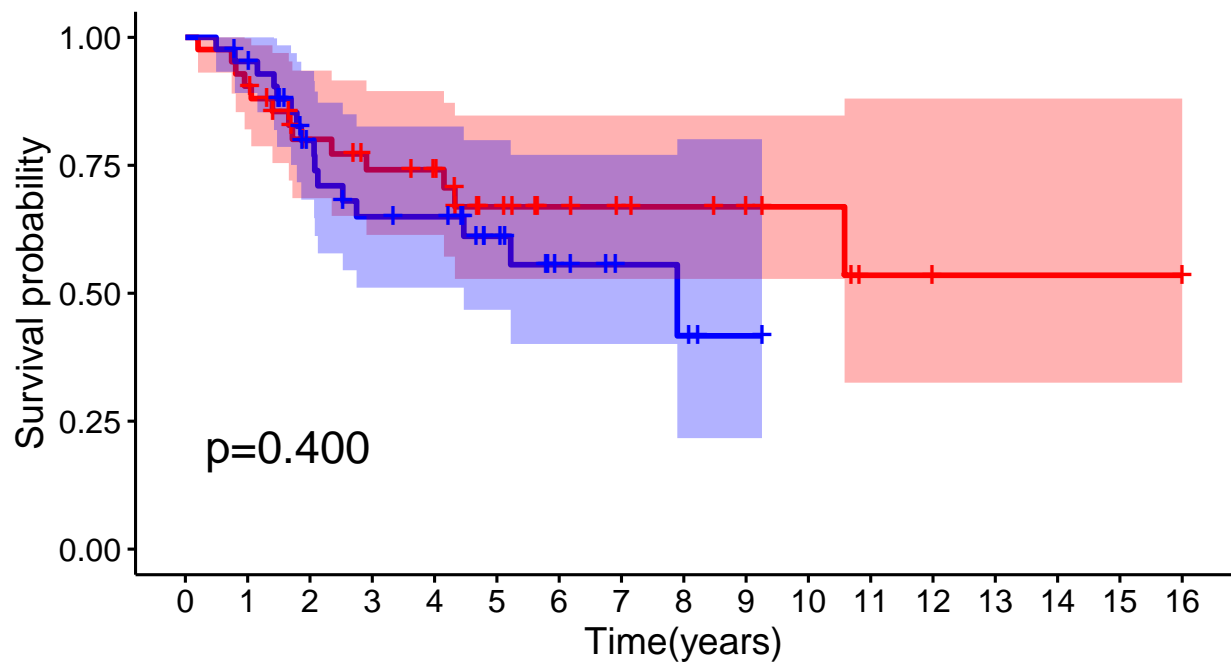

AC091435.2

high

low

|    |    |    |    |    |    |    |   |   |   |   |   |   |   |   |   |
|----|----|----|----|----|----|----|---|---|---|---|---|---|---|---|---|
| 42 | 38 | 28 | 24 | 22 | 15 | 11 | 9 | 8 | 6 | 5 | 2 | 1 | 1 | 1 | 1 |
| 43 | 40 | 27 | 21 | 20 | 13 | 7  | 4 | 3 | 1 | 0 | 0 | 0 | 0 | 0 | 0 |

Time(years)

Supplement: Supplementary Document 1 — Kaplan-Meier curve of the 518 genes associated with survival. [file DataSheet_1.zip › Supplementary Document 1/sur.AC091435.2.pdf]

AC092118.1 + high + low

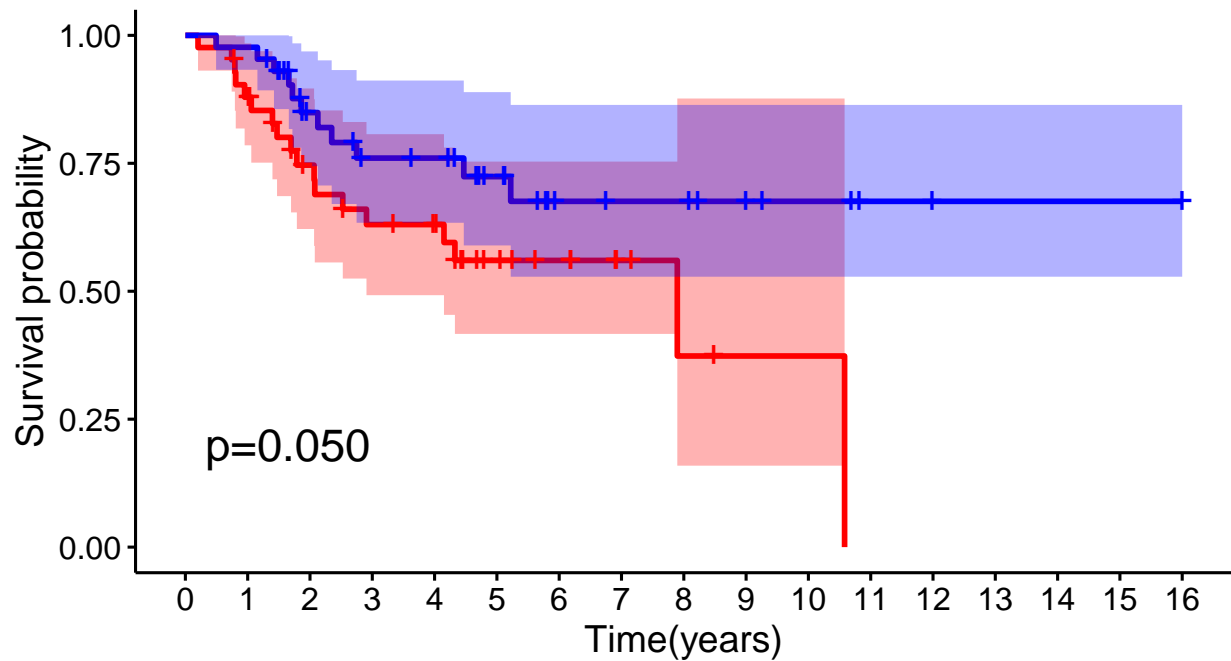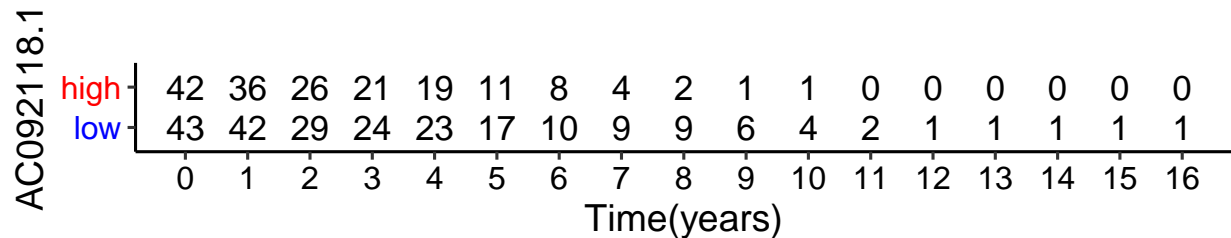

Supplement: Supplementary Document 1 — Kaplan-Meier curve of the 518 genes associated with survival. [file DataSheet_1.zip › Supplementary Document 1/sur.AC092118.1.pdf]

AC092325.1 + high + low

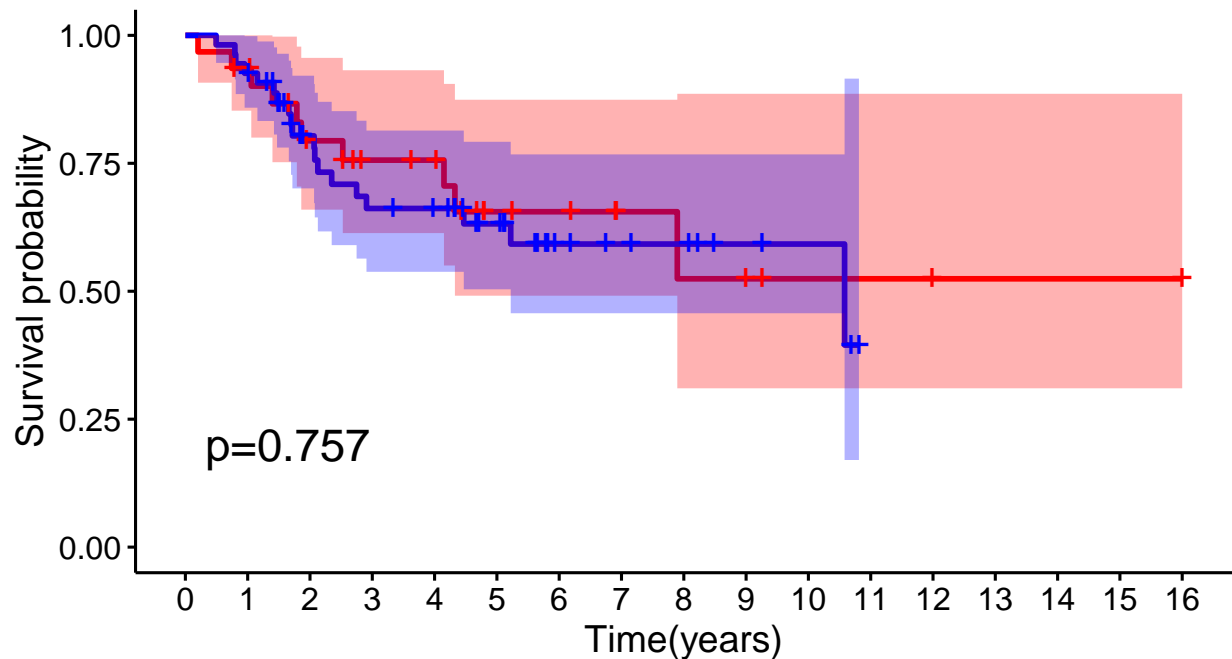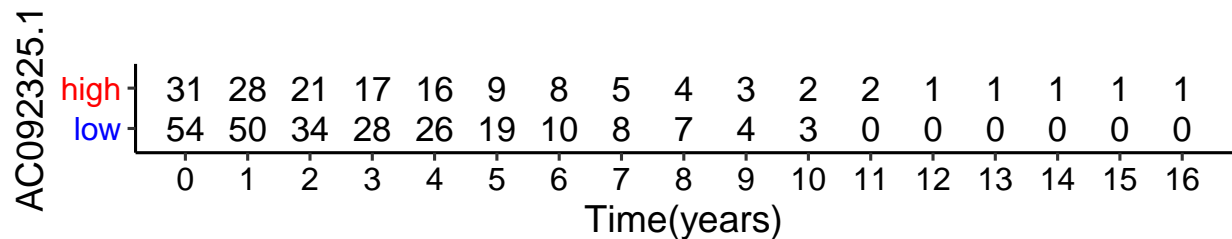

Supplement: Supplementary Document 1 — Kaplan-Meier curve of the 518 genes associated with survival. [file DataSheet_1.zip › Supplementary Document 1/sur.AC092325.1.pdf]

AC092436.2

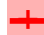

high

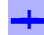

low

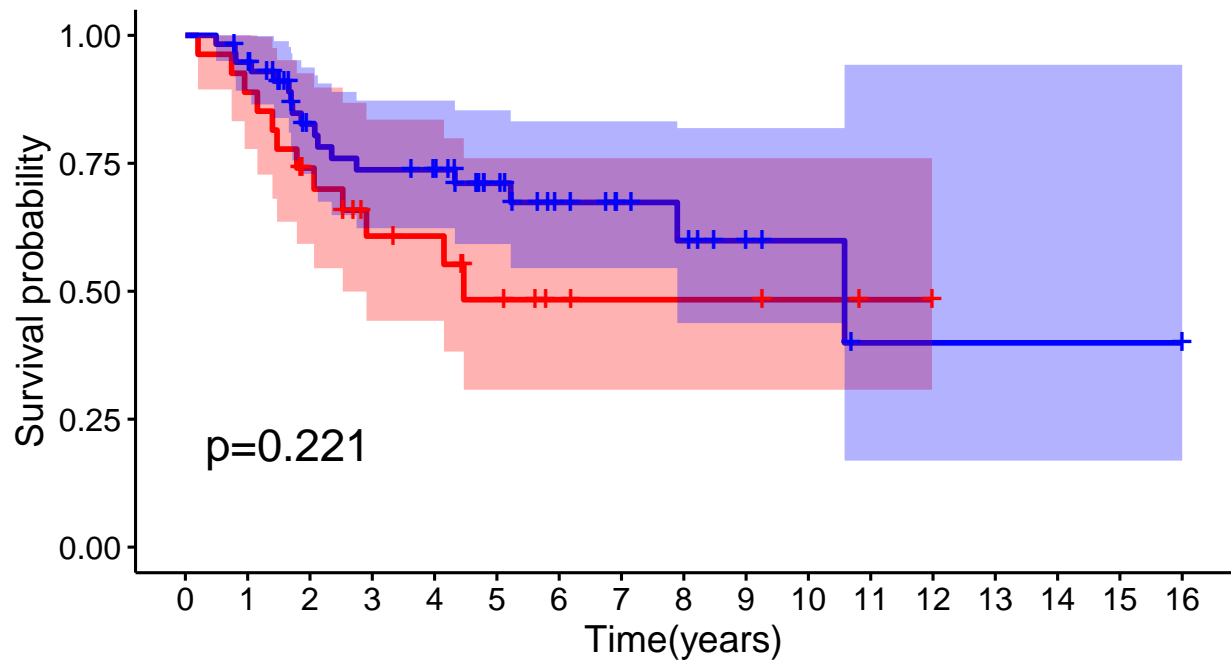

AC092436.2

high

low

|    |    |    |    |    |    |    |    |   |   |   |   |   |   |   |   |
|----|----|----|----|----|----|----|----|---|---|---|---|---|---|---|---|
| 27 | 24 | 18 | 12 | 11 | 7  | 4  | 3  | 3 | 3 | 2 | 1 | 0 | 0 | 0 | 0 |
| 58 | 54 | 37 | 33 | 31 | 21 | 14 | 10 | 8 | 4 | 3 | 1 | 1 | 1 | 1 | 1 |

Time(years)

Supplement: Supplementary Document 1 — Kaplan-Meier curve of the 518 genes associated with survival. [file DataSheet_1.zip › Supplementary Document 1/sur.AC092436.2.pdf]

AC092634.5

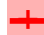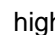

high low

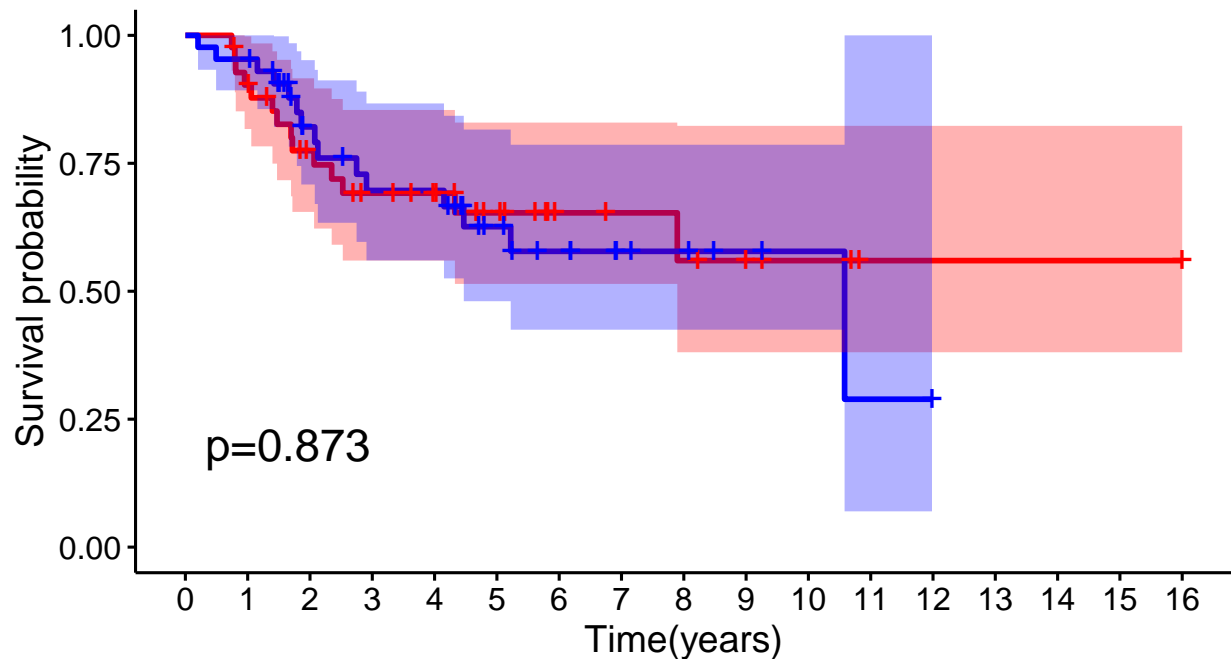

AC092634.5

high

low

|    |    |    |    |    |    |    |   |   |   |    |    |    |    |    |    |
|----|----|----|----|----|----|----|---|---|---|----|----|----|----|----|----|
| 42 | 37 | 28 | 23 | 20 | 14 | 8  | 7 | 6 | 4 | 3  | 1  | 1  | 1  | 1  | 1  |
| 43 | 41 | 27 | 22 | 22 | 14 | 10 | 6 | 5 | 3 | 2  | 1  | 0  | 0  | 0  | 0  |
| 0  | 1  | 2  | 3  | 4  | 5  | 6  | 7 | 8 | 9 | 10 | 11 | 12 | 13 | 14 | 15 |

Time(years)

Supplement: Supplementary Document 1 — Kaplan-Meier curve of the 518 genes associated with survival. [file DataSheet_1.zip › Supplementary Document 1/sur.AC092634.5.pdf]

AC093388.1 + high + low

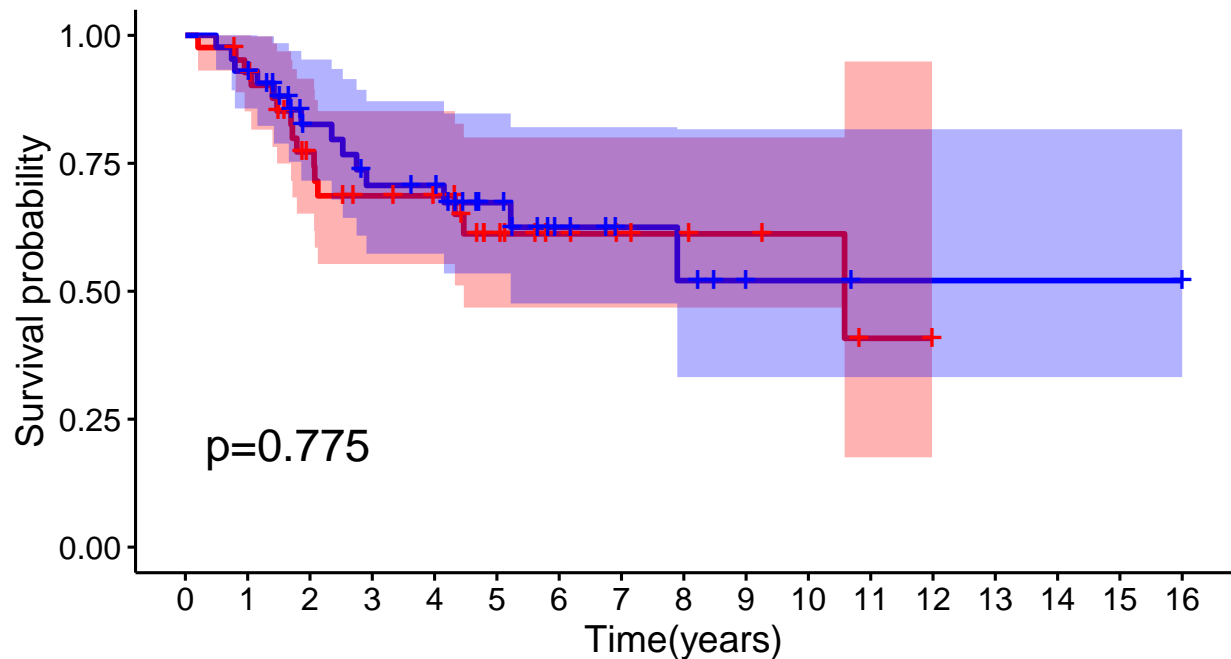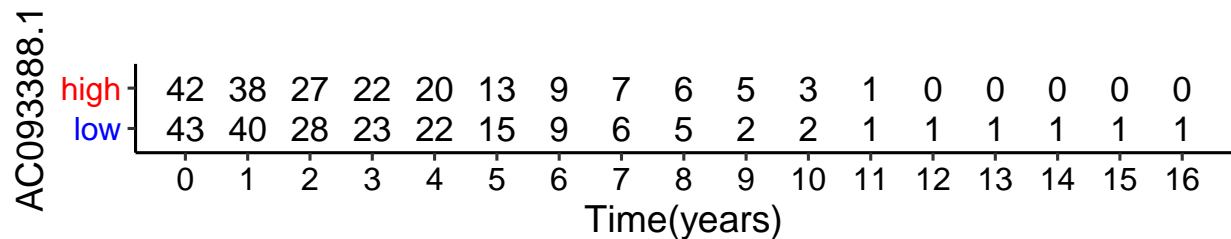

Supplement: Supplementary Document 1 — Kaplan-Meier curve of the 518 genes associated with survival. [file DataSheet_1.zip › Supplementary Document 1/sur.AC093388.1.pdf]

AC093730.1 high low

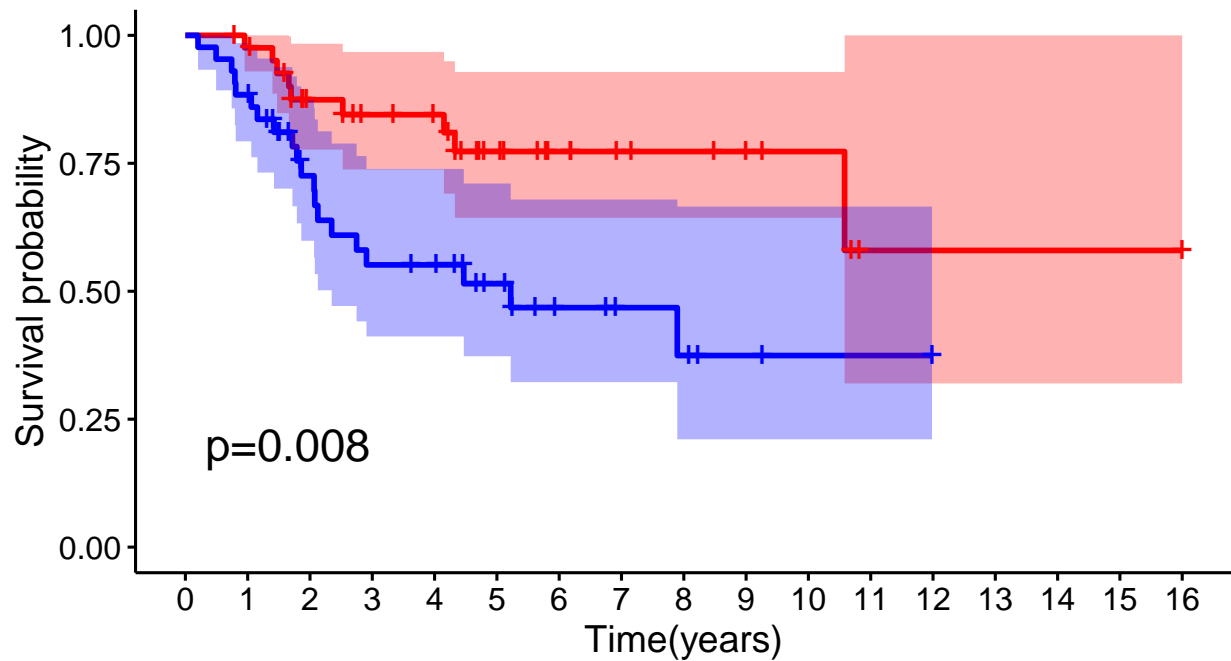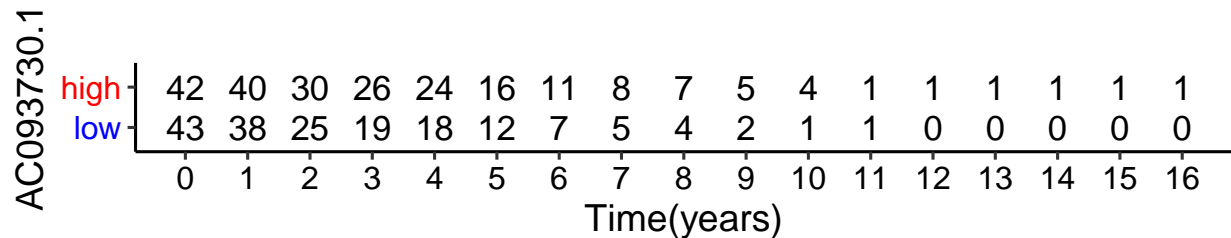

Supplement: Supplementary Document 1 — Kaplan-Meier curve of the 518 genes associated with survival. [file DataSheet_1.zip › Supplementary Document 1/sur.AC093730.1.pdf]

AC093895.1 + high + low

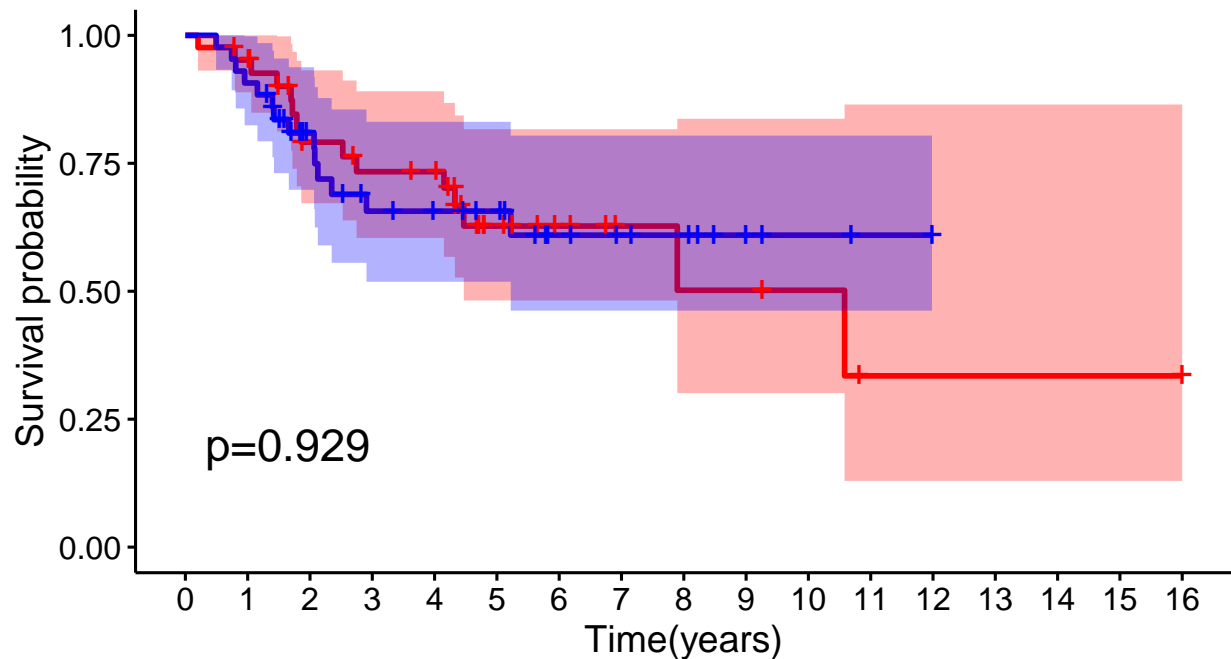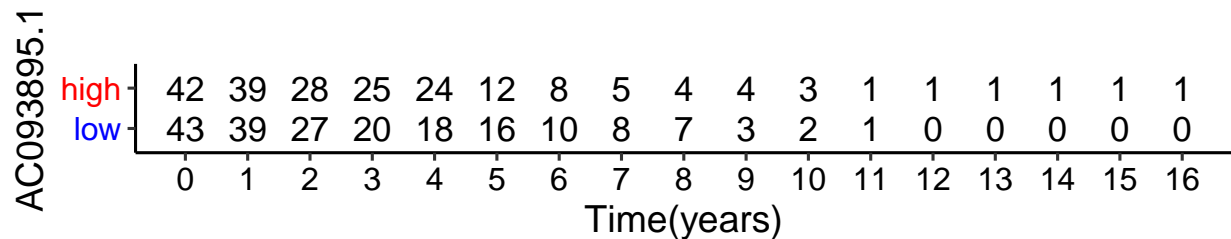

Supplement: Supplementary Document 1 — Kaplan-Meier curve of the 518 genes associated with survival. [file DataSheet_1.zip › Supplementary Document 1/sur.AC093895.1.pdf]

AC096564.1 high low

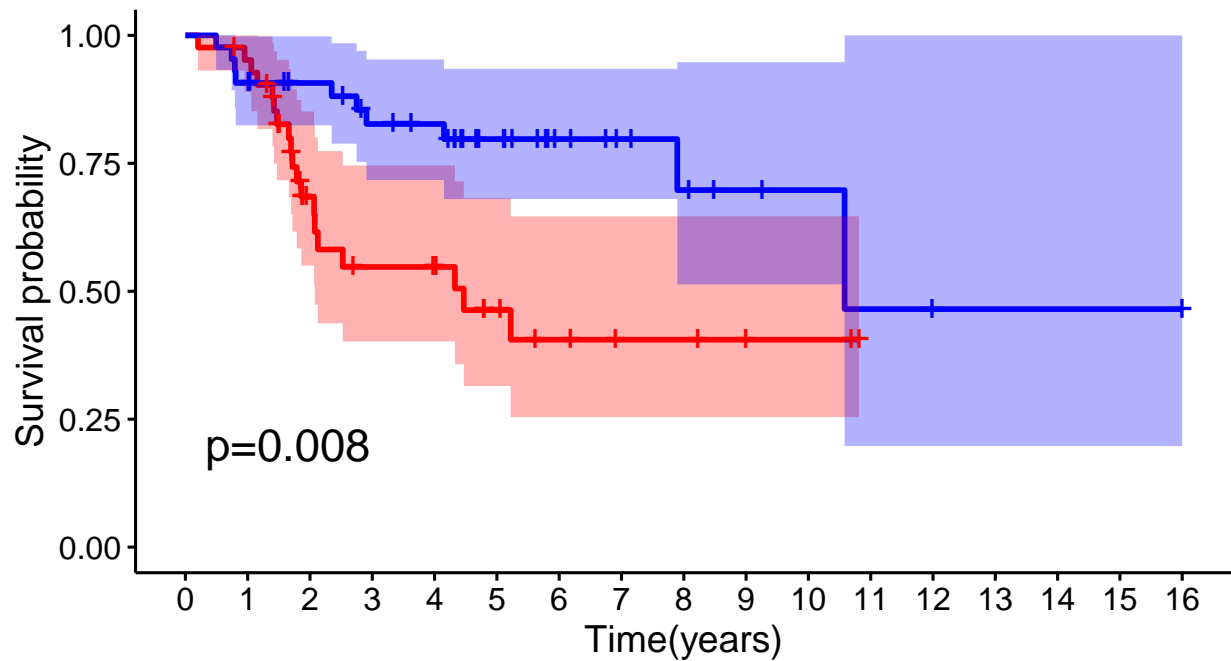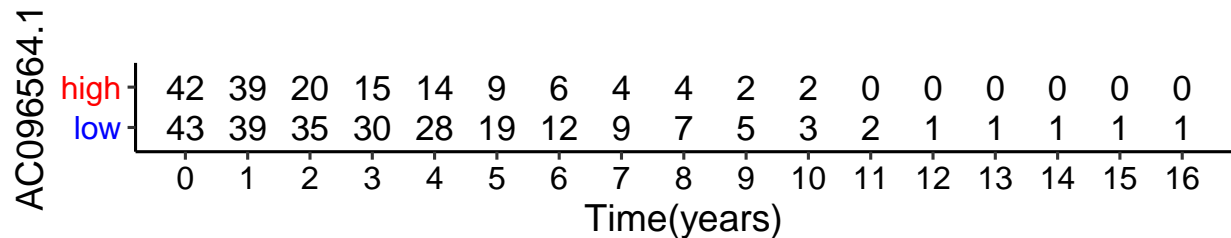

Supplement: Supplementary Document 1 — Kaplan-Meier curve of the 518 genes associated with survival. [file DataSheet_1.zip › Supplementary Document 1/sur.AC096564.1.pdf]

AC097662.1 + high + low

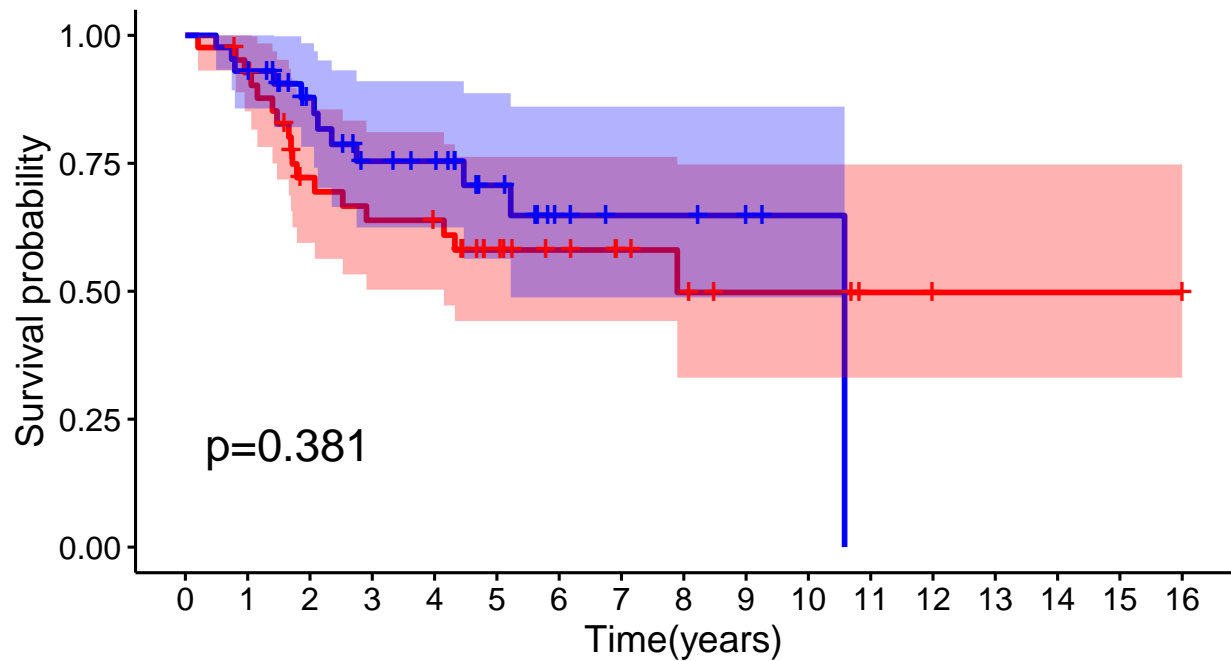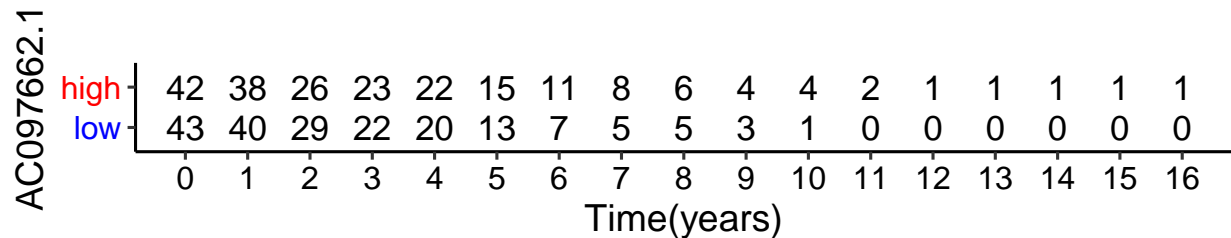

Supplement: Supplementary Document 1 — Kaplan-Meier curve of the 518 genes associated with survival. [file DataSheet_1.zip › Supplementary Document 1/sur.AC097662.1.pdf]

AC099521.1 + high + low

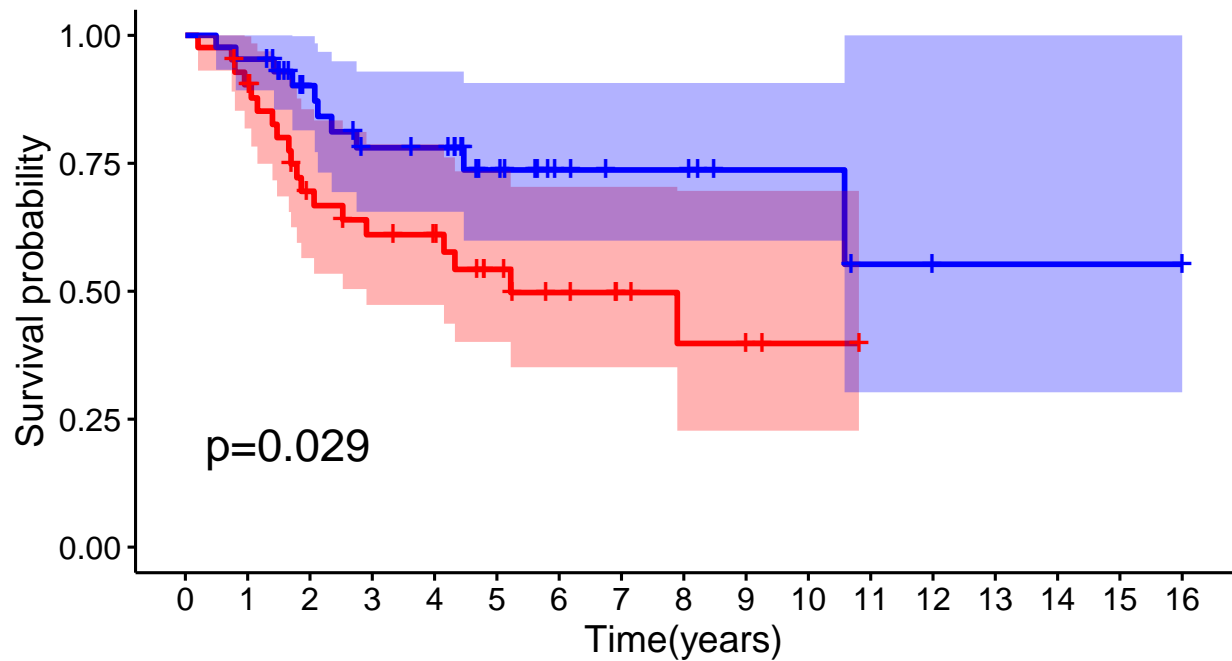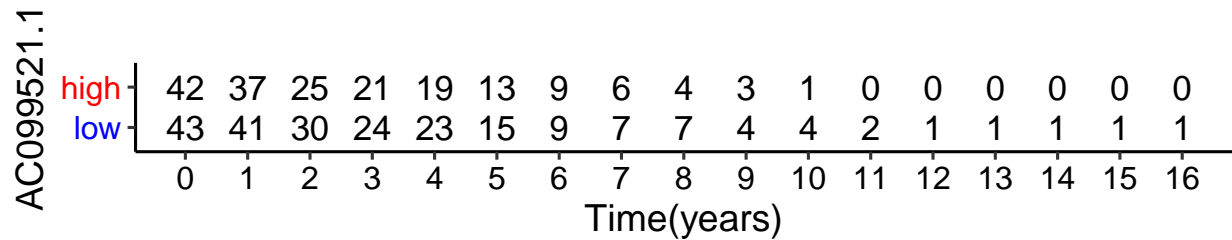

Supplement: Supplementary Document 1 — Kaplan-Meier curve of the 518 genes associated with survival. [file DataSheet_1.zip › Supplementary Document 1/sur.AC099521.1.pdf]

AC100800.1 + high + low

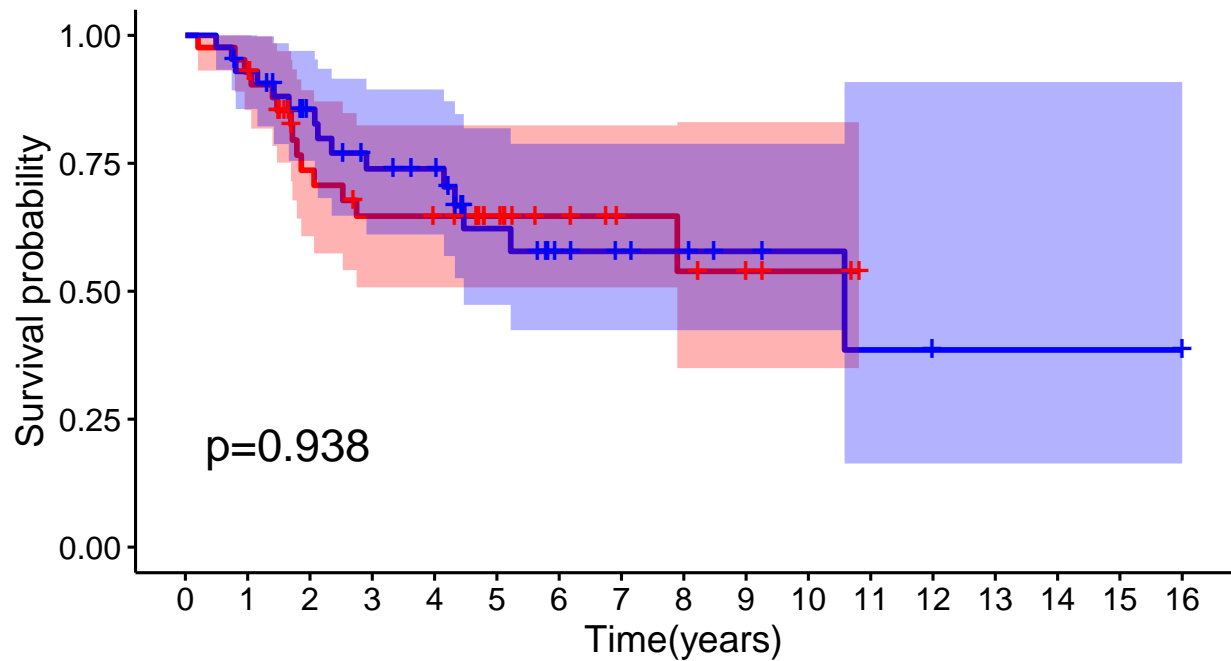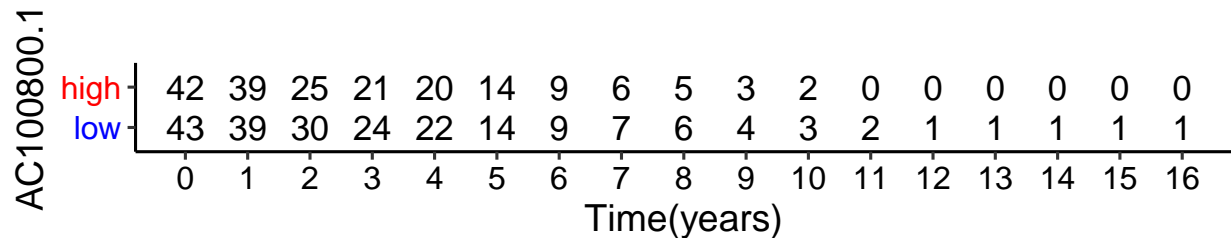

Supplement: Supplementary Document 1 — Kaplan-Meier curve of the 518 genes associated with survival. [file DataSheet_1.zip › Supplementary Document 1/sur.AC100800.1.pdf]

AC104435.2

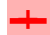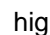

high low

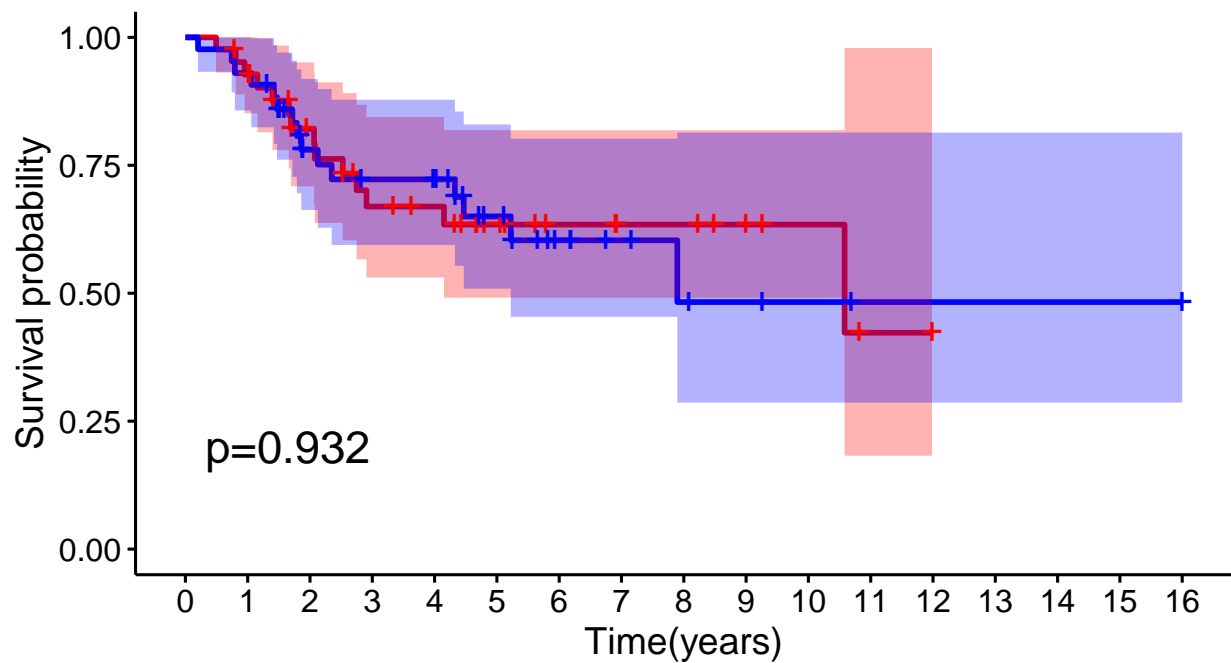

AC104435.2

high

low

|    |    |    |    |    |    |   |   |   |   |    |    |    |    |    |    |
|----|----|----|----|----|----|---|---|---|---|----|----|----|----|----|----|
| 42 | 38 | 28 | 21 | 19 | 13 | 9 | 7 | 7 | 4 | 3  | 1  | 0  | 0  | 0  | 0  |
| 43 | 40 | 27 | 24 | 23 | 15 | 9 | 6 | 4 | 3 | 2  | 1  | 1  | 1  | 1  | 1  |
| 0  | 1  | 2  | 3  | 4  | 5  | 6 | 7 | 8 | 9 | 10 | 11 | 12 | 13 | 14 | 15 |

Time(years)

Supplement: Supplementary Document 1 — Kaplan-Meier curve of the 518 genes associated with survival. [file DataSheet_1.zip › Supplementary Document 1/sur.AC104435.2.pdf]

low

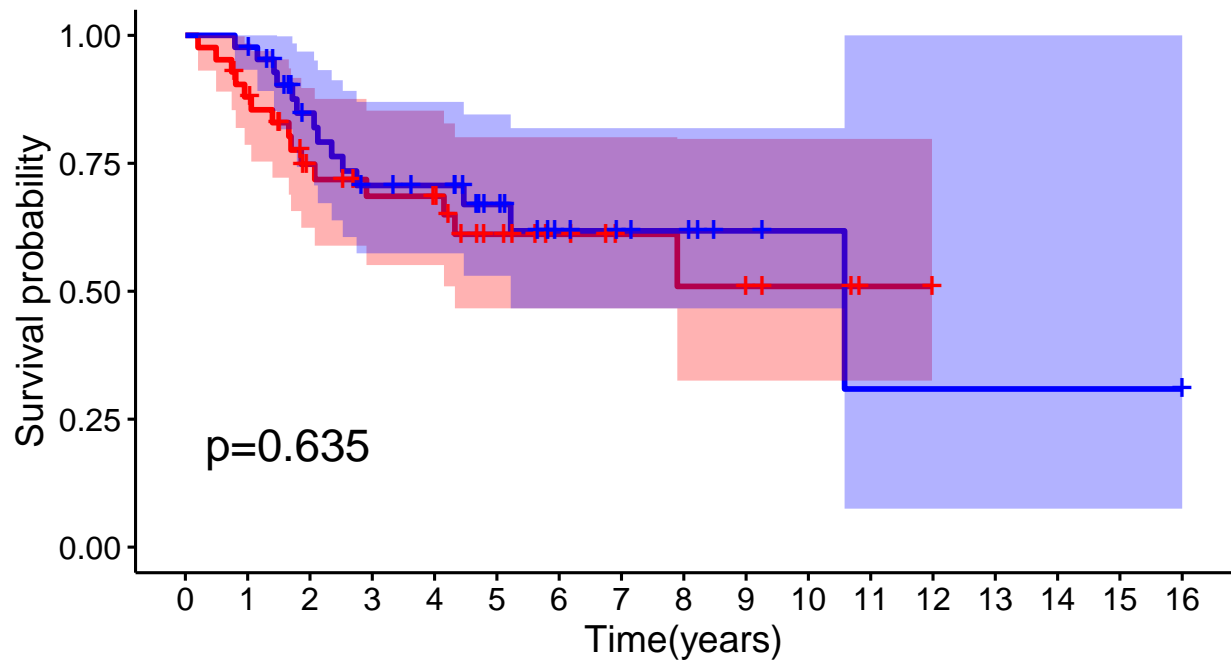

AC105254.1

high

low

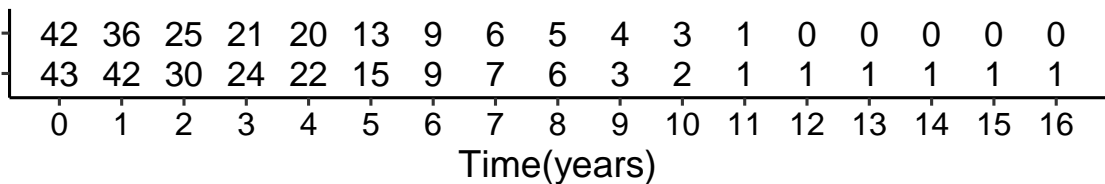

Supplement: Supplementary Document 1 — Kaplan-Meier curve of the 518 genes associated with survival. [file DataSheet_1.zip › Supplementary Document 1/sur.AC105254.1.pdf]

AC105446.1 + high + low

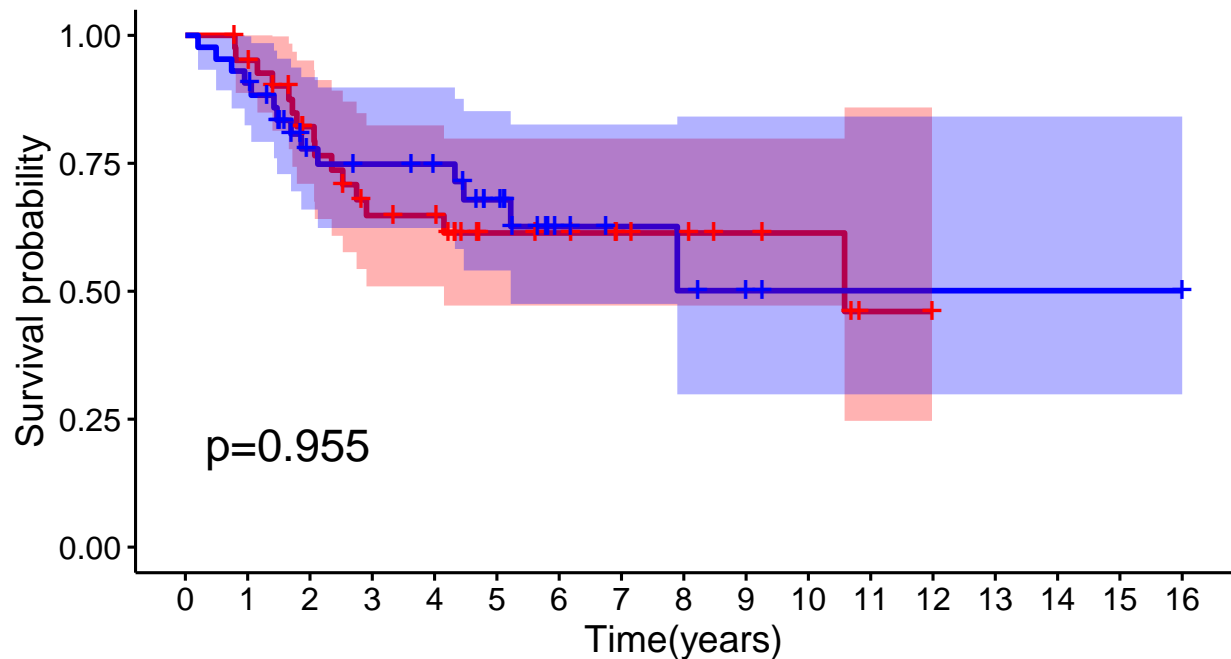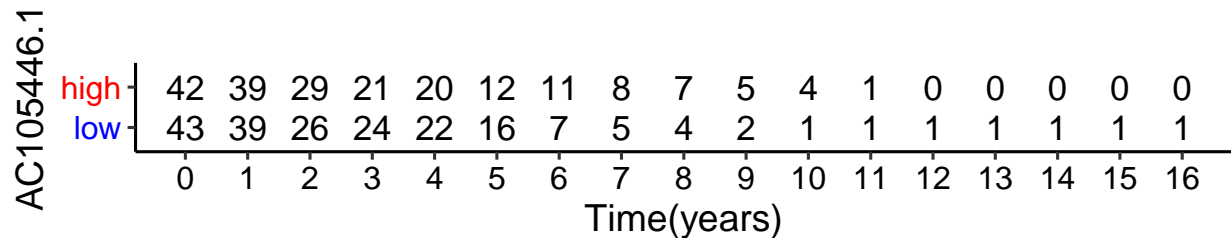

Supplement: Supplementary Document 1 — Kaplan-Meier curve of the 518 genes associated with survival. [file DataSheet_1.zip › Supplementary Document 1/sur.AC105446.1.pdf]

W

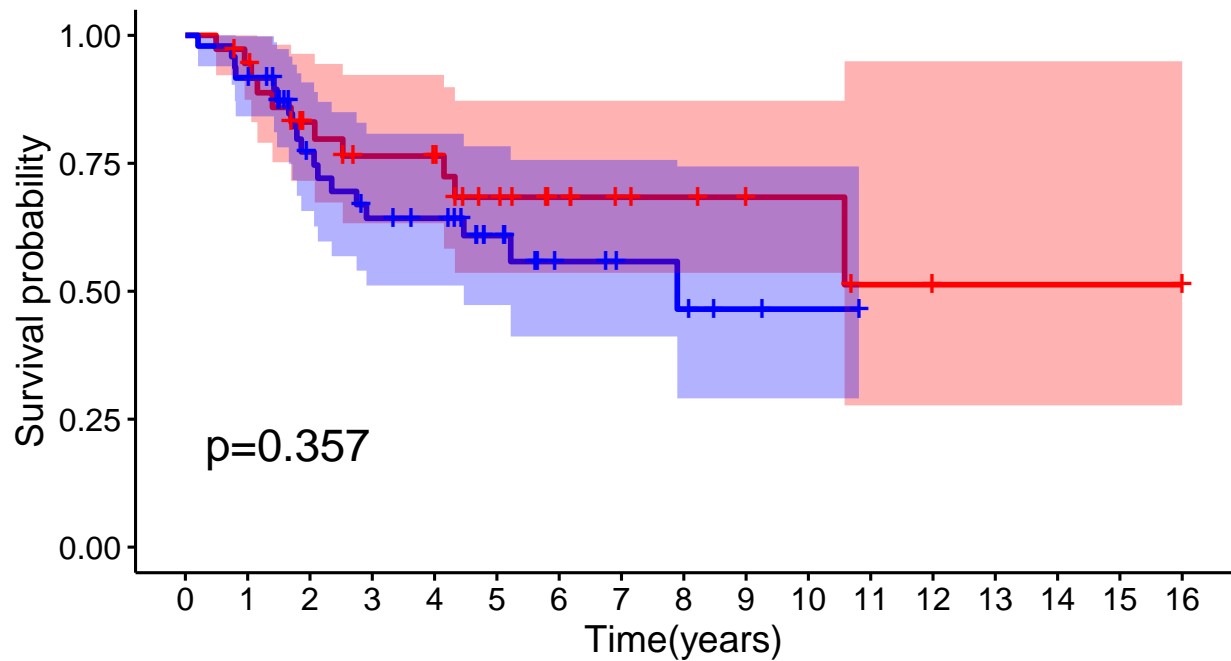

AC109439.2

high

low

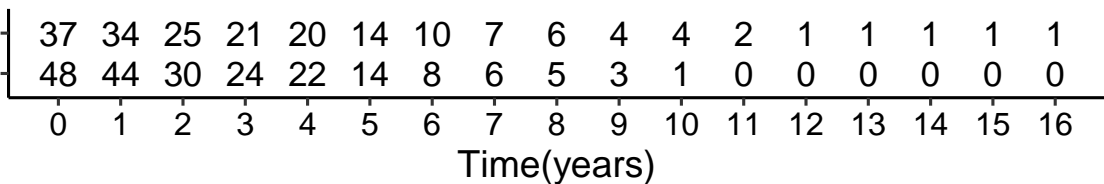

Supplement: Supplementary Document 1 — Kaplan-Meier curve of the 518 genes associated with survival. [file DataSheet_1.zip › Supplementary Document 1/sur.AC109439.2.pdf]

AC114928.1 high low

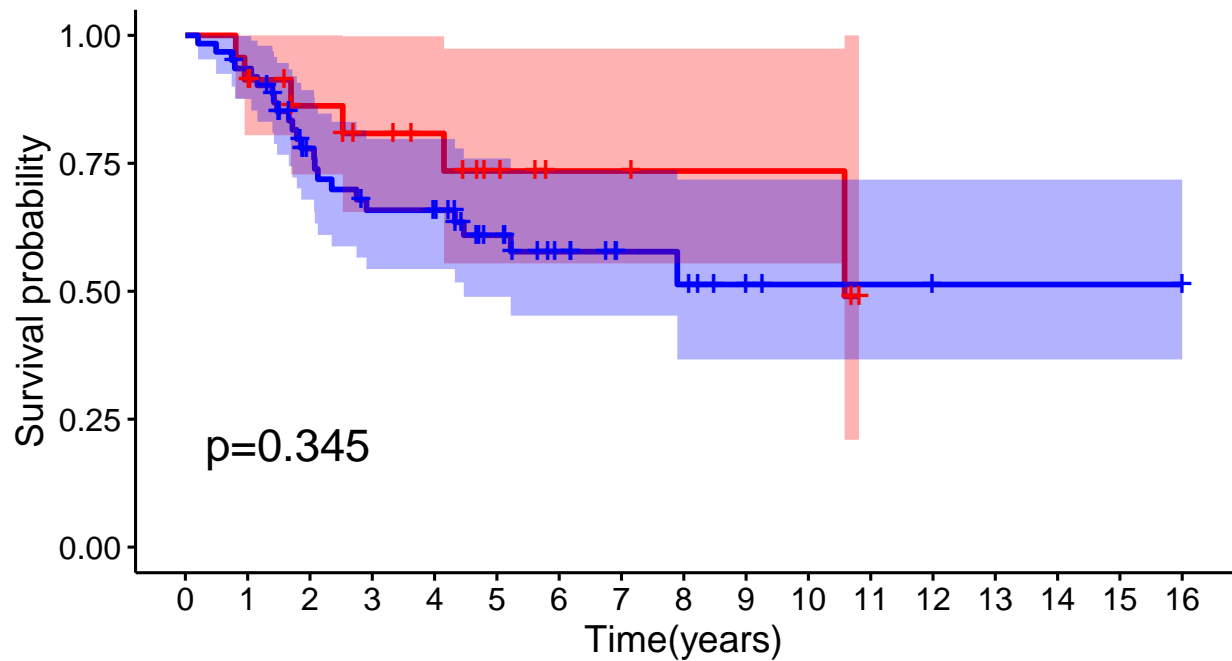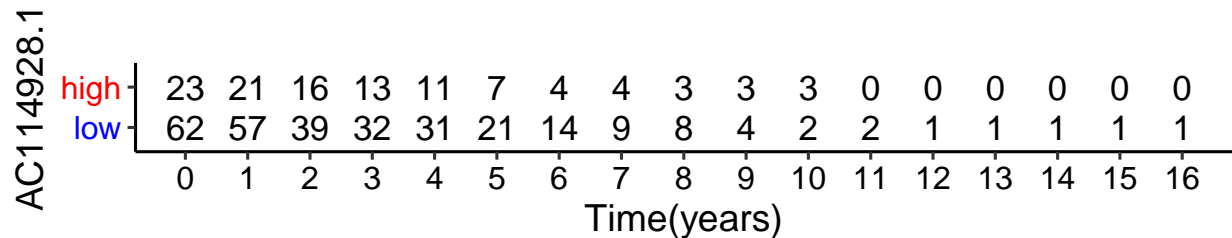

Supplement: Supplementary Document 1 — Kaplan-Meier curve of the 518 genes associated with survival. [file DataSheet_1.zip › Supplementary Document 1/sur.AC114928.1.pdf]

AC116366.2 + high + low

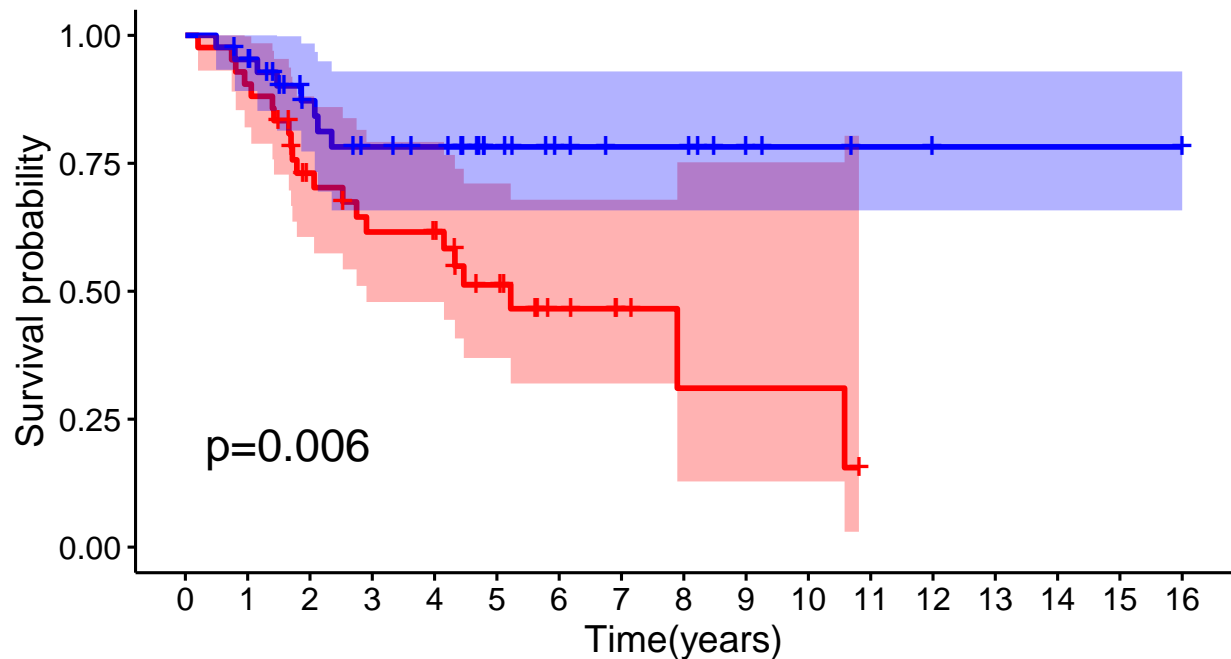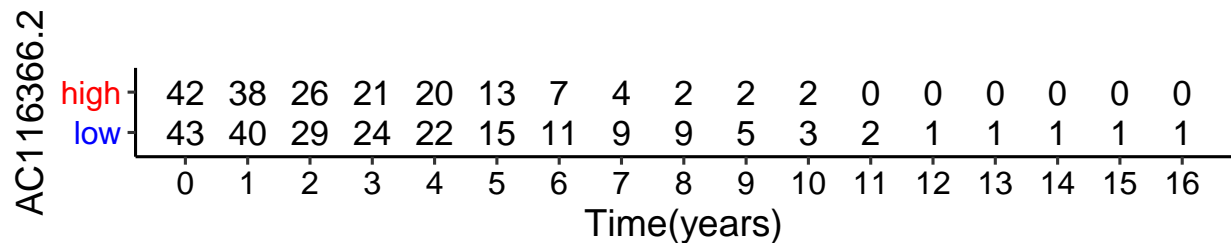

Supplement: Supplementary Document 1 — Kaplan-Meier curve of the 518 genes associated with survival. [file DataSheet_1.zip › Supplementary Document 1/sur.AC116366.2.pdf]

AC116914.2 + high + low

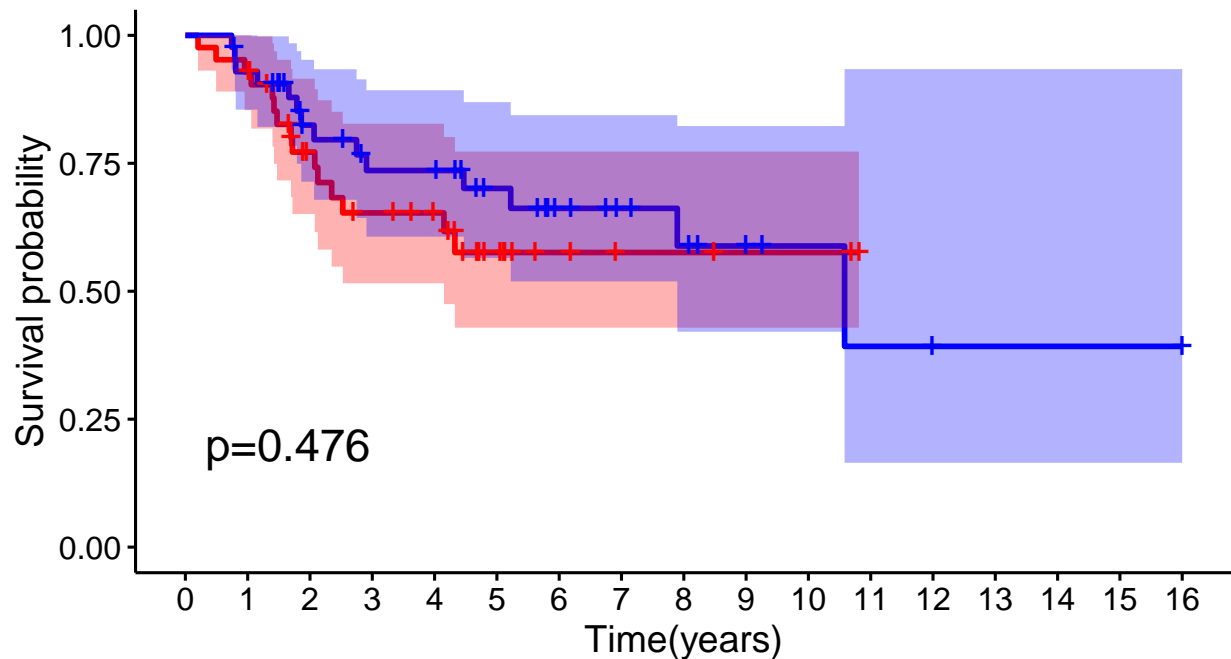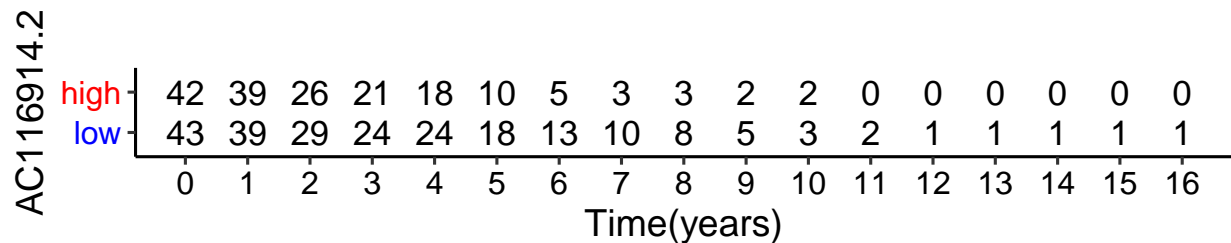

Supplement: Supplementary Document 1 — Kaplan-Meier curve of the 518 genes associated with survival. [file DataSheet_1.zip › Supplementary Document 1/sur.AC116914.2.pdf]

AC121338.2

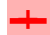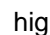

high low

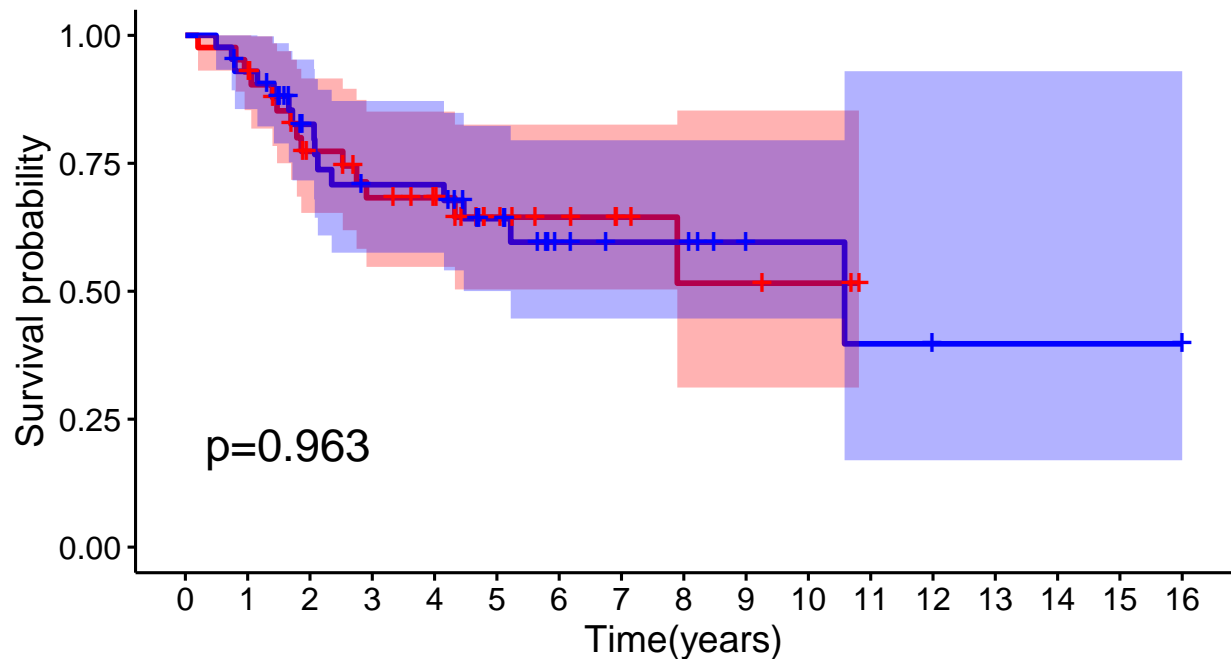

AC121338.2

high

low

|    |    |    |    |    |    |   |   |   |   |    |    |    |    |    |    |
|----|----|----|----|----|----|---|---|---|---|----|----|----|----|----|----|
| 42 | 39 | 27 | 22 | 19 | 12 | 9 | 6 | 4 | 4 | 2  | 0  | 0  | 0  | 0  | 0  |
| 43 | 39 | 28 | 23 | 23 | 16 | 9 | 7 | 7 | 3 | 3  | 2  | 1  | 1  | 1  | 1  |
| 0  | 1  | 2  | 3  | 4  | 5  | 6 | 7 | 8 | 9 | 10 | 11 | 12 | 13 | 14 | 15 |

Time(years)

Supplement: Supplementary Document 1 — Kaplan-Meier curve of the 518 genes associated with survival. [file DataSheet_1.zip › Supplementary Document 1/sur.AC121338.2.pdf]

AC125618.1 + high + low

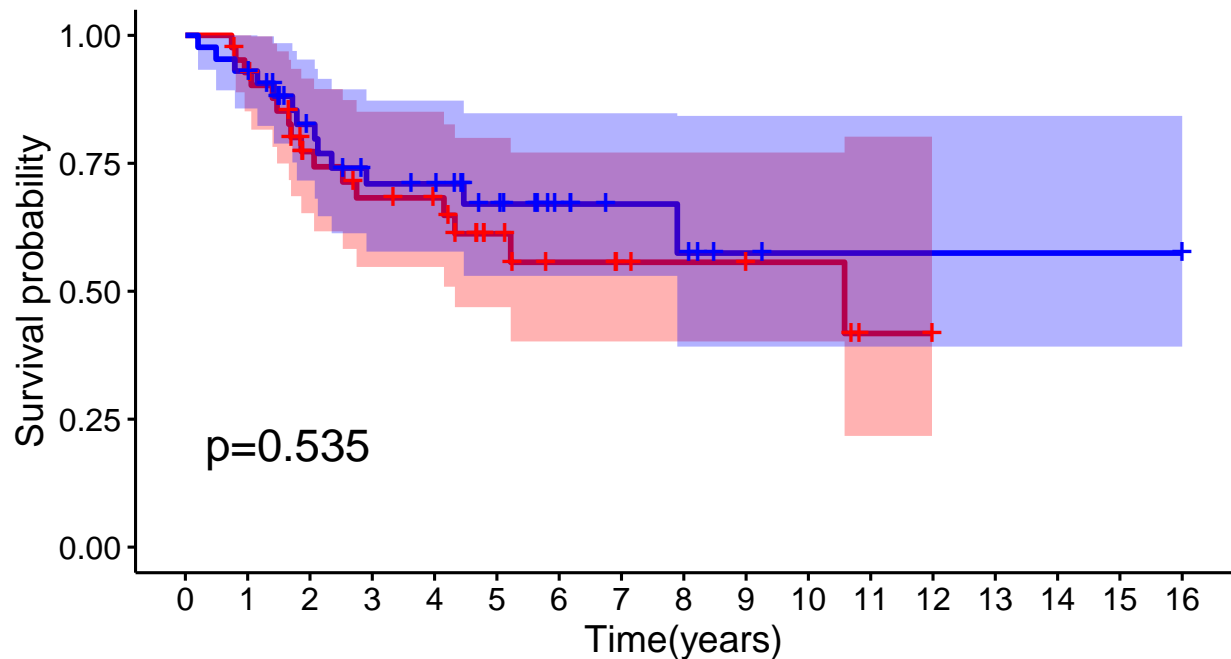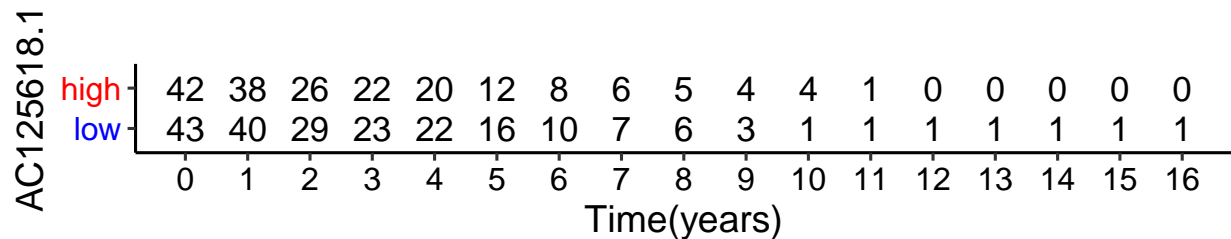

Supplement: Supplementary Document 1 — Kaplan-Meier curve of the 518 genes associated with survival. [file DataSheet_1.zip › Supplementary Document 1/sur.AC125618.1.pdf]

AC126614.1 + high + low

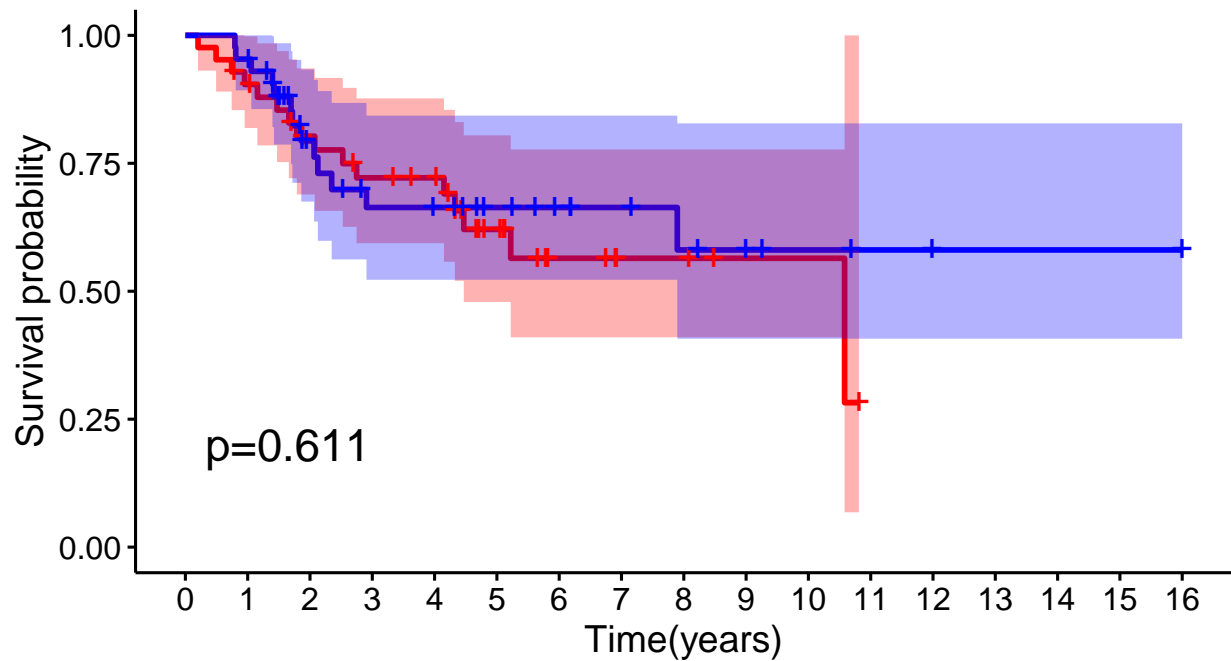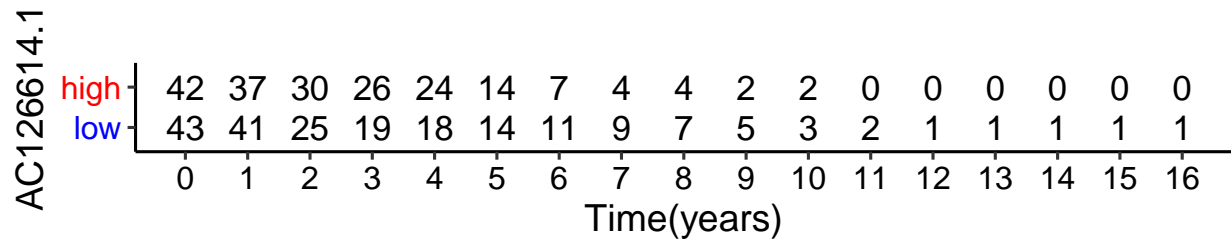

Supplement: Supplementary Document 1 — Kaplan-Meier curve of the 518 genes associated with survival. [file DataSheet_1.zip › Supplementary Document 1/sur.AC126614.1.pdf]

AC133106.1 high low

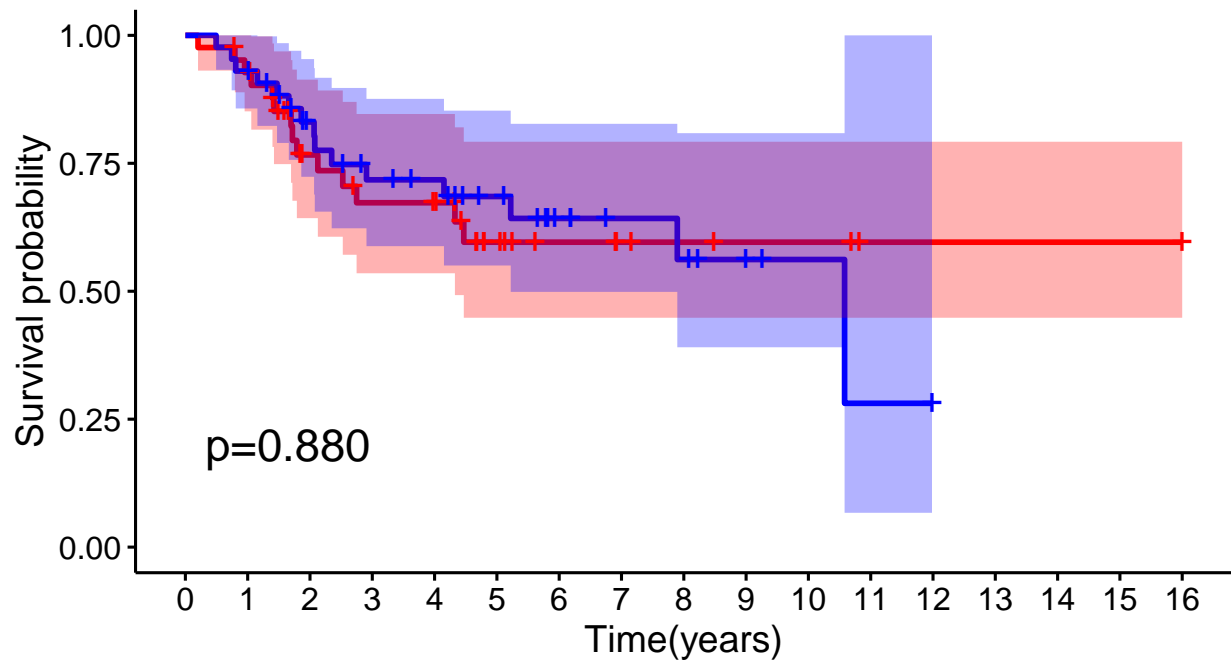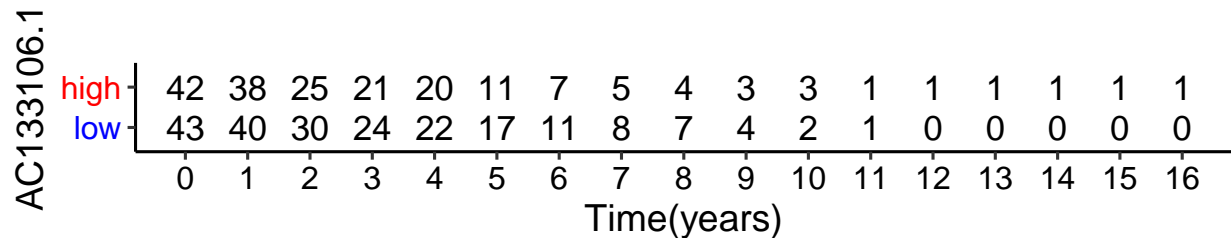

Supplement: Supplementary Document 1 — Kaplan-Meier curve of the 518 genes associated with survival. [file DataSheet_1.zip › Supplementary Document 1/sur.AC133106.1.pdf]

AC135012.3

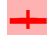

high

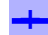

low

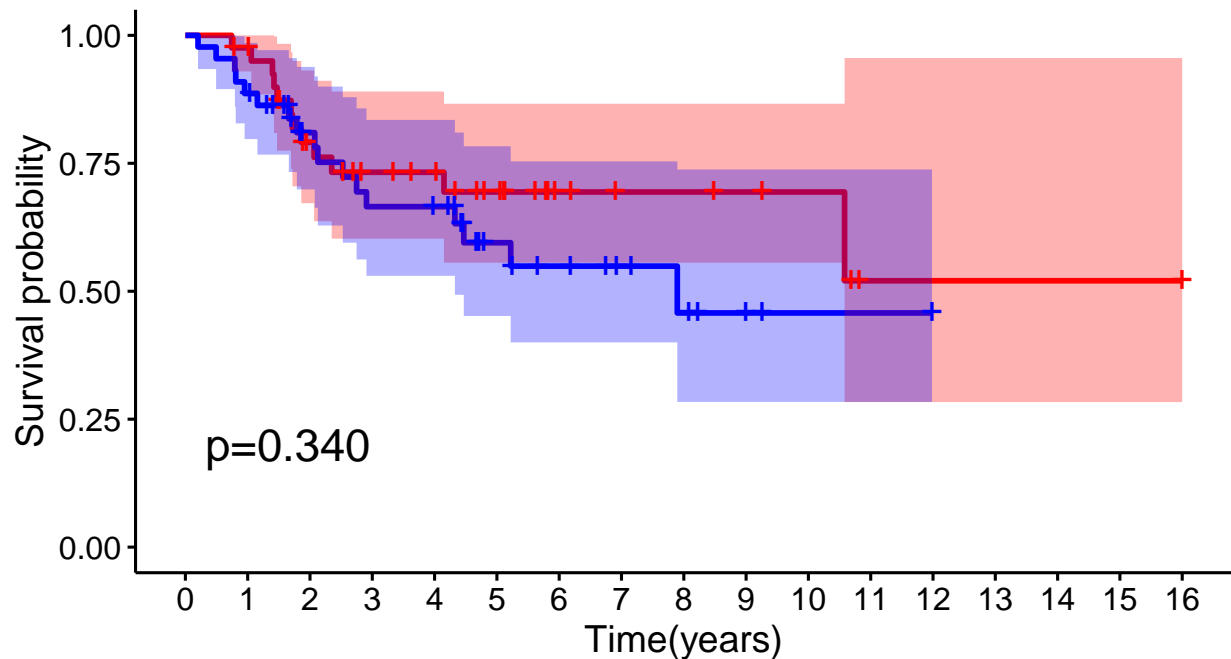

AC135012.3

high

low

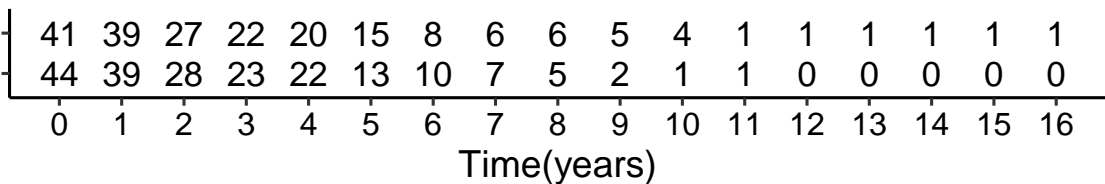

Supplement: Supplementary Document 1 — Kaplan-Meier curve of the 518 genes associated with survival. [file DataSheet_1.zip › Supplementary Document 1/sur.AC135012.3.pdf]

AC137834.2

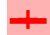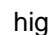

high low

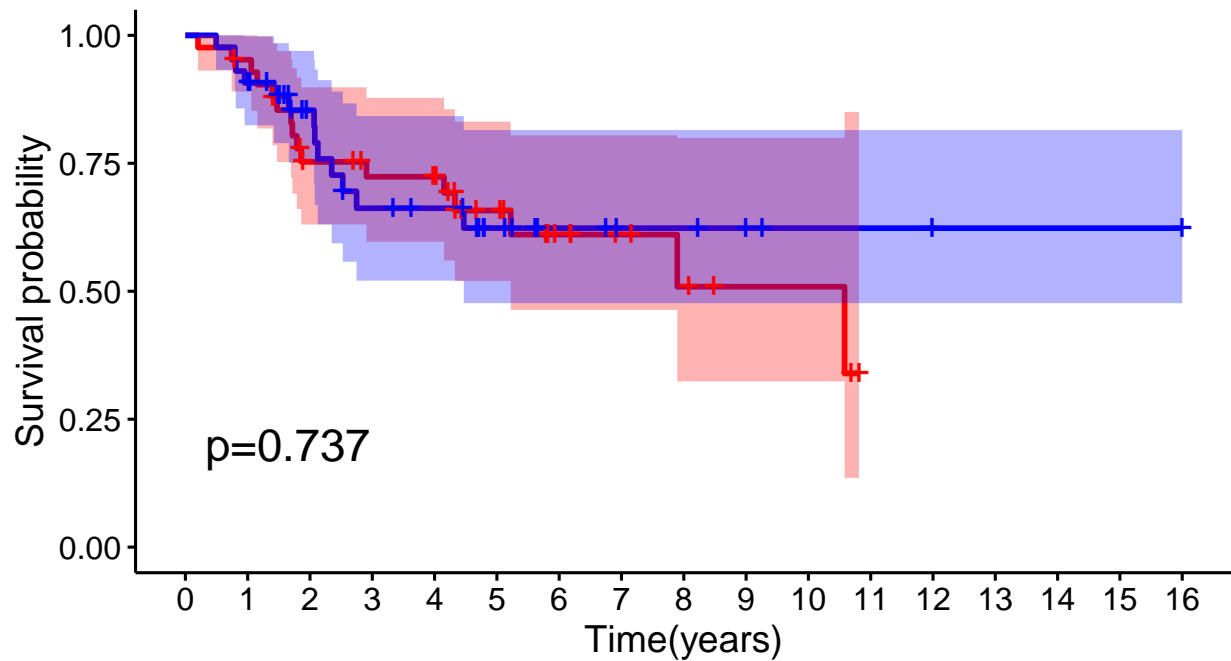

AC137834.2

high

low

|    |    |    |    |    |    |    |   |   |   |    |    |    |    |    |    |
|----|----|----|----|----|----|----|---|---|---|----|----|----|----|----|----|
| 42 | 39 | 28 | 25 | 24 | 16 | 10 | 7 | 5 | 3 | 3  | 0  | 0  | 0  | 0  | 0  |
| 43 | 39 | 27 | 20 | 18 | 12 | 8  | 6 | 6 | 4 | 2  | 2  | 1  | 1  | 1  | 1  |
| 0  | 1  | 2  | 3  | 4  | 5  | 6  | 7 | 8 | 9 | 10 | 11 | 12 | 13 | 14 | 15 |

Time(years)

Supplement: Supplementary Document 1 — Kaplan-Meier curve of the 518 genes associated with survival. [file DataSheet_1.zip › Supplementary Document 1/sur.AC137834.2.pdf]

AC233723.1 + high + low

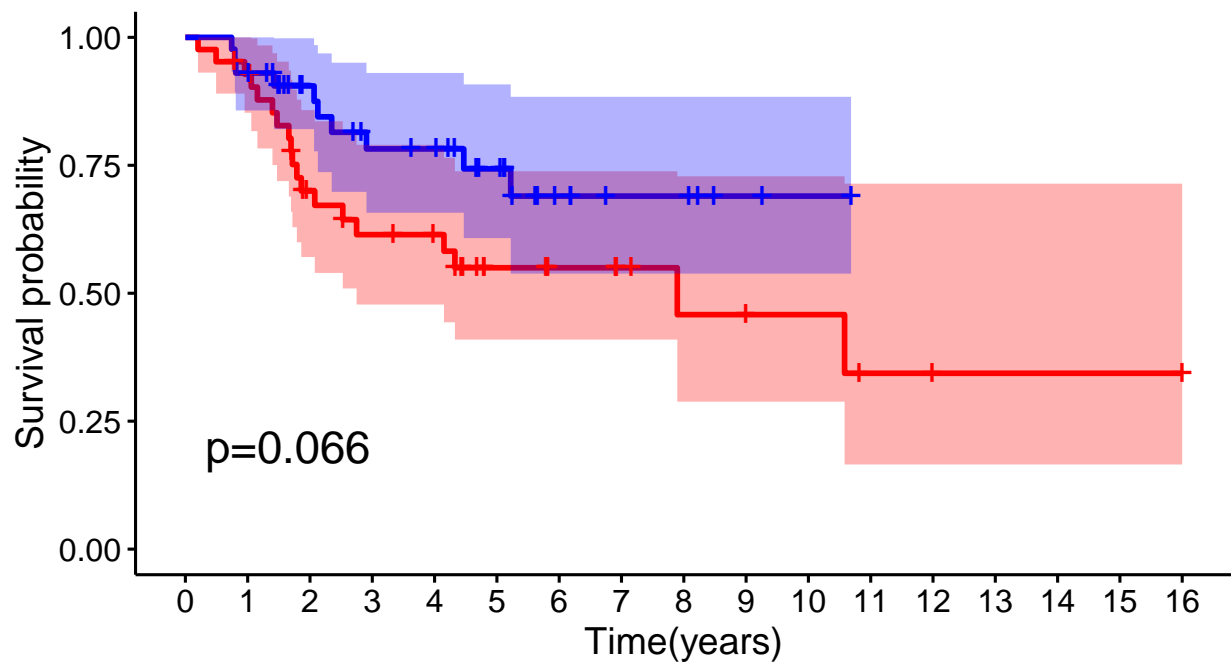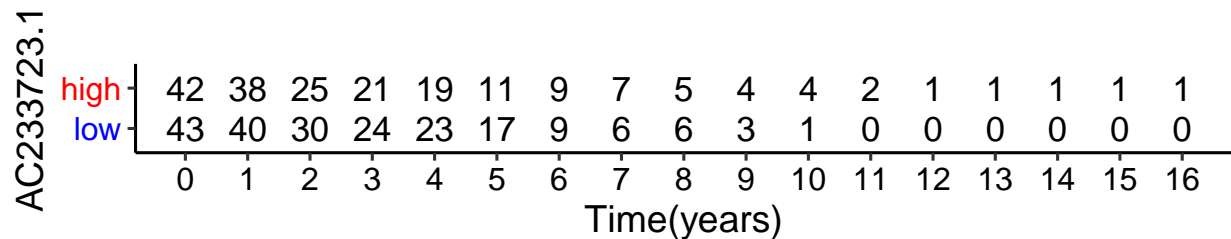

Supplement: Supplementary Document 1 — Kaplan-Meier curve of the 518 genes associated with survival. [file DataSheet_1.zip › Supplementary Document 1/sur.AC233723.1.pdf]

AC243960.3 + high + low

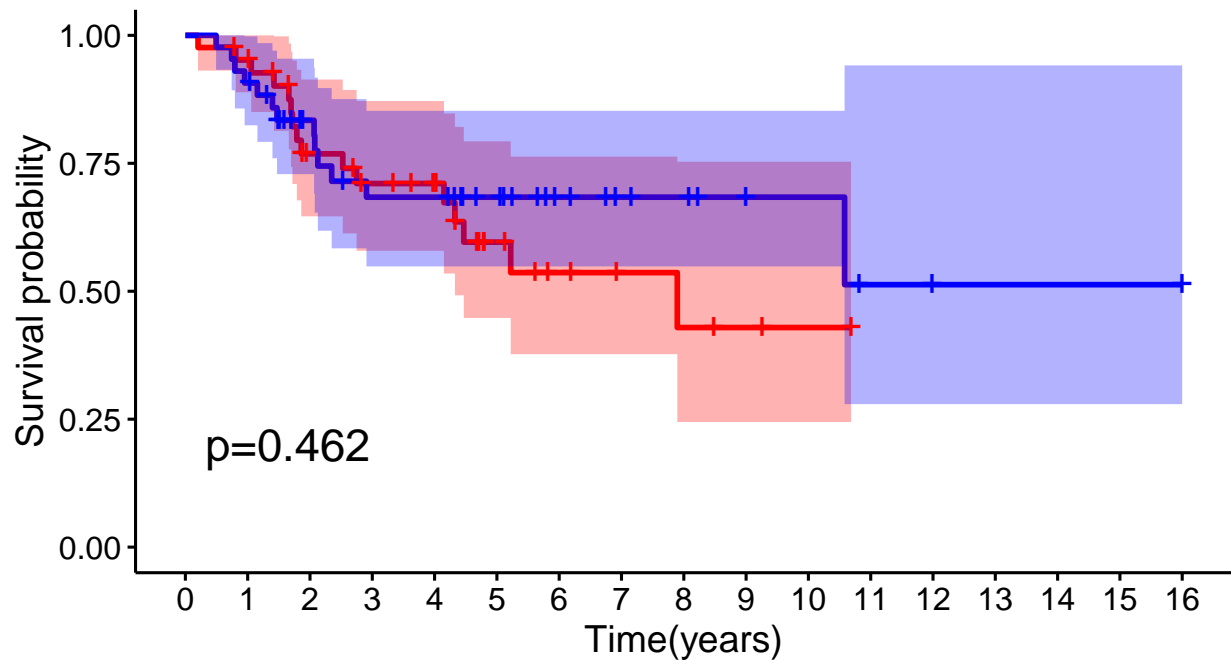

AC243960.3

high

low

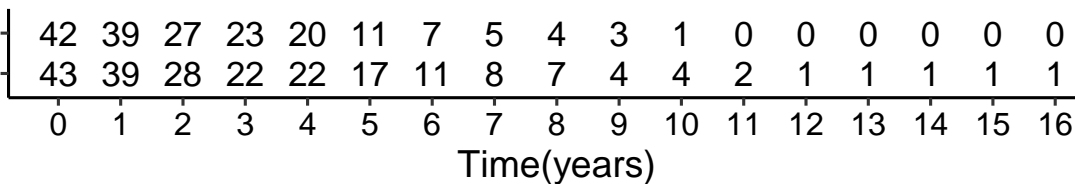

Supplement: Supplementary Document 1 — Kaplan-Meier curve of the 518 genes associated with survival. [file DataSheet_1.zip › Supplementary Document 1/sur.AC243960.3.pdf]

ACP5 + high + low

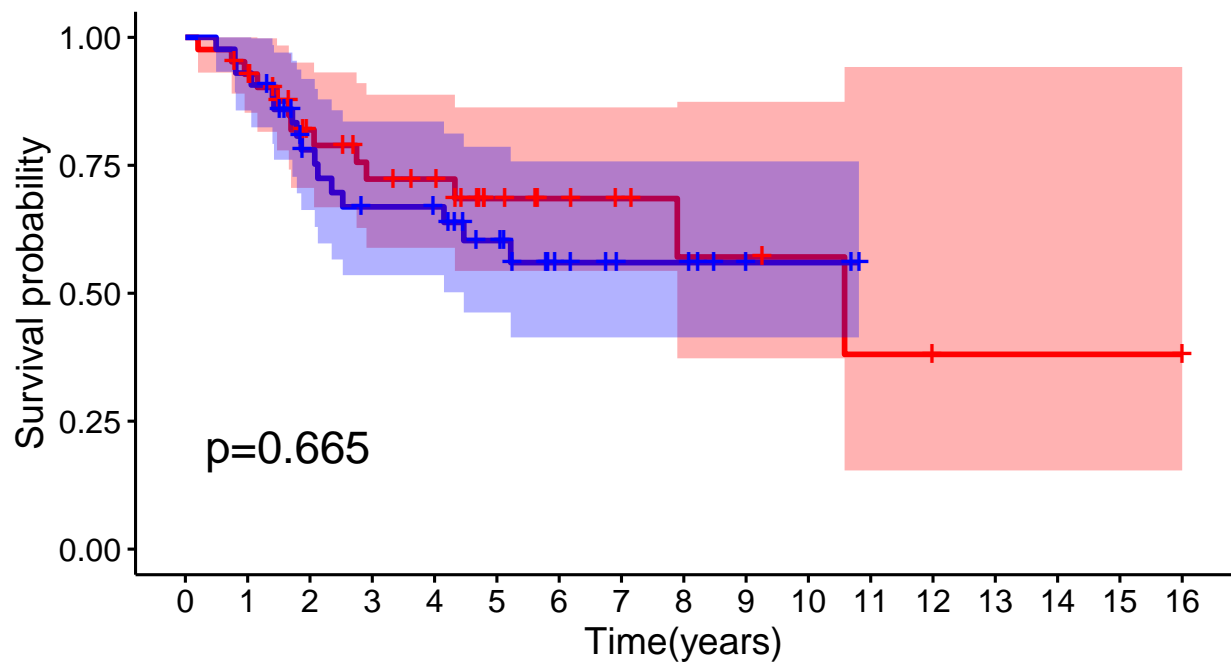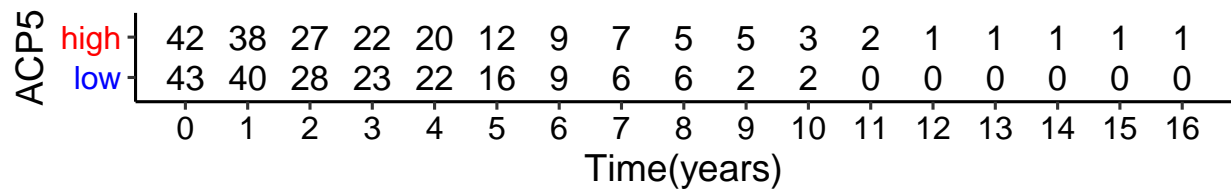

Supplement: Supplementary Document 1 — Kaplan-Meier curve of the 518 genes associated with survival. [file DataSheet_1.zip › Supplementary Document 1/sur.ACP5.pdf]

AFG1L + high + low

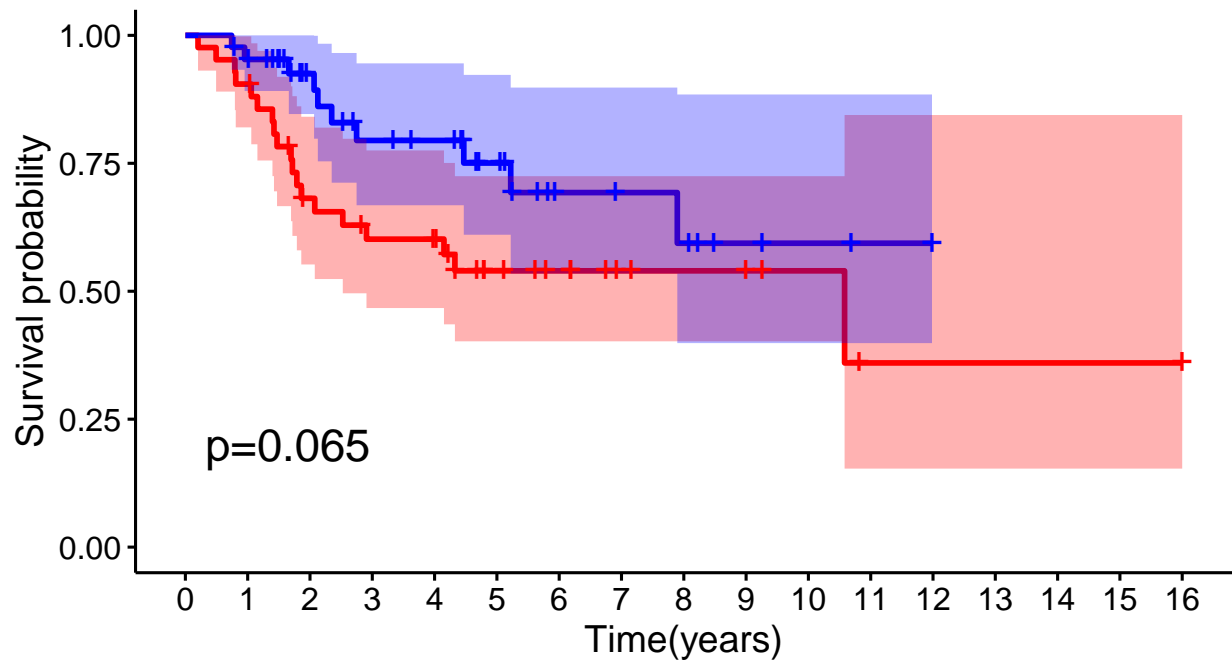

AFG1L

high

low

|    |    |    |    |    |    |    |   |   |   |    |    |    |    |    |    |
|----|----|----|----|----|----|----|---|---|---|----|----|----|----|----|----|
| 42 | 38 | 26 | 22 | 21 | 13 | 10 | 6 | 5 | 4 | 3  | 1  | 1  | 1  | 1  | 1  |
| 43 | 40 | 29 | 23 | 21 | 15 | 8  | 7 | 6 | 3 | 2  | 1  | 0  | 0  | 0  | 0  |
| 0  | 1  | 2  | 3  | 4  | 5  | 6  | 7 | 8 | 9 | 10 | 11 | 12 | 13 | 14 | 15 |

Time(years)

Supplement: Supplementary Document 1 — Kaplan-Meier curve of the 518 genes associated with survival. [file DataSheet_1.zip › Supplementary Document 1/sur.AFG1L.pdf]

AL139095.4 + high + low

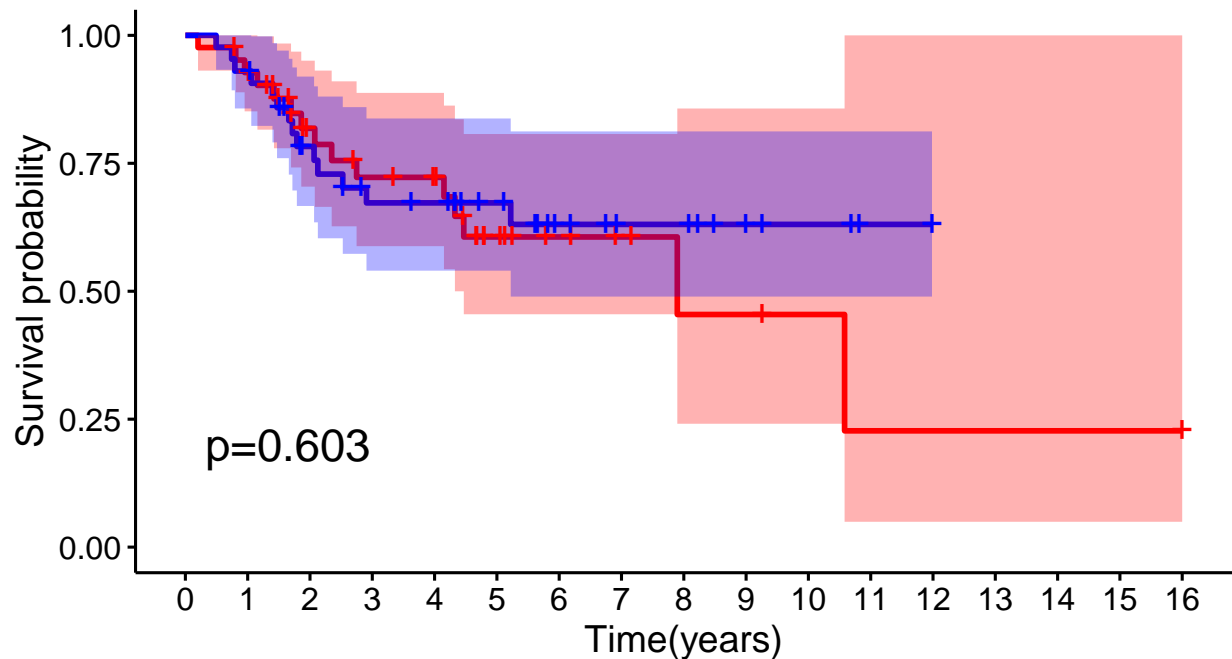

AL139095.4

high  
low

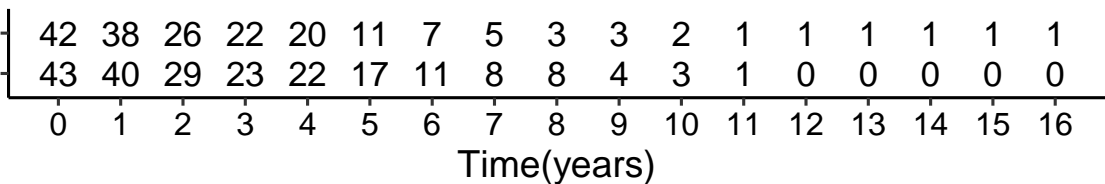

Supplement: Supplementary Document 1 — Kaplan-Meier curve of the 518 genes associated with survival. [file DataSheet_1.zip › Supplementary Document 1/sur.AL139095.4.pdf]

AL139241.1 + high + low

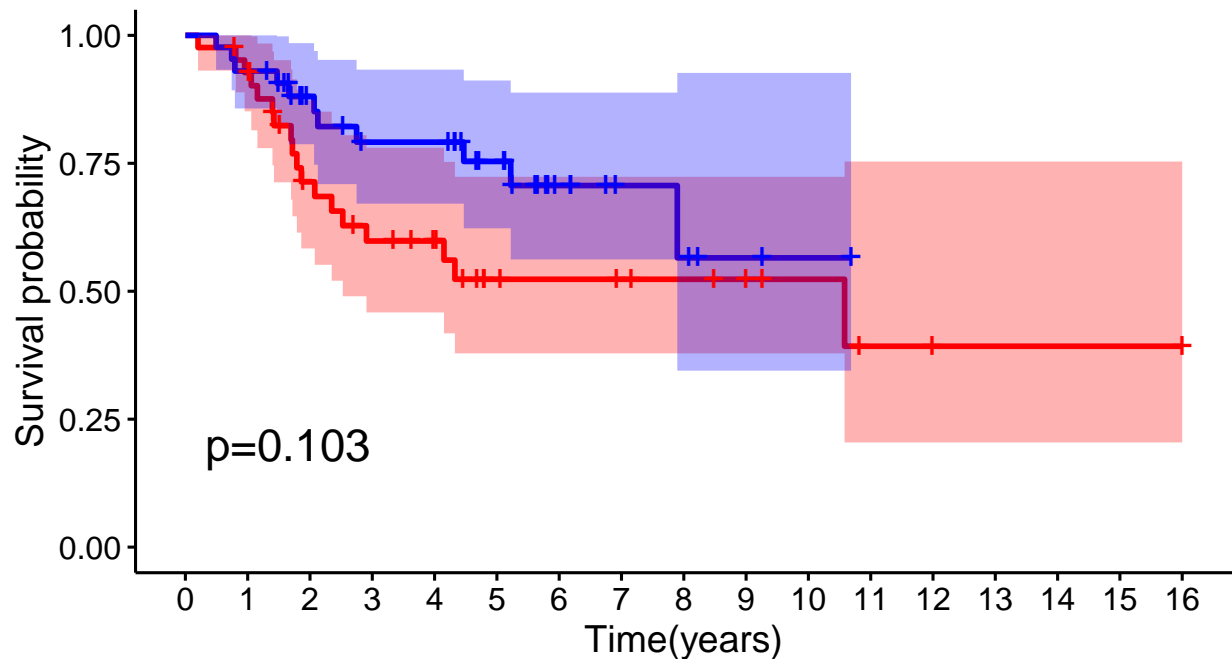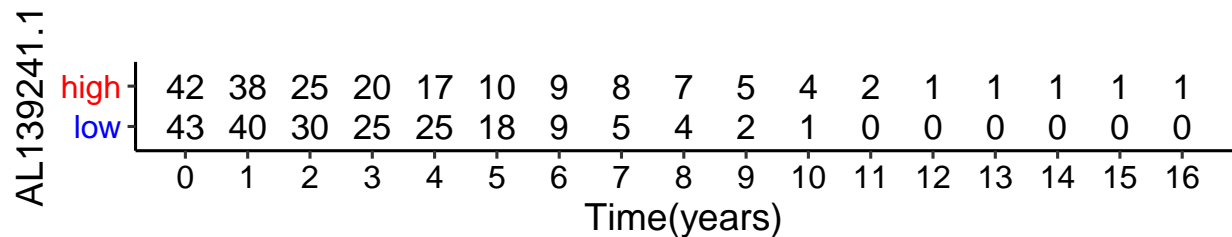

Supplement: Supplementary Document 1 — Kaplan-Meier curve of the 518 genes associated with survival. [file DataSheet_1.zip › Supplementary Document 1/sur.AL139241.1.pdf]

AL157400.4    high    low

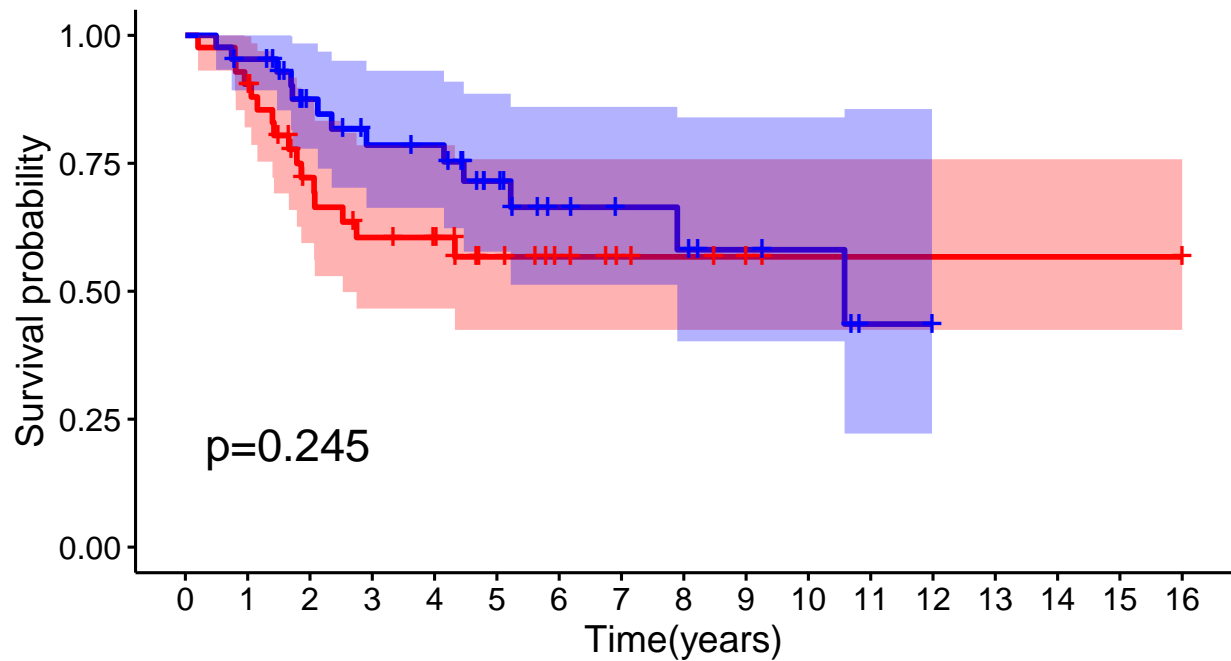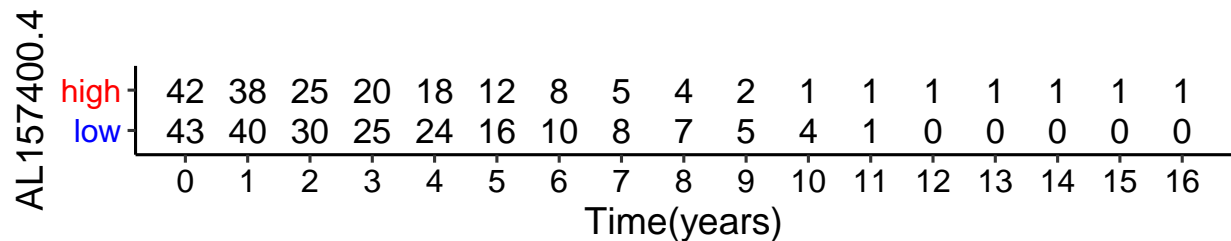

Supplement: Supplementary Document 1 — Kaplan-Meier curve of the 518 genes associated with survival. [file DataSheet_1.zip › Supplementary Document 1/sur.AL157400.4.pdf]

AL158211.1 + high + low

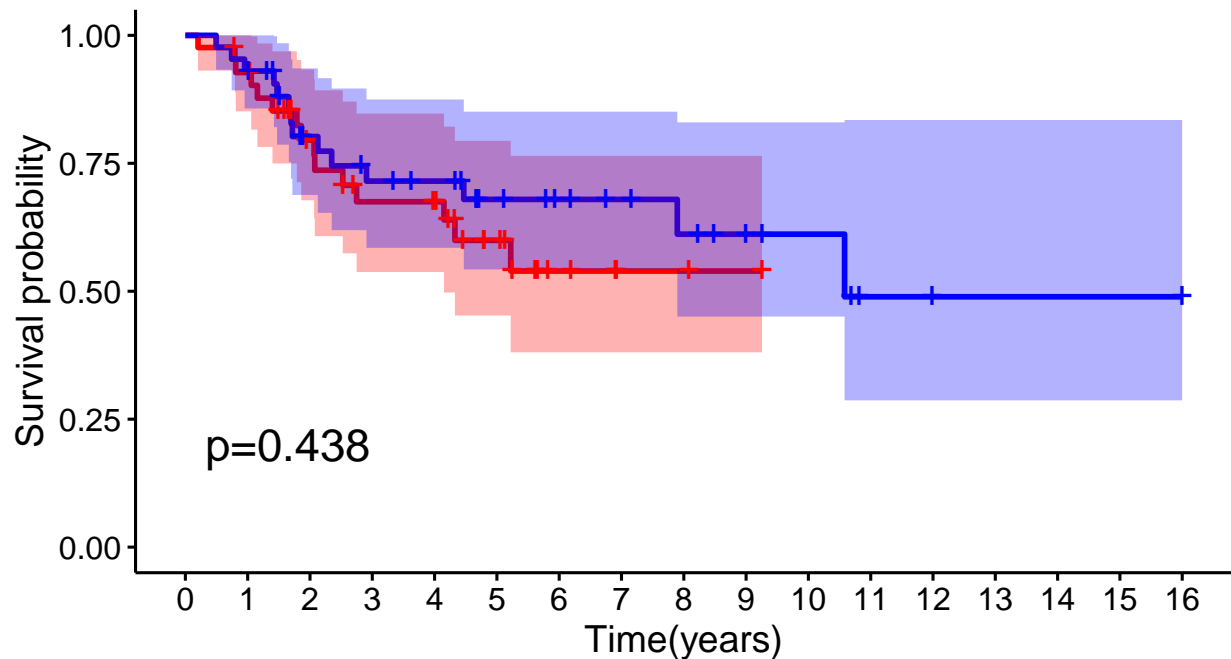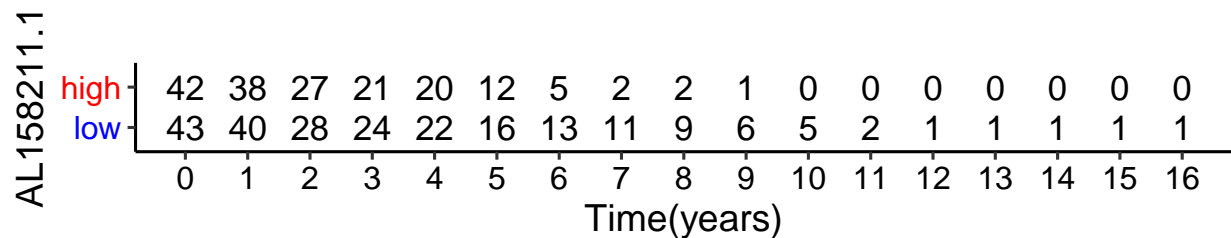

Supplement: Supplementary Document 1 — Kaplan-Meier curve of the 518 genes associated with survival. [file DataSheet_1.zip › Supplementary Document 1/sur.AL158211.1.pdf]

AL161630.1 + high + low

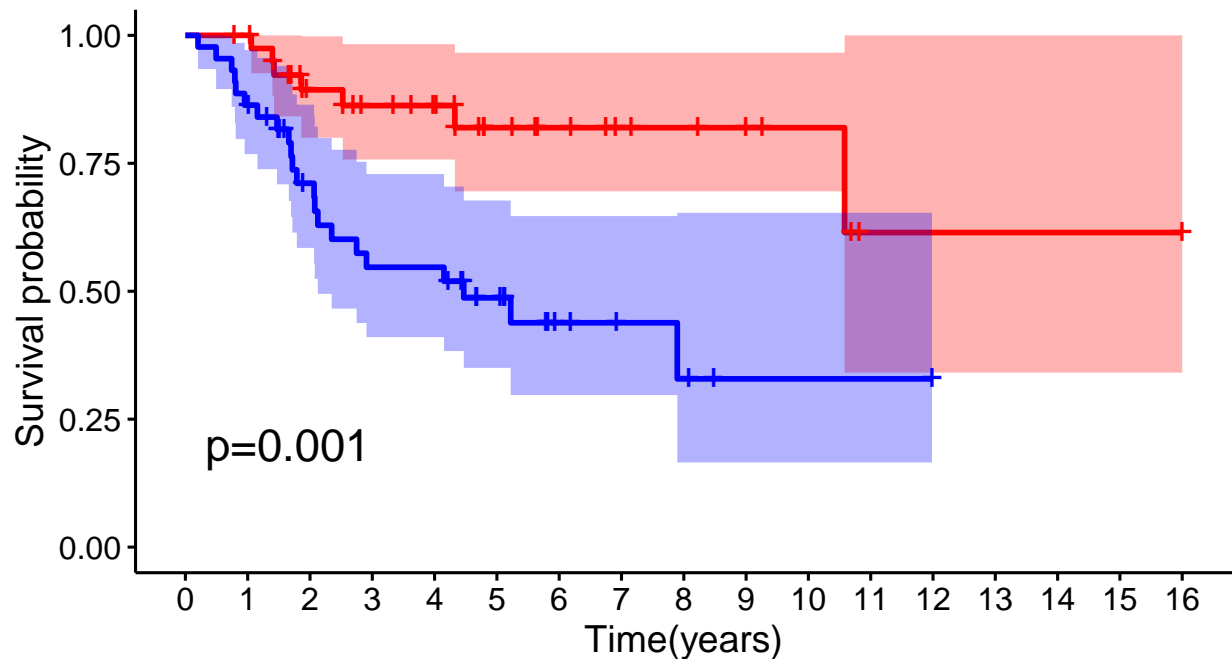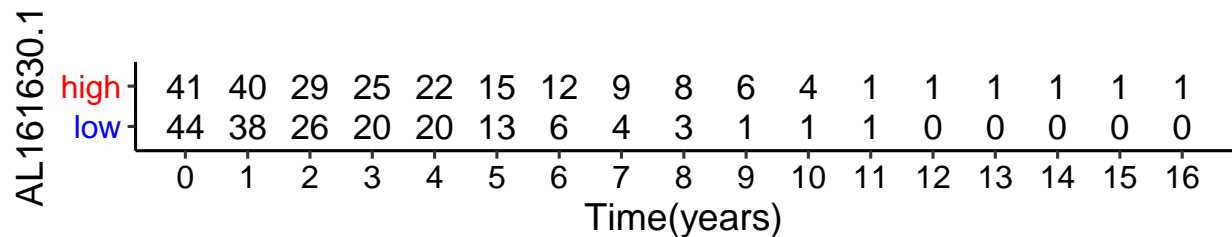

Supplement: Supplementary Document 1 — Kaplan-Meier curve of the 518 genes associated with survival. [file DataSheet_1.zip › Supplementary Document 1/sur.AL161630.1.pdf]

AL358335.2

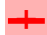

high

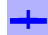

low

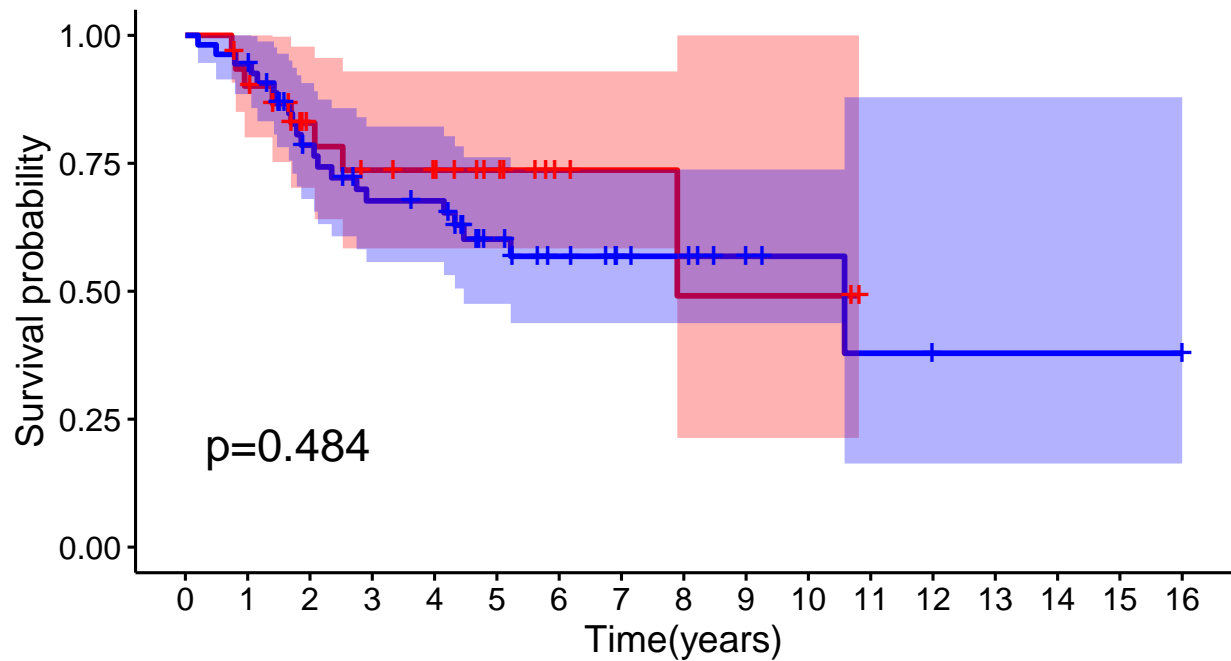

AL358335.2

high

low

|    |    |    |    |    |    |    |    |   |   |    |    |    |    |    |    |
|----|----|----|----|----|----|----|----|---|---|----|----|----|----|----|----|
| 31 | 27 | 18 | 15 | 13 | 9  | 4  | 3  | 2 | 2 | 2  | 0  | 0  | 0  | 0  | 0  |
| 54 | 51 | 37 | 30 | 29 | 19 | 14 | 10 | 9 | 5 | 3  | 2  | 1  | 1  | 1  | 1  |
| 0  | 1  | 2  | 3  | 4  | 5  | 6  | 7  | 8 | 9 | 10 | 11 | 12 | 13 | 14 | 15 |

Time(years)

Supplement: Supplementary Document 1 — Kaplan-Meier curve of the 518 genes associated with survival. [file DataSheet_1.zip › Supplementary Document 1/sur.AL358335.2.pdf]

AL365434.2

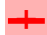

high

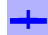

low

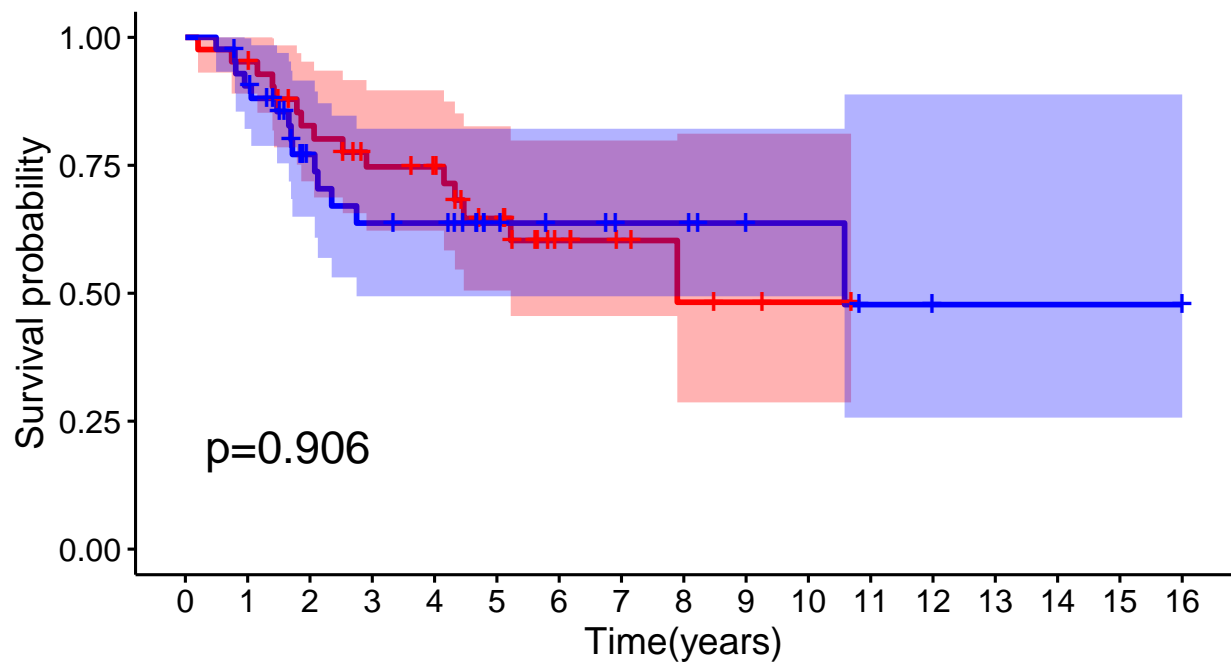

AL365434.2

high

low

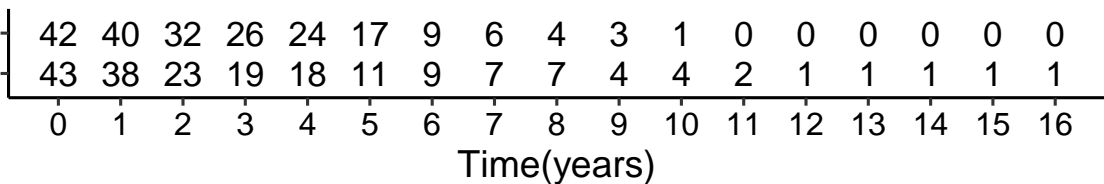

Supplement: Supplementary Document 1 — Kaplan-Meier curve of the 518 genes associated with survival. [file DataSheet_1.zip › Supplementary Document 1/sur.AL365434.2.pdf]

AL451060.1 + high + low

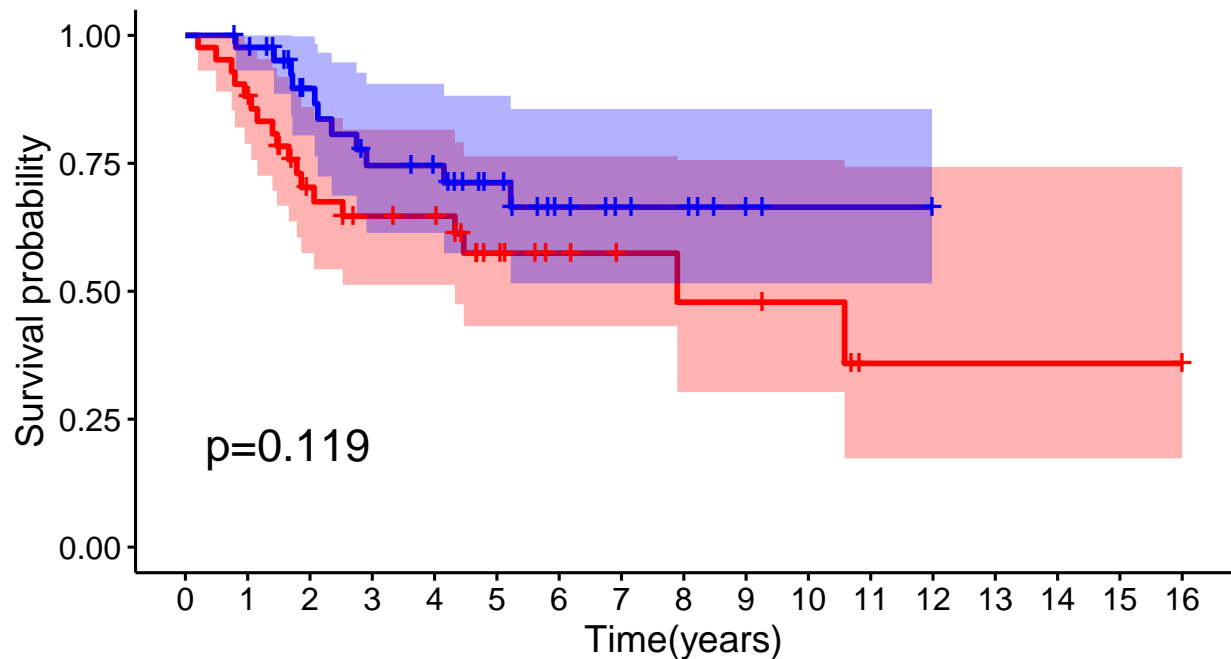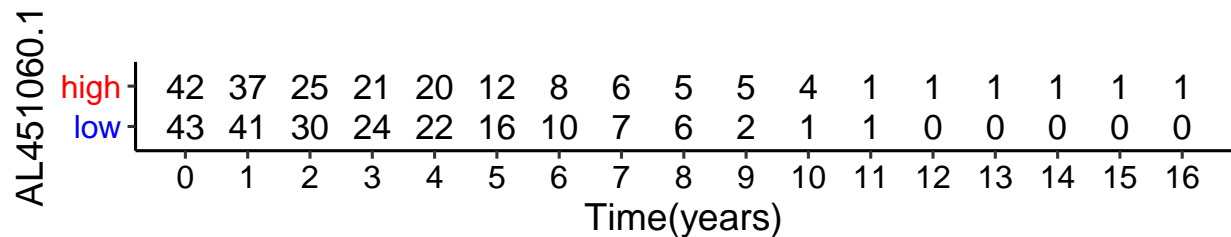

Supplement: Supplementary Document 1 — Kaplan-Meier curve of the 518 genes associated with survival. [file DataSheet_1.zip › Supplementary Document 1/sur.AL451060.1.pdf]

AL451069.1 + high + low

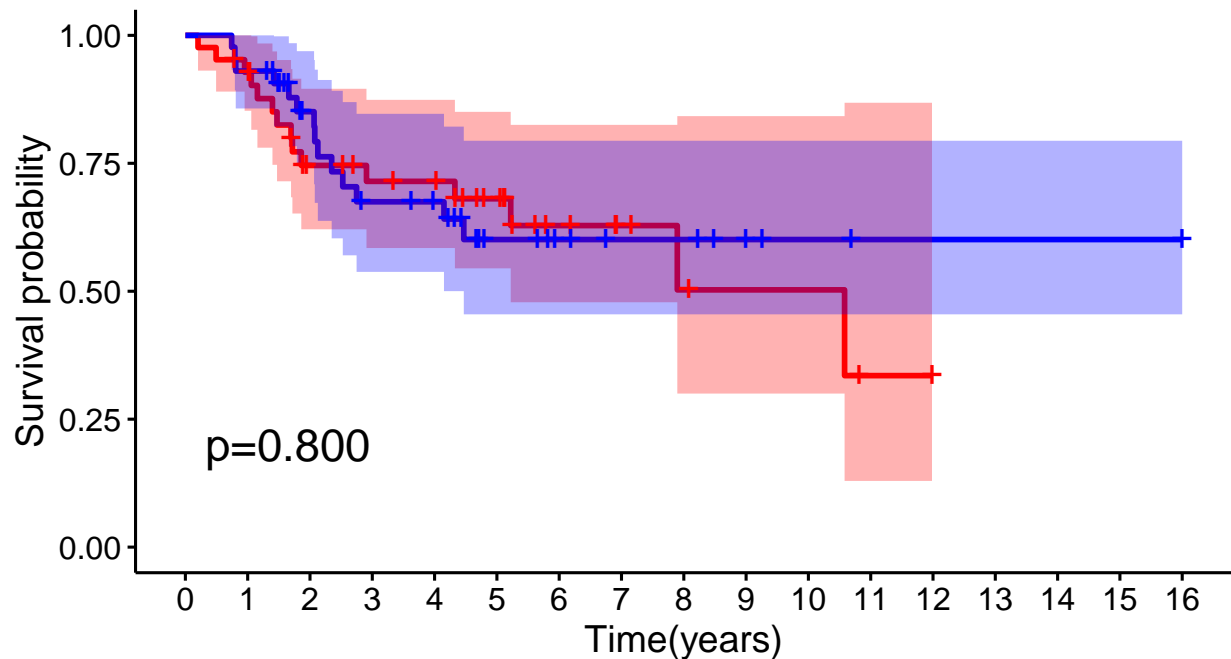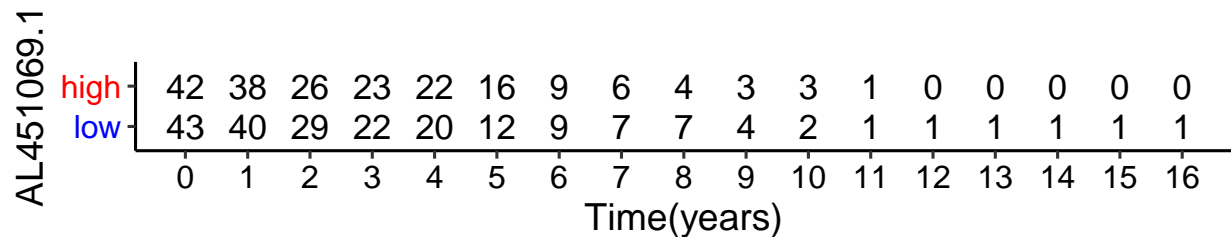

Supplement: Supplementary Document 1 — Kaplan-Meier curve of the 518 genes associated with survival. [file DataSheet_1.zip › Supplementary Document 1/sur.AL451069.1.pdf]

AL512328.1 + high + low

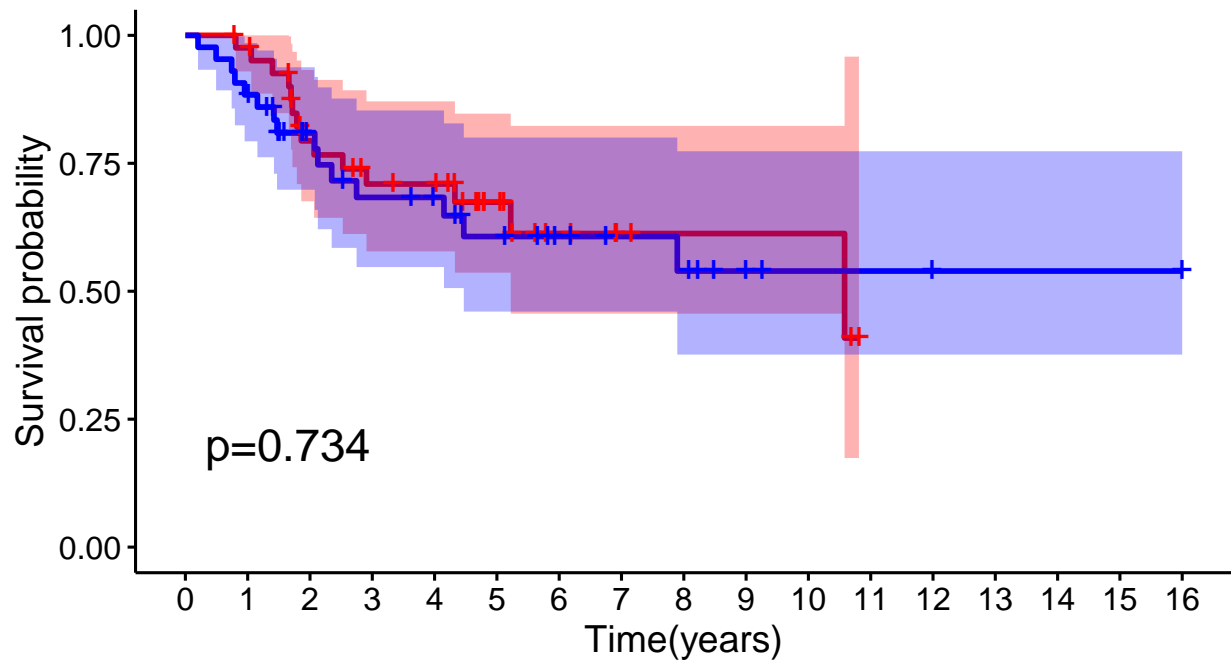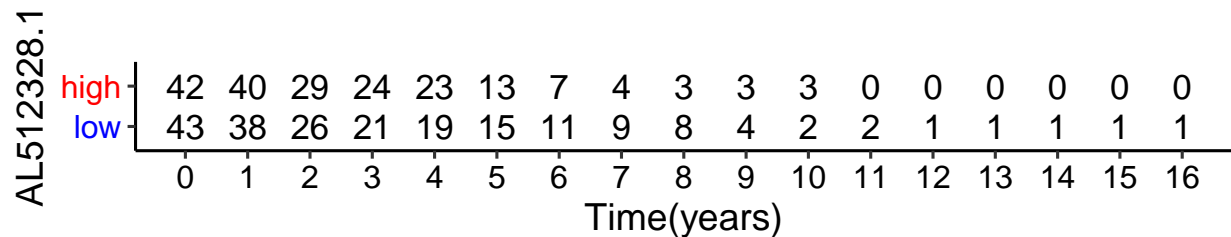

Supplement: Supplementary Document 1 — Kaplan-Meier curve of the 518 genes associated with survival. [file DataSheet_1.zip › Supplementary Document 1/sur.AL512328.1.pdf]

AL513314.1 + high + low

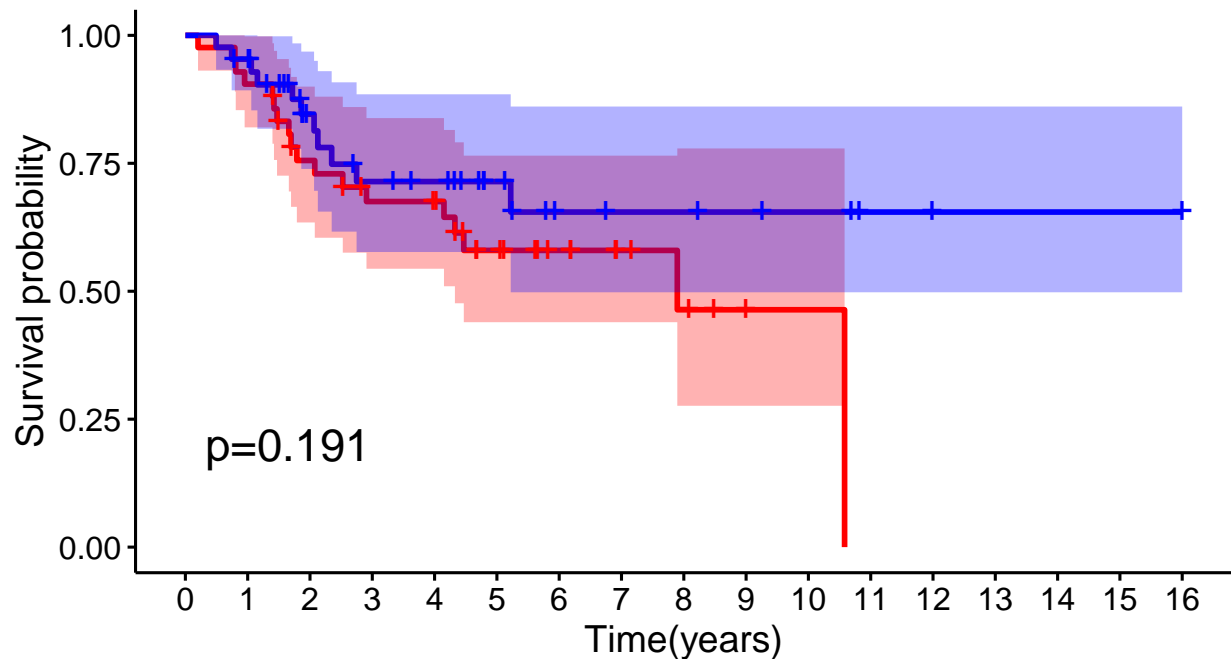

AL513314.1

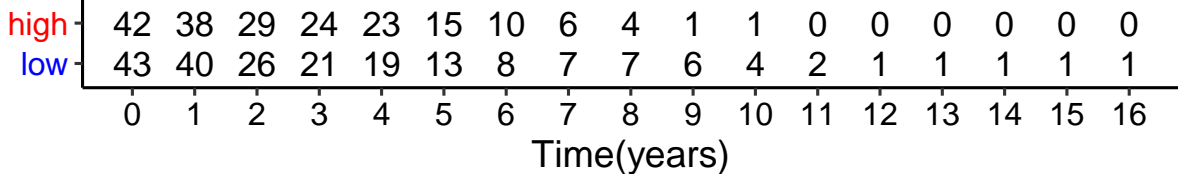

Supplement: Supplementary Document 1 — Kaplan-Meier curve of the 518 genes associated with survival. [file DataSheet_1.zip › Supplementary Document 1/sur.AL513314.1.pdf]

AL583859.2 + high + low

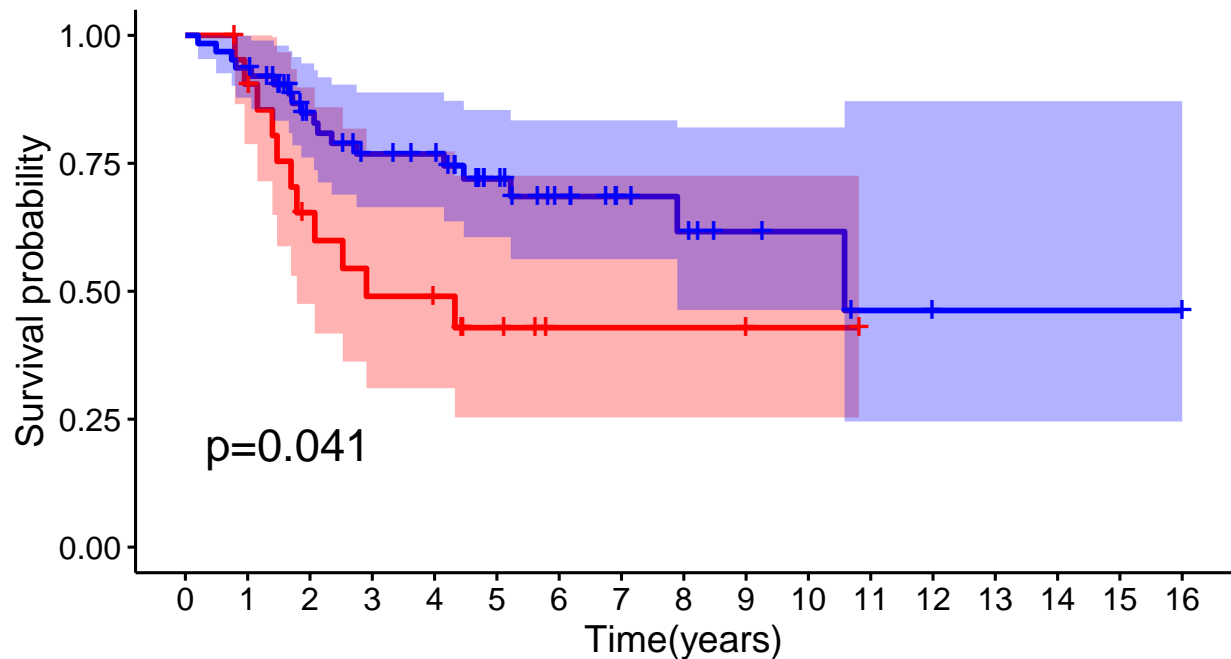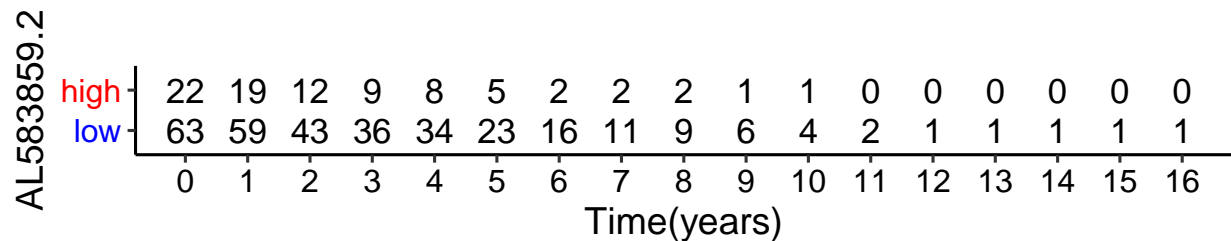

Supplement: Supplementary Document 1 — Kaplan-Meier curve of the 518 genes associated with survival. [file DataSheet_1.zip › Supplementary Document 1/sur.AL583859.2.pdf]

AMELX + high + low

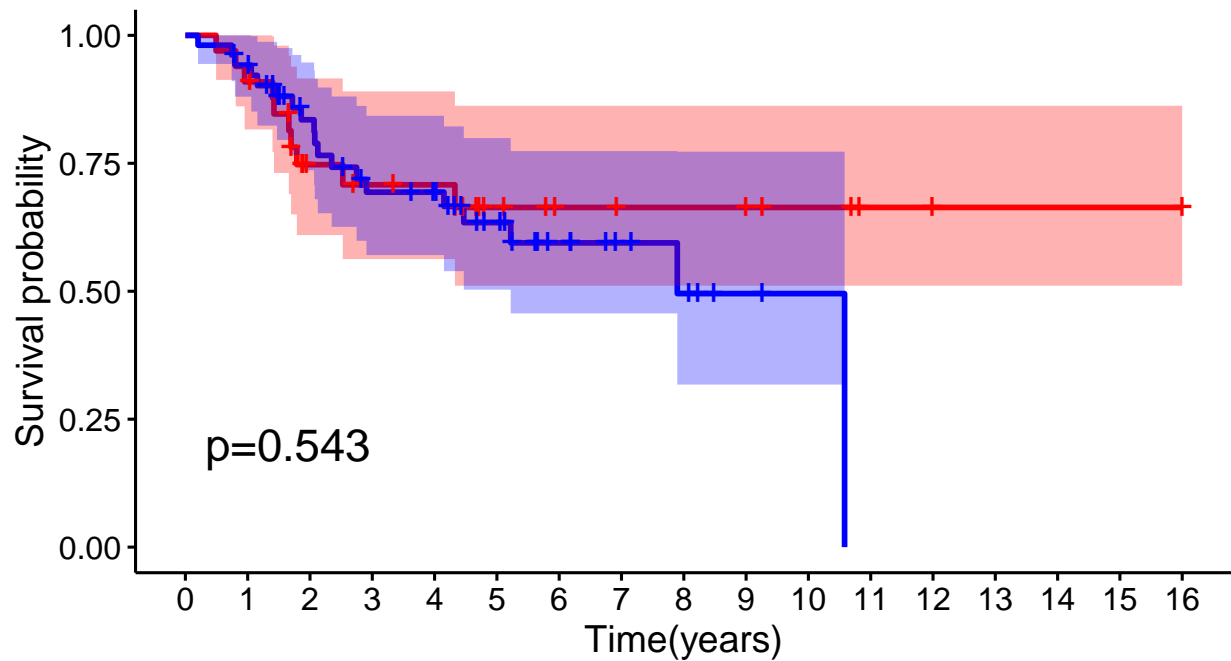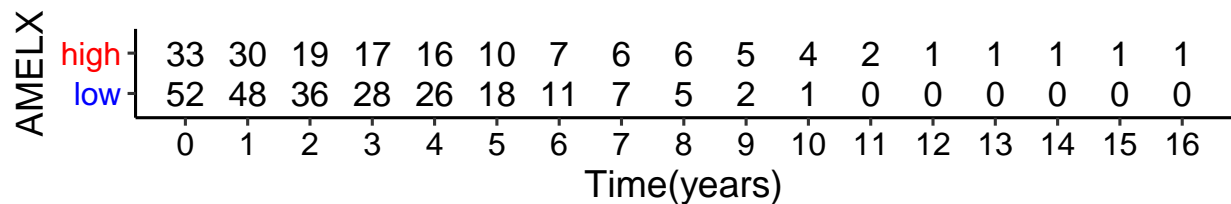

Supplement: Supplementary Document 1 — Kaplan-Meier curve of the 518 genes associated with survival. [file DataSheet_1.zip › Supplementary Document 1/sur.AMELX.pdf]

ANGPT1 high low

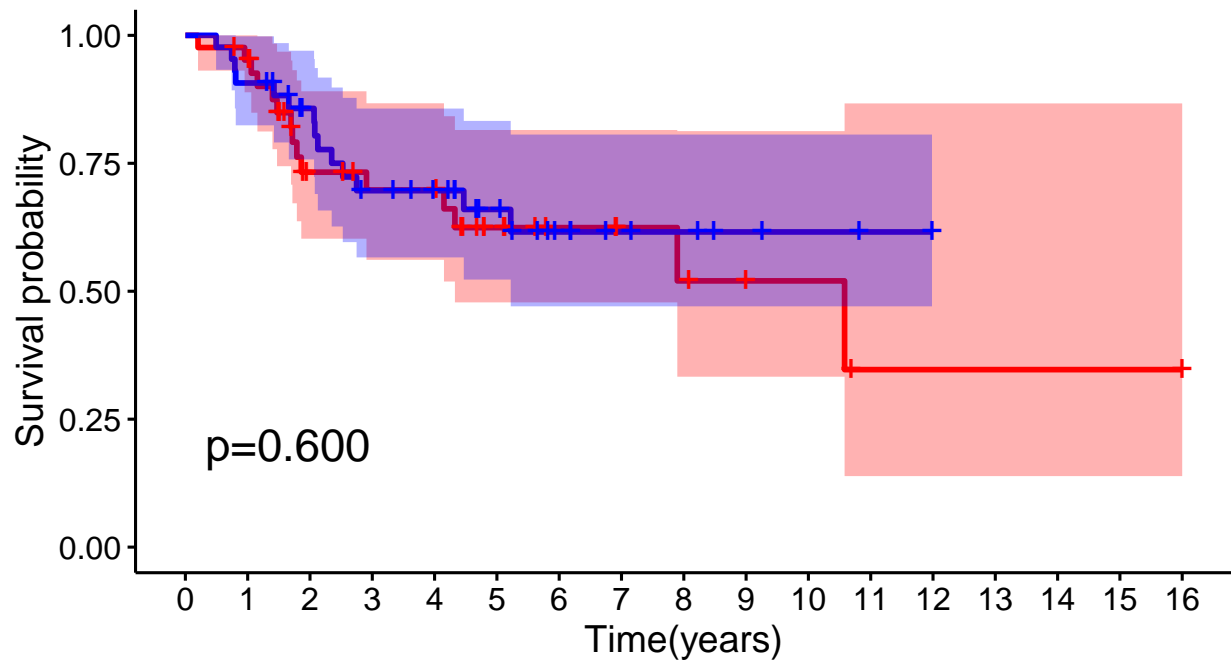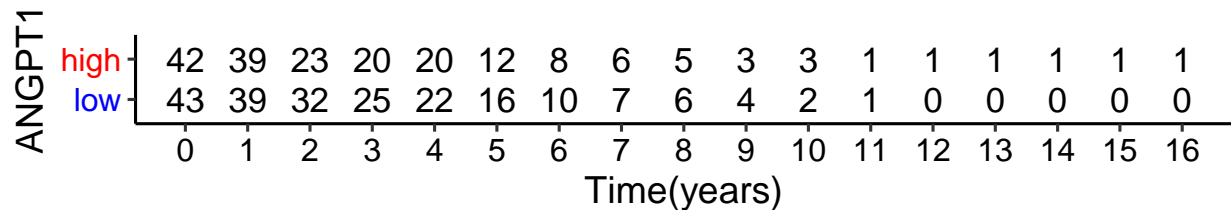

Supplement: Supplementary Document 1 — Kaplan-Meier curve of the 518 genes associated with survival. [file DataSheet_1.zip › Supplementary Document 1/sur.ANGPT1.pdf]

ANO5 + high + low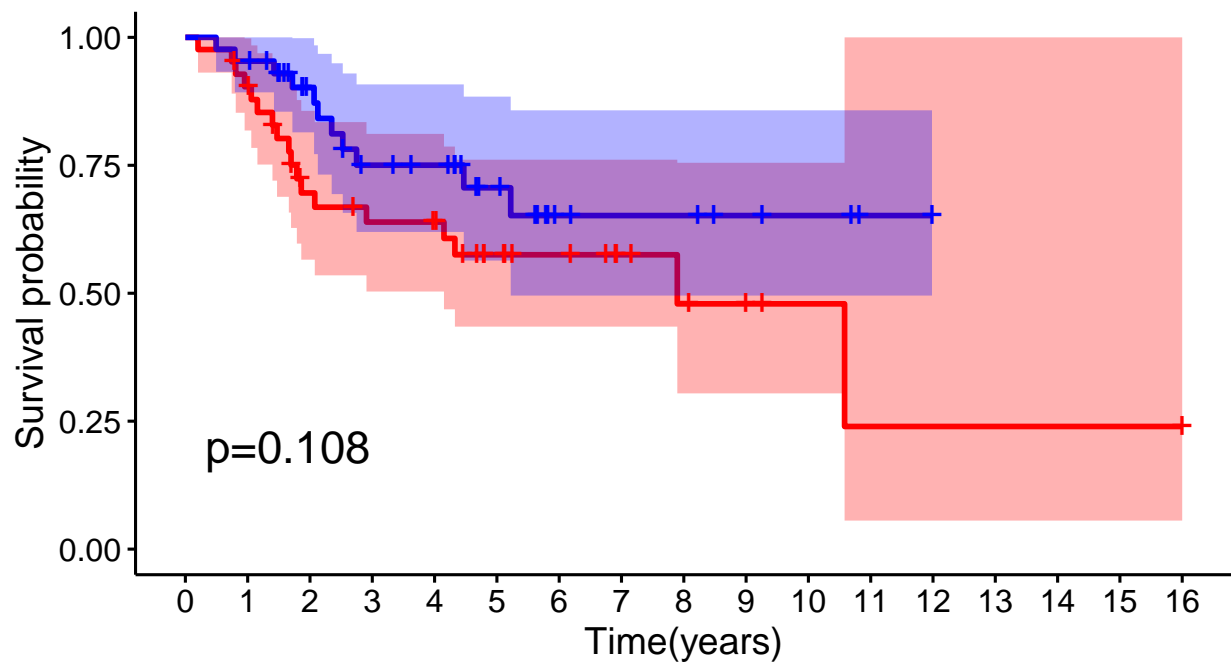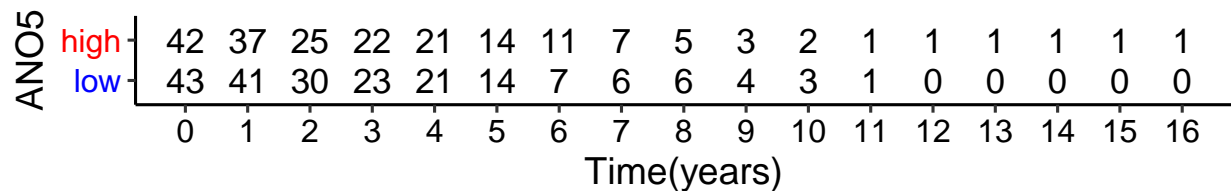

Supplement: Supplementary Document 1 — Kaplan-Meier curve of the 518 genes associated with survival. [file DataSheet_1.zip › Supplementary Document 1/sur.ANO5.pdf]

ANXA13 high low

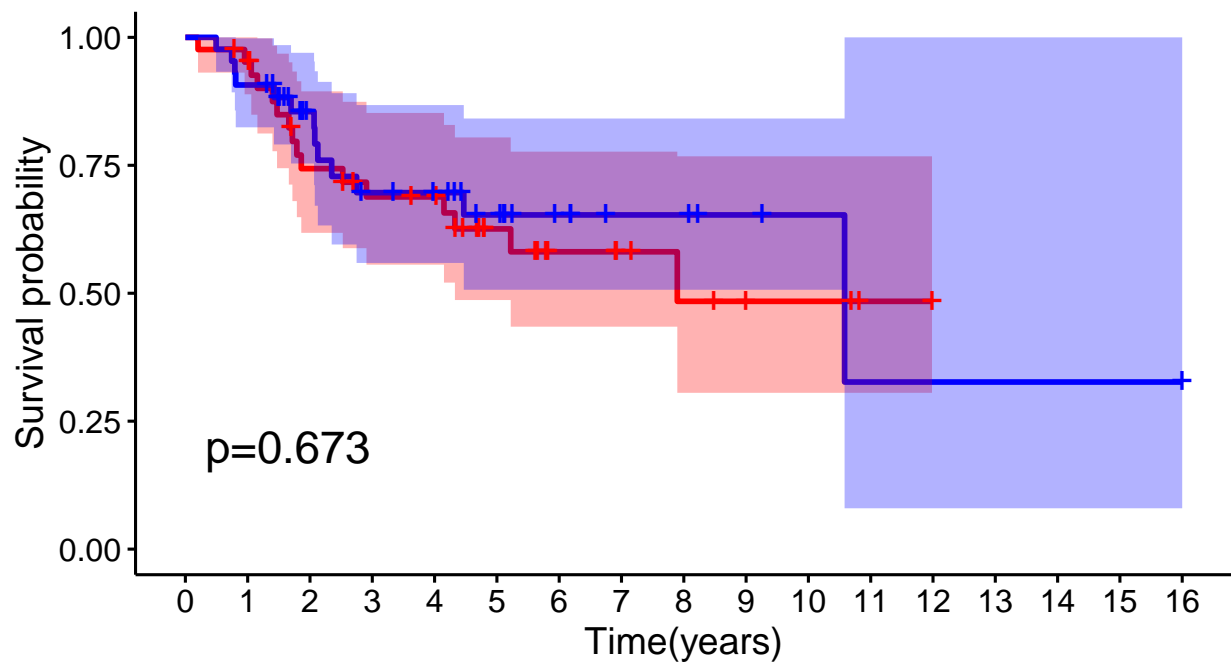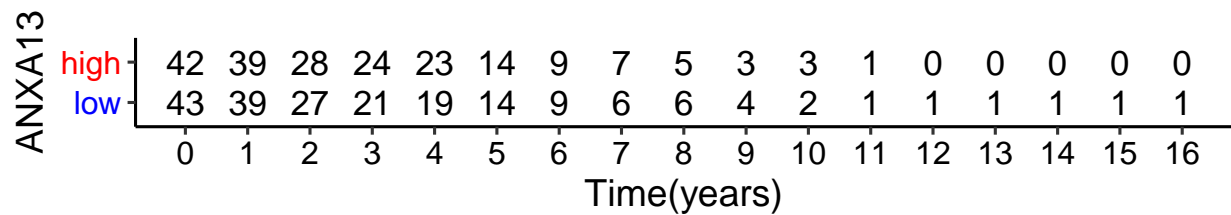

Supplement: Supplementary Document 1 — Kaplan-Meier curve of the 518 genes associated with survival. [file DataSheet_1.zip › Supplementary Document 1/sur.ANXA13.pdf]

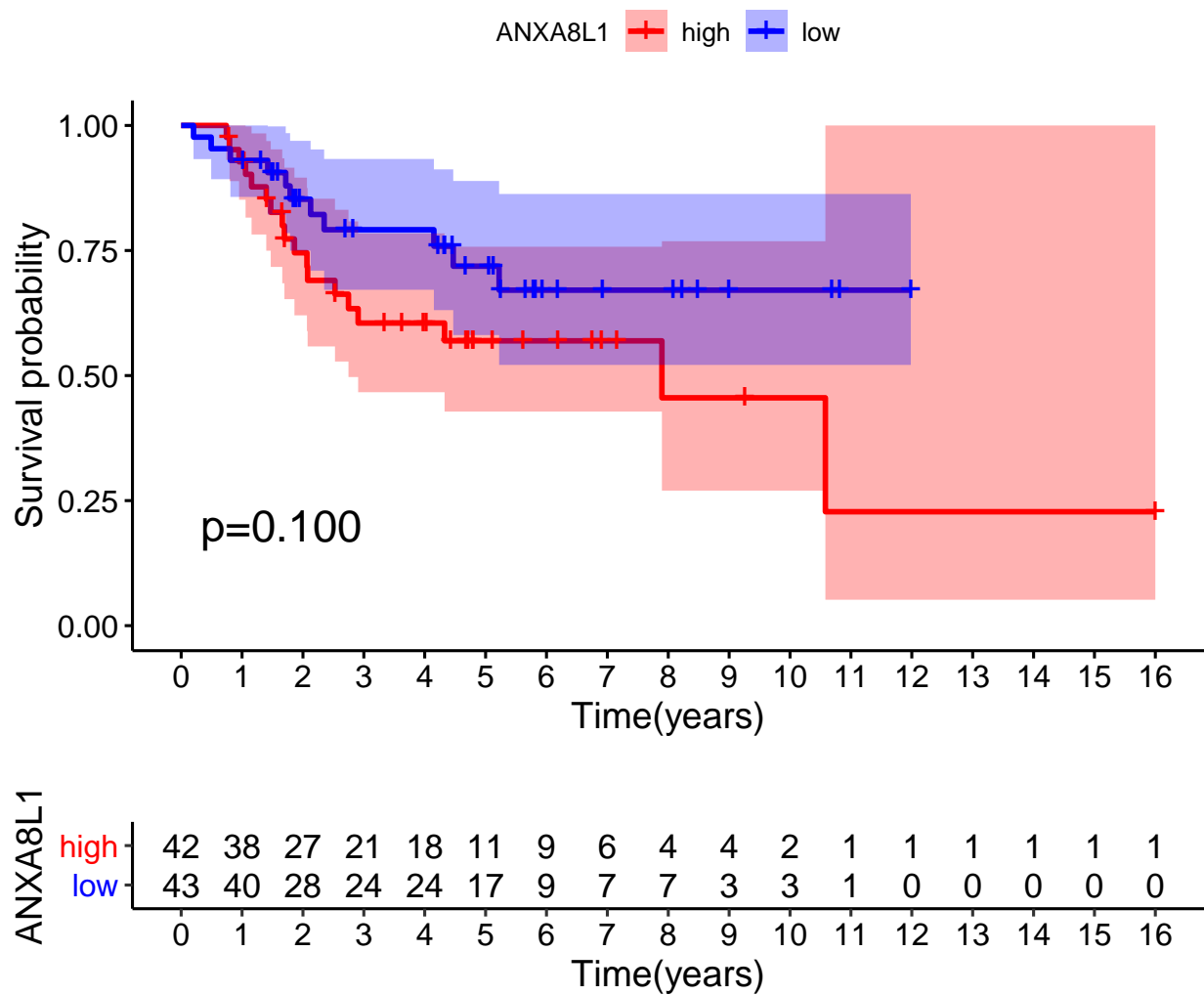

Supplement: Supplementary Document 1 — Kaplan-Meier curve of the 518 genes associated with survival. [file DataSheet_1.zip › Supplementary Document 1/sur.ANXA8L1.pdf]

AP000295.1 + high + low

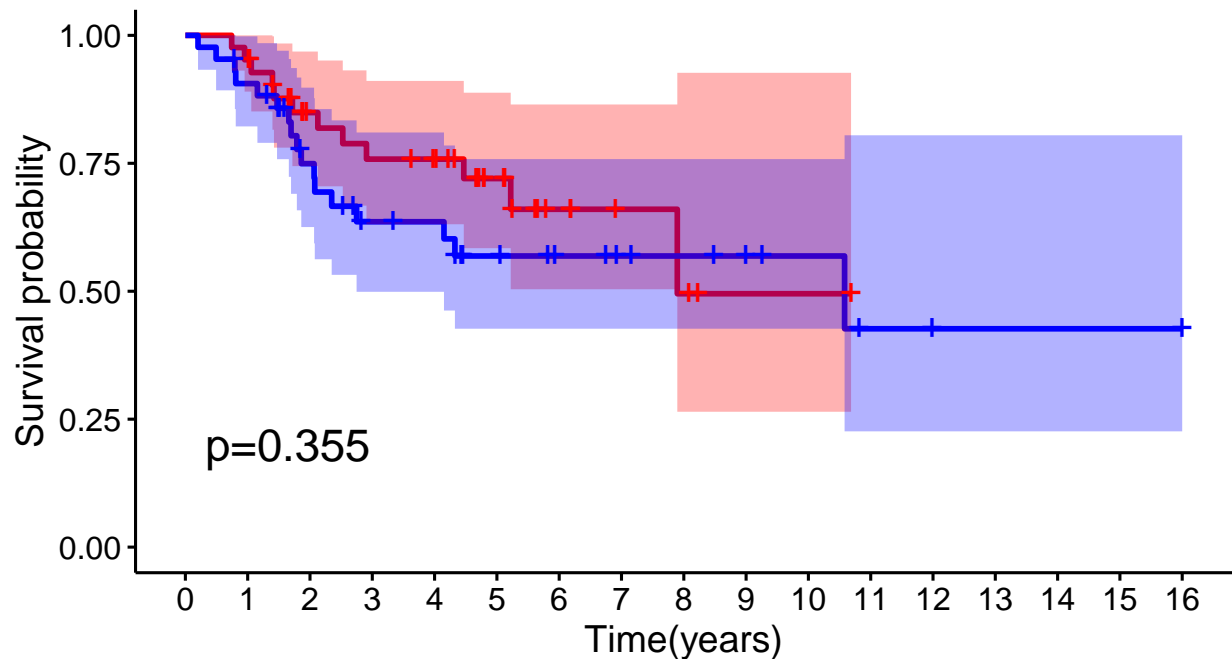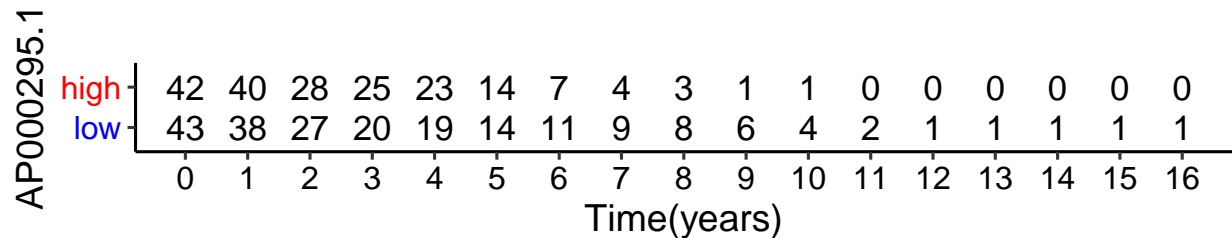

Supplement: Supplementary Document 1 — Kaplan-Meier curve of the 518 genes associated with survival. [file DataSheet_1.zip › Supplementary Document 1/sur.AP000295.1.pdf]

AP000302.1 + high + low

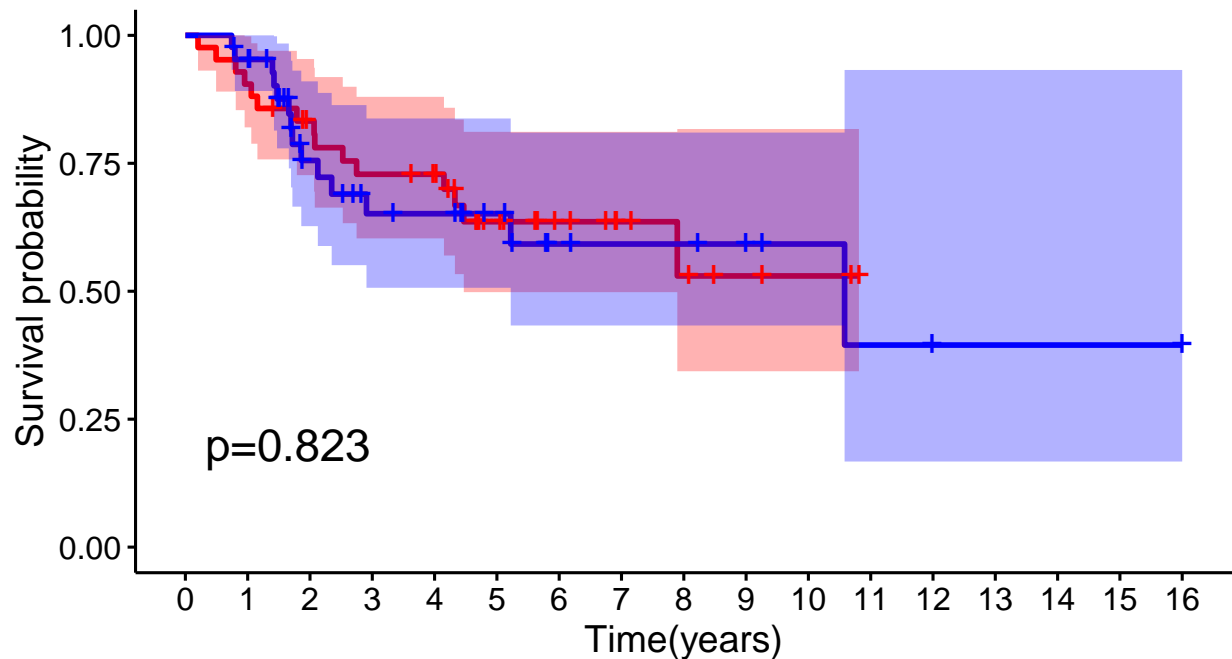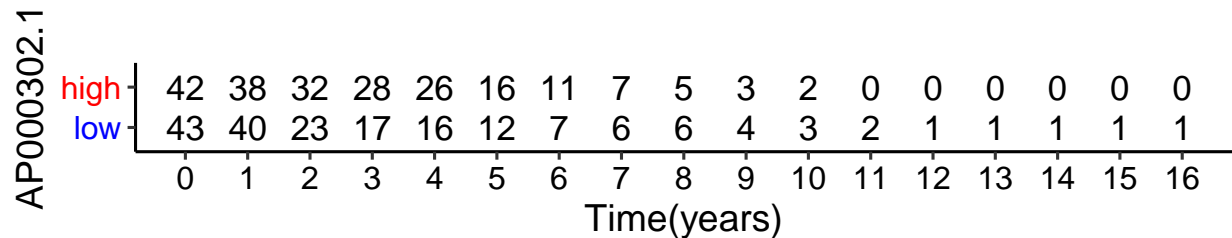

Supplement: Supplementary Document 1 — Kaplan-Meier curve of the 518 genes associated with survival. [file DataSheet_1.zip › Supplementary Document 1/sur.AP000302.1.pdf]

AP000812.1

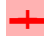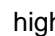

high low

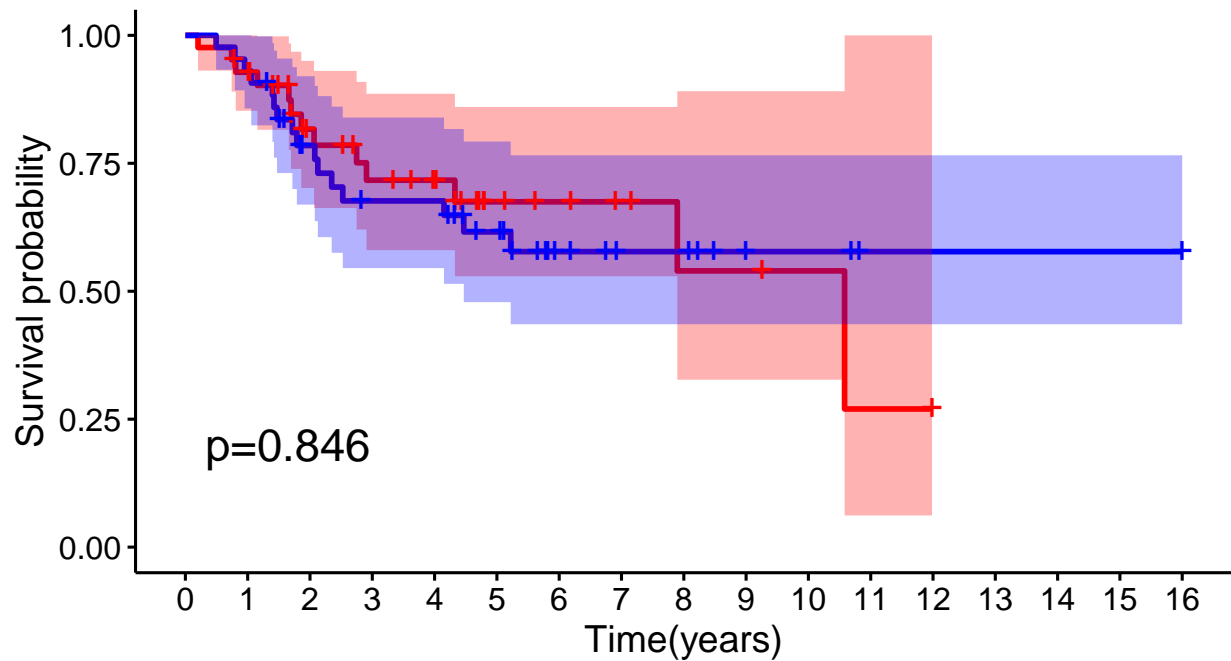

AP000812.1

high

low

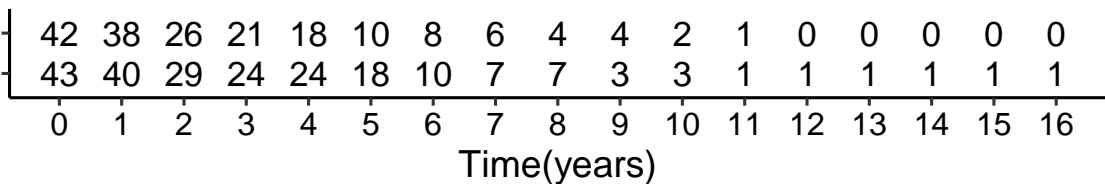

Supplement: Supplementary Document 1 — Kaplan-Meier curve of the 518 genes associated with survival. [file DataSheet_1.zip › Supplementary Document 1/sur.AP000812.1.pdf]

AP000851.1

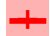

high

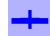

low

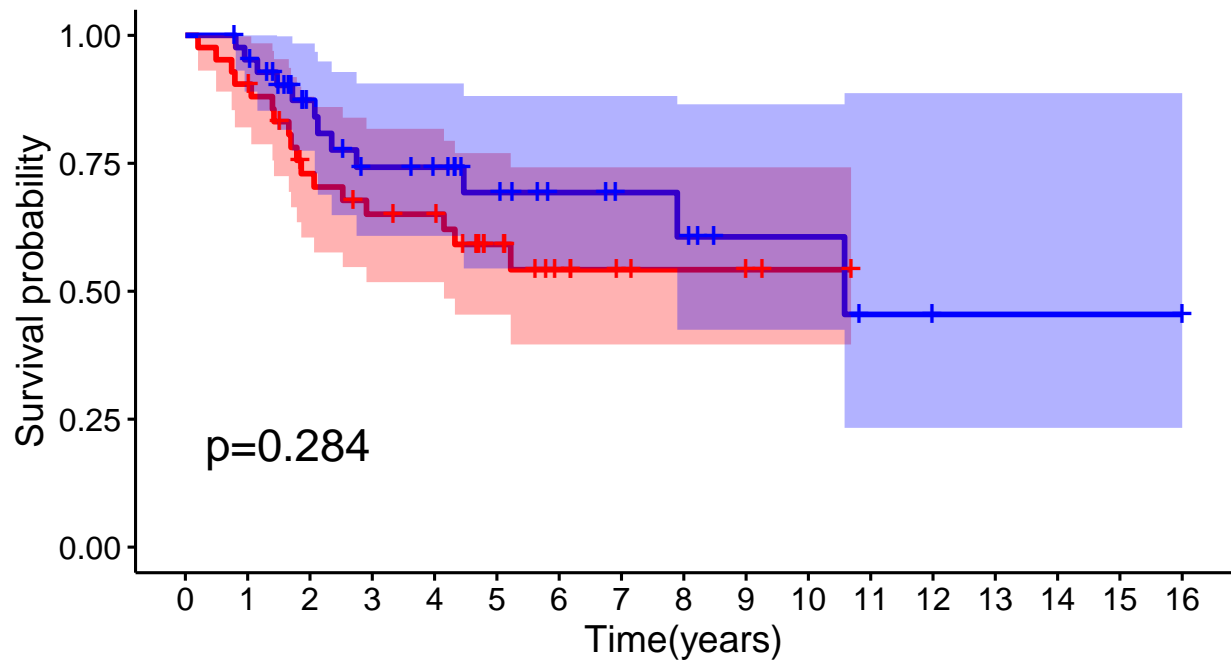

AP000851.1

high

low

|    |    |    |    |    |    |    |   |   |   |    |    |    |    |    |    |
|----|----|----|----|----|----|----|---|---|---|----|----|----|----|----|----|
| 42 | 38 | 28 | 24 | 23 | 14 | 8  | 5 | 4 | 3 | 1  | 0  | 0  | 0  | 0  | 0  |
| 43 | 40 | 27 | 21 | 19 | 14 | 10 | 8 | 7 | 4 | 4  | 2  | 1  | 1  | 1  | 1  |
| 0  | 1  | 2  | 3  | 4  | 5  | 6  | 7 | 8 | 9 | 10 | 11 | 12 | 13 | 14 | 15 |

Time(years)

Supplement: Supplementary Document 1 — Kaplan-Meier curve of the 518 genes associated with survival. [file DataSheet_1.zip › Supplementary Document 1/sur.AP000851.1.pdf]

AP000851.2 + high + low

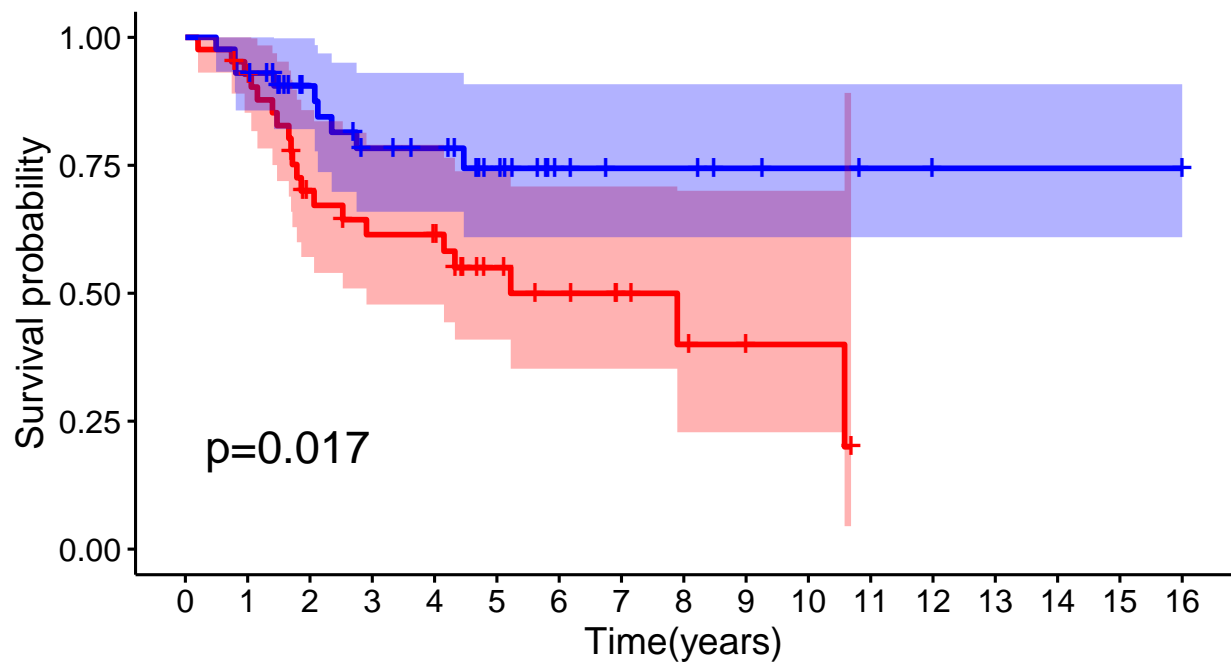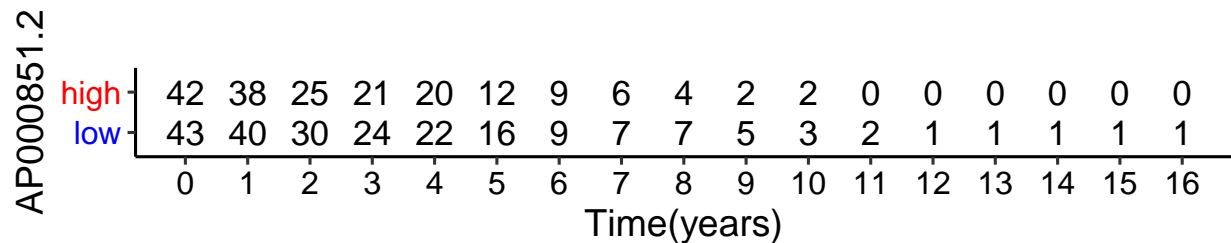

Supplement: Supplementary Document 1 — Kaplan-Meier curve of the 518 genes associated with survival. [file DataSheet_1.zip › Supplementary Document 1/sur.AP000851.2.pdf]

AP000904.1 + high + low

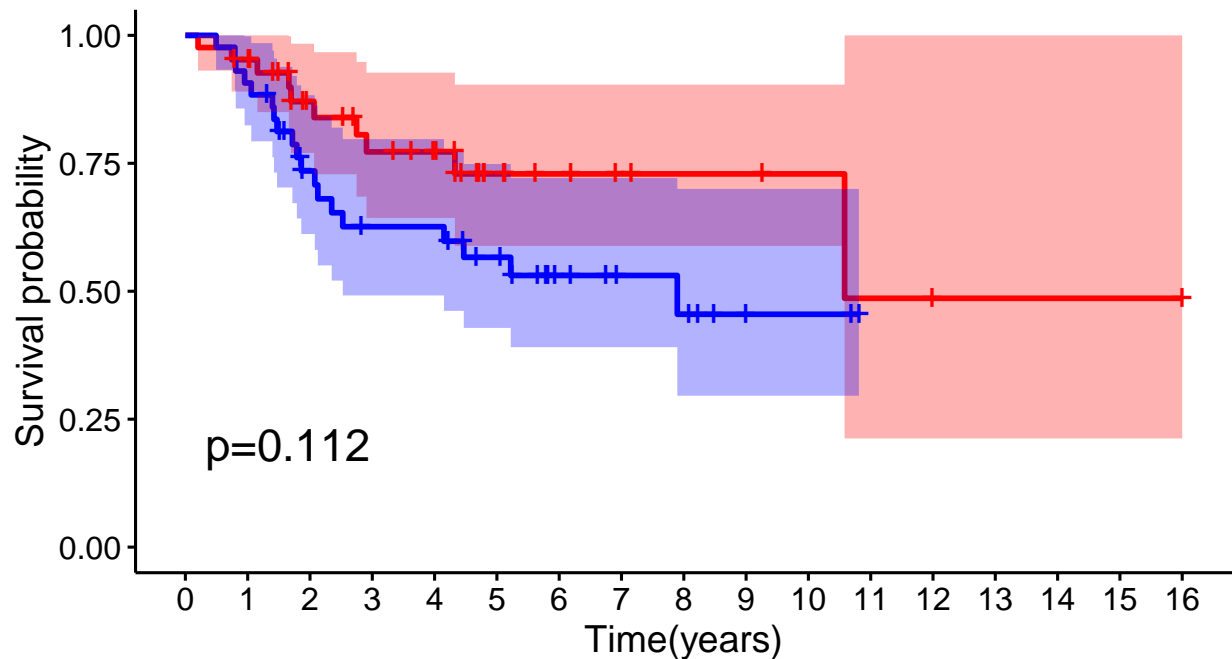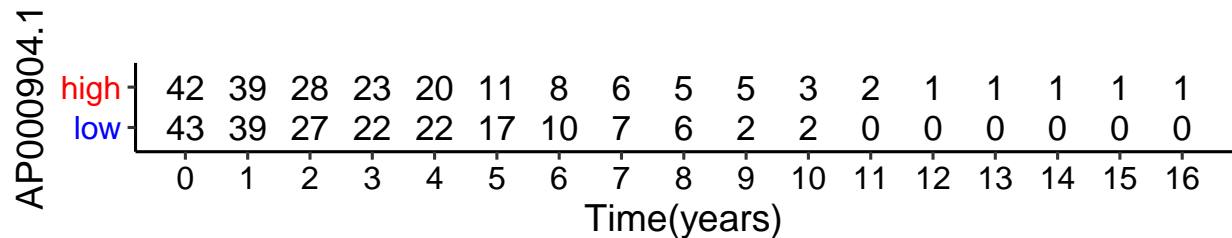

Supplement: Supplementary Document 1 — Kaplan-Meier curve of the 518 genes associated with survival. [file DataSheet_1.zip › Supplementary Document 1/sur.AP000904.1.pdf]

AP001972.4

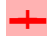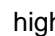

high low

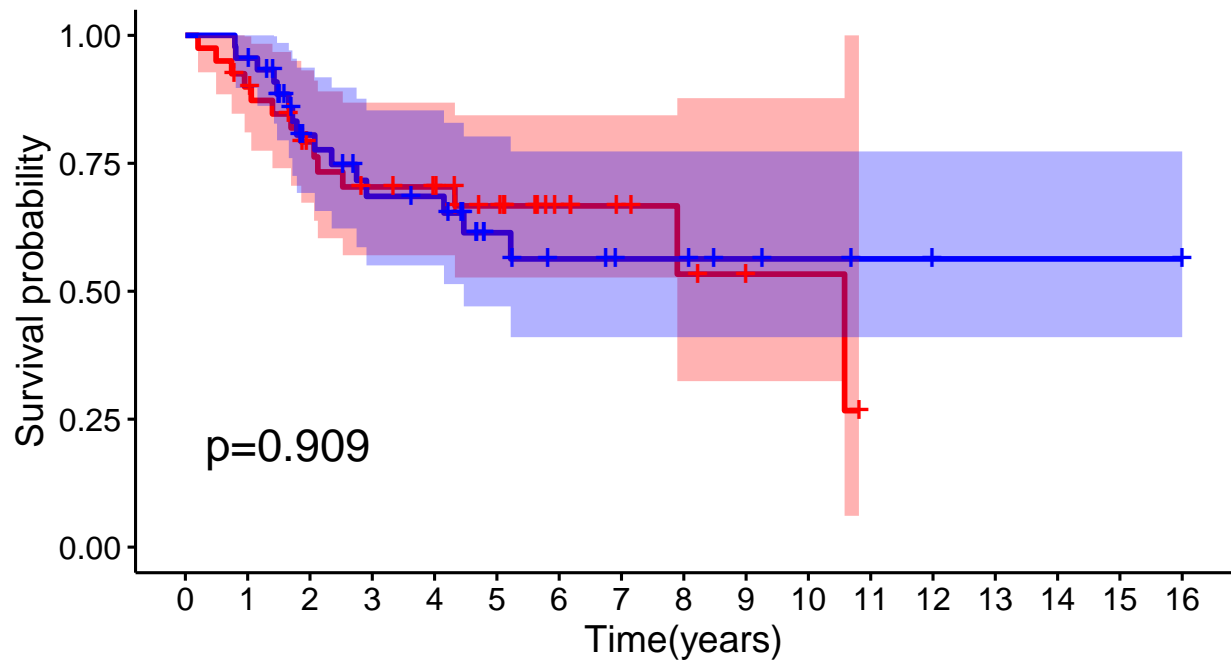

AP001972.4

high

low

|    |    |    |    |    |    |   |   |   |   |    |    |    |    |    |    |
|----|----|----|----|----|----|---|---|---|---|----|----|----|----|----|----|
| 40 | 35 | 27 | 23 | 21 | 16 | 9 | 6 | 4 | 2 | 2  | 0  | 0  | 0  | 0  | 0  |
| 45 | 43 | 28 | 22 | 21 | 12 | 9 | 7 | 7 | 5 | 3  | 2  | 1  | 1  | 1  | 1  |
| 0  | 1  | 2  | 3  | 4  | 5  | 6 | 7 | 8 | 9 | 10 | 11 | 12 | 13 | 14 | 15 |

Time(years)

Supplement: Supplementary Document 1 — Kaplan-Meier curve of the 518 genes associated with survival. [file DataSheet_1.zip › Supplementary Document 1/sur.AP001972.4.pdf]

AP002387.2

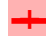

high

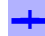

low

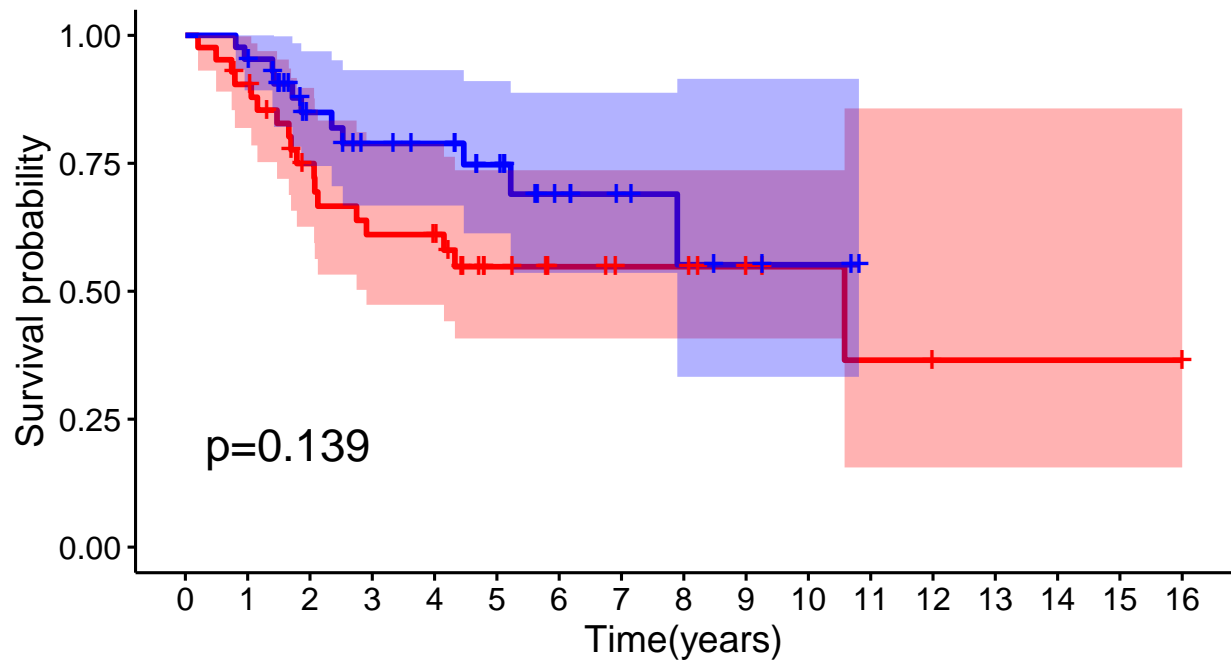

AP002387.2

high

low

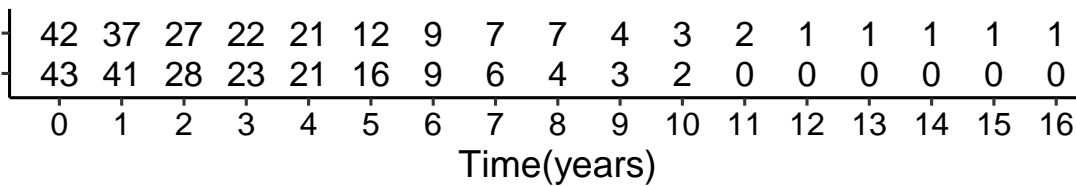

Supplement: Supplementary Document 1 — Kaplan-Meier curve of the 518 genes associated with survival. [file DataSheet_1.zip › Supplementary Document 1/sur.AP002387.2.pdf]

AP002884.1 + high + low

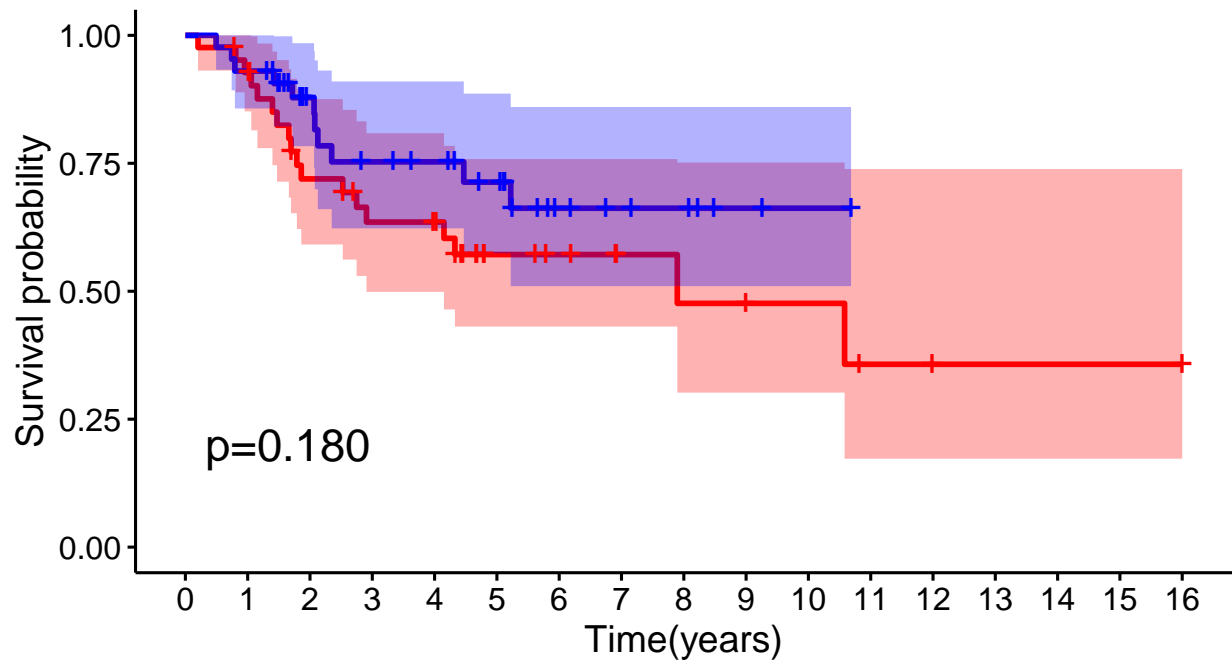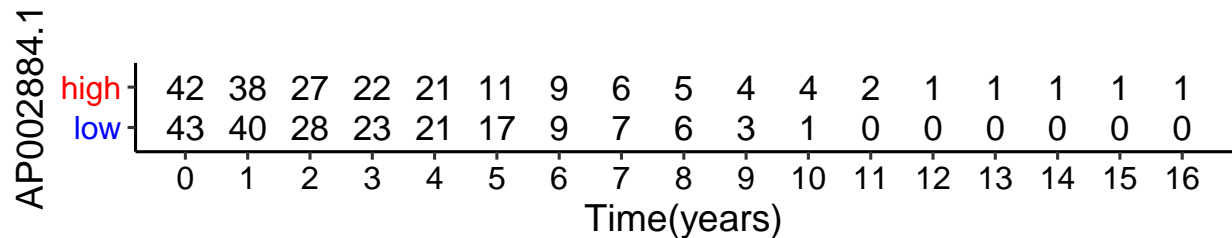

Supplement: Supplementary Document 1 — Kaplan-Meier curve of the 518 genes associated with survival. [file DataSheet_1.zip › Supplementary Document 1/sur.AP002884.1.pdf]

AP002989.1 + high + low

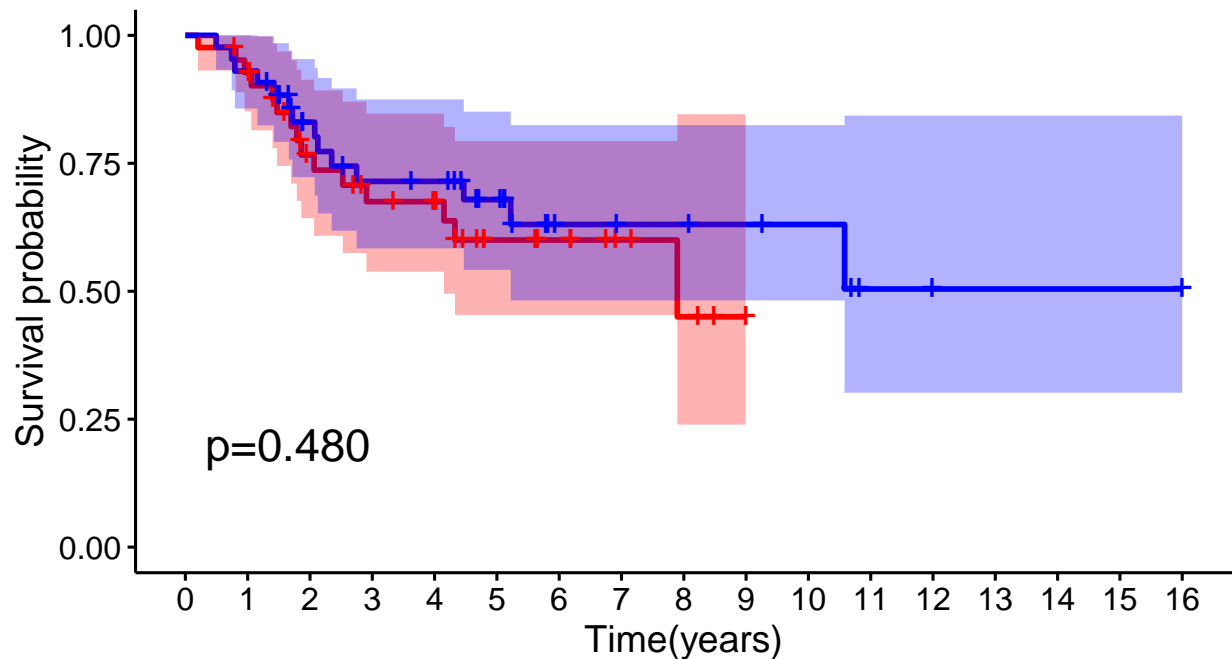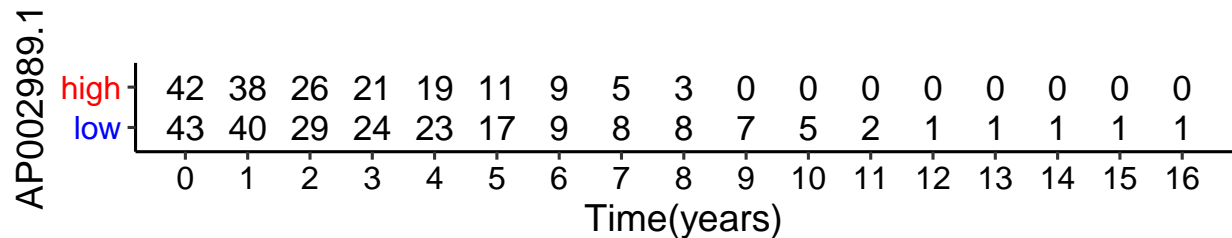

Supplement: Supplementary Document 1 — Kaplan-Meier curve of the 518 genes associated with survival. [file DataSheet_1.zip › Supplementary Document 1/sur.AP002989.1.pdf]

AP003063.1 + high + low

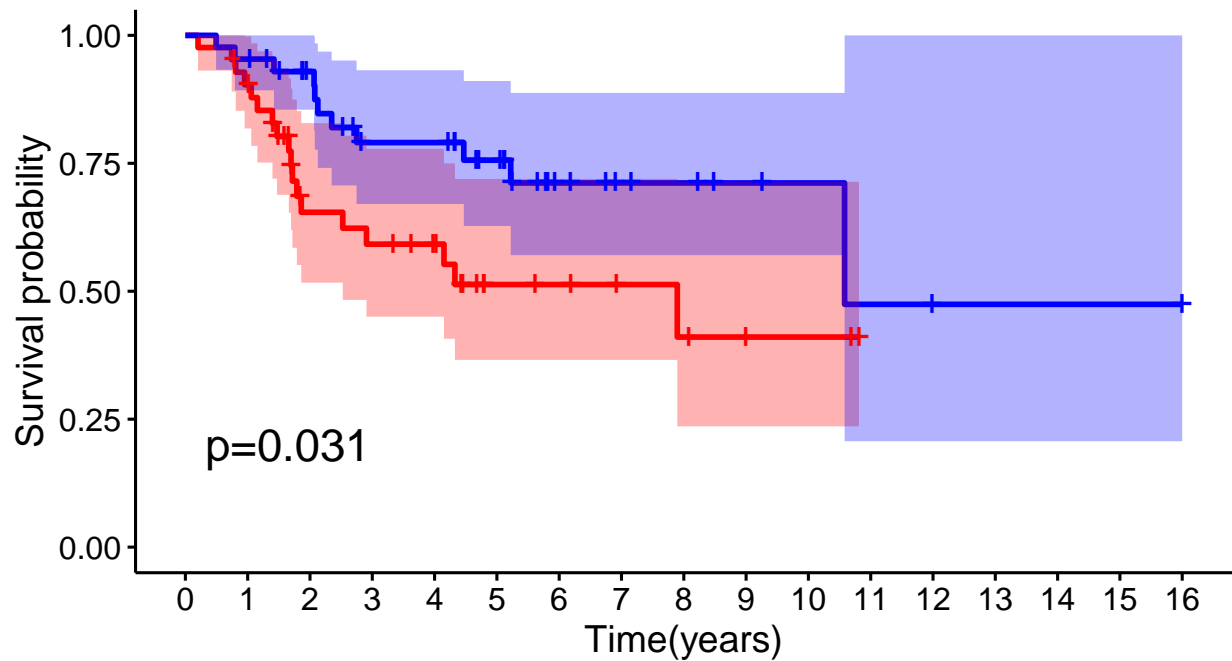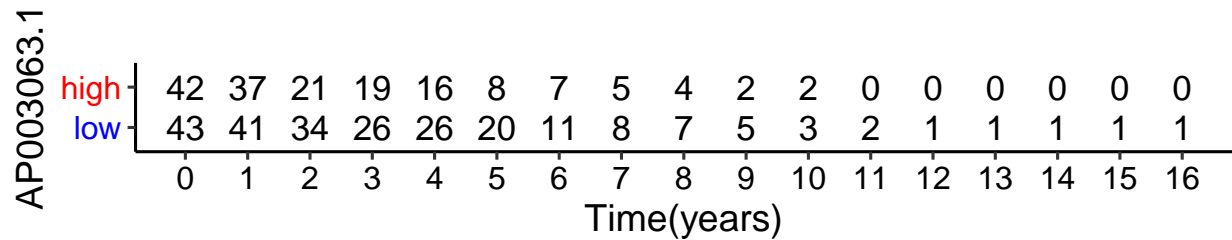

Supplement: Supplementary Document 1 — Kaplan-Meier curve of the 518 genes associated with survival. [file DataSheet_1.zip › Supplementary Document 1/sur.AP003063.1.pdf]

AP003063.2

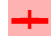

high

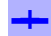

low

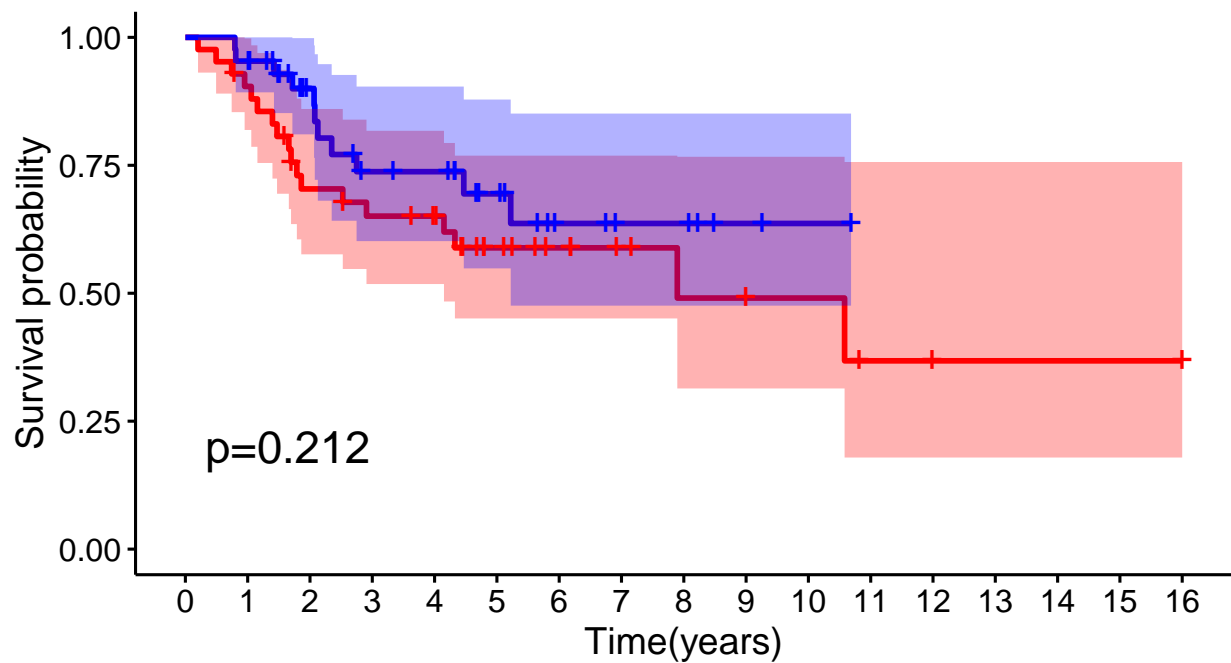

AP003063.2

high

low

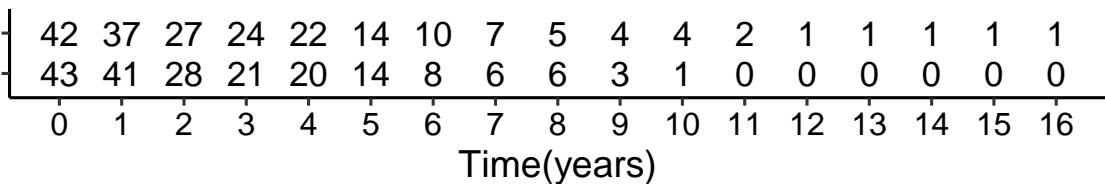

Supplement: Supplementary Document 1 — Kaplan-Meier curve of the 518 genes associated with survival. [file DataSheet_1.zip › Supplementary Document 1/sur.AP003063.2.pdf]

AP003174.1

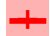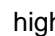

high low

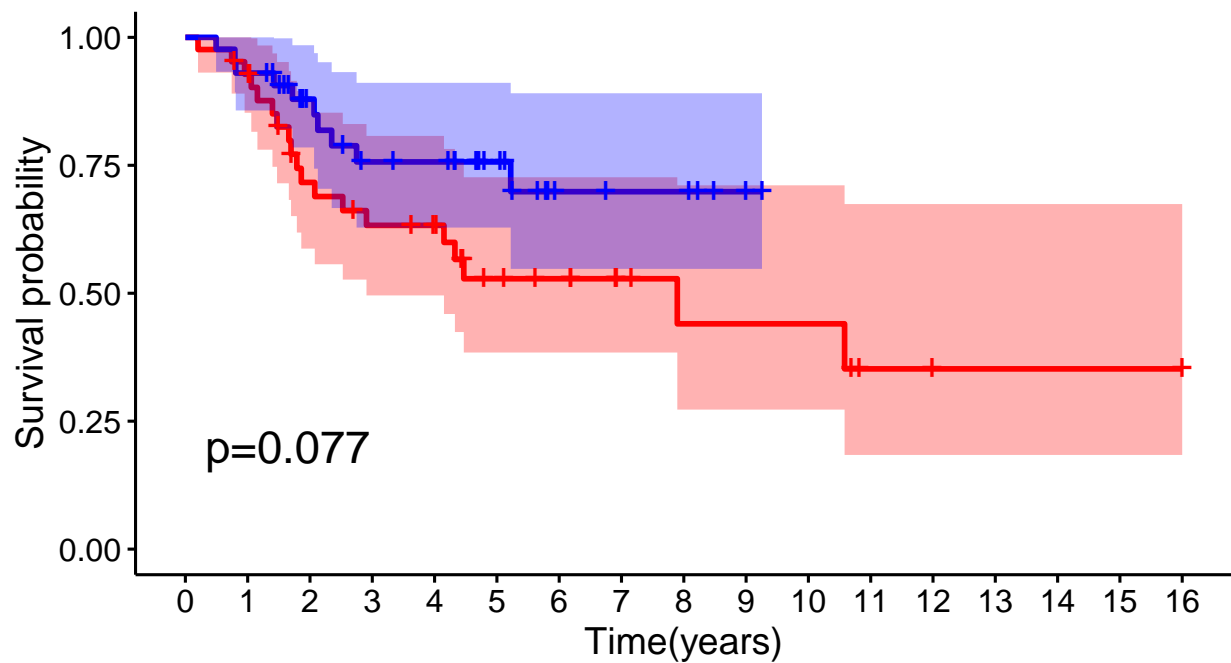

AP003174.1

high

low

|    |    |    |    |    |    |    |   |   |   |    |    |    |    |    |    |    |
|----|----|----|----|----|----|----|---|---|---|----|----|----|----|----|----|----|
| 42 | 38 | 26 | 22 | 20 | 13 | 11 | 7 | 5 | 5 | 5  | 2  | 1  | 1  | 1  | 1  | 1  |
| 43 | 40 | 29 | 23 | 22 | 15 | 7  | 6 | 6 | 2 | 0  | 0  | 0  | 0  | 0  | 0  | 0  |
| 0  | 1  | 2  | 3  | 4  | 5  | 6  | 7 | 8 | 9 | 10 | 11 | 12 | 13 | 14 | 15 | 16 |

Time(years)

Supplement: Supplementary Document 1 — Kaplan-Meier curve of the 518 genes associated with survival. [file DataSheet_1.zip › Supplementary Document 1/sur.AP003174.1.pdf]

AP003469.2

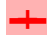

high

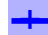

low

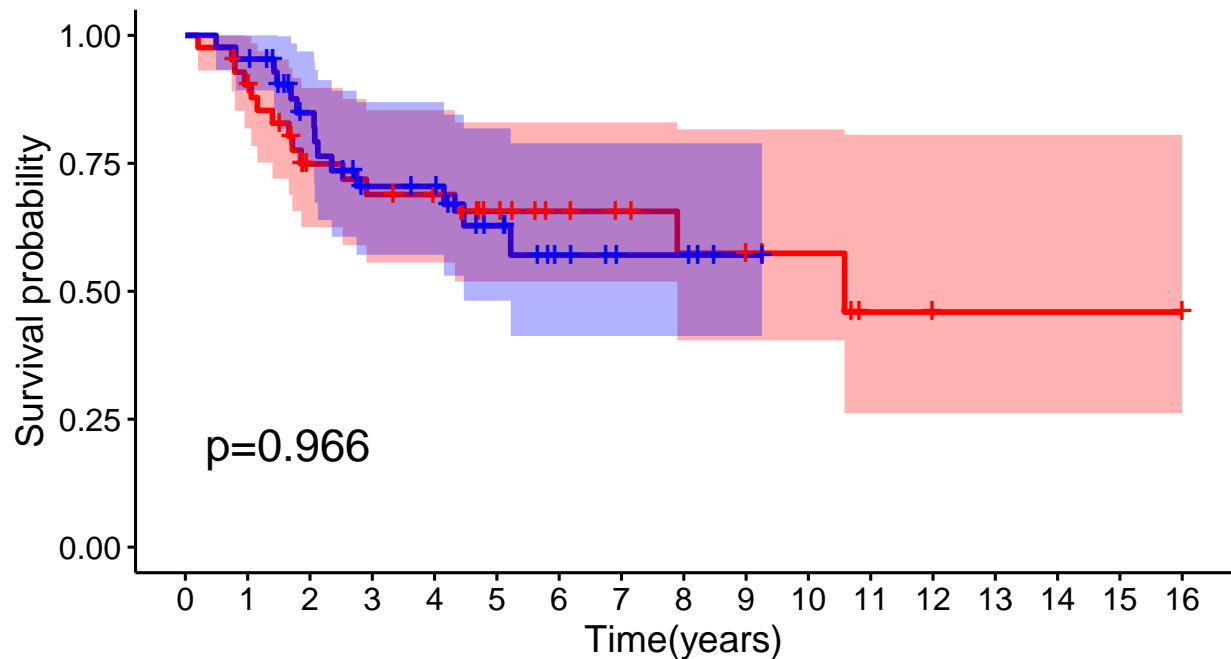

AP003469.2

high

low

|    |    |    |    |    |    |    |   |   |   |    |    |    |    |    |    |
|----|----|----|----|----|----|----|---|---|---|----|----|----|----|----|----|
| 42 | 37 | 25 | 23 | 21 | 15 | 11 | 9 | 7 | 6 | 5  | 2  | 1  | 1  | 1  | 1  |
| 43 | 41 | 30 | 22 | 21 | 13 | 7  | 4 | 4 | 1 | 0  | 0  | 0  | 0  | 0  | 0  |
| 0  | 1  | 2  | 3  | 4  | 5  | 6  | 7 | 8 | 9 | 10 | 11 | 12 | 13 | 14 | 15 |

Time(years)

Supplement: Supplementary Document 1 — Kaplan-Meier curve of the 518 genes associated with survival. [file DataSheet_1.zip › Supplementary Document 1/sur.AP003469.2.pdf]
