# Supplementary figures and images for: Single-Cell Transcriptomics Reveals the Complexity of the Tumor Microenvironment of Treatment-Naive Osteosarcoma (part 2 of 3)
Source: Front Oncol. 2021 Jul 21;11:709210. doi: 10.3389/fonc.2021.709210 (PMC8335545; doi:10.3389/fonc.2021.709210)

ARL15 + high + low

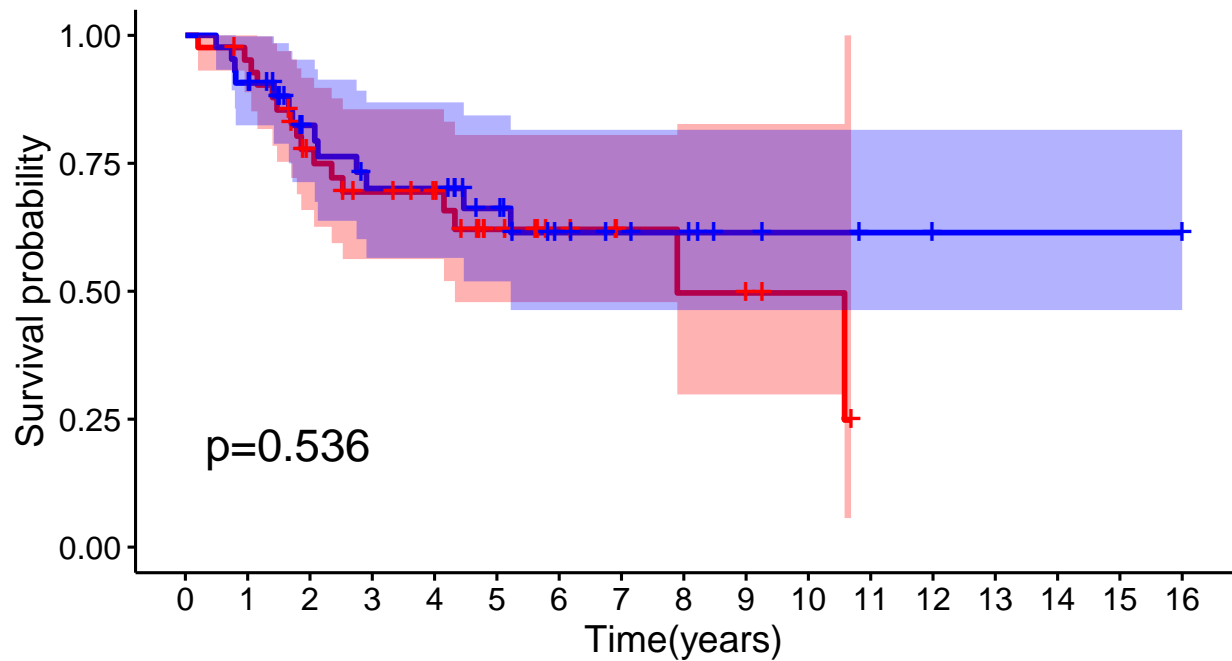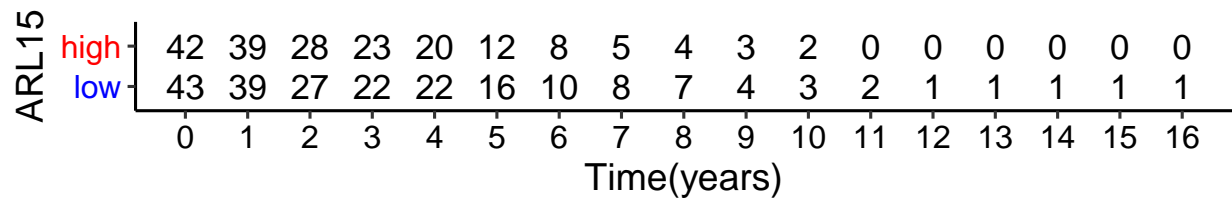

Supplement: Supplementary Document 1 — Kaplan-Meier curve of the 518 genes associated with survival. [file DataSheet_1.zip › Supplementary Document 1/sur.ARL15.pdf]

ARRDC4 + high + low

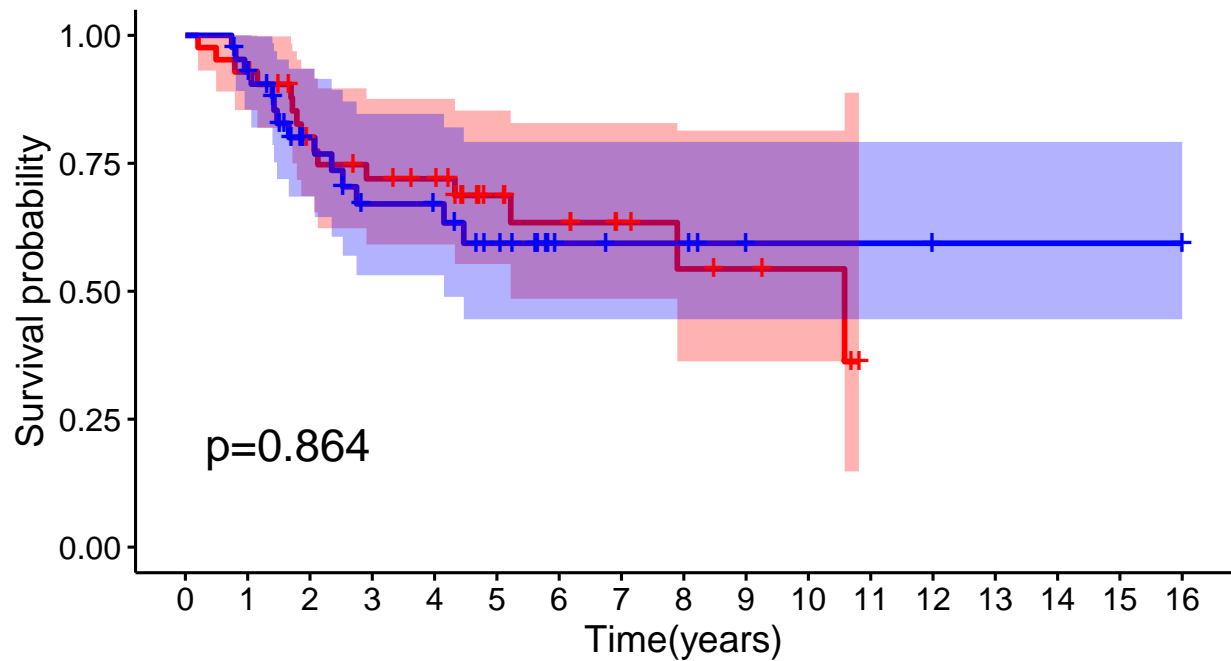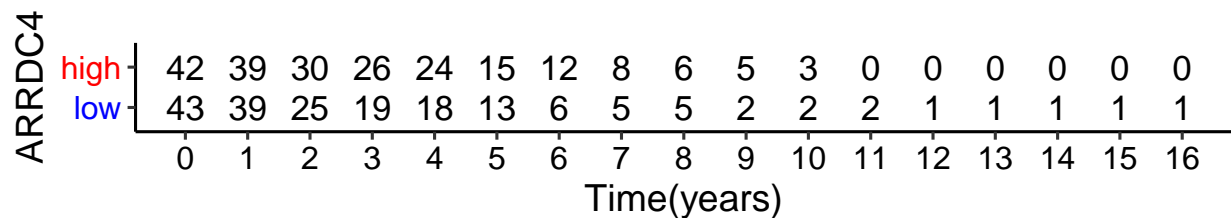

Supplement: Supplementary Document 1 — Kaplan-Meier curve of the 518 genes associated with survival. [file DataSheet_1.zip › Supplementary Document 1/sur.ARRDC4.pdf]

ATAD2 high low

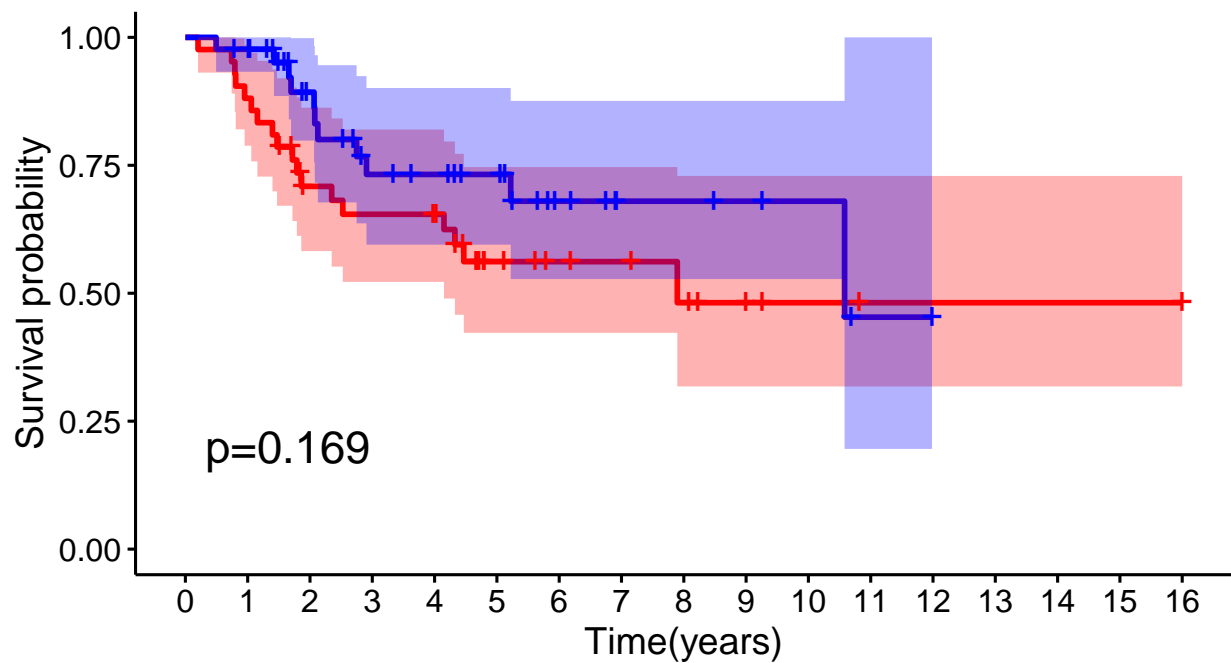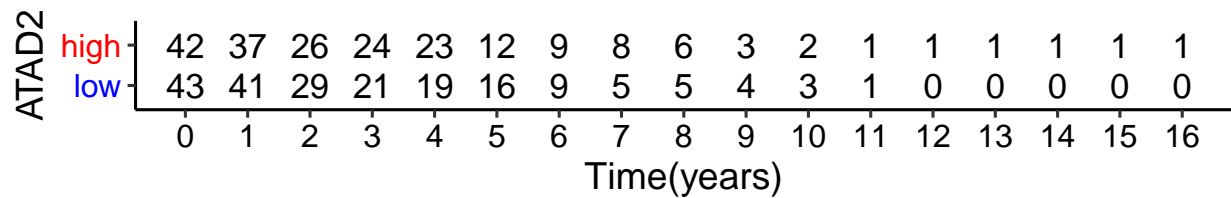

Supplement: Supplementary Document 1 — Kaplan-Meier curve of the 518 genes associated with survival. [file DataSheet_1.zip › Supplementary Document 1/sur.ATAD2.pdf]

ATP11C + high + low

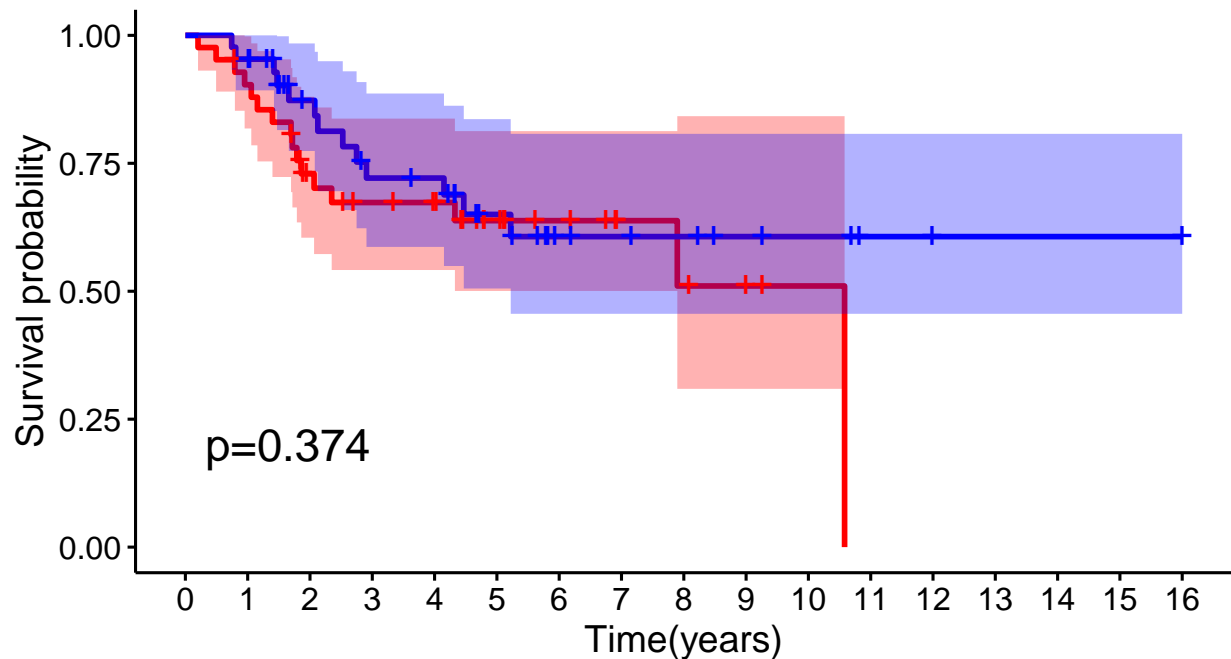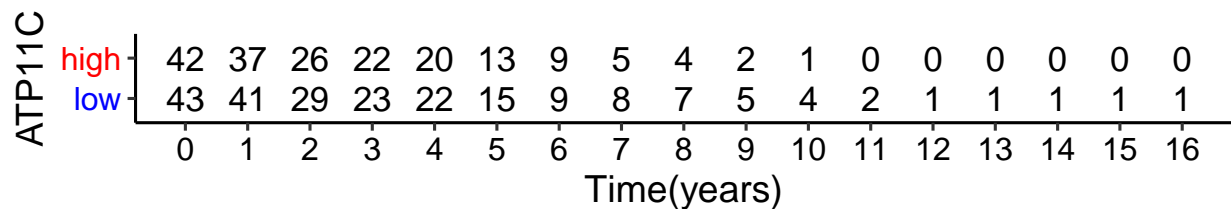

Supplement: Supplementary Document 1 — Kaplan-Meier curve of the 518 genes associated with survival. [file DataSheet_1.zip › Supplementary Document 1/sur.ATP11C.pdf]

ATP6V1B2 + high + low

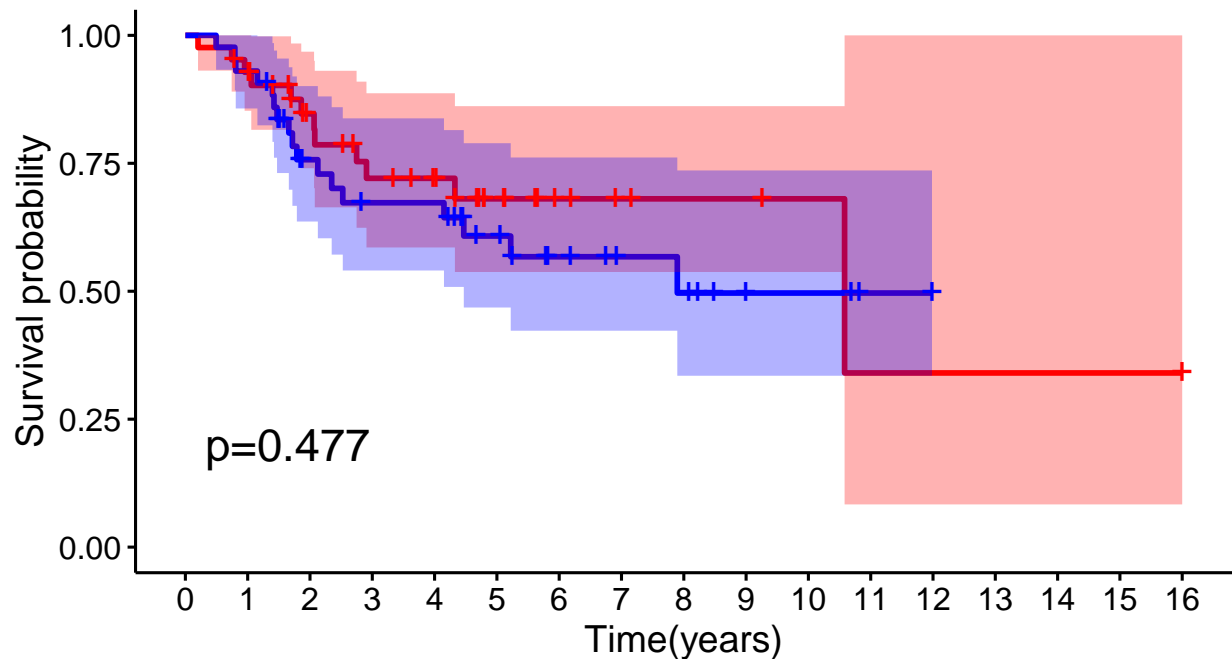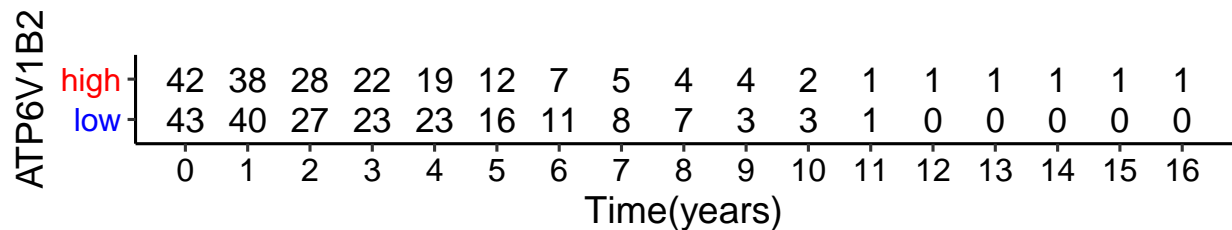

Supplement: Supplementary Document 1 — Kaplan-Meier curve of the 518 genes associated with survival. [file DataSheet_1.zip › Supplementary Document 1/sur.ATP6V1B2.pdf]

ATP6V1C1 + high + low

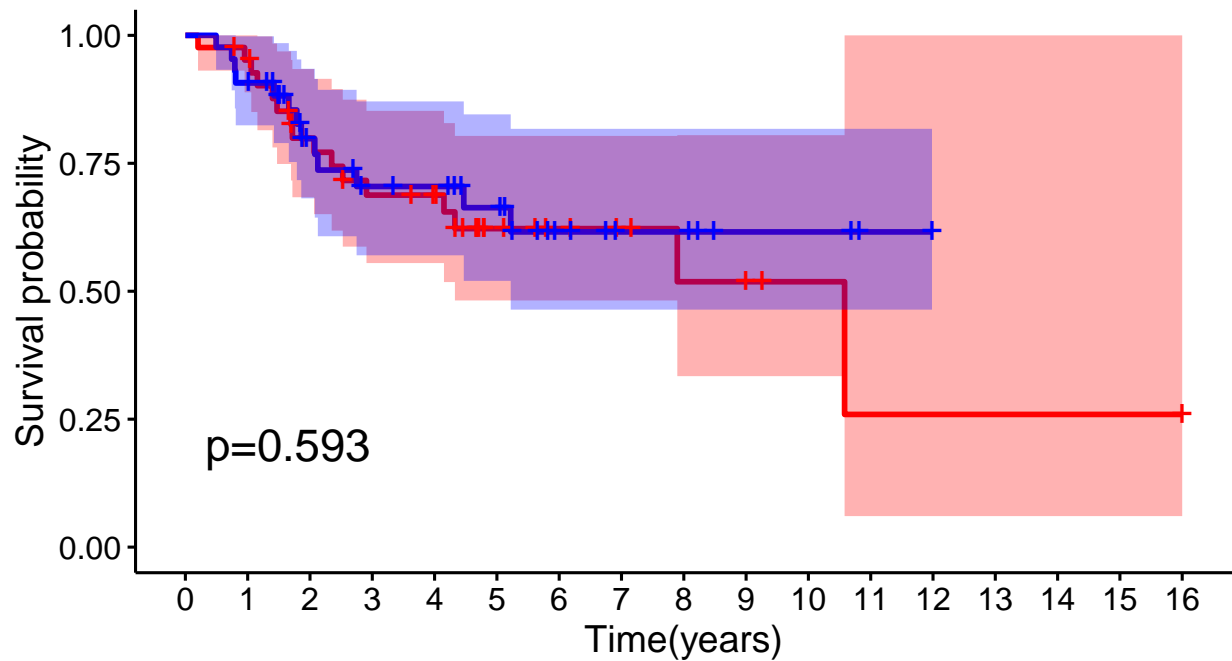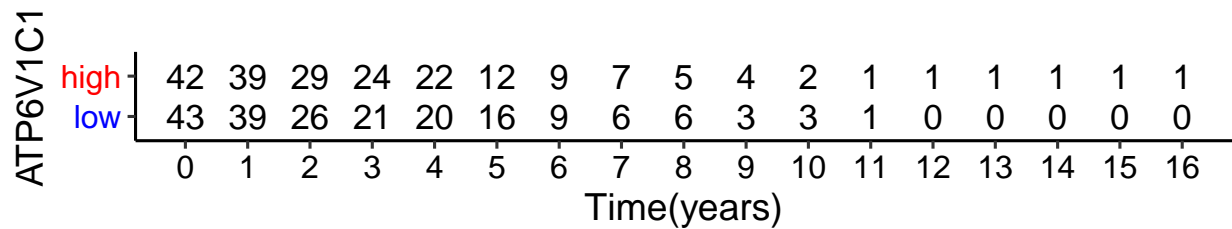

Supplement: Supplementary Document 1 — Kaplan-Meier curve of the 518 genes associated with survival. [file DataSheet_1.zip › Supplementary Document 1/sur.ATP6V1C1.pdf]

ATP8A1 + high + low

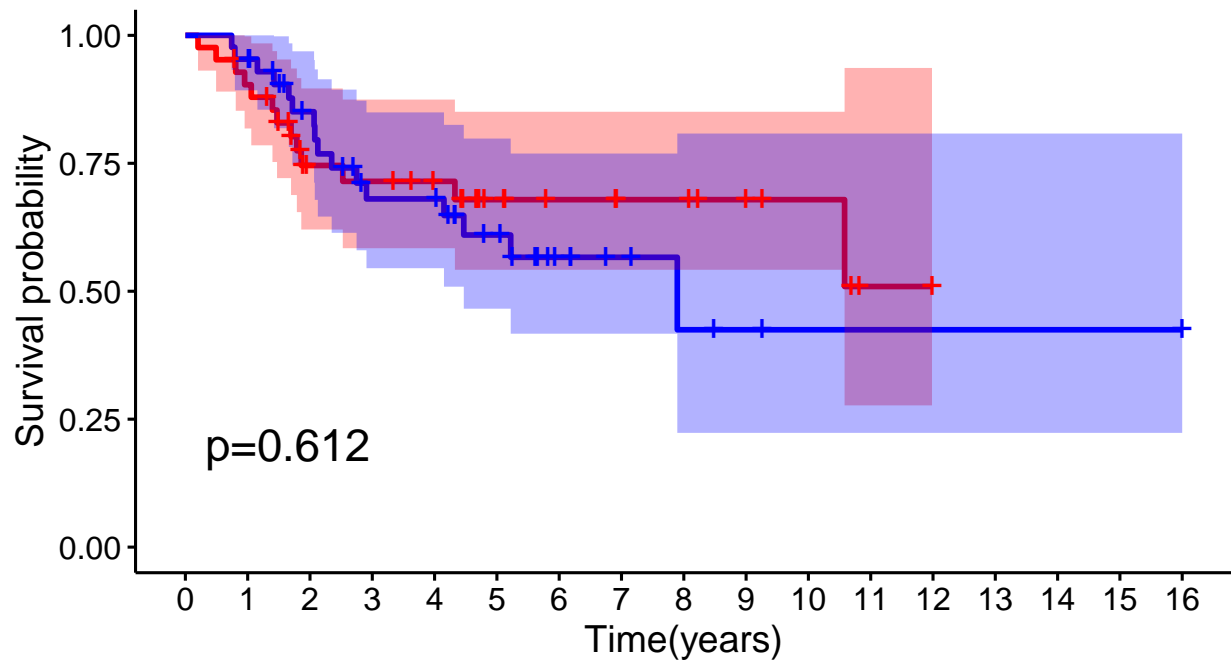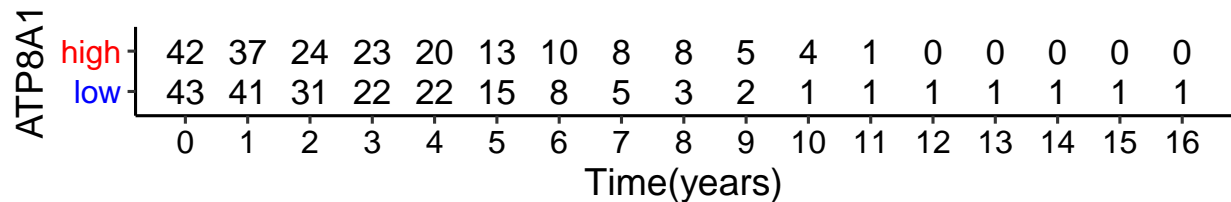

Supplement: Supplementary Document 1 — Kaplan-Meier curve of the 518 genes associated with survival. [file DataSheet_1.zip › Supplementary Document 1/sur.ATP8A1.pdf]

ATP8A2 high low

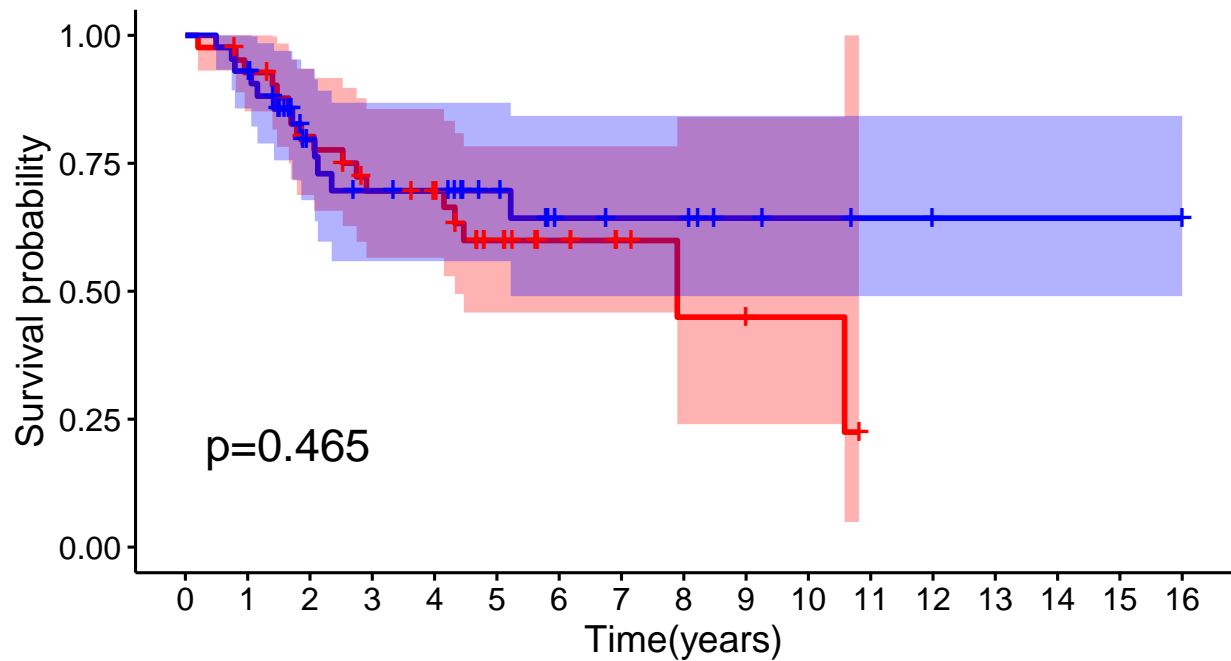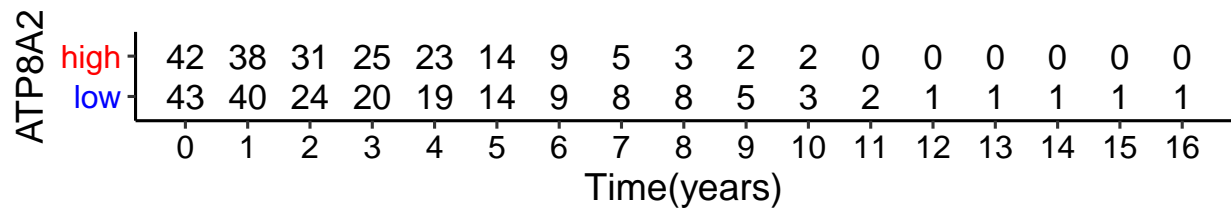

Supplement: Supplementary Document 1 — Kaplan-Meier curve of the 518 genes associated with survival. [file DataSheet_1.zip › Supplementary Document 1/sur.ATP8A2.pdf]

B4GAT1 high low

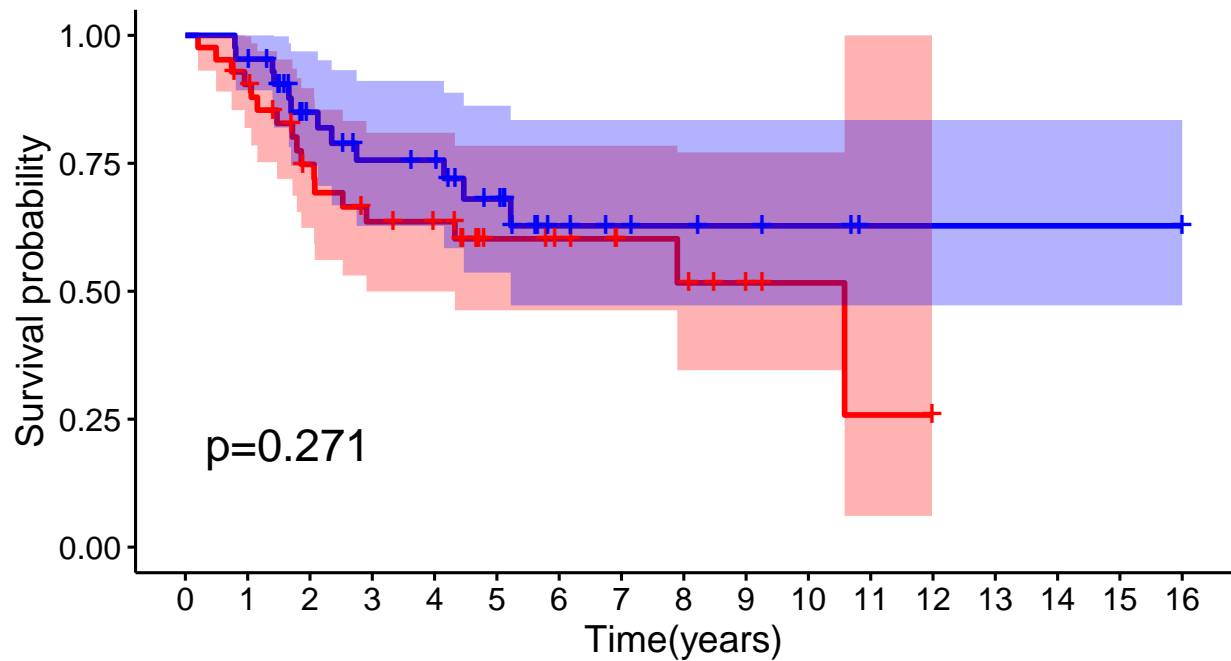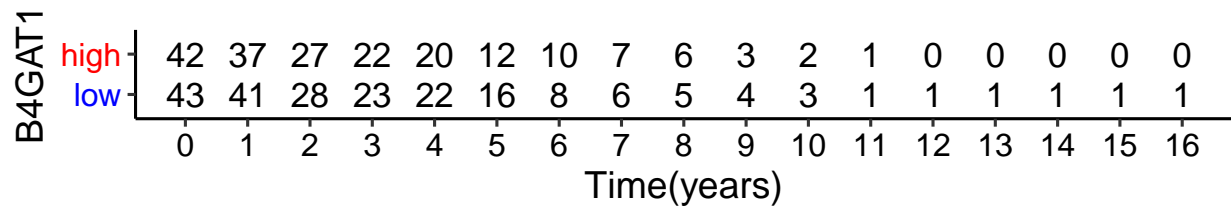

Supplement: Supplementary Document 1 — Kaplan-Meier curve of the 518 genes associated with survival. [file DataSheet_1.zip › Supplementary Document 1/sur.B4GAT1.pdf]

BAIAP2L2 + high + low

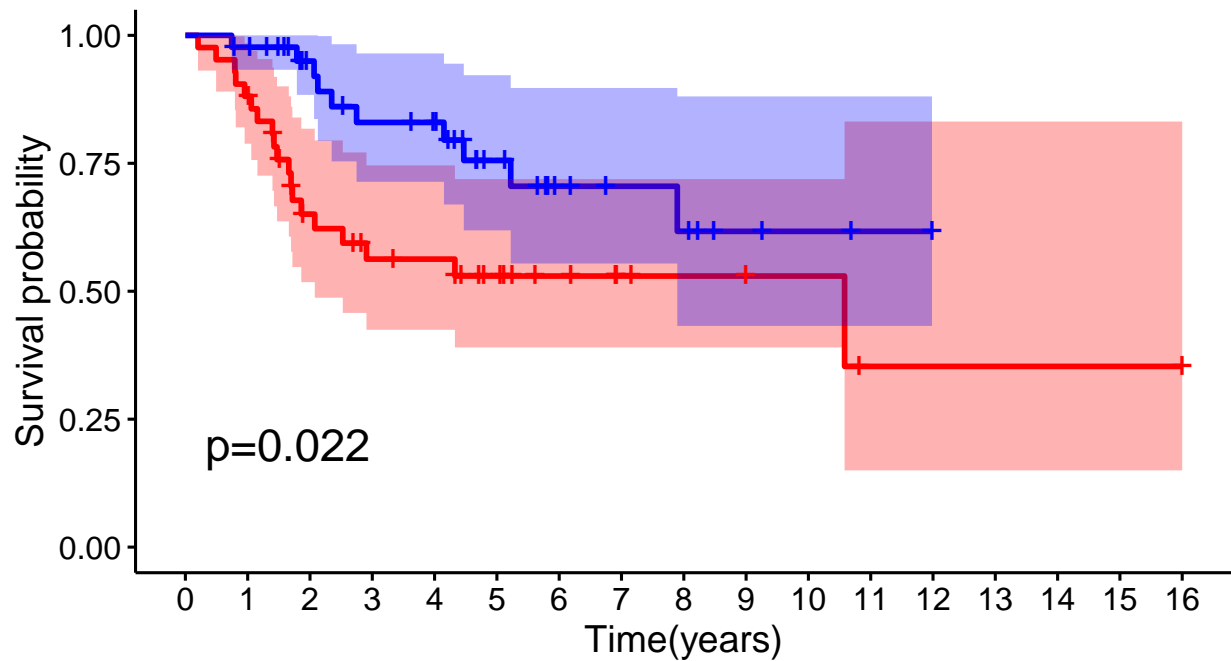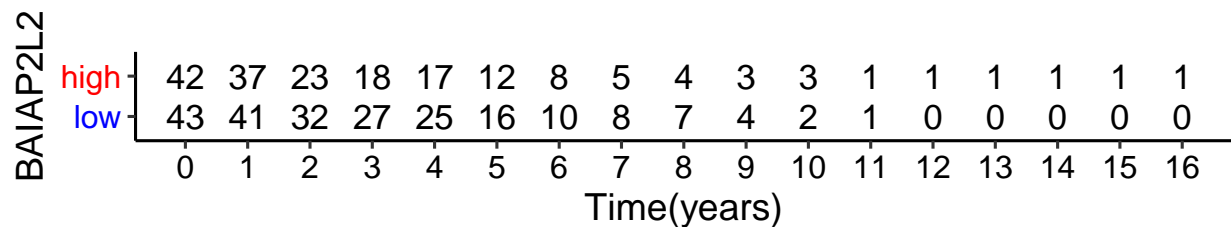

Supplement: Supplementary Document 1 — Kaplan-Meier curve of the 518 genes associated with survival. [file DataSheet_1.zip › Supplementary Document 1/sur.BAIAP2L2.pdf]

BAMBI high low

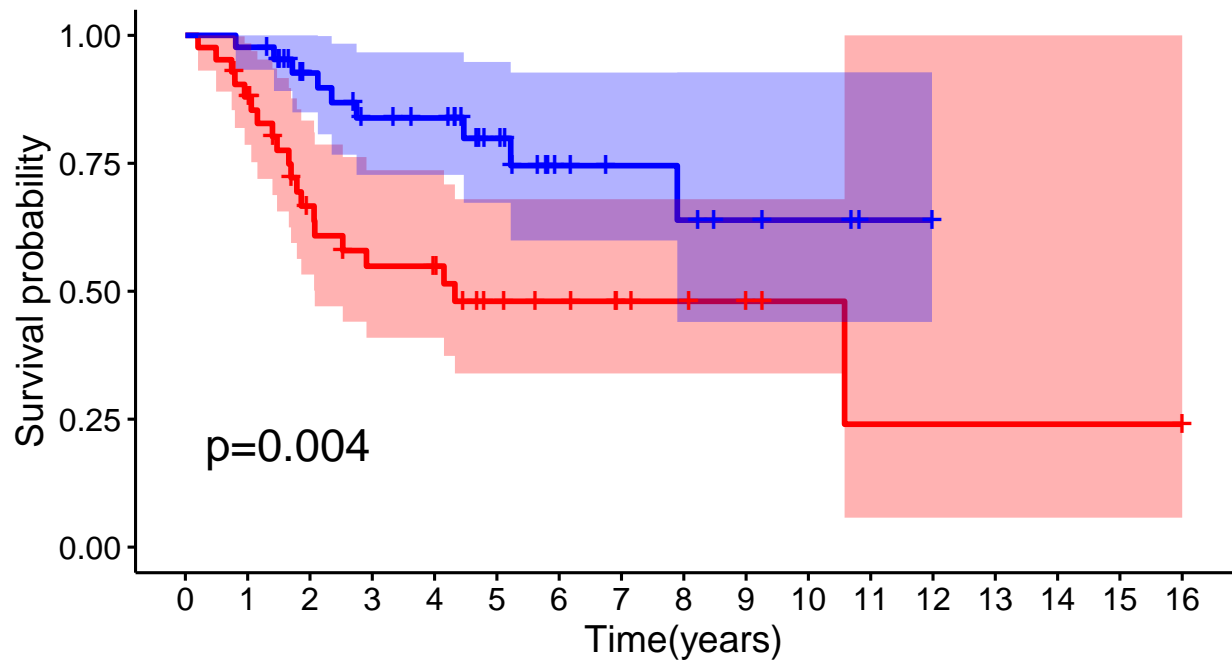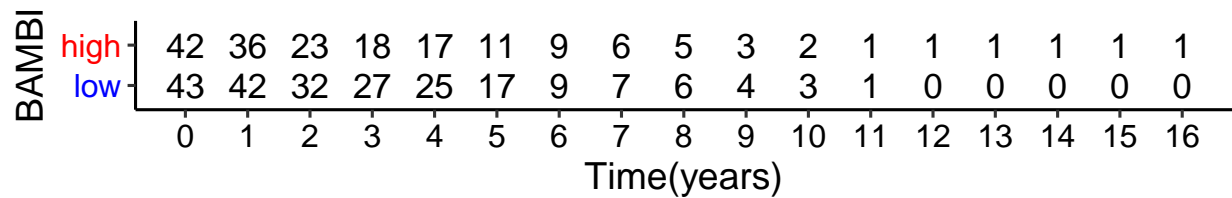

Supplement: Supplementary Document 1 — Kaplan-Meier curve of the 518 genes associated with survival. [file DataSheet_1.zip › Supplementary Document 1/sur.BAMBI.pdf]

BEND2 high low

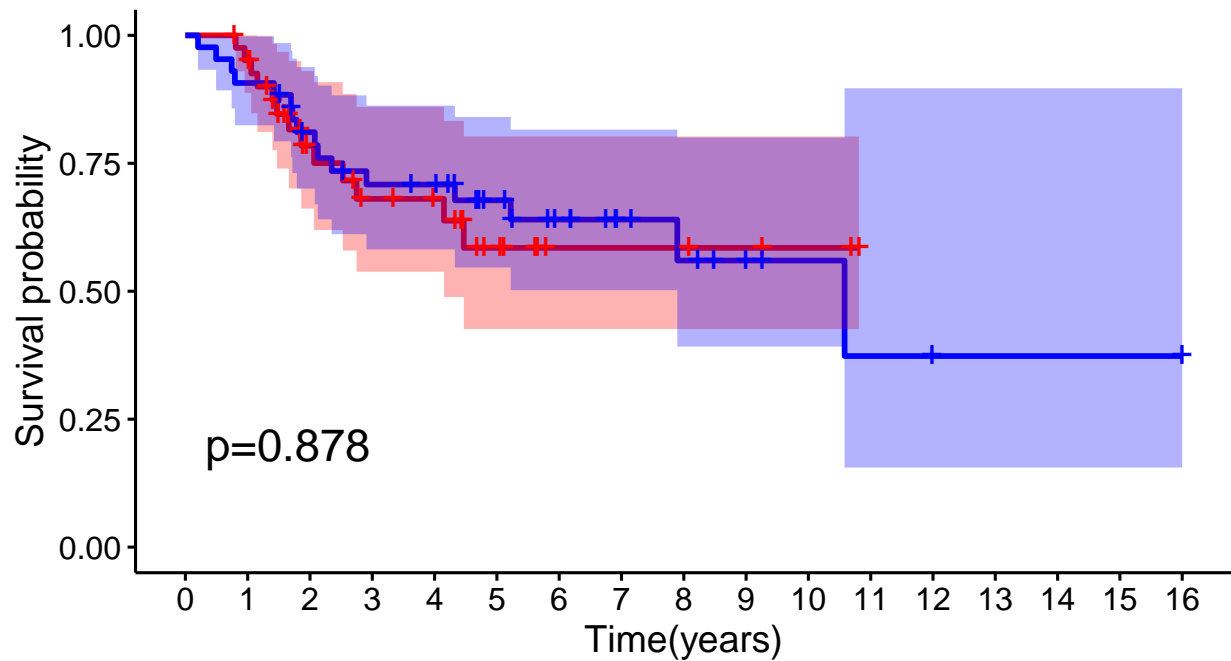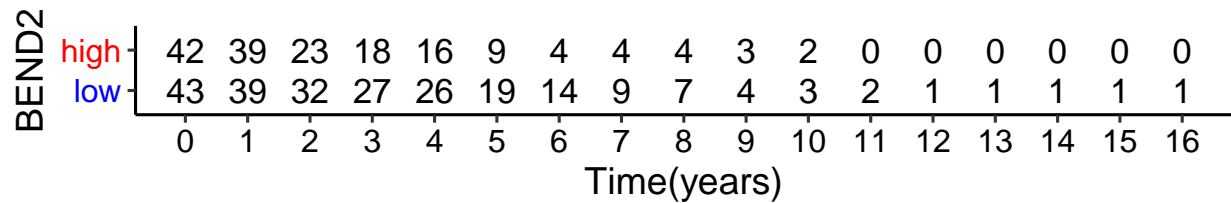

Supplement: Supplementary Document 1 — Kaplan-Meier curve of the 518 genes associated with survival. [file DataSheet_1.zip › Supplementary Document 1/sur.BEND2.pdf]

BMP3 + high + low

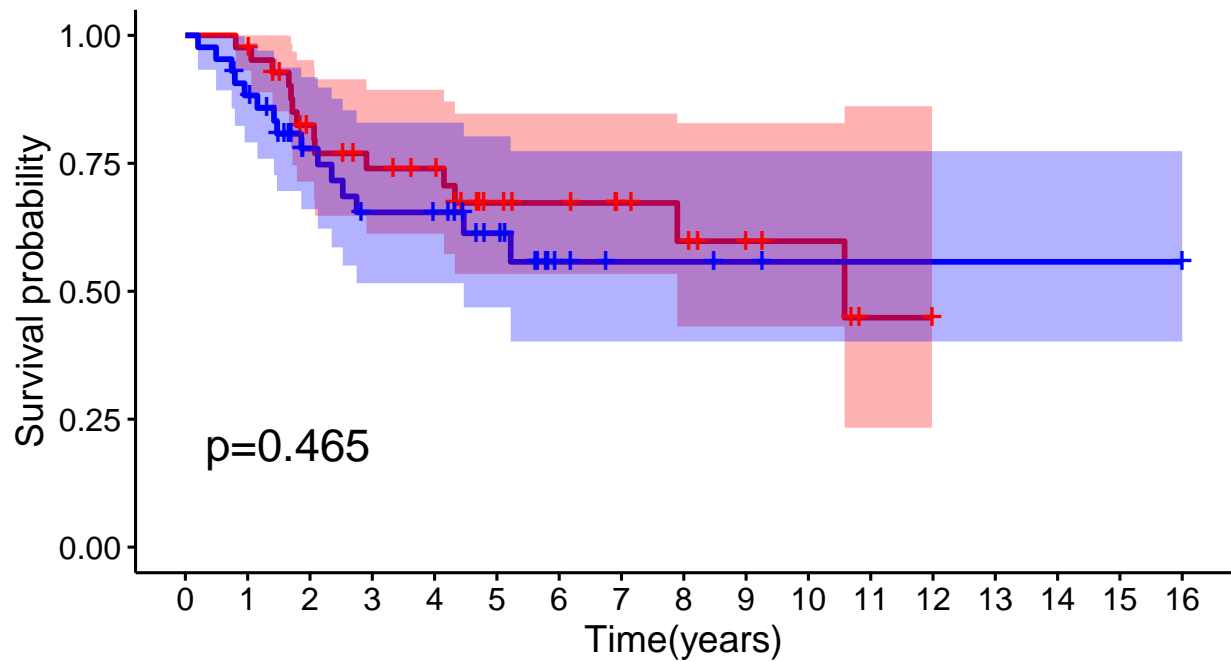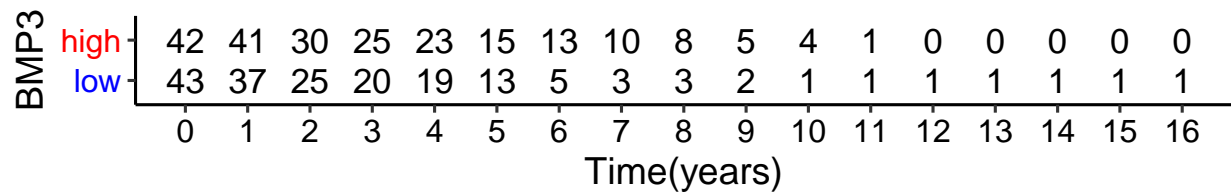

Supplement: Supplementary Document 1 — Kaplan-Meier curve of the 518 genes associated with survival. [file DataSheet_1.zip › Supplementary Document 1/sur.BMP3.pdf]

BMP8A high low

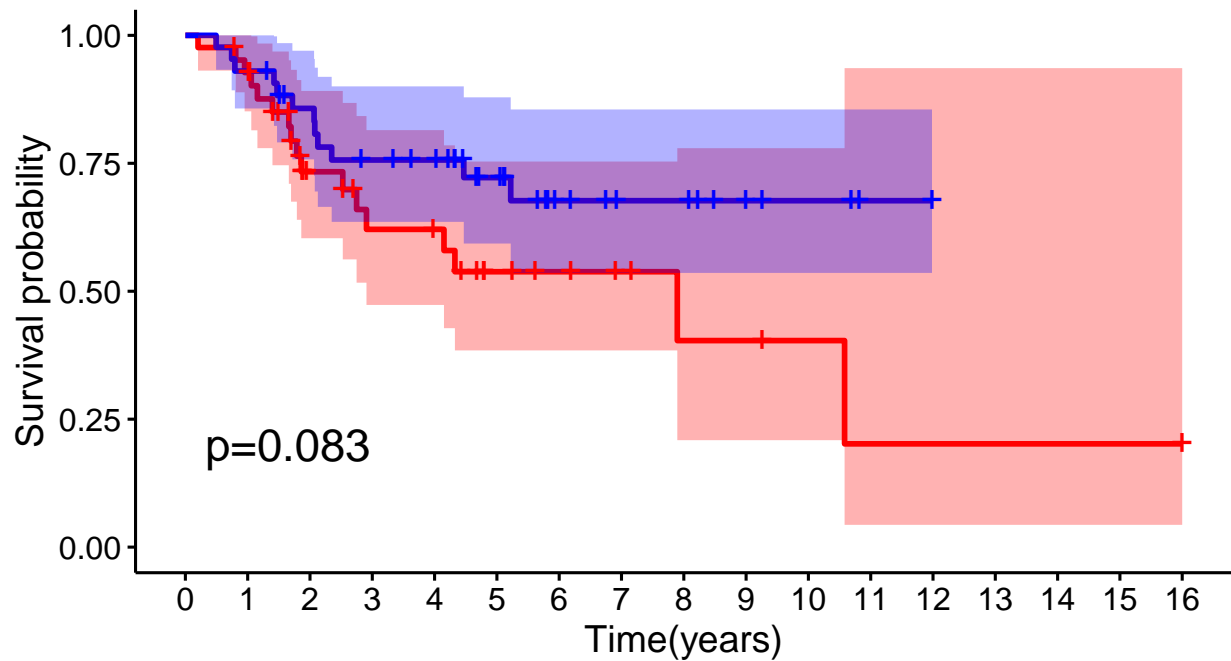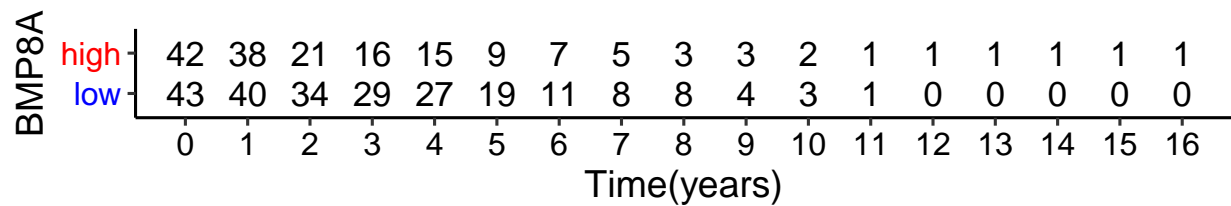

Supplement: Supplementary Document 1 — Kaplan-Meier curve of the 518 genes associated with survival. [file DataSheet_1.zip › Supplementary Document 1/sur.BMP8A.pdf]

BMP8B high low

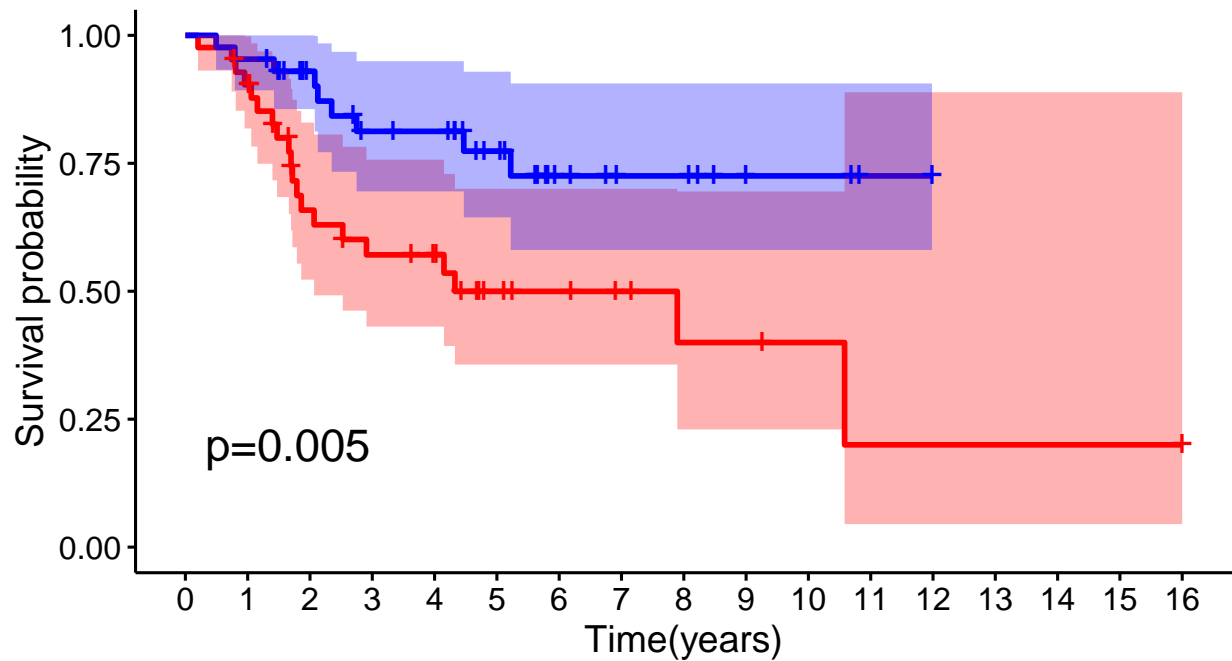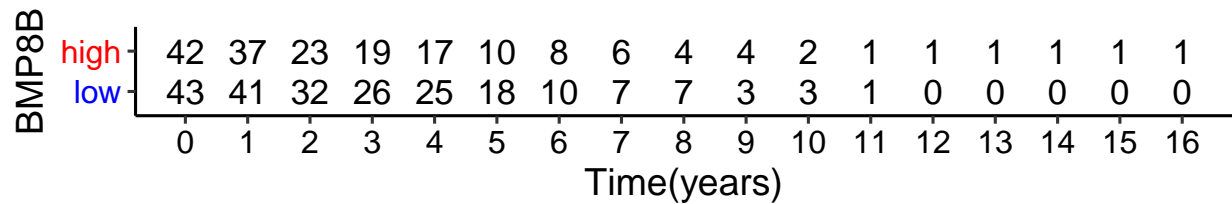

Supplement: Supplementary Document 1 — Kaplan-Meier curve of the 518 genes associated with survival. [file DataSheet_1.zip › Supplementary Document 1/sur.BMP8B.pdf]

BOK + high + low

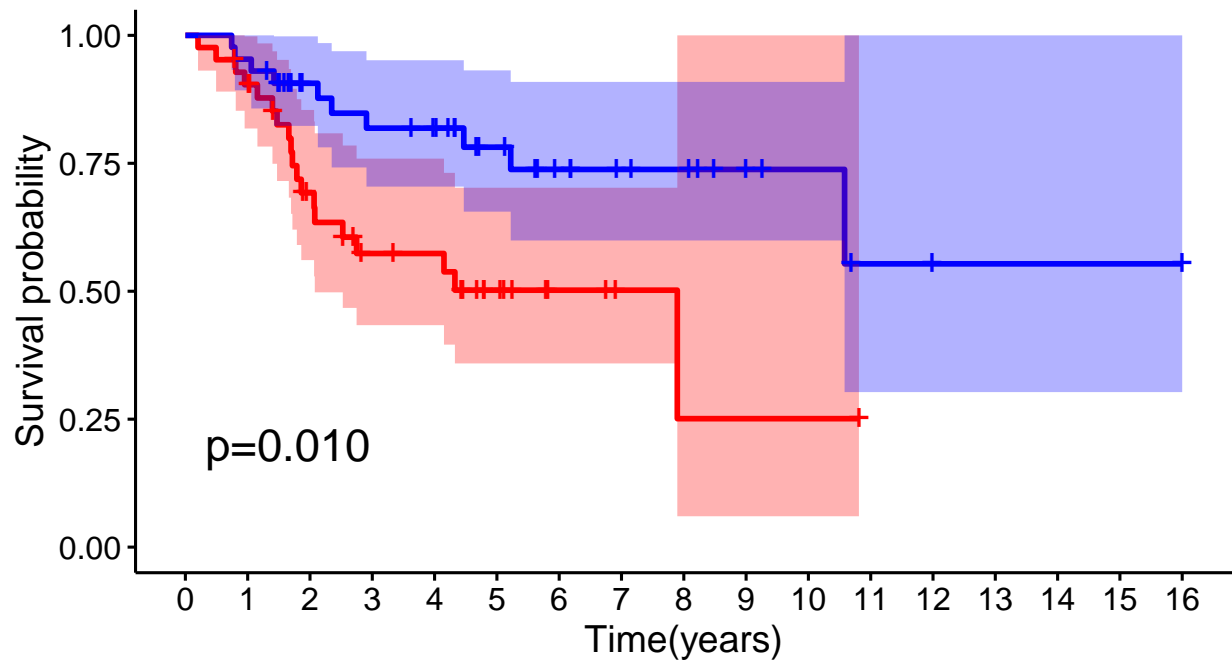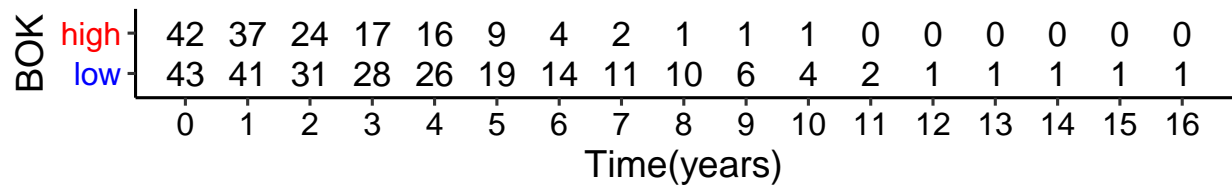

Supplement: Supplementary Document 1 — Kaplan-Meier curve of the 518 genes associated with survival. [file DataSheet_1.zip › Supplementary Document 1/sur.BOK.pdf]

C16orf54 high low

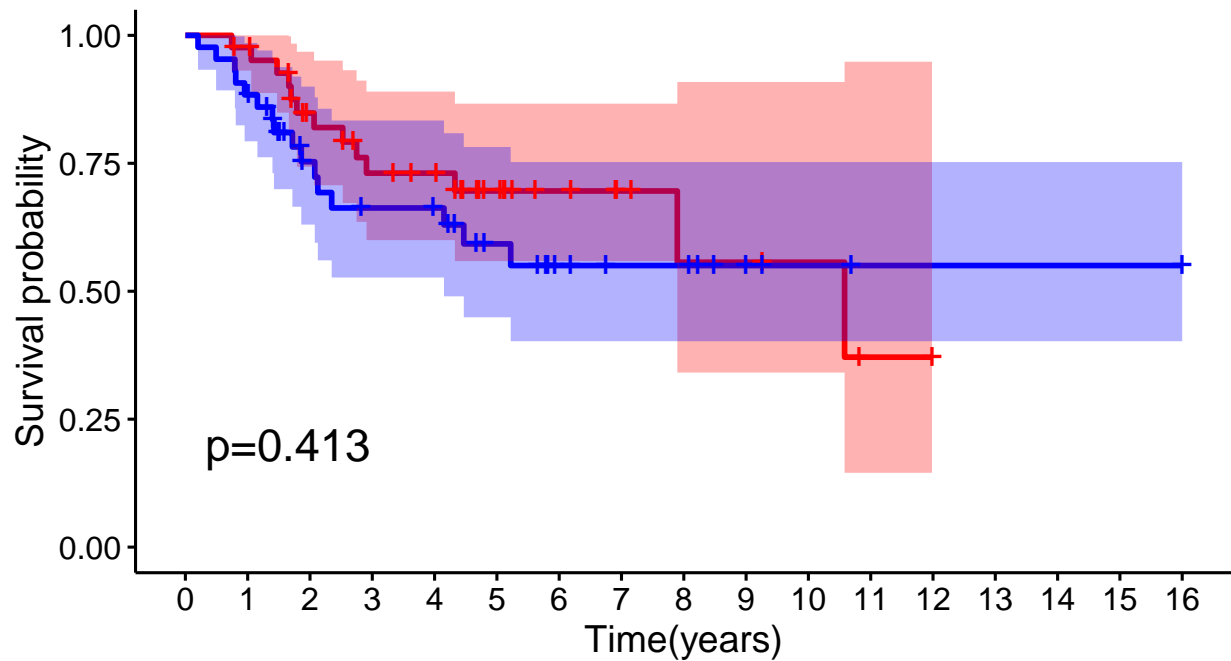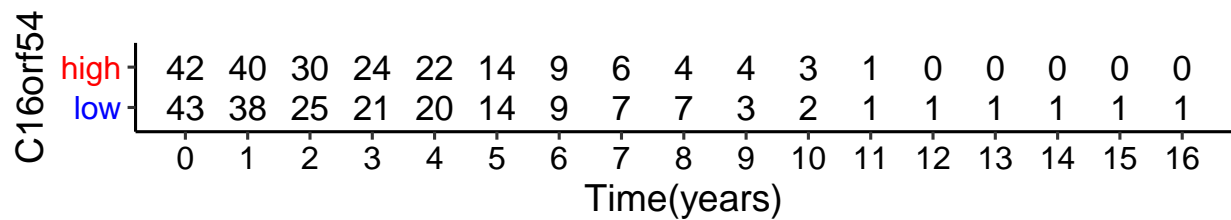

Supplement: Supplementary Document 1 — Kaplan-Meier curve of the 518 genes associated with survival. [file DataSheet_1.zip › Supplementary Document 1/sur.C16orf54.pdf]

C1QTNF1 + high + low

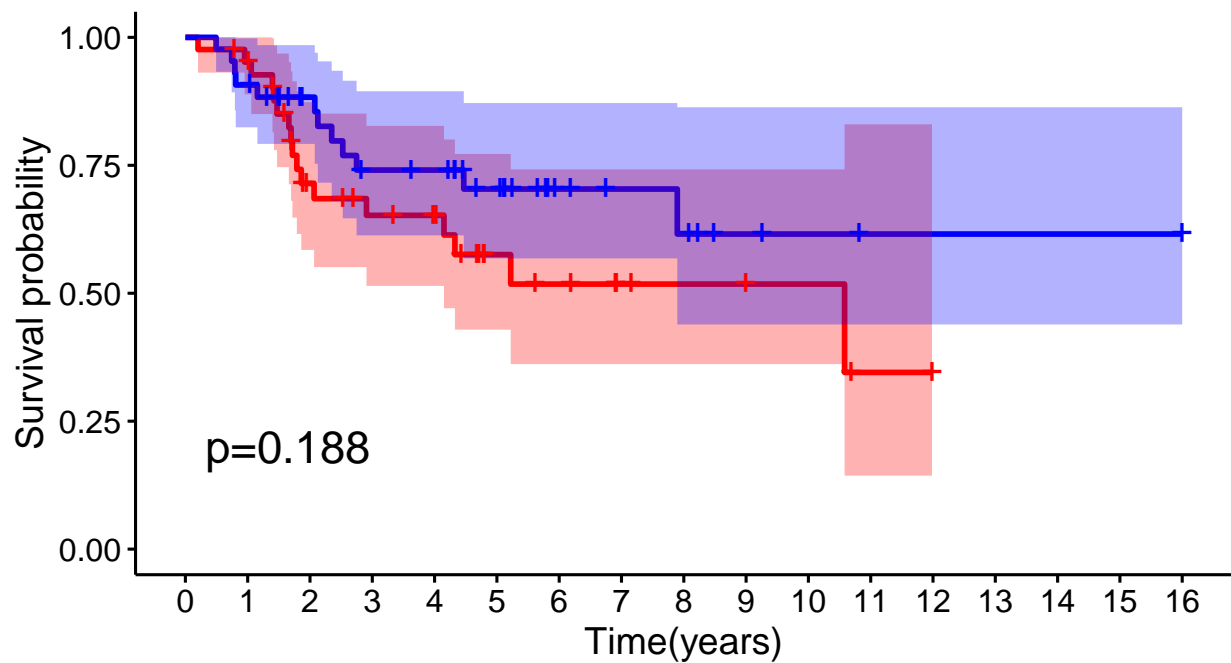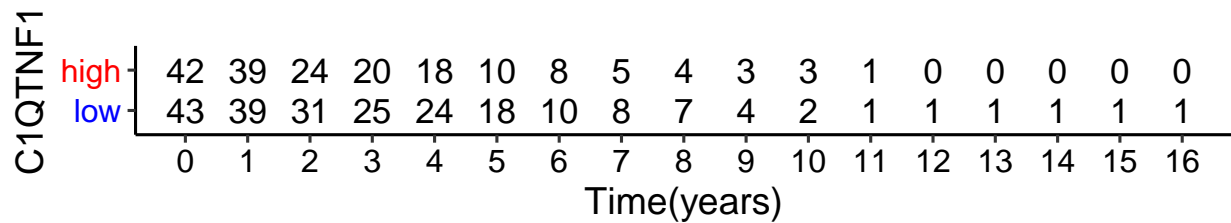

Supplement: Supplementary Document 1 — Kaplan-Meier curve of the 518 genes associated with survival. [file DataSheet_1.zip › Supplementary Document 1/sur.C1QTNF1.pdf]

C5AR2 high low

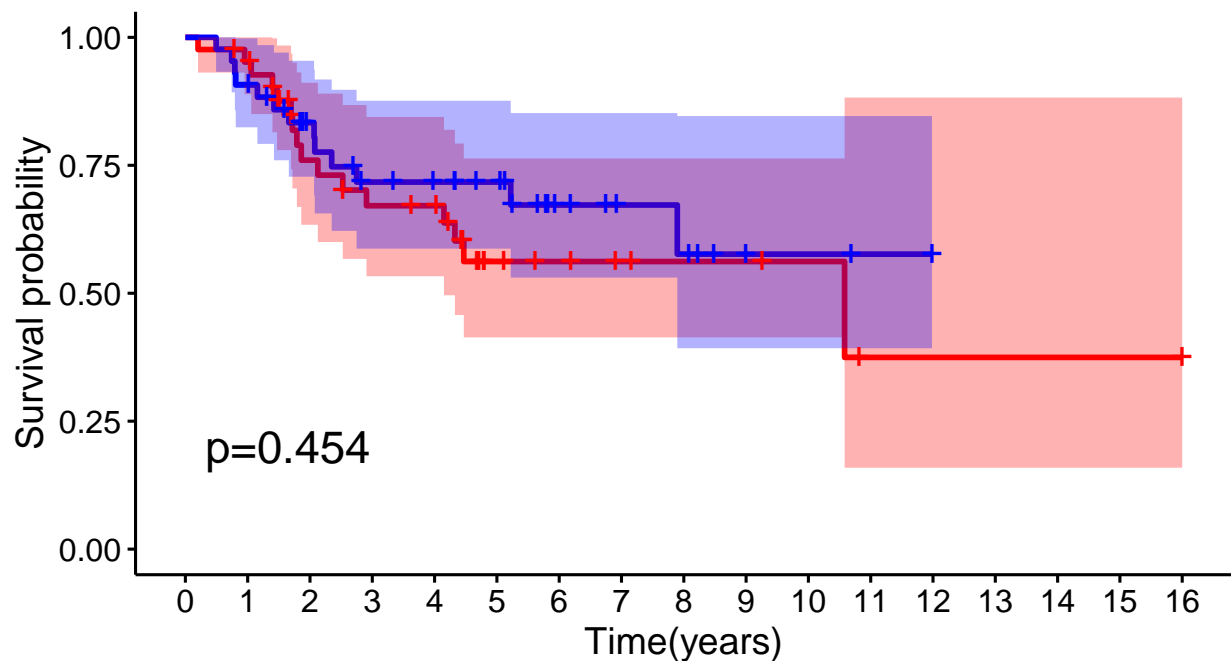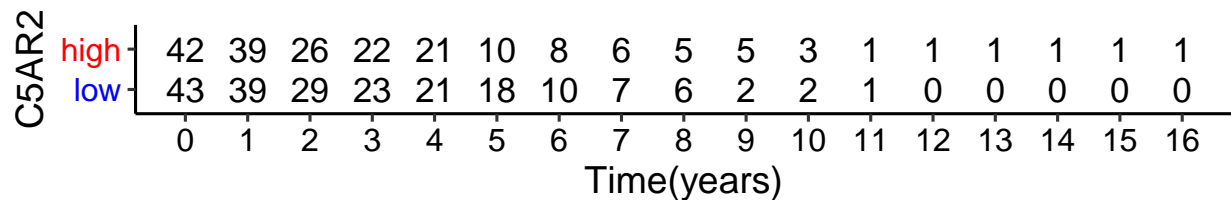

Supplement: Supplementary Document 1 — Kaplan-Meier curve of the 518 genes associated with survival. [file DataSheet_1.zip › Supplementary Document 1/sur.C5AR2.pdf]

CA2 + high + low

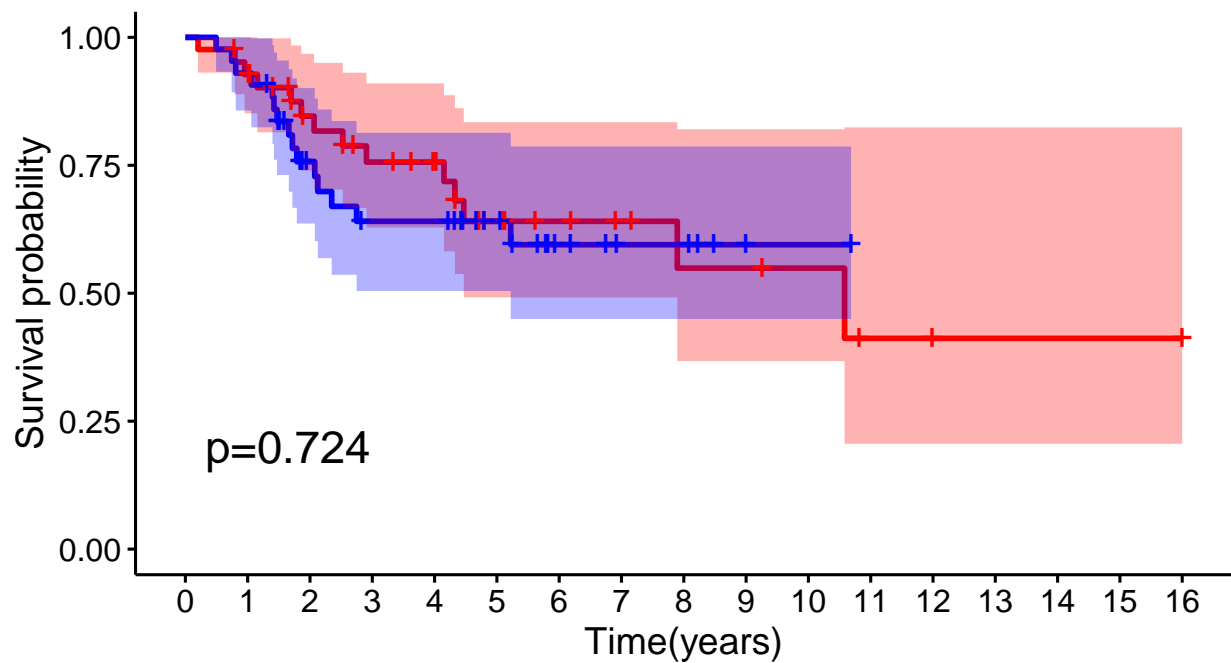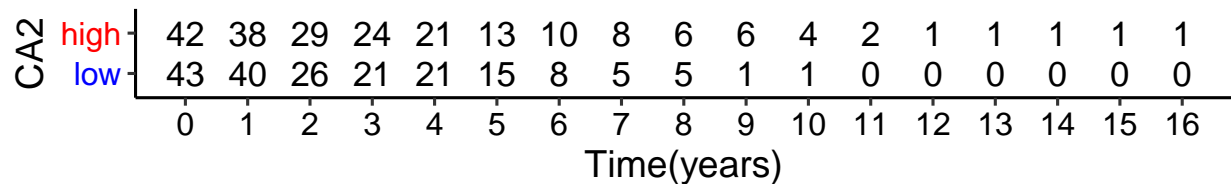

Supplement: Supplementary Document 1 — Kaplan-Meier curve of the 518 genes associated with survival. [file DataSheet_1.zip › Supplementary Document 1/sur.CA2.pdf]

CA3-AS1 + high + low

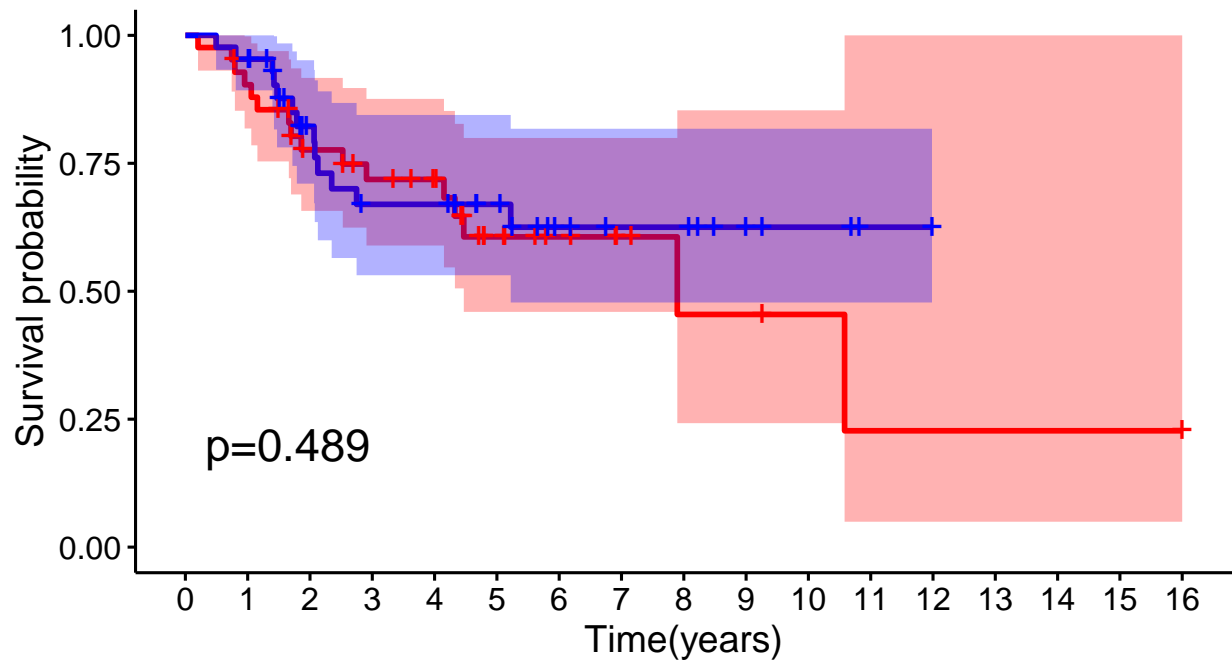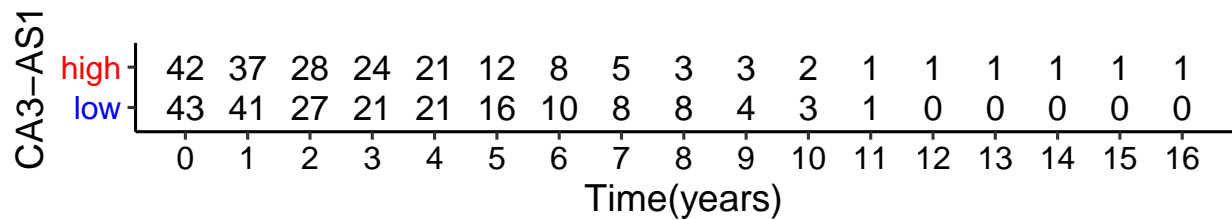

Supplement: Supplementary Document 1 — Kaplan-Meier curve of the 518 genes associated with survival. [file DataSheet_1.zip › Supplementary Document 1/sur.CA3-AS1.pdf]

CA3 + high + low

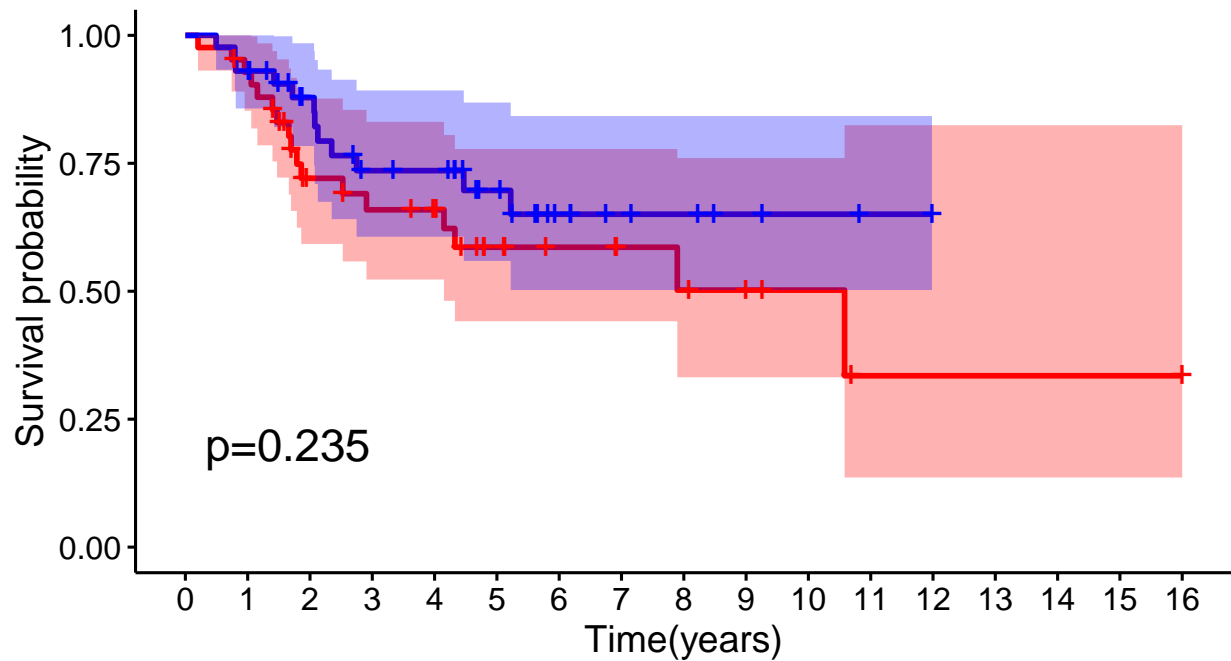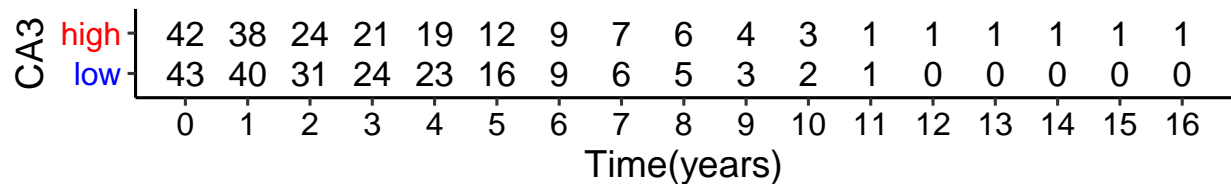

Supplement: Supplementary Document 1 — Kaplan-Meier curve of the 518 genes associated with survival. [file DataSheet_1.zip › Supplementary Document 1/sur.CA3.pdf]

CA5A + high + low

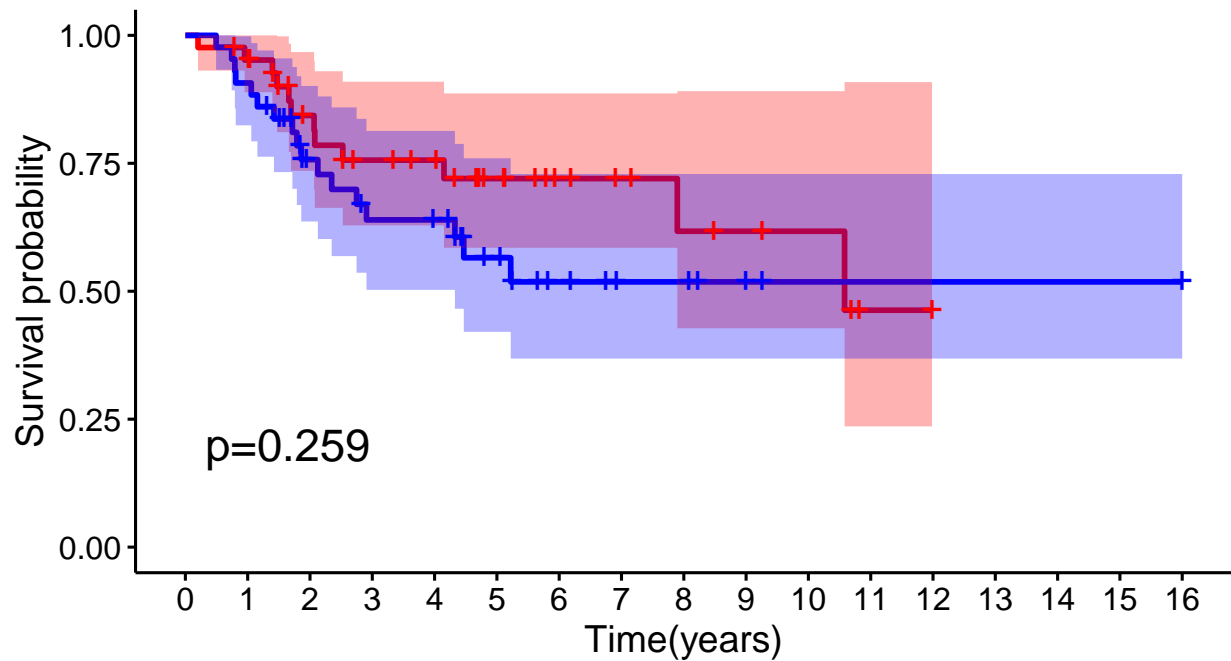

CA5A

|      |    |    |    |    |    |    |    |   |   |   |    |    |    |    |    |    |    |
|------|----|----|----|----|----|----|----|---|---|---|----|----|----|----|----|----|----|
| high | 42 | 39 | 29 | 24 | 22 | 15 | 10 | 8 | 6 | 5 | 4  | 1  | 0  | 0  | 0  | 0  | 0  |
| low  | 43 | 39 | 26 | 21 | 20 | 13 | 8  | 5 | 5 | 2 | 1  | 1  | 1  | 1  | 1  | 1  | 1  |
|      | 0  | 1  | 2  | 3  | 4  | 5  | 6  | 7 | 8 | 9 | 10 | 11 | 12 | 13 | 14 | 15 | 16 |

Time(years)

Supplement: Supplementary Document 1 — Kaplan-Meier curve of the 518 genes associated with survival. [file DataSheet_1.zip › Supplementary Document 1/sur.CA5A.pdf]

CACNA1E + high + low

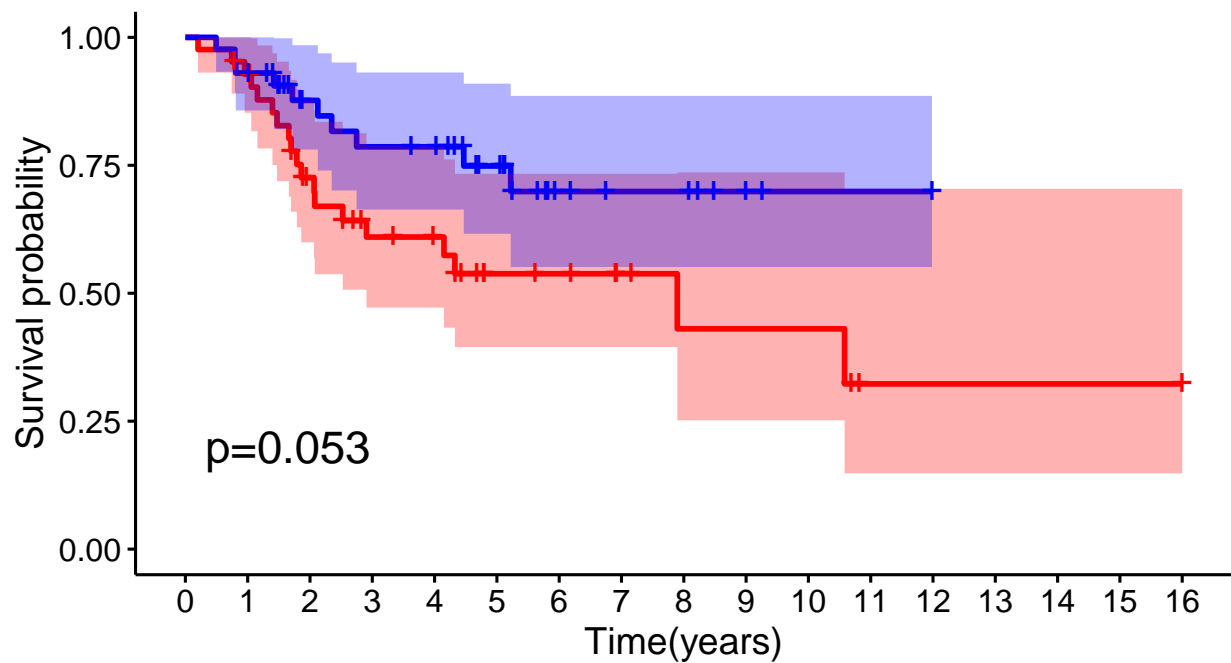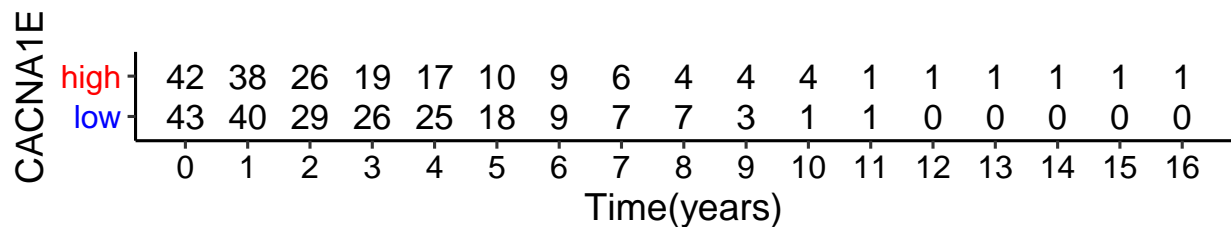

Supplement: Supplementary Document 1 — Kaplan-Meier curve of the 518 genes associated with survival. [file DataSheet_1.zip › Supplementary Document 1/sur.CACNA1E.pdf]

CACNB4 high low

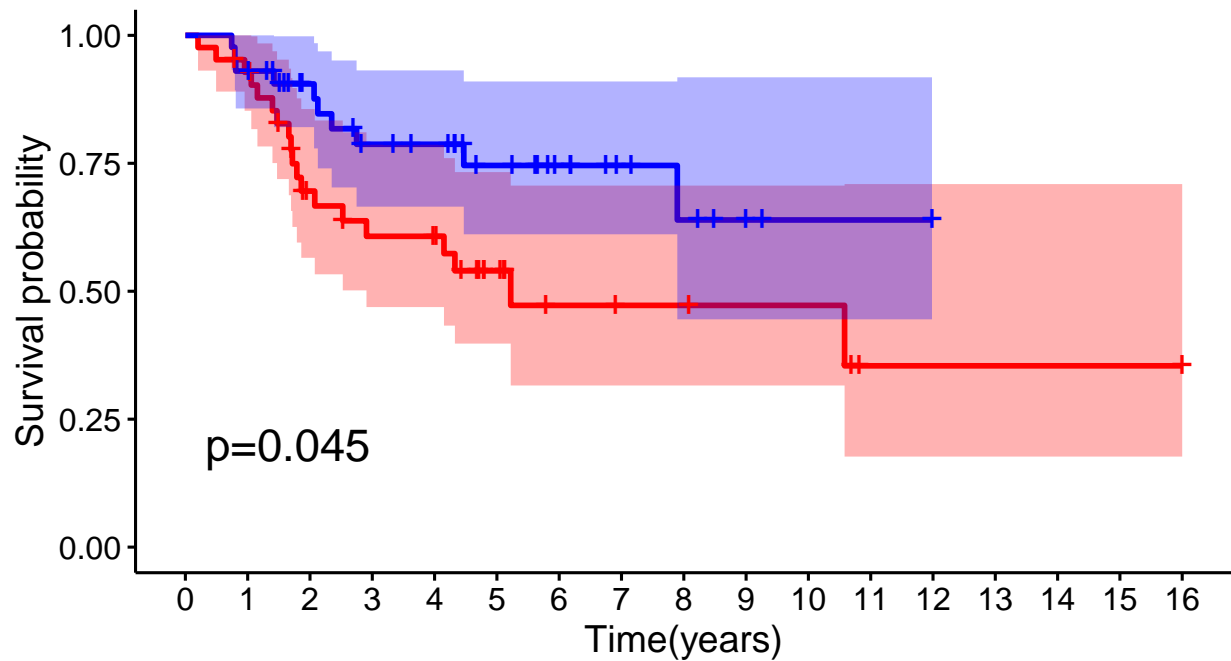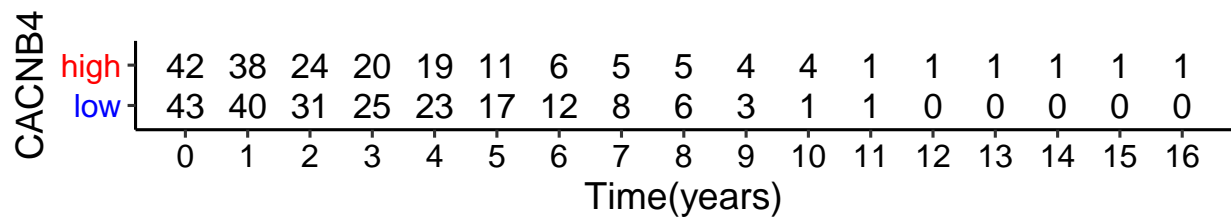

Supplement: Supplementary Document 1 — Kaplan-Meier curve of the 518 genes associated with survival. [file DataSheet_1.zip › Supplementary Document 1/sur.CACNB4.pdf]

Survival probability

CARMIL2 high low

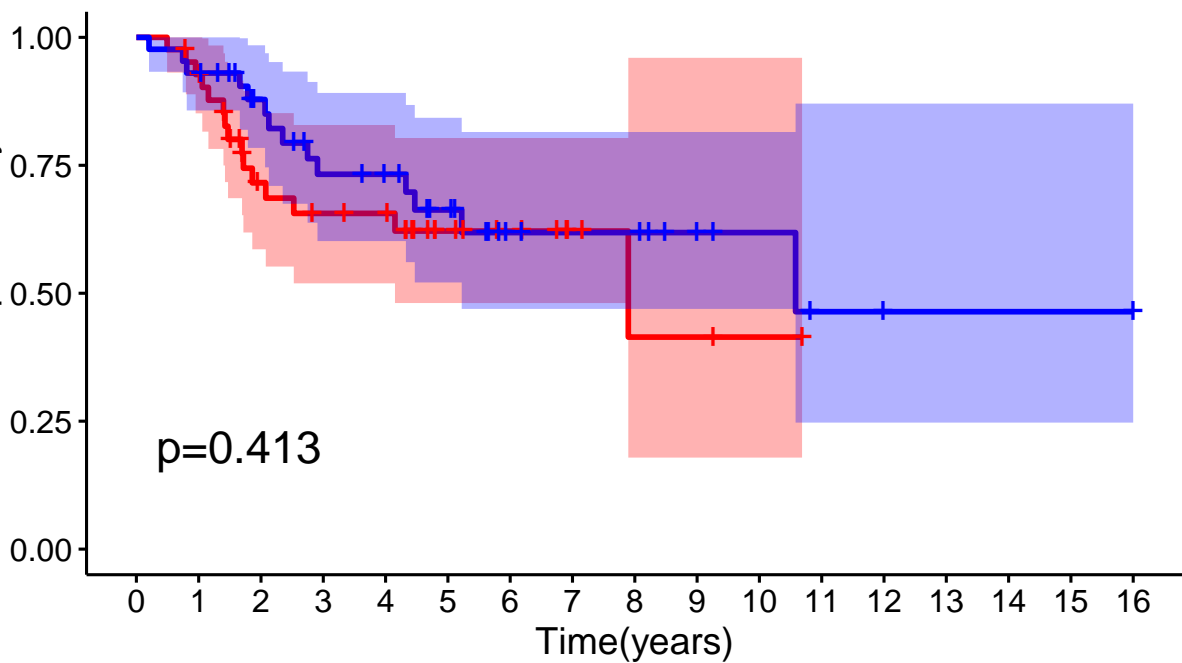

CARMIL2

high

low

|    |    |    |    |    |    |    |   |   |   |    |    |    |    |    |    |
|----|----|----|----|----|----|----|---|---|---|----|----|----|----|----|----|
| 42 | 38 | 24 | 21 | 20 | 11 | 8  | 4 | 2 | 2 | 1  | 0  | 0  | 0  | 0  | 0  |
| 43 | 40 | 31 | 24 | 22 | 17 | 10 | 9 | 9 | 5 | 4  | 2  | 1  | 1  | 1  | 1  |
| 0  | 1  | 2  | 3  | 4  | 5  | 6  | 7 | 8 | 9 | 10 | 11 | 12 | 13 | 14 | 15 |

Time(years)

Supplement: Supplementary Document 1 — Kaplan-Meier curve of the 518 genes associated with survival. [file DataSheet_1.zip › Supplementary Document 1/sur.CARMIL2.pdf]

CASP5 + high + low

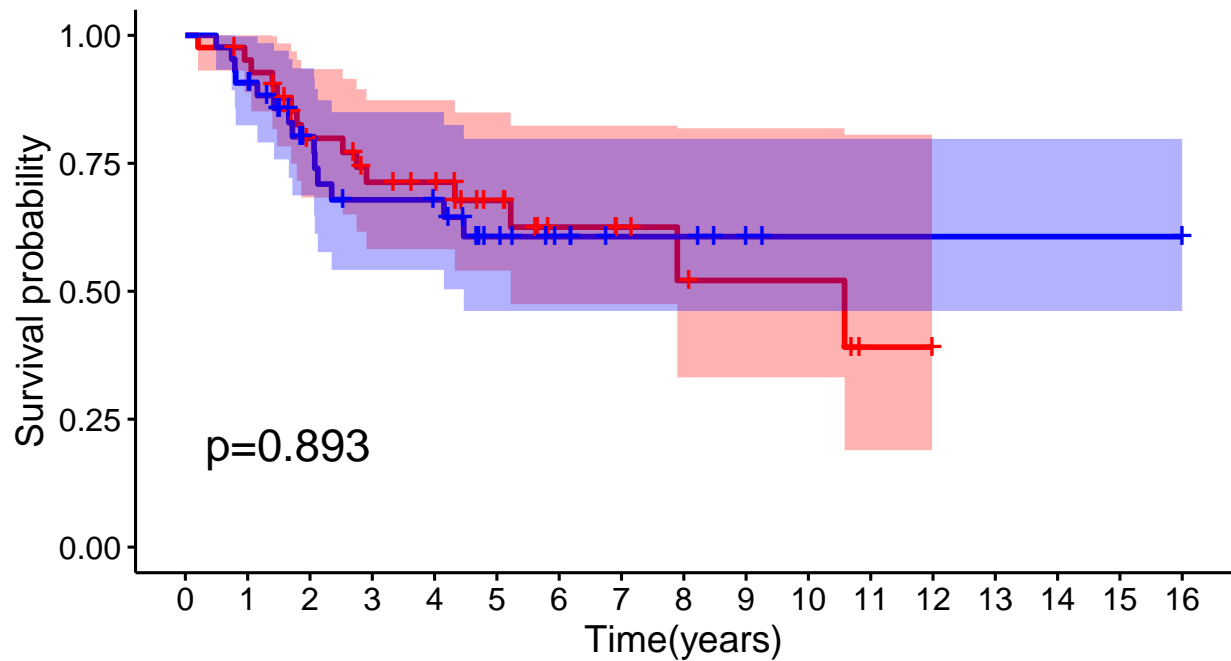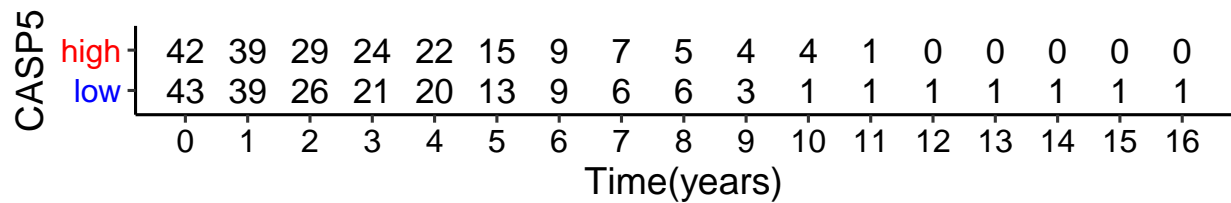

Supplement: Supplementary Document 1 — Kaplan-Meier curve of the 518 genes associated with survival. [file DataSheet_1.zip › Supplementary Document 1/sur.CASP5.pdf]

CCDC158 + high + low

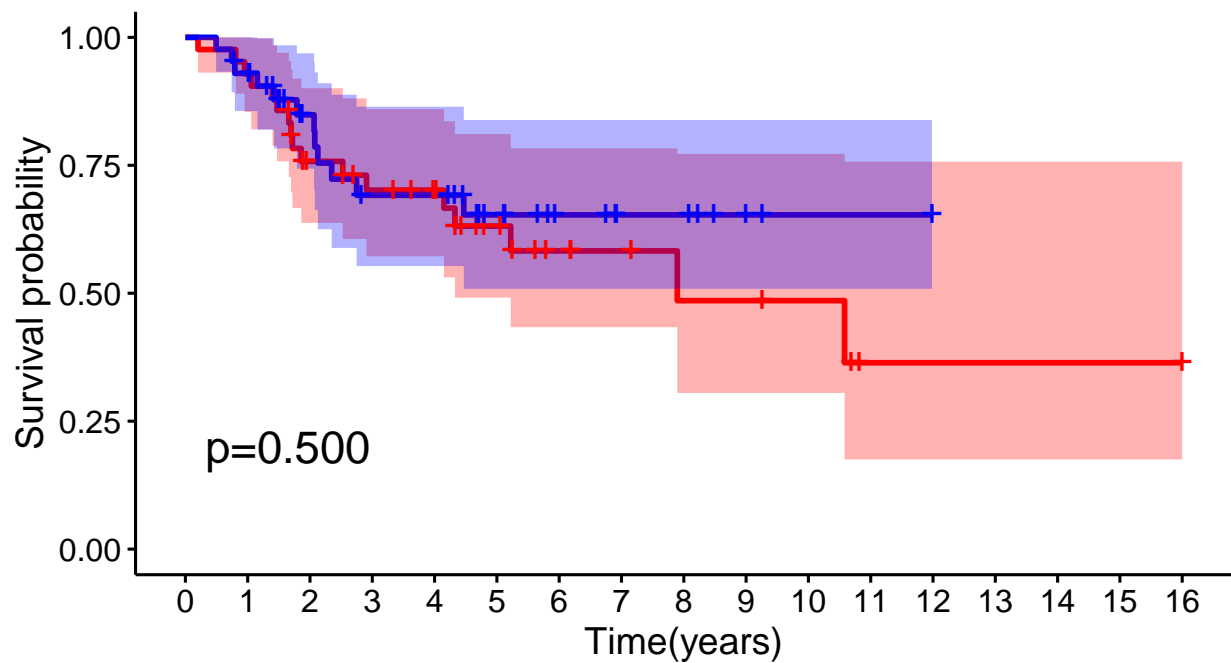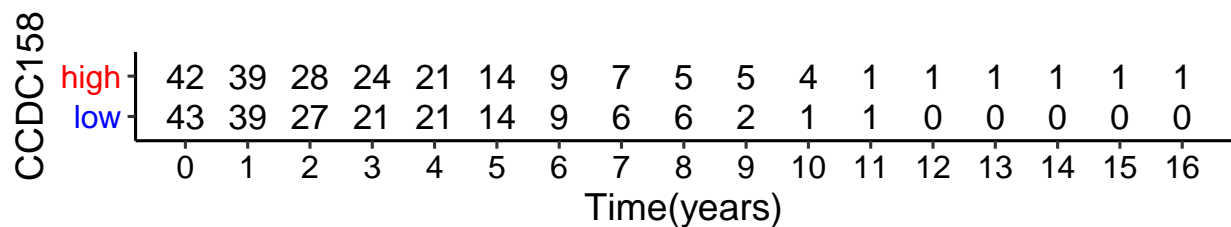

Supplement: Supplementary Document 1 — Kaplan-Meier curve of the 518 genes associated with survival. [file DataSheet_1.zip › Supplementary Document 1/sur.CCDC158.pdf]

CCDC194 + high + low

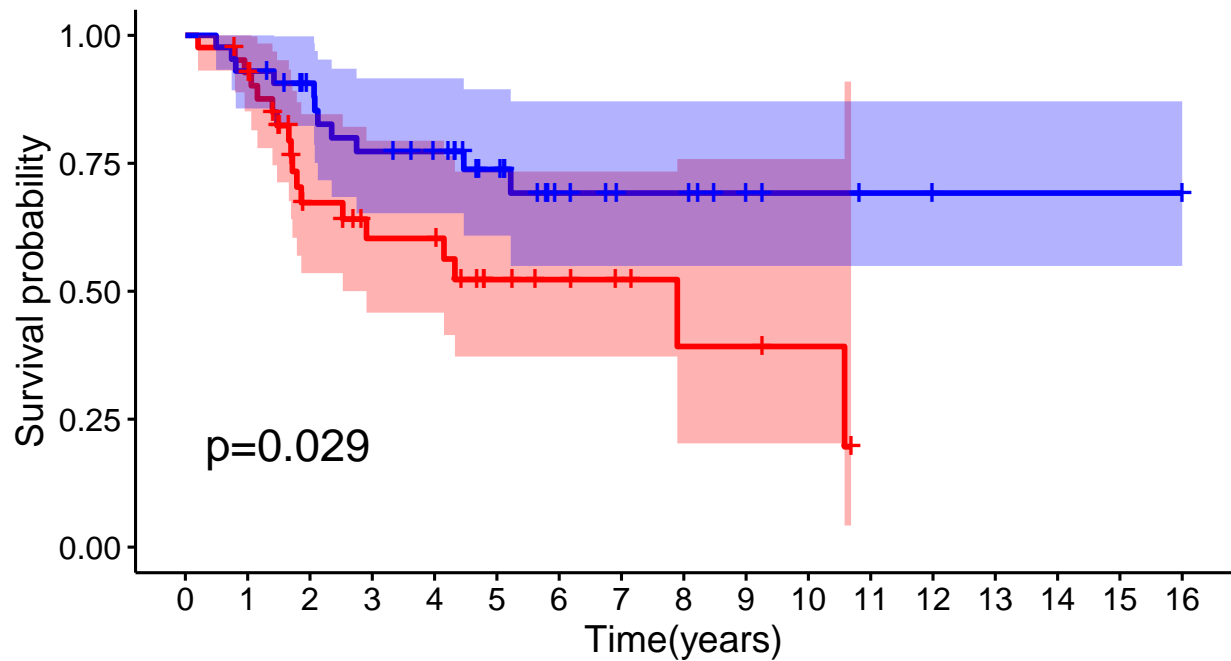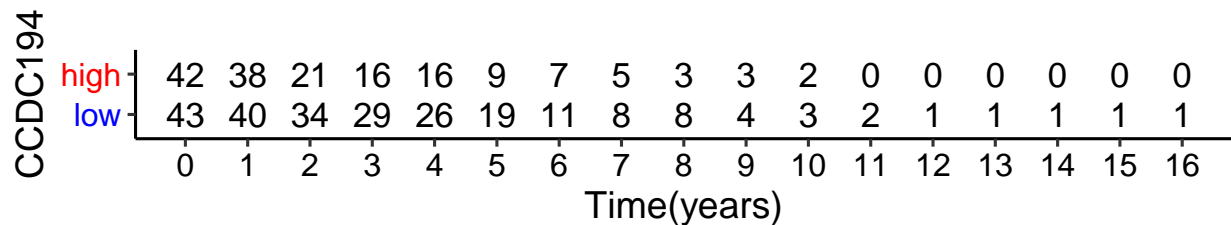

Supplement: Supplementary Document 1 — Kaplan-Meier curve of the 518 genes associated with survival. [file DataSheet_1.zip › Supplementary Document 1/sur.CCDC194.pdf]

CCDC3 + high + low

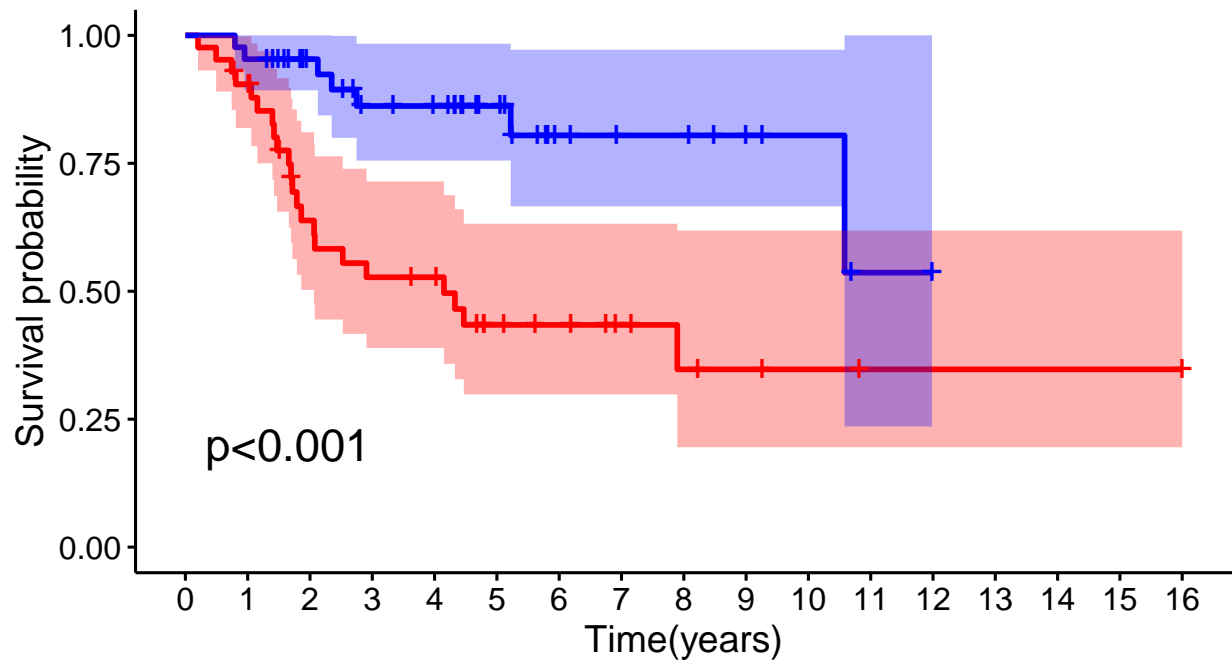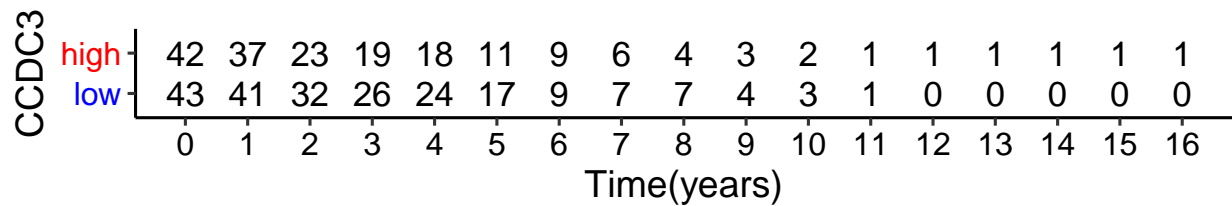

Supplement: Supplementary Document 1 — Kaplan-Meier curve of the 518 genes associated with survival. [file DataSheet_1.zip › Supplementary Document 1/sur.CCDC3.pdf]

CCL3 high low

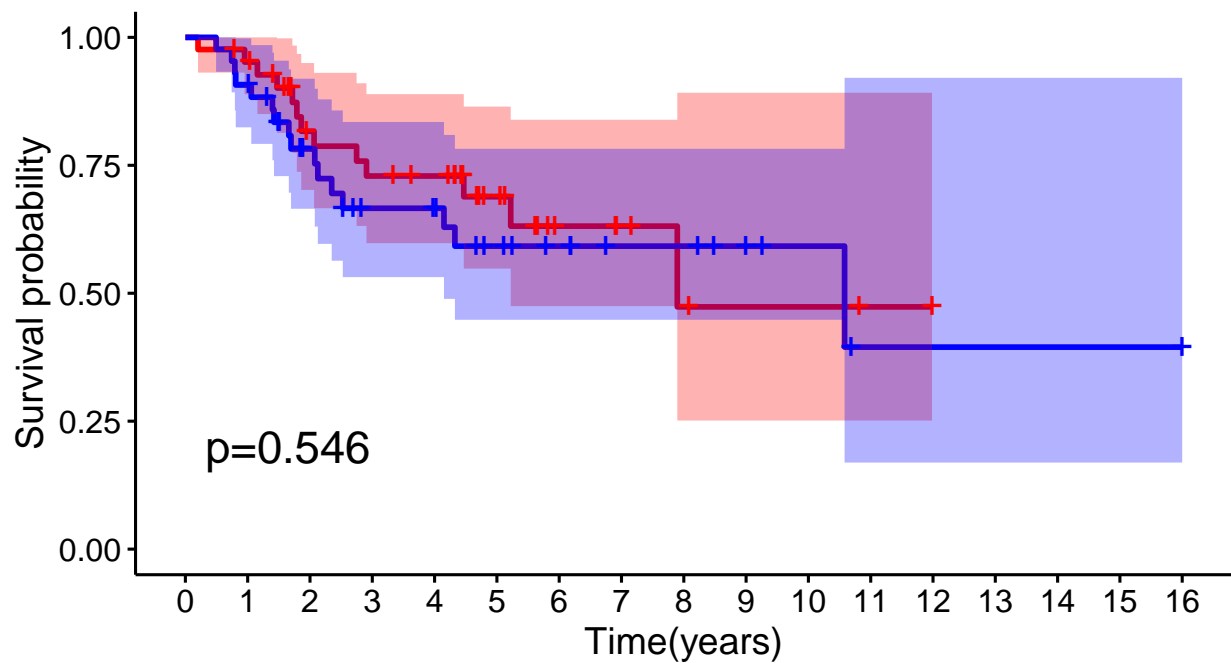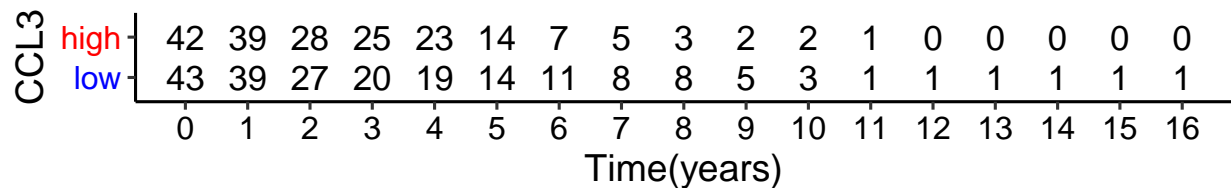

Supplement: Supplementary Document 1 — Kaplan-Meier curve of the 518 genes associated with survival. [file DataSheet_1.zip › Supplementary Document 1/sur.CCL3.pdf]

CD36 + high + low

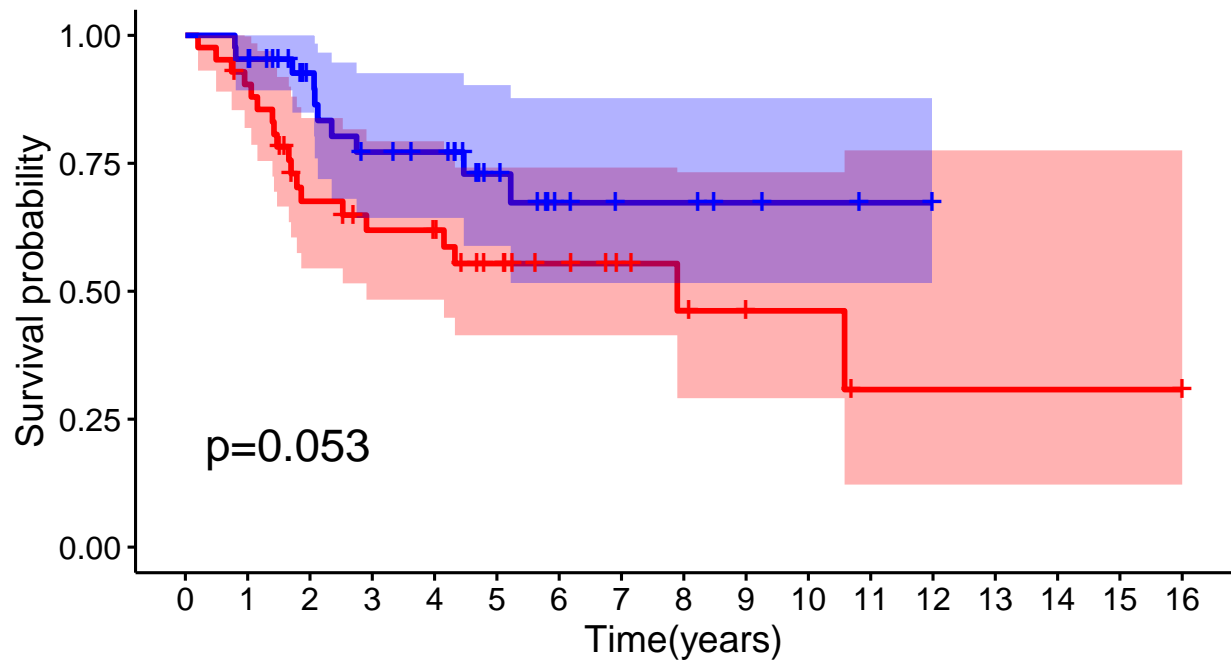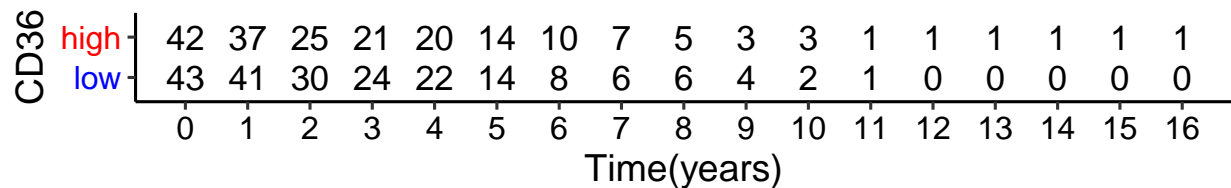

Supplement: Supplementary Document 1 — Kaplan-Meier curve of the 518 genes associated with survival. [file DataSheet_1.zip › Supplementary Document 1/sur.CD36.pdf]

CDC14A high low

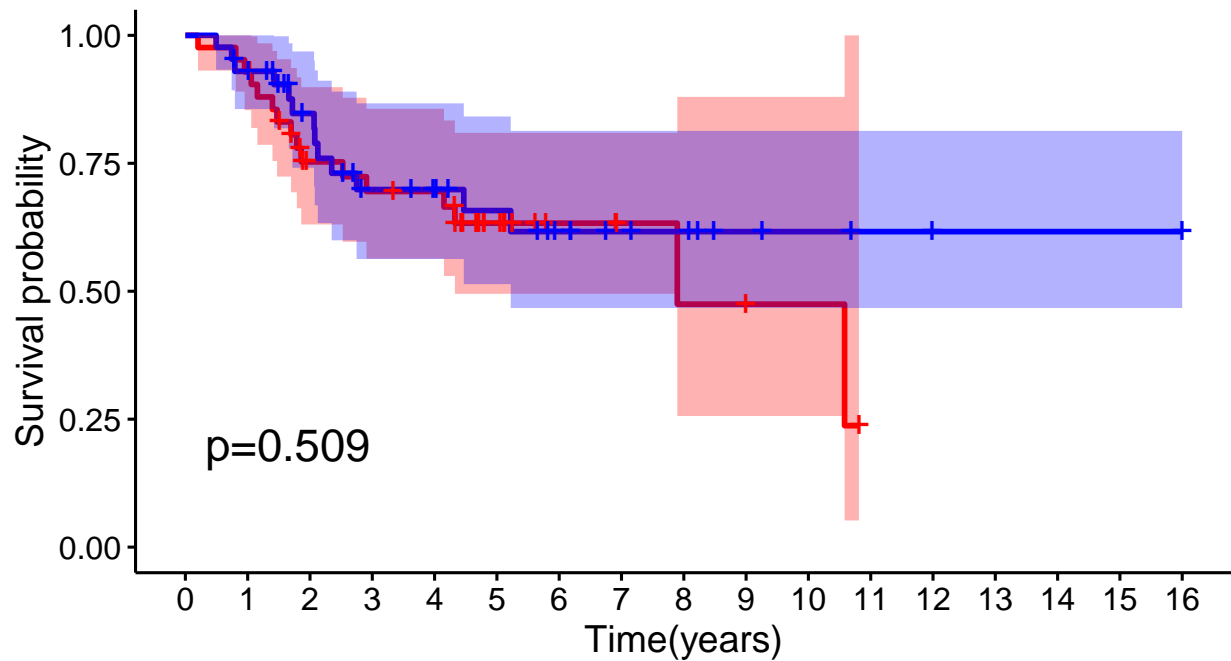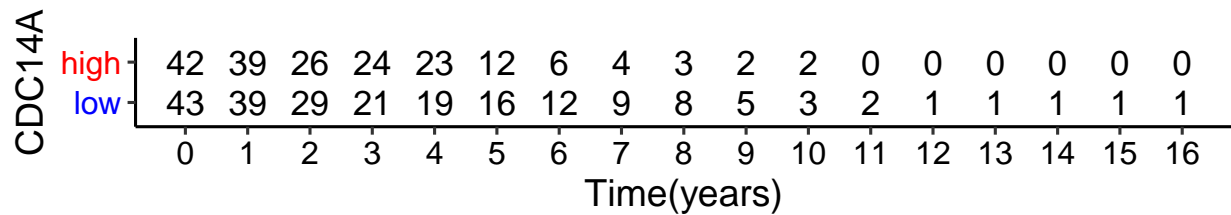

Supplement: Supplementary Document 1 — Kaplan-Meier curve of the 518 genes associated with survival. [file DataSheet_1.zip › Supplementary Document 1/sur.CDC14A.pdf]

CDC20B high low

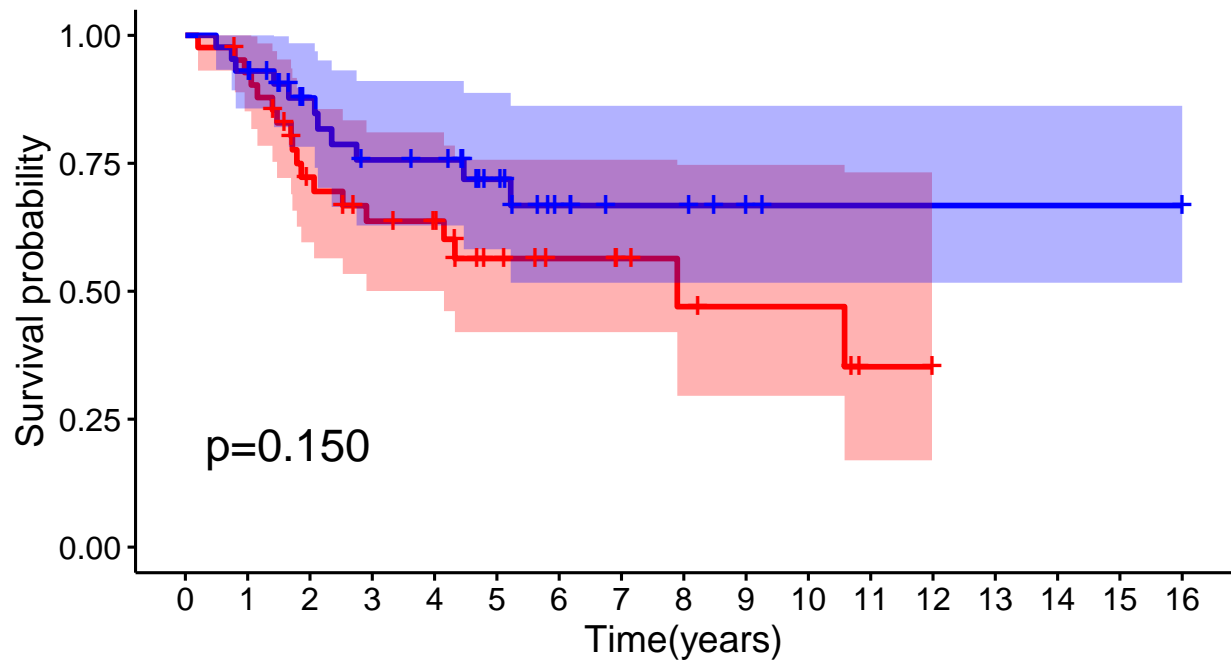

CDC20B

high

low

|    |    |    |    |    |    |   |   |   |   |    |    |    |    |    |    |
|----|----|----|----|----|----|---|---|---|---|----|----|----|----|----|----|
| 42 | 38 | 26 | 21 | 19 | 12 | 9 | 7 | 5 | 4 | 4  | 1  | 0  | 0  | 0  | 0  |
| 43 | 40 | 29 | 24 | 23 | 16 | 9 | 6 | 6 | 3 | 1  | 1  | 1  | 1  | 1  | 1  |
| 0  | 1  | 2  | 3  | 4  | 5  | 6 | 7 | 8 | 9 | 10 | 11 | 12 | 13 | 14 | 15 |

Time(years)

Supplement: Supplementary Document 1 — Kaplan-Meier curve of the 518 genes associated with survival. [file DataSheet_1.zip › Supplementary Document 1/sur.CDC20B.pdf]

CDC42EP3 + high + low

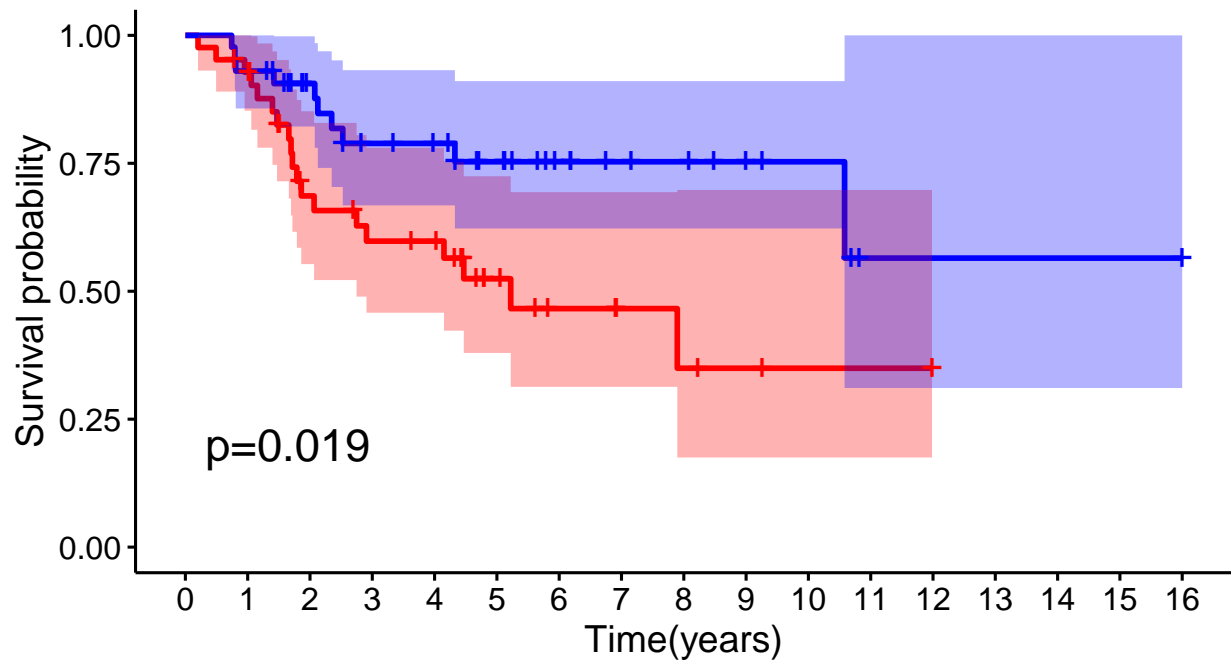

CDC42EP3

high  
low

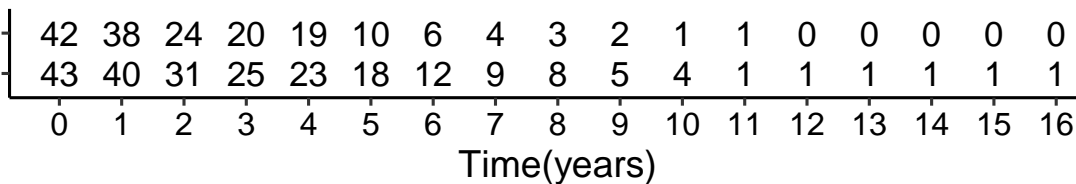

Supplement: Supplementary Document 1 — Kaplan-Meier curve of the 518 genes associated with survival. [file DataSheet_1.zip › Supplementary Document 1/sur.CDC42EP3.pdf]

CDH15 + high + low

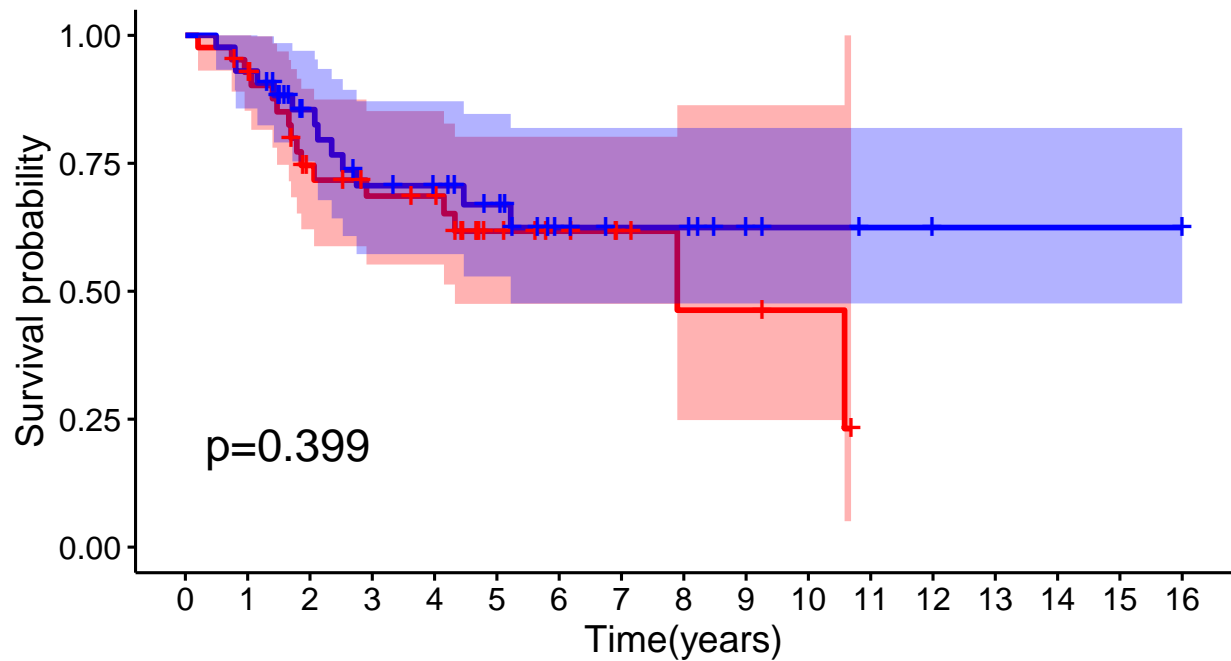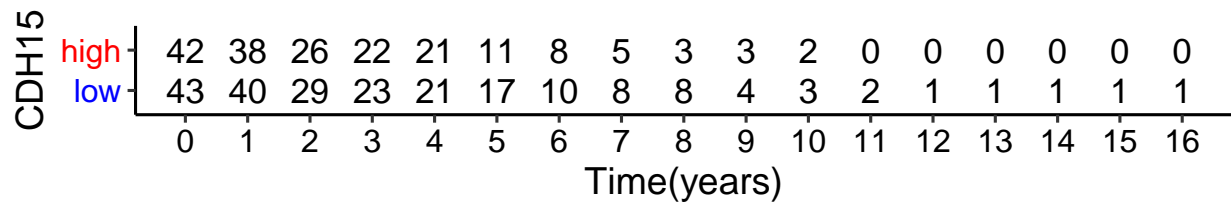

Supplement: Supplementary Document 1 — Kaplan-Meier curve of the 518 genes associated with survival. [file DataSheet_1.zip › Supplementary Document 1/sur.CDH15.pdf]

CDH8 + high + low

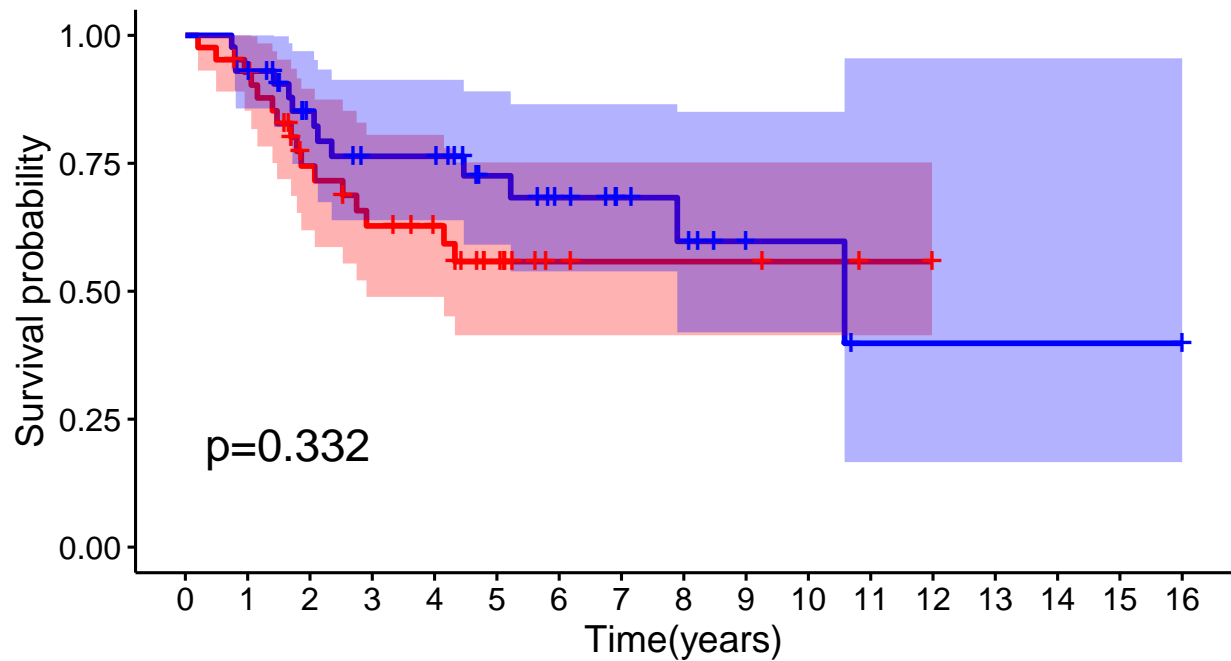

CDH8

|      |    |    |    |    |    |    |    |   |   |   |    |    |    |    |    |    |    |
|------|----|----|----|----|----|----|----|---|---|---|----|----|----|----|----|----|----|
| high | 42 | 38 | 26 | 21 | 18 | 11 | 5  | 4 | 4 | 4 | 2  | 1  | 0  | 0  | 0  | 0  | 0  |
| low  | 43 | 40 | 29 | 24 | 24 | 17 | 13 | 9 | 7 | 3 | 3  | 1  | 1  | 1  | 1  | 1  | 1  |
|      | 0  | 1  | 2  | 3  | 4  | 5  | 6  | 7 | 8 | 9 | 10 | 11 | 12 | 13 | 14 | 15 | 16 |

Time(years)

Supplement: Supplementary Document 1 — Kaplan-Meier curve of the 518 genes associated with survival. [file DataSheet_1.zip › Supplementary Document 1/sur.CDH8.pdf]

CDK18 high low

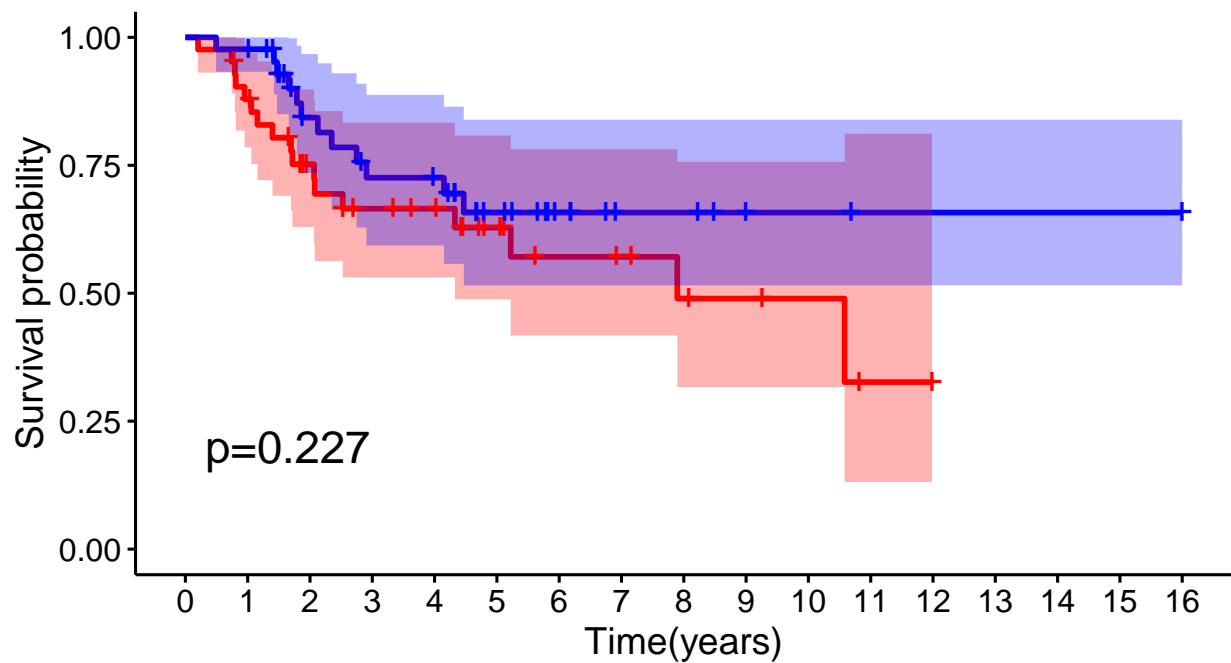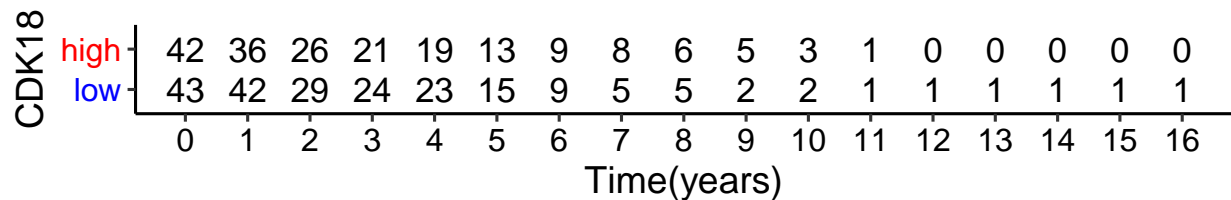

Supplement: Supplementary Document 1 — Kaplan-Meier curve of the 518 genes associated with survival. [file DataSheet_1.zip › Supplementary Document 1/sur.CDK18.pdf]

CFAP300 + high + low

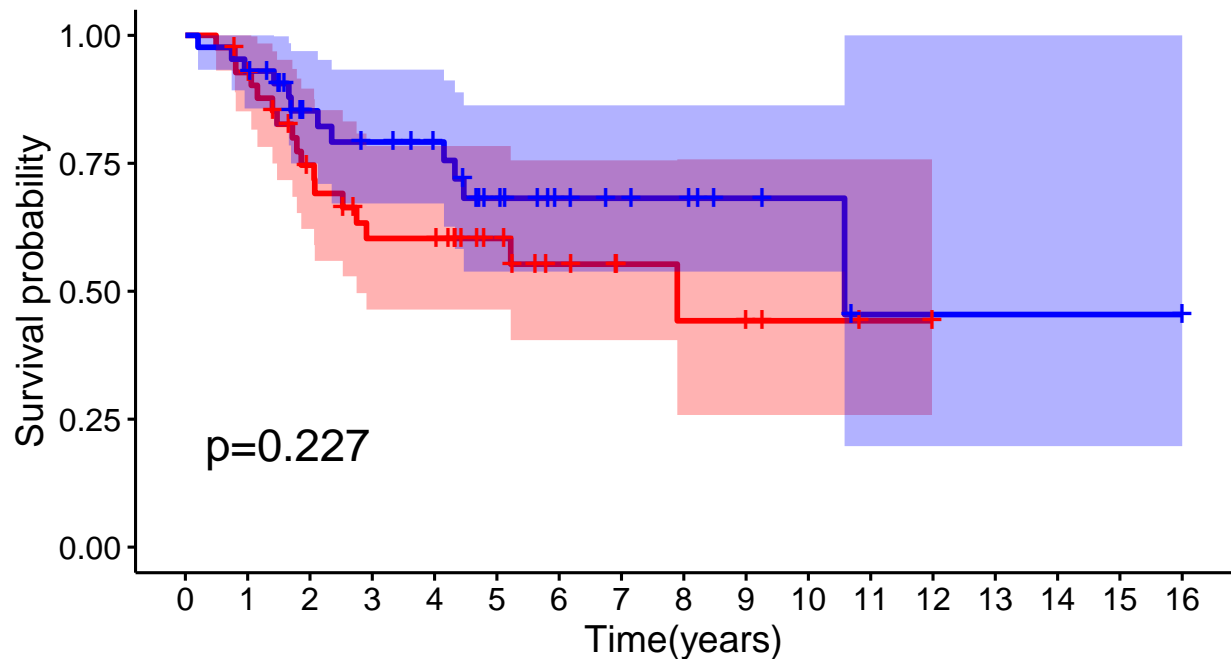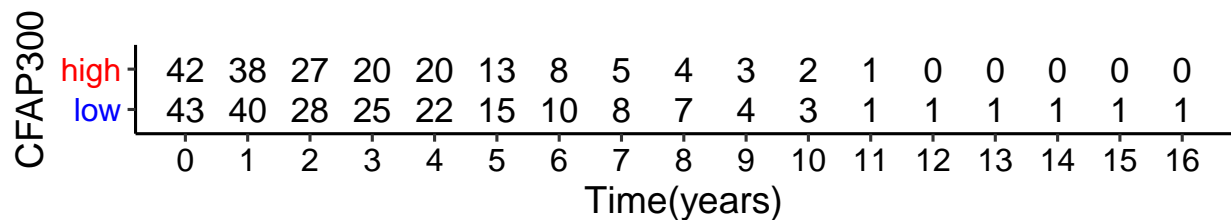

Supplement: Supplementary Document 1 — Kaplan-Meier curve of the 518 genes associated with survival. [file DataSheet_1.zip › Supplementary Document 1/sur.CFAP300.pdf]

CFAP44 + high + low

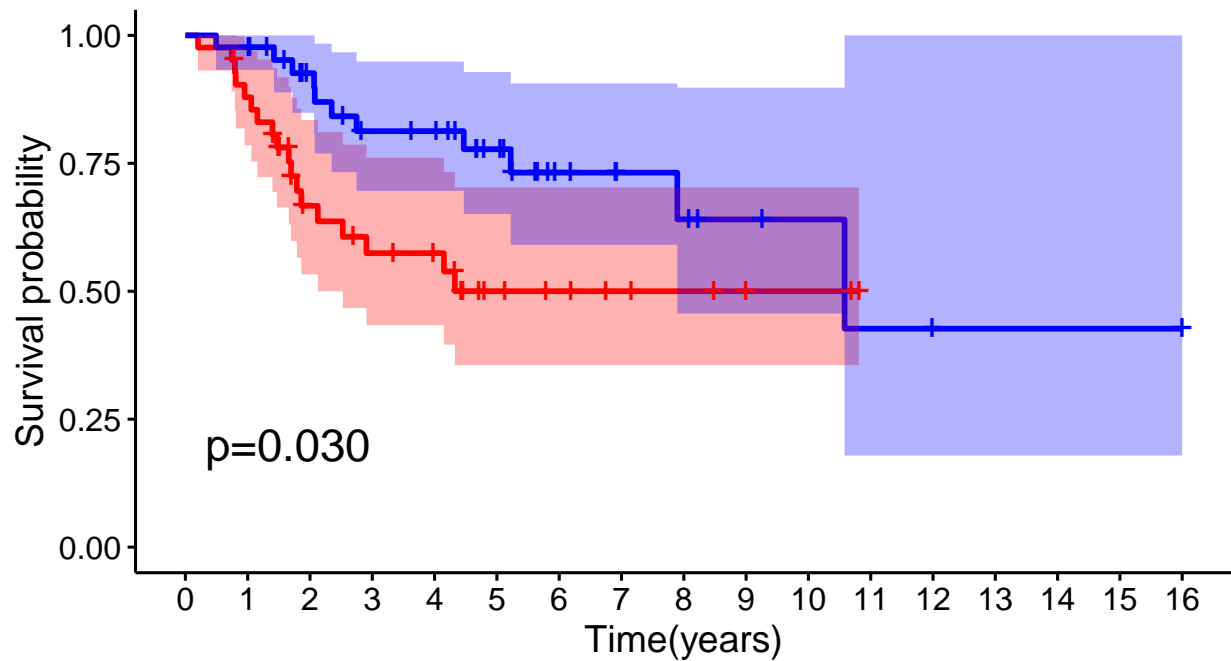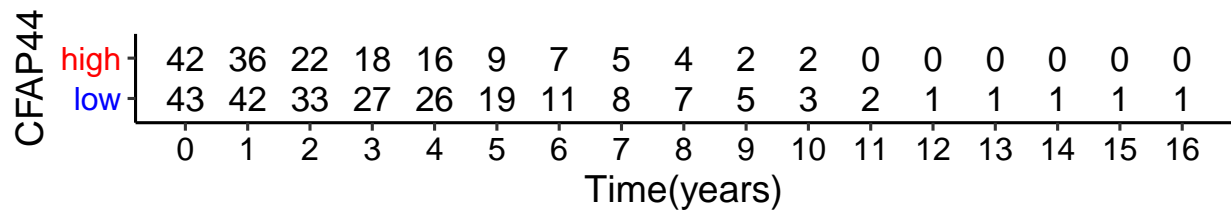

Supplement: Supplementary Document 1 — Kaplan-Meier curve of the 518 genes associated with survival. [file DataSheet_1.zip › Supplementary Document 1/sur.CFAP44.pdf]

CGREF1 high low

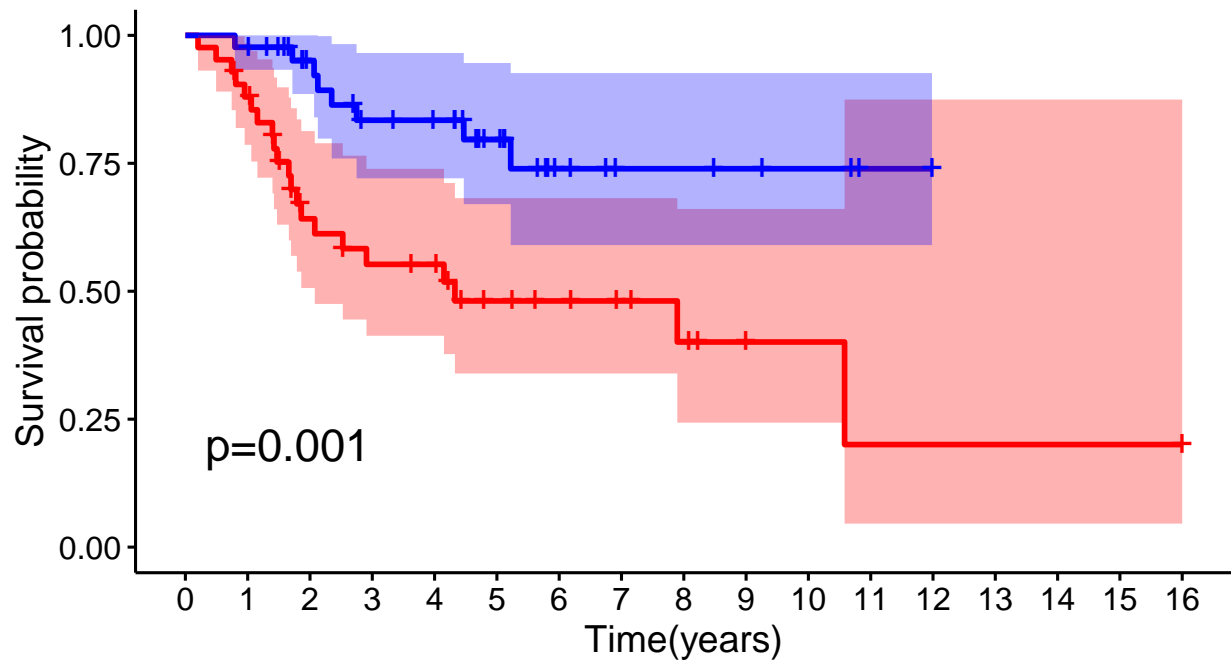

CGREF1

high

low

|    |    |    |    |    |    |   |   |   |   |    |    |    |    |    |    |
|----|----|----|----|----|----|---|---|---|---|----|----|----|----|----|----|
| 42 | 36 | 22 | 18 | 17 | 11 | 9 | 7 | 5 | 2 | 2  | 1  | 1  | 1  | 1  | 1  |
| 43 | 42 | 33 | 27 | 25 | 17 | 9 | 6 | 6 | 5 | 3  | 1  | 0  | 0  | 0  | 0  |
| 0  | 1  | 2  | 3  | 4  | 5  | 6 | 7 | 8 | 9 | 10 | 11 | 12 | 13 | 14 | 15 |

Time(years)

Supplement: Supplementary Document 1 — Kaplan-Meier curve of the 518 genes associated with survival. [file DataSheet_1.zip › Supplementary Document 1/sur.CGREF1.pdf]

CHML + high + low

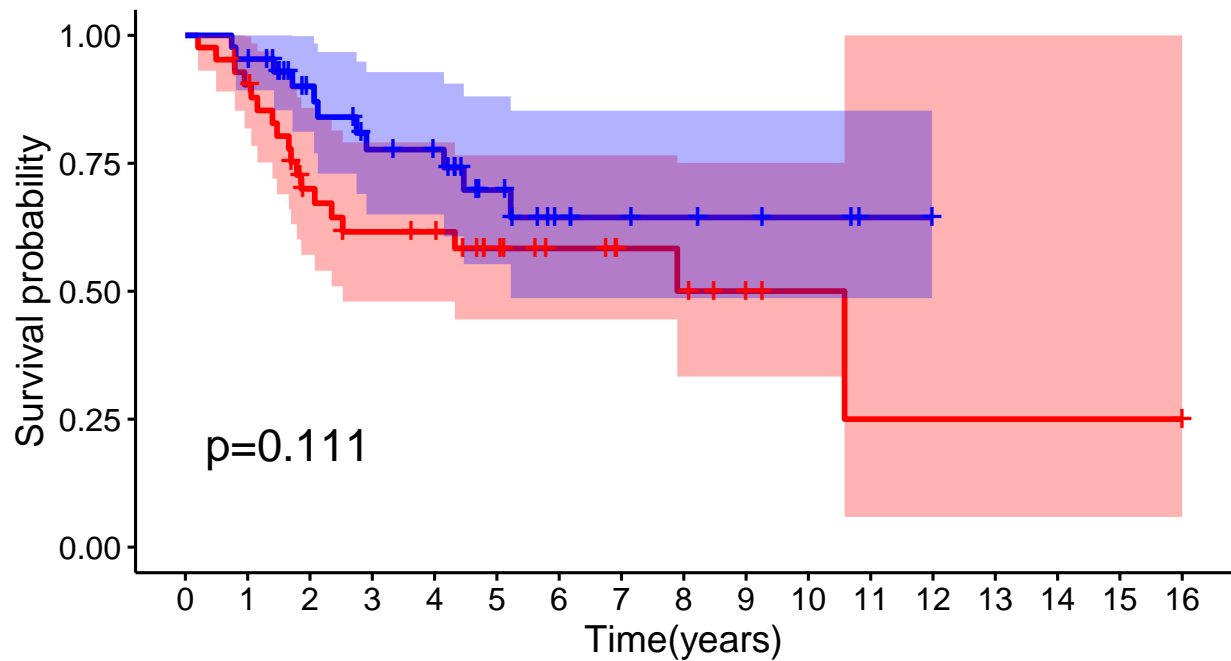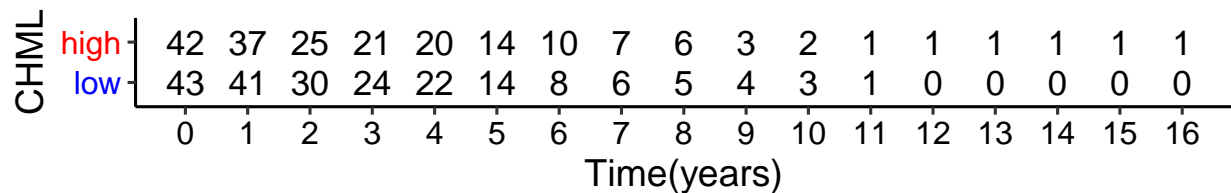

Supplement: Supplementary Document 1 — Kaplan-Meier curve of the 518 genes associated with survival. [file DataSheet_1.zip › Supplementary Document 1/sur.CHML.pdf]

CHMP4C high low

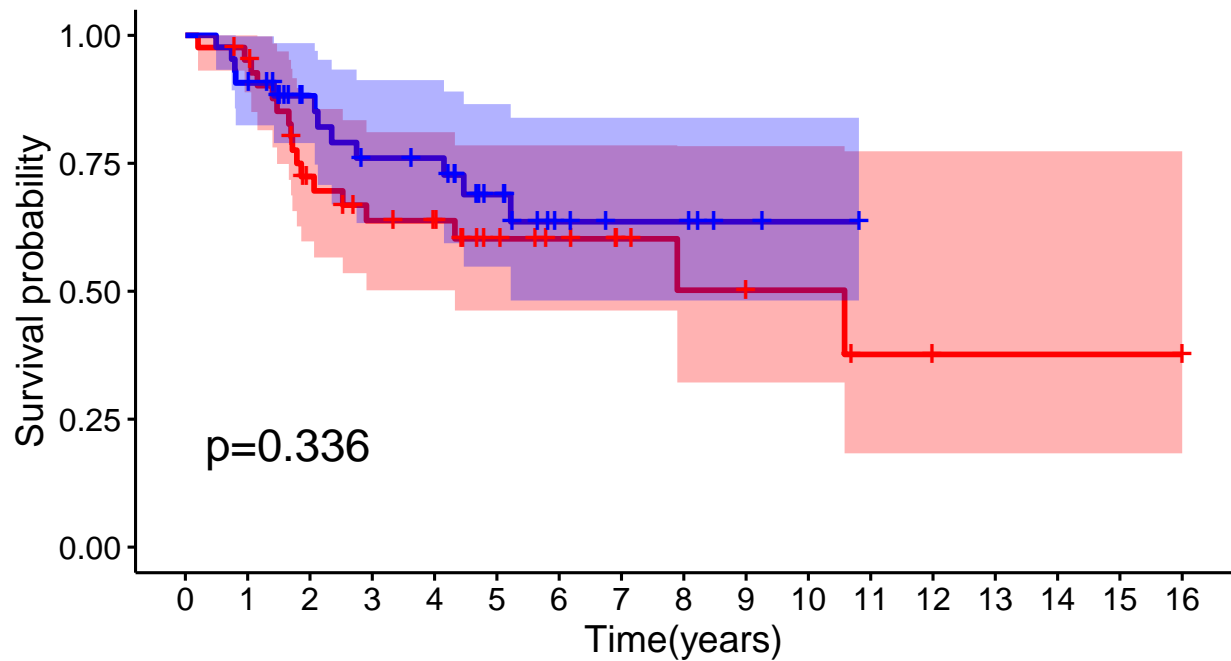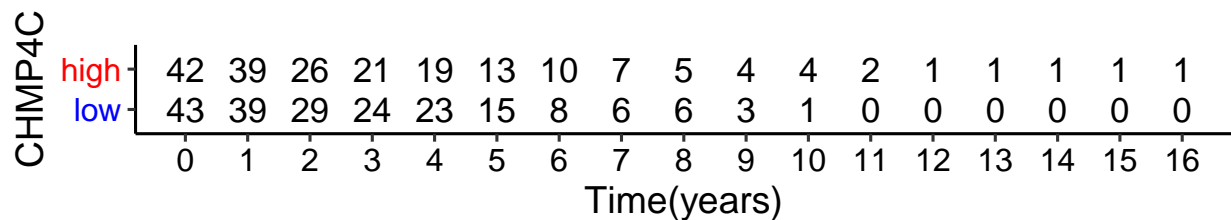

Supplement: Supplementary Document 1 — Kaplan-Meier curve of the 518 genes associated with survival. [file DataSheet_1.zip › Supplementary Document 1/sur.CHMP4C.pdf]

CHST13 high low

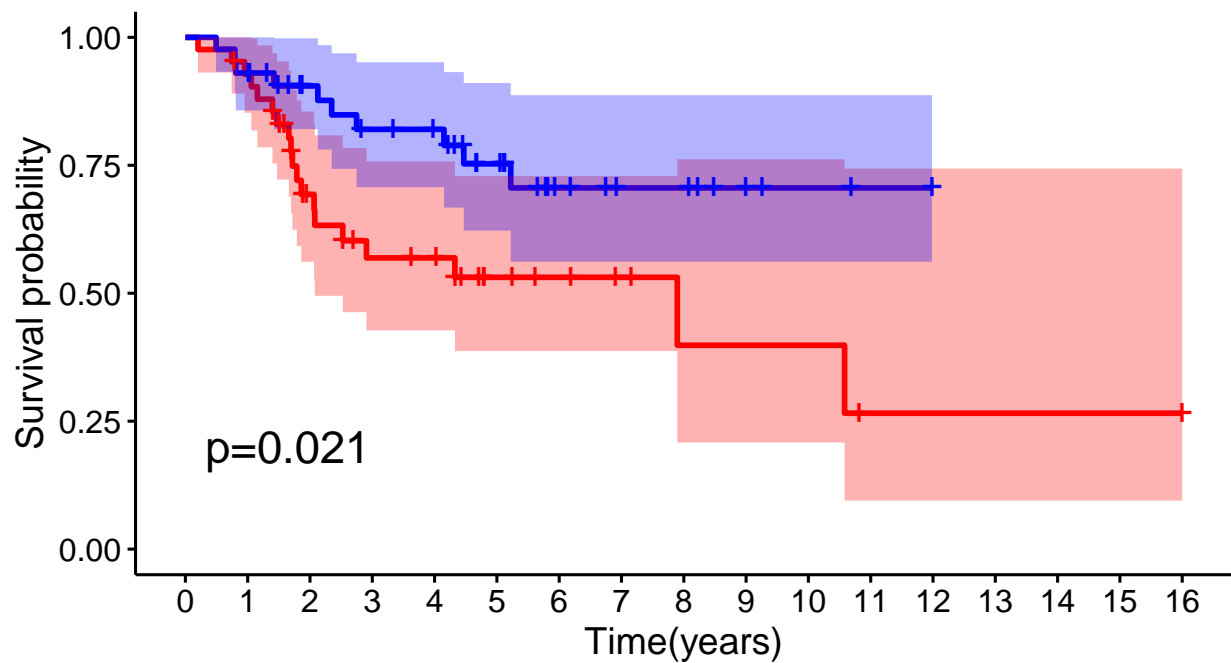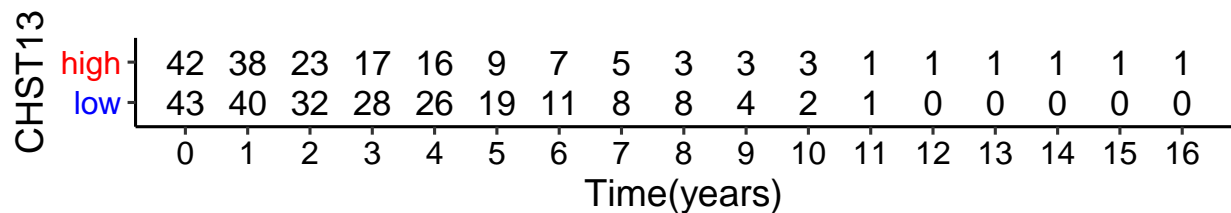

Supplement: Supplementary Document 1 — Kaplan-Meier curve of the 518 genes associated with survival. [file DataSheet_1.zip › Supplementary Document 1/sur.CHST13.pdf]

CHST8 + high + low

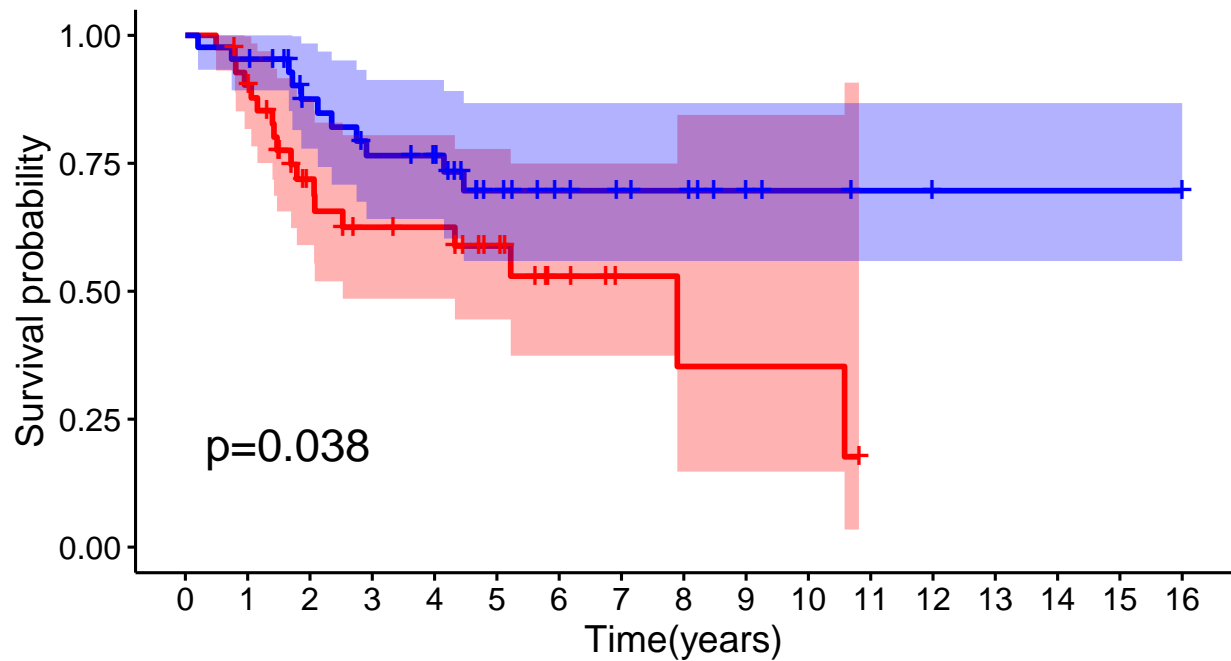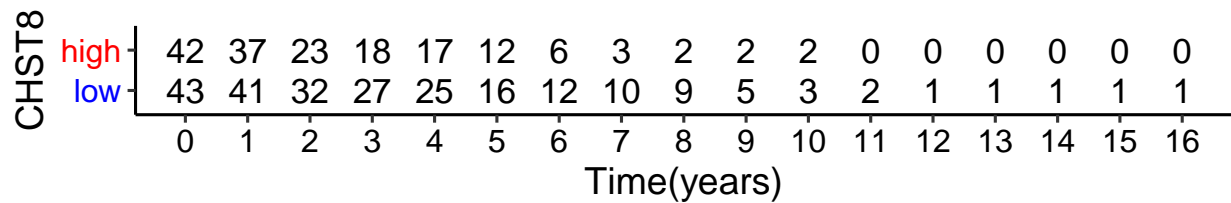

Supplement: Supplementary Document 1 — Kaplan-Meier curve of the 518 genes associated with survival. [file DataSheet_1.zip › Supplementary Document 1/sur.CHST8.pdf]

CITED4 + high + low

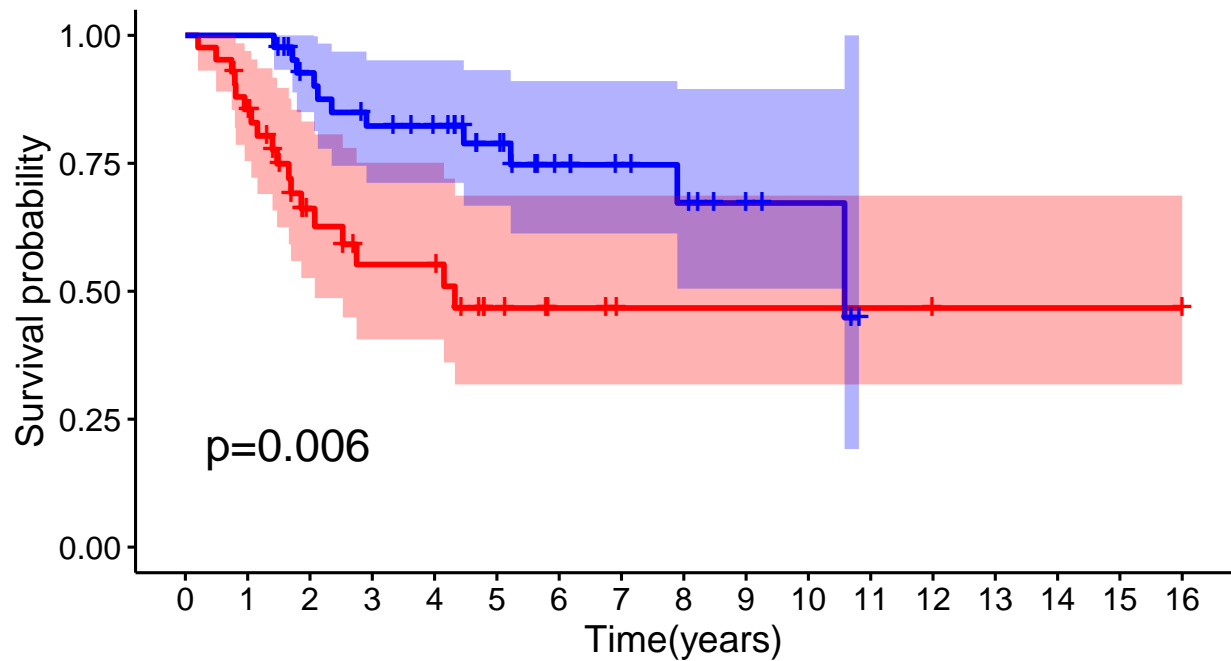

CITED4

|      |    |    |    |    |    |    |    |    |   |   |    |    |    |    |    |    |    |
|------|----|----|----|----|----|----|----|----|---|---|----|----|----|----|----|----|----|
| high | 42 | 35 | 19 | 14 | 14 | 7  | 4  | 2  | 2 | 2 | 2  | 2  | 1  | 1  | 1  | 1  | 1  |
| low  | 43 | 43 | 36 | 31 | 28 | 21 | 14 | 11 | 9 | 5 | 3  | 0  | 0  | 0  | 0  | 0  | 0  |
|      | 0  | 1  | 2  | 3  | 4  | 5  | 6  | 7  | 8 | 9 | 10 | 11 | 12 | 13 | 14 | 15 | 16 |

Time(years)

Supplement: Supplementary Document 1 — Kaplan-Meier curve of the 518 genes associated with survival. [file DataSheet_1.zip › Supplementary Document 1/sur.CITED4.pdf]

CKB + high + low

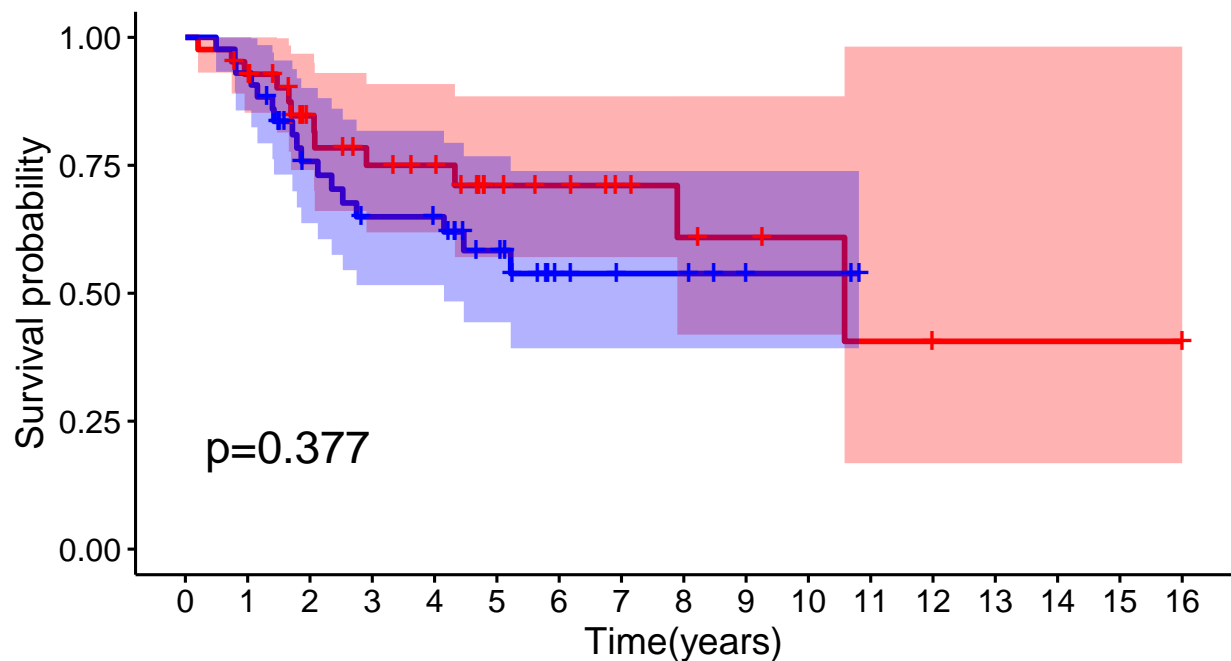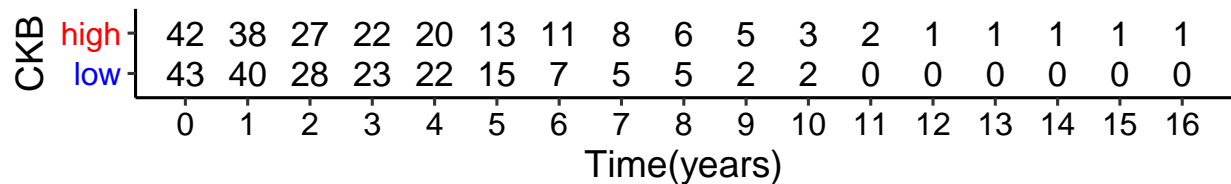

Supplement: Supplementary Document 1 — Kaplan-Meier curve of the 518 genes associated with survival. [file DataSheet_1.zip › Supplementary Document 1/sur.CKB.pdf]

CKMT1B high low

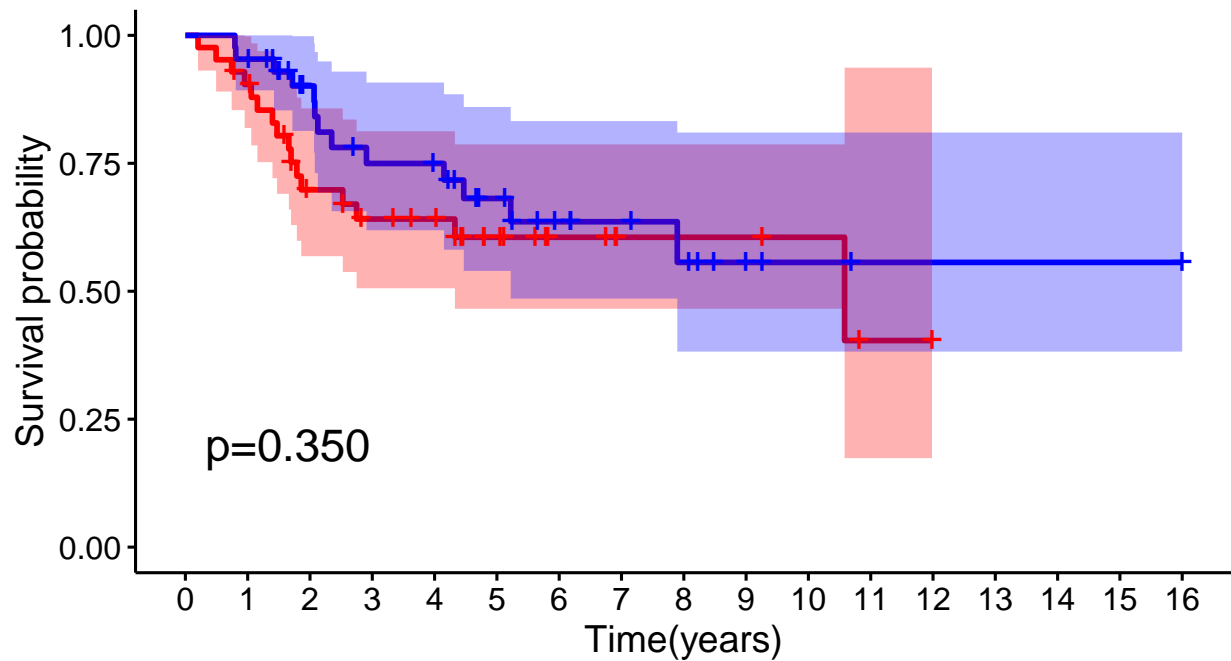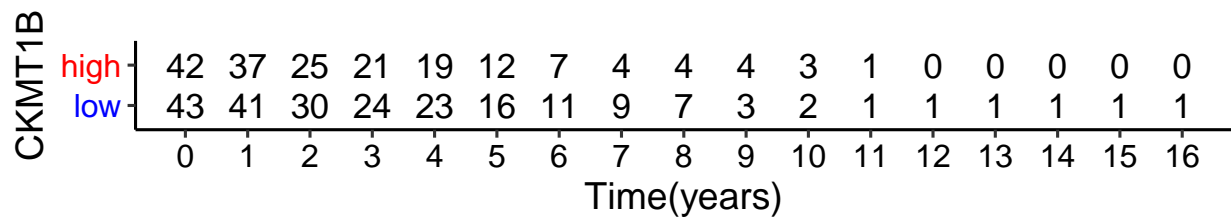

Supplement: Supplementary Document 1 — Kaplan-Meier curve of the 518 genes associated with survival. [file DataSheet_1.zip › Supplementary Document 1/sur.CKMT1B.pdf]

CLDN11 + high + low

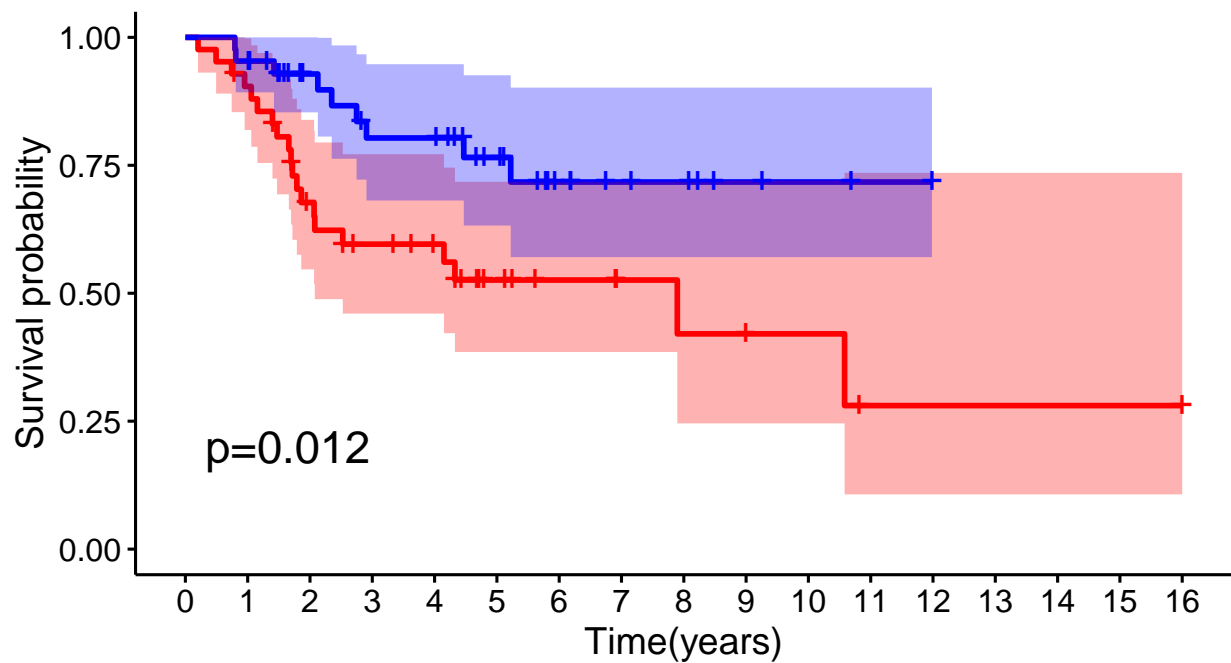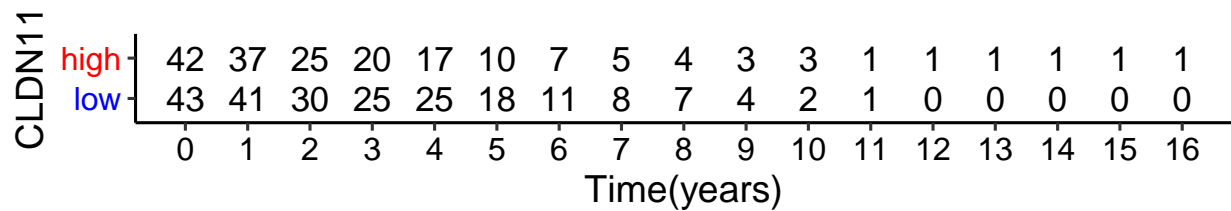

Supplement: Supplementary Document 1 — Kaplan-Meier curve of the 518 genes associated with survival. [file DataSheet_1.zip › Supplementary Document 1/sur.CLDN11.pdf]

CLEC4A high low

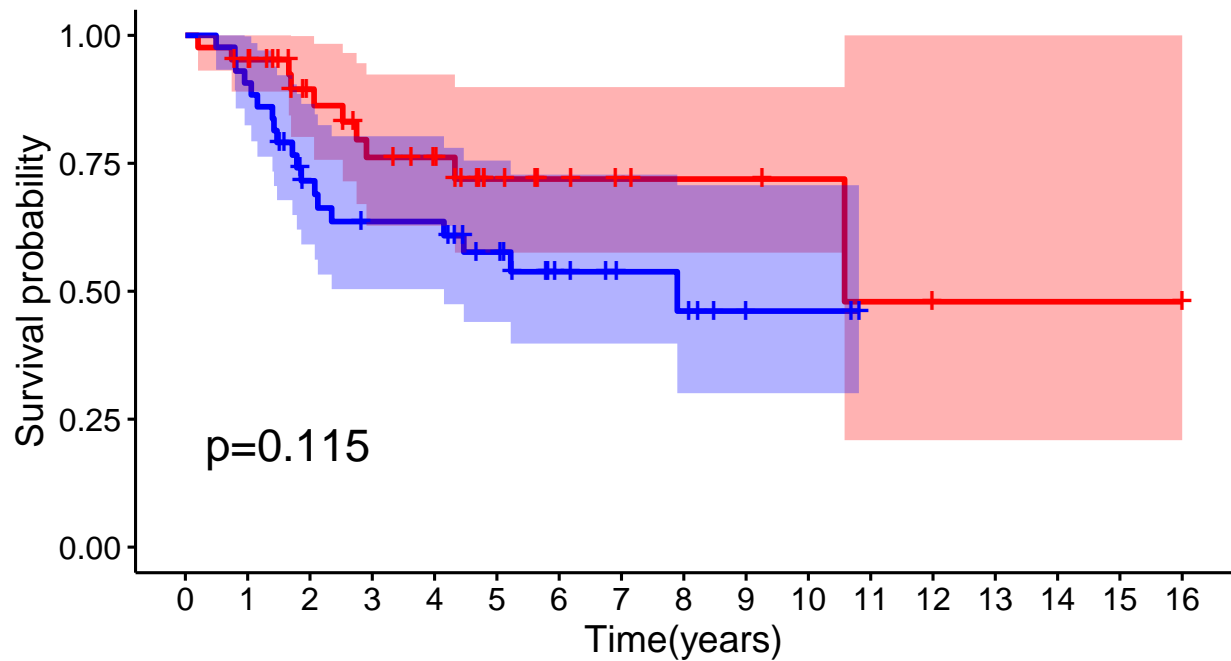

CLEC4A

high

low

|    |    |    |    |    |    |    |   |   |   |    |    |    |    |    |    |    |
|----|----|----|----|----|----|----|---|---|---|----|----|----|----|----|----|----|
| 42 | 39 | 28 | 22 | 19 | 11 | 8  | 6 | 5 | 5 | 3  | 2  | 1  | 1  | 1  | 1  | 1  |
| 43 | 39 | 27 | 23 | 23 | 17 | 10 | 7 | 6 | 2 | 2  | 0  | 0  | 0  | 0  | 0  | 0  |
| 0  | 1  | 2  | 3  | 4  | 5  | 6  | 7 | 8 | 9 | 10 | 11 | 12 | 13 | 14 | 15 | 16 |

Time(years)

Supplement: Supplementary Document 1 — Kaplan-Meier curve of the 518 genes associated with survival. [file DataSheet_1.zip › Supplementary Document 1/sur.CLEC4A.pdf]

CLIC5 + high + low

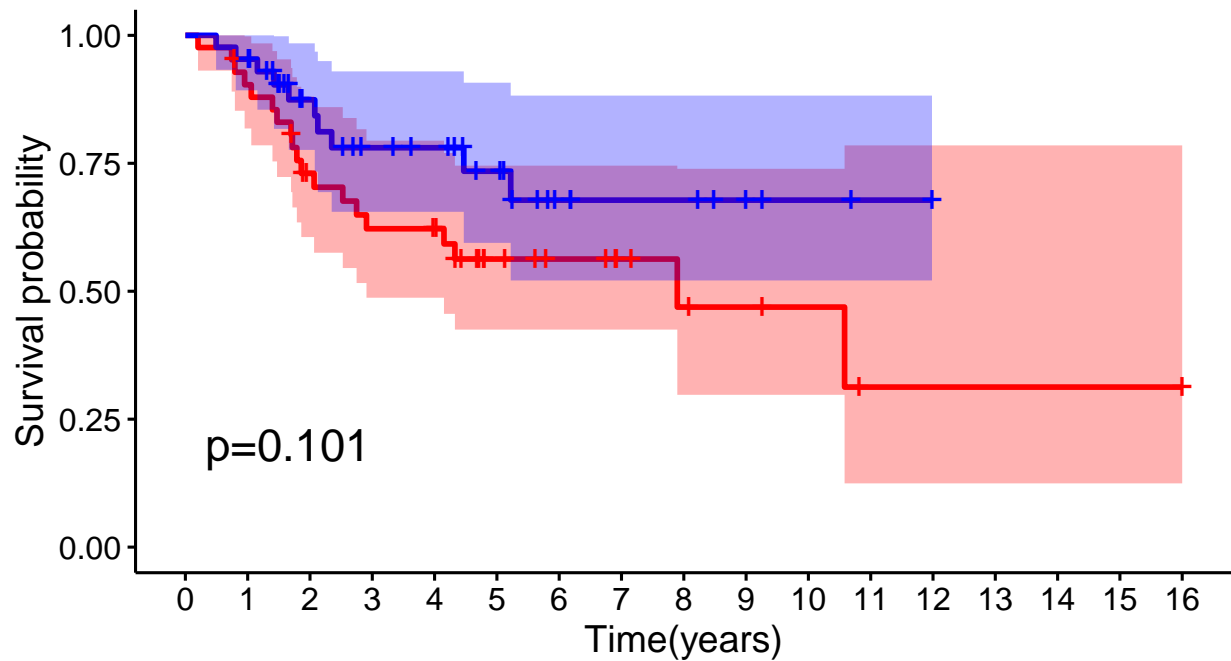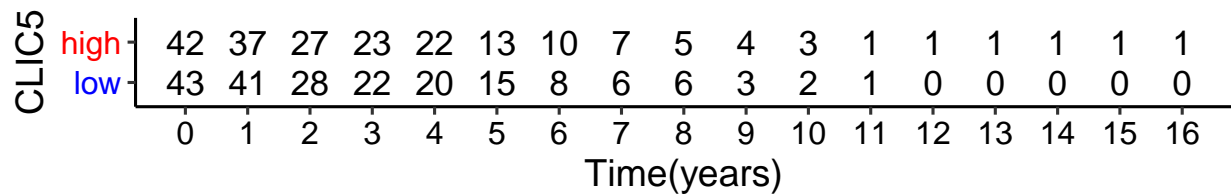

Supplement: Supplementary Document 1 — Kaplan-Meier curve of the 518 genes associated with survival. [file DataSheet_1.zip › Supplementary Document 1/sur.CLIC5.pdf]

CLIC6 + high + low

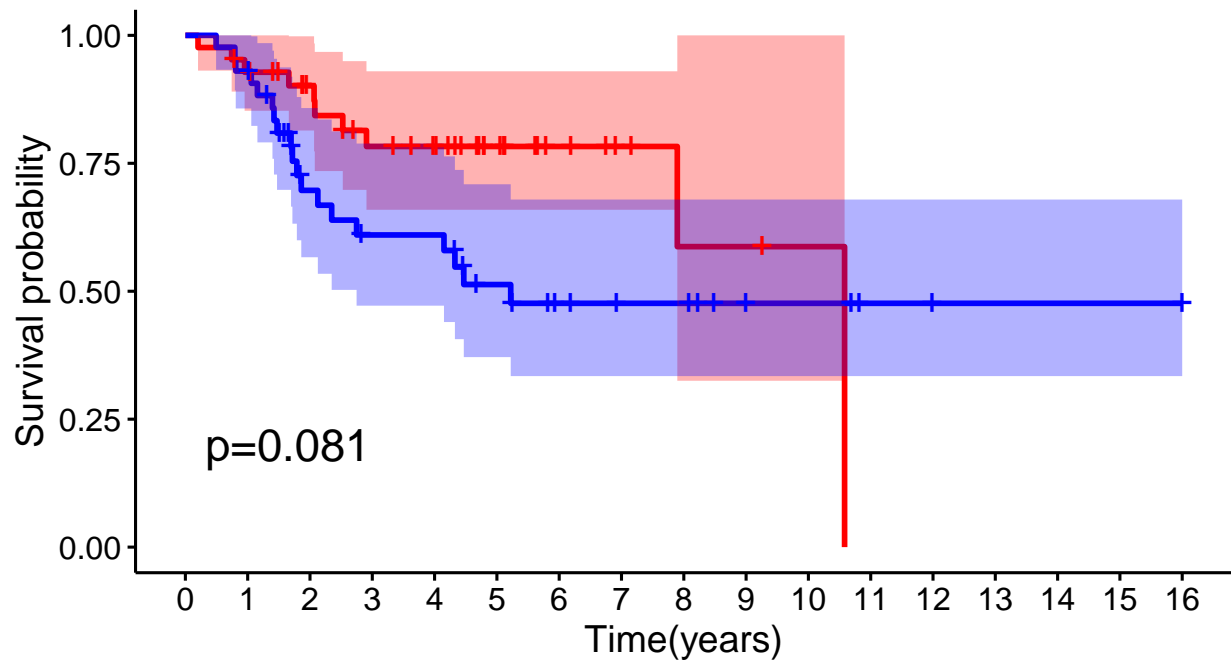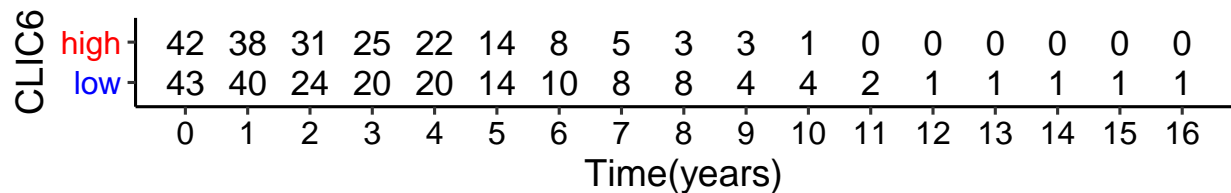

Supplement: Supplementary Document 1 — Kaplan-Meier curve of the 518 genes associated with survival. [file DataSheet_1.zip › Supplementary Document 1/sur.CLIC6.pdf]

CNNM2 + high + low

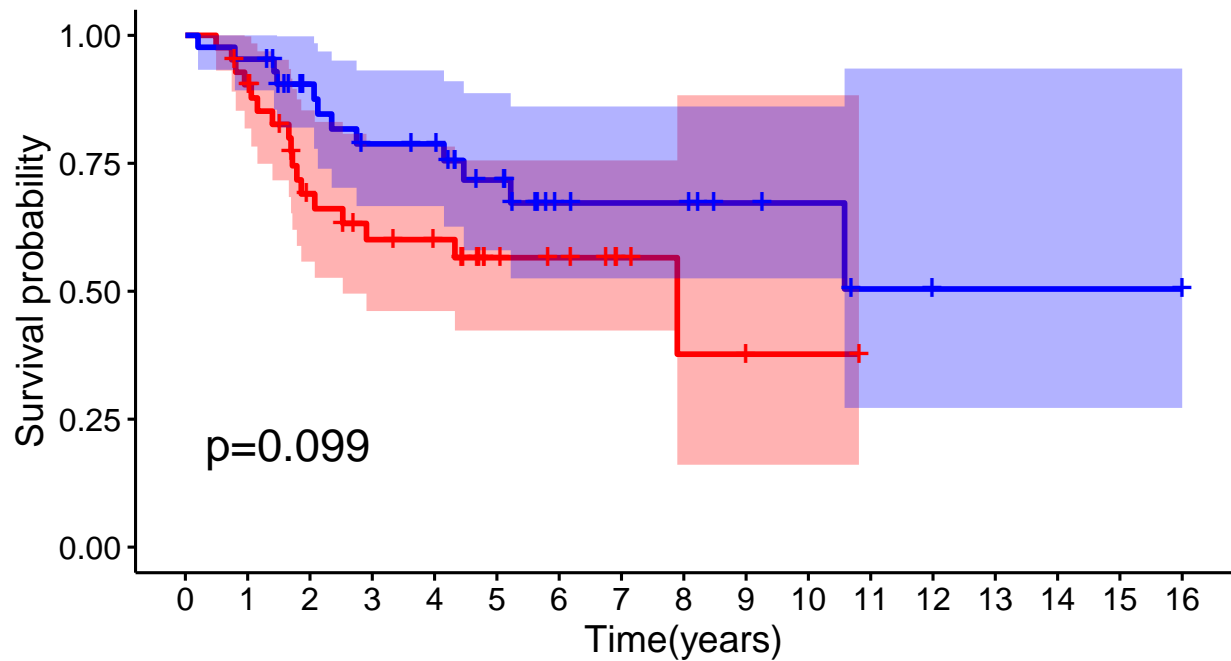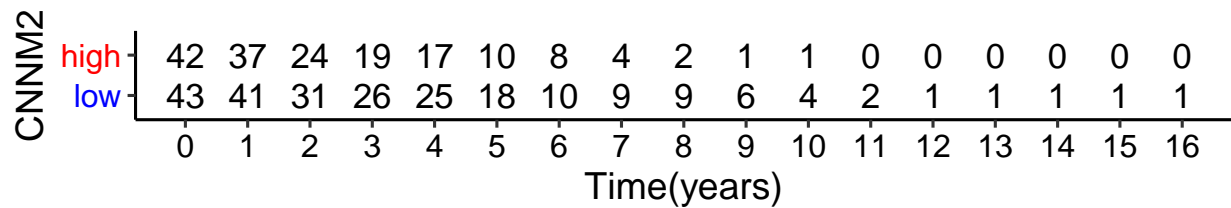

Supplement: Supplementary Document 1 — Kaplan-Meier curve of the 518 genes associated with survival. [file DataSheet_1.zip › Supplementary Document 1/sur.CNNM2.pdf]

COBL high low

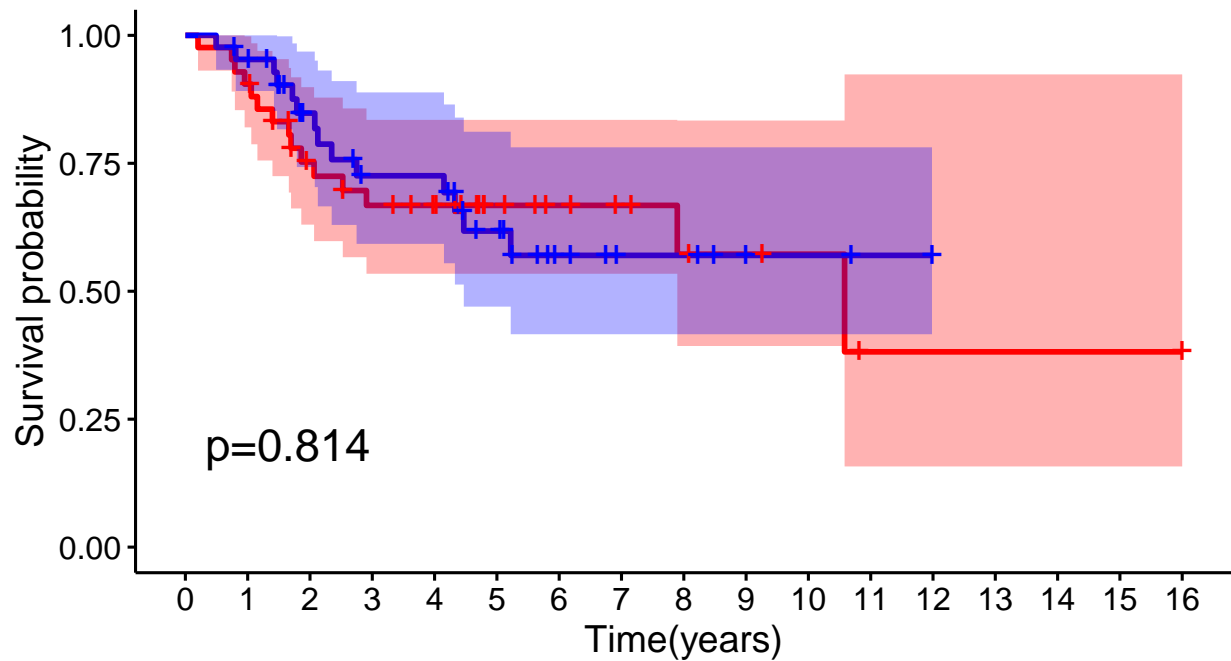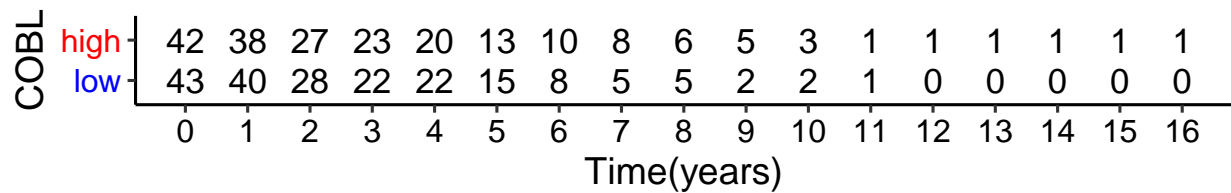

Supplement: Supplementary Document 1 — Kaplan-Meier curve of the 518 genes associated with survival. [file DataSheet_1.zip › Supplementary Document 1/sur.COBL.pdf]

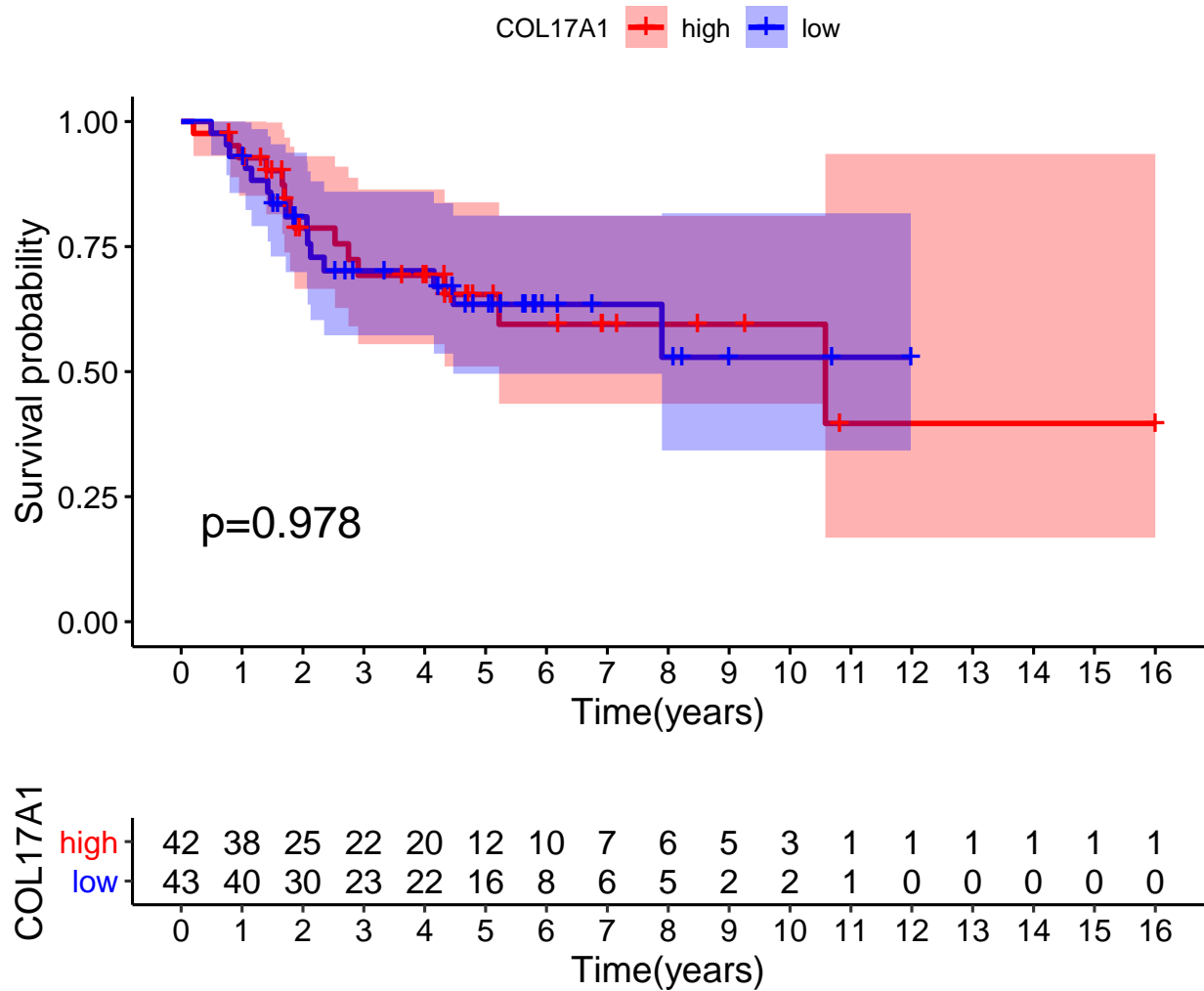

Supplement: Supplementary Document 1 — Kaplan-Meier curve of the 518 genes associated with survival. [file DataSheet_1.zip › Supplementary Document 1/sur.COL17A1.pdf]

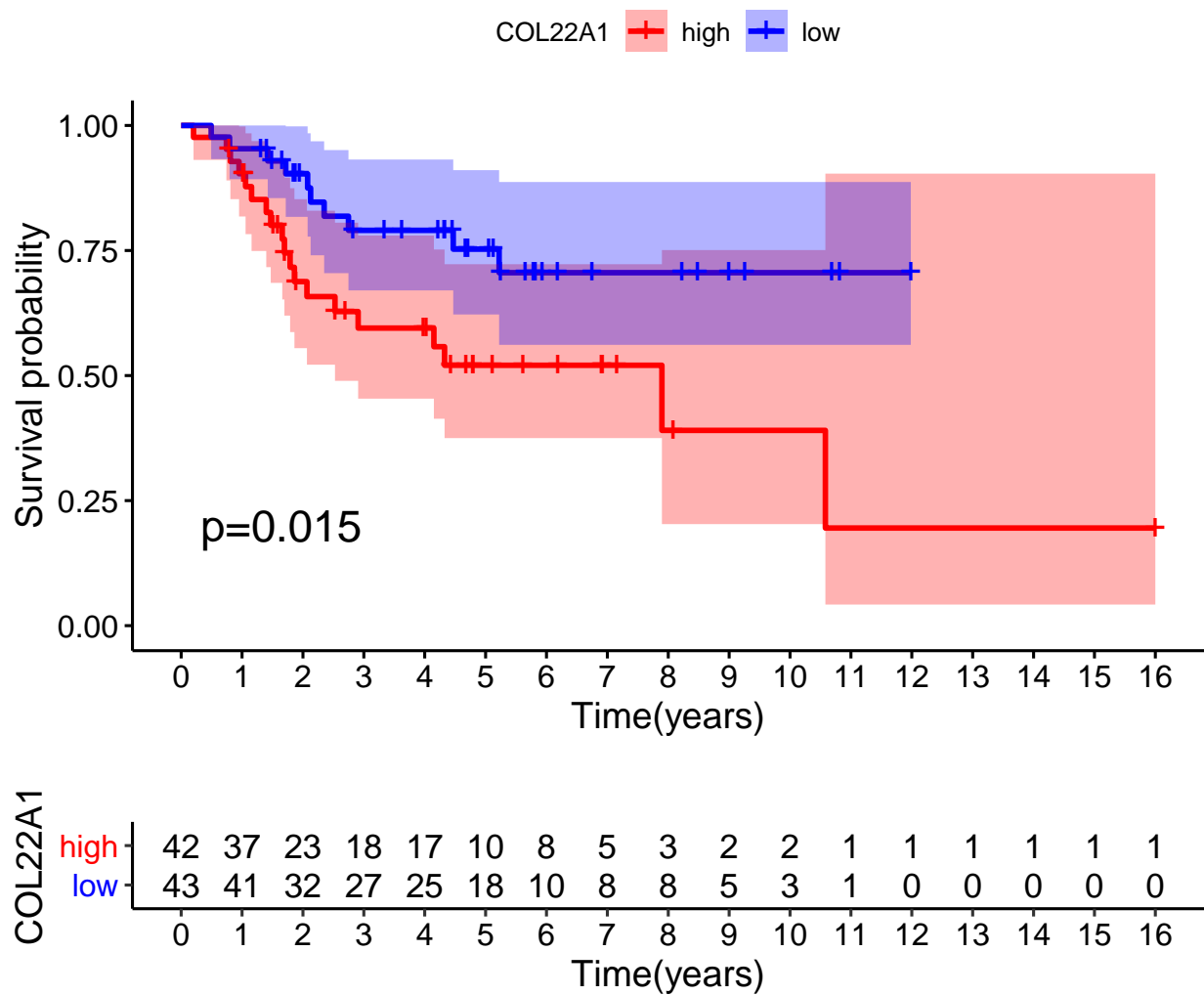

Supplement: Supplementary Document 1 — Kaplan-Meier curve of the 518 genes associated with survival. [file DataSheet_1.zip › Supplementary Document 1/sur.COL22A1.pdf]

COL4A3 + high + low

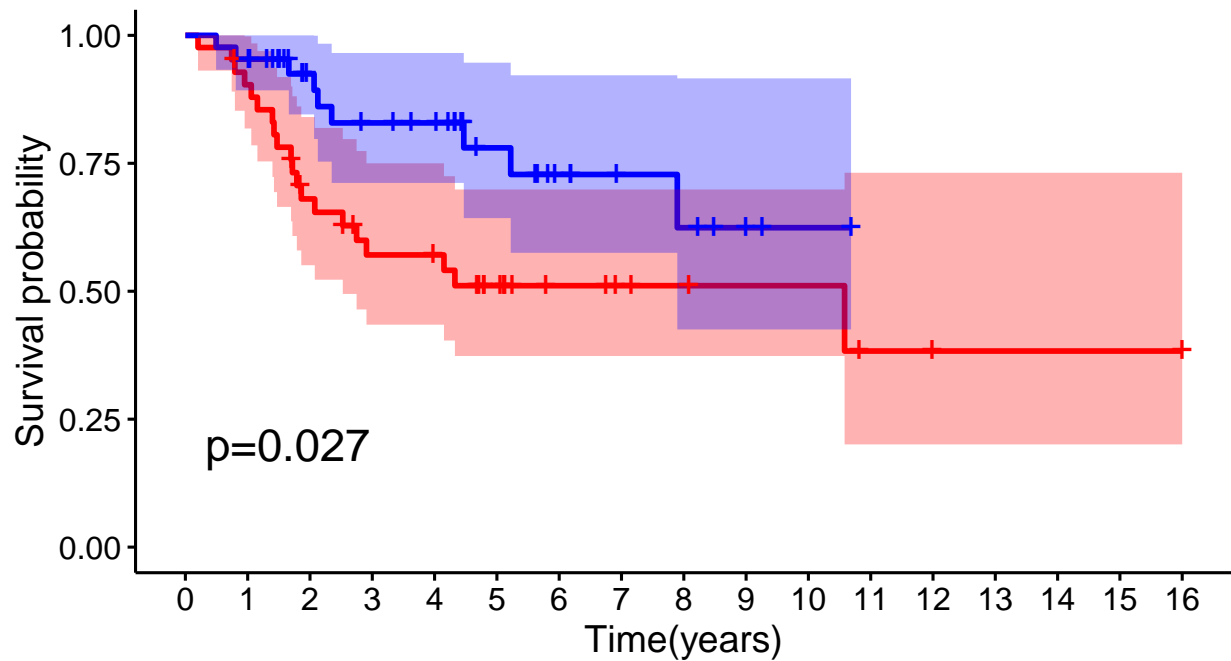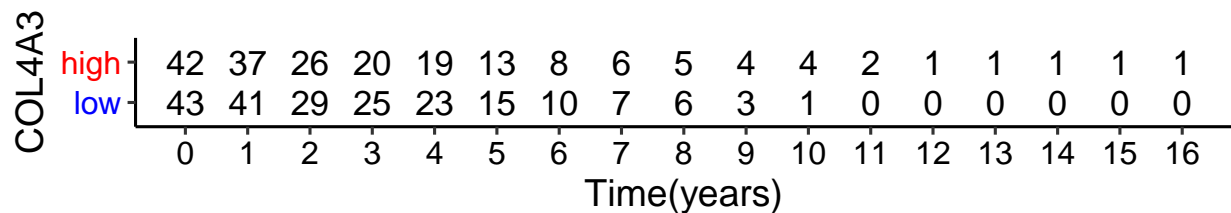

Supplement: Supplementary Document 1 — Kaplan-Meier curve of the 518 genes associated with survival. [file DataSheet_1.zip › Supplementary Document 1/sur.COL4A3.pdf]

COL4A4 + high + low

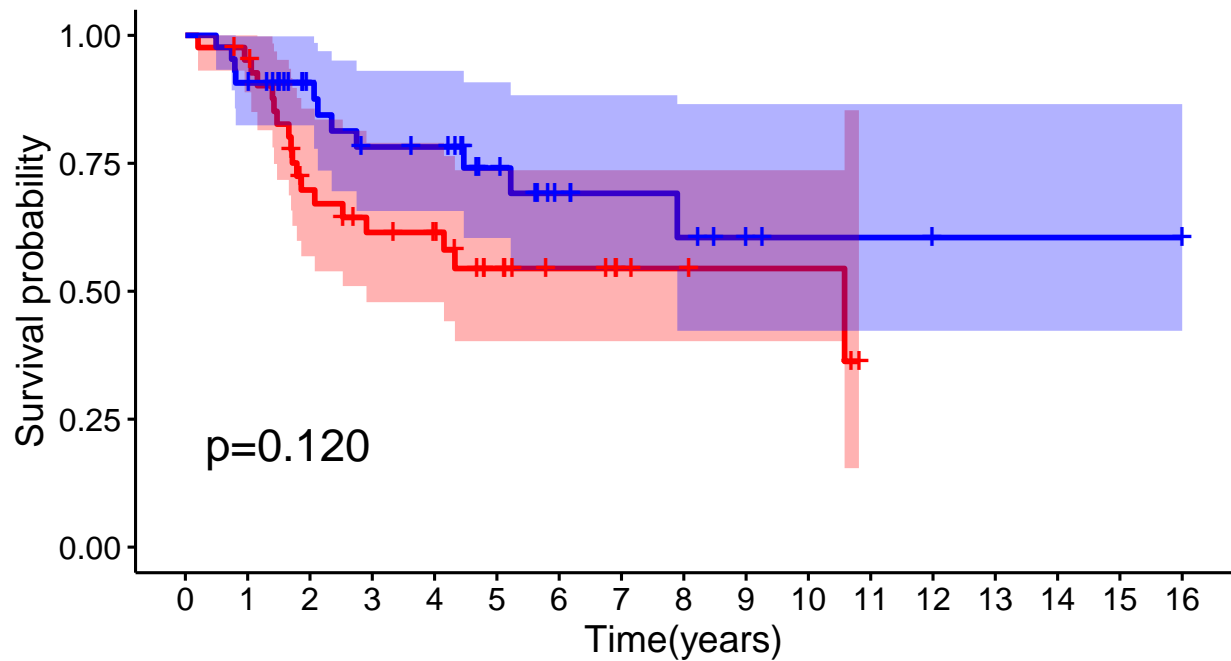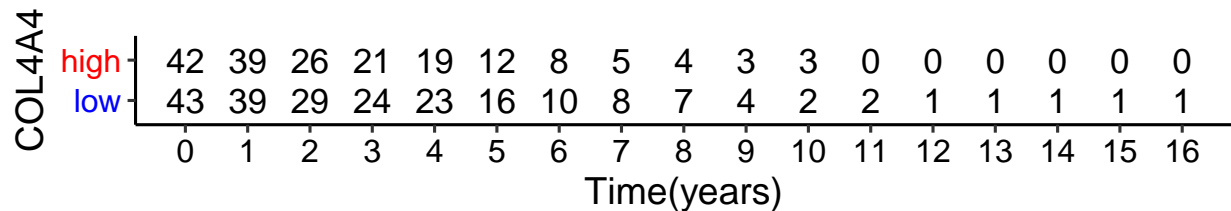

Supplement: Supplementary Document 1 — Kaplan-Meier curve of the 518 genes associated with survival. [file DataSheet_1.zip › Supplementary Document 1/sur.COL4A4.pdf]

CPE + high - low

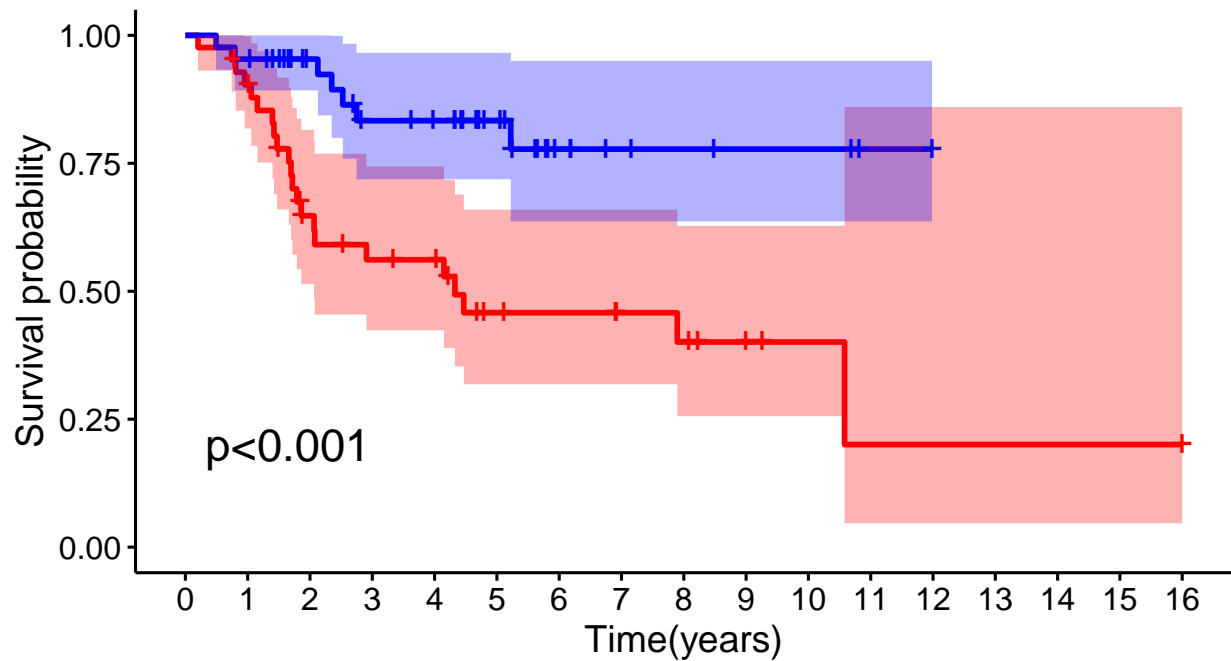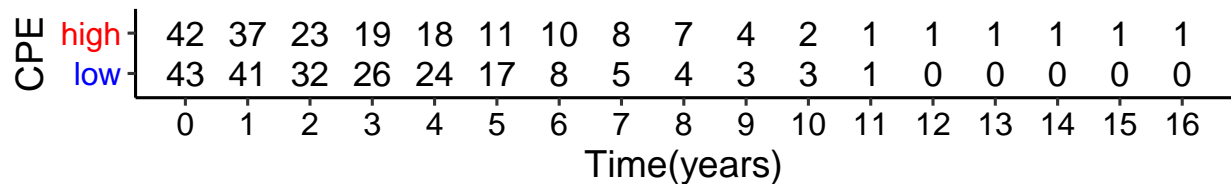

Supplement: Supplementary Document 1 — Kaplan-Meier curve of the 518 genes associated with survival. [file DataSheet_1.zip › Supplementary Document 1/sur.CPE.pdf]

CPN2 + high + low

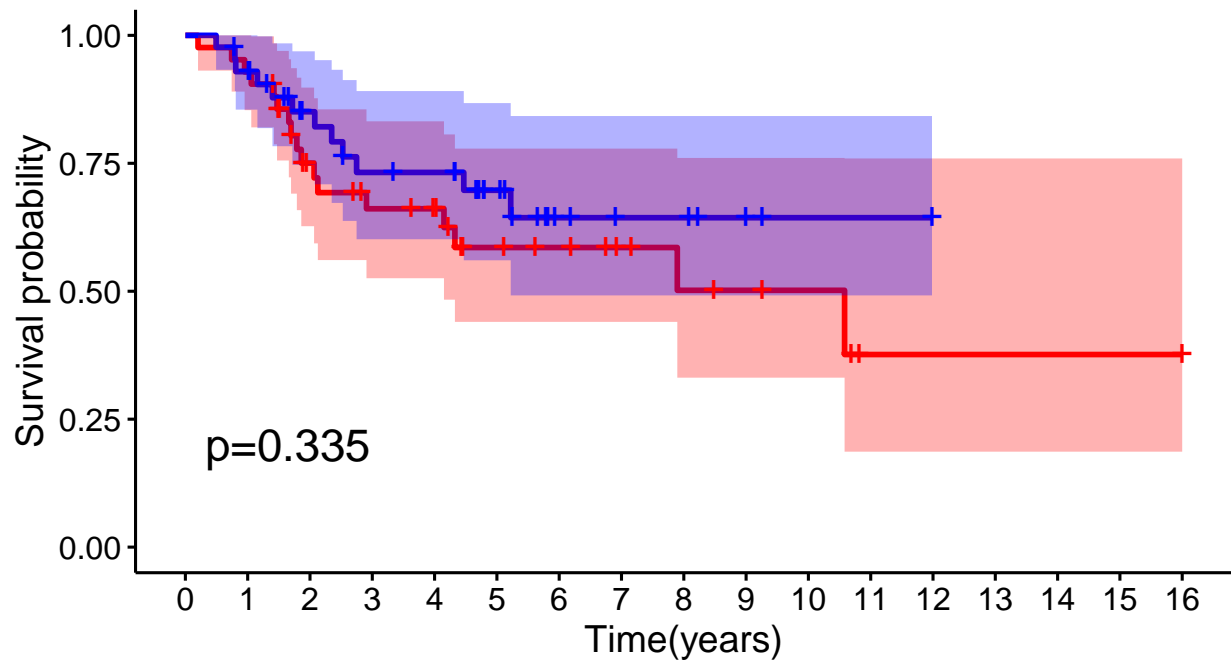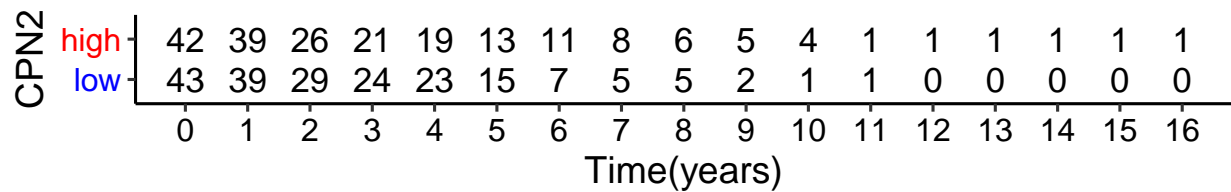

Supplement: Supplementary Document 1 — Kaplan-Meier curve of the 518 genes associated with survival. [file DataSheet_1.zip › Supplementary Document 1/sur.CPN2.pdf]

CSTB + high + low

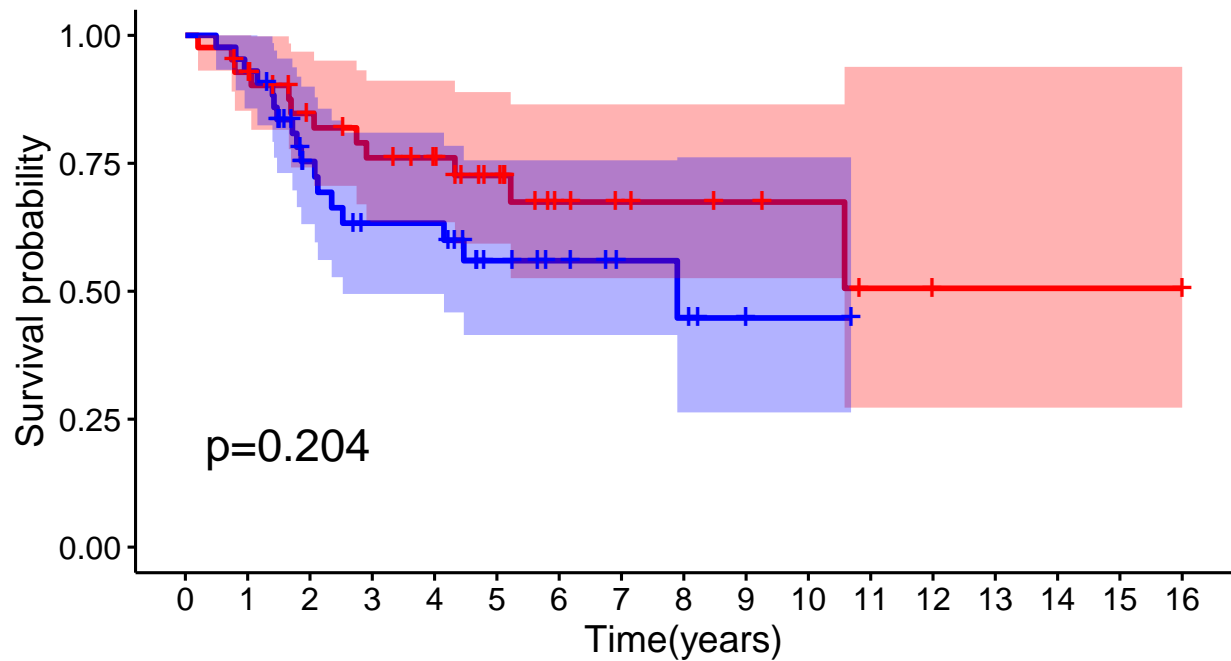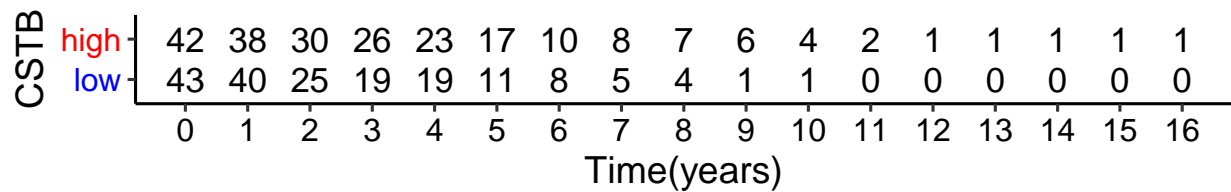

Supplement: Supplementary Document 1 — Kaplan-Meier curve of the 518 genes associated with survival. [file DataSheet_1.zip › Supplementary Document 1/sur.CSTB.pdf]

CTH + high + low

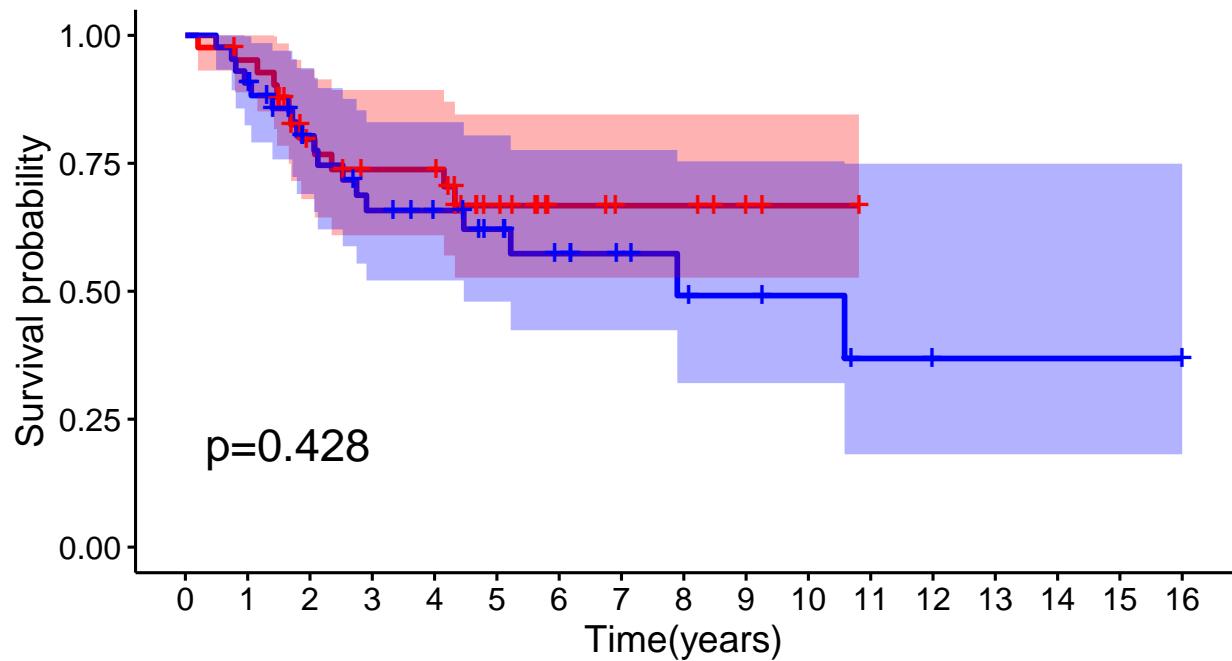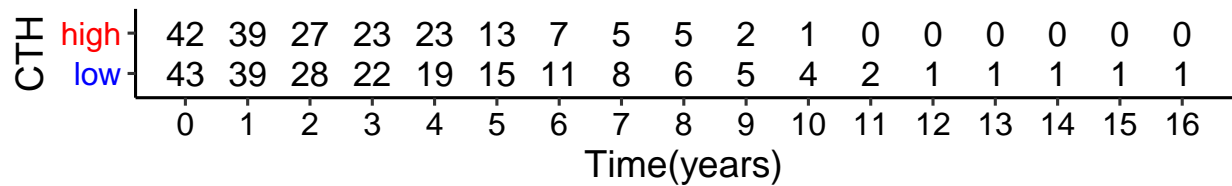

Supplement: Supplementary Document 1 — Kaplan-Meier curve of the 518 genes associated with survival. [file DataSheet_1.zip › Supplementary Document 1/sur.CTH.pdf]

CTSK + high + low

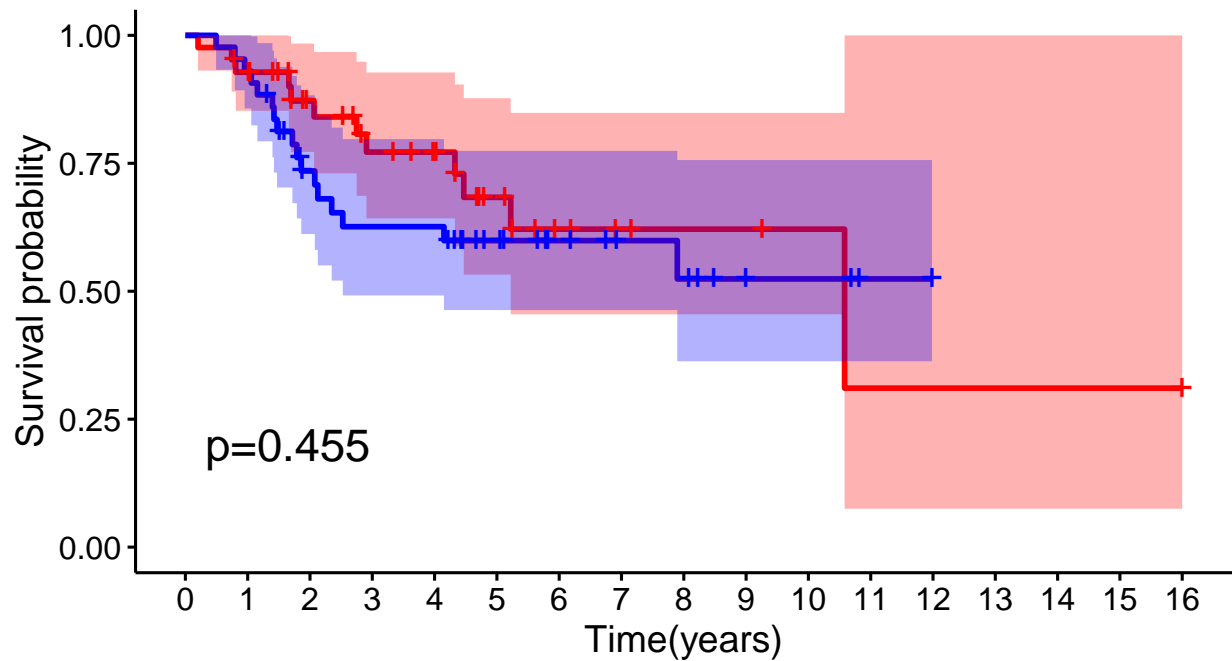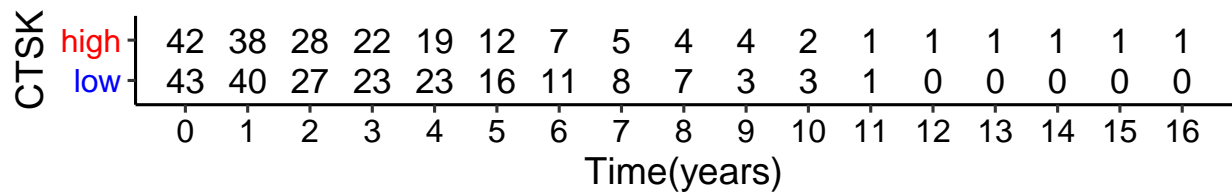

Supplement: Supplementary Document 1 — Kaplan-Meier curve of the 518 genes associated with survival. [file DataSheet_1.zip › Supplementary Document 1/sur.CTSK.pdf]

CXADR + high + low

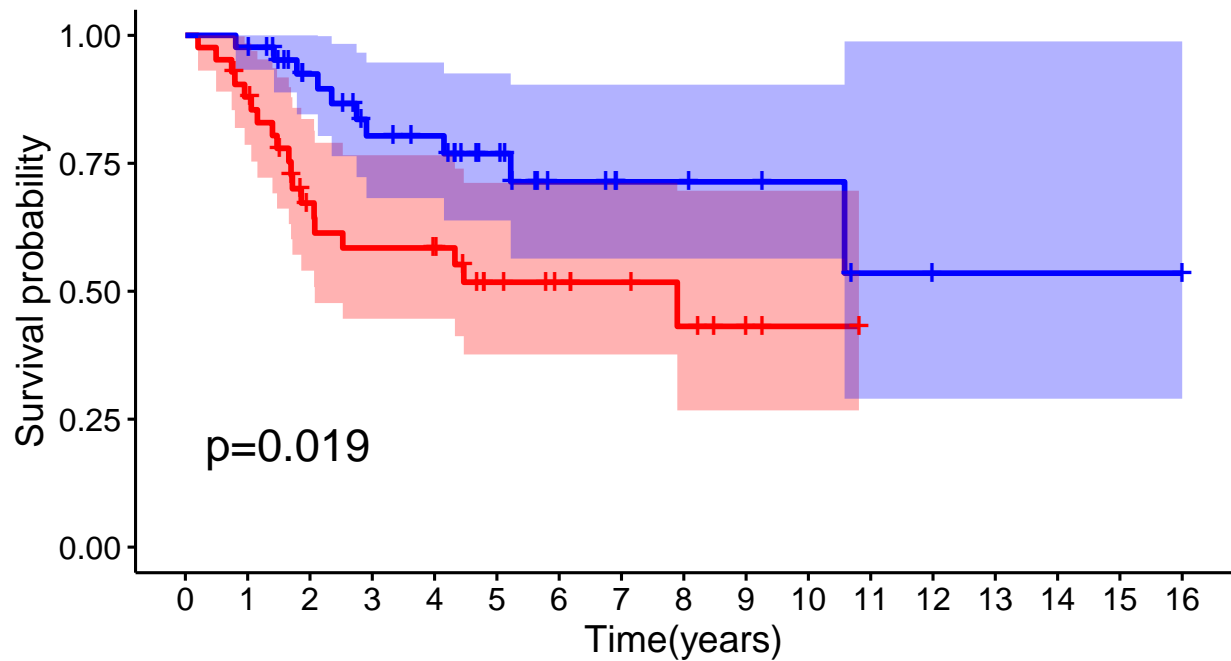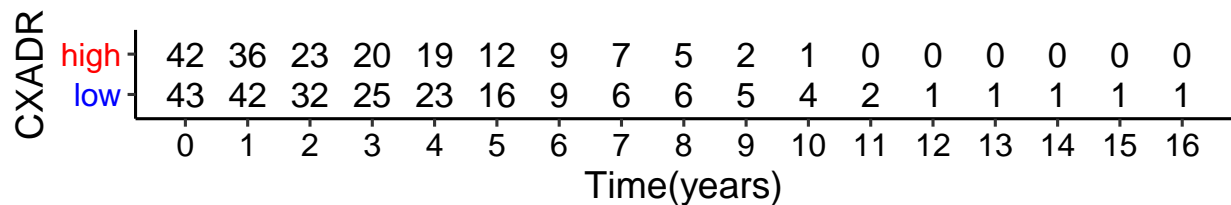

Supplement: Supplementary Document 1 — Kaplan-Meier curve of the 518 genes associated with survival. [file DataSheet_1.zip › Supplementary Document 1/sur.CXADR.pdf]

CYFIP2 high low

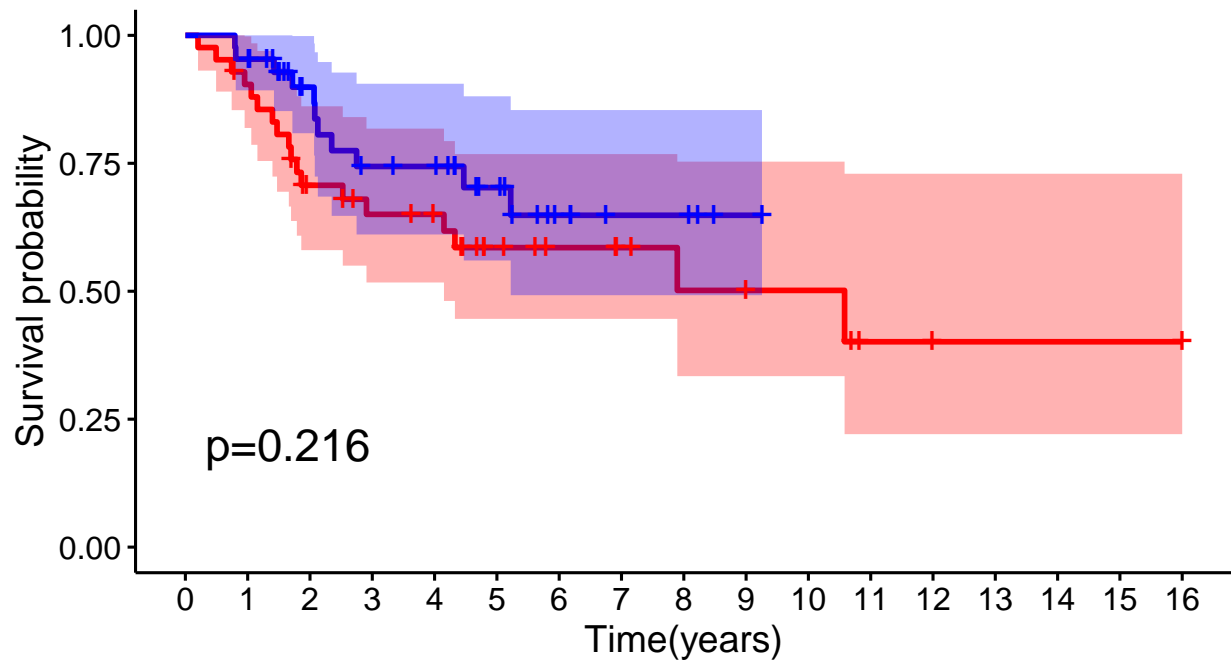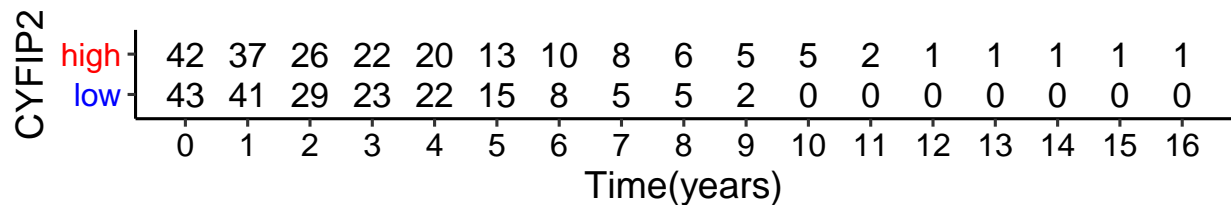

Supplement: Supplementary Document 1 — Kaplan-Meier curve of the 518 genes associated with survival. [file DataSheet_1.zip › Supplementary Document 1/sur.CYFIP2.pdf]

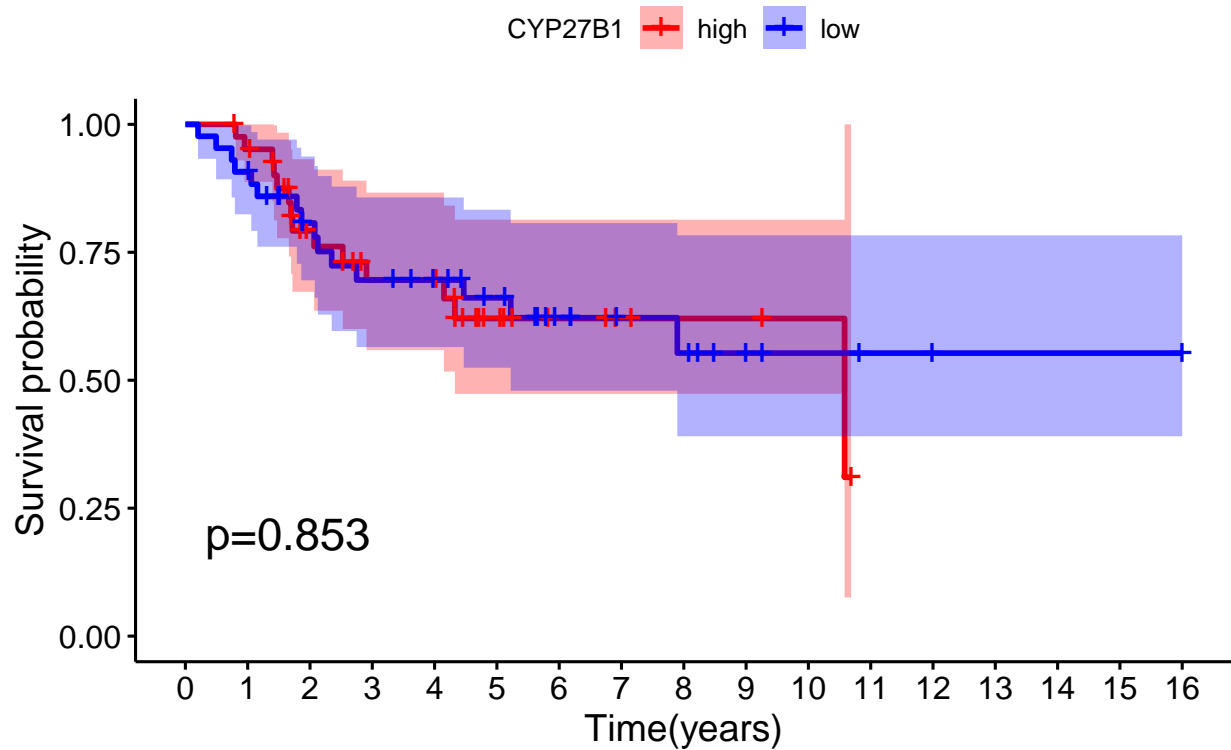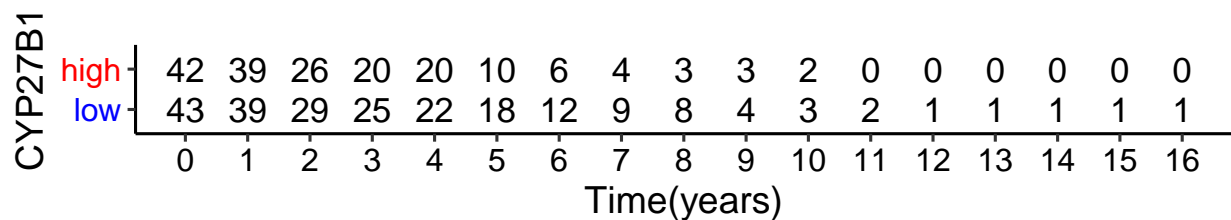

Supplement: Supplementary Document 1 — Kaplan-Meier curve of the 518 genes associated with survival. [file DataSheet_1.zip › Supplementary Document 1/sur.CYP27B1.pdf]

DAPL1 + high + low

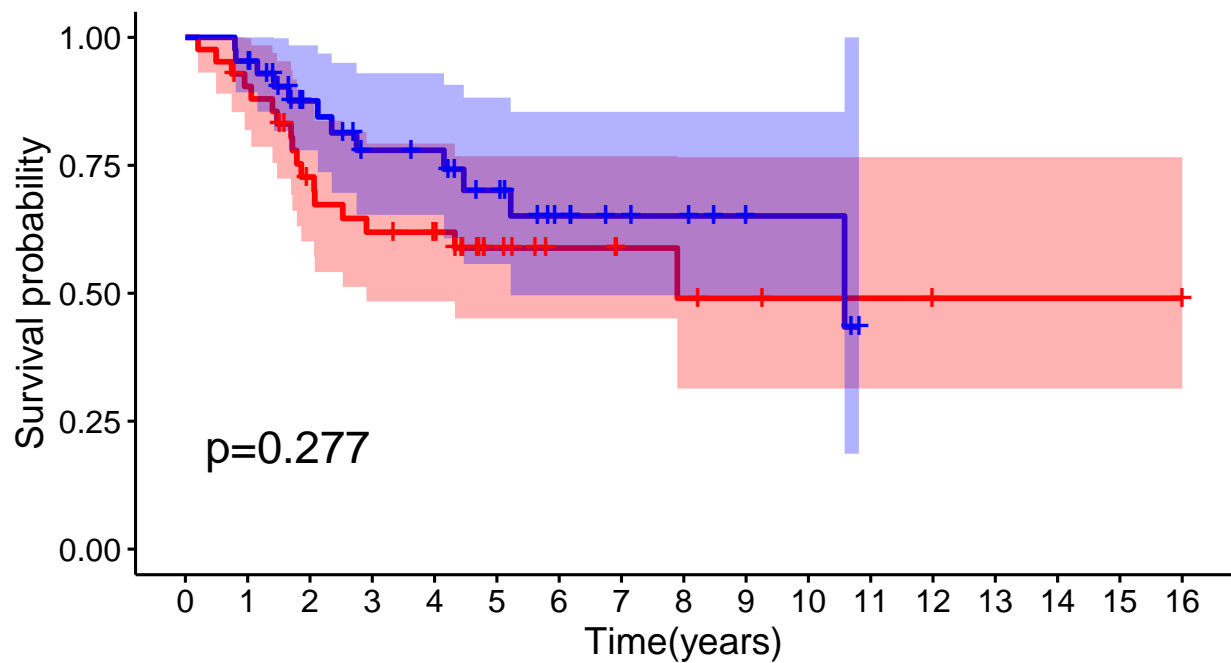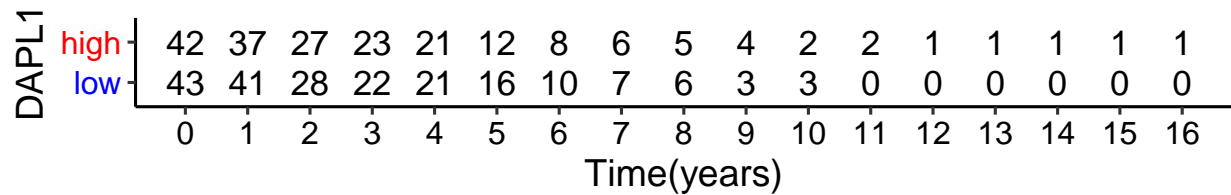

Supplement: Supplementary Document 1 — Kaplan-Meier curve of the 518 genes associated with survival. [file DataSheet_1.zip › Supplementary Document 1/sur.DAPL1.pdf]

DBNDD2 high low

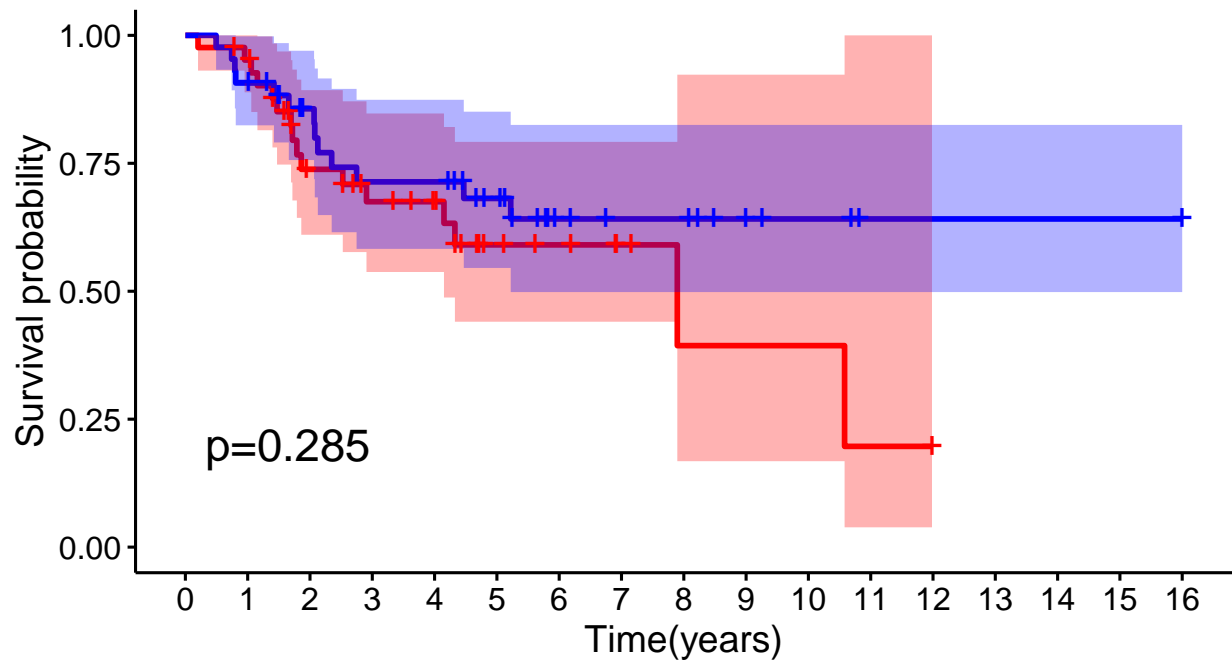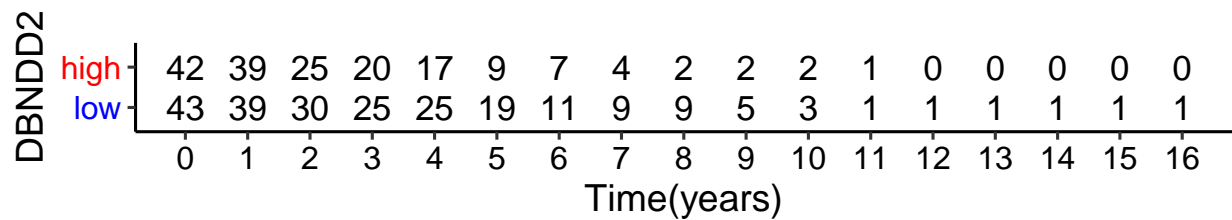

Supplement: Supplementary Document 1 — Kaplan-Meier curve of the 518 genes associated with survival. [file DataSheet_1.zip › Supplementary Document 1/sur.DBNDD2.pdf]

DCAF13 high low

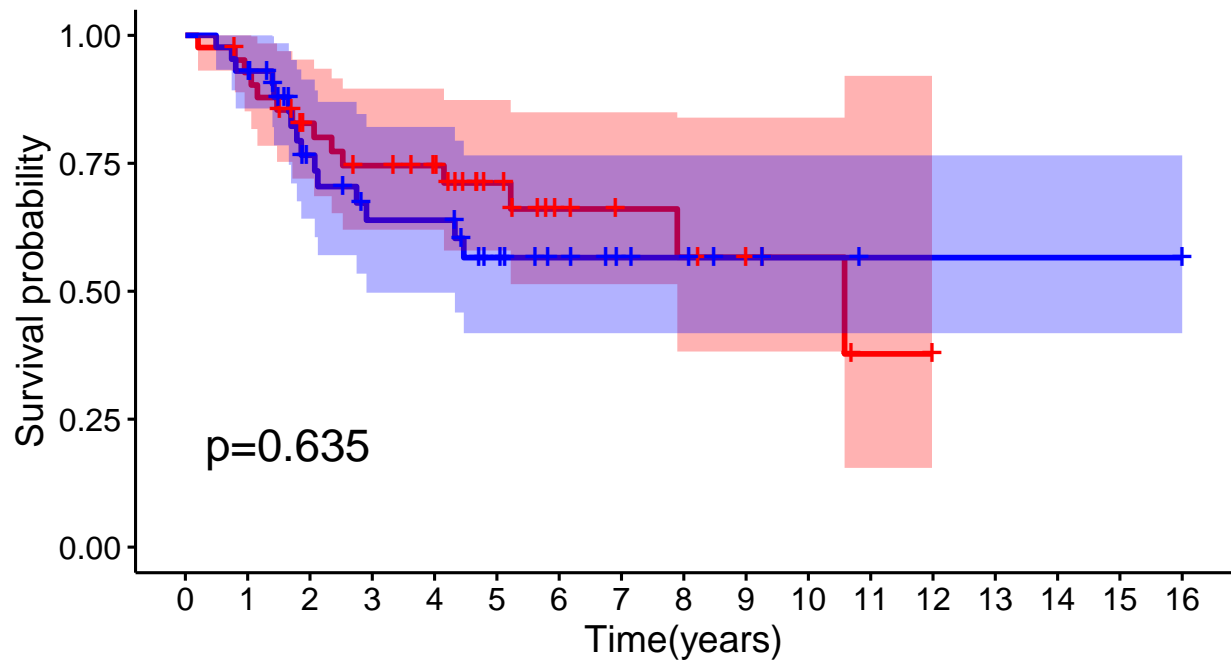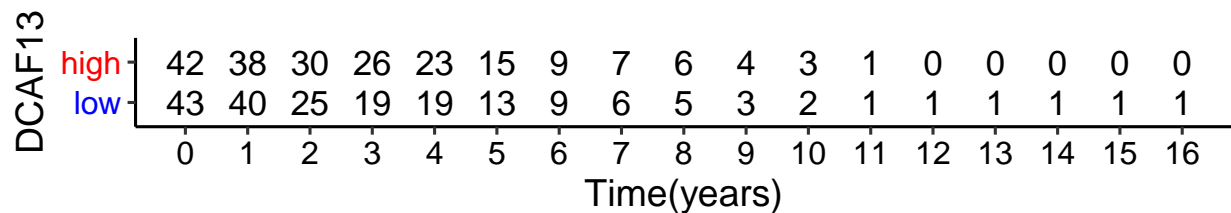

Supplement: Supplementary Document 1 — Kaplan-Meier curve of the 518 genes associated with survival. [file DataSheet_1.zip › Supplementary Document 1/sur.DCAF13.pdf]

DCSTAMP + high + low

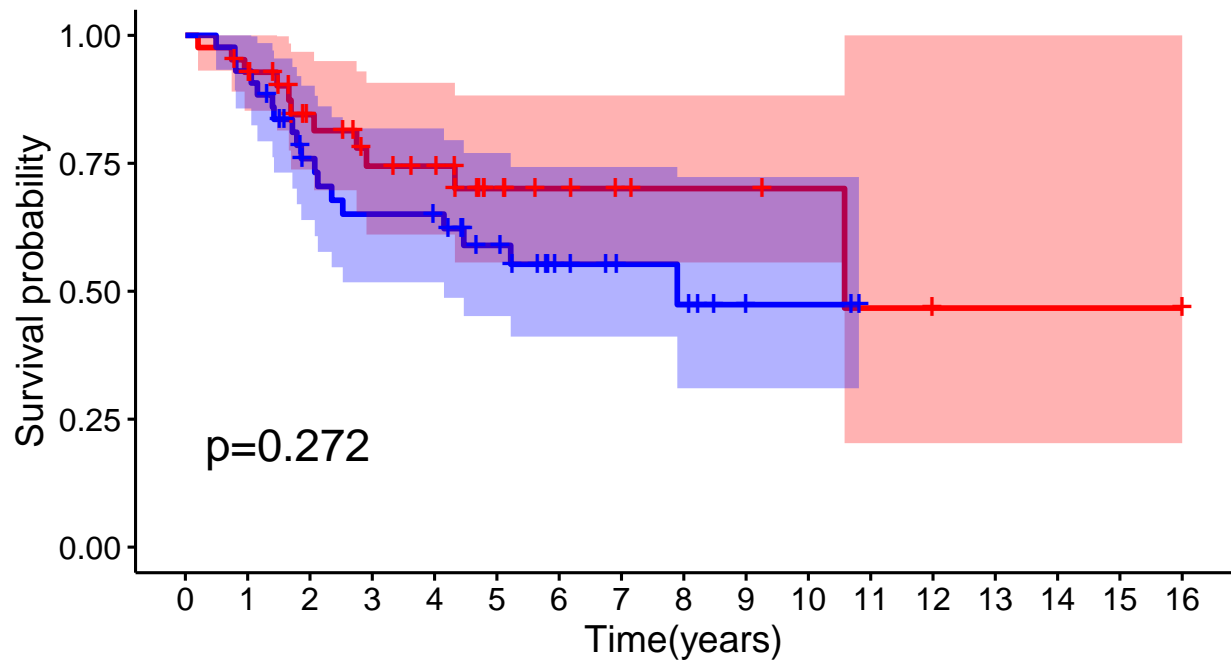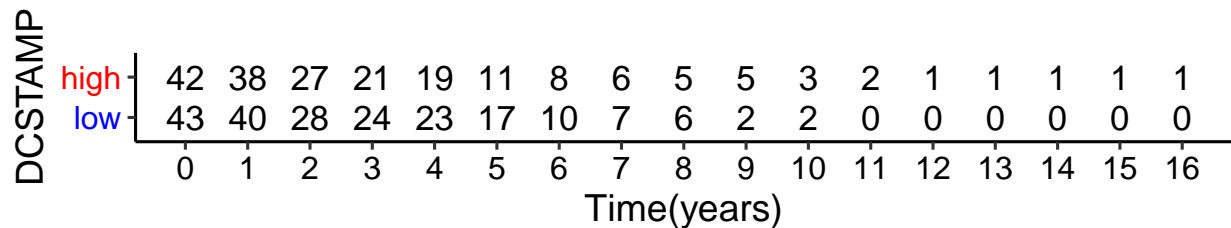

Supplement: Supplementary Document 1 — Kaplan-Meier curve of the 518 genes associated with survival. [file DataSheet_1.zip › Supplementary Document 1/sur.DCSTAMP.pdf]

DIRAS2 high low

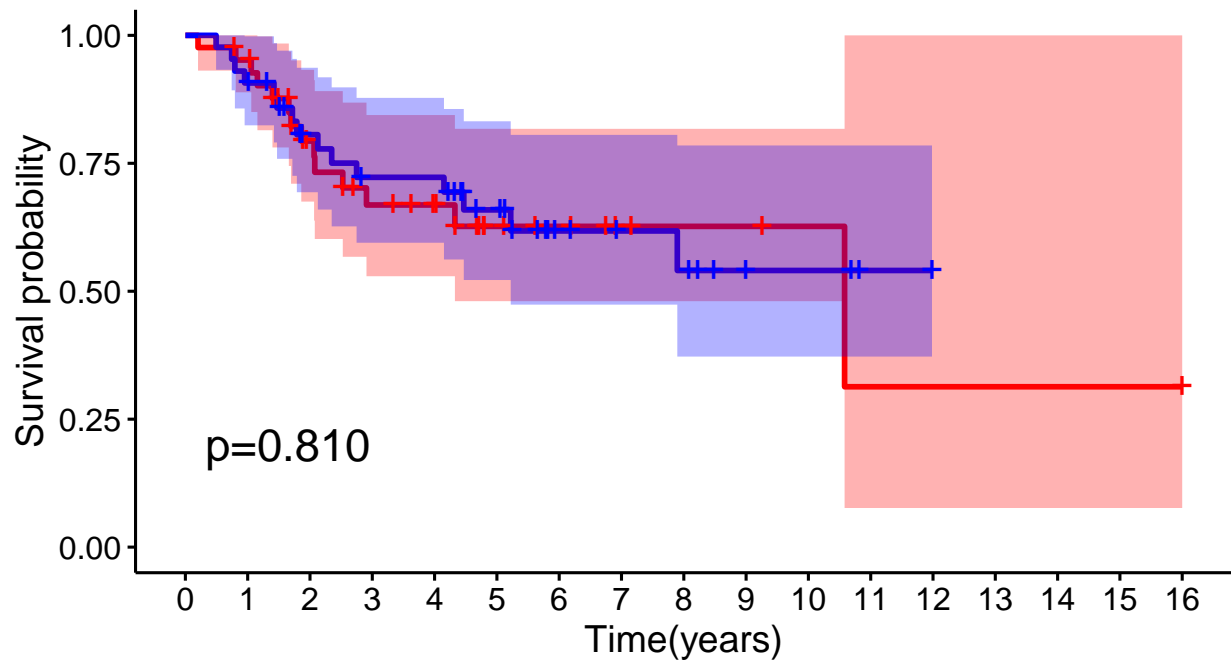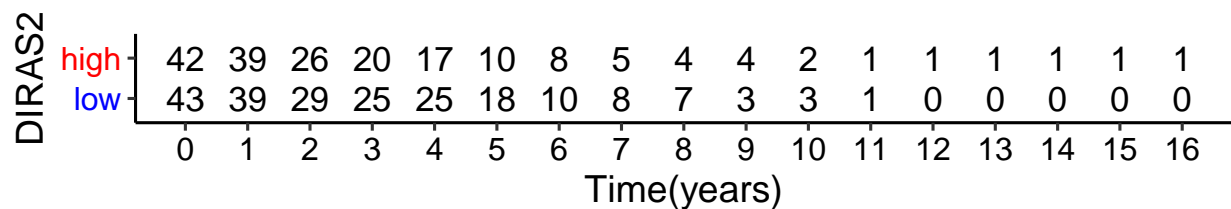

Supplement: Supplementary Document 1 — Kaplan-Meier curve of the 518 genes associated with survival. [file DataSheet_1.zip › Supplementary Document 1/sur.DIRAS2.pdf]

DKK1 + high + low

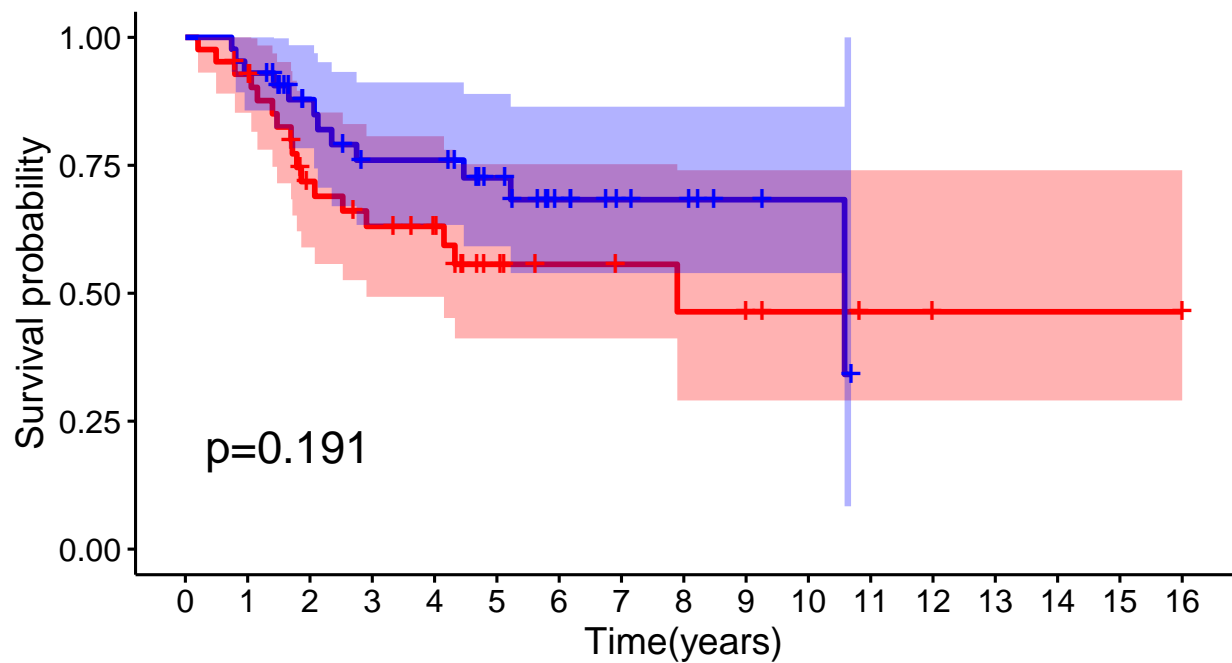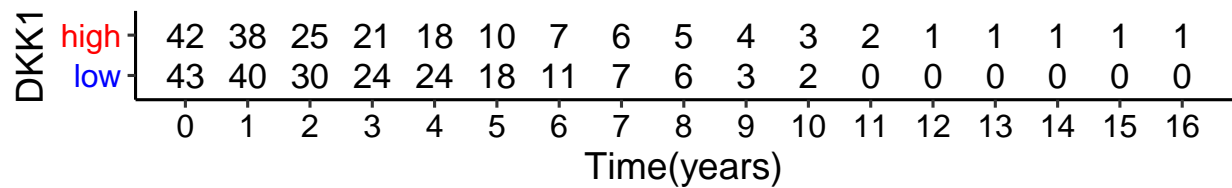

Supplement: Supplementary Document 1 — Kaplan-Meier curve of the 518 genes associated with survival. [file DataSheet_1.zip › Supplementary Document 1/sur.DKK1.pdf]

DLX2 + high + low

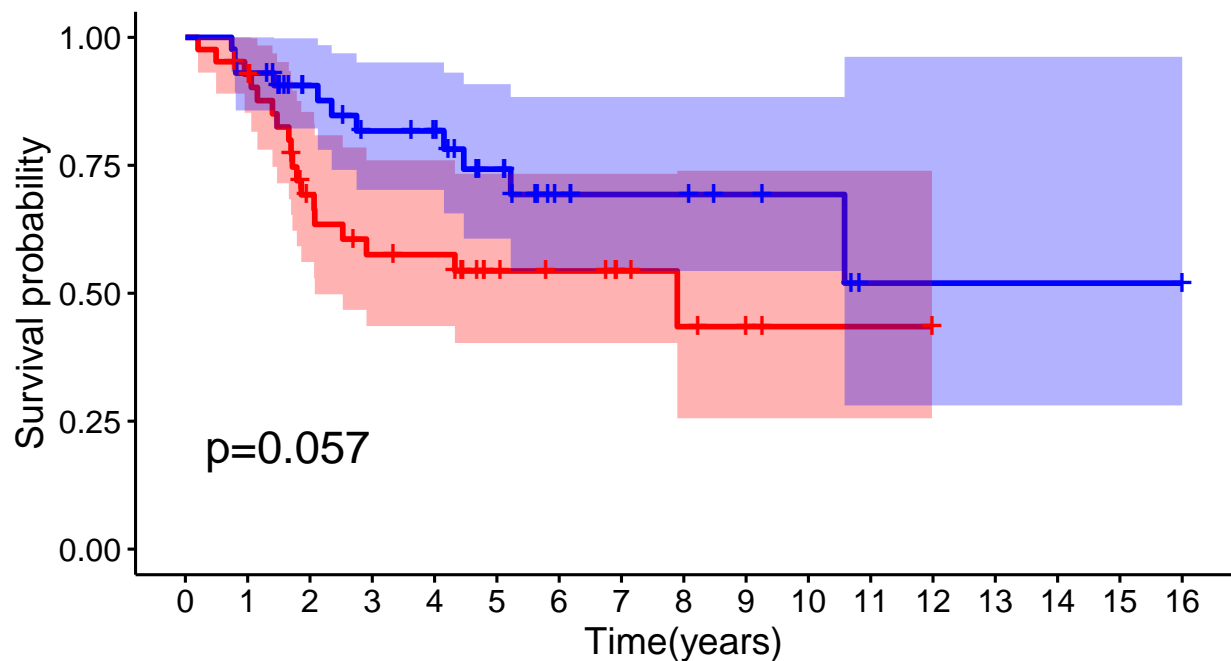

DLX2

|      |    |    |    |    |    |    |   |   |   |   |    |    |    |    |    |    |    |
|------|----|----|----|----|----|----|---|---|---|---|----|----|----|----|----|----|----|
| high | 42 | 38 | 24 | 19 | 18 | 11 | 9 | 6 | 4 | 2 | 1  | 1  | 0  | 0  | 0  | 0  | 0  |
| low  | 43 | 40 | 31 | 26 | 24 | 17 | 9 | 7 | 7 | 5 | 4  | 1  | 1  | 1  | 1  | 1  | 1  |
|      | 0  | 1  | 2  | 3  | 4  | 5  | 6 | 7 | 8 | 9 | 10 | 11 | 12 | 13 | 14 | 15 | 16 |

Time(years)

Supplement: Supplementary Document 1 — Kaplan-Meier curve of the 518 genes associated with survival. [file DataSheet_1.zip › Supplementary Document 1/sur.DLX2.pdf]

DMP1 + high + low

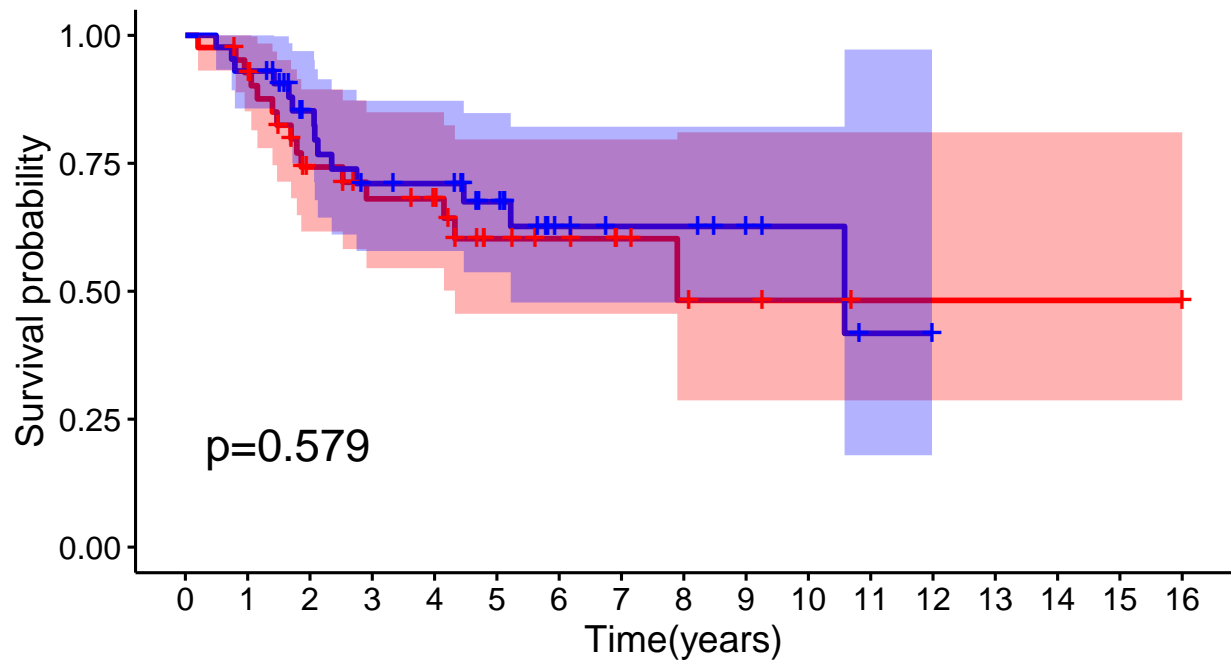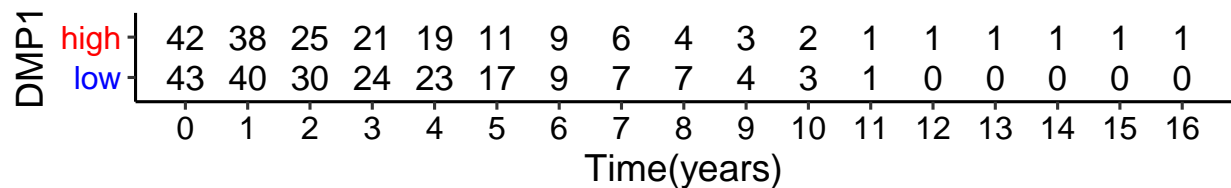

Supplement: Supplementary Document 1 — Kaplan-Meier curve of the 518 genes associated with survival. [file DataSheet_1.zip › Supplementary Document 1/sur.DMP1.pdf]

DMRT2 high low

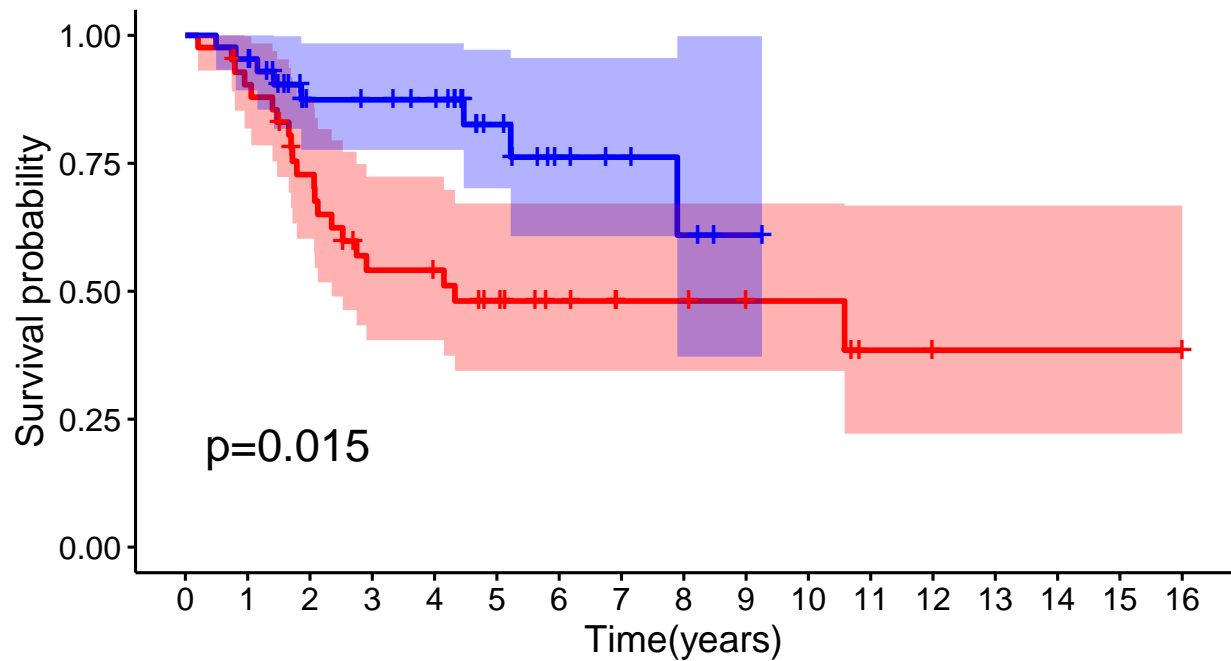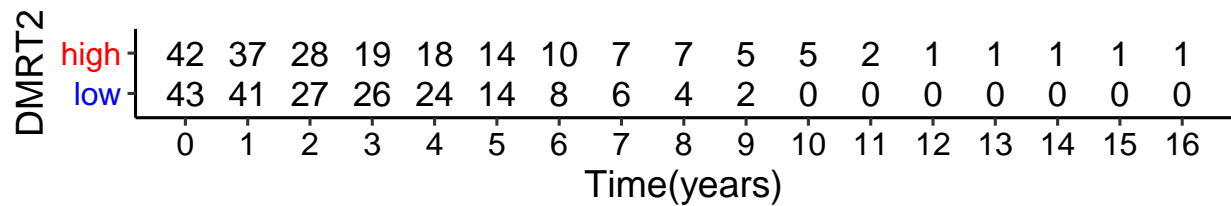

Supplement: Supplementary Document 1 — Kaplan-Meier curve of the 518 genes associated with survival. [file DataSheet_1.zip › Supplementary Document 1/sur.DMRT2.pdf]

DNAAF4 high low

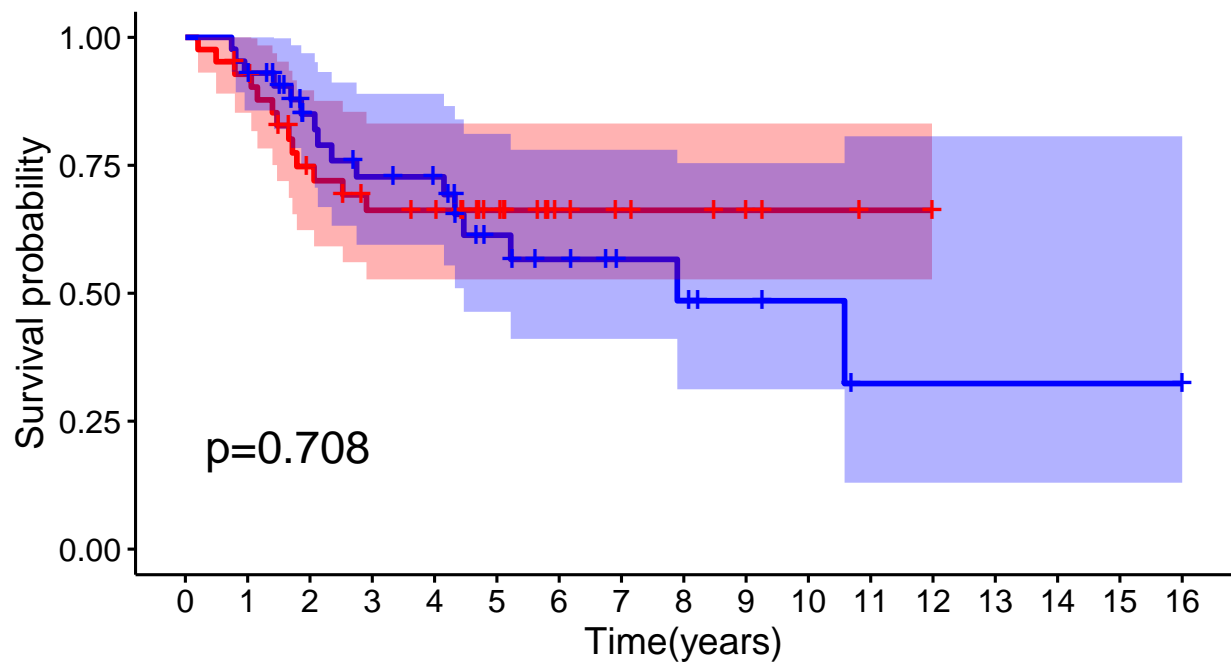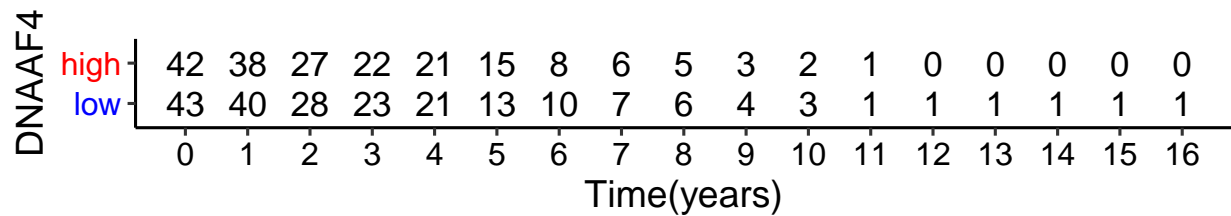

Supplement: Supplementary Document 1 — Kaplan-Meier curve of the 518 genes associated with survival. [file DataSheet_1.zip › Supplementary Document 1/sur.DNAAF4.pdf]

DNAH6 + high + low

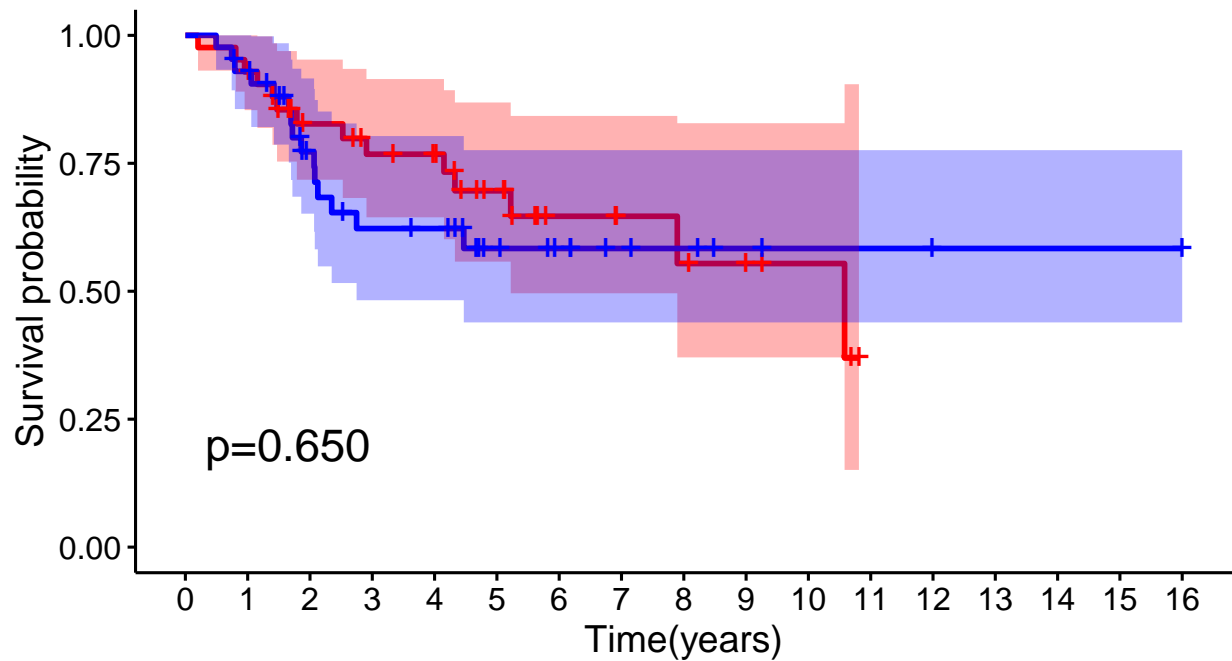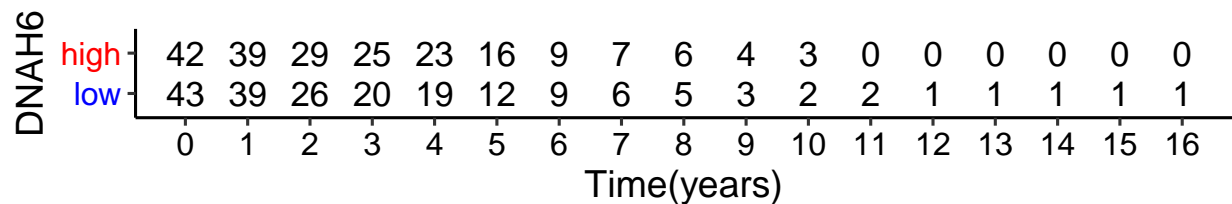

Supplement: Supplementary Document 1 — Kaplan-Meier curve of the 518 genes associated with survival. [file DataSheet_1.zip › Supplementary Document 1/sur.DNAH6.pdf]

ABLIM1 high low

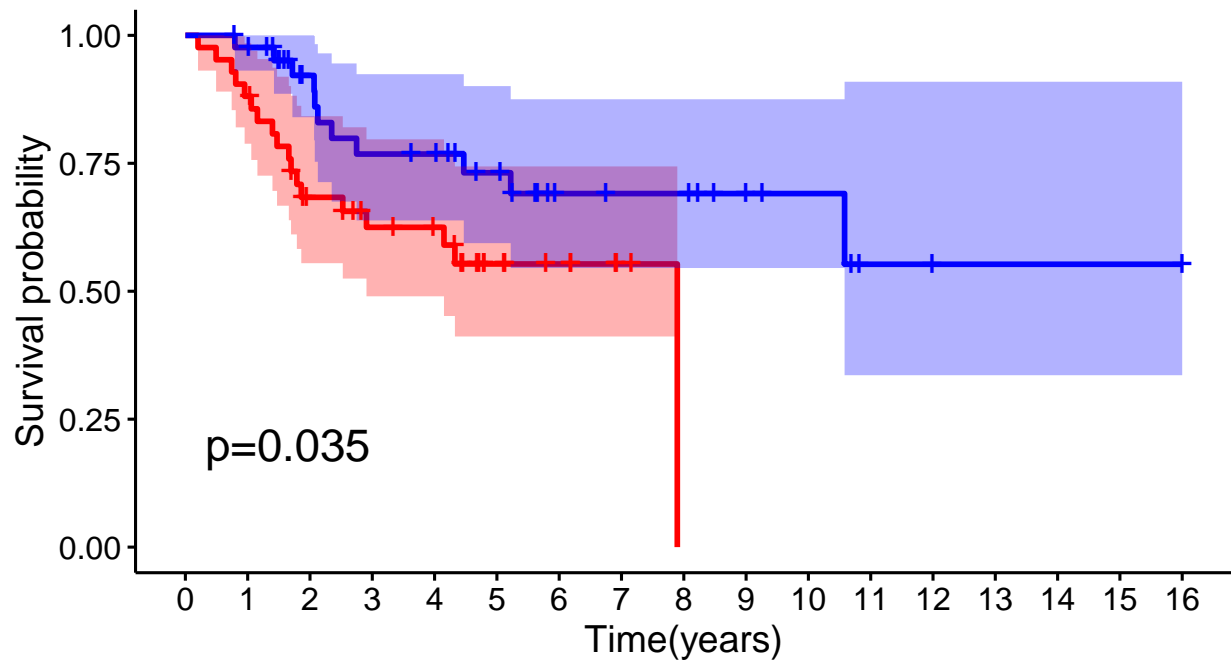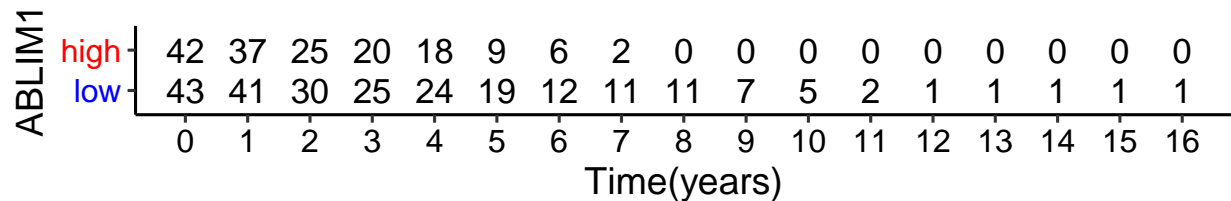

Supplement: Supplementary Document 2 — Kaplan-Meier curve of the 85 malignant genes associated with survival. [file DataSheet_2.zip › Supplementary Document 2/sur.ABLIM1.pdf]

AC092118.1 + high + low

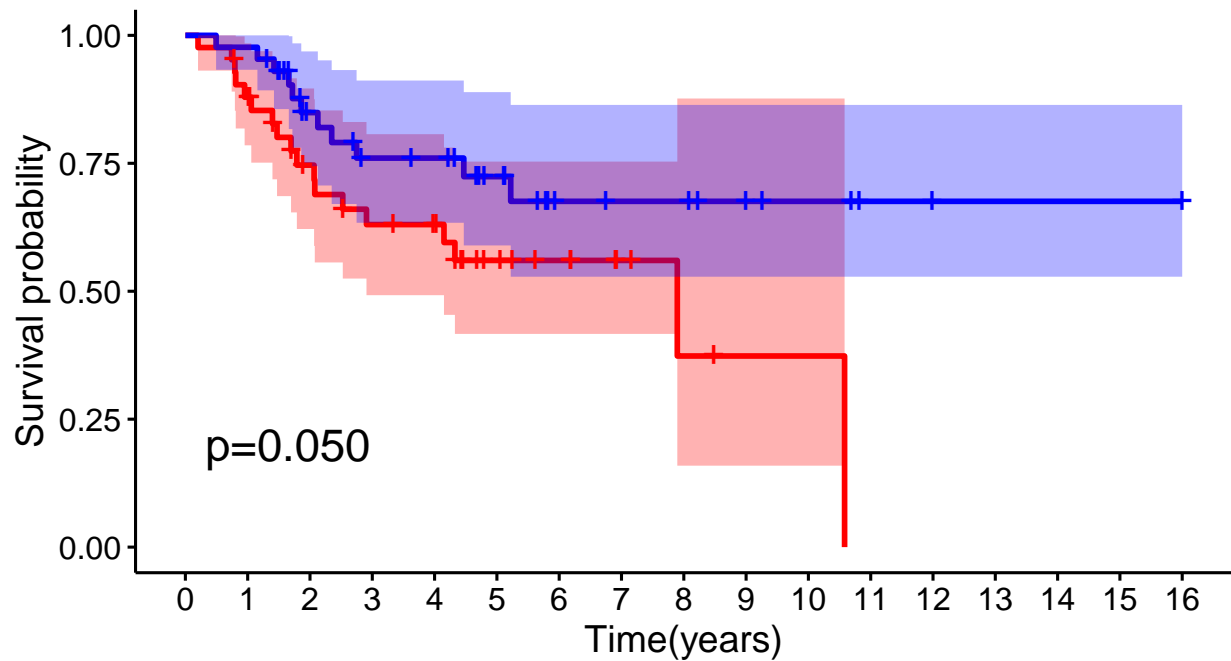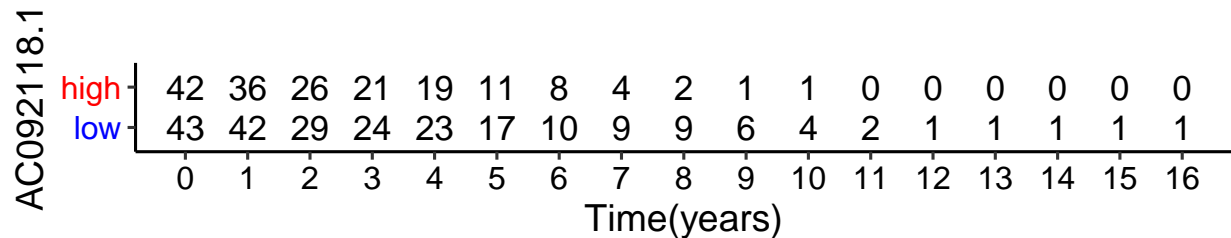

Supplement: Supplementary Document 2 — Kaplan-Meier curve of the 85 malignant genes associated with survival. [file DataSheet_2.zip › Supplementary Document 2/sur.AC092118.1.pdf]

AC093730.1 + high + low

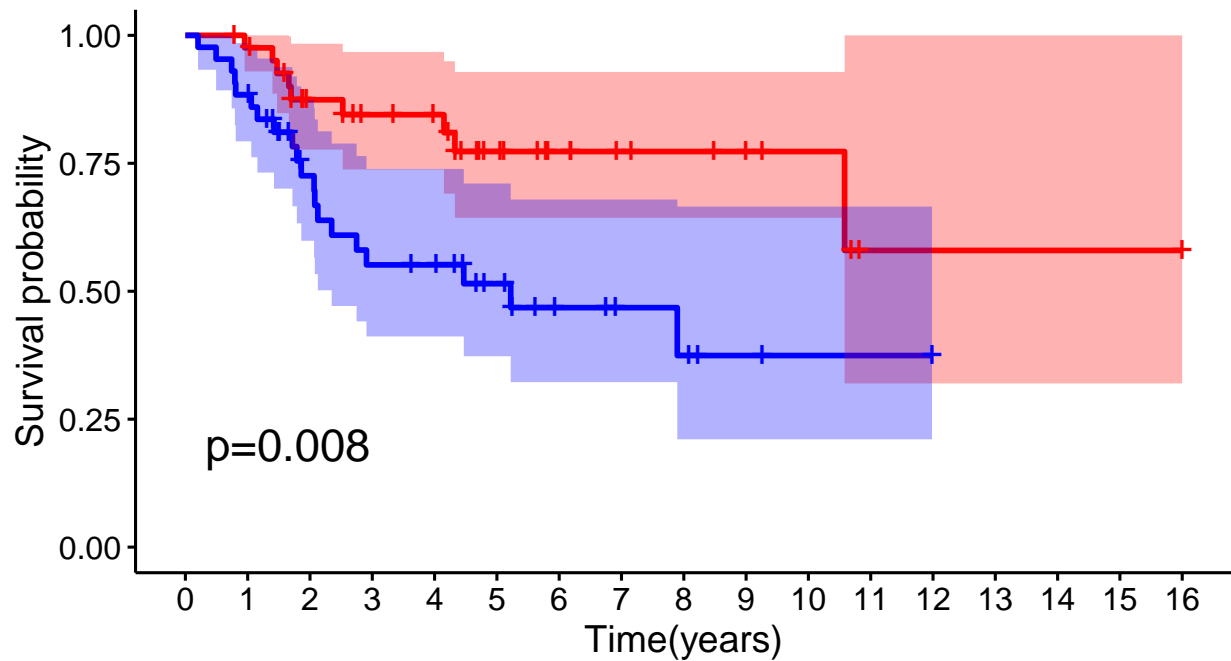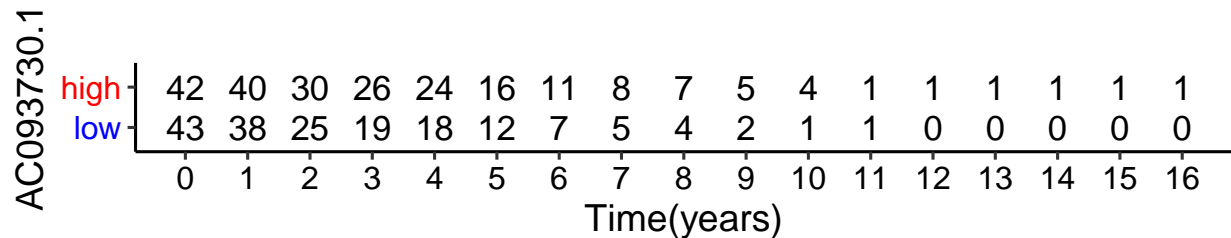

Supplement: Supplementary Document 2 — Kaplan-Meier curve of the 85 malignant genes associated with survival. [file DataSheet_2.zip › Supplementary Document 2/sur.AC093730.1.pdf]

AC096564.1 + high + low

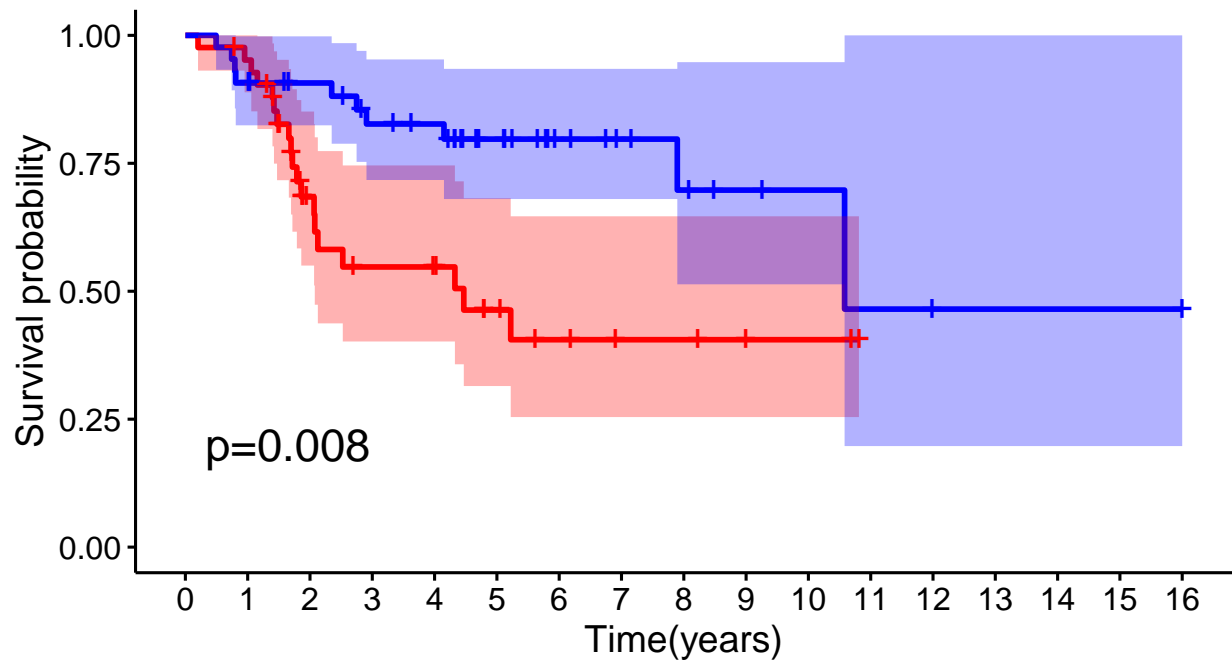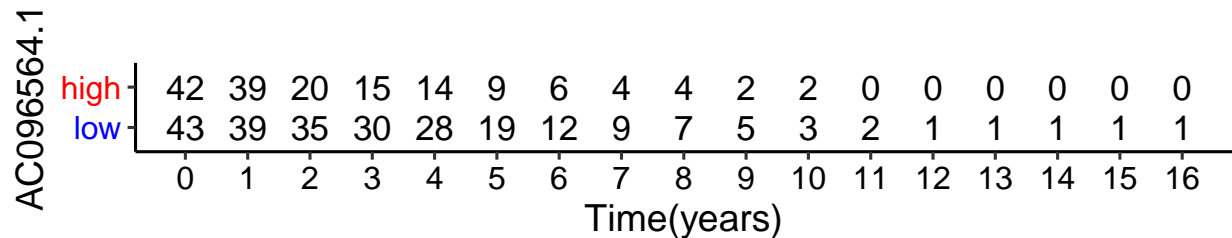

Supplement: Supplementary Document 2 — Kaplan-Meier curve of the 85 malignant genes associated with survival. [file DataSheet_2.zip › Supplementary Document 2/sur.AC096564.1.pdf]

AC099521.1 high low

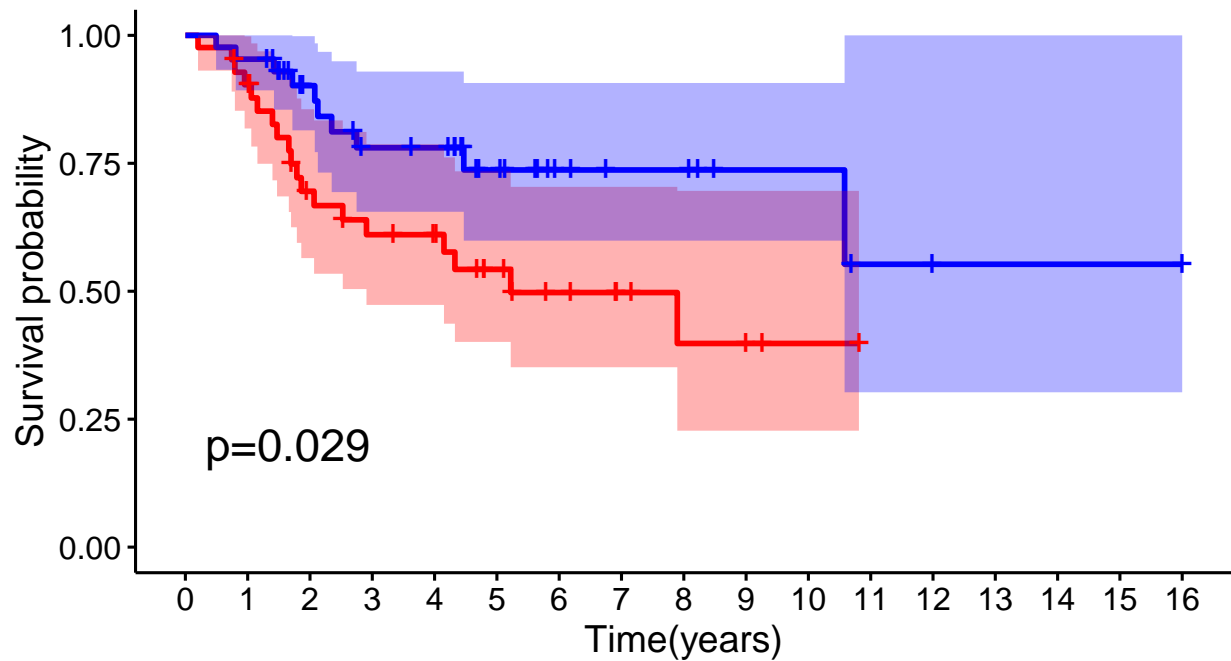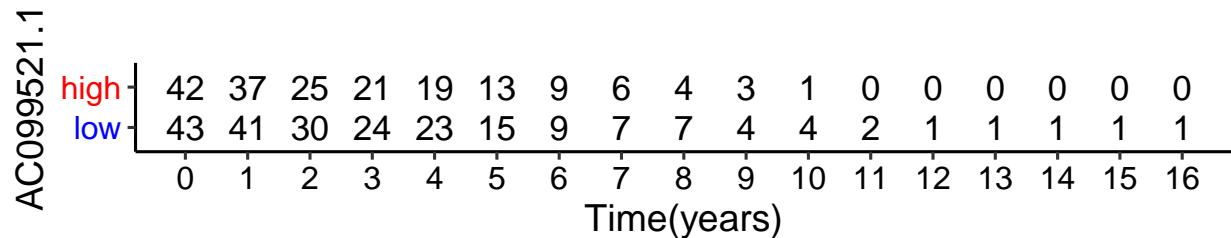

Supplement: Supplementary Document 2 — Kaplan-Meier curve of the 85 malignant genes associated with survival. [file DataSheet_2.zip › Supplementary Document 2/sur.AC099521.1.pdf]

AC116366.2 + high + low

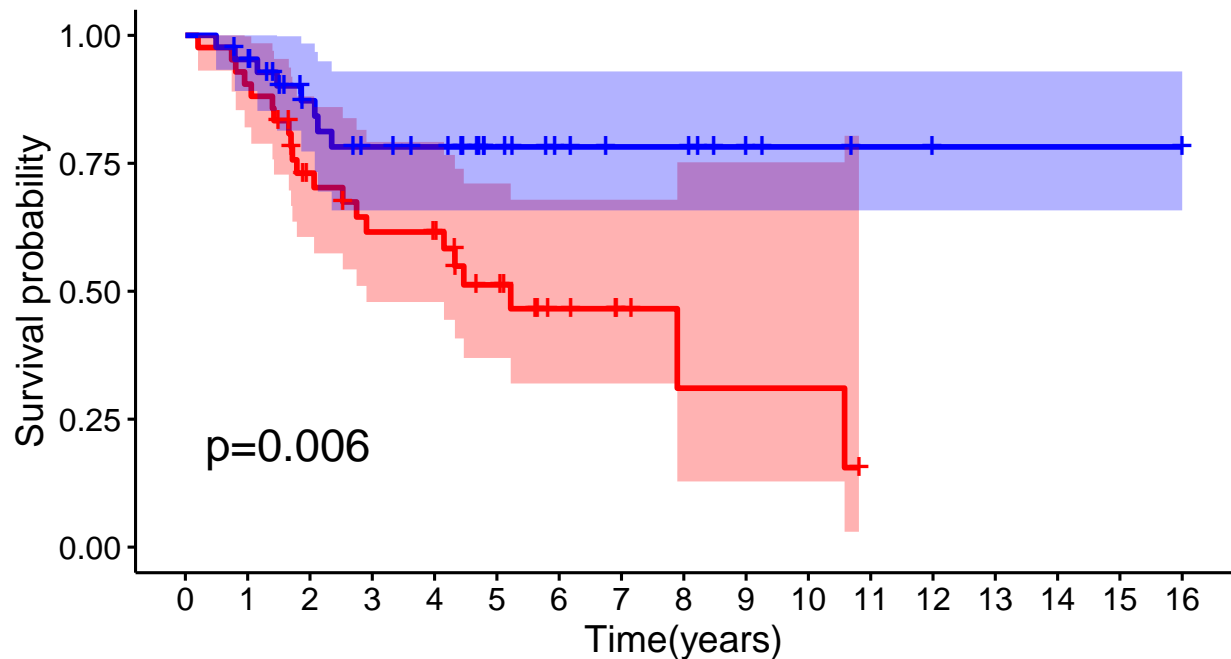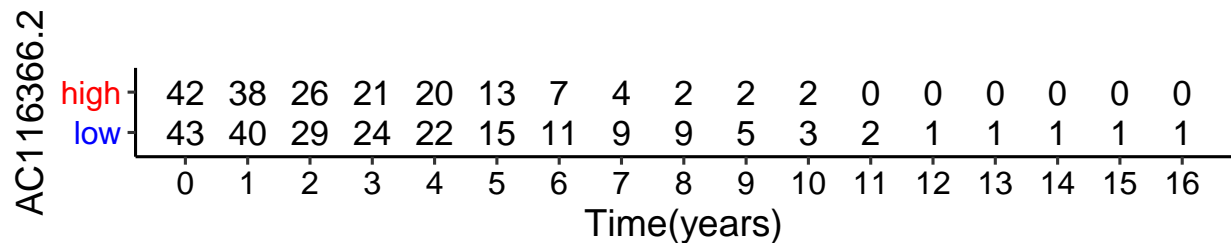

Supplement: Supplementary Document 2 — Kaplan-Meier curve of the 85 malignant genes associated with survival. [file DataSheet_2.zip › Supplementary Document 2/sur.AC116366.2.pdf]

AL161630.1 + high + low

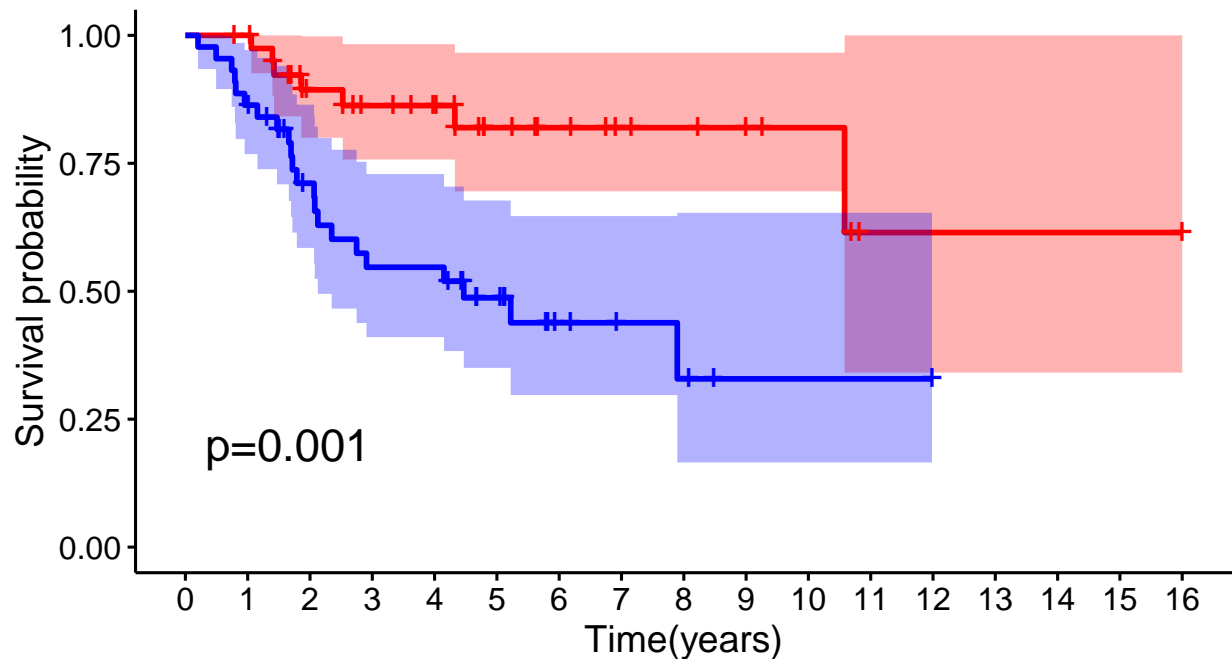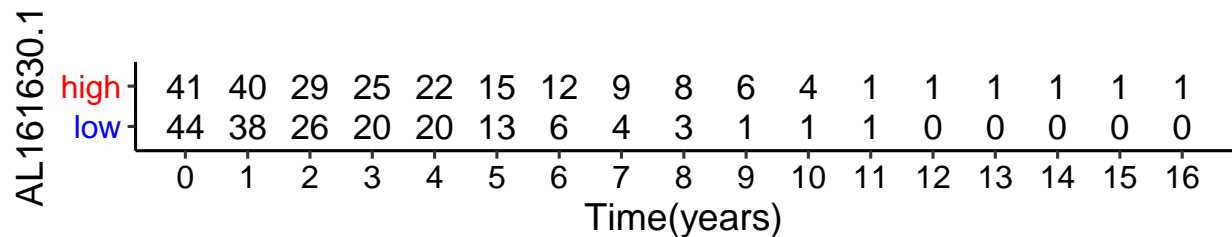

Supplement: Supplementary Document 2 — Kaplan-Meier curve of the 85 malignant genes associated with survival. [file DataSheet_2.zip › Supplementary Document 2/sur.AL161630.1.pdf]

AL583859.2 + high + low

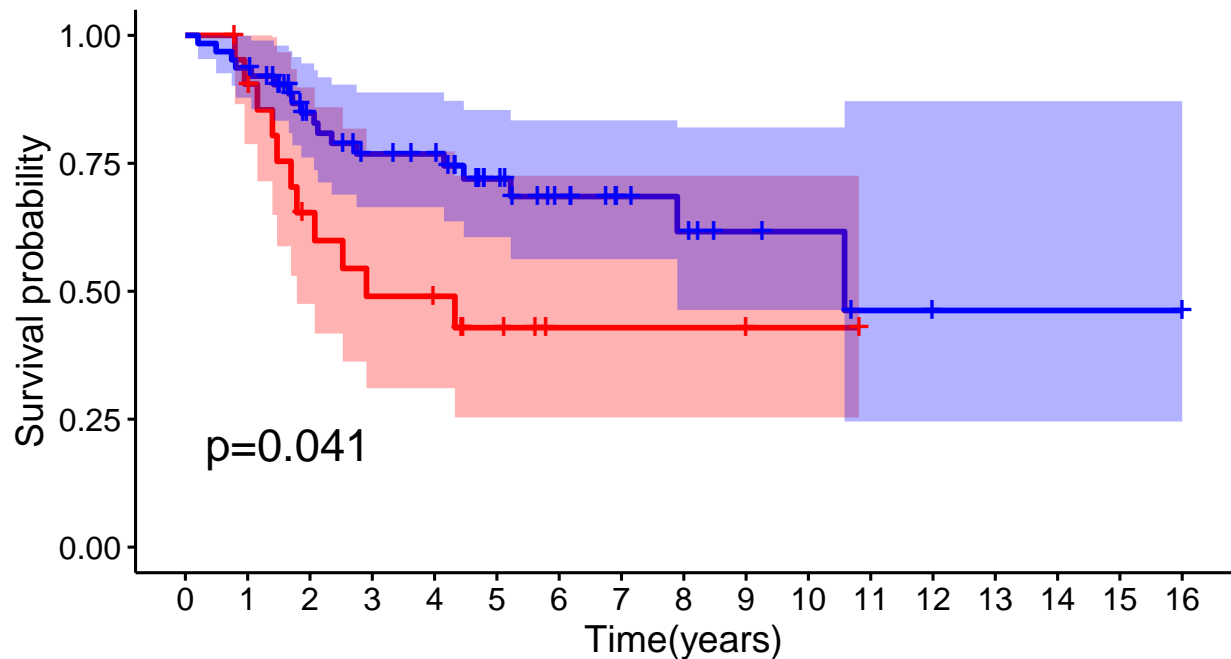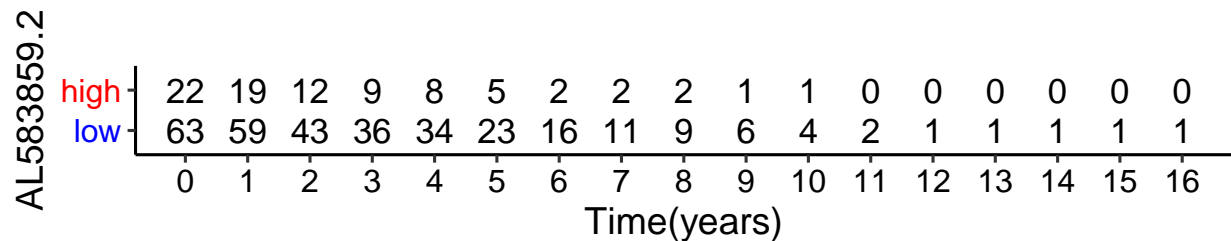

Supplement: Supplementary Document 2 — Kaplan-Meier curve of the 85 malignant genes associated with survival. [file DataSheet_2.zip › Supplementary Document 2/sur.AL583859.2.pdf]

AP000851.2 + high + low

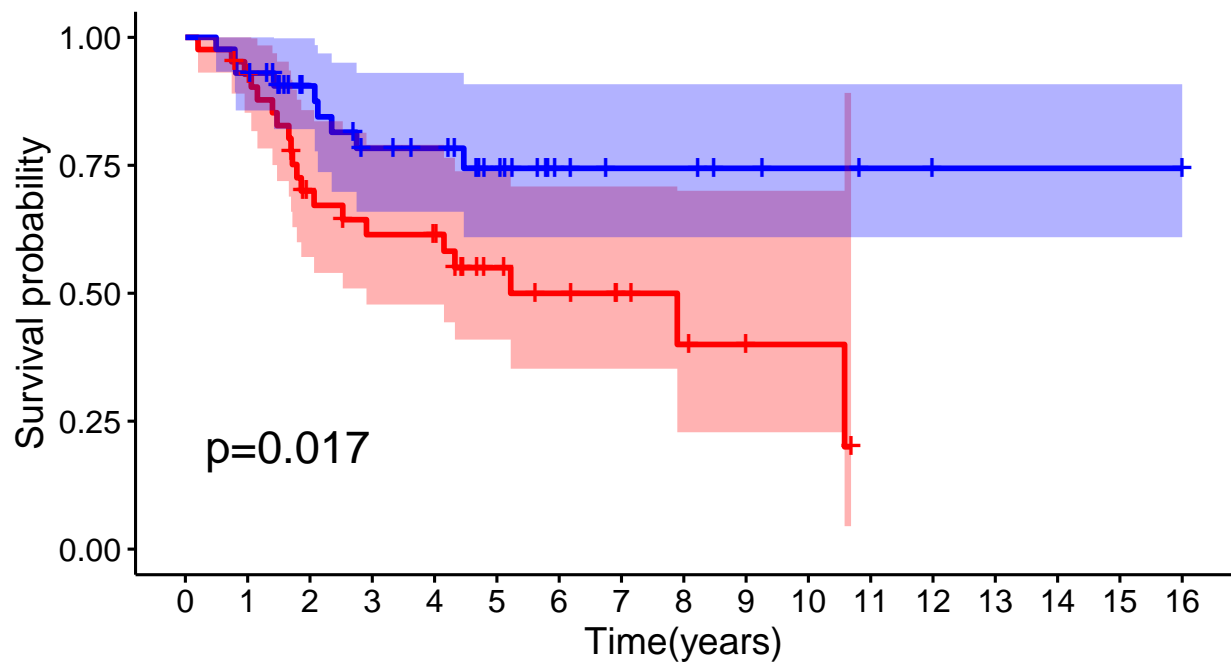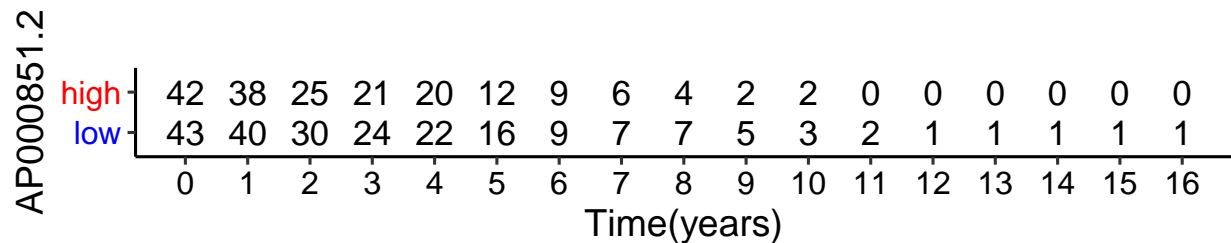

Supplement: Supplementary Document 2 — Kaplan-Meier curve of the 85 malignant genes associated with survival. [file DataSheet_2.zip › Supplementary Document 2/sur.AP000851.2.pdf]

AP003063.1 + high + low

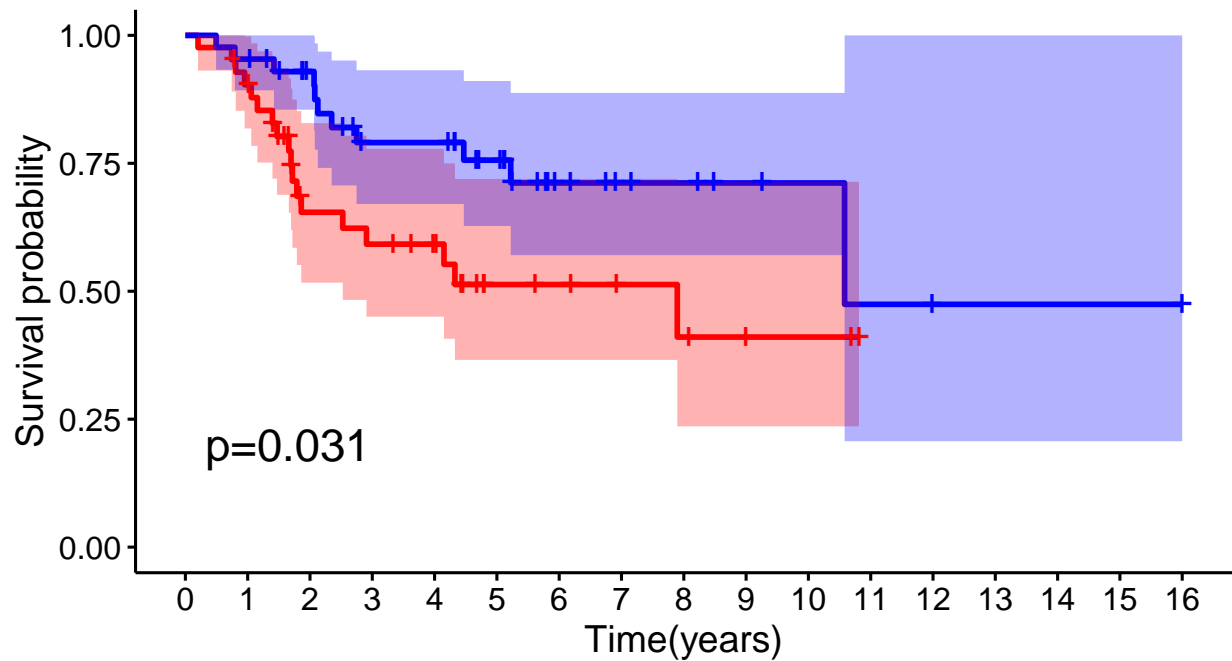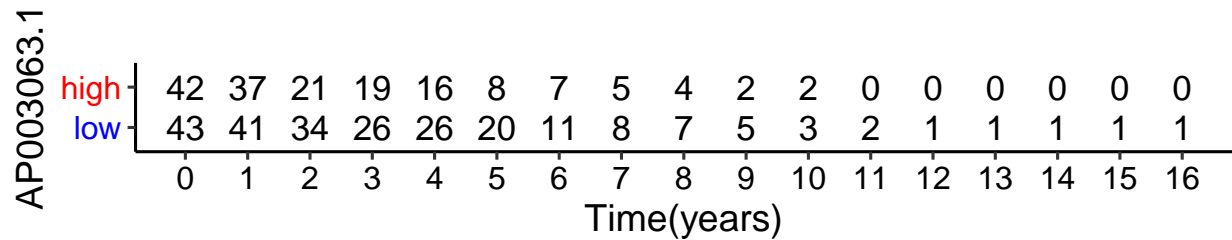

Supplement: Supplementary Document 2 — Kaplan-Meier curve of the 85 malignant genes associated with survival. [file DataSheet_2.zip › Supplementary Document 2/sur.AP003063.1.pdf]

BAIAP2L2 + high + low

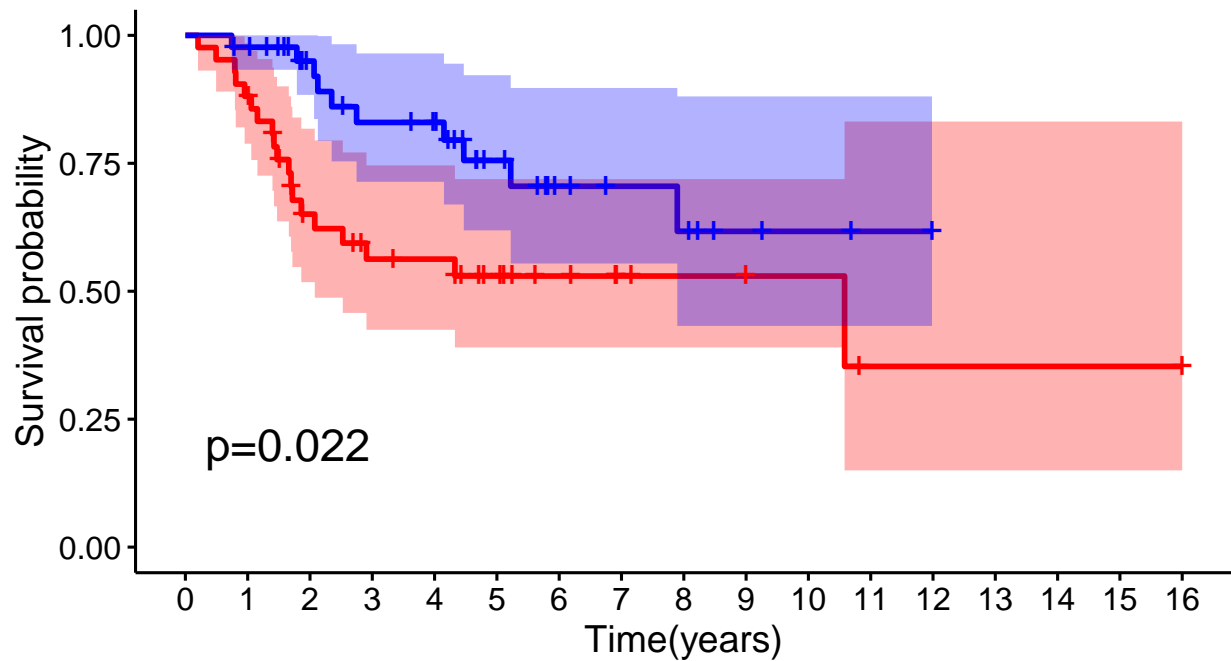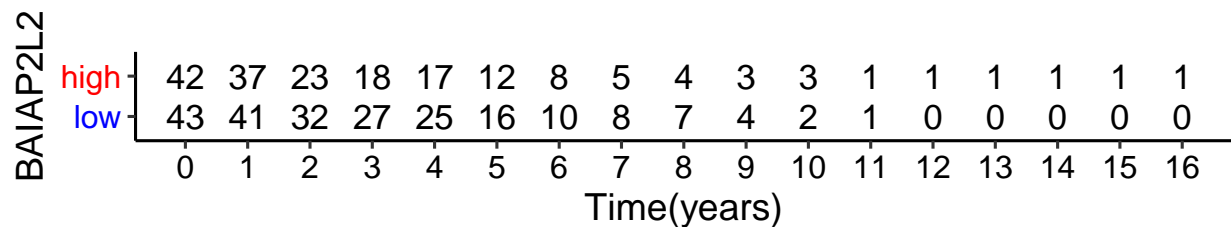

Supplement: Supplementary Document 2 — Kaplan-Meier curve of the 85 malignant genes associated with survival. [file DataSheet_2.zip › Supplementary Document 2/sur.BAIAP2L2.pdf]

BAMBI high low

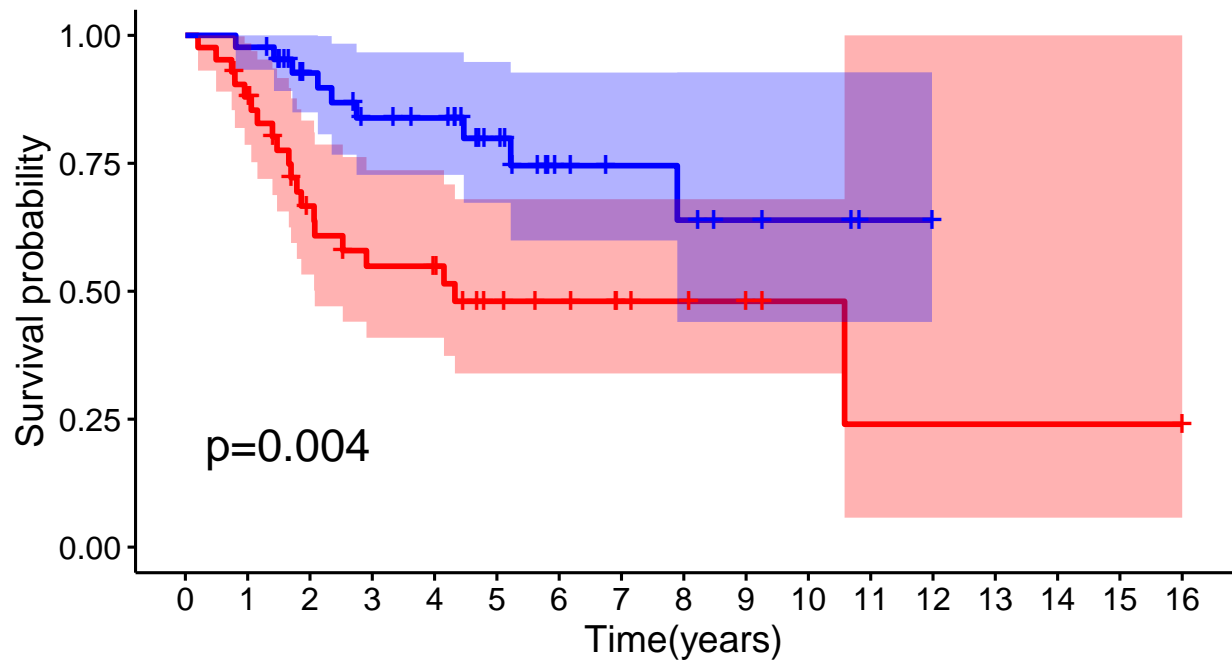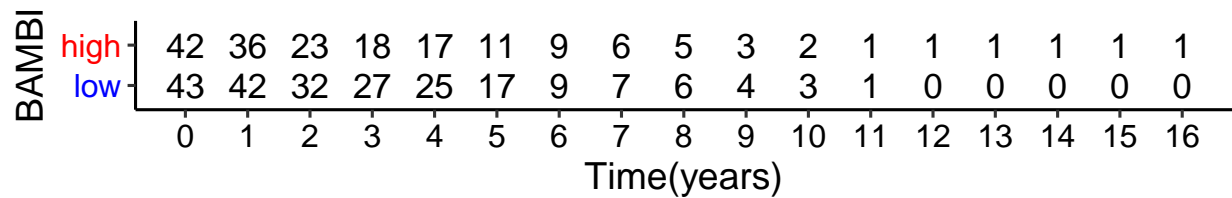

Supplement: Supplementary Document 2 — Kaplan-Meier curve of the 85 malignant genes associated with survival. [file DataSheet_2.zip › Supplementary Document 2/sur.BAMBI.pdf]

BMP8B high low

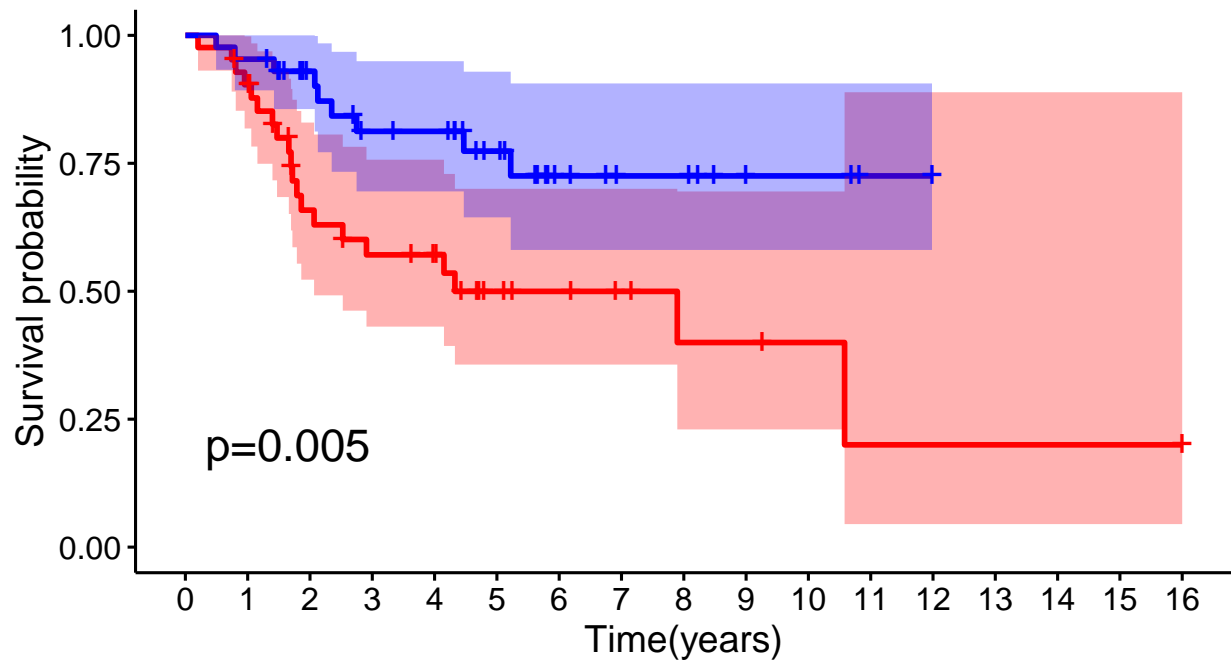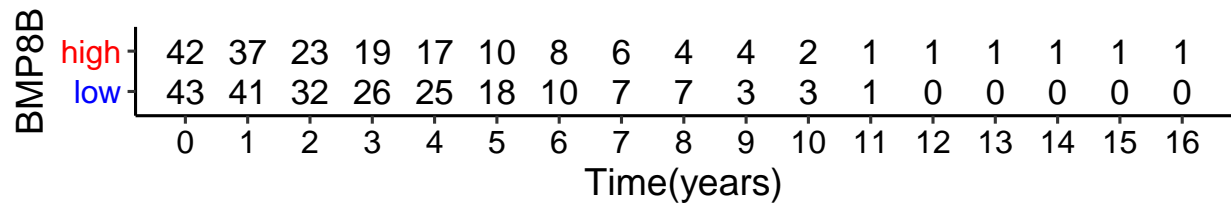

Supplement: Supplementary Document 2 — Kaplan-Meier curve of the 85 malignant genes associated with survival. [file DataSheet_2.zip › Supplementary Document 2/sur.BMP8B.pdf]

BOK + high + low

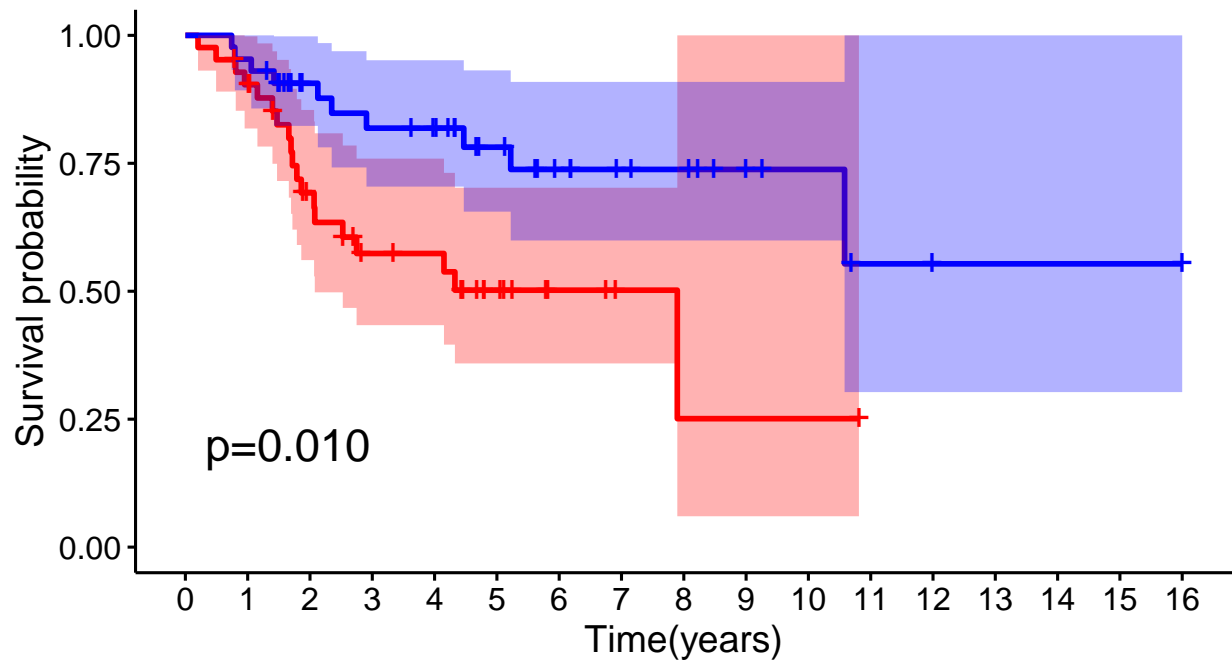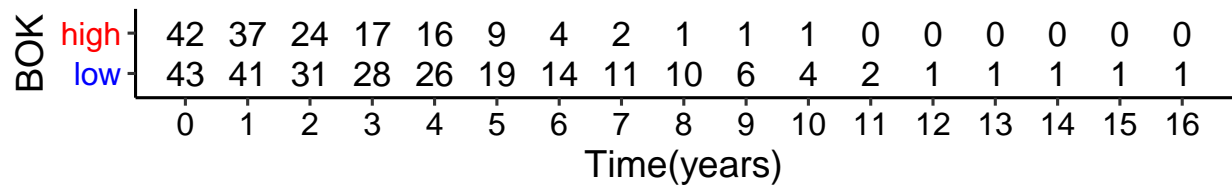

Supplement: Supplementary Document 2 — Kaplan-Meier curve of the 85 malignant genes associated with survival. [file DataSheet_2.zip › Supplementary Document 2/sur.BOK.pdf]

CACNB4 high low

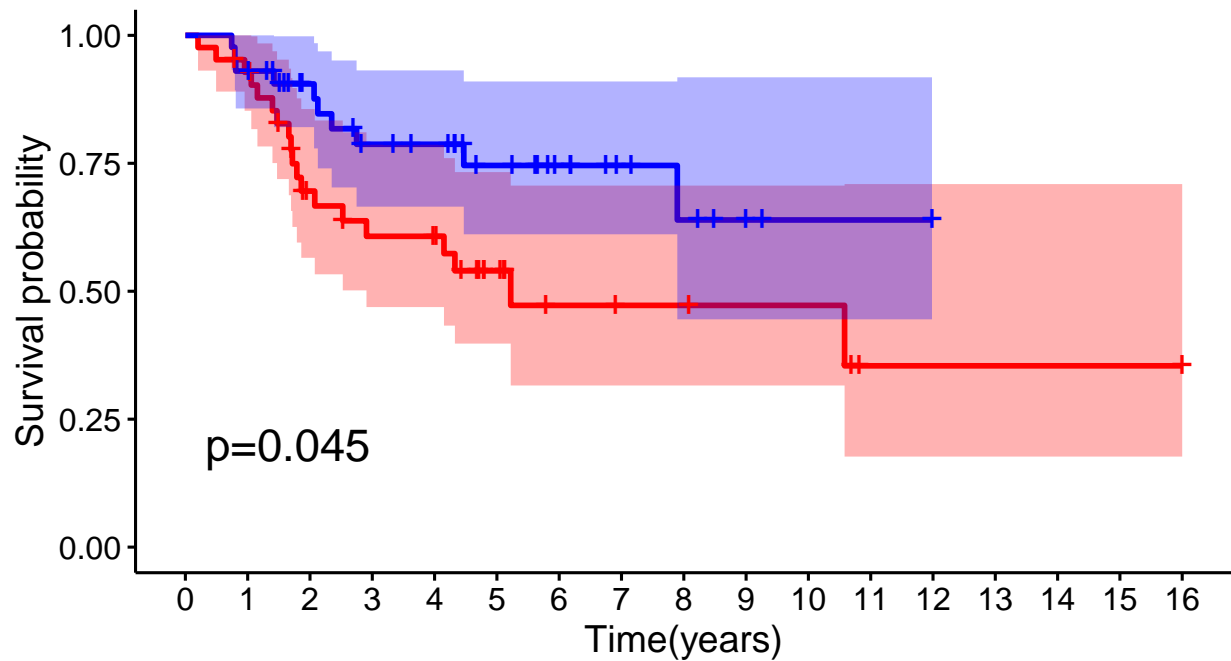

CACNB4

high

low

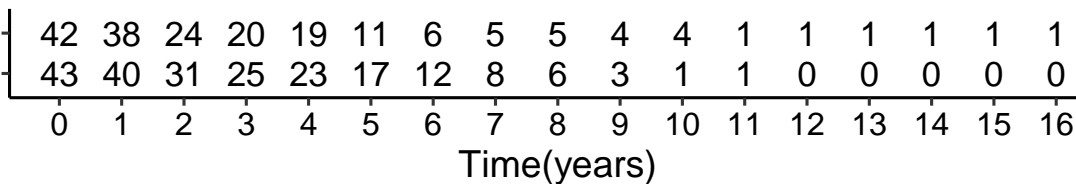

Supplement: Supplementary Document 2 — Kaplan-Meier curve of the 85 malignant genes associated with survival. [file DataSheet_2.zip › Supplementary Document 2/sur.CACNB4.pdf]

CCDC194 + high + low

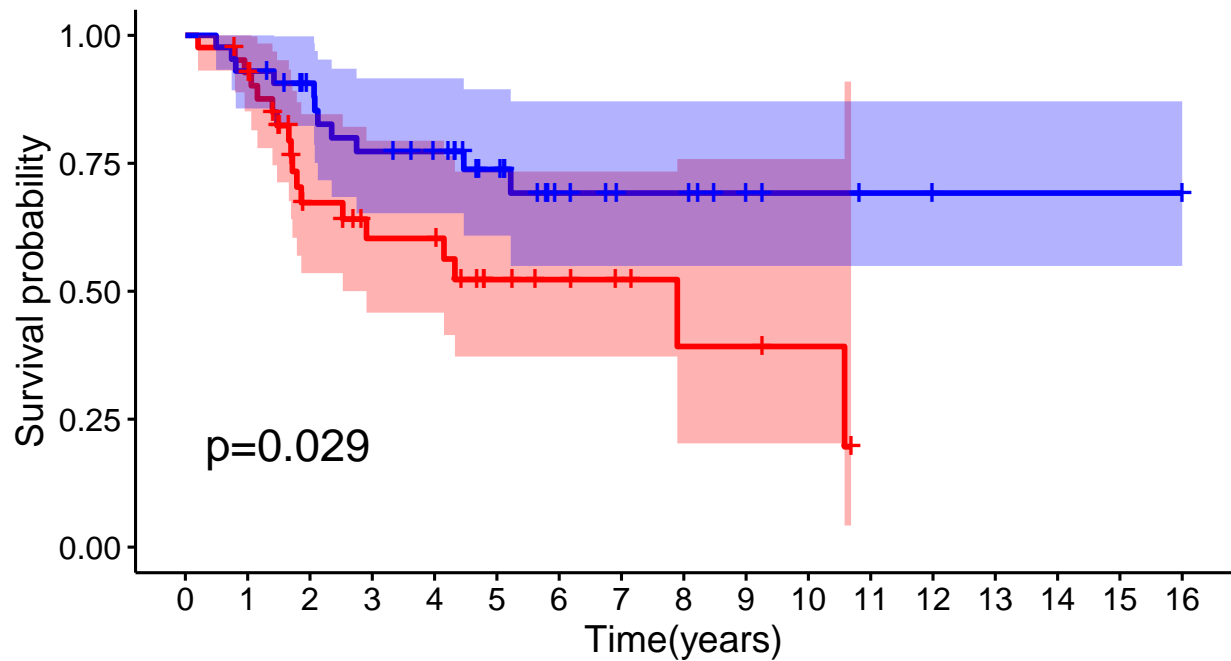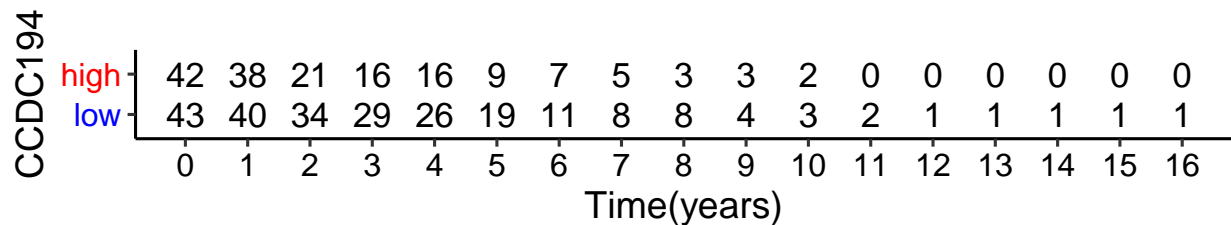

Supplement: Supplementary Document 2 — Kaplan-Meier curve of the 85 malignant genes associated with survival. [file DataSheet_2.zip › Supplementary Document 2/sur.CCDC194.pdf]

CCDC3 + high + low

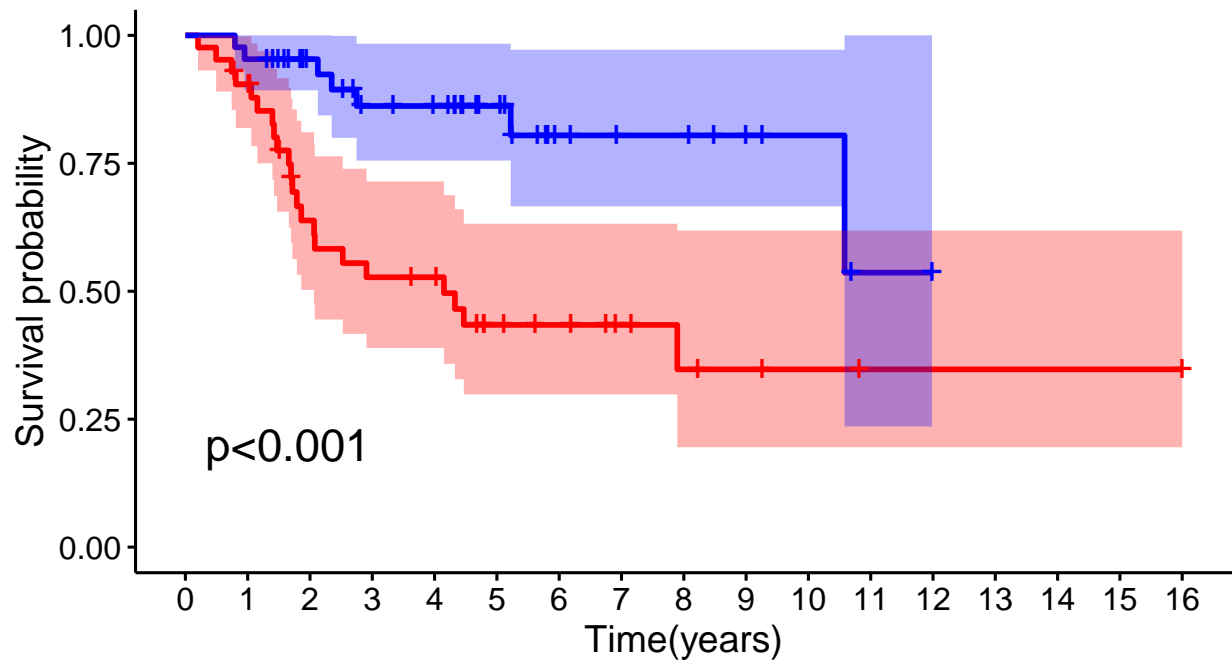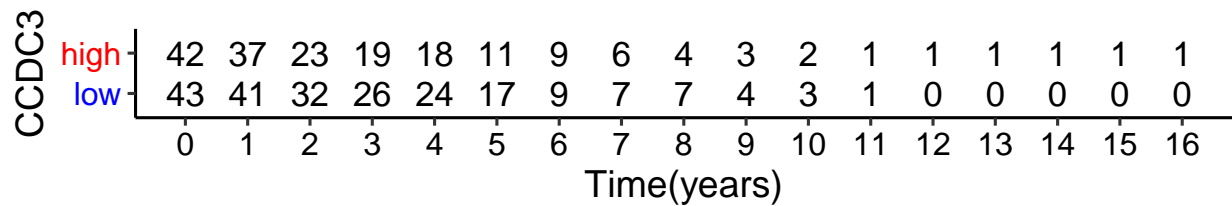

Supplement: Supplementary Document 2 — Kaplan-Meier curve of the 85 malignant genes associated with survival. [file DataSheet_2.zip › Supplementary Document 2/sur.CCDC3.pdf]

CDC42EP3 + high + low

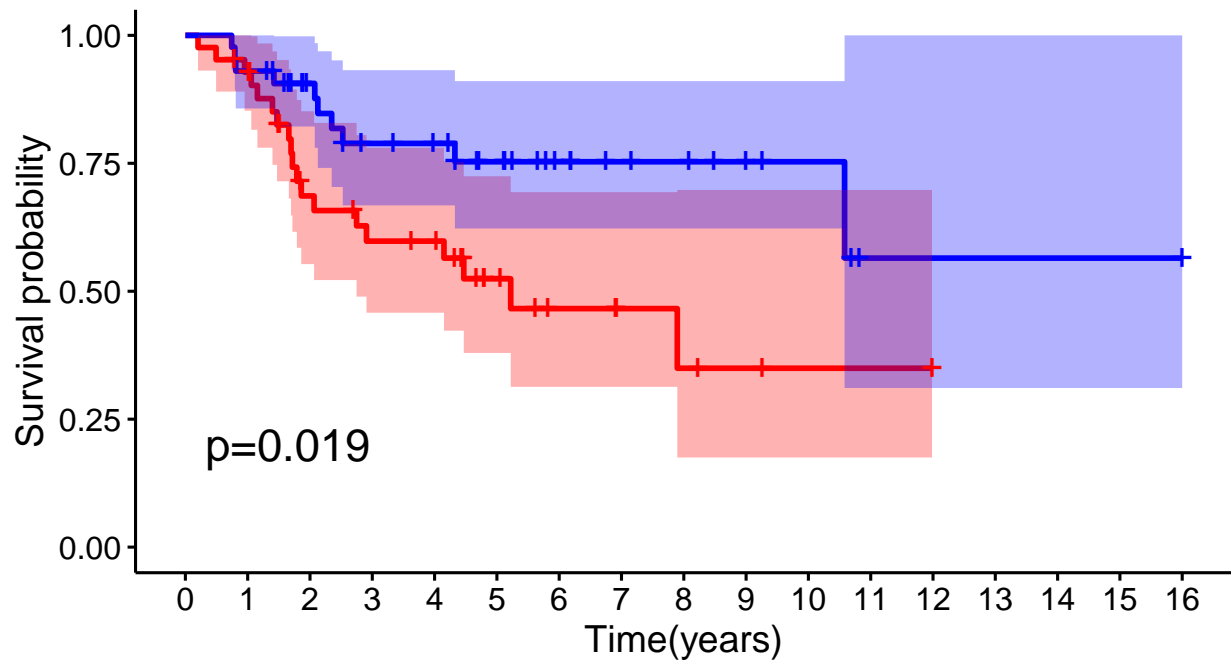

CDC42EP3

high  
low

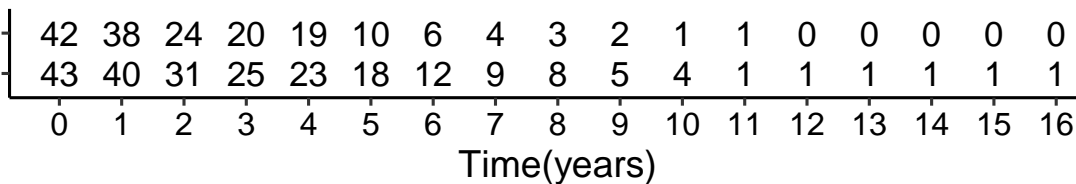

Supplement: Supplementary Document 2 — Kaplan-Meier curve of the 85 malignant genes associated with survival. [file DataSheet_2.zip › Supplementary Document 2/sur.CDC42EP3.pdf]

CFAP44 + high + low

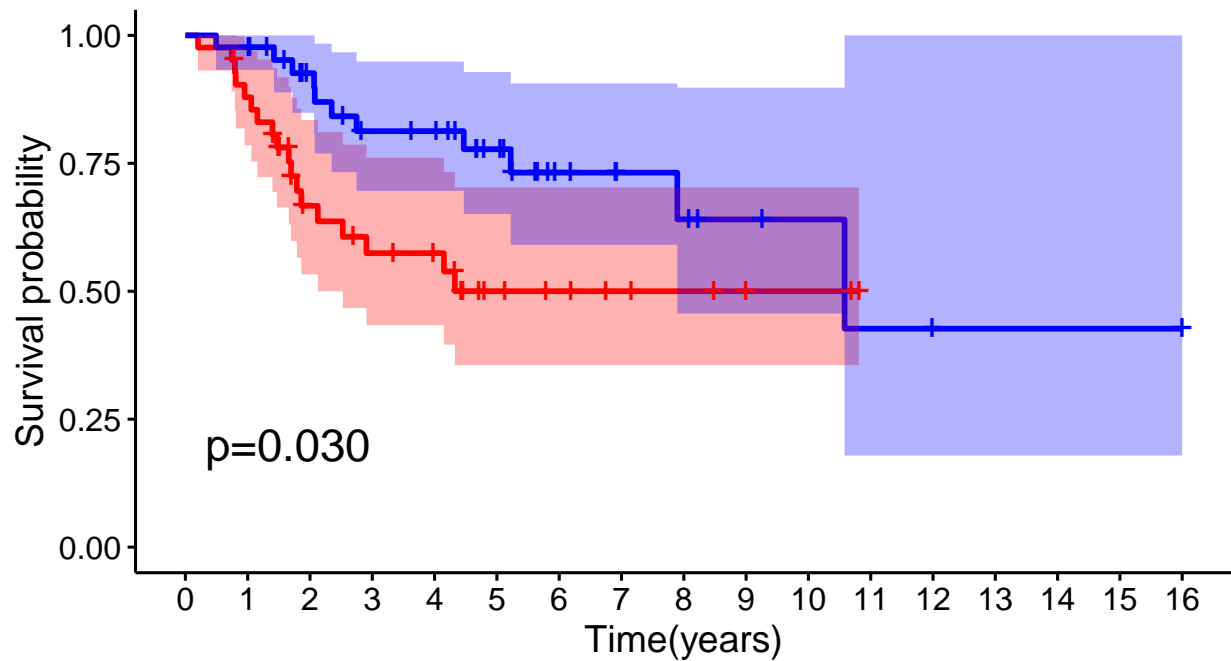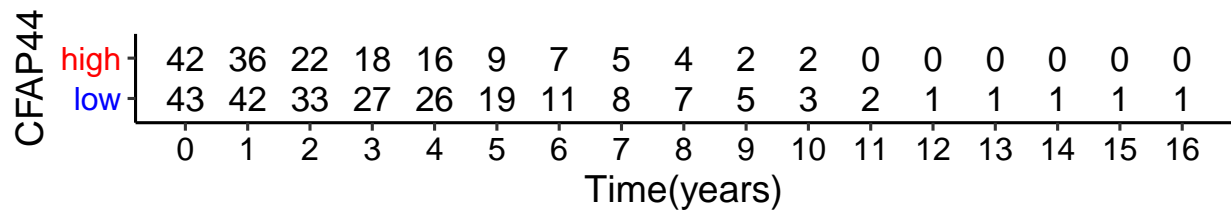

Supplement: Supplementary Document 2 — Kaplan-Meier curve of the 85 malignant genes associated with survival. [file DataSheet_2.zip › Supplementary Document 2/sur.CFAP44.pdf]

CGREF1 high low

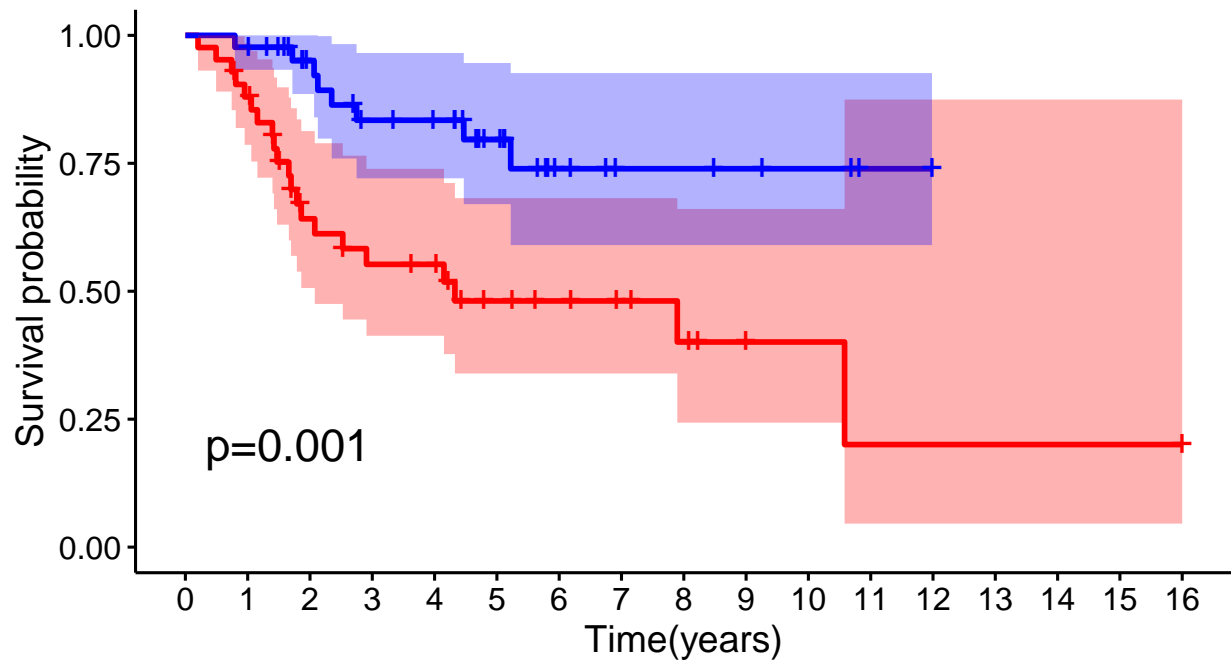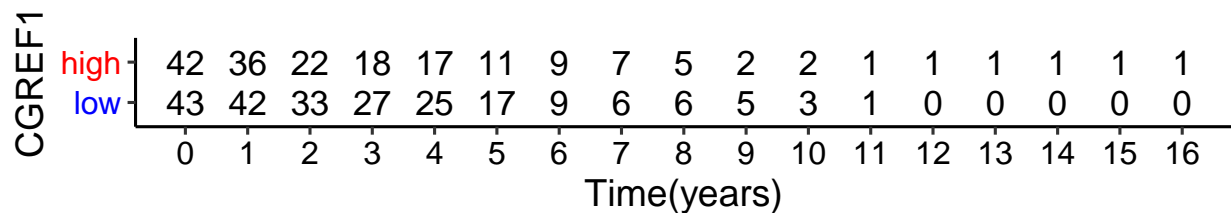

Supplement: Supplementary Document 2 — Kaplan-Meier curve of the 85 malignant genes associated with survival. [file DataSheet_2.zip › Supplementary Document 2/sur.CGREF1.pdf]

CHST13 high low

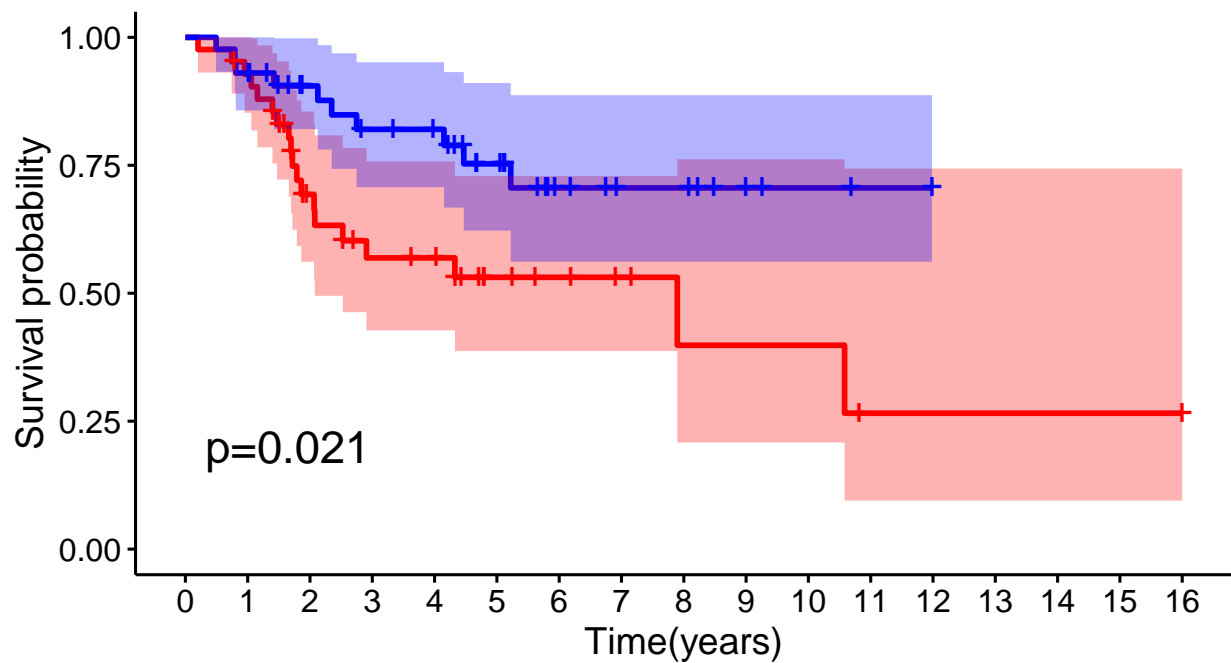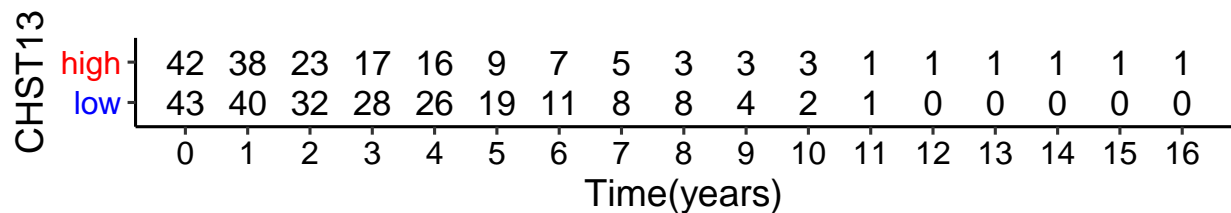

Supplement: Supplementary Document 2 — Kaplan-Meier curve of the 85 malignant genes associated with survival. [file DataSheet_2.zip › Supplementary Document 2/sur.CHST13.pdf]

CHST8 + high + low

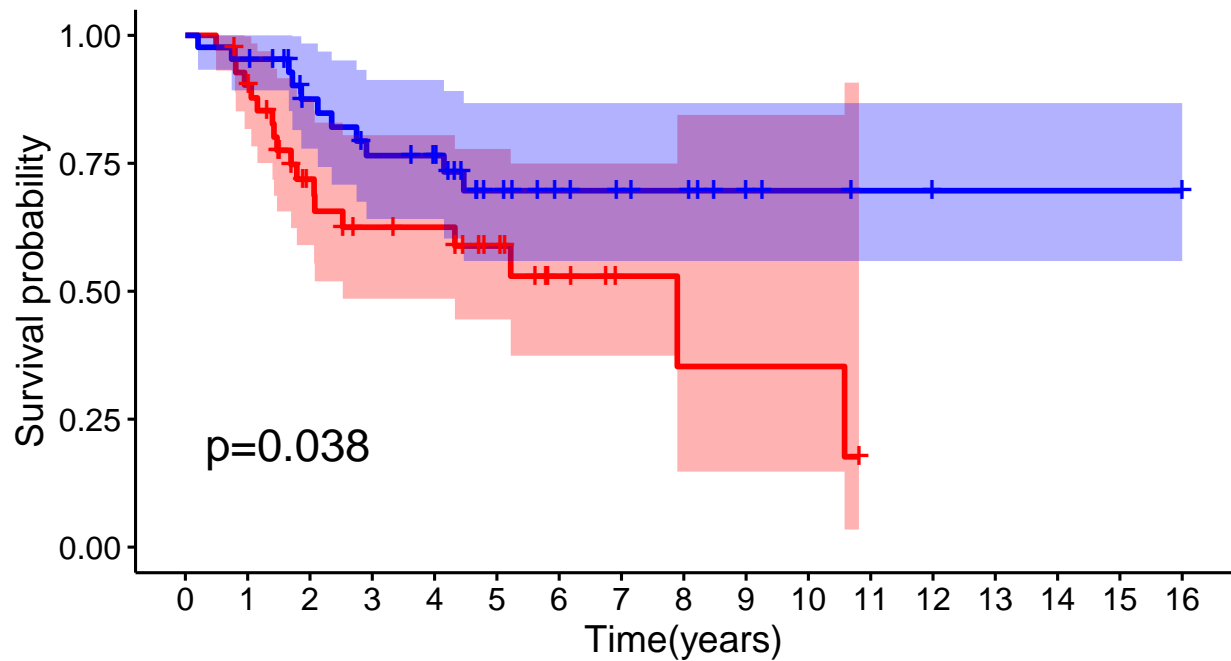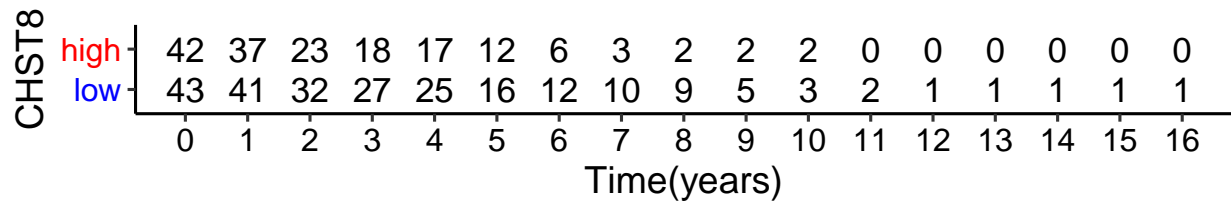

Supplement: Supplementary Document 2 — Kaplan-Meier curve of the 85 malignant genes associated with survival. [file DataSheet_2.zip › Supplementary Document 2/sur.CHST8.pdf]

CITED4 + high + low

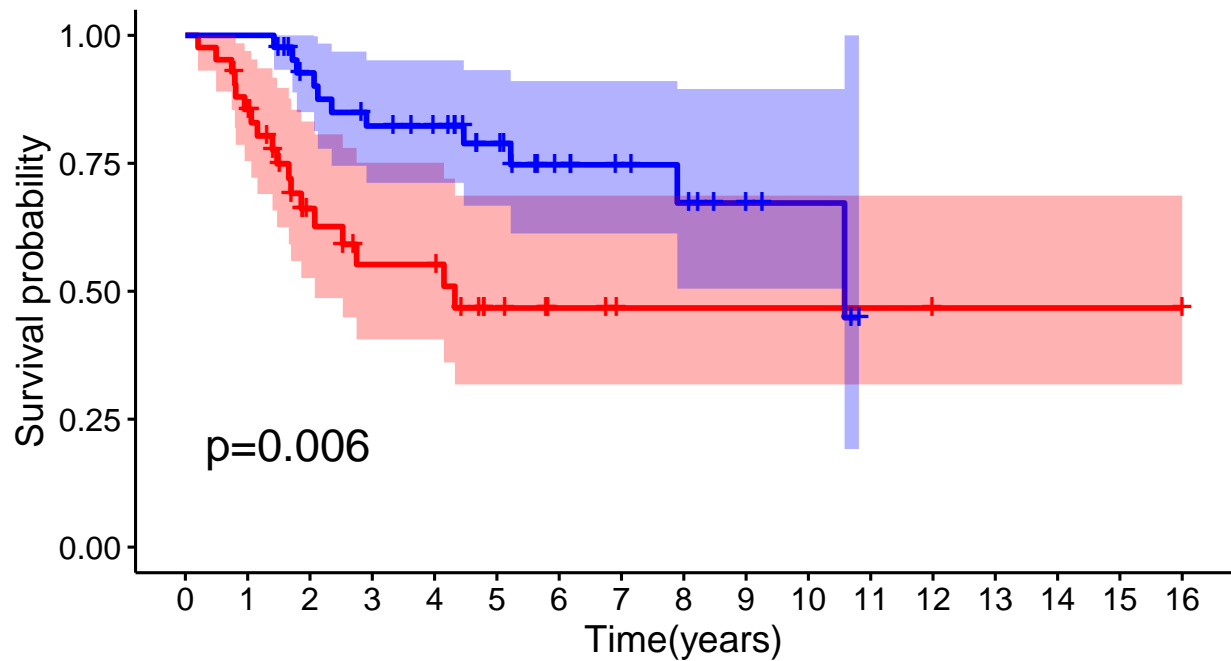

CITED4

|      |             |    |    |    |    |    |    |    |   |   |    |    |    |    |    |    |    |
|------|-------------|----|----|----|----|----|----|----|---|---|----|----|----|----|----|----|----|
| high | 42          | 35 | 19 | 14 | 14 | 7  | 4  | 2  | 2 | 2 | 2  | 2  | 1  | 1  | 1  | 1  | 1  |
| low  | 43          | 43 | 36 | 31 | 28 | 21 | 14 | 11 | 9 | 5 | 3  | 0  | 0  | 0  | 0  | 0  | 0  |
|      | 0           | 1  | 2  | 3  | 4  | 5  | 6  | 7  | 8 | 9 | 10 | 11 | 12 | 13 | 14 | 15 | 16 |
|      | Time(years) |    |    |    |    |    |    |    |   |   |    |    |    |    |    |    |    |

Supplement: Supplementary Document 2 — Kaplan-Meier curve of the 85 malignant genes associated with survival. [file DataSheet_2.zip › Supplementary Document 2/sur.CITED4.pdf]
