# Supplementary figures and images for: Single-Cell Transcriptomics Reveals the Complexity of the Tumor Microenvironment of Treatment-Naive Osteosarcoma (part 3 of 3)
Source: Front Oncol. 2021 Jul 21;11:709210. doi: 10.3389/fonc.2021.709210 (PMC8335545; doi:10.3389/fonc.2021.709210)

CLDN11 + high + low

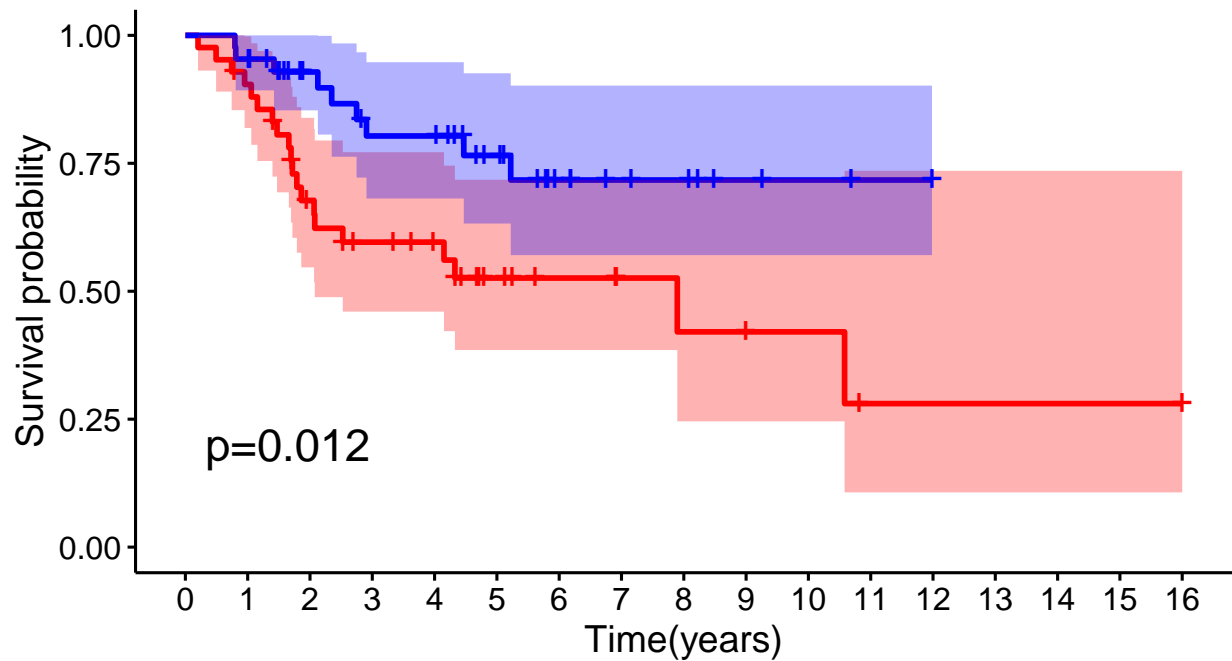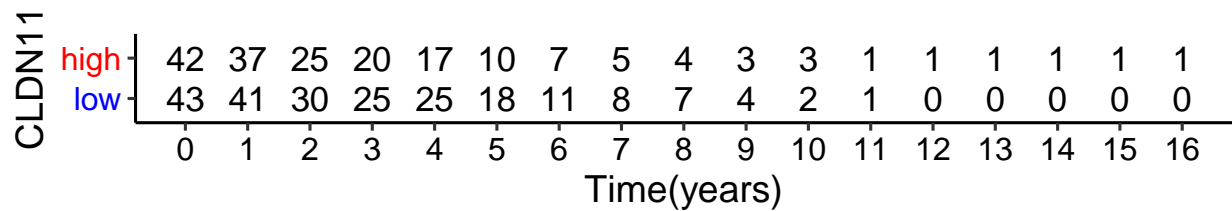

Supplement: Supplementary Document 2 — Kaplan-Meier curve of the 85 malignant genes associated with survival. [file DataSheet_2.zip › Supplementary Document 2/sur.CLDN11.pdf]

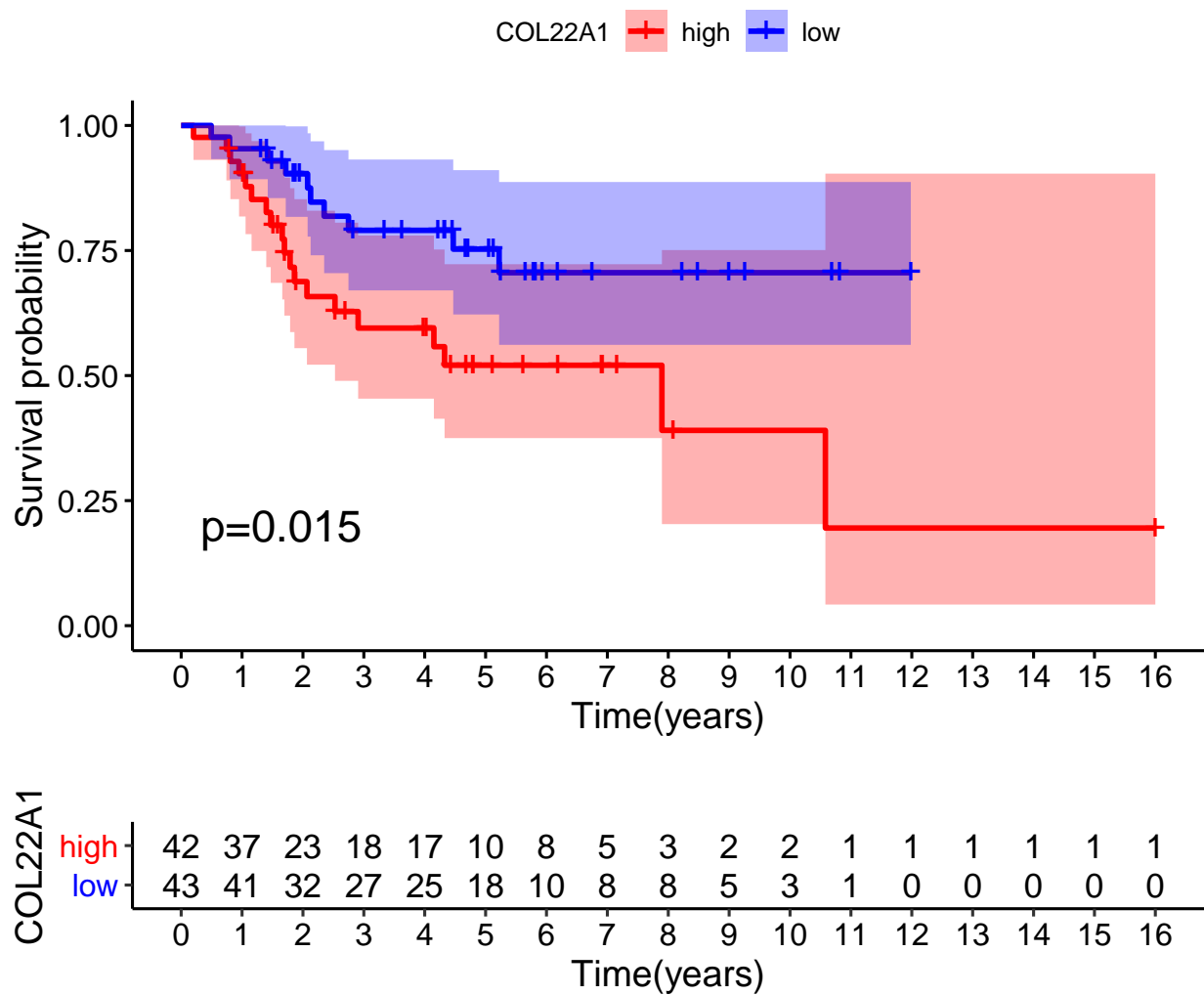

Supplement: Supplementary Document 2 — Kaplan-Meier curve of the 85 malignant genes associated with survival. [file DataSheet_2.zip › Supplementary Document 2/sur.COL22A1.pdf]

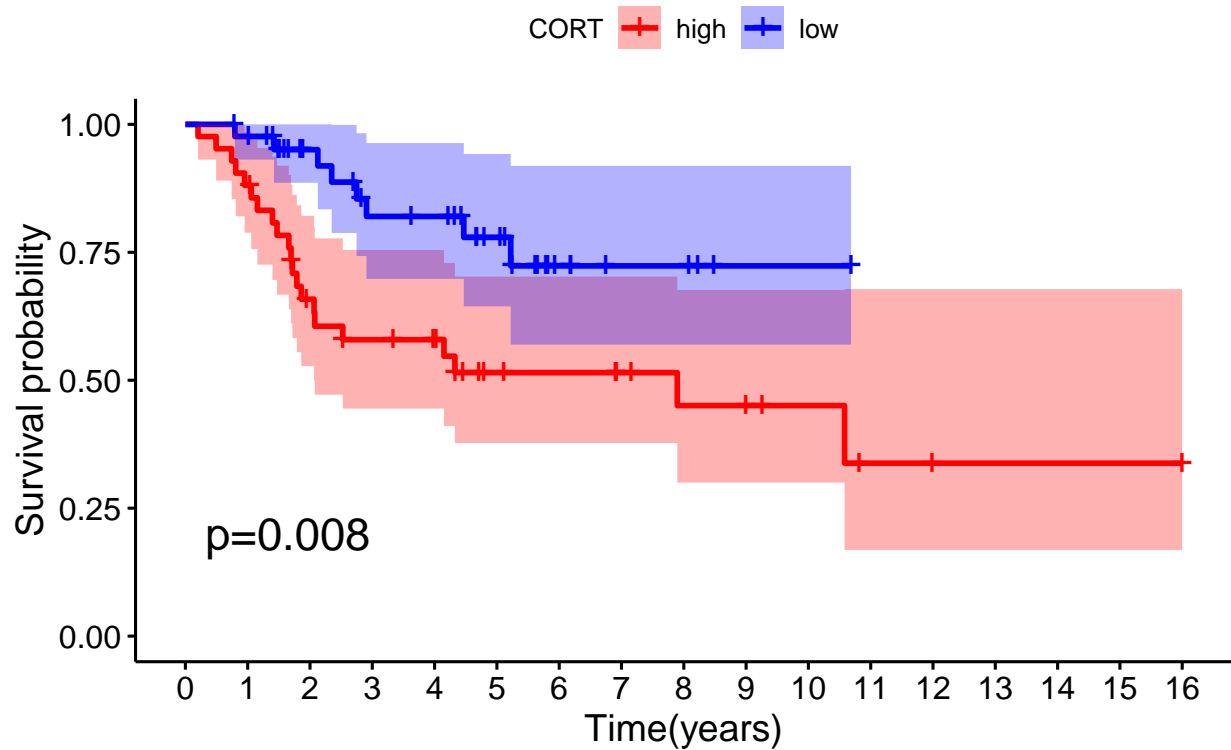

| CORT | Time(years) |    |    |    |    |    |    |   |   |   |    |    |    |    |    |    |    |
|------|-------------|----|----|----|----|----|----|---|---|---|----|----|----|----|----|----|----|
|      | 0           | 1  | 2  | 3  | 4  | 5  | 6  | 7 | 8 | 9 | 10 | 11 | 12 | 13 | 14 | 15 | 16 |
| high | 42          | 37 | 25 | 21 | 19 | 12 | 11 | 9 | 7 | 6 | 4  | 2  | 1  | 1  | 1  | 1  | 1  |
| low  | 43          | 41 | 30 | 24 | 23 | 16 | 7  | 4 | 4 | 1 | 1  | 0  | 0  | 0  | 0  | 0  | 0  |

Supplement: Supplementary Document 2 — Kaplan-Meier curve of the 85 malignant genes associated with survival. [file DataSheet_2.zip › Supplementary Document 2/sur.CORT.pdf]

CPE + high + low

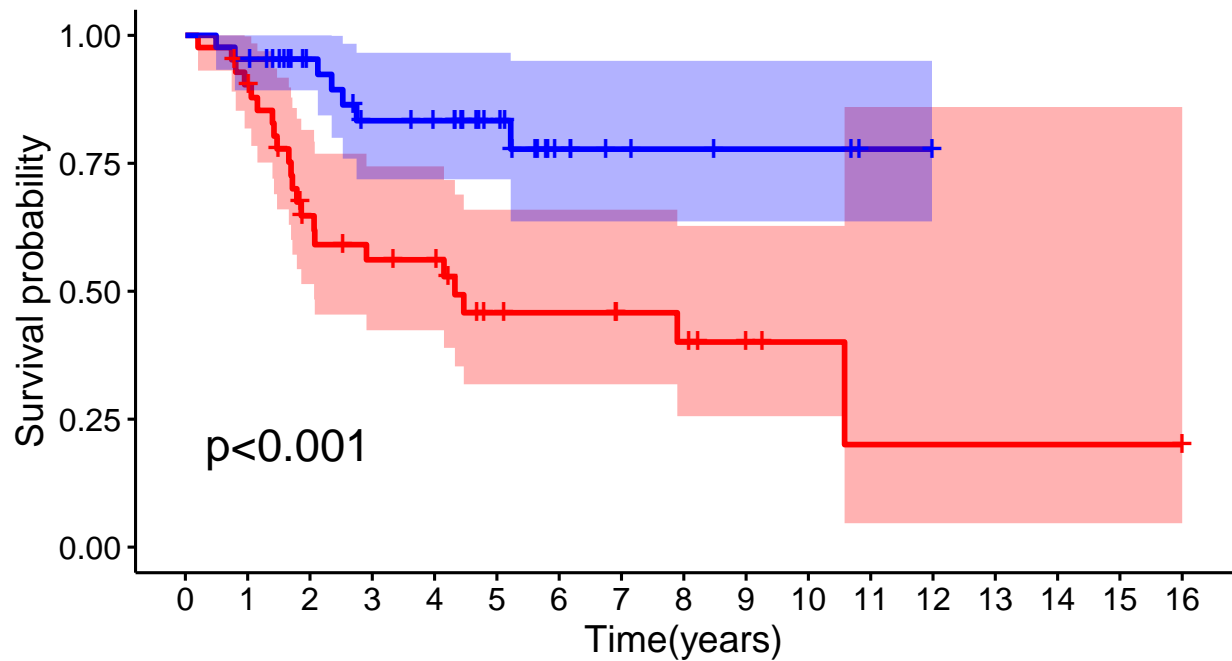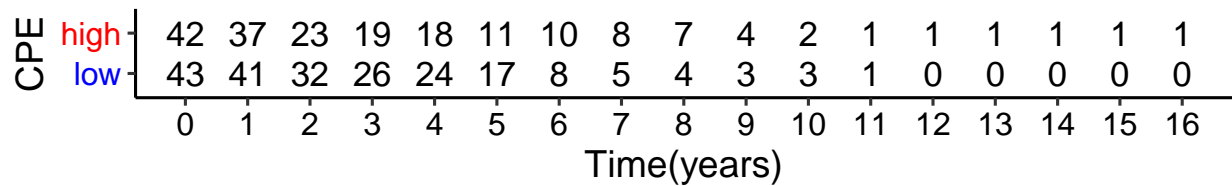

Supplement: Supplementary Document 2 — Kaplan-Meier curve of the 85 malignant genes associated with survival. [file DataSheet_2.zip › Supplementary Document 2/sur.CPE.pdf]

CXADR + high + low

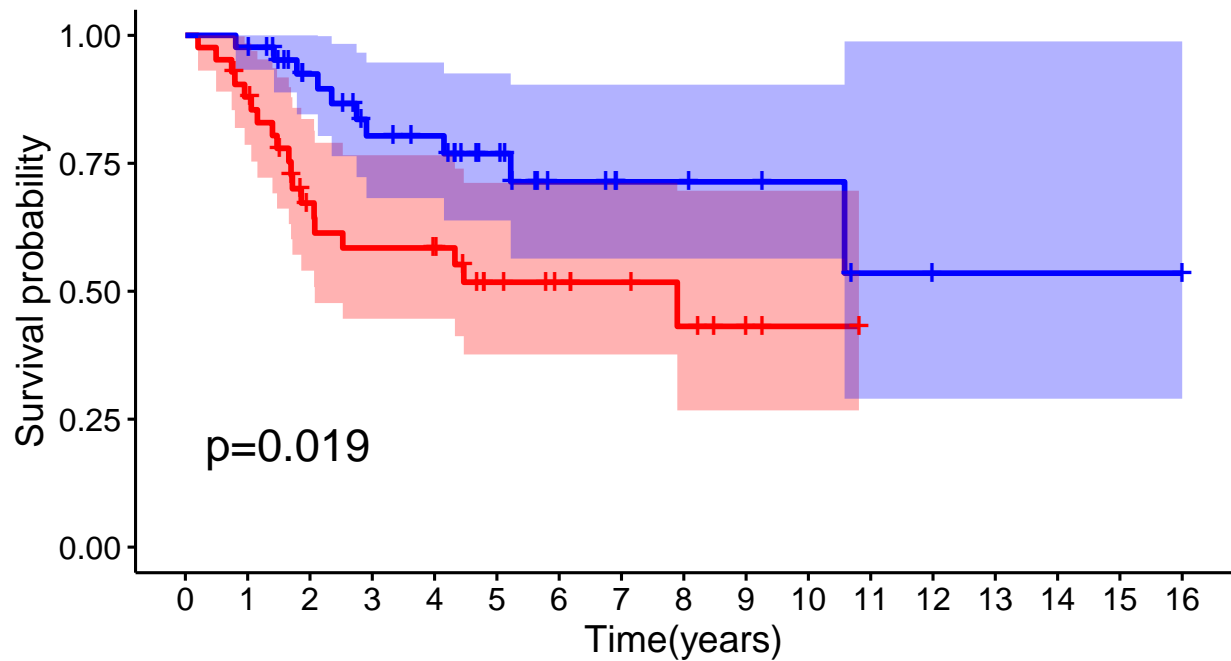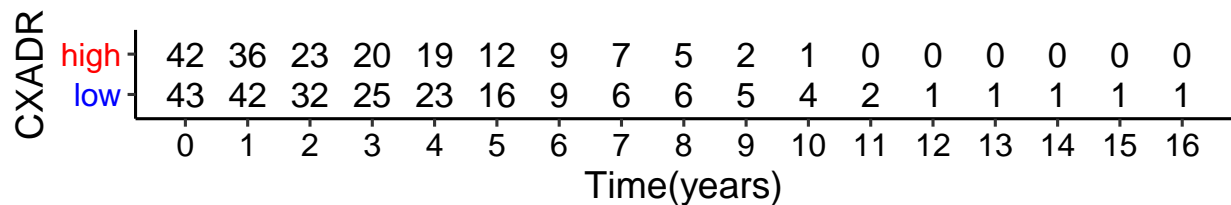

Supplement: Supplementary Document 2 — Kaplan-Meier curve of the 85 malignant genes associated with survival. [file DataSheet_2.zip › Supplementary Document 2/sur.CXADR.pdf]

DLX3 + high + low

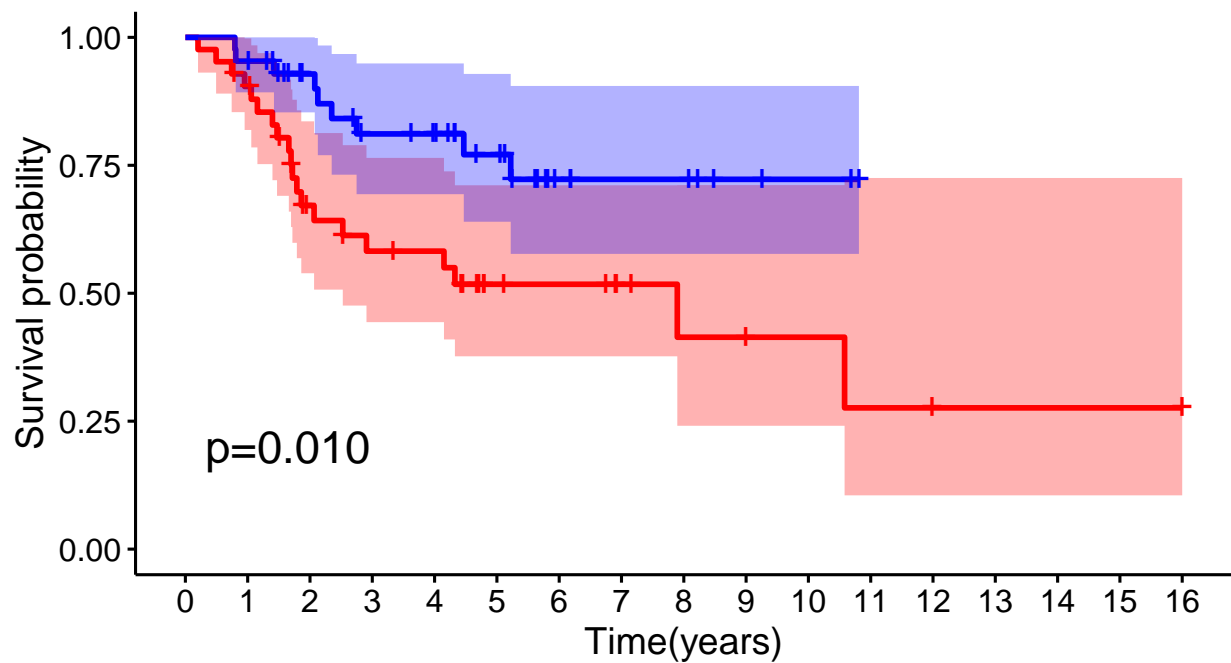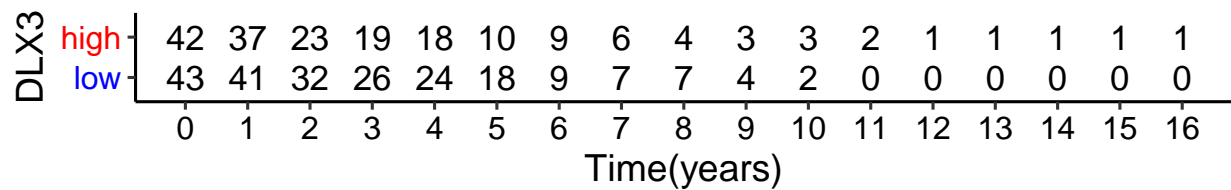

Supplement: Supplementary Document 2 — Kaplan-Meier curve of the 85 malignant genes associated with survival. [file DataSheet_2.zip › Supplementary Document 2/sur.DLX3.pdf]

DMRT2 high low

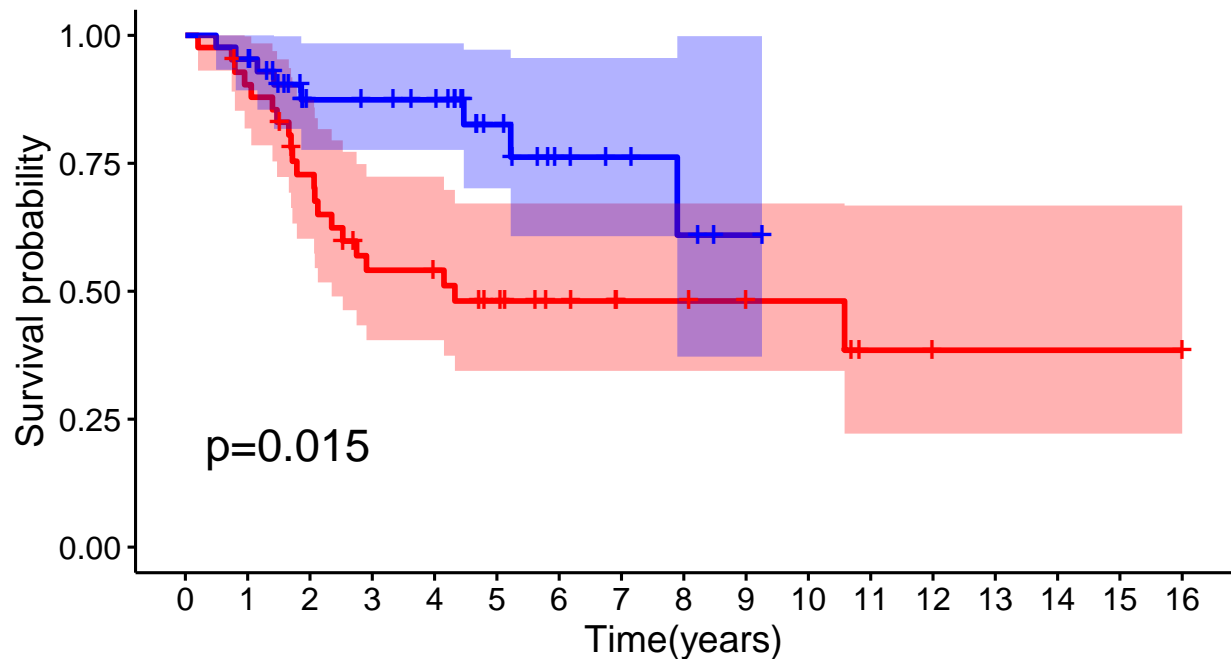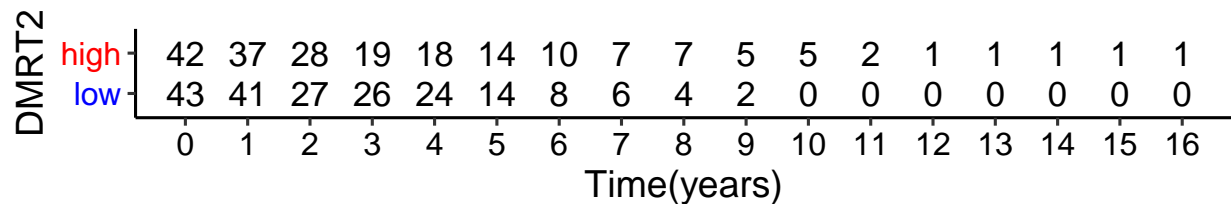

Supplement: Supplementary Document 2 — Kaplan-Meier curve of the 85 malignant genes associated with survival. [file DataSheet_2.zip › Supplementary Document 2/sur.DMRT2.pdf]

DPEP1 high low

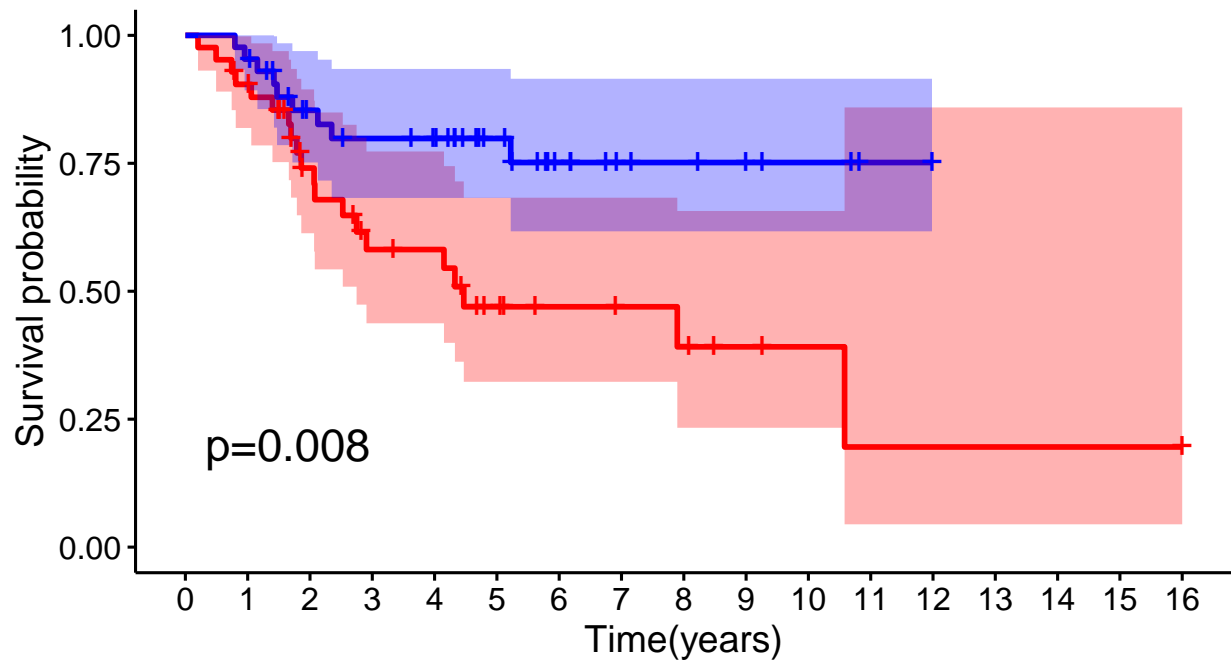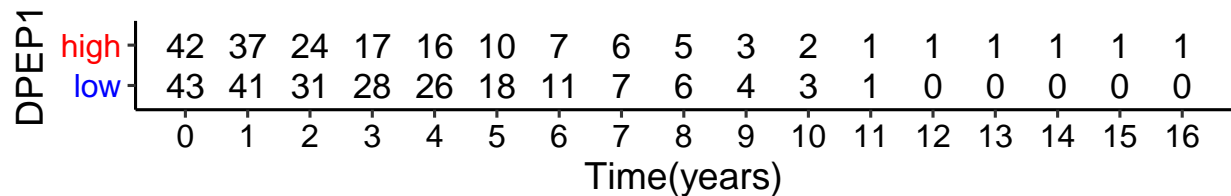

Supplement: Supplementary Document 2 — Kaplan-Meier curve of the 85 malignant genes associated with survival. [file DataSheet_2.zip › Supplementary Document 2/sur.DPEP1.pdf]

ERMP1 + high + low

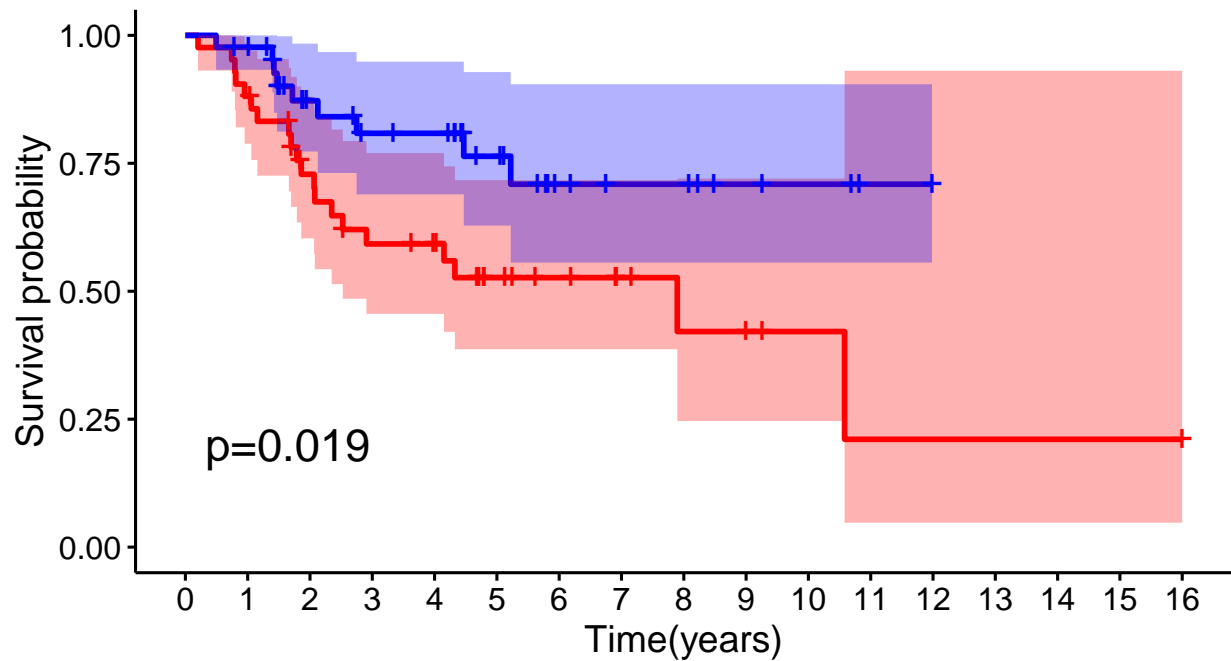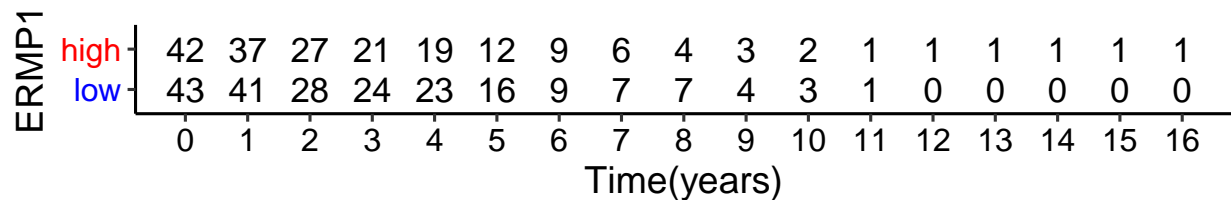

Supplement: Supplementary Document 2 — Kaplan-Meier curve of the 85 malignant genes associated with survival. [file DataSheet_2.zip › Supplementary Document 2/sur.ERMP1.pdf]

FAM166B + high + low

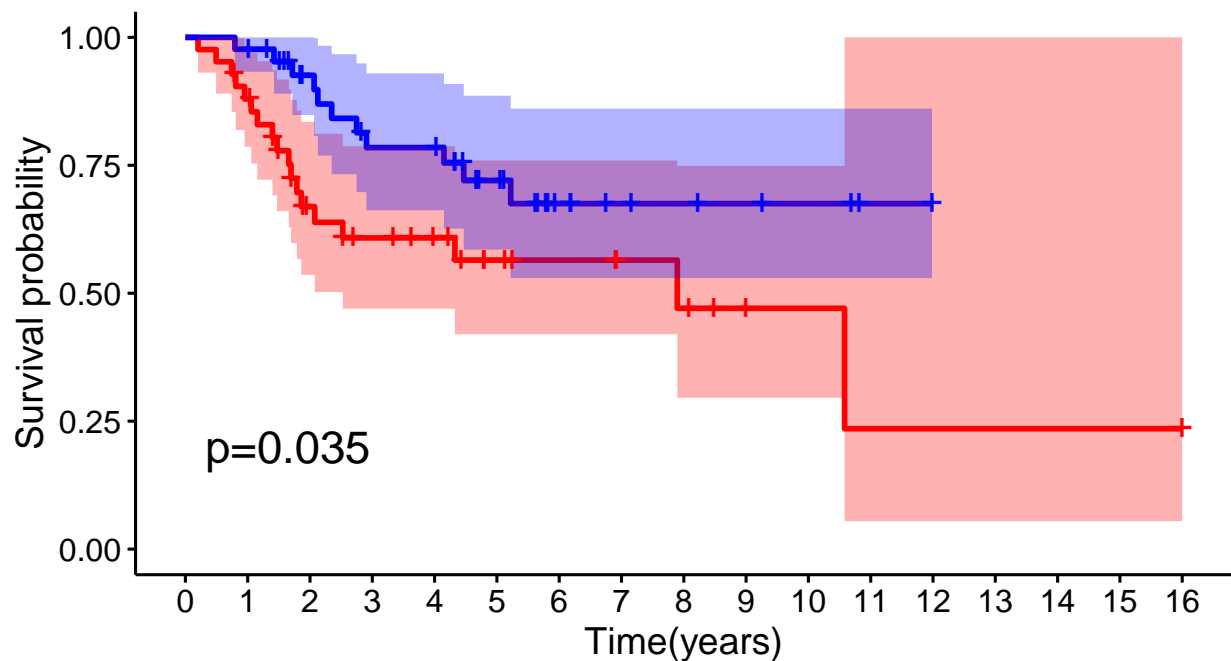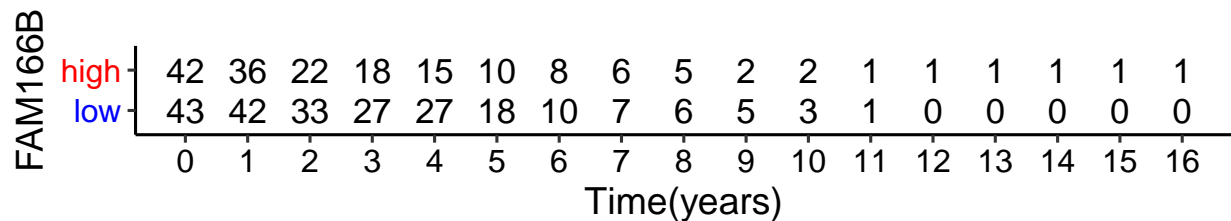

Supplement: Supplementary Document 2 — Kaplan-Meier curve of the 85 malignant genes associated with survival. [file DataSheet_2.zip › Supplementary Document 2/sur.FAM166B.pdf]

Survival probability

FAM91A1 high low

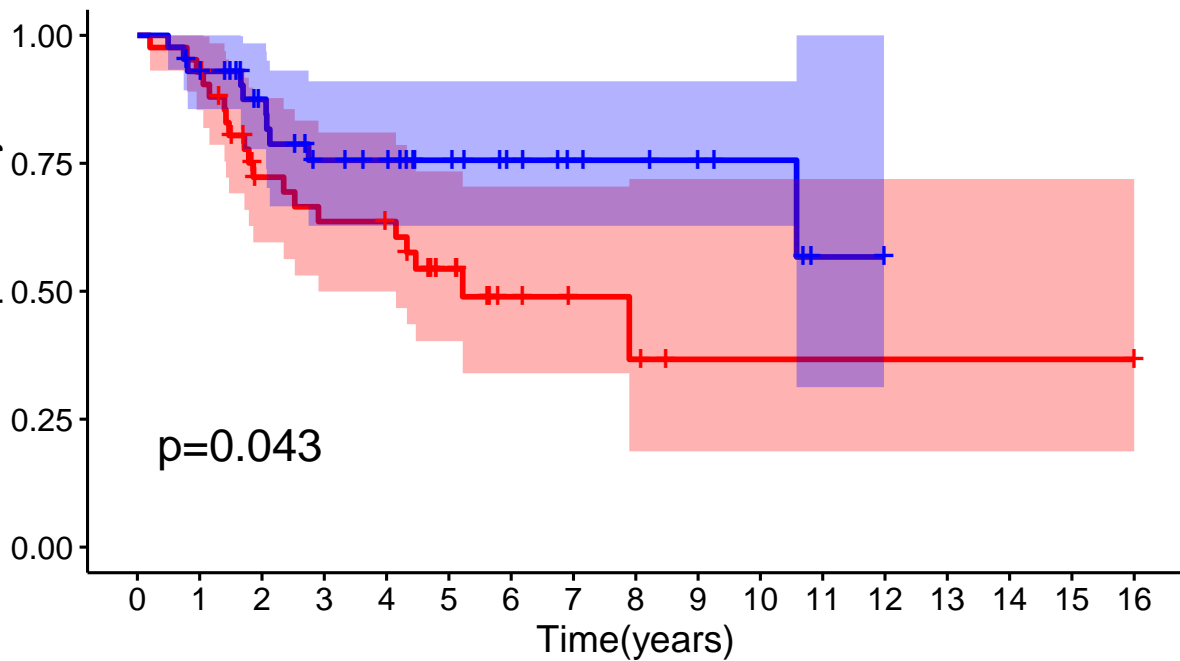

FAM91A1

high  
low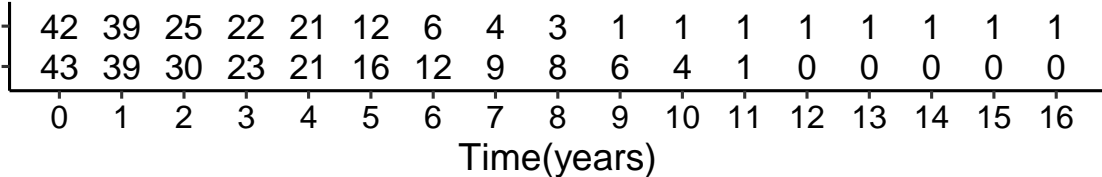

Supplement: Supplementary Document 2 — Kaplan-Meier curve of the 85 malignant genes associated with survival. [file DataSheet_2.zip › Supplementary Document 2/sur.FAM91A1.pdf]

FDX1 + high + low

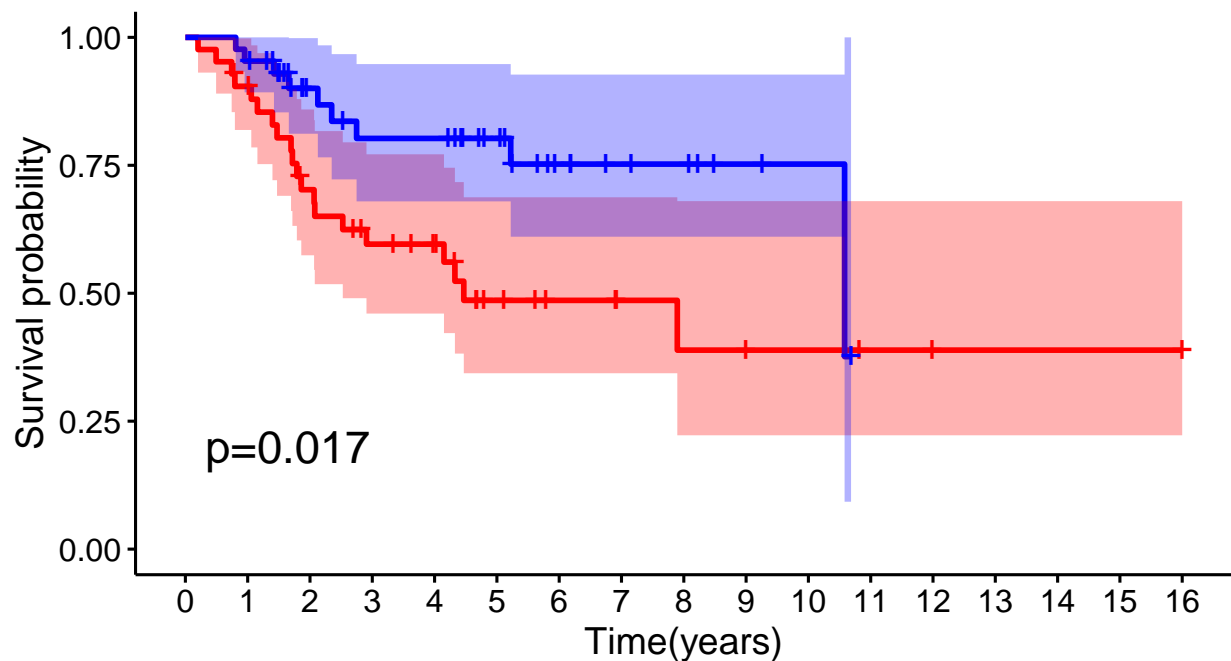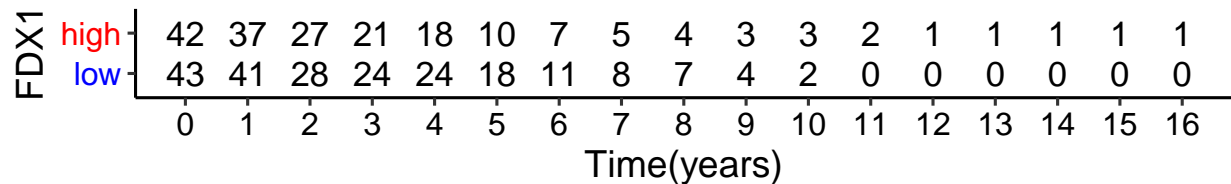

Supplement: Supplementary Document 2 — Kaplan-Meier curve of the 85 malignant genes associated with survival. [file DataSheet_2.zip › Supplementary Document 2/sur.FDX1.pdf]

FLJ45513 + high + low

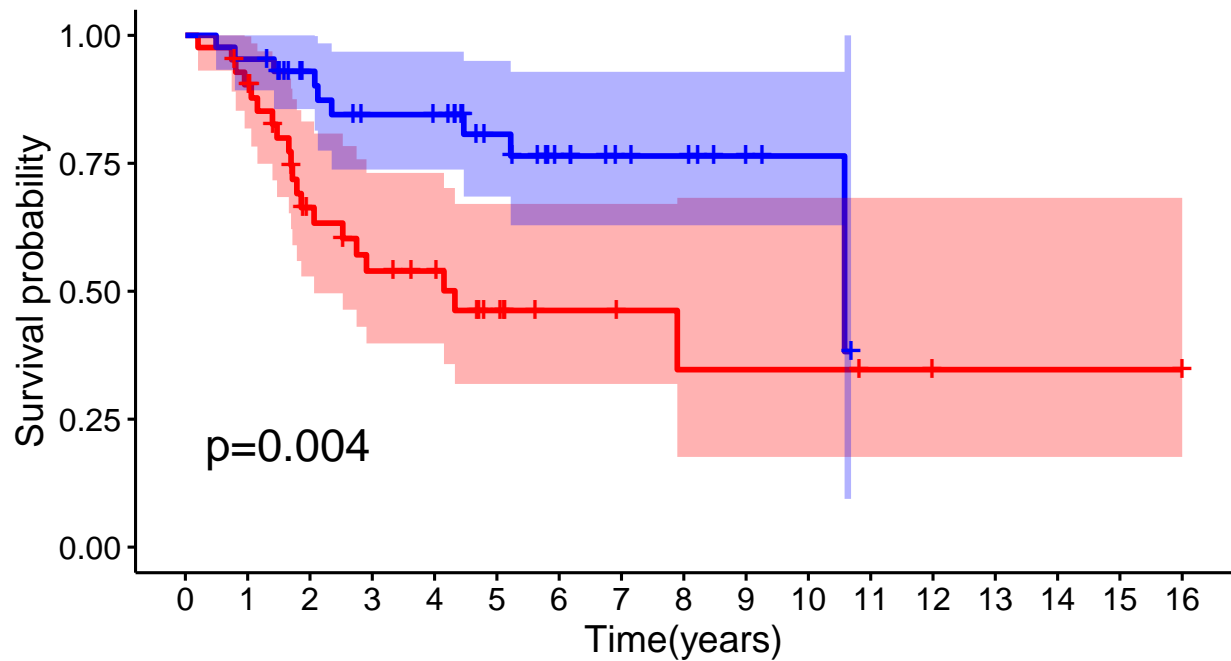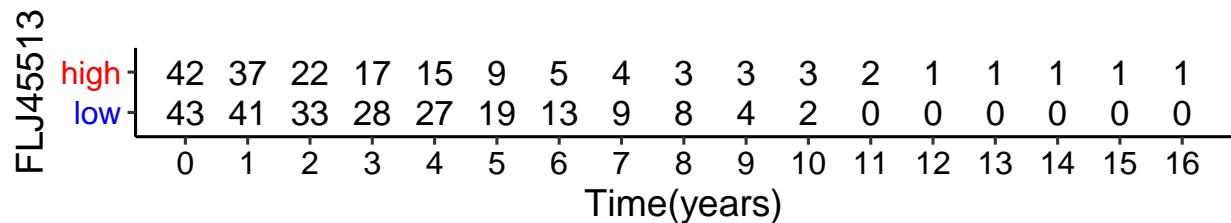

Supplement: Supplementary Document 2 — Kaplan-Meier curve of the 85 malignant genes associated with survival. [file DataSheet_2.zip › Supplementary Document 2/sur.FLJ45513.pdf]

FXYP6 + high + low

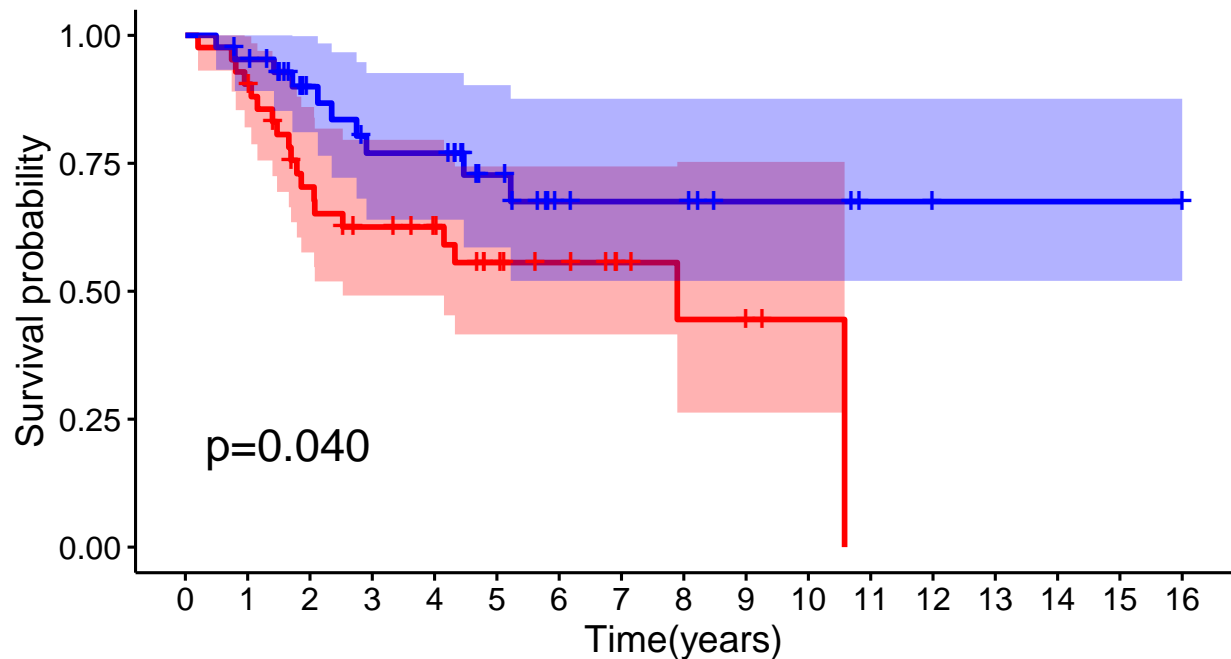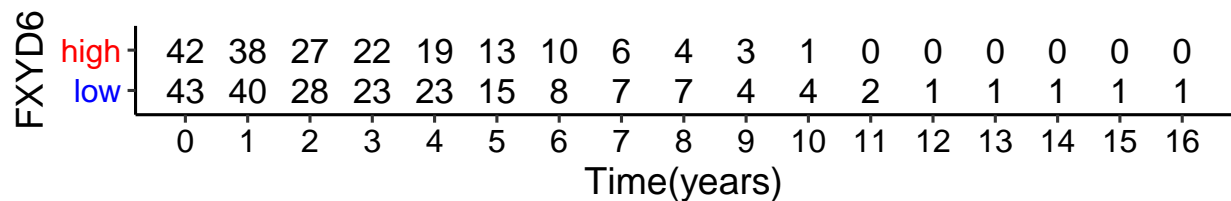

Supplement: Supplementary Document 2 — Kaplan-Meier curve of the 85 malignant genes associated with survival. [file DataSheet_2.zip › Supplementary Document 2/sur.FXYD6.pdf]

GNG4 + high + low

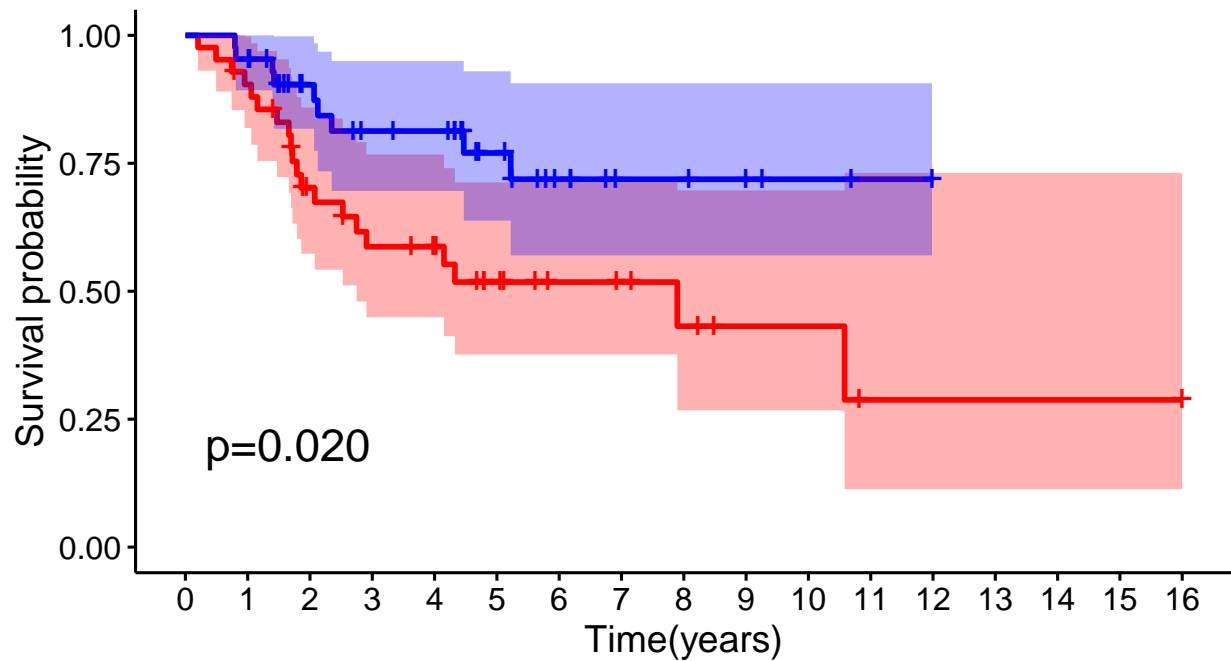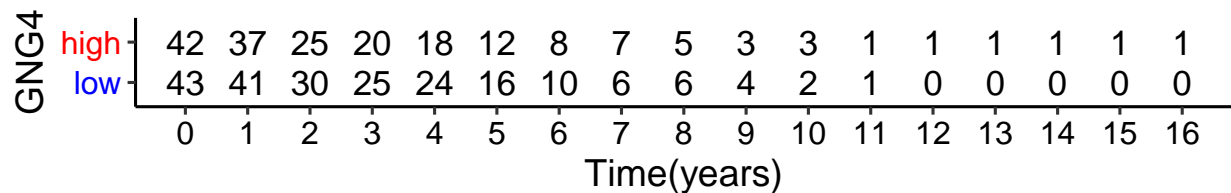

Supplement: Supplementary Document 2 — Kaplan-Meier curve of the 85 malignant genes associated with survival. [file DataSheet_2.zip › Supplementary Document 2/sur.GNG4.pdf]

GRAMD1B + high + low

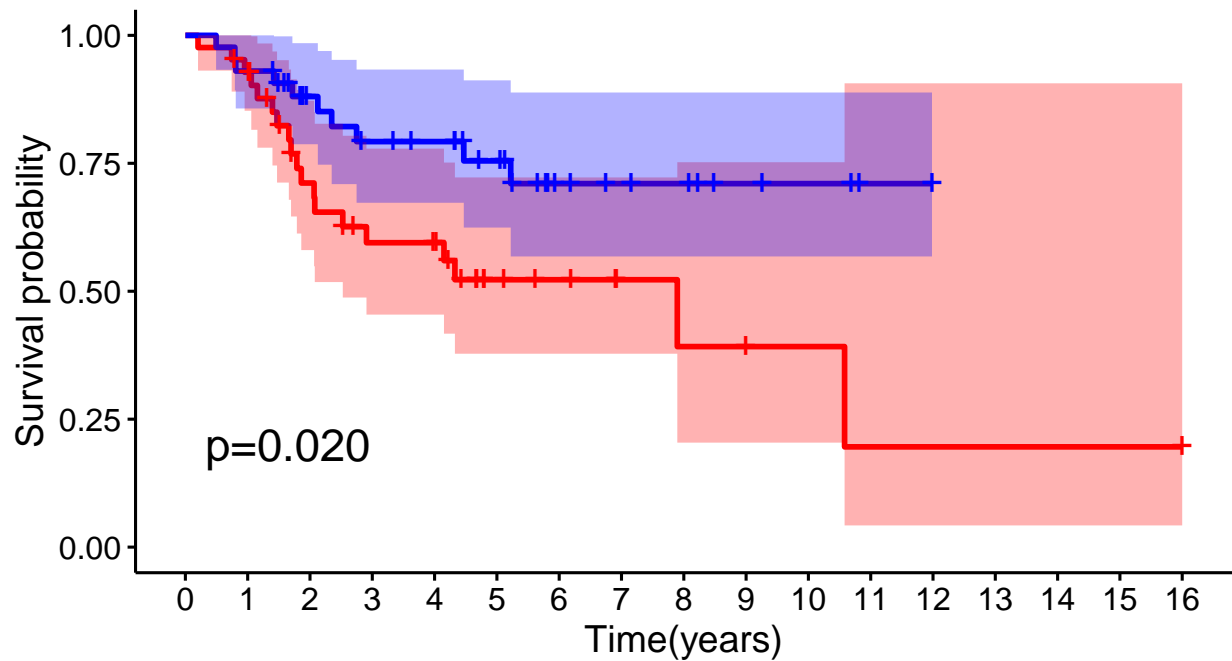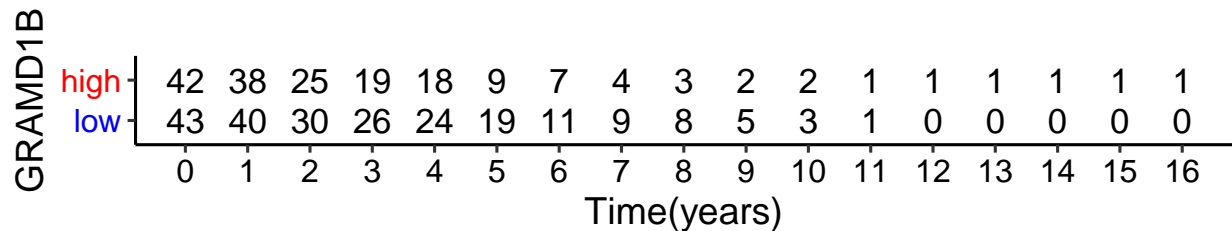

Supplement: Supplementary Document 2 — Kaplan-Meier curve of the 85 malignant genes associated with survival. [file DataSheet_2.zip › Supplementary Document 2/sur.GRAMD1B.pdf]

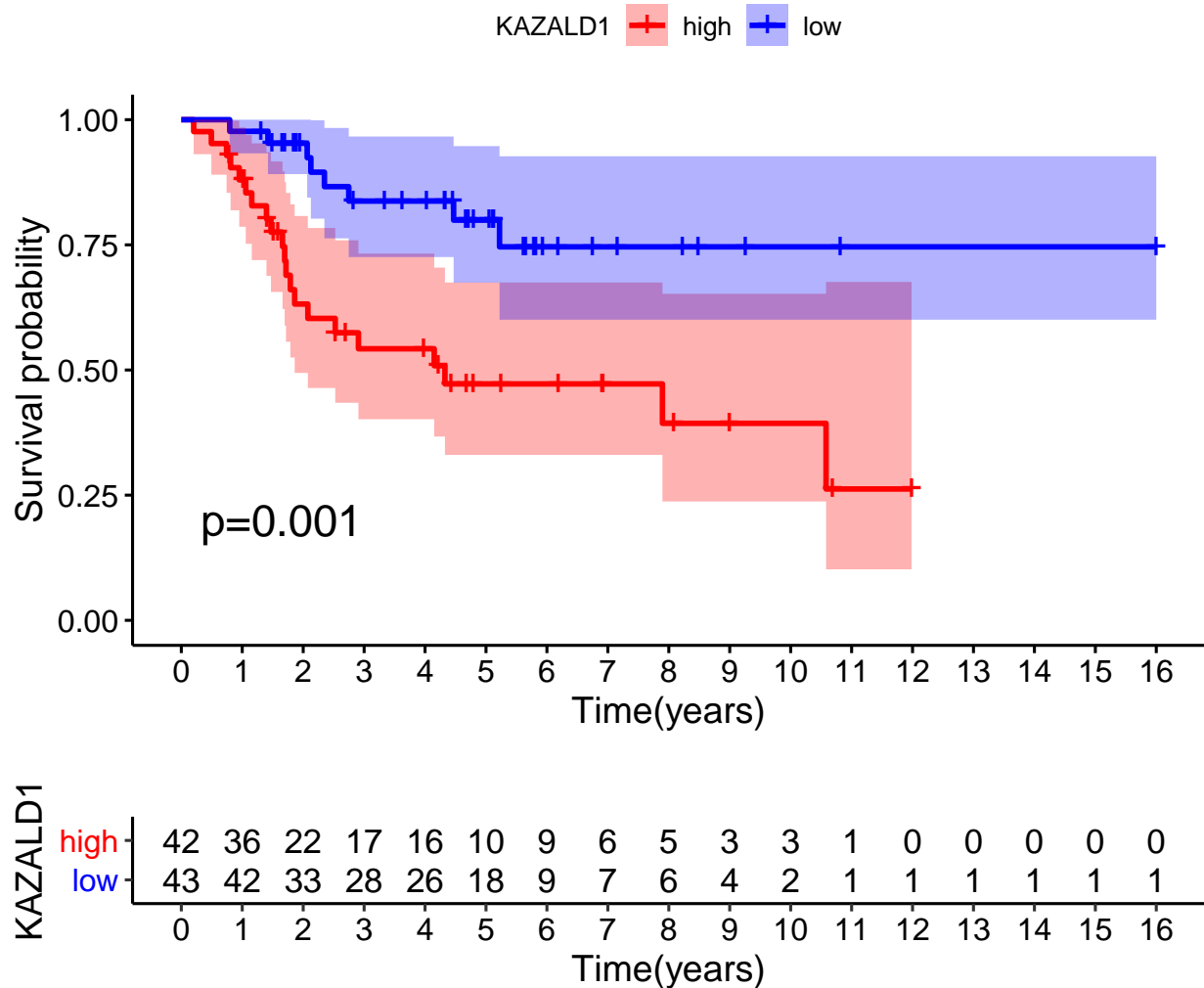

Supplement: Supplementary Document 2 — Kaplan-Meier curve of the 85 malignant genes associated with survival. [file DataSheet_2.zip › Supplementary Document 2/sur.KAZALD1.pdf]

LGR6 high low

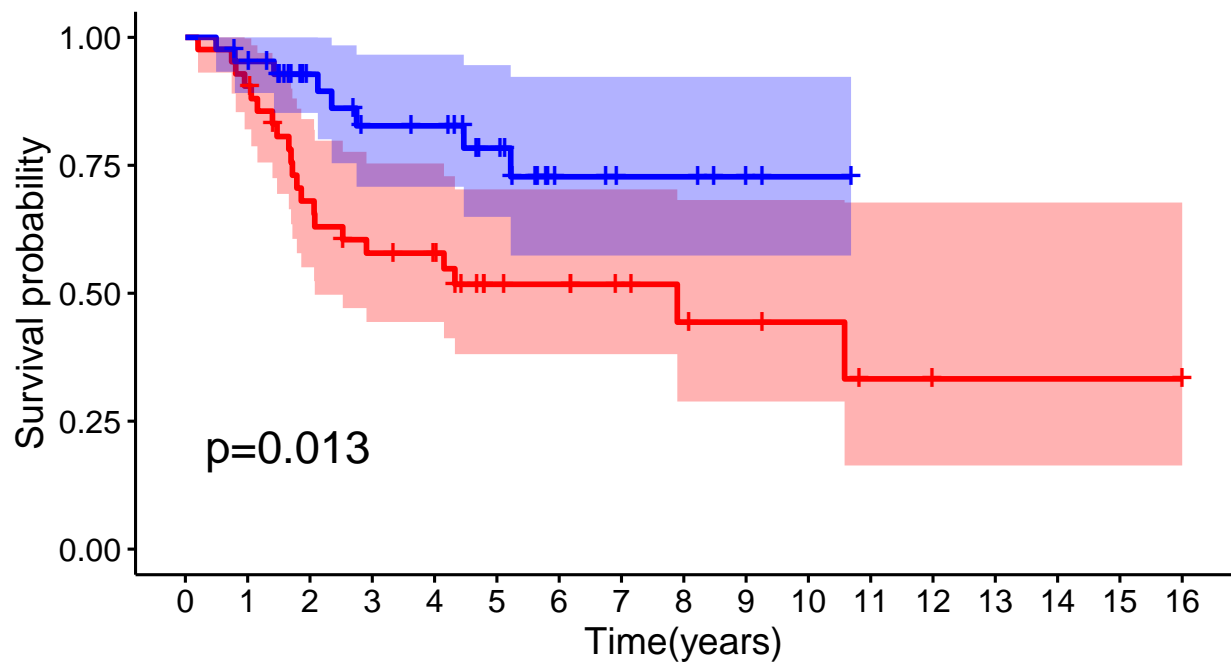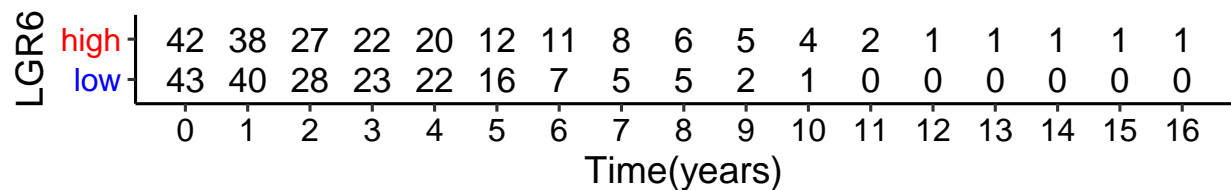

Supplement: Supplementary Document 2 — Kaplan-Meier curve of the 85 malignant genes associated with survival. [file DataSheet_2.zip › Supplementary Document 2/sur.LGR6.pdf]

LINC02044 + high + low

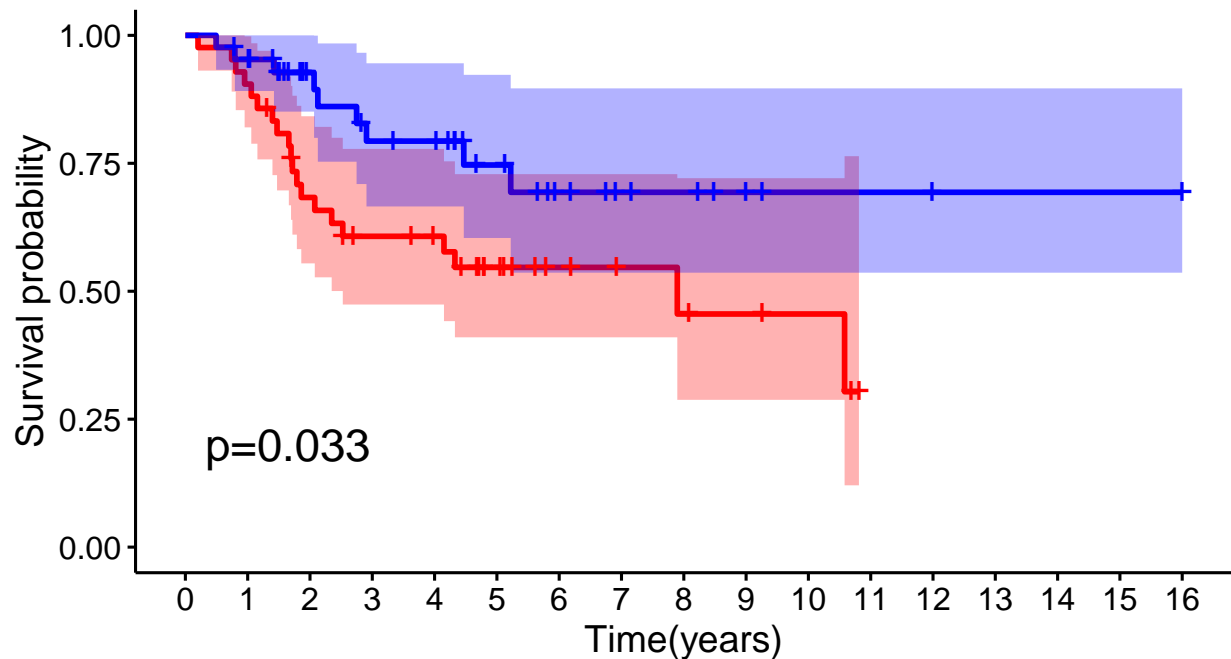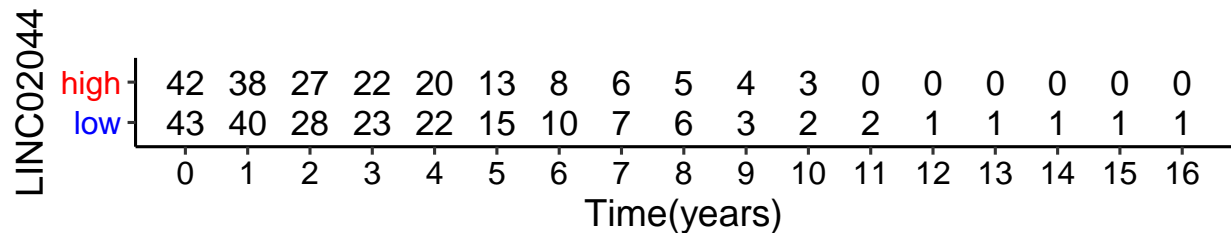

Supplement: Supplementary Document 2 — Kaplan-Meier curve of the 85 malignant genes associated with survival. [file DataSheet_2.zip › Supplementary Document 2/sur.LINC02044.pdf]

LOXL4 + high + low

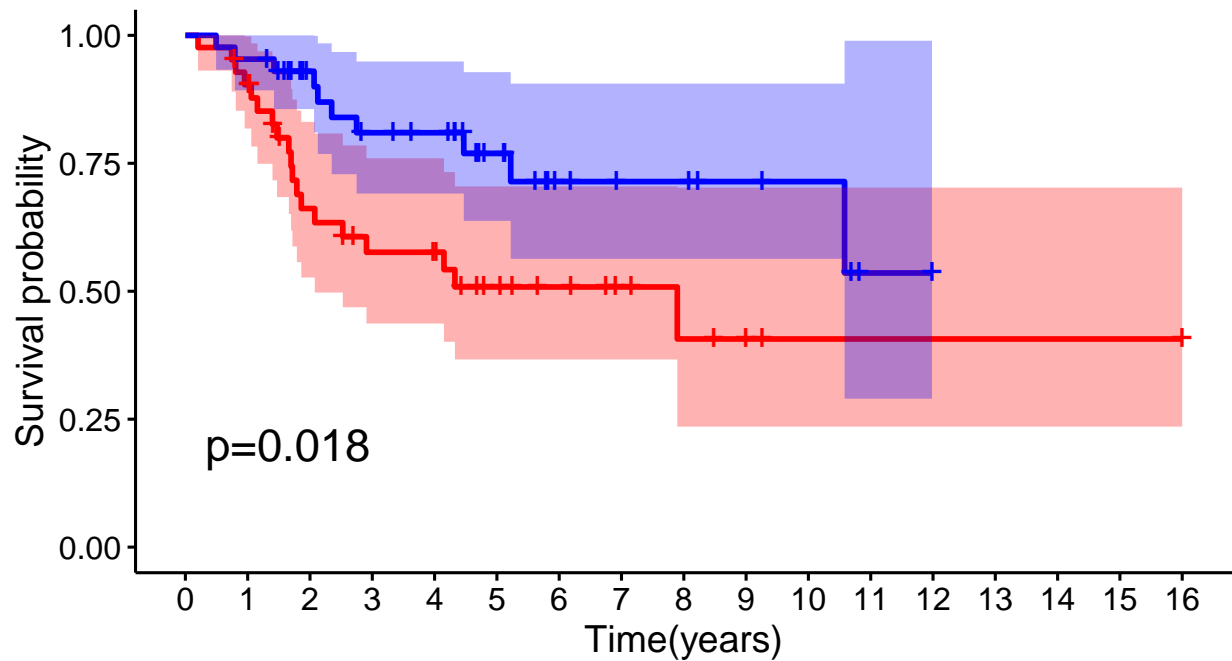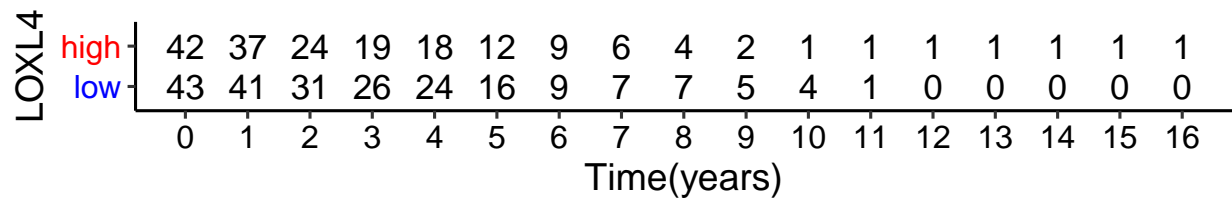

Supplement: Supplementary Document 2 — Kaplan-Meier curve of the 85 malignant genes associated with survival. [file DataSheet_2.zip › Supplementary Document 2/sur.LOXL4.pdf]

LTK high low

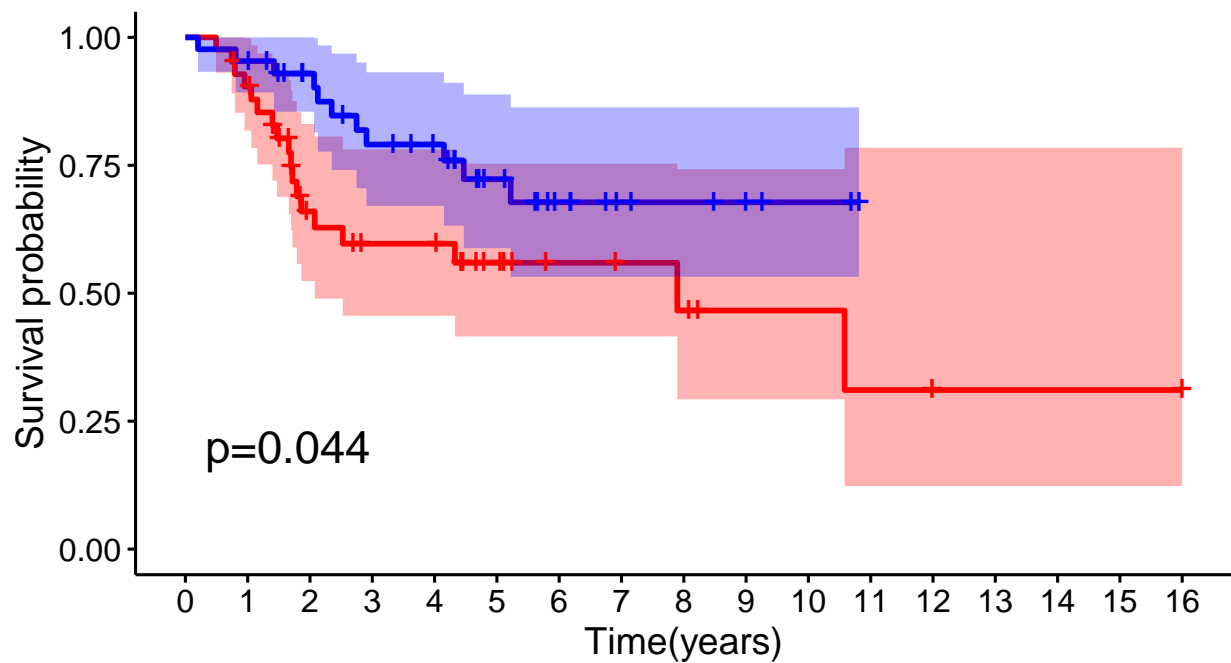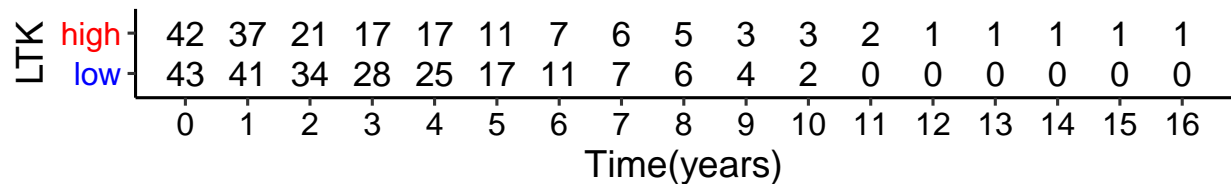

Supplement: Supplementary Document 2 — Kaplan-Meier curve of the 85 malignant genes associated with survival. [file DataSheet_2.zip › Supplementary Document 2/sur.LTK.pdf]

MCAM + high + low

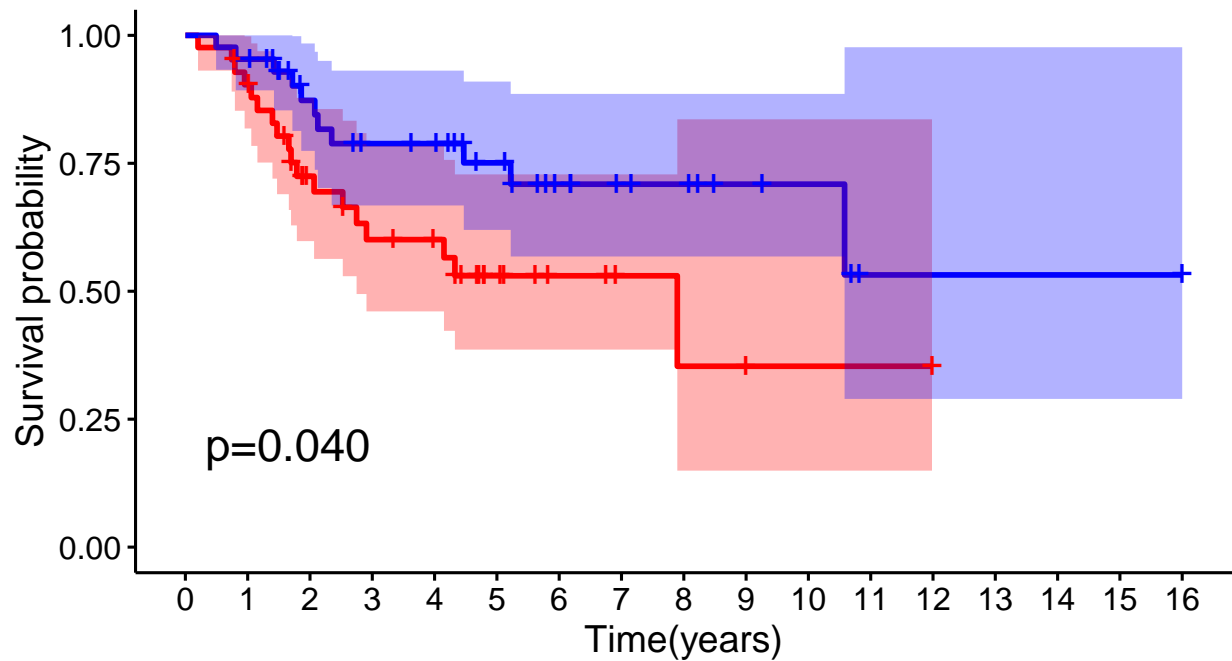

MCAM

|      |    |    |    |    |    |    |    |    |   |   |    |    |    |    |    |    |    |
|------|----|----|----|----|----|----|----|----|---|---|----|----|----|----|----|----|----|
| high | 42 | 37 | 24 | 19 | 17 | 9  | 5  | 3  | 2 | 1 | 1  | 1  | 0  | 0  | 0  | 0  | 0  |
| low  | 43 | 41 | 31 | 26 | 25 | 19 | 13 | 10 | 9 | 6 | 4  | 1  | 1  | 1  | 1  | 1  | 1  |
|      | 0  | 1  | 2  | 3  | 4  | 5  | 6  | 7  | 8 | 9 | 10 | 11 | 12 | 13 | 14 | 15 | 16 |

Time(years)

Supplement: Supplementary Document 2 — Kaplan-Meier curve of the 85 malignant genes associated with survival. [file DataSheet_2.zip › Supplementary Document 2/sur.MCAM.pdf]

MT1A + high + low

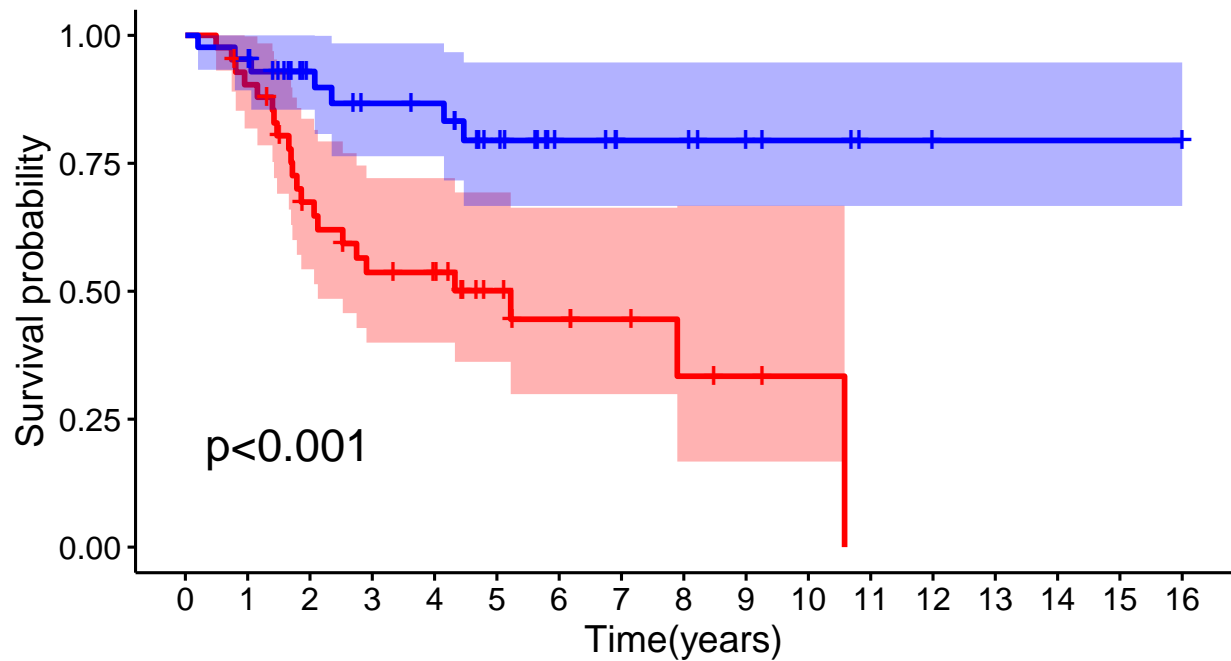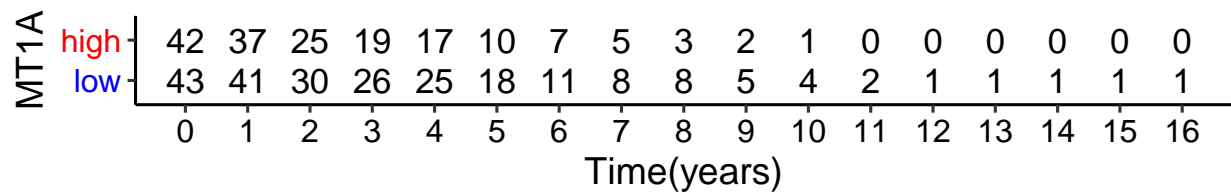

Supplement: Supplementary Document 2 — Kaplan-Meier curve of the 85 malignant genes associated with survival. [file DataSheet_2.zip › Supplementary Document 2/sur.MT1A.pdf]

MTSS1 high low

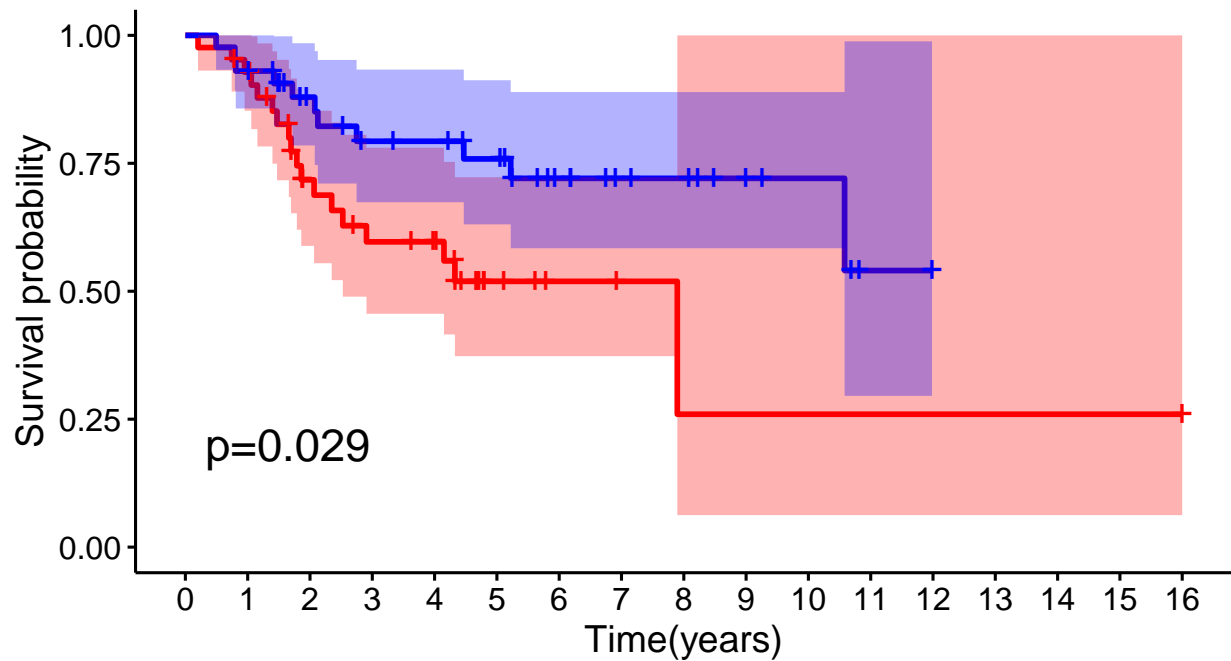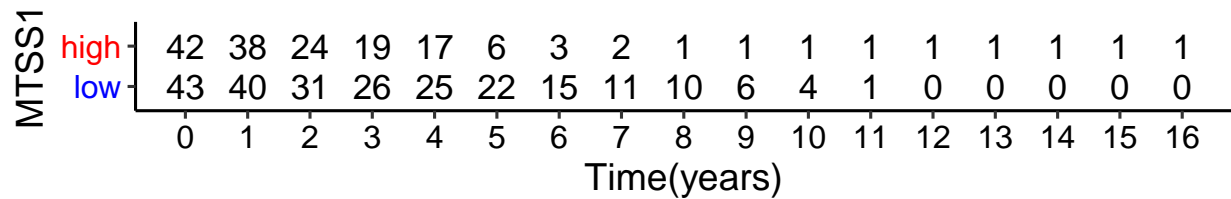

Supplement: Supplementary Document 2 — Kaplan-Meier curve of the 85 malignant genes associated with survival. [file DataSheet_2.zip › Supplementary Document 2/sur.MTSS1.pdf]

NCAM1 high low

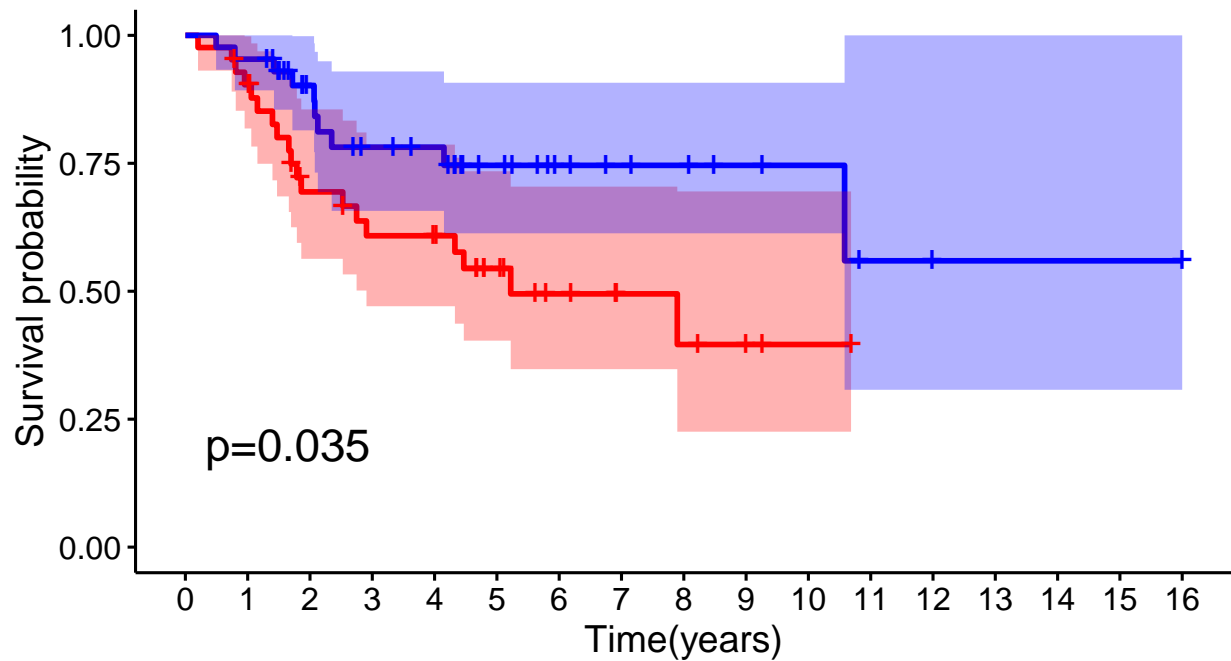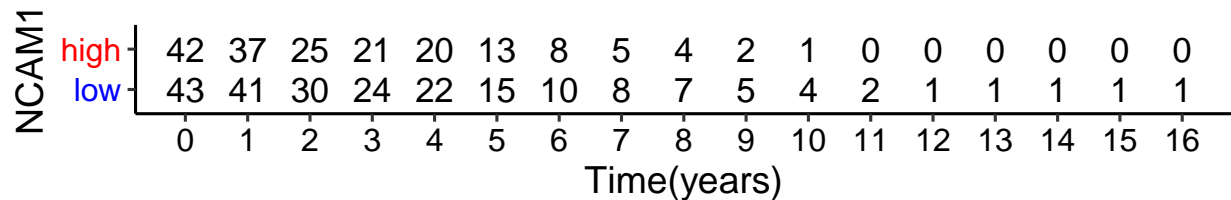

Supplement: Supplementary Document 2 — Kaplan-Meier curve of the 85 malignant genes associated with survival. [file DataSheet_2.zip › Supplementary Document 2/sur.NCAM1.pdf]

NLRP2B high low

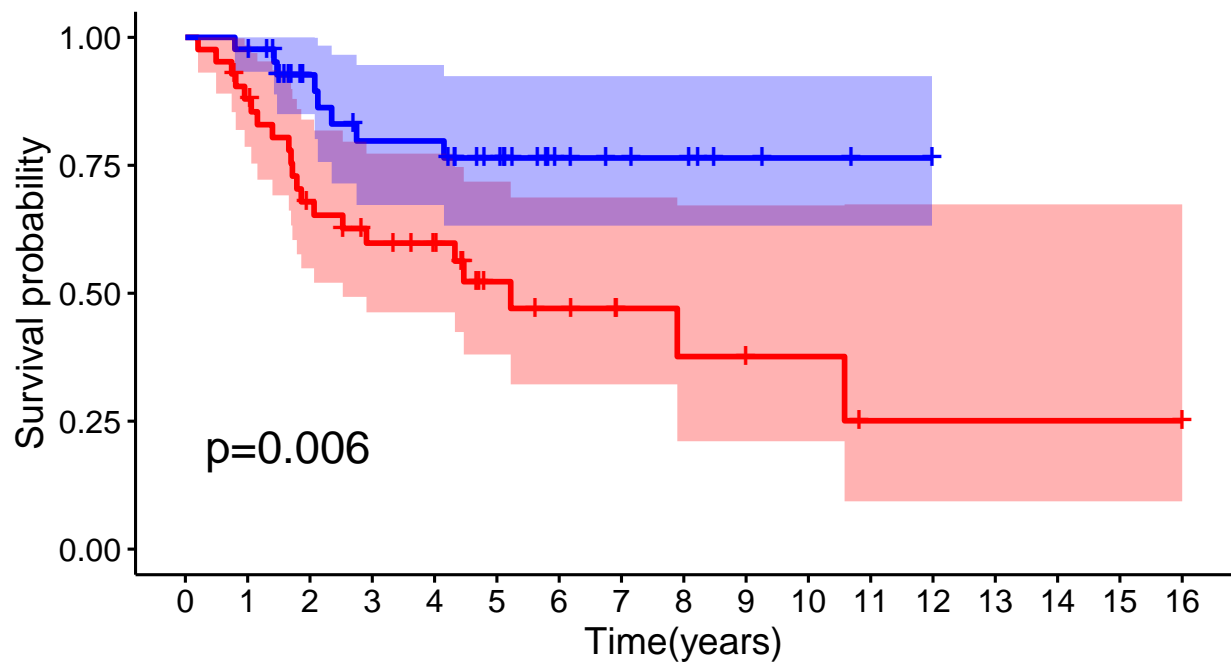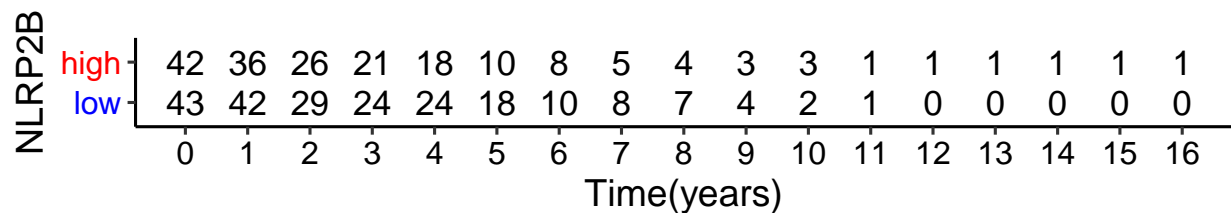

Supplement: Supplementary Document 2 — Kaplan-Meier curve of the 85 malignant genes associated with survival. [file DataSheet_2.zip › Supplementary Document 2/sur.NLRP2B.pdf]

OPN3 + high + low

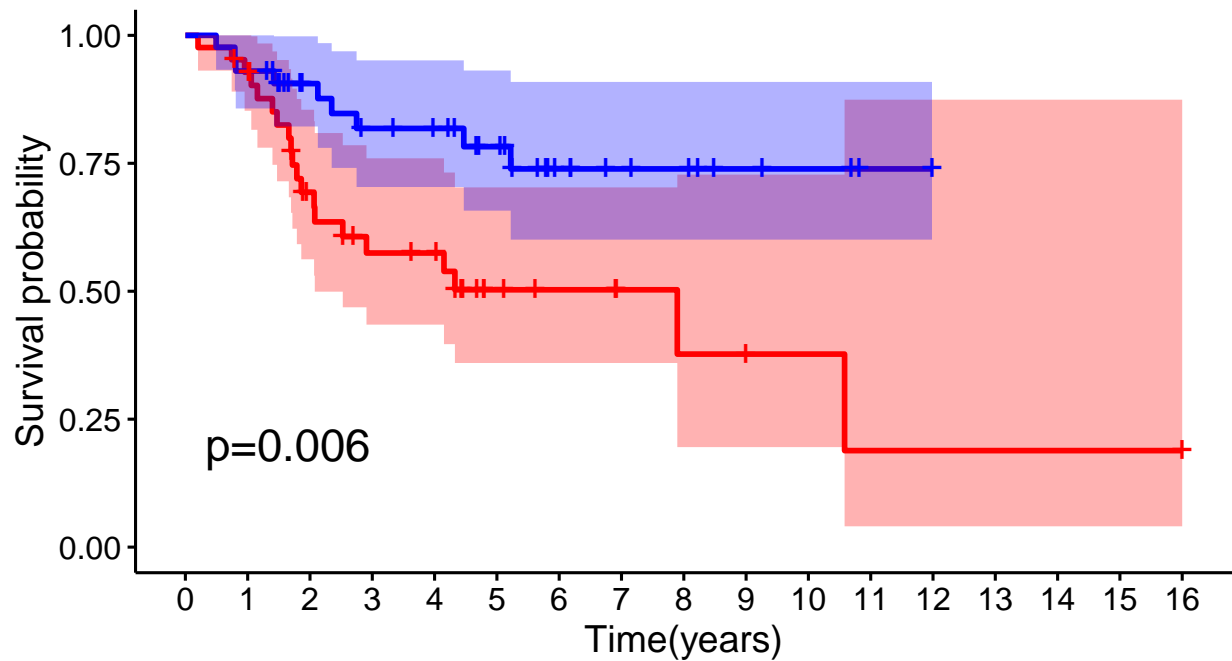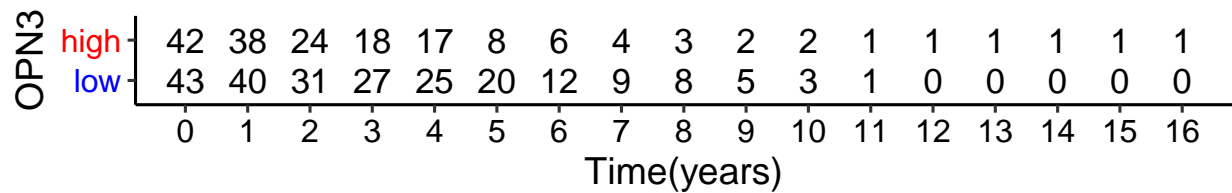

Supplement: Supplementary Document 2 — Kaplan-Meier curve of the 85 malignant genes associated with survival. [file DataSheet_2.zip › Supplementary Document 2/sur.OPN3.pdf]

OXCT2 high low

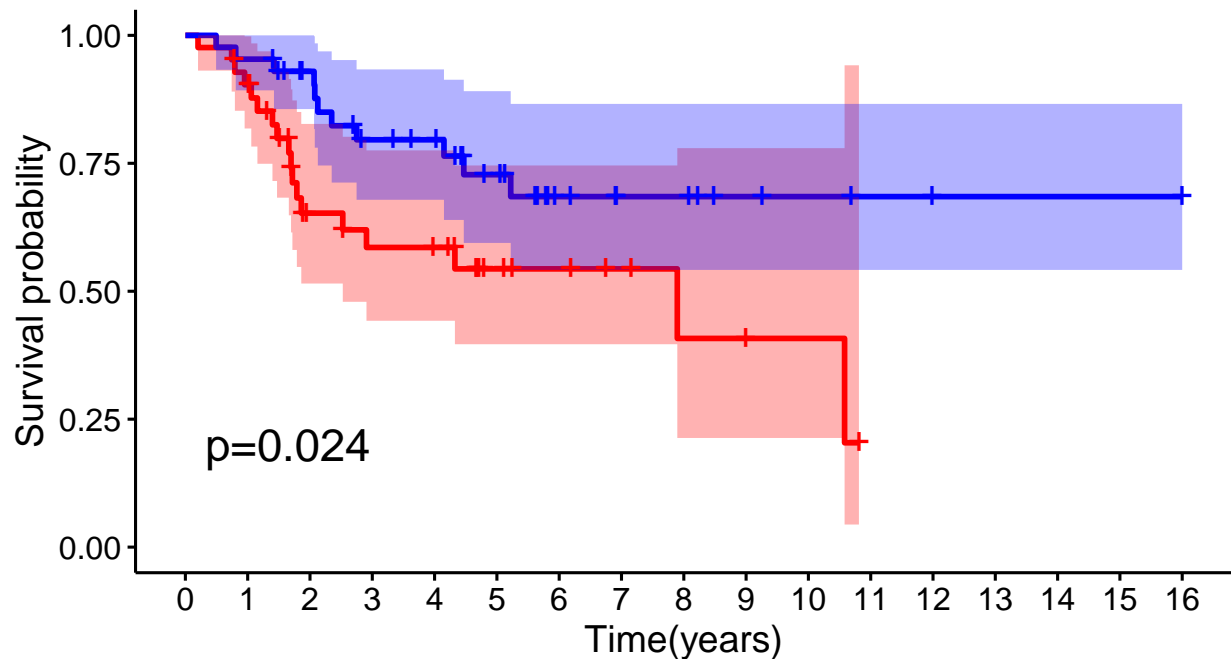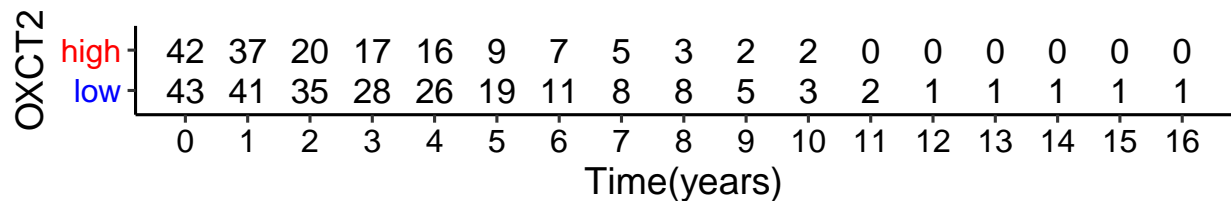

Supplement: Supplementary Document 2 — Kaplan-Meier curve of the 85 malignant genes associated with survival. [file DataSheet_2.zip › Supplementary Document 2/sur.OXCT2.pdf]

PROSER2 + high + low

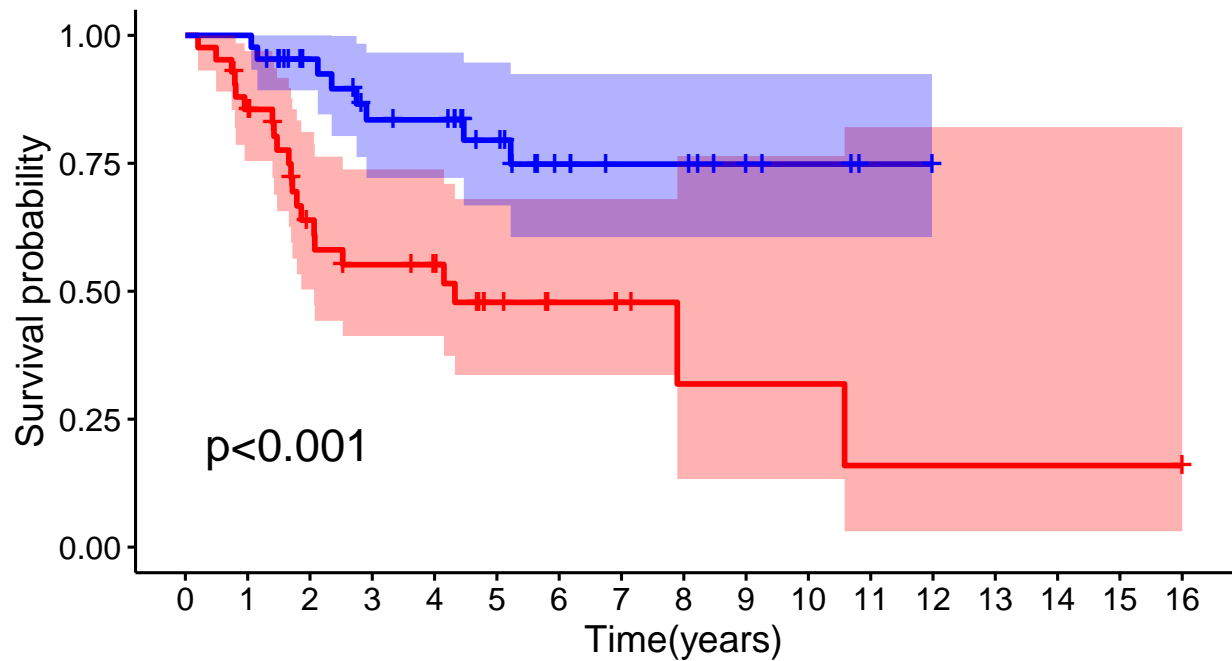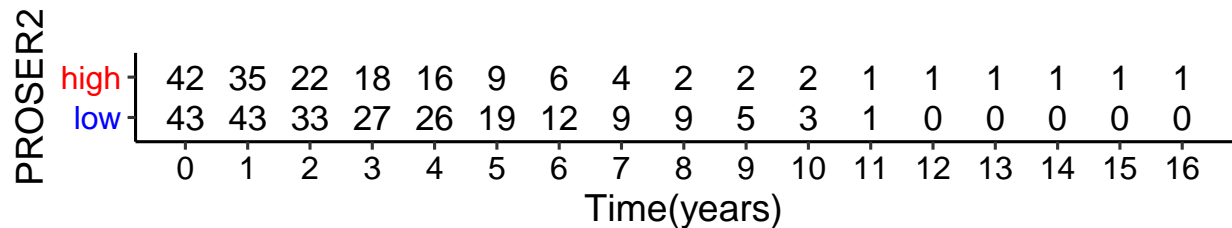

Supplement: Supplementary Document 2 — Kaplan-Meier curve of the 85 malignant genes associated with survival. [file DataSheet_2.zip › Supplementary Document 2/sur.PROSER2.pdf]

RAMP1 high low

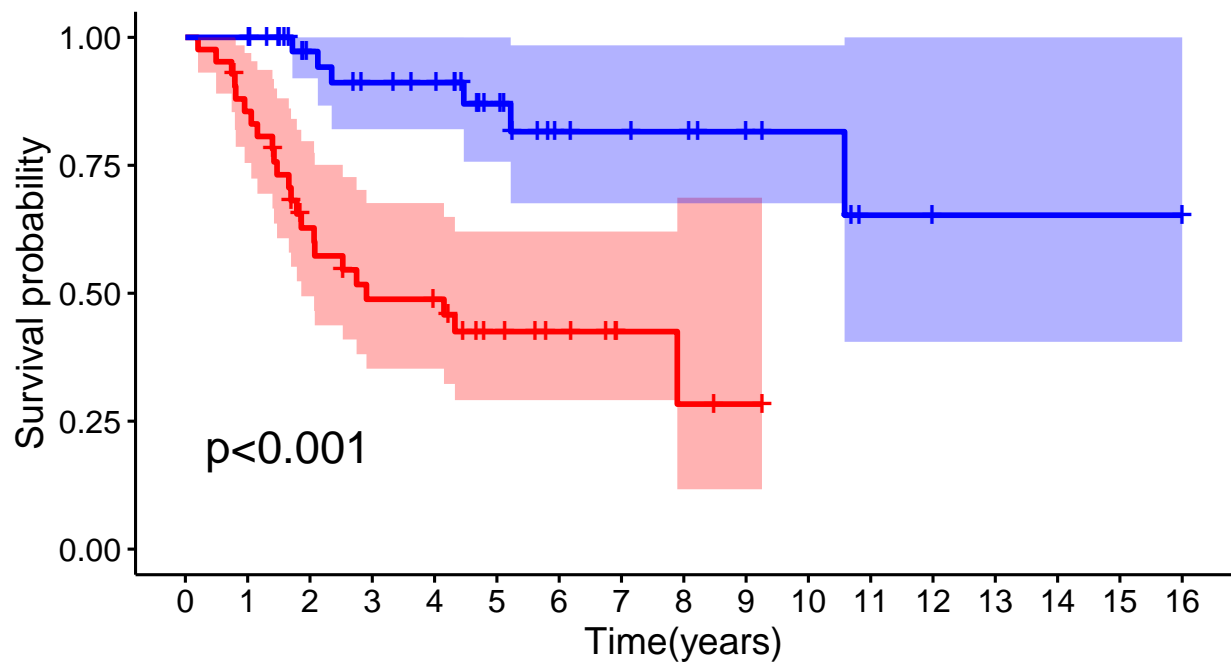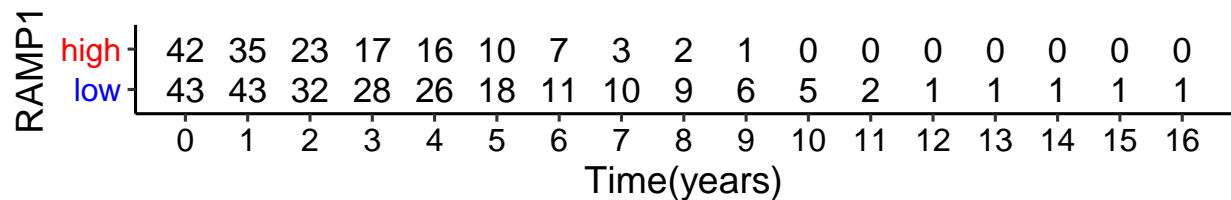

Supplement: Supplementary Document 2 — Kaplan-Meier curve of the 85 malignant genes associated with survival. [file DataSheet_2.zip › Supplementary Document 2/sur.RAMP1.pdf]

RHBDL2 high low

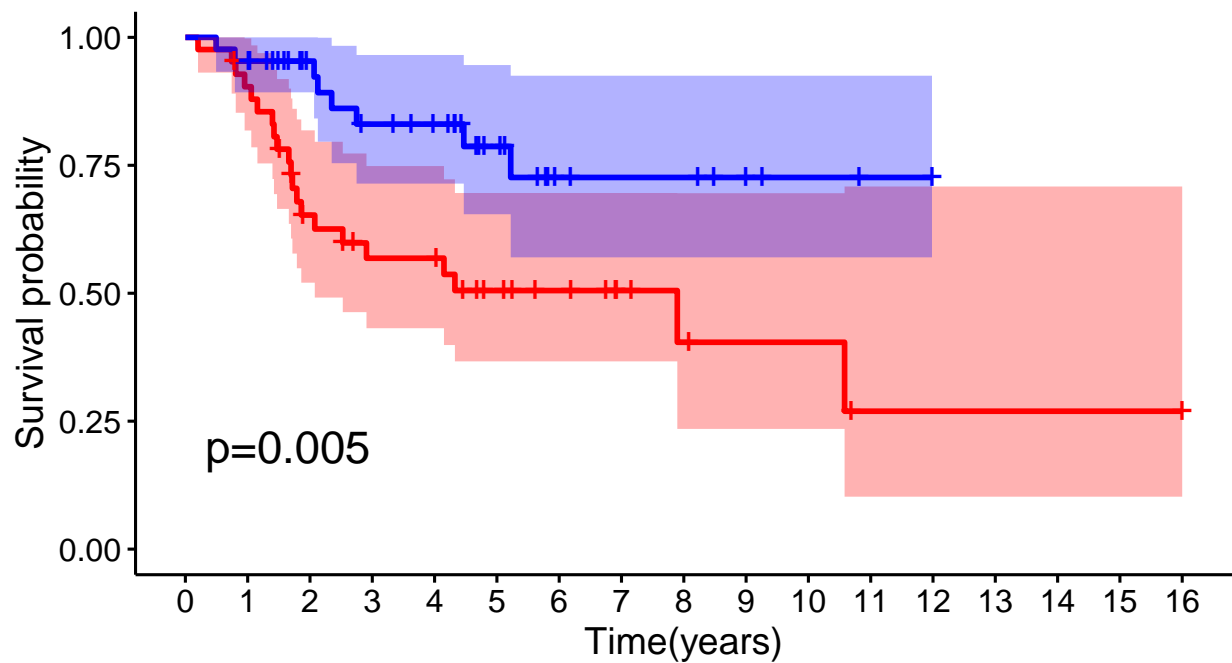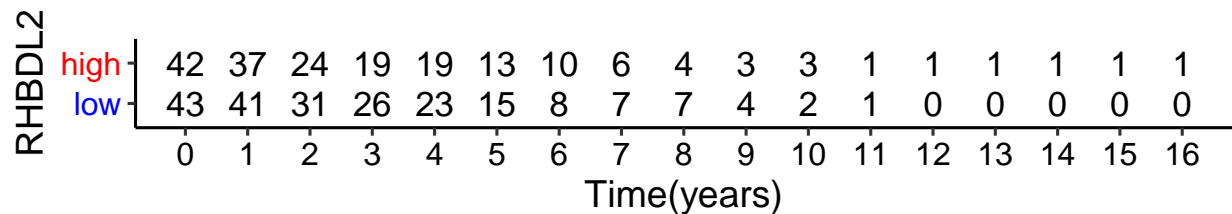

Supplement: Supplementary Document 2 — Kaplan-Meier curve of the 85 malignant genes associated with survival. [file DataSheet_2.zip › Supplementary Document 2/sur.RHBDL2.pdf]

RNF139 high low

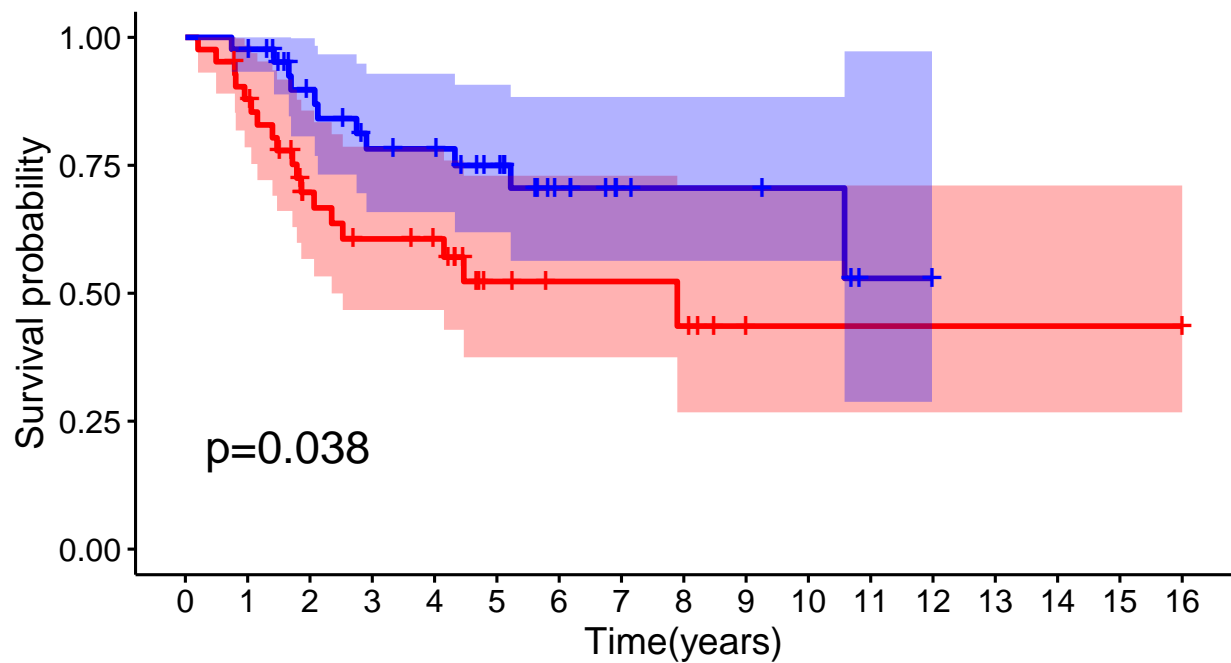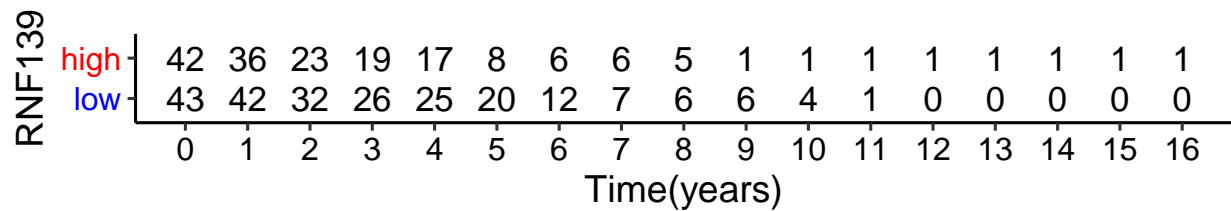

Supplement: Supplementary Document 2 — Kaplan-Meier curve of the 85 malignant genes associated with survival. [file DataSheet_2.zip › Supplementary Document 2/sur.RNF139.pdf]

SATB2 + high + low

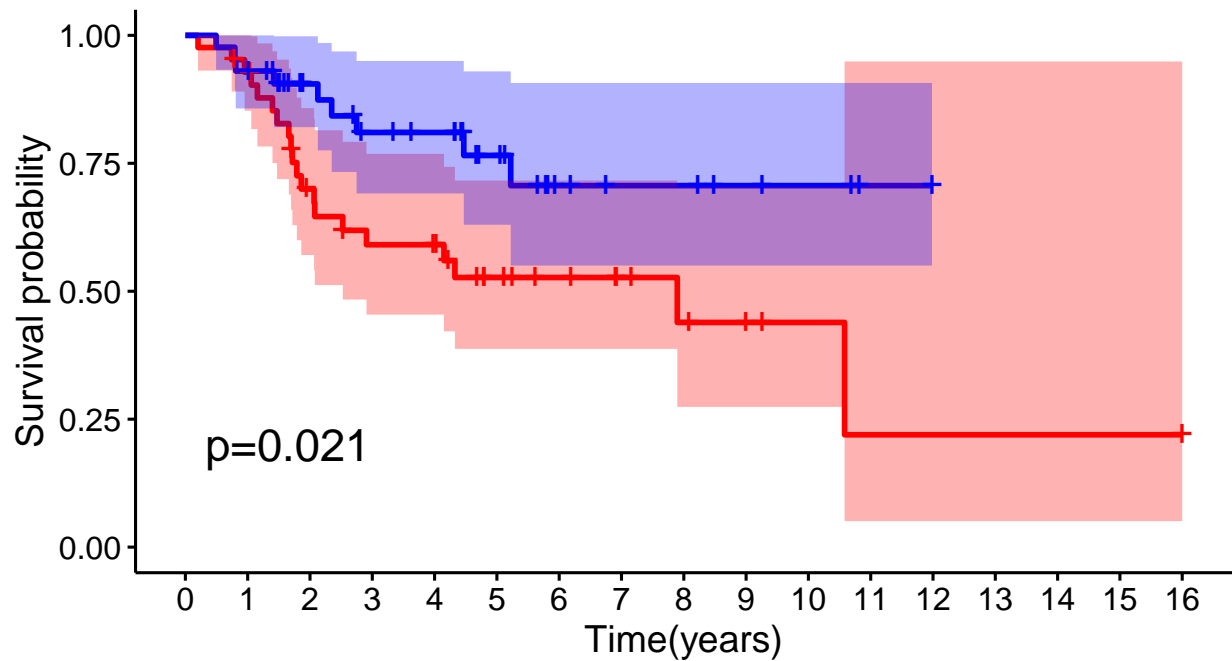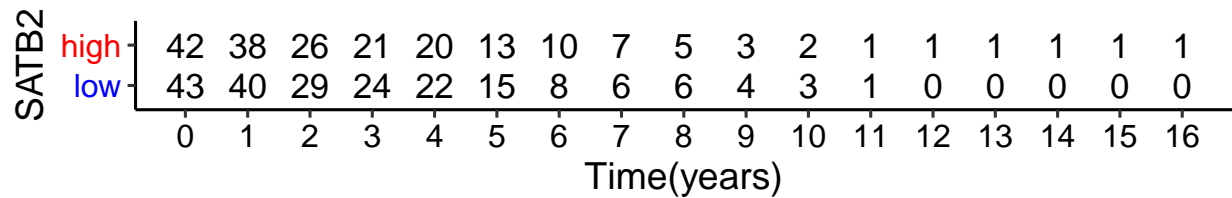

Supplement: Supplementary Document 2 — Kaplan-Meier curve of the 85 malignant genes associated with survival. [file DataSheet_2.zip › Supplementary Document 2/sur.SATB2.pdf]

SEMA5A + high + low

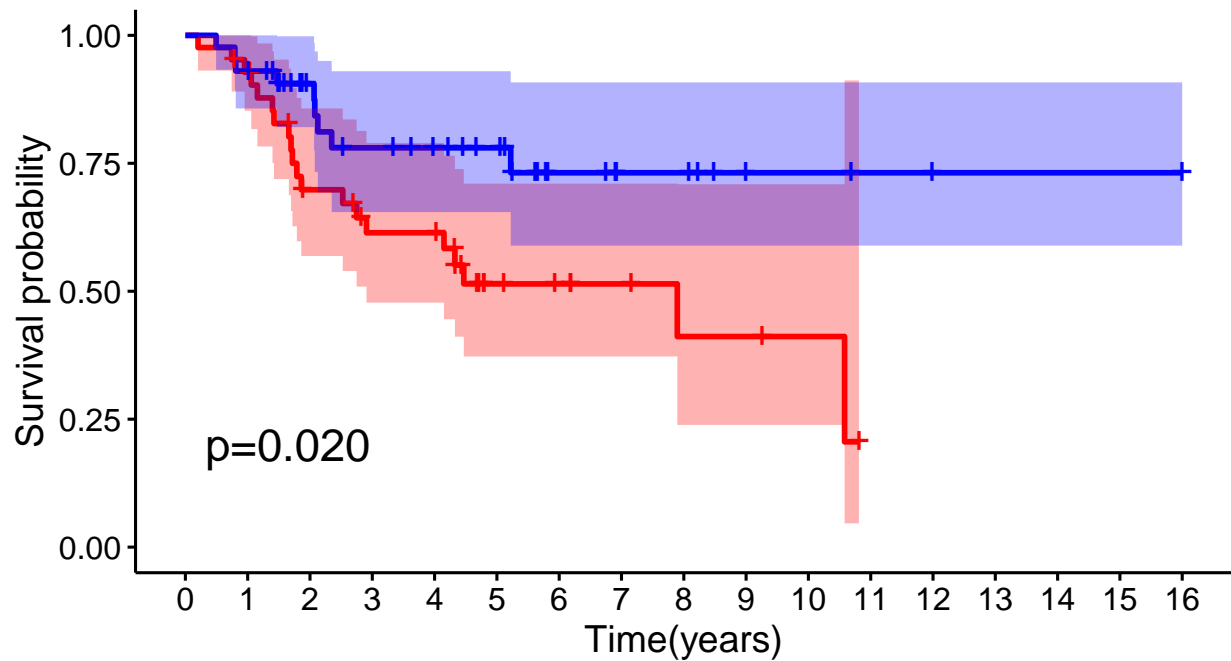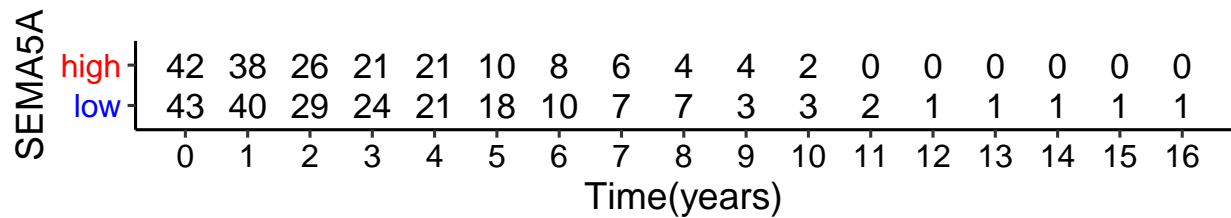

Supplement: Supplementary Document 2 — Kaplan-Meier curve of the 85 malignant genes associated with survival. [file DataSheet_2.zip › Supplementary Document 2/sur.SEMA5A.pdf]

SGMS2 + high + low

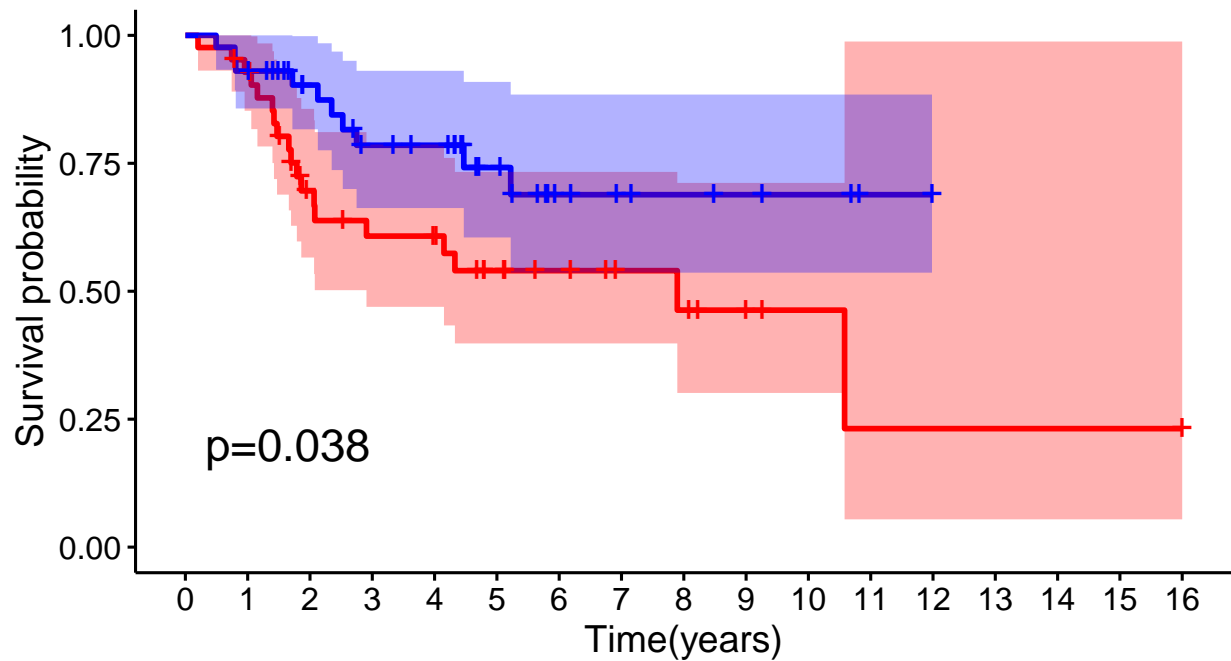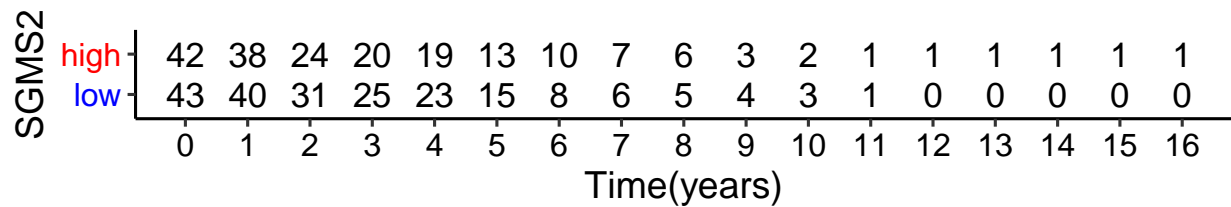

Supplement: Supplementary Document 2 — Kaplan-Meier curve of the 85 malignant genes associated with survival. [file DataSheet_2.zip › Supplementary Document 2/sur.SGMS2.pdf]

SLC8A3 + high + low

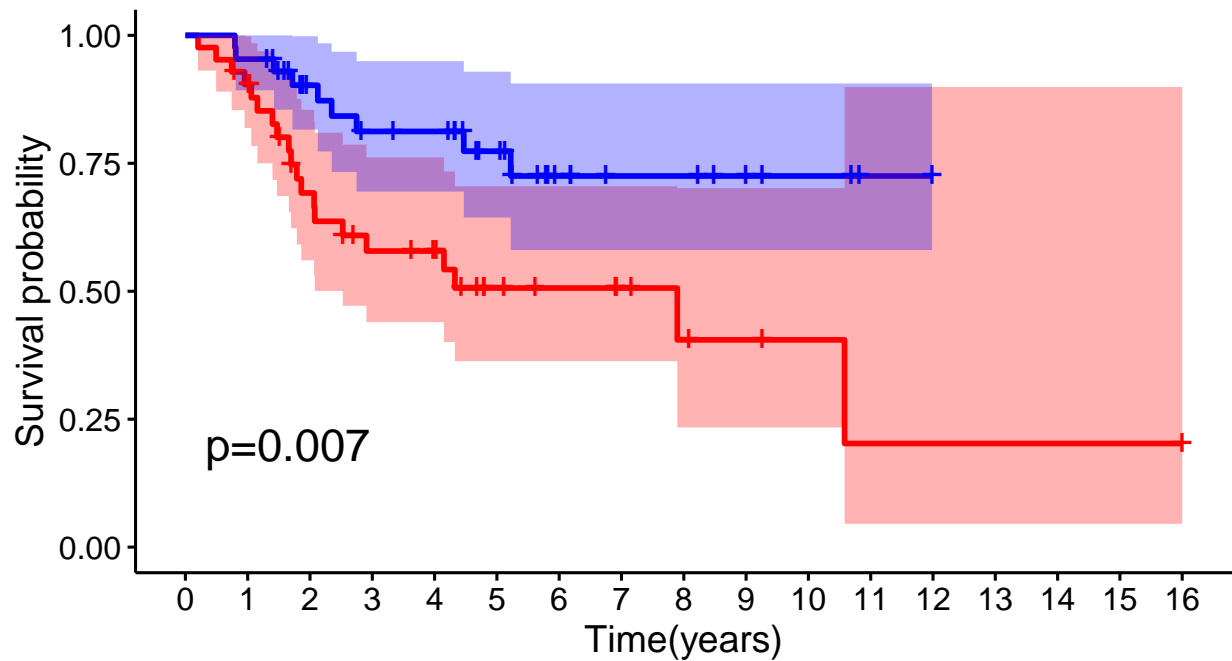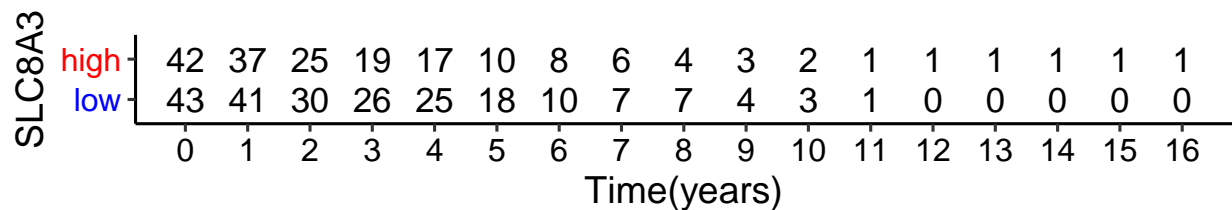

Supplement: Supplementary Document 2 — Kaplan-Meier curve of the 85 malignant genes associated with survival. [file DataSheet_2.zip › Supplementary Document 2/sur.SLC8A3.pdf]

SQLE + high + low

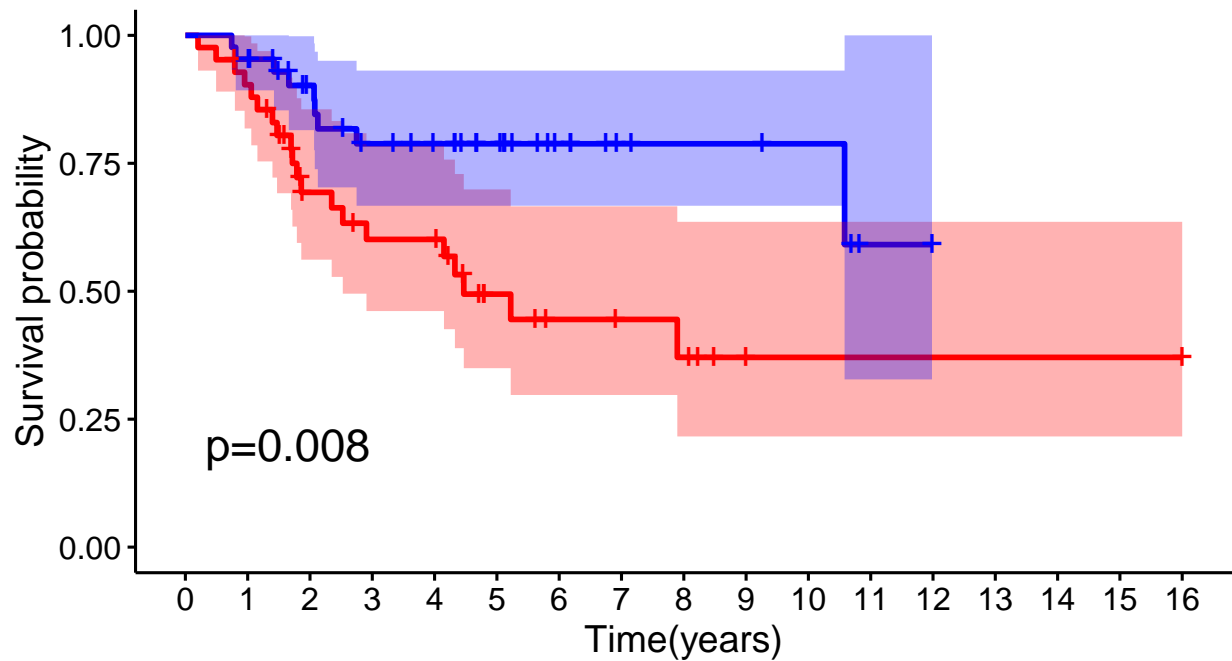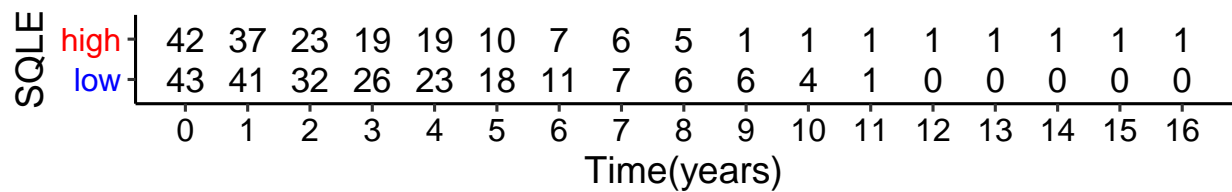

Supplement: Supplementary Document 2 — Kaplan-Meier curve of the 85 malignant genes associated with survival. [file DataSheet_2.zip › Supplementary Document 2/sur.SQLE.pdf]

SYT12 + high + low

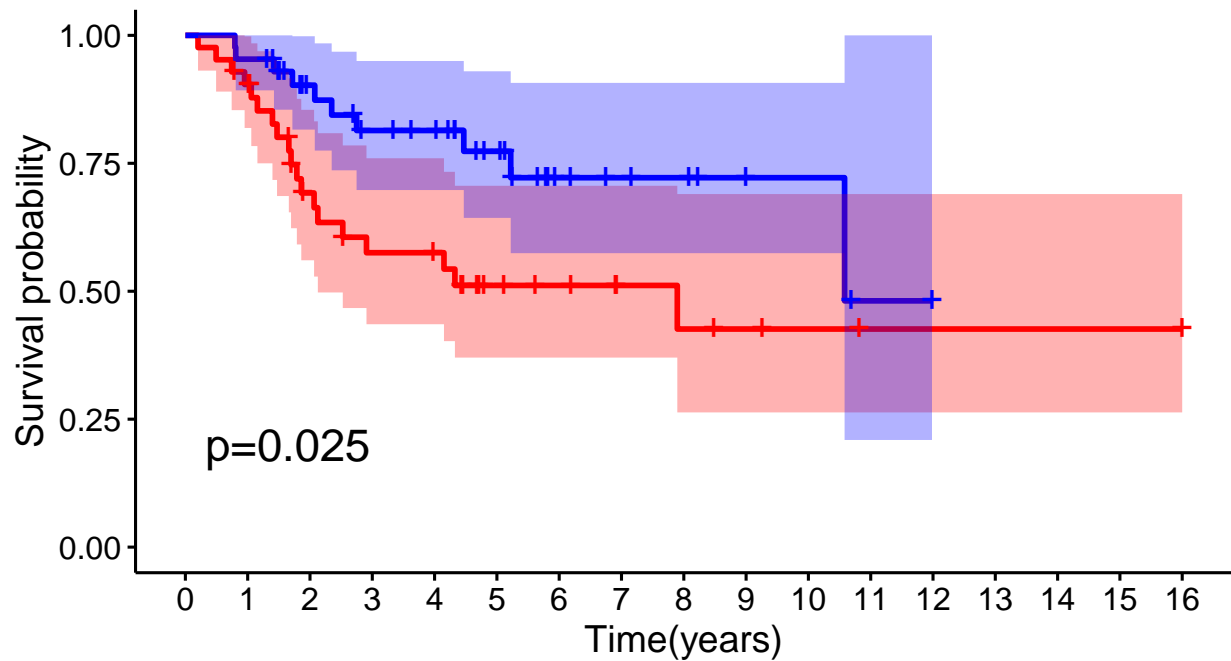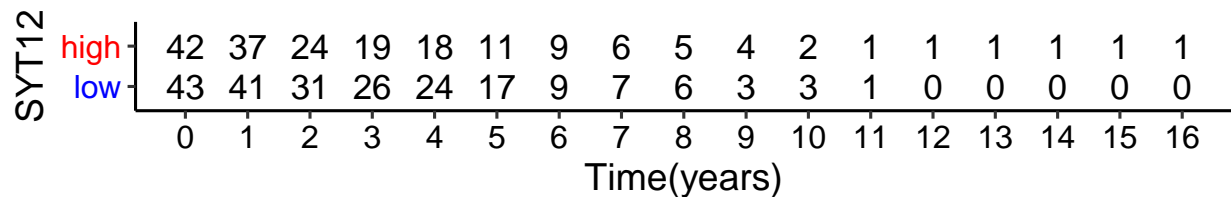

Supplement: Supplementary Document 2 — Kaplan-Meier curve of the 85 malignant genes associated with survival. [file DataSheet_2.zip › Supplementary Document 2/sur.SYT12.pdf]

TBRG1 + high + low

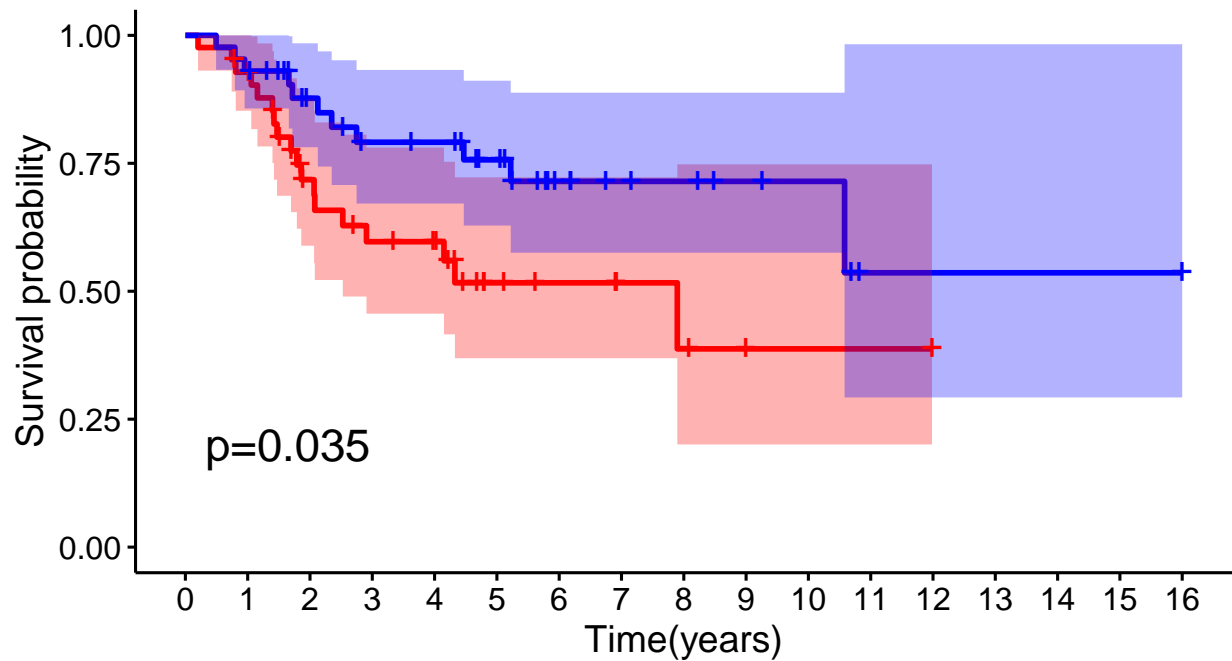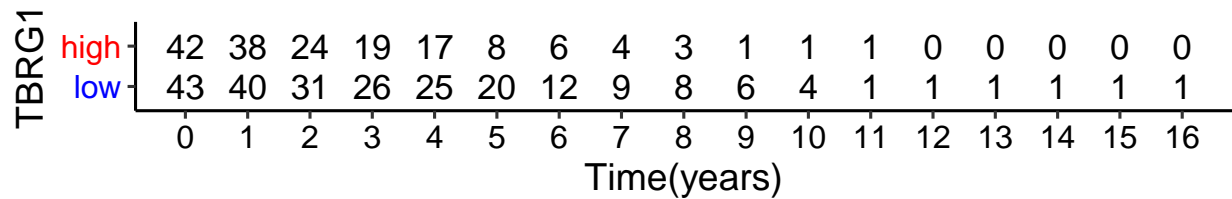

Supplement: Supplementary Document 2 — Kaplan-Meier curve of the 85 malignant genes associated with survival. [file DataSheet_2.zip › Supplementary Document 2/sur.TBRG1.pdf]

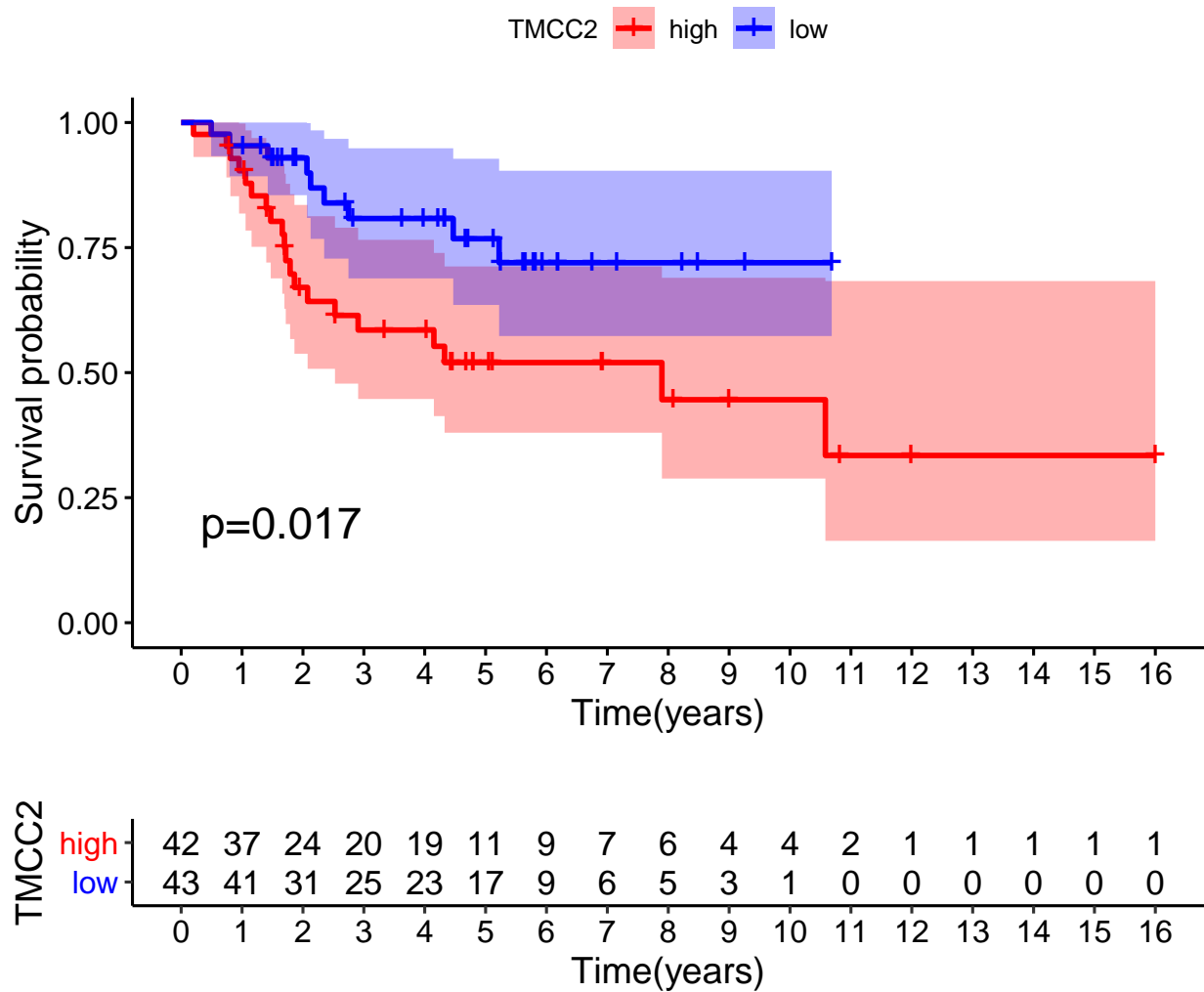

Supplement: Supplementary Document 2 — Kaplan-Meier curve of the 85 malignant genes associated with survival. [file DataSheet_2.zip › Supplementary Document 2/sur.TMCC2.pdf]

TP53I11 + high + low

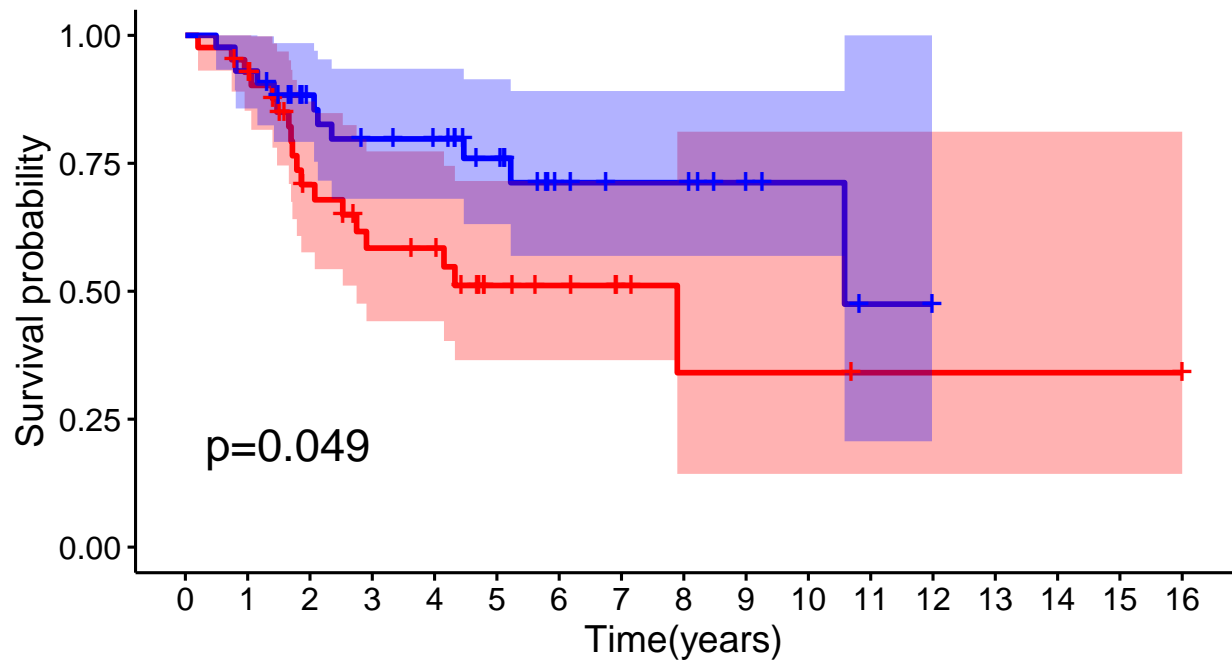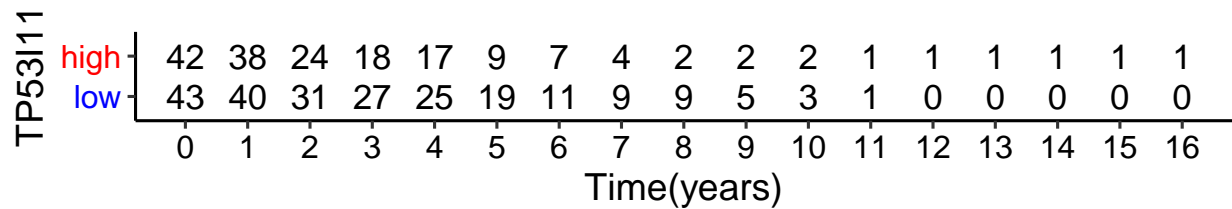

Supplement: Supplementary Document 2 — Kaplan-Meier curve of the 85 malignant genes associated with survival. [file DataSheet_2.zip › Supplementary Document 2/sur.TP53I11.pdf]

TRIM21 high low

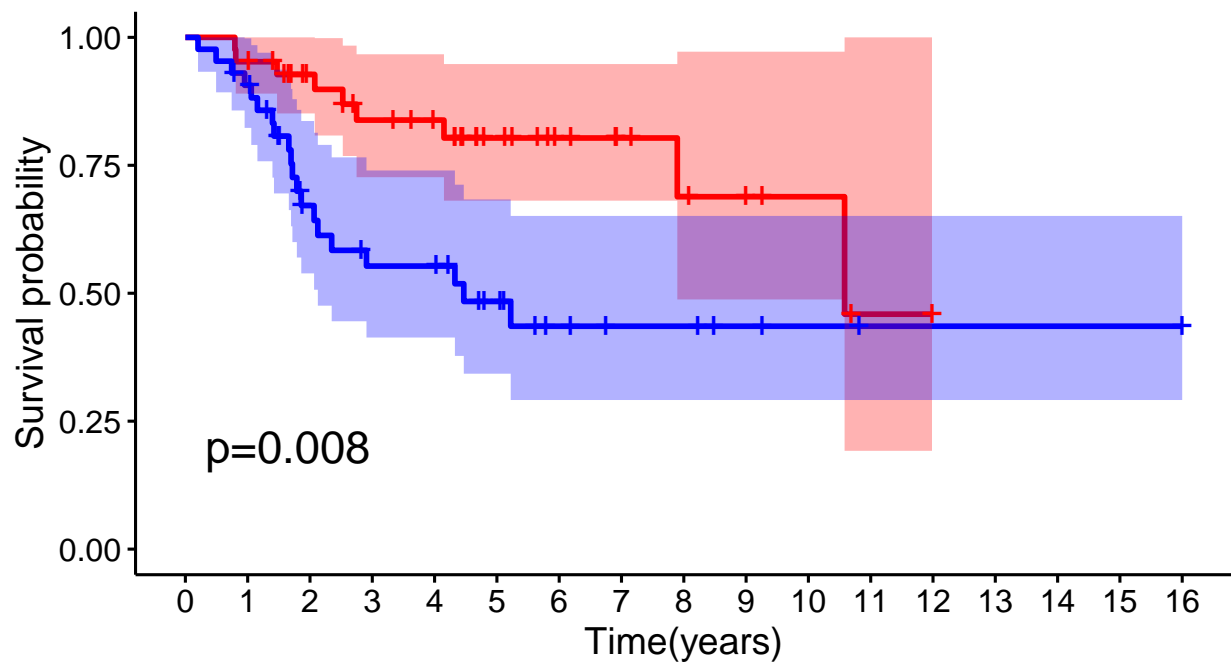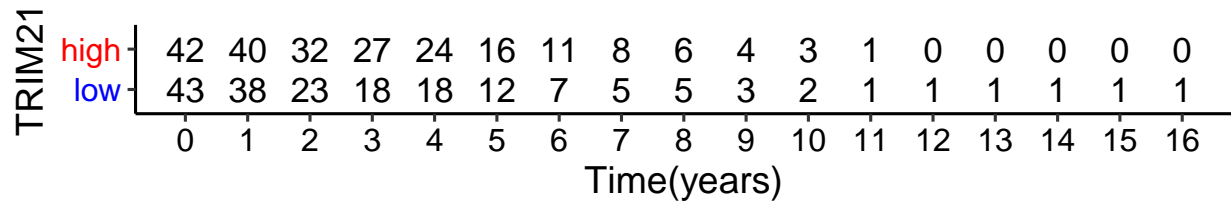

Supplement: Supplementary Document 2 — Kaplan-Meier curve of the 85 malignant genes associated with survival. [file DataSheet_2.zip › Supplementary Document 2/sur.TRIM21.pdf]

WDR66 high low

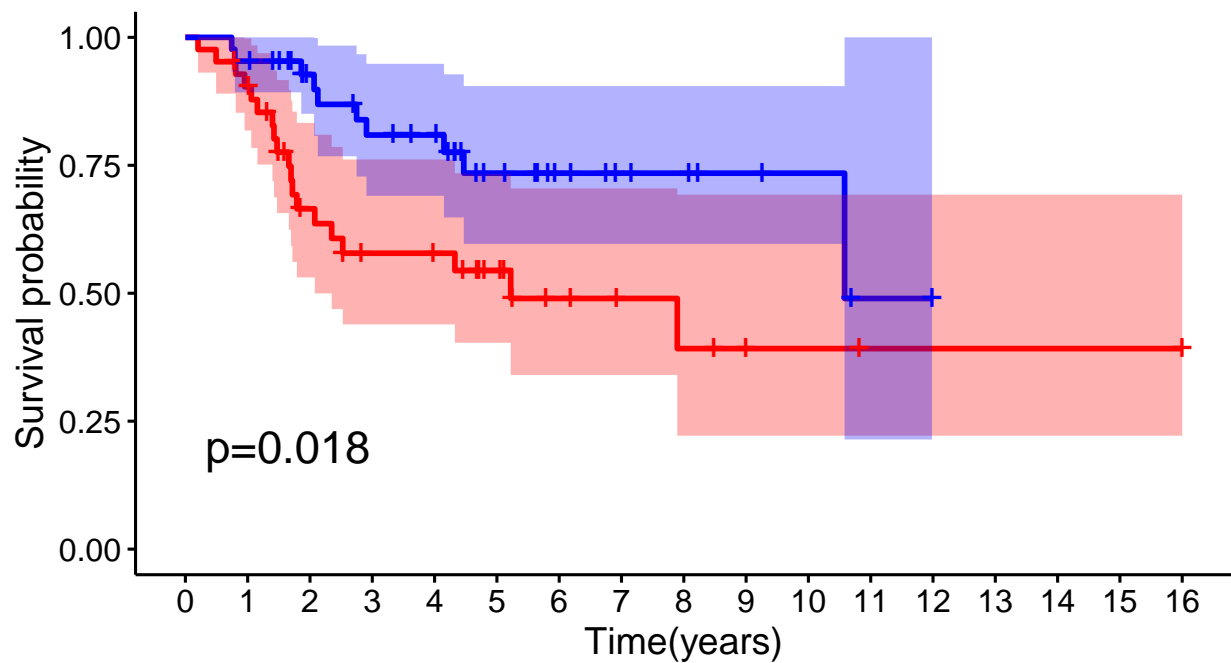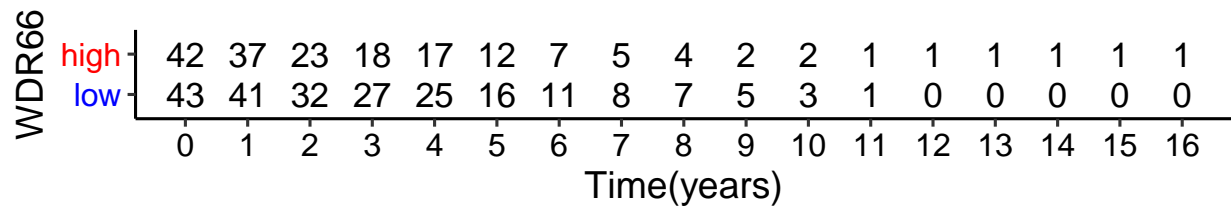

Supplement: Supplementary Document 2 — Kaplan-Meier curve of the 85 malignant genes associated with survival. [file DataSheet_2.zip › Supplementary Document 2/sur.WDR66.pdf]
