# Supplementary material for: Single-Cell Transcriptomics Reveals the Complexity of the Tumor Microenvironment of Treatment-Naive Osteosarcoma
Source: Front Oncol. 2021 Jul 21;11:709210. doi: 10.3389/fonc.2021.709210 (PMC8335545; doi:10.3389/fonc.2021.709210)
Supplement: Supplementary file 6 [file Table_1.docx]

**Table S1. Characteristics of the 6 patients with OS included in this study**

| Patient ID | Sex | Age | Neoadjuvant chemotherapy | Tissue samples | Histological diagnosis |
| --- | --- | --- | --- | --- | --- |
| OS1 | Male | 16 | NO | Tumor tissue | Classical osteosarcoma |
| OS2 | Female | 19 | NO | Tumor tissue | Classical osteosarcoma |
| OS3 | Female | 45 | NO | Tumor tissue | Classical osteosarcoma |
| OS4 | Male | 19 | NO | Tumor tissue | Classical osteosarcoma |
| OS5 | Male | 14 | NO | Tumor tissue | Classical osteosarcoma |
| OS6 | Male | 13 | NO | Tumor tissue | Classical osteosarcoma |
