# Supplementary material for: Single-Cell Transcriptomics Reveals the Complexity of the Tumor Microenvironment of Treatment-Naive Osteosarcoma
Source: Front Oncol. 2021 Jul 21;11:709210. doi: 10.3389/fonc.2021.709210 (PMC8335545; doi:10.3389/fonc.2021.709210)
Supplement: Supplementary Document 1 — Kaplan-Meier curve of the 518 genes associated with survival. [file sur.CPA6.pdf › Supplementary Document 1/sur.CPA6.pdf]

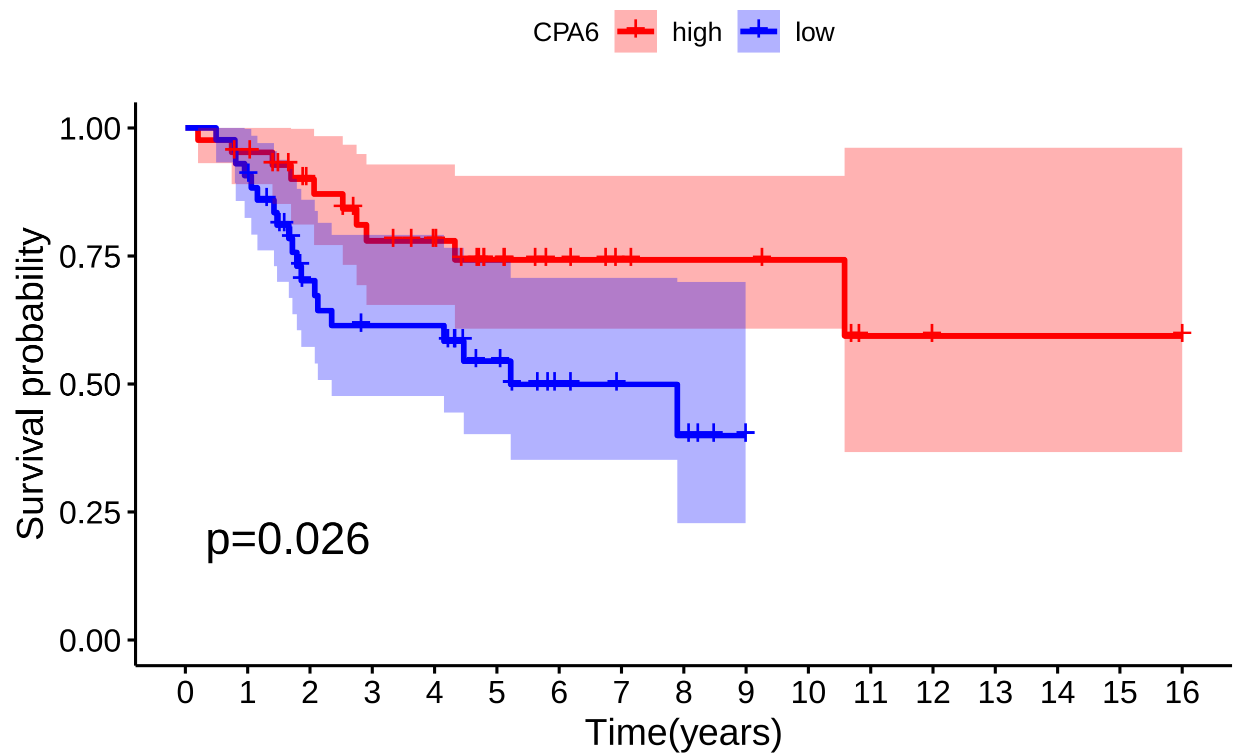

CPA6	0	1	2	3	4	5	6	7	8	9	10	11	12	13	14	15	16
high	42	39	31	25	22	15	11	8	7	7	5	2	1	1	1	1	1
low	43	39	24	20	20	13	7	5	4	0	0	0	0	0	0	0	0

Time(years)
