# Supplementary material for: Single-Cell Transcriptomics Reveals the Complexity of the Tumor Microenvironment of Treatment-Naive Osteosarcoma
Source: Front Oncol. 2021 Jul 21;11:709210. doi: 10.3389/fonc.2021.709210 (PMC8335545; doi:10.3389/fonc.2021.709210)
Supplement: Supplementary Document 2 — Kaplan-Meier curve of the 85 malignant genes associated with survival. [file sur.TM4SF19.pdf › Supplementary Document 2/sur.TM4SF19.pdf]

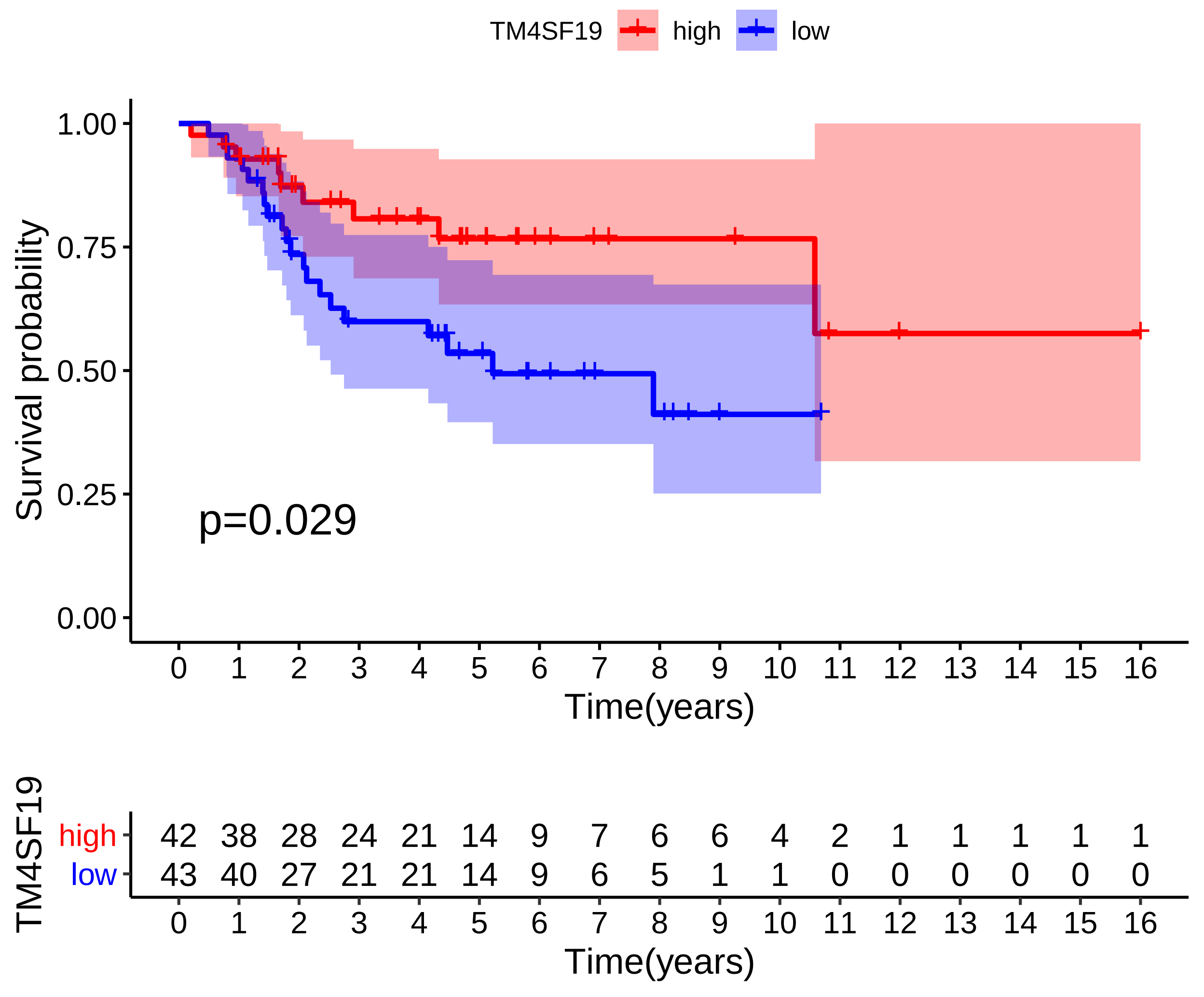
TM4SF19
high
low
1.00
0.75
0.50
0.25
0.00
Survival probability
p=0.029
0
1
2
3
4
5
6
7
8
9
10
11
12
13
14
15
16
Time(years)
TM4SF19
high
low
42 38 28 24 21 14 9 7 6 6 4 2 1 1 1 1 1
43 40 27 21 21 14 9 6 5 1 1 0 0 0 0 0 0
0 1 2 3 4 5 6 7 8 9 10 11 12 13 14 15 16
Time(years)
